# Defect-induced tuning of polarity-dependent adsorption in hydrophobic–hydrophilic UiO-66

**DOI:** 10.1038/s42004-022-00742-z

**Published:** 2022-10-07

**Authors:** Gabriela Jajko, Sofia Calero, Paweł Kozyra, Wacław Makowski, Andrzej Sławek, Barbara Gil, Juan José Gutiérrez-Sevillano

**Affiliations:** 1 Faculty of Chemistry, Jagiellonian University in Kraków, Gronostajowa 2, 30-387 Kraków, Poland; 2 Materials Simulation and Modelling, Department of Applied Physics, Eindhoven University of Technology, 5600 MB Eindhoven, The Netherlands; 3 AGH University of Science and Technology, Academic Centre for Materials and Nanotechnology, al. A. Mickiewicza 30, 30-059 Kraków, Poland; 4 Department of Physical, Chemical and Natural Systems, Universidad Pablo de Olavide, Ctra. Utrera Km. 1, Seville, ES-41013 Spain

**Keywords:** Organic-inorganic nanostructures, Porous materials, Metal-organic frameworks, Computational chemistry

## Abstract

Structural defects in metal–organic frameworks can be exploited to tune material properties. In the case of UiO-66 material, they may change its nature from hydrophobic to hydrophilic and therefore affect the mechanism of adsorption of polar and non-polar molecules. In this work, we focused on understanding this mechanism during adsorption of molecules with different dipole moments, using the standard volumetric adsorption measurements, IR spectroscopy, DFT + D calculations, and Monte Carlo calculations. Average occupation profiles showed that polar and nonpolar molecules change their preferences for adsorption sites. Hence, defects in the structure can be used to tune the adsorption properties of the MOF as well as to control the position of the adsorbates within the micropores of UiO-66.

## Abstract (web summary)

Structural defects in metal–organic frameworks can be exploited to tune material properties. Here, a combined theoretical and experimental study demonstrates that the introduction of defects to UiO-66 alters its nature from hydrophobic to hydrophilic, affecting the adsorption mechanism of polar and non-polar molecules.

## Introduction

Molecular polarity is determined by the dipole moment as a result of nonsymmetric charge distribution^1–3^. Since polarity highly influences adsorption, it is important to model the interactions of polar and nonpolar molecules in hydrophobic and hydrophilic structures properly. These interactions play a key role in many biochemical processes, such as protein folding, where hydrophobic interactions with nonpolar groups contribute to the stability of folded proteins, bases in helical nucleic acids, and membrane layers^4,5^. In this regard, accurate descriptions of interactions between adsorbate molecules and porous solids are easier to achieve for nonpolar molecules than for polar ones. This is because when describing nonpolar molecules the electrostatic part of the model can be neglected, reducing the number of parameters (point charges of the guest molecule or/and of the host structure). Modeling interactions with polar molecules involves the use of coulombic interactions in combination with other potentials to describe van der Waals interactions. Finding a proper set of charges for the structure is crucial and can be a challenging task^6–8^.

Besides, tuning the hydrophobic and hydrophilic character of structures may also have a role in many environmental and catalytic applications^9–11^. There are many possibilities in the use of MOFs as adsorbents. They differ in the ability to adsorb molecules, the pressure at which the pores are filled and the shape of the adsorption isotherm. In the literature, many papers can be found on the effect of functionalization, or the size and morphology of pores on the adsorption of water, which is one of the most important representatives of polar molecules^12–14^. It has also been shown that the adsorption in MOFs is mainly dependent on the morphology and pore size, while modification of the chemical nature of the linker allows tuning the hydrophilicity of the material^15^. However, is some cases the transition from hydrophobic to hydrophilic character may be induced by the introduction of structural defects^16–19^, the addition of salts, or the co-adsorption of water and other polar molecules^20^.

**Fig. 1:**
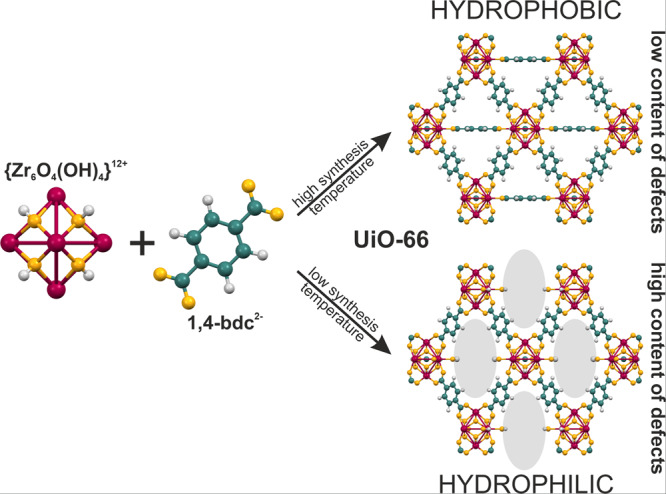
UiO-66 structure with different content of defects. Schematic representation of the influence of the synthesis temperature on the number of defects introduced into the structure and its dependence on the nature of the material.

In this work, we used experimental methodology in combination with computational techniques to model and understand the adsorption of molecules with different dipole moments in the UiO-66 MOF. An additional aspect is the change of the adsorption mechanism of these molecules on the UiO-66 structure as defects are created (Fig. 1). As we know from the previous studies^7,21–23^, UiO-66 may act as either hydrophobic or hydrophilic adsorbent, which alters the adsorption of polar and nonpolar molecules.

## Results and discussion

In this work we used experimental methods such as powder X-ray diffraction (PXRD), volumetric adsorption and IR spectroscopy; and computational ones: DFT + D and Monte Carlo simulations.

### PXRD

The crystal structures of the materials were confirmed by the powder X-ray diffraction (Fig. S1b in the ESI). Symmetry forbidden low-angle reflections in the most defective UiO-66_100 sample were confirmed by modeling and are traced by vertical dashed lines (Fig. S1a in the ESI). Previous studies have shown that the lower the synthesis temperature, the greater the number of structural defects in the form of a missing linker^7,22^. Thus, the material synthesized at 220 °C (UiO-66_200) was assumed to be ideal, while the ones synthesized at 160 °C (UiO-66_160) and 100 °C (UiO-66_100) contained some defects.

### DFT+D calculations

Adsorption in UiO-66 material may occur with a preference for interactions with the metal-oxide zirconium clusters or more favorable interactions with aromatic linker rings, depending on the chemical nature of the adsorbate molecule. To compare the energy preferences of interactions at different adsorption sites, we selected two representatives: polar (methanol) and nonpolar molecules (carbon dioxide). The initial configurations for the molecules were selected on the basis of the preliminary Monte Carlo calculations. We used Density Functional Theory (DFT) to obtain the adsorption energy when adsorbed on the zirconium cluster and on the organic linker. These systems were optimized, and the adsorption energy was calculated as:

$$\triangle {G}_{{ads}}={G}_{{guest}+{host}}-{G}_{{host}}-{G}_{{guest}}$$

where G_guest+host_ is the energy of the adsorbed molecule in the framework, G_host_ is the energy of the framework and G_guest_ is the energy of the molecule. The adsorption energies are summarized in Table 1. Lower adsorption energy, *i.e*. more favorable interactions, can be observed for carbon dioxide adsorbed near the 1,4-bdc linkers. In the case of methanol molecules, the lowest energy occurs in the interactions with the zirconium cluster. This gives the first prerequisite for the preferred adsorption sites of polar and nonpolar molecules.

**Table 1:** Results from DFT+D calculations of adsorption energies.

	ΔG_ads_	
	Metal cluster	Organic linker
Carbon dioxide	1.89	−7.86
Methanol	−7.70	−3.27

Calculated for the adsorption of carbon dioxide and methanol in the vicinity of the metal cluster and organic linkers, respectively. All energies are given in kcal·mol^−1^.

### Adsorption measurements and Monte Carlo calculations

To extend the information obtained from the DFT calculations, we combined experiments with classical simulations to analyze the behavior of polar and nonpolar molecules. First, we studied nonpolar molecules possessing quadrupole moments, then polar molecules, and finally nonpolar molecules without quadrupole. In all cases, we used a set of charges for the structures that was developed to accurately reproduce the adsorption of water in UiO-66 frameworks^7^.

Nitrogen adsorption isotherms at -196 °C (Fig. S2 in the ESI) were performed to determine the textural properties of UiO-66 samples (Table 2). Then, we calculated the adsorption isotherms using Grand Canonical Monte Carlo (GCMC) simulation (Fig. S3 in the ESI). It was found that nitrogen adsorption strongly depends on the presence of defects, but only because of the availability of micropore volume. As shown in the Average Occupation Profiles (AOPs) (Figs. S4 and S5 in the ESI) the available places within the defects are filled at the highest pressures. Fig. S4 depicts the adsorption isotherm for the ideal structure with three points marked on the figure. Then, the AOPs for the selected pressure are shown. Fig. S5 depicts the AOPs at the selected pressure but for all the studied structures (ideal and defective). At low pressures, the interactions in the smaller tetrahedral cages are much stronger, causing the N_2_ molecules to fill these cavities first. Depending on the number of vacancies, saturation loading increases proportionally. We found that the sorption capacity for the experiment and calculations differs, in particular for the comparison of the UiO-66_220 material with the lowest number of defects with the ideal UiO-66_0 computational structure. This discrepancy may be due to the fact that small amount of contaminants may still be present in the pores of the UiO-66_220 sample, even after activation, e.g. unwashed linker residues which occupies the space in the pores and thus reduce the adsorption capacity. Still, even if the absolute values do not match, the trend observed in the experiments is correctly reflected by the simulations.

**Table 2:** Structural properties of the UiO-66 samples.

	UiO-66_220	UiO-66_160	UiO-66_100
PV/cm^3^·g^−1^	0.315	0.410	0.444
BET/m^2^·g^−1^	980	1260	1438

Micropore volume (PV) and surface area (BET) determined from N_2_ adsorption isotherms measures at −196 °C.

**Fig. 2:**
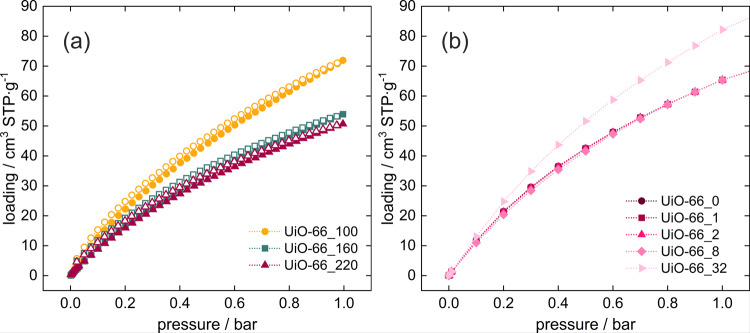
Adsorption isotherms for carbon dioxide in UiO-66. **a** From experiment, **b** from calculations, measured/computed at 27 °C. Closed symbols stand for adsorption, open for desorption.

Carbon dioxide also has a nonzero quadrupole moment, but its adsorption mechanism differs significantly from that observed for nitrogen. At first sight, the presence of low number of defects does not affect the shape of the adsorption isotherm, both in experiments and in simulations in the range 0–1 bar (Fig. 2) for the low number of defects and only from 5 to 20 bars, small differences are noticeable for 8 defects (Fig. S6). However, for the structure with 32 defects the changes are remarkable (Fig. S6) in the whole pressure range studied. Figure 2 shows a good agreement between simulation and experiment, which allows to obtain information about the adsorption of CO_2_ molecules in the UiO-66 framework.

The pore-filling mechanism is shown in the AOPs (Figs. S7 and S8 in the ESI). The molecules of carbon dioxide are mostly adsorbed near the organic linkers, avoiding the metal clusters containing -OH groups (which are the preferential adsorption sites for water molecules^24^). Additional -OH groups are introduced into the structure together with the missing linker defects, but their presence does not affect the adsorption isotherms of carbon dioxide. Despite the increasing available micropore volume, interactions with hydroxyl groups are so unfavorable that carbon dioxide molecules only fill these adsorption sites at very high pressures.

**Fig. 3:**
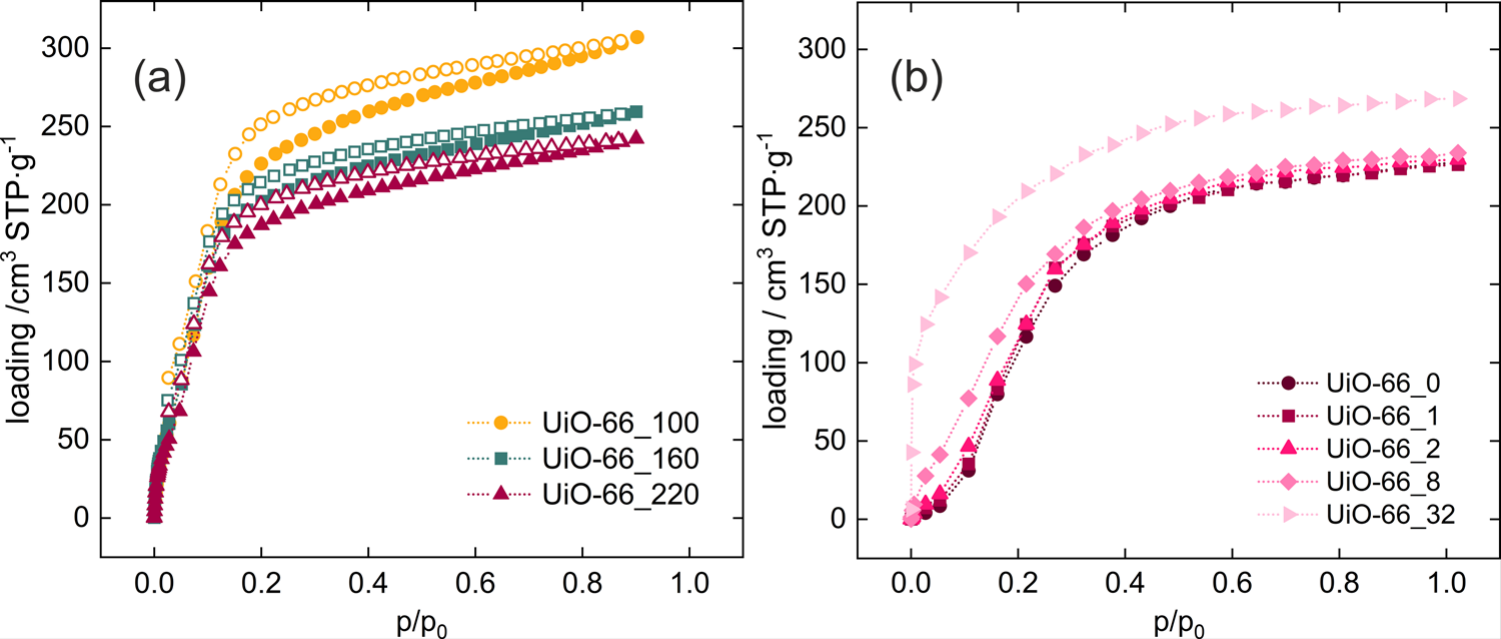
Adsorption isotherms of methanol in UiO-66. **a** From experiment, **b** from calculations, measured/computed at 27 °C. Closed symbols stand for adsorption, open for desorption.

We selected methanol as a representative of a polar molecule to evaluate whether the adsorption mechanism for this molecule is similar to that of the water molecules^7^. In this case, we also found that defective structures adsorb methanol at significantly lower pressures and that the saturation loading is higher (Fig. 3a). The adsorption mechanism was found to be predictable. Most of the uptake takes place on the hydroxyl groups present on the metal clusters and on the created defects, which induces stronger and higher adsorption.

Since in polar methanol molecule electrostatic interactions are important, the set of charges used for our model of UiO-66 have to be verified to ensure they can accurately predict the adsorption mechanism. For this purpose, we calculated the adsorption isotherms with our recent developed set of charges^7^ and with charges derived from EQeq charge method (used in our previous works^25^). Figs. S9 and S10 in the ESI show that our set of charges leads to a better description of the experimental isotherm.

Monte Carlo simulations show that the influence of defects is noticeable (Fig. 3b) – with increased number of missing linkers the adsorption steep shifts from ca. 0.15 to 0.01 p/p_0_, and the adsorption capacity increases from 218 to 268 cm^3^ STP/g. This adsorption mechanism is shown on Average Occupation Profiles in Figs. S11 and S12 of the ESI. The additional -OH groups positioned in of defects attract the methanol molecules already at low pressure. This behavior is most pronounced in the structure with 32 defects. In that case, the adsorption occurs already at a relative pressure of 0.01, resulting in almost instant filling of the voids formed after removal of the organic linkers.

**Fig. 4:**
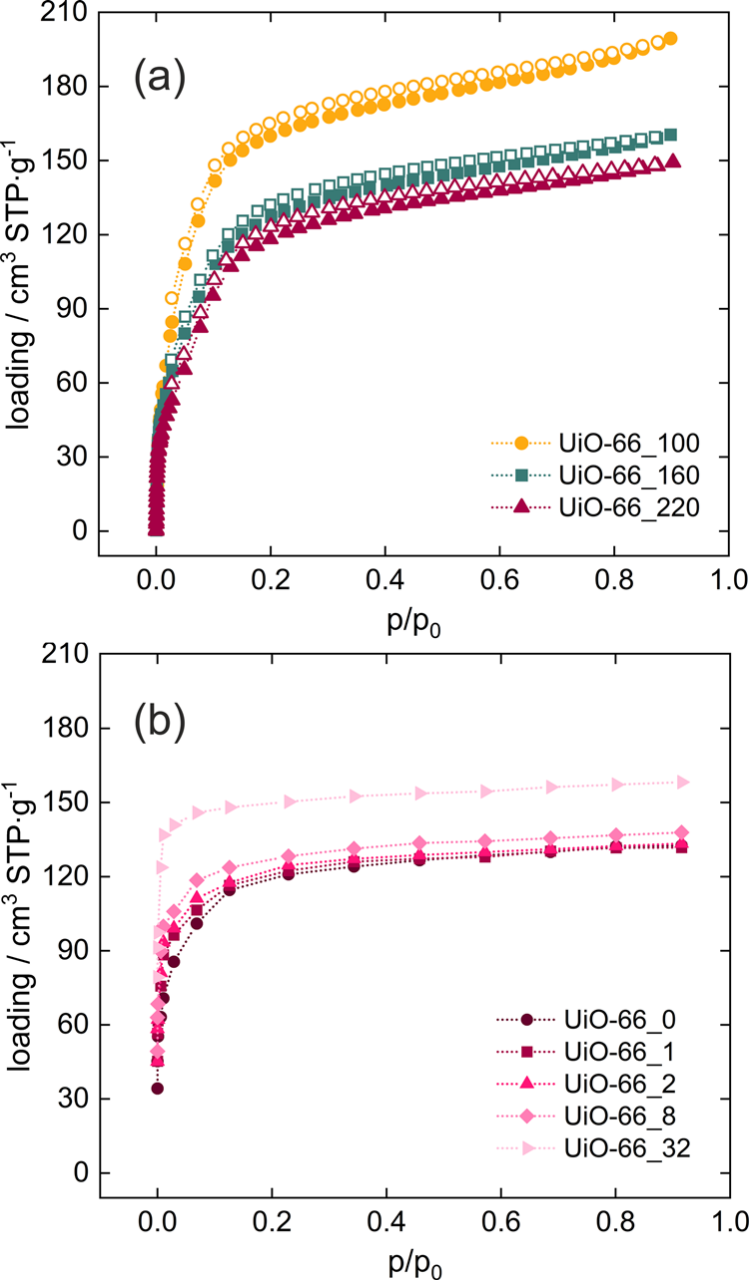
Adsorption isotherms of ethanol in UiO-66. **a** From experiment, **b** from calculations, measured/computed at 27 °C. Closed symbols stand for adsorption, open for desorption.

The adsorption of ethanol is similar to that of methanol (Fig. 4a). As the number of structural defects increases, the adsorption shifts to lower relative pressure, and the adsorption capacity increases. When calculating the isotherm, we found that even with our new set of charges, the calculations underestimated the experimental data. Therefore, it was necessary to modify the Lennard-Jones parameters of the force field to increase the van der Waals interactions between ethanol and UiO-66 framework (Fig. S13, Table S2 in the ESI). The new set of LJ parameters leads to a better agreement with experiment in the and the most defective structures (Fig. S14 in the ESI). Thus calculated adsorption isotherms show a shift from 0.1 to almost 0.0 p/p_0_ with increasing hydrophilicity of the UiO-66 structure (Fig. 4b). The adsorption capacity also increases significantly from 125 cm^3^ STP/g for the defect-free material to 160 cm^3^ STP/g for UiO-66_32. Similarly to the methanol adsorption, ethanol is adsorbed at first near the -OH groups introduced after removal of the linkers, and then fills the other voids in the UiO-66 structures (Figs. S15 and S16 in the ESI).

Methane, as a nonpolar organic molecule, should preferentially adsorb near organic linkers, following the assumption that the nonpolar molecules do not have strong affinity for any adsorption site. This assumption is corroborated by the isotherms calculated up to 80 bars (Fig. S17 in the ESI). They show that introduction of defects, and thus increased hydrophilicity, has no significant effect on methane adsorption. Average Occupation Profiles (Figs. S18 and S19 in the ESI) show that methane molecules adsorb in all accessible volume, with no particular affinity for the metal clusters or the -OH groups.

**Fig. 5:**
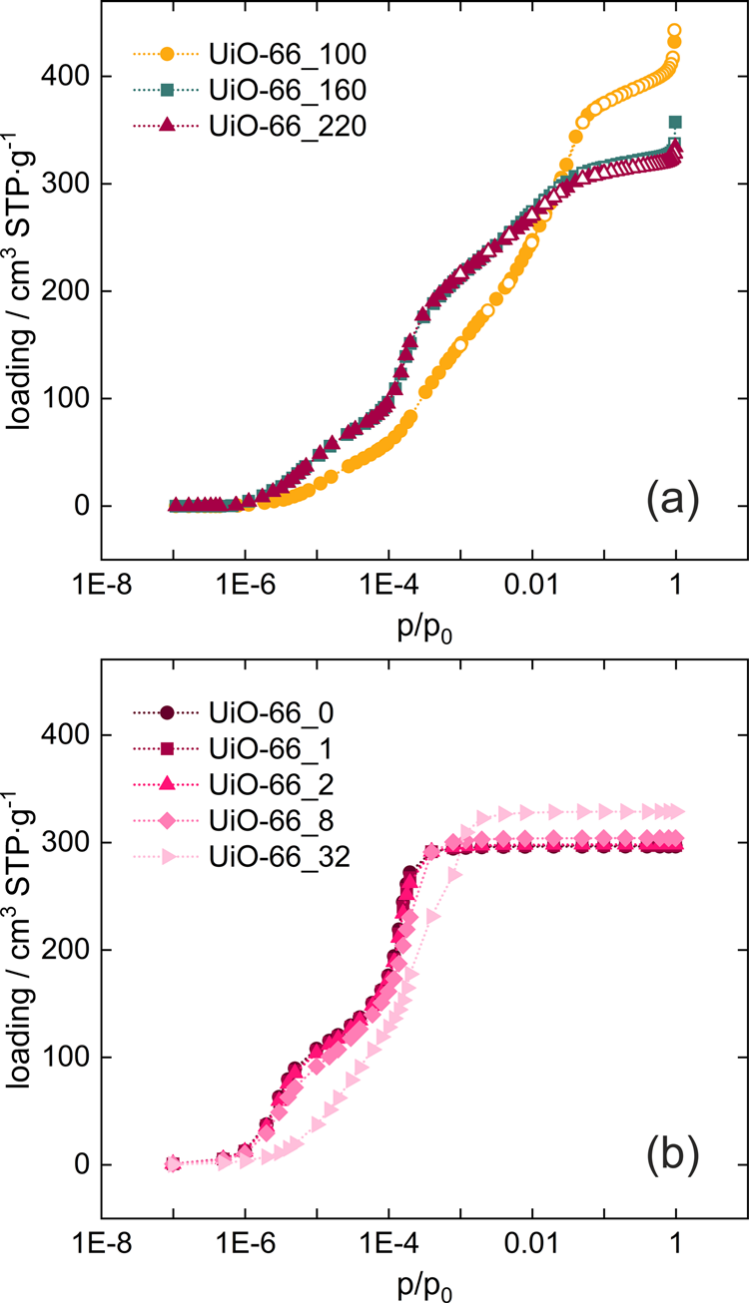
Adsorption isotherms of argon in UiO-66. **a** From experiment^7^, **b** from calculations, measured/computed at −186 °C. Closed symbols stand for adsorption, open for desorption.

Argon, as a relatively small, nonpolar, monoatomic molecule, also has no preferential adsorption sites. The shape of the isotherm depends only on the size and number of pores in the structure (Fig. 5a). As defects are created, Ar molecules fill the pores at a higher relative pressure but to a greater extent. Based on the argon isotherm, we calculated the pore size distributions of the studied materials, which may be found also in our previous work^7^ (Fig. S20 in the ESI). On this basis, two types of micropores at 7 Å and 9 Å can be identified, which can correspond to the two types of cavities present in the UiO-66 structure for samples _160 and _220. For sample UiO-66_100, the pore size distribution differs from the others, showing larger micropores (7.5–11 Å and 17 Å).

We performed Monte Carlo simulations to trace the mechanism of argon adsorption in the pores of UiO-66 (Fig. 5b), as it was done for other molecules. Due to material specificity, the simulations do not accurately reflect the experiment (Figure S21 in the ESI), but the trend is kept. AOPs based on the calculations help to understand the adsorption mechanism (Figs. S22 and S23 in the ESI). Argon is frequently used as a probe molecule to obtain the distribution of micropores in a structure. Argon fills first the smaller tetrahedral cages, then the corners of the octahedral cages and finally the voids created by linkers removal. In this case, AOPs do not provide a clear picture of the sorption mechanism, and only in the structure with 32 defects is it possible to see some “blurring” where linkers are missing.

In addition to the adsorption isotherms, we calculated the heats of adsorption for all studied molecules using the Widom particle-insertion method at zero coverage. The heats of adsorption change significantly with the dipole moment of the adsorbates. (Table 3). For nonpolar molecules (methane and argon) and quadrupole molecules (nitrogen and carbon dioxide) the heat of adsorption is lower as the structure becomes more hydrophilic due to the increasing number of defects. For the two polar molecules (methanol and ethanol) the heats of adsorption are the highest in the most hydrophilic structure UiO-66_32 and the lowest in the most hydrophobic UiO-66_0. This is a consequence of the adsorption of the polar molecules next to the -OH groups incorporated after the removal of the linkers. Therefore, it can be concluded that these sites are even more attractive than the initial adsorption sites on defect-free UiO-66, *i.e*. in the vicinity of the zirconium clusters. This is in line with the previous conclusions drawn directly from adsorption isotherms: the lower the adsorption onset, the higher the heat of adsorption. In the case of nonpolar molecules and molecules with quadrupole moment, the heat of adsorption shows little change with increasing number of structural defects, since there are no significant electrostatic interactions with the framework.

**Table 3:** Heats of adsorption of polar and nonpolar molecules in UiO-66 structures.

	UiO-66_0	UiO-66_1	UiO-66_2	UiO-66_8	UiO-66_32
Argon	13.63	13.62	13.61	13.58	13.33
Methane	14.12	14.09	14.05	13.81	12.33
Nitrogen	15.06	14.81	14.80	14.76	14.58
Carbon dioxide	20.06	19.85	19.83	19.67	19.13
Methanol	25.88	31.80	41.83	44.08	54.05
Ethanol	62.41	62.49	62.64	66.76	81.24

All data were calculated for 2×2×2 super-cells, and are given in kJ·mol^−1^.

### FT-IR spectroscopy

**Fig. 6:**
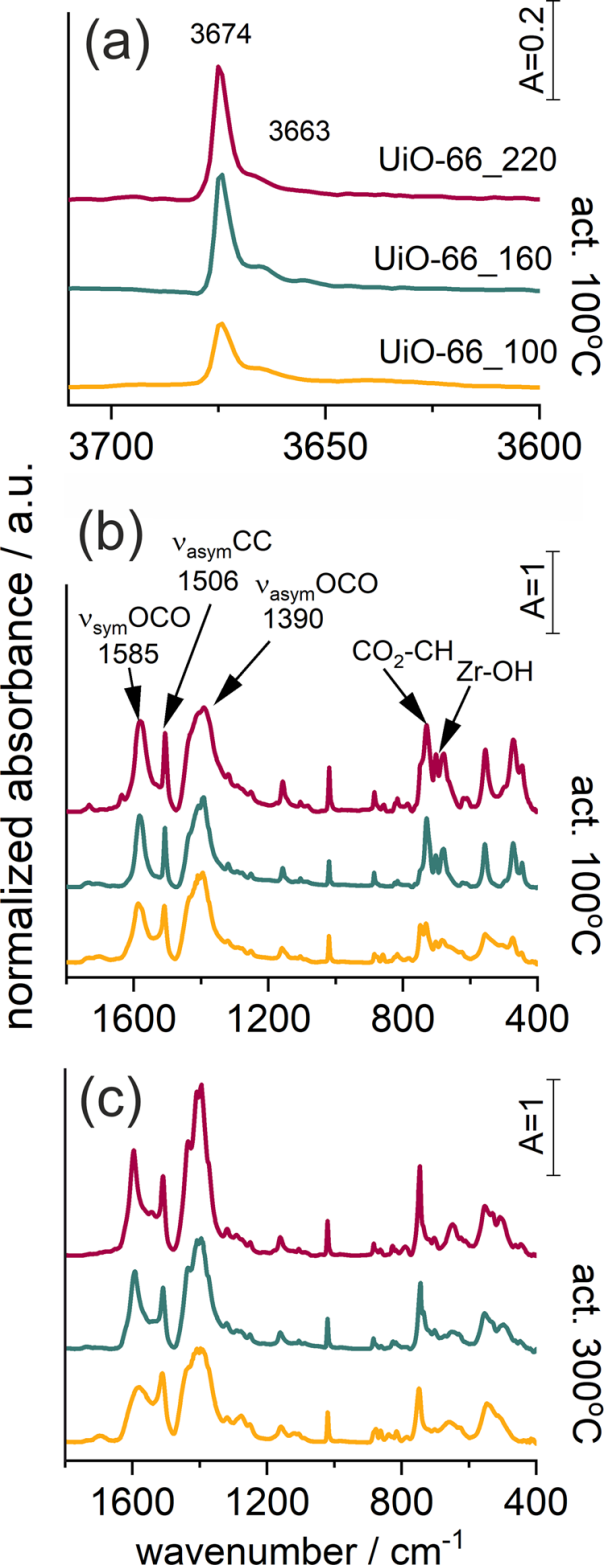
IR spectra of UiO-66 material. **a** The range of OH groups, **b** the range of skeletal vibrations, both for sample activated at 100°C; **c** the range of skeletal vibrations for sample activated at 300 °C. Normalized to the same band height of 1020 cm^−1^.

The activation process (heating at 100 and 300 °C) and adsorption of polar methanol and nonpolar carbon dioxide was followed by in situ FT-IR spectroscopy. Two activation temperatures were chosen to observe the changes in the organic linker and of the inorganic unit caused by thermal treatment (Fig. 6). Activation at 300 °C caused changes in the skeletal vibration, indicated by the disappearance of the OH groups located in the zirconium cluster (IR bands at 730 and 680 cm^−1^) and in coordination of the linker, observed as the increase of the 745 cm^−1^ maximum characteristic of CO_2_-CH vibrations (Fig. 6 and more detailed Figure S24).

Dehydration and dehydroxylation caused by thermal treatment can be observed on the IR spectra in the range of O-H stretching vibrations (Fig. 6 and Fig. S25). After activation at 100 °C two maxima can be observed at 3674 cm^−1^ and 3663 cm^−1^. The former is characteristic of isolated -OH groups attached to the metal cluster (the bending vibrations seen at 730 and 680 cm^−1^). The position of the bands was confirmed by DFT+D calculations of the Vibrational Analysis performed with the DMol3 software, where the band characteristic of isolated -OH groups appears at 3700 cm^−1^, asymmetric OZrO at 747 cm^−1^ and bending Zr-OH at 682 cm^−1^. Additional maxima seen in the OH region, at 3663 cm^−1^, may be assigned to the interaction of OH groups with residual water molecules in very small amount. This assignment was based on the analysis of the IR spectra recorded during dehydration of the UiO-66_220 sample (Fig. S26 in the ESI). Both maxima disappear after activation at 300 °C indicating complete sample dehydroxylation.

Carbon dioxide was adsorbed in hydroxylated and dehydroxylated UiO-66 samples to confirm that (as observed in DFT+D, DMol3 calculations) CO_2_ molecule adsorbs preferentially near the zirconium cluster for the hydroxylated sample, and on the organic linkers for the dehydrated and dehydroxylated one. Both structures were optimized using DFT+D (DMol3) and vibration analysis was performed to identify the IR bands: 2366 cm^−1^ was assigned to carbon dioxide adsorbed on organic linkers, and 2353 cm^−1^ to carbon dioxide near the OH group located on the zirconium linker.

The frequencies observed in the IR spectra and recorded during carbon dioxide adsorption are shifted (Fig. S27 in the ESI) to 2340 and 2336 cm^−1^, characteristic of CO_2_··OH and CO_2_··linker, respectively. There is a third carbon dioxide band present in the spectra, at 2328 cm^−1^, which may be assigned to perturbed CO_2_ structures formed due to co-adsorption with other components such as water^26^.

**Fig. 7:**
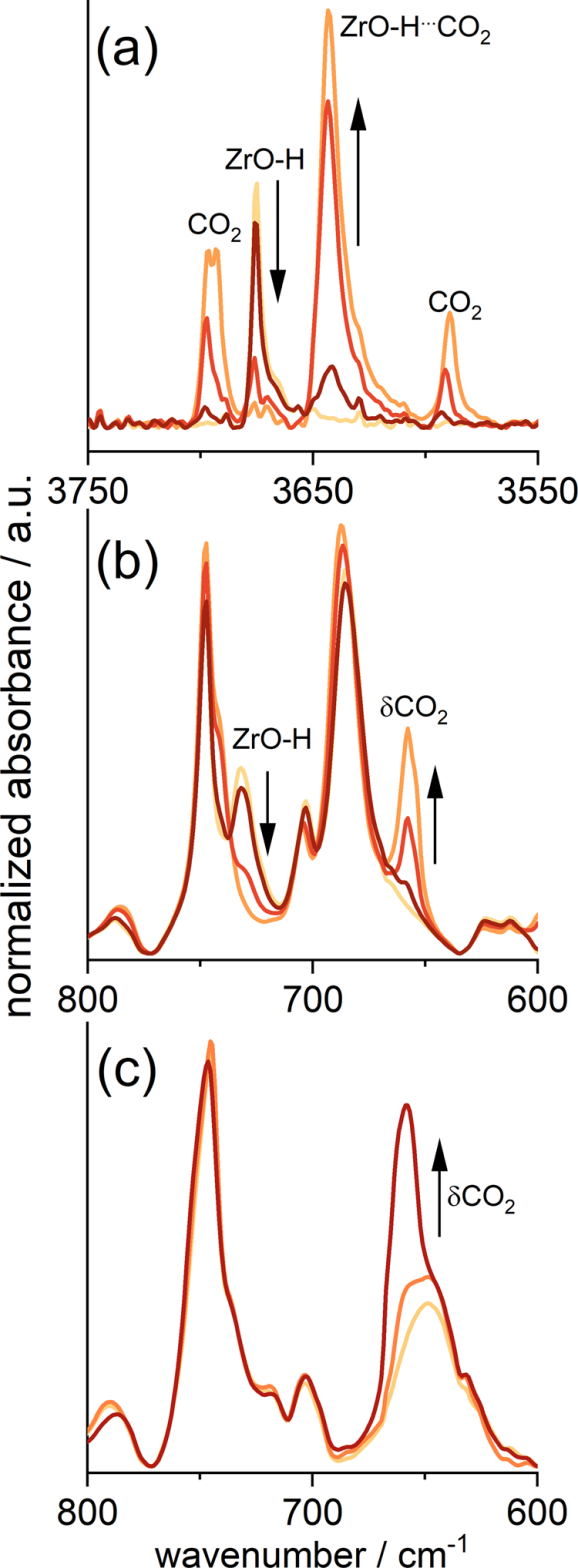
IR spectra of carbon dioxide adsorbed in UiO-66_220. **a** The range of OH groups, **b** the range of skeletal vibrations, both for sample activated at 100 °C; **c** the range of skeletal vibrations for sample activated at 300 °C.

Carbon dioxide adsorption affects the vibrations of the MOF functional groups (Fig. 7). The bands at 3675 and 730 cm^−1^, characteristic of OH vibrations near the zirconium cluster (stretching and bending vibrations, respectively), disappear after carbon dioxide adsorption, and new bands are observed – at 3640 cm^−1^ (red-shifted OH vibrations) and 658 cm^−1^ (CO_2_ bending vibrations). The latter band position is constant, independently of carbon dioxide adsorption on the cluster or on the linker The results, presented here for UiO-66_220 are qualitatively the same for all other UiO-66 samples.

**Fig. 8:**
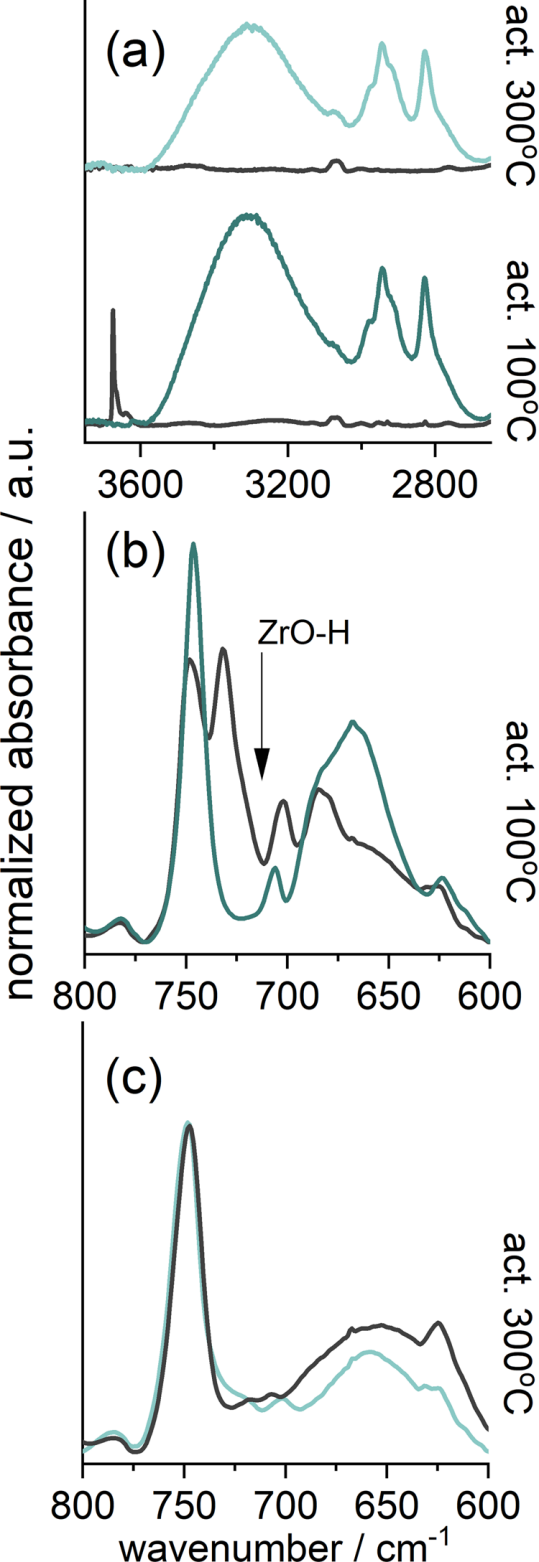
IR spectra of methanol adsorbed in UiO-66_160. **a** The range of OH groups for sample activated at 100 °C and 300 °C; the range of skeletal vibrations for sample activated at 100 °C (**b**) and 300 °C (**c**). The gray line indicates the state before adsorption.

Methanol, as the example of polar molecules, was adsorbed on OH-terminated and dehydroxylated structures (Fig. 8) and followed with FT-IR spectroscopy. For comparison with experiment, models analogous to those of carbon dioxide (with adsorption on linkers and metal clusters) were created and optimized and vibrational analysis was calculated to confirm and identify the IR bands.

Analysis of the spectra confirms that methanol adsorbs strongly on both the cluster -OH groups and the linker. The IR spectra are dominated by the wide band of hydrogen-bonded methanol molecules (at 3310 cm^−1^) resulting mostly from the interaction between adjacent methanol molecules. The -OH groups, present in the sample activated at 100 °C, are also hydrogen-bonded to methanol. After adsorption, the bands of free -OH at 3675 and 730 cm^−1^ disappear.

Adsorption of nonpolar molecules (argon and methane) was also performed and, according to the calculation, no specific adsorption site was found. The spectra in the skeletal vibrations were the same before and after adsorption and the molecules remained symmetric and therefore IR invisible.

## Conclusion

We analyzed the effect of the adsorption of polar, nonpolar and quadruple molecules on UiO-66 structures with changing hydrophobic to hydrophilic character. Using a combination of experiments and calculations, we obtained a molecular-level view of the processes involved. It has been found that, in the case of the nitrogen molecule, the available sites within the cavities are filled only at the highest pressure, governing by the strongest guest-host interactions in smaller tetrahedral cages. For methane and argon, adsorption takes place on all available sites, with no preference for being closer to the metal clusters or -OH groups. In the case of carbon dioxide, the presence of a small number of defects does not significantly affect the shape of the adsorption isotherm, and molecules adsorb mainly on the organic linkers, avoiding adsorption sites close to metal clusters containing -OH groups. For polar molecules such as methanol and ethanol, the mechanism is analogous to that of water. The additional -OH groups introduced at the defect sites attract the molecules, resulting in adsorption at the lowest pressure.

In conclusion, switching the properties of the material from hydrophobic to hydrophilic has no major effect on the adsorption of nonpolar molecules such as argon or methane. However, if the nonpolar molecule has a nonzero quadrupole moment, as is the case for carbon dioxide and nitrogen, the high-pressure adsorption varies significantly as the amount of structural defects increases. In the case of polar methanol and ethanol molecules, increased hydrophilicity significantly affects adsorption, with a shift of the isotherm onset towards lower pressure values and an increase in adsorption capacity. The use of FT-IR spectroscopy correlated with DFT+D (DMol3) calculations allowed preliminary estimates of the preferential adsorption sites for carbon dioxide and methanol.

The preferred adsorption sites of molecules with different polarities were also determined from an atomistic point of view, together with the increase of the hydrophilicity of the structure. Monte Carlo simulations showed that the selection of the set of point charges for the structure plays a key role in accurately describing the adsorption of polar molecules in UiO-66. In this regard, the new set of charges proposed by us results in better agreement with experiments than the EQeq charges previously used in the literature. For the ethanol molecule, a new set of LJ parameters for the guest-host interaction was also needed to reproduce the experimental results.

## Experimental

All chemicals and solvents were purchased from commercial sources (Merck, Avantor) and were used without further purification. UiO-66 samples, with and without defects, were synthesized based on the previously published methods^7,22^. Powder X-ray diffraction (PXRD) patterns were recorded at room temperature (25 °C) on a Rigaku MiniFlex diffractometer in refection mode with Cu-K_α_ radiation (λ = 0.154 nm) in a 2θ range from 3° to 40° with a 0.02° step and 3°/min scan speed.

Adsorption isotherms were measured using static volumetric Autosorb IQ apparatus (Quantachrome Instruments) at -196 °C for nitrogen, and 27 °C for carbon dioxide, methanol, and ethanol. Adsorption isotherms for argon were taken from our previous work^7^. Prior to measurements, all samples were activated under vacuum for 8 h at 100 °C (2 °C/min ramp). Specific surface areas were determined from N_2_ isotherms with the use of the Brunauer-Emmett-Teller (BET) method, and the pore volumes were obtained using the t-plot method.

FT-IR spectra were recorded on a Bruker Tensor 27 spectrometer, equipped with an MCT detector, working at 2 cm^−1^ resolution. UiO-66 samples were deposited as thin layers on a silicon wafer by evaporating a few drops of the MOF suspension in methanol. The wafer was placed in an IR cell closed with KBr windows. The cell was designed to allow outgassing under vacuum and the adsorption of gases at different temperatures. MOF samples were outgassed in vacuum at 100 or 300 °C for 1 h prior to adsorption of the probe molecules. The adsorption temperatures were: -100 °C for CO_2_ and RT for methanol. All measured spectra were normalized to the same intensity of the 1020 cm^−1^ maximum characteristic of C-H out-of-plane bending vibrations.

To describe the molecules of methanol and ethanol we used the TraPPE force field^27^. For carbon dioxide we used the model and Lennard-Jones (LJ) parameters developed by García-Sánchez et al.^28^, while for nitrogen, argon and methane the models and LJ parameters developed by Martín-Calvo et al.^29^. For alcohols, point charges were placed on the hydrogen, oxygen, and alkyl pseudoatoms near the atom of oxygen. To reproduce the quadrupole moments of carbon dioxide and nitrogen, the negative charge was placed on oxygen and nitrogen atoms and the double-positive charge on the center of mass. This offsets the total charge of the molecules to zero^30^. All these models and sets of LJ parameters are well validated^31^. Details are shown in Tables S1 and S2 in the Supporting information. For the UiO-66 structures, we used ideal and defective models from the previous work^7^. For clarity, we used the same labels, where UiO-66_0 represents the defect-free structure, while UiO-66_1, UiO-66_2, UiO-66_8, and UiO-66_32 have one, two, eight, and thirty-two 1,4-bdc linkers removed in the 2×2×2 supercell.

Crossed guest-guest interactions and guest-host interactions between the adsorbates and the MOFs were calculated with standard Lorentz-Berthelot mixing rules. Effective potentials were cut and shifted at a cut-off distance of 12 Å. The Lennard-Jones parameters for the framework were taken from the DREIDING^32^ force field for all the atoms except for zirconium, which was taken from UFF^33^ (Table S3 in the ESI). Coulombic interactions were calculated with the Ewald summation method with a relative precision of 10^−6^. Partial charges of the framework atoms were taken from the previous work^7^. The full set of charges and parameters for the L-J potentials for guests and host is shown in Tables S3 and S4 of the Supporting information.

Simulations were performed in a simulation box of 2×2×2 unit cells, using periodic boundary conditions^34^. The crystal lattice was treated as rigid during the Monte Carlo calculations. Heats of adsorption and helium void fractions (HVF) were calculated using the Widom particle-insertion method^35^. Pore volumes were obtained from the HVF and framework density. Characteristics of the tested systems are provided in Table S5 in the ESI. The calculations of adsorption isotherms were conducted with the Grand Canonical Monte Carlo (GCMC) method, with fixed chemical potential, volume, and temperature^36^. The pressure is related to the chemical potential by fugacity, using the Peng-Robinson equation of state. Absolute adsorption is related to excess adsorption using the equation from Frenkel et al.^36^: ${\theta }_{{excess}}={\theta }_{{absolute}}-\frac{{pV}}{{zRT}}$, where p, V, T are the pressure, volume and temperature of the system, R is the gas constant, and z is the gas compressibility. Each point on the adsorption isotherm was computed by running 5·10^4^ initialization cycles and 5·10^5^ production cycles of equally probable trial moves translation, rotation, swap, and reinsertion. All simulations were performed using the RASPA code^37,38^.

Quantum-chemical periodic DFT calculations were performed using DMol3 code^39^ using the RPBE^40,41^ functional with correction for dispersion London interactions TS^42^. The orbitals were described using the basis set of DND with DSPP pseudopotentials^43^. The Fermi smearing and the spatial cut-off distance were set to 0.005 Ha and 4.5 Å respectively. The optimization energy convergence was set to 2·10^-5 ^Ha, SCF density convergence to 10^−5 ^Ha, and the maximal atom displacement to 5·10^−3 ^Å. The UiO-66 structure has a large unit cell, so the Γ-point sampling of the irreducible part of the first Brillouin zone (IBZ) and the k-point separation in the reciprocal cell turned out to be sufficient. The entropic contributions were calculated using classic, harmonic vibrational analysis. Gibbs free energies were calculated keeping same temperature as for the IR experiments. The Vibrational Analysis was analyzed using the DMol3 software.

## Supplementary information

Calero_PR File

Supplementary Information

Description of Additional Supplementary Files

Supplementary Data 1

Supplementary Data 2

Supplementary Data 3

Supplementary Data 4

Supplementary Data 5

## Data Availability

Any relevant data are available from the authors upon reasonable request and in the Zenodo repository (10.5281/zenodo.7112787). The cif files of the structures used during the simulations are also available as Supplementary Data files S1-S5 or from CCDC with Deposition Numbers CCDC 2209071-2209075.

## Code availability

RASPA code is free available from the website https://iraspa.org/raspa/. DMol3 code is available from its website.
