# Supplementary material for: Defect-induced tuning of polarity-dependent adsorption in hydrophobic–hydrophilic UiO-66
Source: Commun Chem. 2022 Oct 7;5:120. doi: 10.1038/s42004-022-00742-z (PMC9814431; doi:10.1038/s42004-022-00742-z)
Supplement: Supplementary file 2 — Supplementary Information [file 42004_2022_742_MOESM2_ESM.pdf]

Supporting information to article:

Defect-induced tuning of polarity-dependent adsorption in hydrophobic–hydrophilic UiO-66

Gabriela Jajko [a], Sofia Calero [b,*], Paweł Kozyra [a,*], Wacław Makowski [a], Andrzej Sławek [c], Barbara Gil [a], Juan José Gutiérrez Sevillano [d,*]

[a] Faculty of Chemistry, Jagiellonian University in Kraków, Gronostajowa 2, 30-387 Kraków, Poland
[b] Materials Simulation and Modelling, Department of Applied Physics, Eindhoven University of Technology, 5600 MB Eindhoven, The Netherlands
[c] AGH University of Science and Technology, Academic Centre for Materials and Nanotechnology, al. A. Mickiewicza 30, 30-059 Kraków, Poland
[d] Department of Physical, Chemical and Natural Systems, Universidad Pablo de Olavide, Ctra. Utrera Km. 1, Seville ES-41013,
*corresponding author: s.calero@tue.nl, kozyra@chemia.uj.edu.pl, jjgutierrez@upo.es

Table S1. Models of molecules used for the Monte Carlo simulation.

**CARBON DIOXIDE**			
**Nonbonded interactions**			
(pseudo)atom	ε/k_B [K]	σ [Å]	q [e⁻]
C	29.933	2.745	0.6512
O	85.671	3.017	-0.3256
**Bond lengths**			
$r_{O=C}$ [Å]		$r_{C=O}$ [Å]	
1.15		1.15	
**Bond Angles**			
$\theta_{O=C=O}$ [deg]			
180			
**NITROGEN**			
**Nonbonded interactions**			
(pseudo)atom	ε/k_B [K]	σ [Å]	q [e⁻]
N	38.298	3.306	-0.40484
M	-	-	0.8096
**Bond lengths**			
$r_{N\equiv N}$ [Å]		r_{N-M} [Å]	
1.1		0.55	
**Bond Angles**			
θ_{N-M-N} [deg]			
180			
**METHANOL**			
**Nonbonded interactions**			
(pseudo)atom	ε/k_B [K]	σ [Å]	q [e⁻]
CH_3	98	3.75	0.265
O	93	3.02	-0.7
H	0	0	0.435
**Bond lengths**			
r_{CH3-OH} [Å]		r_{O-H} [Å]	
1.43		0.945	
**Bond Angles**			
$\theta_{CH3-O-H}$ [deg]		k_θ/k_B [K/rad]	
108.5		55400	
**ETHANOL**			
**Nonbonded interactions***			
(pseudo)atom	ε/k_B [K]	σ [Å]	q [e⁻]
CH_3	98	3.75	0
CH_2	46	3.95	0.265

O	93	3.02	-0.7
H	0	0	0.435
**Bond lengths**			
$r_{CH3\text{-}CH2}$ [Å]	$r_{CH2\text{-}OH}$ [Å]	$r_{O\text{-}H}$ [Å]	
1.54	1.43	0.945	
**Bond Angles**			
$\theta_{CH3\text{-}CH2\text{-}O}$ [deg]	k_θ/k_B [K/rad]		
109.47	50400		
$\theta_{CH2\text{-}O\text{-}H}$ [deg]	k_θ/k_B [K/rad]		
108.5	55400		
**Torsion$_{CH3\text{-}CH2\text{-}O\text{-}H}$**			
c_0/k_B [K]	c_1/k_B [K]	c_2/k_B [K]	c_3/k_B [K]
0	209.82	-29.17	187.93
**METHANE**			
**Nonbonded interactions**			
(pseudo)atom	ε/k_B [K]	σ [Å]	q [e$^-$]
CH_4	158.5	3.72	0
**ARGON**			
**Nonbonded interactions**			
(pseudo)atom	ε/k_B [K]	σ [Å]	q [e$^-$]
Ar	124.07	3.38	0

* Unmodified parameters. The increased van der Waals interactions are listed in Table S2.

Nonbonded interactions between guest molecules and the framework were modelled using a Lennard-Jones potential:

$$U^{L-J}(r_{ij}) = 4\varepsilon_{ij}\left[\left(\frac{\sigma_{ij}}{r_{ij}}\right)^{12} - \left(\frac{\sigma_{ij}}{r_{ij}}\right)^{6}\right] + \frac{q_i q_j}{4\pi\varepsilon_0 r_{ij}} \quad \text{(S1)}$$

where r_{ij} is distance between i and j atoms. For each UiO-66_X framework atom and adsorbates (pseudo)atoms ε and σ values from Table S1 were used, which were mixed using Lorentz-Berthelot rules:

$$\varepsilon_{ij} = \sqrt{\varepsilon_i \cdot \varepsilon_j} \quad \text{(S2)}$$

$$\sigma_{ij} = \frac{\sigma_i + \sigma_j}{2} \quad \text{(S3)}$$

Intramolecular bonded interactions within the molecules were also used:

1) Harmonic bonding potential:

$$U^{bond}(r_{ij}) = \frac{1}{2}(r_{ij} - r_{ij}^0)^2 \quad \text{(S4)}$$

2) Harmonic bending potential:

$$U^{bend}(\theta_{ijk}) = \frac{1}{2}(\theta_{ijk} - \theta_{ijk}^0)^2 \quad \text{(S5)}$$

3) Torsions TraPPE cosine series potential:

$$U^{torsion}(\phi_{ijkl}) = p_0 + p_1[1 + \cos\phi_{ijkl}] + p_2[1 - \cos 2\phi_{ijkl}] + p_3[1 + \cos 3\phi_{ijkl}] \quad \text{(S6)}$$

where r_{ij}/r_{ij}^0 is actual/equilibrium bond length, $\theta_{ijk}/\theta_{ijk}^0$ is actual/equilibrium bend angle, and ϕ_{ijkl} is actual dihedral angle.

Considering the underestimation of the interactions for ethanol adsorption, the interactions between (pseudo)atoms and atoms of the UiO-66 framework were appropriately modified using the Lorentz-Berthelot mixing rules. The modified parameters are summarized in Table S2.

Table S2. Modified Lorentz-Berthelot mixing rules for ethanol adsorption in UiO-66.

Ethanol (pseudo)atoms	UiO-66 atoms	ε/k_B [K]	σ[Å]
CH_3	C	136.971	3.612
CH_2		93.842	3.712
O		133.431	3.247
H		none	
CH_3	H	54.761	3.298
CH_2		37.518	3.398
O		53.346	2.933
H		none	
CH_3	O	137.443	3.392
CH_2		94.165	3.492
O		133.891	3.027
H		none	
CH_3	Zr	116.666	3.267
CH_2		79.930	3.367
O		113.651	2.902
H		none	

Table S3. Lennard-Jones parameters for the framework atoms.

Framework atoms	ε/k_B [K]	σ[Å]
Zr	34.7221	2.78317
C	47.86	3.473
O	48.19	3.0331
H	7.65	2.8464

Table S4. Point charges [e^-] for all the atoms in the frameworks.

Framework atoms	UiO-66_0	UiO-66_1	UiO-66_2	UiO-66_8	UiO-66_32
C1	0.529571	0.529571	0.529571	0.529571	0.529571
C2	0.013192	0.013192	0.013192	0.013192	0.013192
C3	-0.137721	-0.137721	-0.137721	-0.137721	-0.137721
H1	0.037378	0.037378	0.037378	0.037378	0.037378
H2	0.241634	0.241634	0.241634	0.241634	0.241634
H3	-	0.450062	0.450030	0.450006	0.450000
O1	-0.739224	-0.739224	-0.739224	-0.739224	-0.739224
O2	-3.300538	-3.300538	-3.300538	-3.300538	-3.300538
O3	-	-0.942727	-0.942727	-0.942727	-0.942727
Zr1	4.503001	4.503000	4.503000	4.503000	4.503000

Table S5. Characteristics of the structures used in this work, where PV is pore volume and HVF is helium void fraction. All parameters are given for a 2x2x2 supercell.

	UiO-66_0	UiO-66_1	UiO-66_2	UiO-66_8	UiO-66_32
HVF	0.5071	0.5084	0.5090	0.5149	0.5395
PV [$cm^3 \cdot g^{-1}$]	0.4071	0.4089	0.4101	0.4194	0.4597
Cell lengths [Å] (a=b=c)	41.4008	41.4008	41.4008	41.4008	41.4008
Cell angles [°] (α=β=γ)	90	90	90	90	90
Framework density [$kg \cdot m^{-3}$]	1246	1243	1241	1228	1174

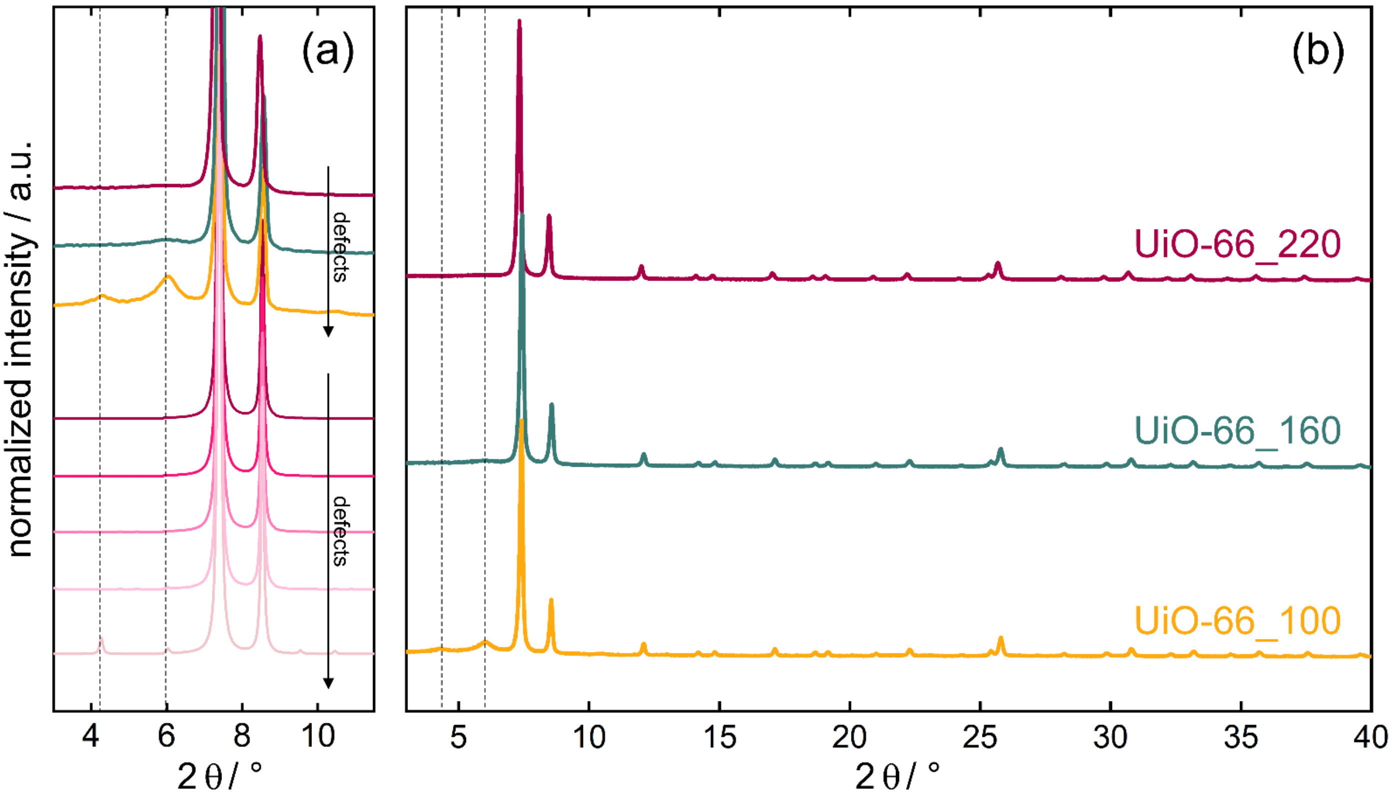

Figure S1. (a) Low-angle range of PXRD patterns for synthesized (top) and calculated (bottom) UiO-66 with different content of defects. (b) PXRD patterns for synthesized UiO-66 structures with different content of defects in the range 2θ = 3 - 40°.

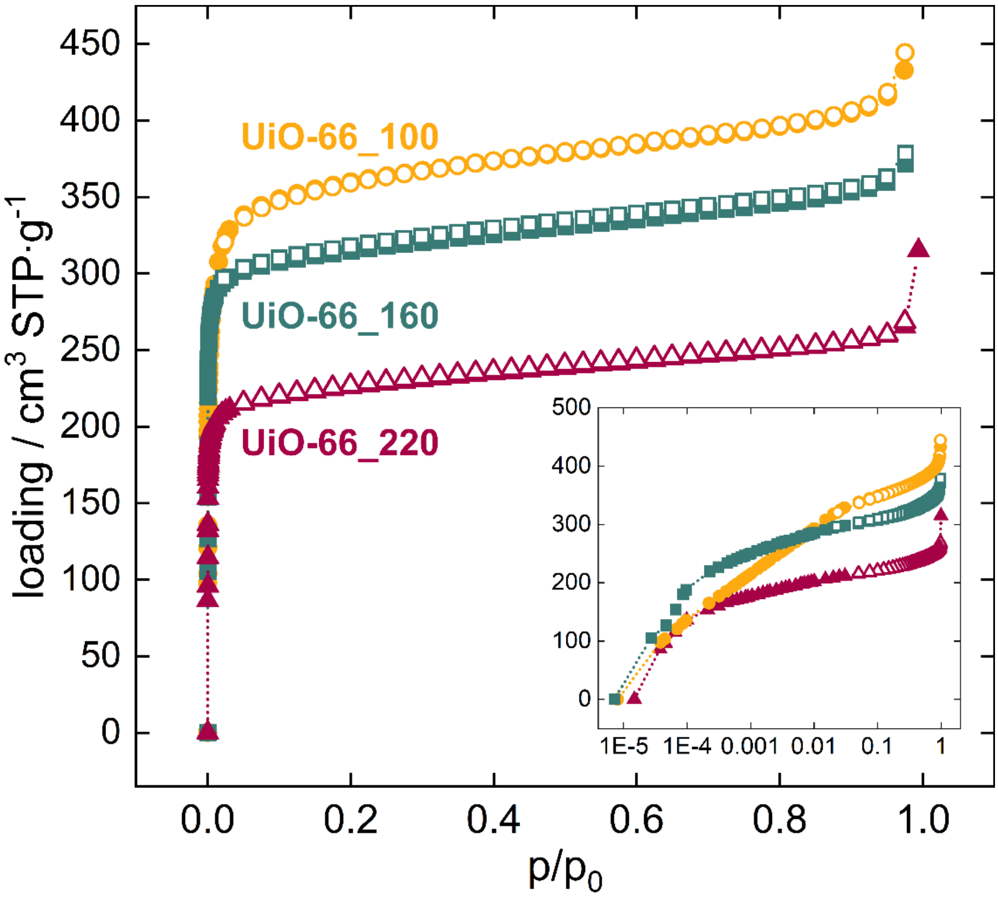

Figure S2. Experimental adsorption isotherms of nitrogen in UiO-66 without and with defects measured at -196°C. Closed symbols represent adsorption and open symbols represent desorption. Inset shows the isotherms in the logarithmic scale.

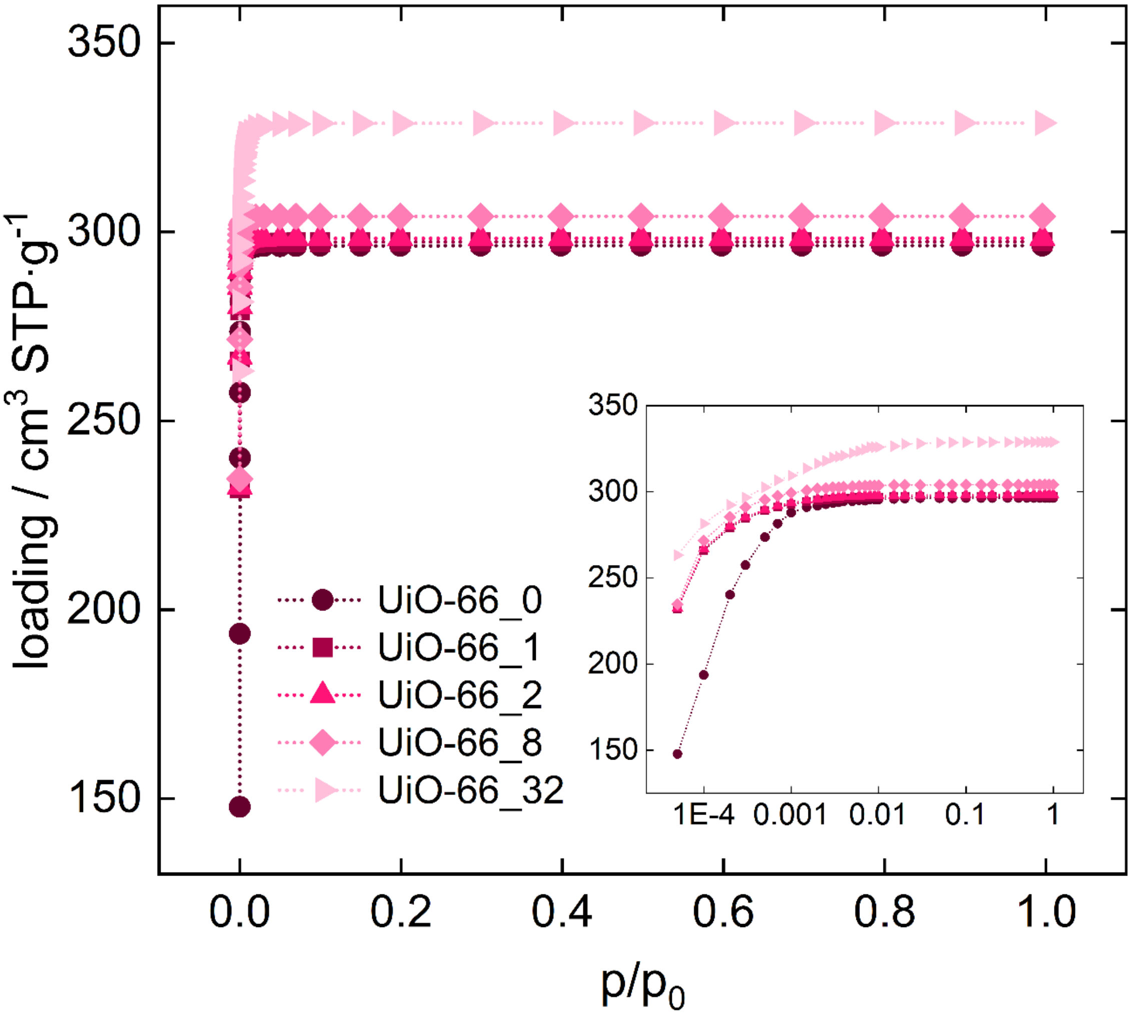

Figure S3. Calculated adsorption isotherms of nitrogen in UiO-66 structures at -196°C. Inset shows the isotherms in the logarithmic scale.

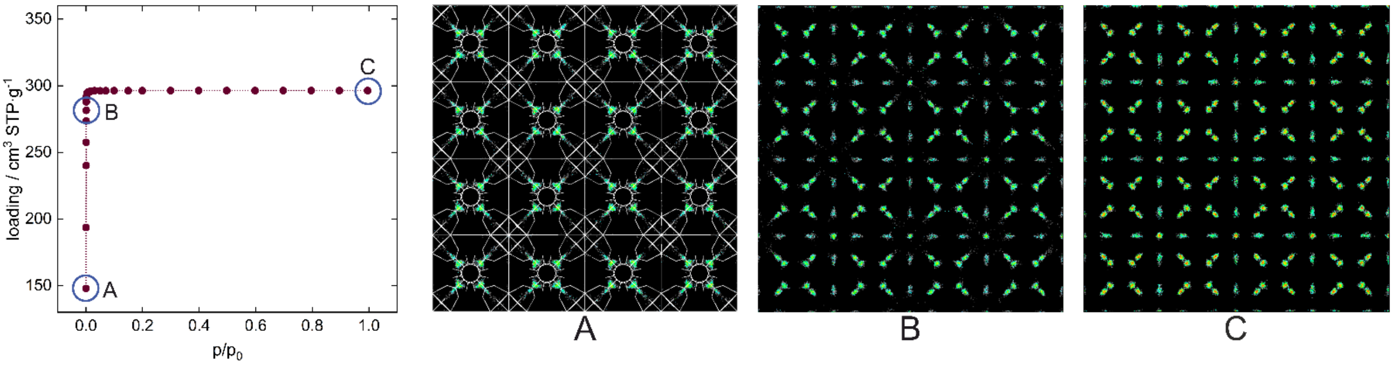

Figure S4. Calculated adsorption isotherm of nitrogen (left) in UiO-66_0 structure together with Average Occupation Profiles simulated at marked pressures.

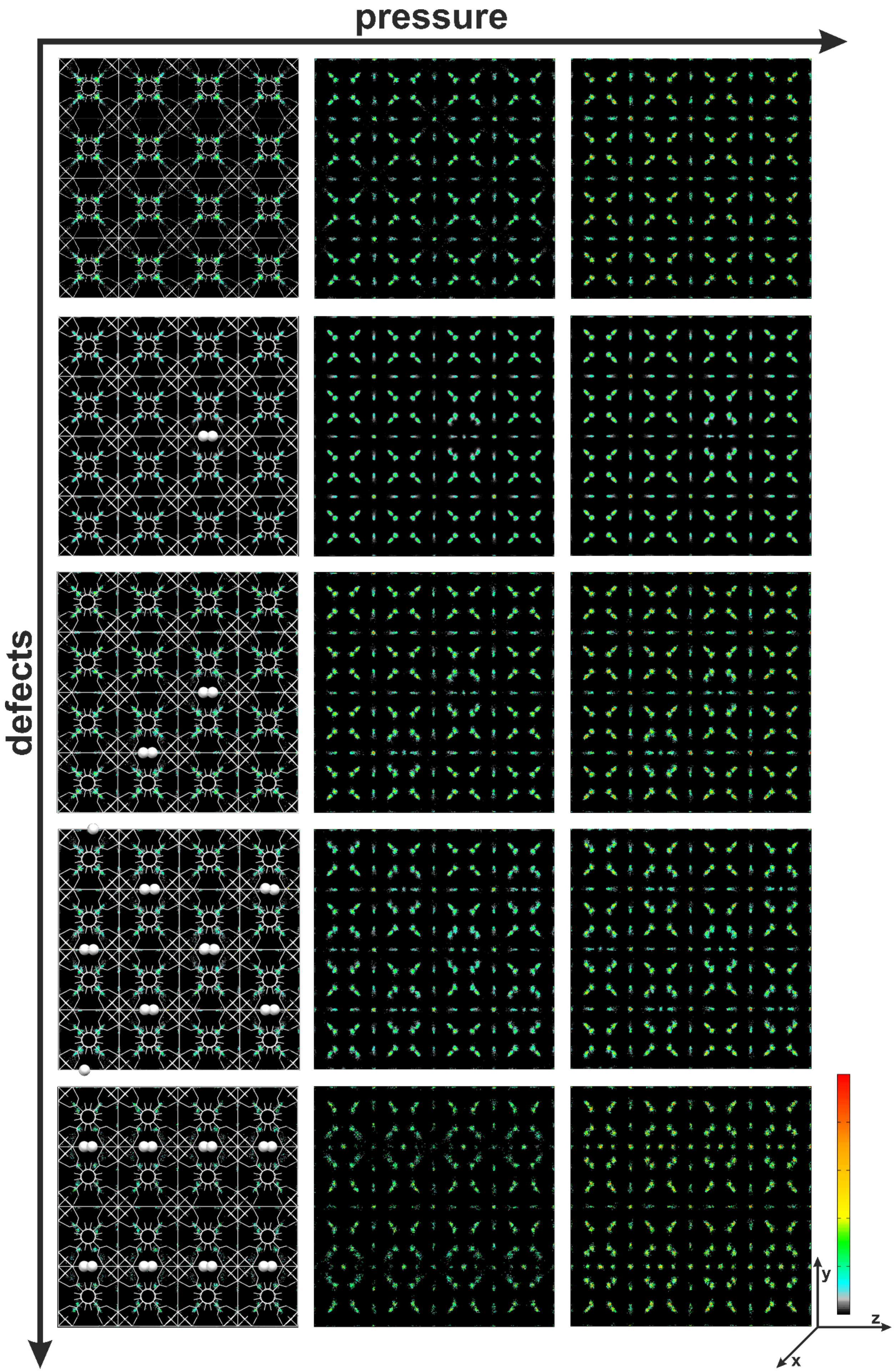

Figure S5. Average Occupation Profiles for adsorption of nitrogen in all UiO-66 structures with increasing number of defects at different values of pressure. The position of the defects are marked with white spheres.

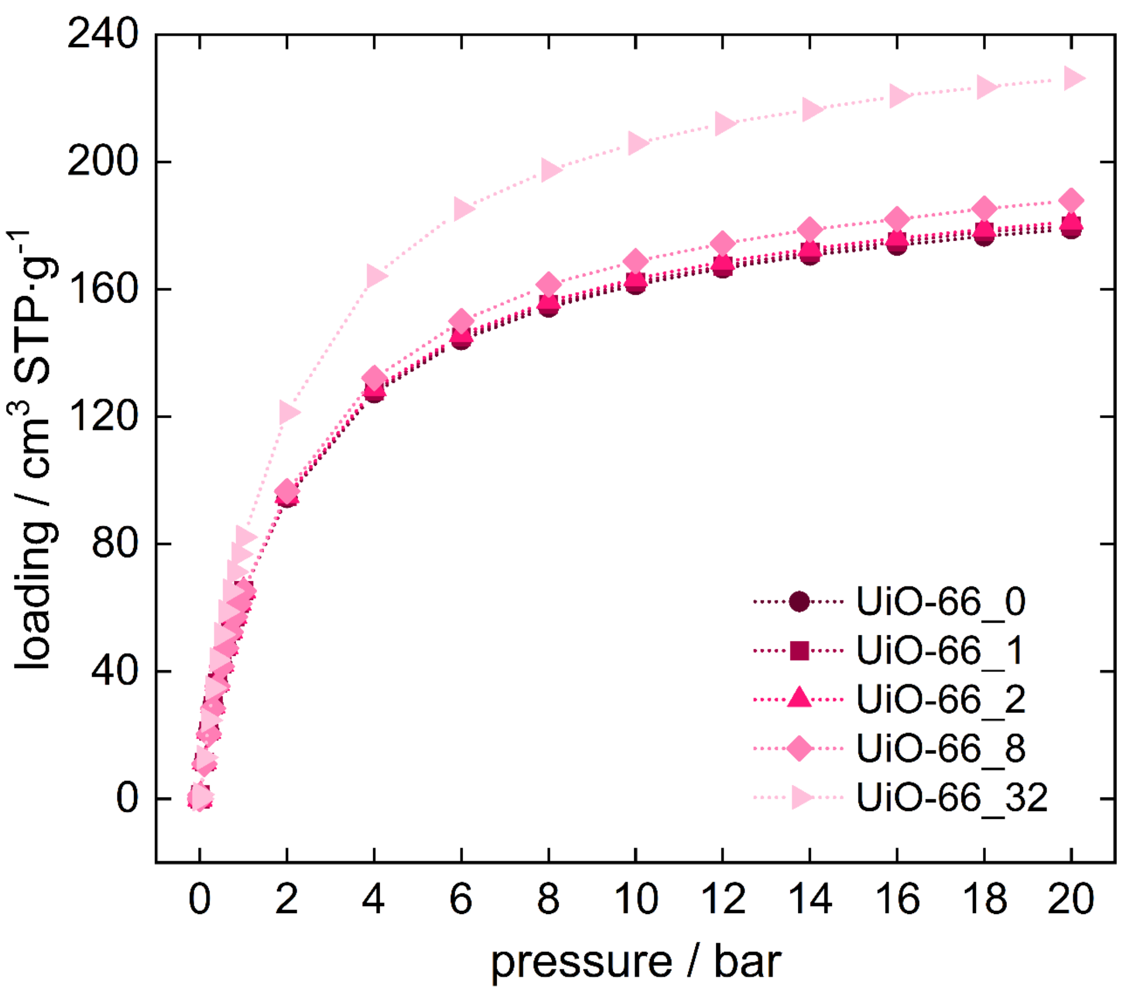

Figure S6. Calculated adsorption isotherms of carbon dioxide in UiO-66 structures at 27°C and up to 20 bar.

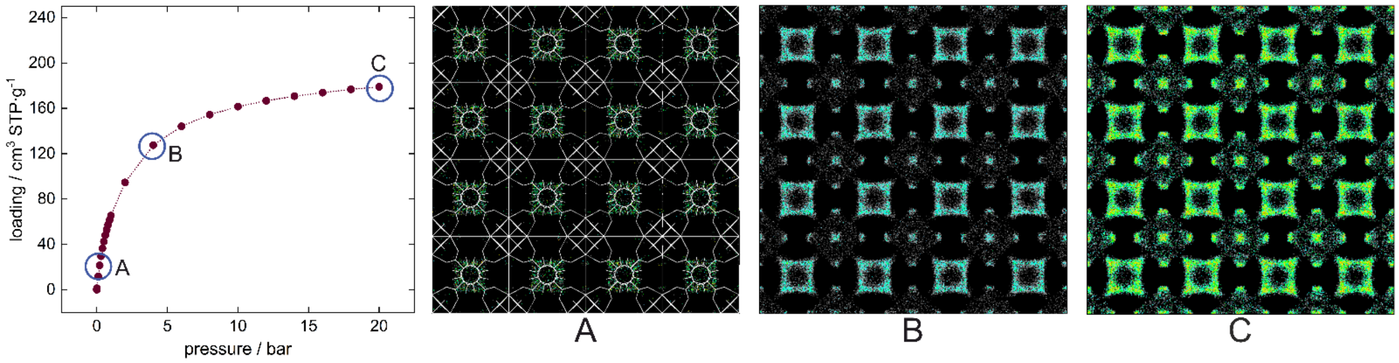

Figure S7. Calculated adsorption isotherm of carbon dioxide (left) in UiO-66_0 structure together with Average Occupation Profiles simulated at marked pressures.

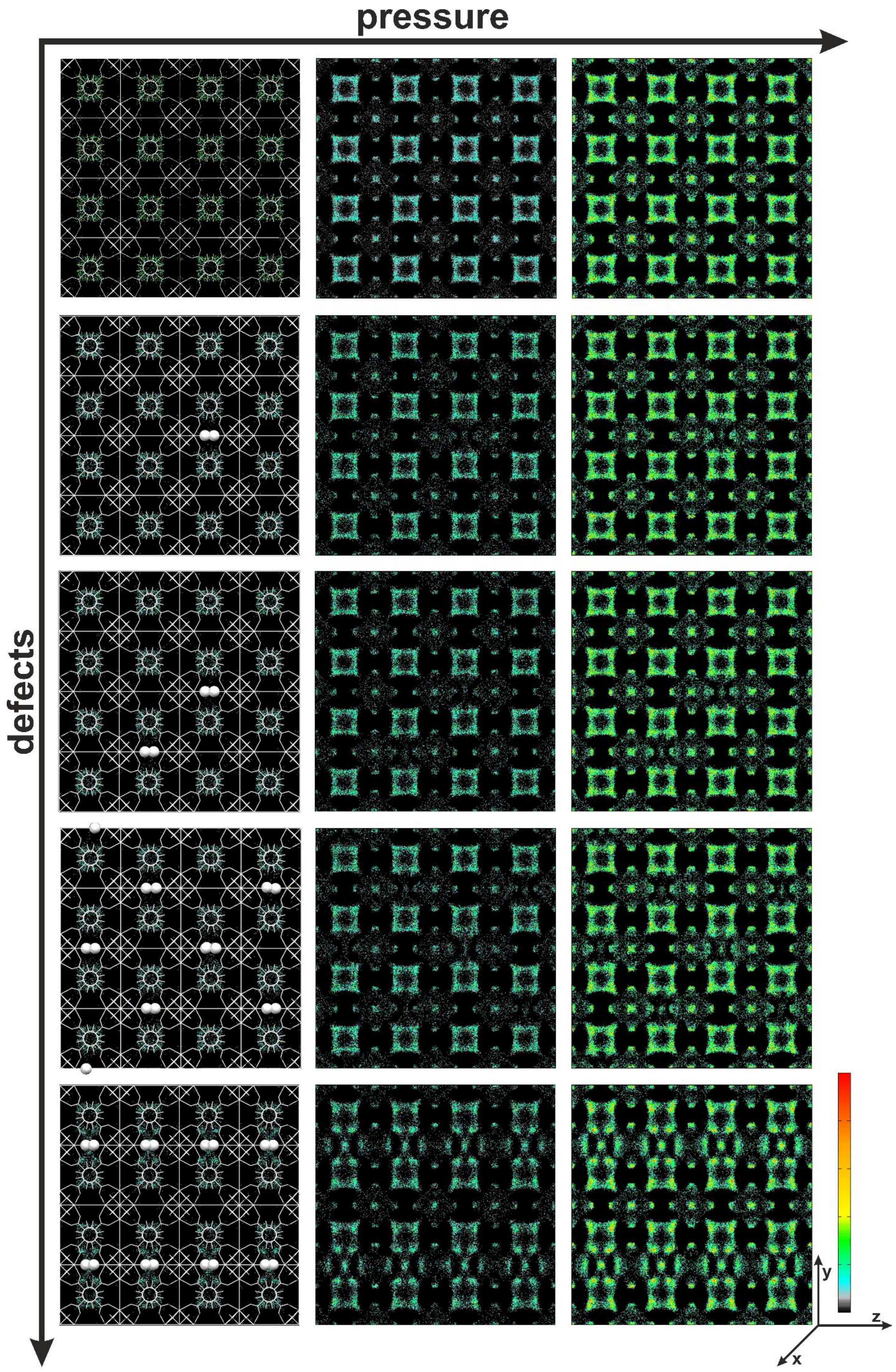

Figure S8. Average Occupation Profiles for adsorption of carbon dioxide in all UiO-66 structures with increasing number of defects at different values of pressure. The position of the defects are marked with white spheres.

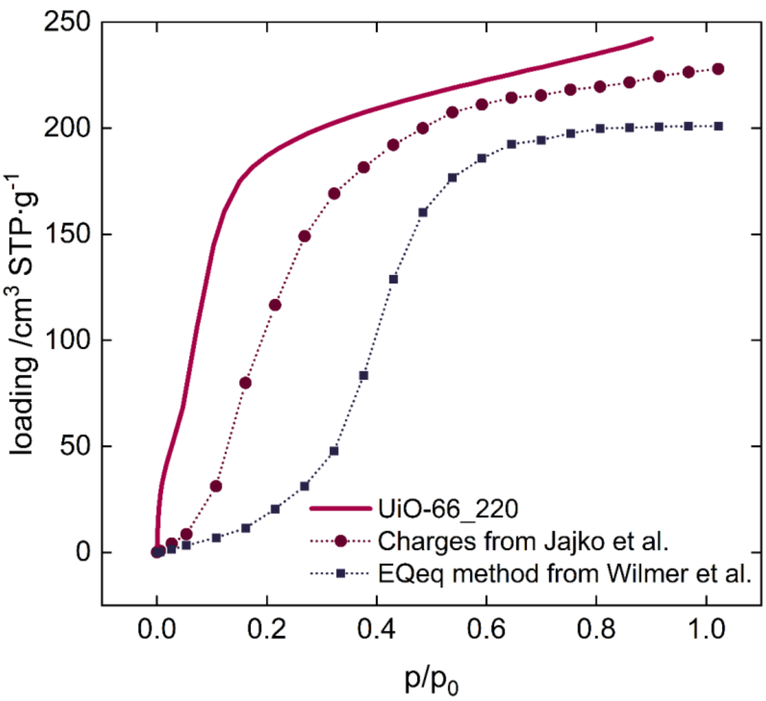

Figure S9. Validation of the set of charges of the materials used in the simulations [1] for methanol adsorption in the UiO-66_220 sample and the UiO-66_0 model compared to the set of charges obtained by the EQeq method [2] at 27°C.

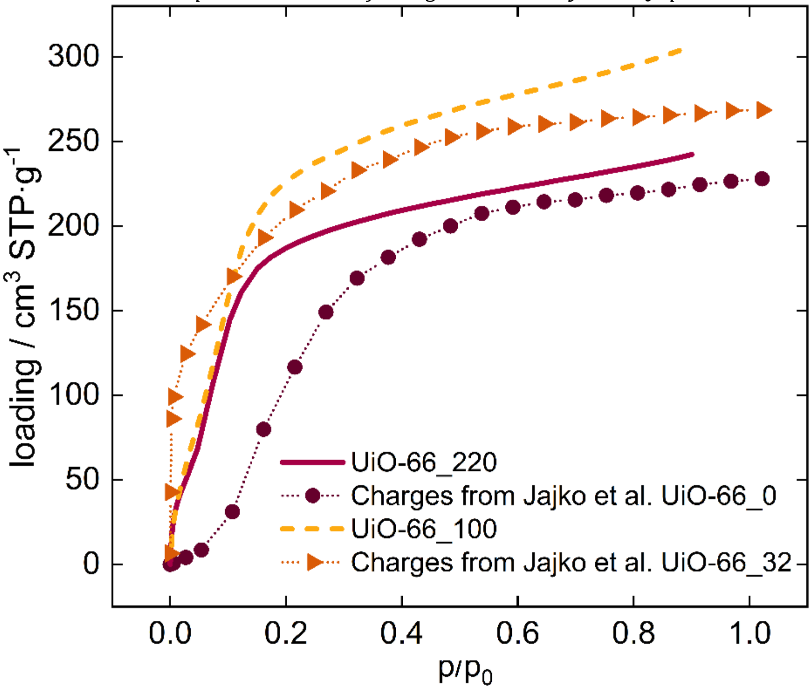

Figure S10. Validation of the set of charges for the materials used in the simulations [1] for methanol adsorption in the UiO-66_220 and UiO-66_100 samples and the UiO-66_0 and UiO-66_32 models at 27°C.

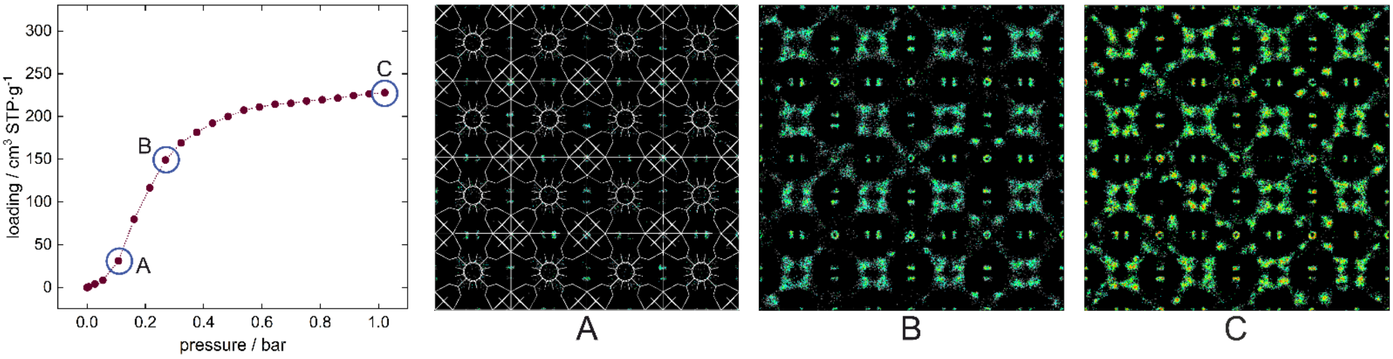

Figure S11. Calculated adsorption isotherm of methanol (left) in UiO-66_0 structure together with Average Occupation Profiles simulated at marked pressures.

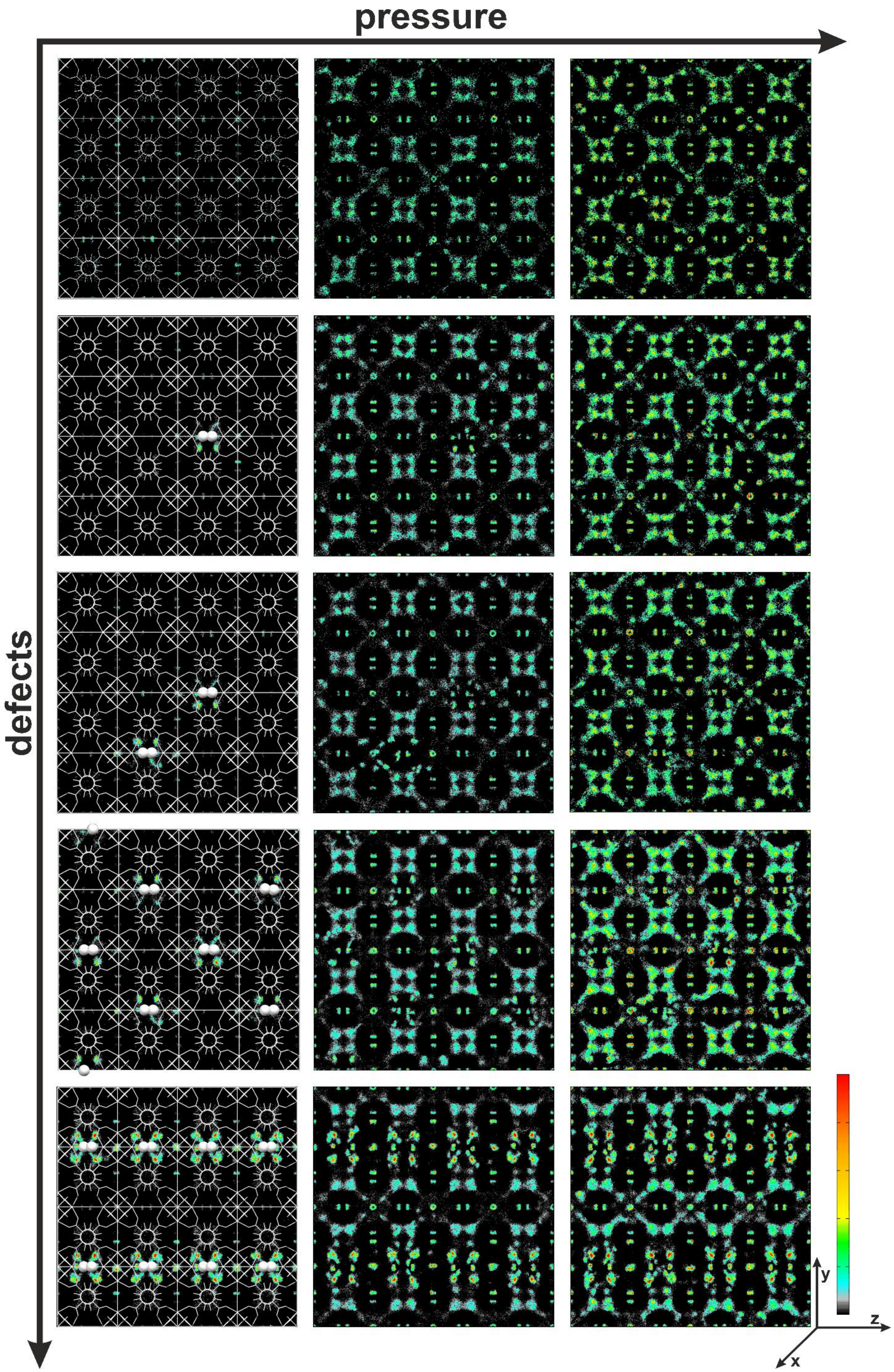

Figure S12. Average Occupation Profiles for adsorption of methanol in all UiO-66 structures with increasing number of defects at different values of pressure. The position of the defects are marked with white spheres.

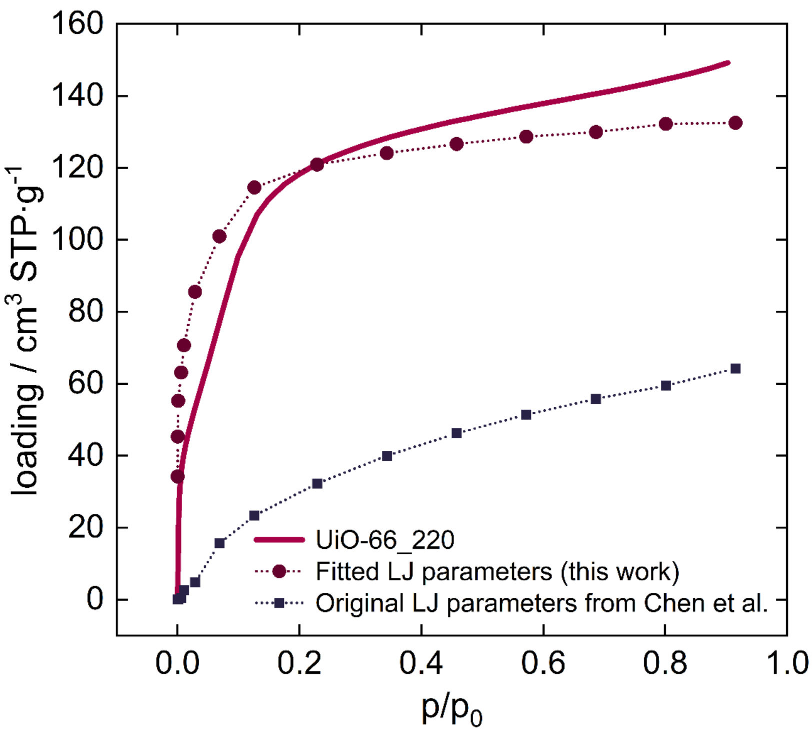

Figure S13. Comparison of the original (from Chen et al. [3]) and developed sets of LJ parameters for ethanol adsorption in the UiO-66_220 sample and the UiO-66_0 at 27°C.

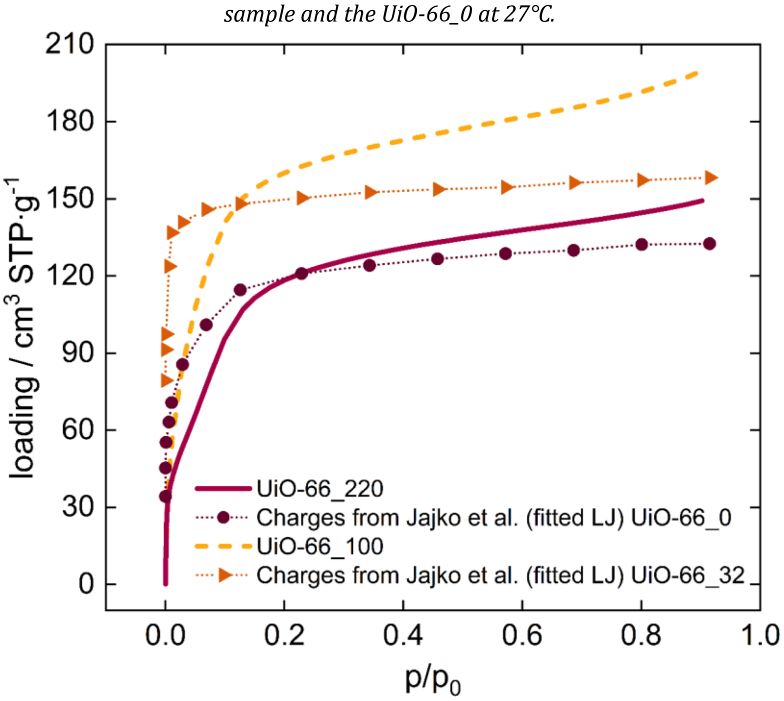

Figure S14. Validation of the set of charges of the materials used in the simulations [1] and the new LJ parameters for ethanol adsorption in the UiO-66_220 and UiO-66_100 samples and the UiO-66_0 and UiO-66_32 models at 27°C.

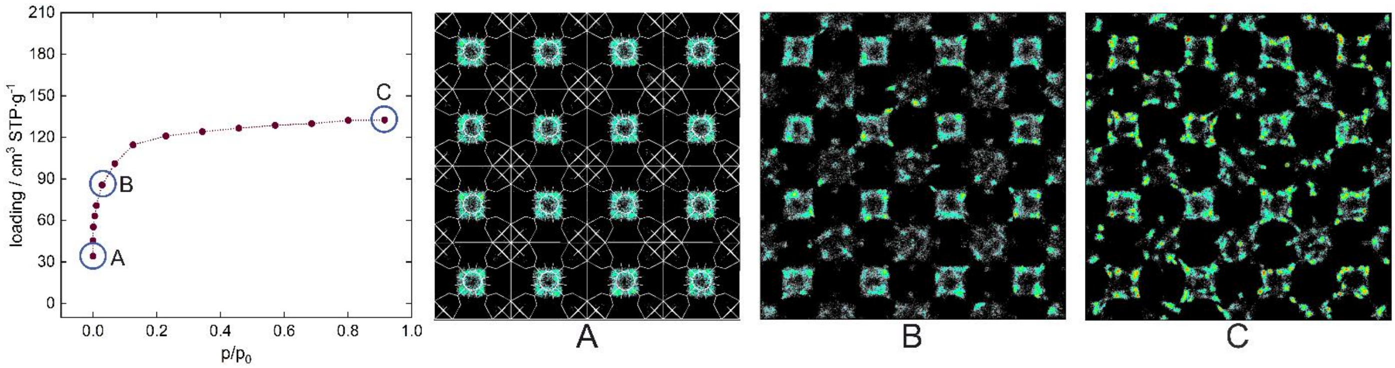

Figure S15. Calculated adsorption isotherm of ethanol (left) in UiO-66_0 structure together with Average Occupation Profiles simulated at marked pressures.

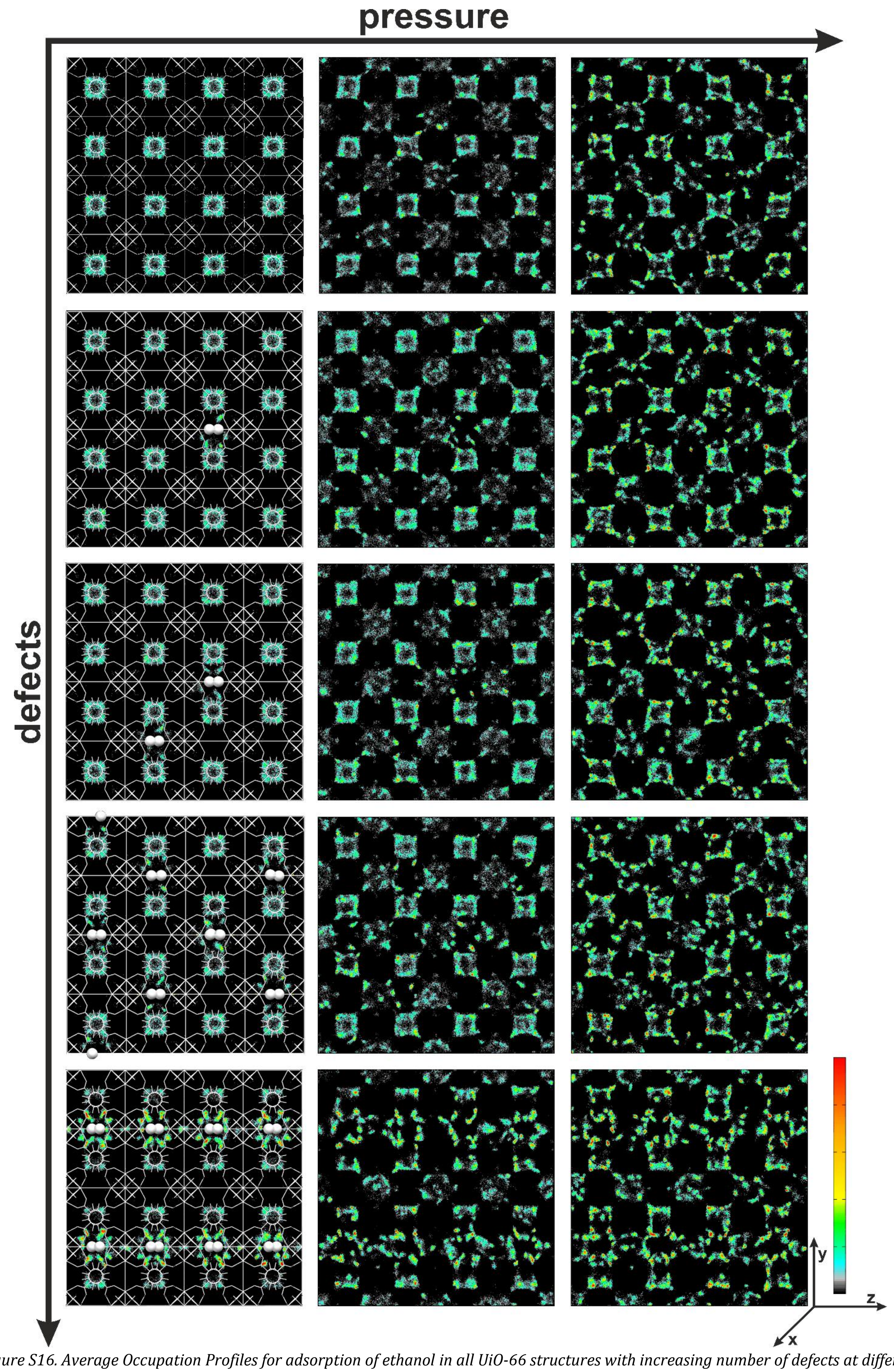

Figure S16. Average Occupation Profiles for adsorption of ethanol in all UiO-66 structures with increasing number of defects at different values of pressure. The position of the defects are marked with white spheres.

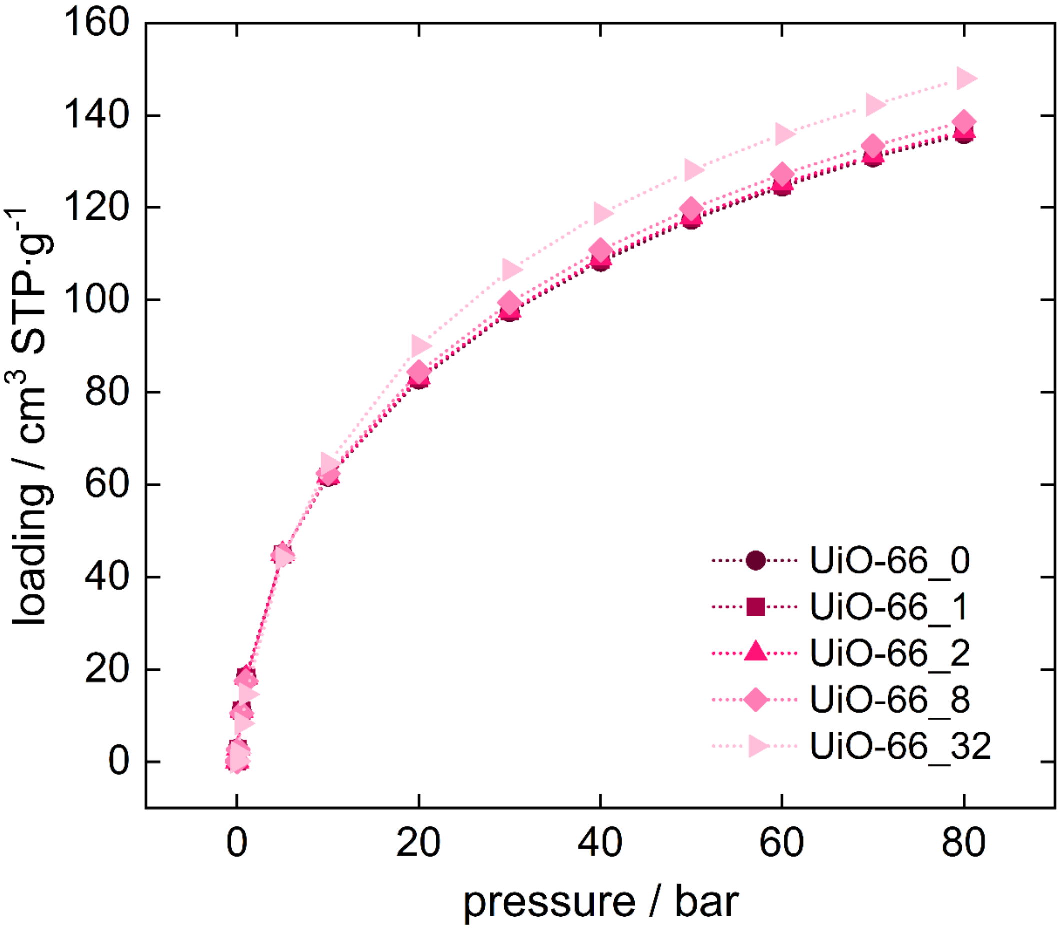

Figure S17. Calculated adsorption isotherms of methane in UiO-66 structures at 27°C.

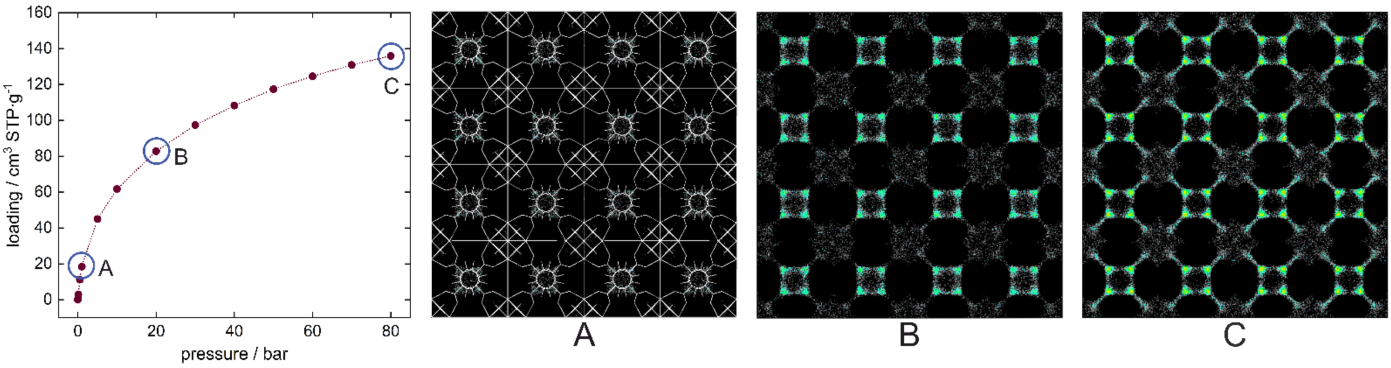

Figure S18. Calculated adsorption isotherm of methane (left) in UiO-66_0 structure together with Average Occupation Profiles simulated at marked pressures.

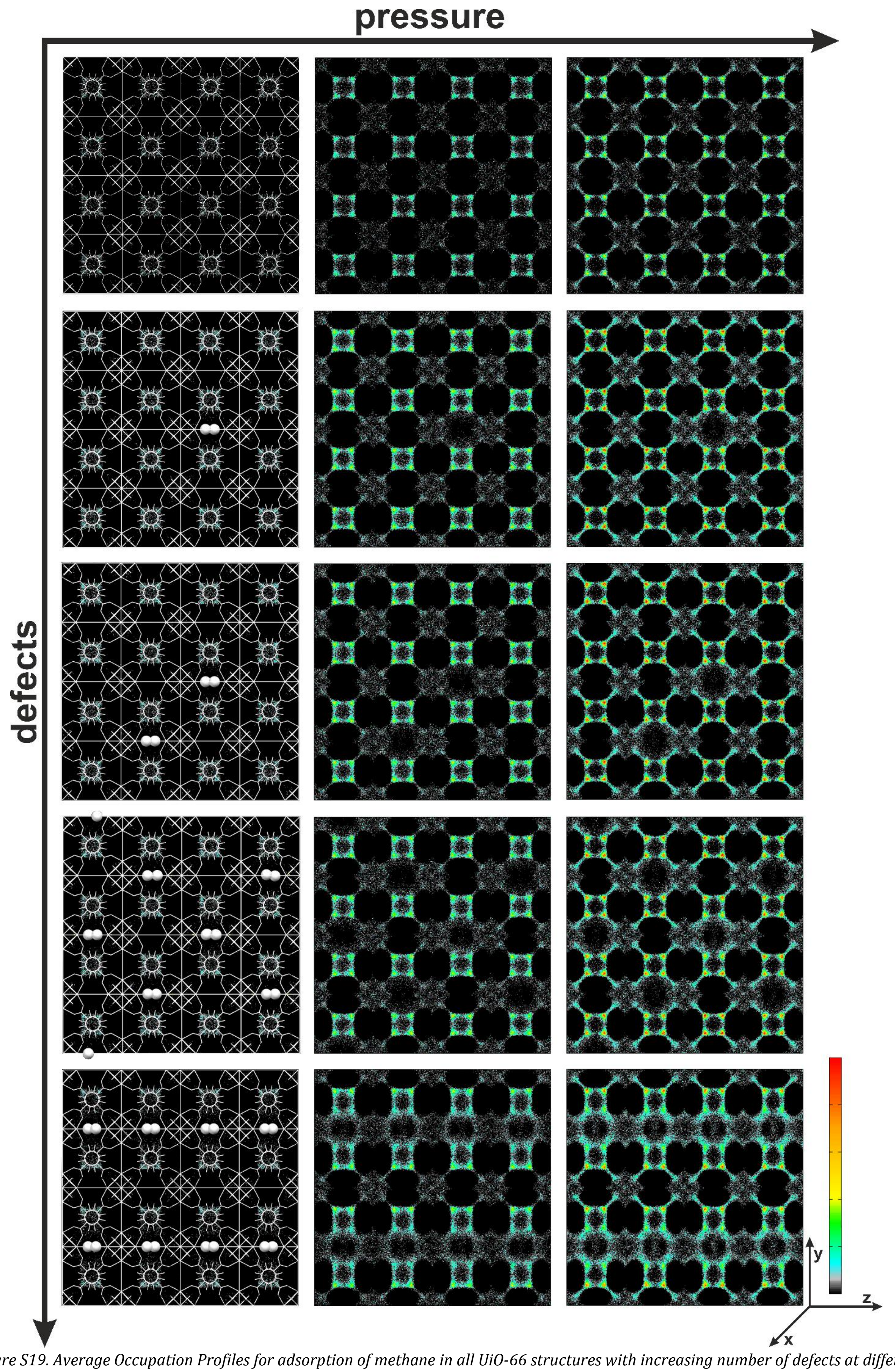

Figure S19. Average Occupation Profiles for adsorption of methane in all UiO-66 structures with increasing number of defects at different values of pressure. The position of the defects are marked with white spheres.

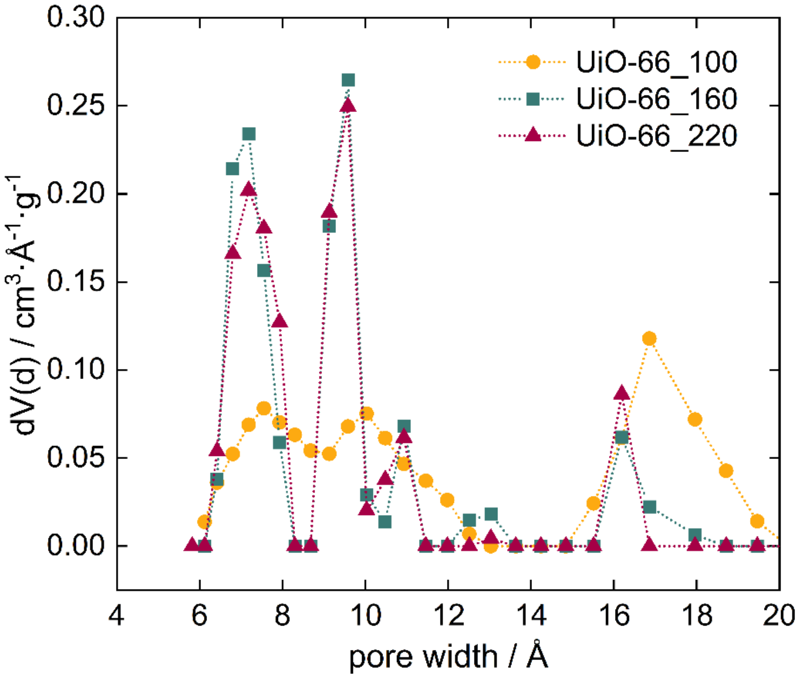

Figure S20. Pore size distribution for UiO-66 structures, obtained from argon adsorption isotherms. [1]

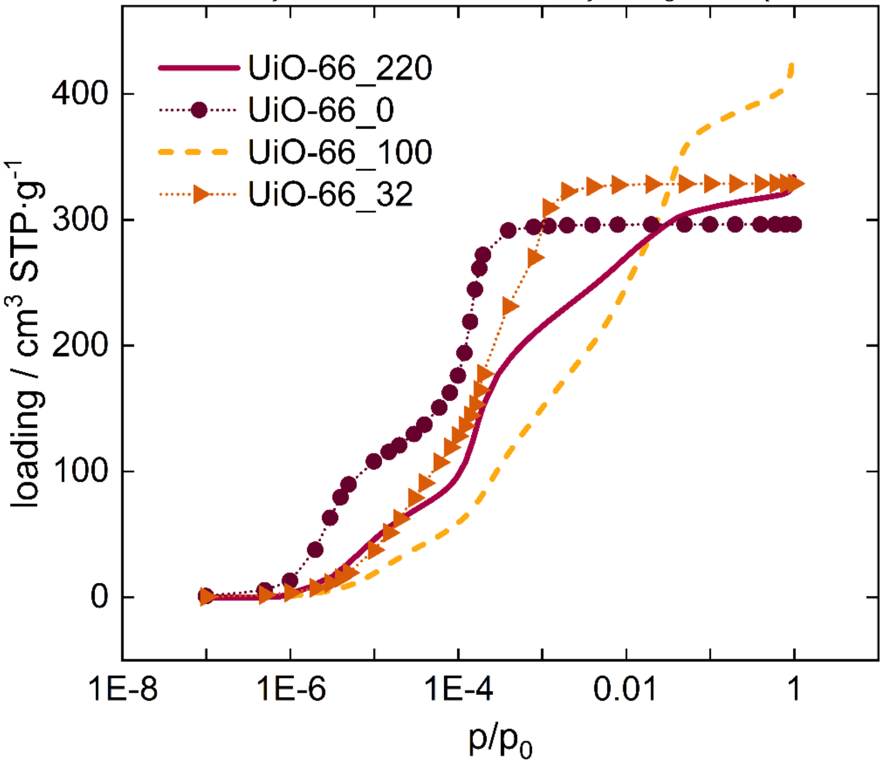

Figure S21. Argon adsorption in the UiO-66_220 and UiO-66_100 samples and the UiO-66_0 and UiO-66_32 models at -186°C.

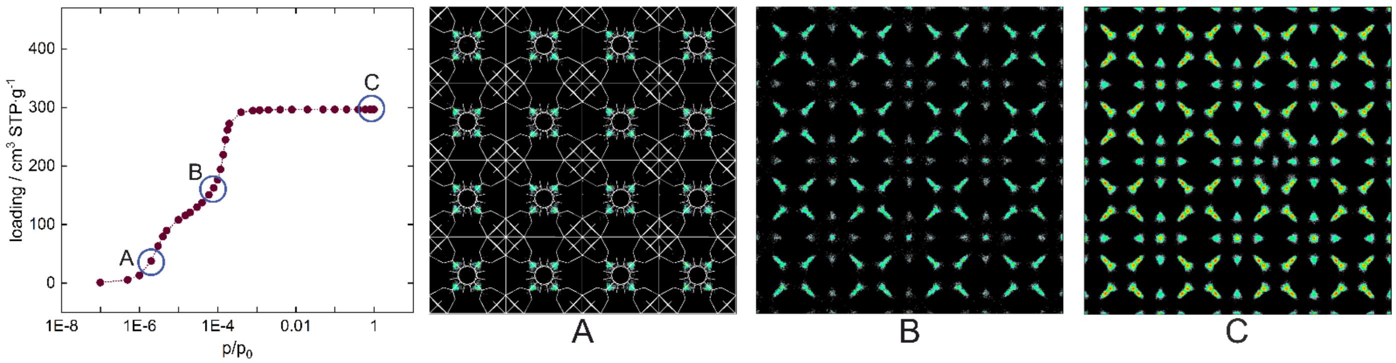

Figure S22. Calculated adsorption isotherm of argon (left) in UiO-66_0 structure together with Average Occupation Profiles simulated at marked pressures.

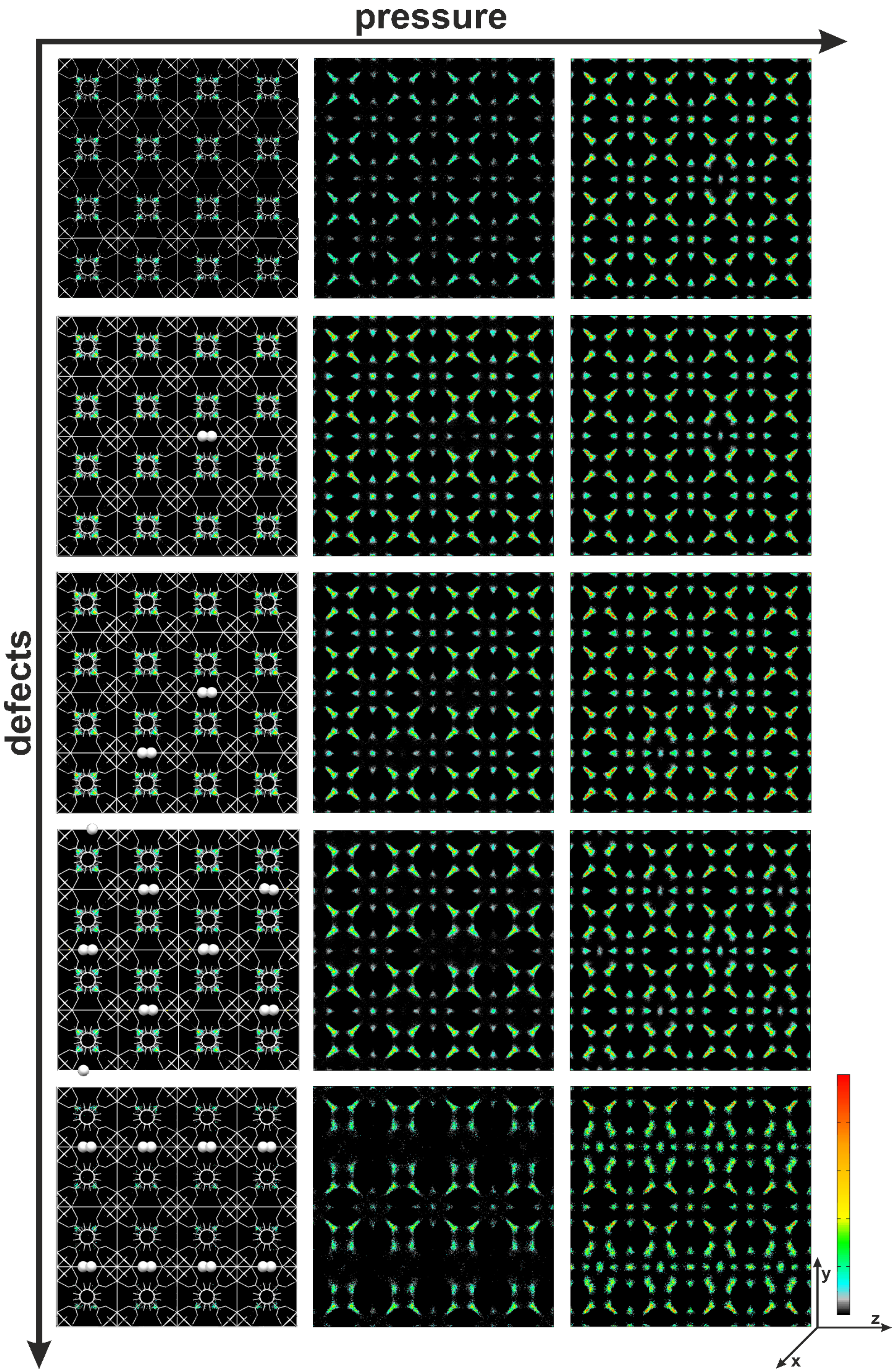

Figure S23. Average Occupation Profiles for adsorption of argon in all UiO-66 structures with increasing number of defects at different values of pressure. The position of the defects are marked with white spheres.

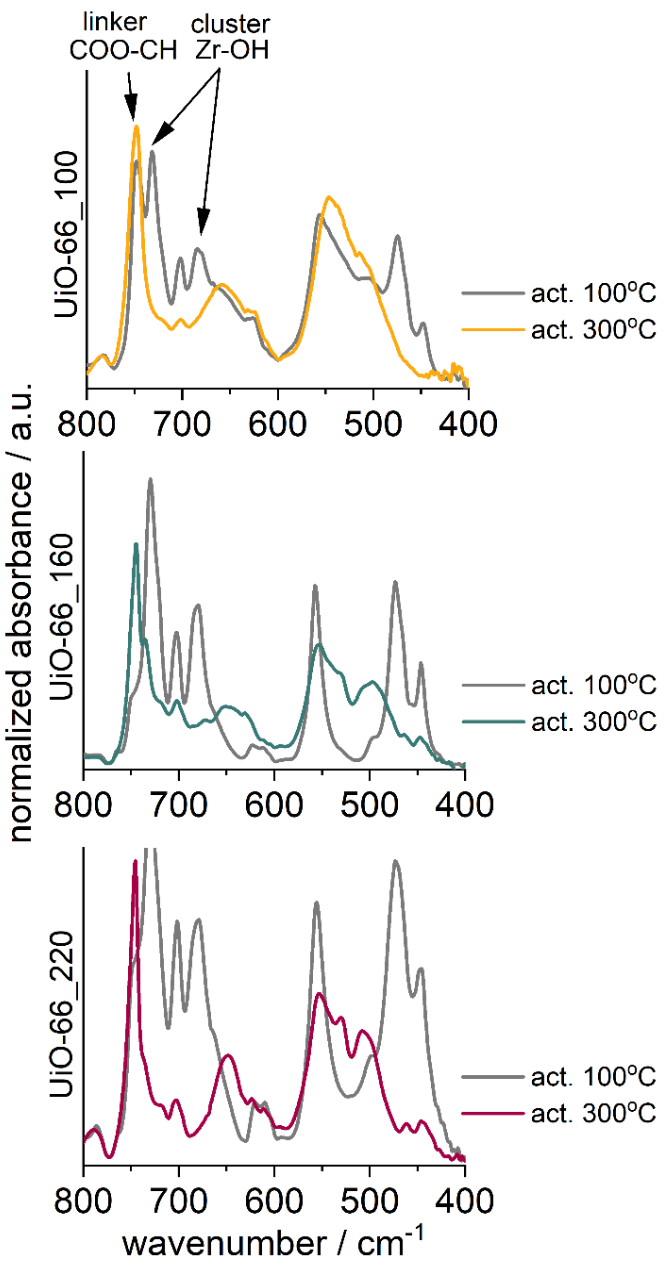

Figure S24. IR spectra of UiO-66-100, UiO-66-100 and UiO-66-100 in the range 400-900 cm⁻¹ after activation at 100°C and 300°C.

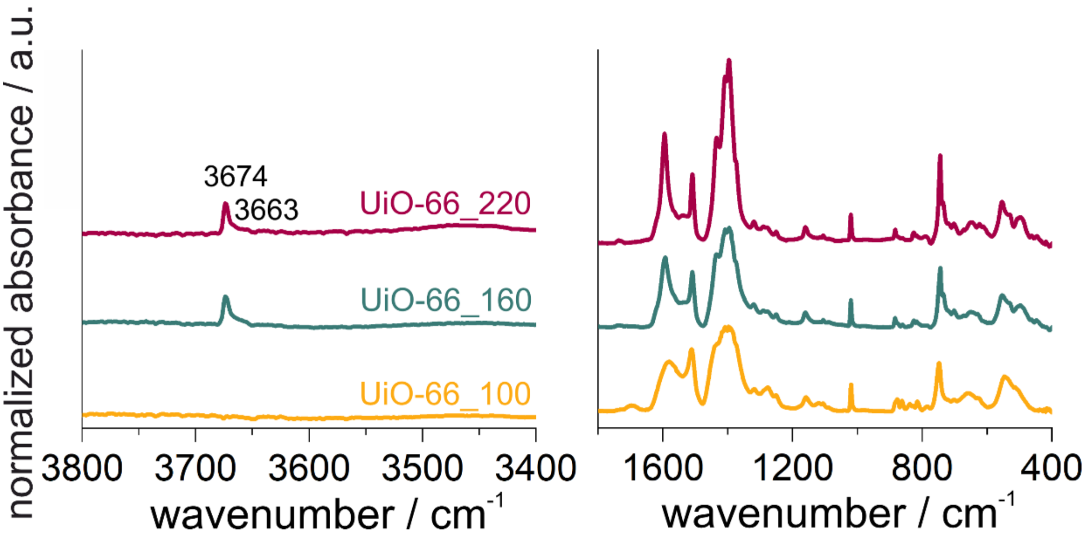

Figure S25. IR spectra in the range of OH groups and skeletal vibrations for UiO-66 materials activated at 300°C. Normalized to the same band height of 1020 cm^{-1}.

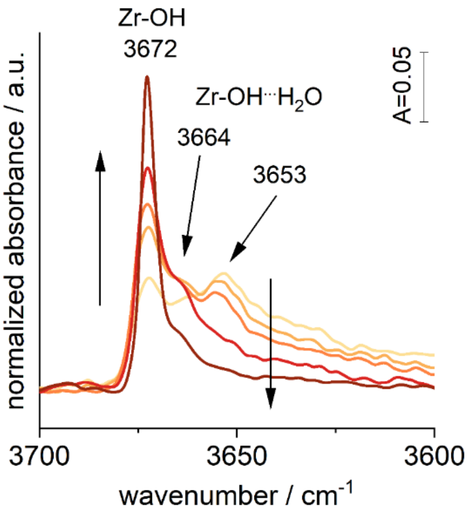

Figure S26. IR spectra in the region of OH stretching vibration or UiO-66_220 after evacuation at RT and 100°C (spectrum with highest intensity of 3672 cm^{-1}). Arrows mark direction of changes during dehydration: increase of the intensity of 3672 cm^{-1} band and decrease of the intensities of 3664 and 3653 cm^{-1} bands.

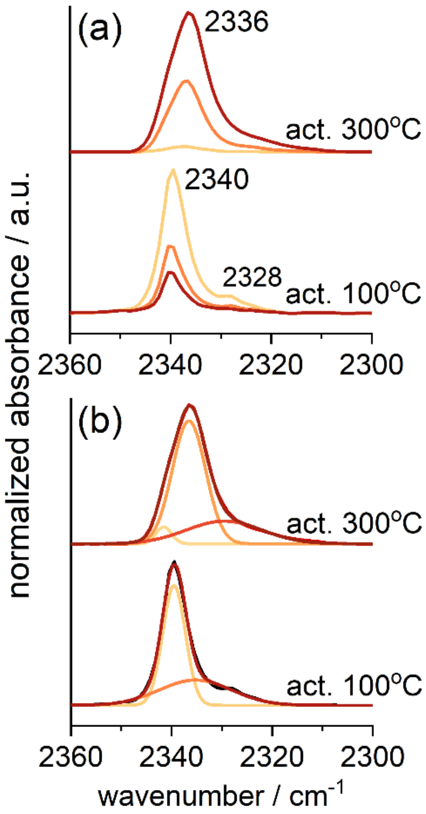

Figure S27. CO_2 adsorption in UiO-66_220 activated at 100 and 300°C (a) and band fitting for the representative spectra for both activation temperatures (b).

Supplementary References:

(1) Jajko, G.; Gutiérrez-Sevillano, J. J.; Sławek, A.; Szufla, M.; Kozyra, P.; Matoga, D.; Makowski, W.; S., C. Water Adsorption in Ideal and Defective UiO-66 Structures. *Microporous Mesoporous Mater.* **2021**, *330*, 111555. https://doi.org/10.1016/j.micromeso.2021.111555.

(2) Wilmer, C. E.; Kim, K. C.; Snurr, R. Q. An Extended Charge Equilibration Method. *J. Phys. Chem. Lett.* **2012**, *3* (17), 2506–2511. https://doi.org/10.1021/jz3008485.

(3) Chen, B.; Potoff, J. J.; Siepmann, J. I. Monte Carlo Calculations for Alcohols and Their Mixtures with Alkanes. Transferable Potentials for Phase Equilibria. 5. United-Atom Description of Primary, Secondary, and Tertiary Alcohols. *J. Phys. Chem. B* **2002**, *105*, 3093–3104. https://doi.org/10.1021/jp003882x.
