# Supplementary material for: Defect-induced tuning of polarity-dependent adsorption in hydrophobic–hydrophilic UiO-66
Source: Commun Chem. 2022 Oct 7;5:120. doi: 10.1038/s42004-022-00742-z (PMC9814431; doi:10.1038/s42004-022-00742-z)
Supplement: Supplementary file 3 — Description of Additional Supplementary Files [file 42004_2022_742_MOESM3_ESM.docx]

Description of Additional Supplementary Files

**File name:** Supplementary Data 1

**Description:** Cif file for the UiO-66 structure without defects.

**File name:** Supplementary Data 2

**Description:** Cif file for the UiO-66 structure with 1 defect per unit cell

**File name:** Supplementary Data 3

**Description:** Cif file for the UiO-66 structure with 2 defect per unit cell

**File name:** Supplementary Data 4

**Description:** Cif file for the UiO-66 structure with 8 defect per unit cell

**File name:** Supplementary Data 5

**Description:** Cif file for the UiO-66 structure with 32 defect per unit cell
