# Supplementary material for: Defect-induced tuning of polarity-dependent adsorption in hydrophobic–hydrophilic UiO-66
Source: Commun Chem. 2022 Oct 7;5:120. doi: 10.1038/s42004-022-00742-z (PMC9814431; doi:10.1038/s42004-022-00742-z)
Supplement: Supplementary file 4 — Supplementary Data 1 [file 42004_2022_742_MOESM4_ESM.pdf]

```
data_UiO-66

_cell_length_a    20.70040
_cell_length_b    20.70040
_cell_length_c    20.70040
_cell_angle_alpha 90
_cell_angle_beta  90
_cell_angle_gamma 90


_symmetry_equiv_pos_as_xyz 'x,y,z'

loop_
_atom_site_label
_atom_site_type_symbol
_atom_site_fract_x
_atom_site_fract_y
_atom_site_fract_z
C2    C      0.267500144925     0.000000000000     0.184399818361
C2    C      0.267500144925     0.500000000000     0.684399818361
C2    C      0.767500144925     0.000000000000     0.684399818361
C2    C      0.767500144925     0.500000000000     0.184399818361
C2    C      0.732499855075     0.000000000000     0.184399818361
C2    C      0.732499855075     0.500000000000     0.684399818361
C2    C      0.232499855075     0.000000000000     0.684399818361
C2    C      0.232499855075     0.500000000000     0.184399818361
C2    C      0.732499855075     0.000000000000     0.815600181639
C2    C      0.732499855075     0.500000000000     0.315600181639
C2    C      0.232499855075     0.000000000000     0.315600181639
C2    C      0.232499855075     0.500000000000     0.815600181639
C2    C      0.267500144925     0.000000000000     0.815600181639
C2    C      0.267500144925     0.500000000000     0.315600181639
C2    C      0.767500144925     0.000000000000     0.315600181639
C2    C      0.767500144925     0.500000000000     0.815600181639
C2    C      0.184399818361     0.267500144925     0.000000000000
C2    C      0.184399818361     0.767500144925     0.500000000000
C2    C      0.684399818361     0.267500144925     0.500000000000
C2    C      0.684399818361     0.767500144925     0.000000000000
C2    C      0.184399818361     0.732499855075     0.000000000000
C2    C      0.184399818361     0.232499855075     0.500000000000
C2    C      0.684399818361     0.732499855075     0.500000000000
C2    C      0.684399818361     0.232499855075     0.000000000000
C2    C      0.815600181639     0.732499855075     0.000000000000
C2    C      0.815600181639     0.232499855075     0.500000000000
C2    C      0.315600181639     0.732499855075     0.500000000000
C2    C      0.315600181639     0.232499855075     0.000000000000
C2    C      0.815600181639     0.267500144925     0.000000000000
C2    C      0.815600181639     0.767500144925     0.500000000000
C2    C      0.315600181639     0.267500144925     0.500000000000
C2    C      0.315600181639     0.767500144925     0.000000000000
C2    C      0.000000000000     0.184399818361     0.267500144925
C2    C      0.000000000000     0.684399818361     0.767500144925
C2    C      0.500000000000     0.184399818361     0.767500144925
C2    C      0.500000000000     0.684399818361     0.267500144925
```

55	C2	C	0.000000000000	0.184399818361	0.732499855075
56	C2	C	0.000000000000	0.684399818361	0.232499855075
57	C2	C	0.500000000000	0.184399818361	0.232499855075
58	C2	C	0.500000000000	0.684399818361	0.732499855075
59	C2	C	0.000000000000	0.815600181639	0.732499855075
60	C2	C	0.000000000000	0.315600181639	0.232499855075
61	C2	C	0.500000000000	0.815600181639	0.232499855075
62	C2	C	0.500000000000	0.315600181639	0.732499855075
63	C2	C	0.000000000000	0.815600181639	0.267500144925
64	C2	C	0.000000000000	0.315600181639	0.767500144925
65	C2	C	0.500000000000	0.815600181639	0.767500144925
66	C2	C	0.500000000000	0.315600181639	0.267500144925
67	C2	C	0.000000000000	0.267500144925	0.815600181639
68	C2	C	0.000000000000	0.767500144925	0.315600181639
69	C2	C	0.500000000000	0.267500144925	0.315600181639
70	C2	C	0.500000000000	0.767500144925	0.815600181639
71	C2	C	0.000000000000	0.732499855075	0.815600181639
72	C2	C	0.000000000000	0.232499855075	0.315600181639
73	C2	C	0.500000000000	0.732499855075	0.315600181639
74	C2	C	0.500000000000	0.232499855075	0.815600181639
75	C2	C	0.000000000000	0.732499855075	0.184399818361
76	C2	C	0.000000000000	0.232499855075	0.684399818361
77	C2	C	0.500000000000	0.732499855075	0.684399818361
78	C2	C	0.500000000000	0.232499855075	0.184399818361
79	C2	C	0.000000000000	0.267500144925	0.184399818361
80	C2	C	0.000000000000	0.767500144925	0.684399818361
81	C2	C	0.500000000000	0.267500144925	0.684399818361
82	C2	C	0.500000000000	0.767500144925	0.184399818361
83	C2	C	0.267500144925	0.184399818361	0.000000000000
84	C2	C	0.267500144925	0.684399818361	0.500000000000
85	C2	C	0.767500144925	0.184399818361	0.500000000000
86	C2	C	0.767500144925	0.684399818361	0.000000000000
87	C2	C	0.732499855075	0.184399818361	0.000000000000
88	C2	C	0.732499855075	0.684399818361	0.500000000000
89	C2	C	0.232499855075	0.184399818361	0.500000000000
90	C2	C	0.232499855075	0.684399818361	0.000000000000
91	C2	C	0.732499855075	0.815600181639	0.000000000000
92	C2	C	0.732499855075	0.315600181639	0.500000000000
93	C2	C	0.232499855075	0.815600181639	0.500000000000
94	C2	C	0.232499855075	0.315600181639	0.000000000000
95	C2	C	0.267500144925	0.815600181639	0.000000000000
96	C2	C	0.267500144925	0.315600181639	0.500000000000
97	C2	C	0.767500144925	0.815600181639	0.500000000000
98	C2	C	0.767500144925	0.315600181639	0.000000000000
99	C2	C	0.184399818361	0.000000000000	0.732499855075
100	C2	C	0.184399818361	0.500000000000	0.232499855075
101	C2	C	0.684399818361	0.000000000000	0.232499855075
102	C2	C	0.684399818361	0.500000000000	0.732499855075
103	C2	C	0.184399818361	0.000000000000	0.267500144925
104	C2	C	0.184399818361	0.500000000000	0.767500144925
105	C2	C	0.684399818361	0.000000000000	0.767500144925
106	C2	C	0.684399818361	0.500000000000	0.267500144925
107	C2	C	0.815600181639	0.000000000000	0.267500144925
108	C2	C	0.815600181639	0.500000000000	0.767500144925

109	C2	C	0.315600181639	0.000000000000	0.767500144925
110	C2	C	0.315600181639	0.500000000000	0.267500144925
111	C2	C	0.815600181639	0.000000000000	0.732499855075
112	C2	C	0.815600181639	0.500000000000	0.232499855075
113	C2	C	0.315600181639	0.000000000000	0.232499855075
114	C2	C	0.315600181639	0.500000000000	0.732499855075
115	C1	C	0.153599930436	0.000000000000	0.846400069564
116	C1	C	0.153599930436	0.500000000000	0.346400069564
117	C1	C	0.653599930436	0.000000000000	0.346400069564
118	C1	C	0.653599930436	0.500000000000	0.846400069564
119	C1	C	0.846400069564	0.000000000000	0.846400069564
120	C1	C	0.846400069564	0.500000000000	0.346400069564
121	C1	C	0.346400069564	0.000000000000	0.346400069564
122	C1	C	0.346400069564	0.500000000000	0.846400069564
123	C1	C	0.846400069564	0.000000000000	0.153599930436
124	C1	C	0.846400069564	0.500000000000	0.653599930436
125	C1	C	0.346400069564	0.000000000000	0.653599930436
126	C1	C	0.346400069564	0.500000000000	0.153599930436
127	C1	C	0.153599930436	0.000000000000	0.153599930436
128	C1	C	0.153599930436	0.500000000000	0.653599930436
129	C1	C	0.653599930436	0.000000000000	0.653599930436
130	C1	C	0.653599930436	0.500000000000	0.153599930436
131	C1	C	0.846400069564	0.153599930436	0.000000000000
132	C1	C	0.846400069564	0.653599930436	0.500000000000
133	C1	C	0.346400069564	0.153599930436	0.500000000000
134	C1	C	0.346400069564	0.653599930436	0.000000000000
135	C1	C	0.846400069564	0.846400069564	0.000000000000
136	C1	C	0.846400069564	0.346400069564	0.500000000000
137	C1	C	0.346400069564	0.846400069564	0.500000000000
138	C1	C	0.346400069564	0.346400069564	0.000000000000
139	C1	C	0.153599930436	0.846400069564	0.000000000000
140	C1	C	0.153599930436	0.346400069564	0.500000000000
141	C1	C	0.653599930436	0.846400069564	0.500000000000
142	C1	C	0.653599930436	0.346400069564	0.000000000000
143	C1	C	0.153599930436	0.153599930436	0.000000000000
144	C1	C	0.153599930436	0.653599930436	0.500000000000
145	C1	C	0.653599930436	0.153599930436	0.500000000000
146	C1	C	0.653599930436	0.653599930436	0.000000000000
147	C1	C	0.000000000000	0.846400069564	0.153599930436
148	C1	C	0.000000000000	0.346400069564	0.653599930436
149	C1	C	0.500000000000	0.846400069564	0.653599930436
150	C1	C	0.500000000000	0.346400069564	0.153599930436
151	C1	C	0.000000000000	0.846400069564	0.846400069564
152	C1	C	0.000000000000	0.346400069564	0.346400069564
153	C1	C	0.500000000000	0.846400069564	0.346400069564
154	C1	C	0.500000000000	0.346400069564	0.846400069564
155	C1	C	0.000000000000	0.153599930436	0.846400069564
156	C1	C	0.000000000000	0.653599930436	0.346400069564
157	C1	C	0.500000000000	0.153599930436	0.346400069564
158	C1	C	0.500000000000	0.653599930436	0.846400069564
159	C1	C	0.000000000000	0.153599930436	0.153599930436
160	C1	C	0.000000000000	0.653599930436	0.653599930436
161	C1	C	0.500000000000	0.153599930436	0.653599930436
162	C1	C	0.500000000000	0.653599930436	0.153599930436

163	C3	C	0.204999903384	0.000000000000	0.204999903384
164	C3	C	0.204999903384	0.500000000000	0.704999903384
165	C3	C	0.704999903384	0.000000000000	0.704999903384
166	C3	C	0.704999903384	0.500000000000	0.204999903384
167	C3	C	0.795000096616	0.000000000000	0.204999903384
168	C3	C	0.795000096616	0.500000000000	0.704999903384
169	C3	C	0.295000096616	0.000000000000	0.704999903384
170	C3	C	0.295000096616	0.500000000000	0.204999903384
171	C3	C	0.795000096616	0.000000000000	0.795000096616
172	C3	C	0.795000096616	0.500000000000	0.295000096616
173	C3	C	0.295000096616	0.000000000000	0.295000096616
174	C3	C	0.295000096616	0.500000000000	0.795000096616
175	C3	C	0.204999903384	0.000000000000	0.795000096616
176	C3	C	0.204999903384	0.500000000000	0.295000096616
177	C3	C	0.704999903384	0.000000000000	0.295000096616
178	C3	C	0.704999903384	0.500000000000	0.795000096616
179	C3	C	0.204999903384	0.204999903384	0.000000000000
180	C3	C	0.204999903384	0.704999903384	0.500000000000
181	C3	C	0.704999903384	0.204999903384	0.500000000000
182	C3	C	0.704999903384	0.704999903384	0.000000000000
183	C3	C	0.204999903384	0.795000096616	0.000000000000
184	C3	C	0.204999903384	0.295000096616	0.500000000000
185	C3	C	0.704999903384	0.795000096616	0.500000000000
186	C3	C	0.704999903384	0.295000096616	0.000000000000
187	C3	C	0.795000096616	0.795000096616	0.000000000000
188	C3	C	0.795000096616	0.295000096616	0.500000000000
189	C3	C	0.295000096616	0.795000096616	0.500000000000
190	C3	C	0.295000096616	0.295000096616	0.000000000000
191	C3	C	0.795000096616	0.204999903384	0.000000000000
192	C3	C	0.795000096616	0.704999903384	0.500000000000
193	C3	C	0.295000096616	0.204999903384	0.500000000000
194	C3	C	0.295000096616	0.704999903384	0.000000000000
195	C3	C	0.000000000000	0.204999903384	0.204999903384
196	C3	C	0.000000000000	0.704999903384	0.704999903384
197	C3	C	0.500000000000	0.204999903384	0.704999903384
198	C3	C	0.500000000000	0.704999903384	0.204999903384
199	C3	C	0.000000000000	0.204999903384	0.795000096616
200	C3	C	0.000000000000	0.704999903384	0.295000096616
201	C3	C	0.500000000000	0.204999903384	0.295000096616
202	C3	C	0.500000000000	0.704999903384	0.795000096616
203	C3	C	0.000000000000	0.795000096616	0.795000096616
204	C3	C	0.000000000000	0.295000096616	0.295000096616
205	C3	C	0.500000000000	0.795000096616	0.295000096616
206	C3	C	0.500000000000	0.295000096616	0.795000096616
207	C3	C	0.000000000000	0.795000096616	0.204999903384
208	C3	C	0.000000000000	0.295000096616	0.704999903384
209	C3	C	0.500000000000	0.795000096616	0.704999903384
210	C3	C	0.500000000000	0.295000096616	0.204999903384
211	H1	H	0.278520221831	0.999932851539	0.133090181832
212	H1	H	0.278520221831	0.499931885374	0.633090181832
213	H1	H	0.778518772584	0.999930919209	0.633090181832
214	H1	H	0.778523120326	0.499939131611	0.133090181832
215	H1	H	0.721477845839	0.000063766884	0.133090181832
216	H1	H	0.721481227416	0.500070046956	0.633090181832

217	H1	H	0.221478812004	0.000066182296	0.633090181832
218	H1	H	0.221481227416	0.500070046956	0.133090181832
219	H1	H	0.721478812004	0.999928020715	0.866909818168
220	H1	H	0.721478812004	0.499933817704	0.366909818168
221	H1	H	0.221479778169	0.999930919209	0.366909818168
222	H1	H	0.221482193581	0.499937199281	0.866910784333
223	H1	H	0.278520221831	0.000067148461	0.866909818168
224	H1	H	0.278517806419	0.500065216131	0.366910784333
225	H1	H	0.778522154161	0.000067148461	0.366909818168
226	H1	H	0.778517806419	0.500072945450	0.866910784333
227	H1	H	0.133090181832	0.278521187996	0.999928020715
228	H1	H	0.133090181832	0.778521187996	0.499939131611
229	H1	H	0.633090181832	0.278520221831	0.499929953044
230	H1	H	0.633089215667	0.778513941760	0.999940097776
231	H1	H	0.133090181832	0.721477845839	0.000062800719
232	H1	H	0.133090181832	0.221479778169	0.500070046956
233	H1	H	0.633090181832	0.721477845839	0.500065216131
234	H1	H	0.633090181832	0.221479778169	0.000074877780
235	H1	H	0.866909818168	0.721479778169	0.999925122220
236	H1	H	0.866909818168	0.221481227416	0.499939131611
237	H1	H	0.366908852003	0.721476879674	0.499927054550
238	H1	H	0.366909818168	0.221479778169	0.999942996271
239	H1	H	0.866909818168	0.278518772584	0.000059902224
240	H1	H	0.866909818168	0.778520221831	0.500076810110
241	H1	H	0.366909818168	0.278520221831	0.500059902224
242	H1	H	0.366909818168	0.778520221831	0.000075843945
243	H1	H	0.999934783869	0.133090181832	0.278522154161
244	H1	H	0.999927054550	0.633090181832	0.778520221831
245	H1	H	0.499934783869	0.133091147997	0.778525052656
246	H1	H	0.499928020715	0.633090181832	0.278520221831
247	H1	H	0.000068114626	0.133090181832	0.721477845839
248	H1	H	0.000066182296	0.633090181832	0.221477845839
249	H1	H	0.500062800719	0.133090181832	0.221477845839
250	H1	H	0.500071979285	0.633090181832	0.721478812004
251	H1	H	0.999932851539	0.866909818168	0.721479778169
252	H1	H	0.999932851539	0.366908852003	0.221475913509
253	H1	H	0.499930919209	0.866909818168	0.221479778169
254	H1	H	0.499937199281	0.366909818168	0.721476879674
255	H1	H	0.000069080791	0.866909818168	0.278521187996
256	H1	H	0.000062800719	0.366909818168	0.778523120326
257	H1	H	0.500072945450	0.866908852003	0.778523120326
258	H1	H	0.500060868389	0.366910784333	0.278518772584
259	H1	H	0.000065216131	0.278522154161	0.866909818168
260	H1	H	0.000069080791	0.778521187996	0.366909818168
261	H1	H	0.500066182296	0.278520221831	0.366909818168
262	H1	H	0.500076810110	0.778520221831	0.866909818168
263	H1	H	0.999927054550	0.721478812004	0.866909818168
264	H1	H	0.999932851539	0.221475913509	0.366908852003
265	H1	H	0.499930919209	0.721481227416	0.366909818168
266	H1	H	0.499937199281	0.221477845839	0.866909818168
267	H1	H	0.000062800719	0.721477845839	0.133090181832
268	H1	H	0.000067148461	0.221477845839	0.633090181832
269	H1	H	0.500071013121	0.721477845839	0.633090181832
270	H1	H	0.500066182296	0.221482193581	0.133089215667

271	H1	H	0.999931885374	0.278524086491	0.133091147997
272	H1	H	0.999931885374	0.778517806419	0.633090181832
273	H1	H	0.499936233116	0.278523120326	0.633090181832
274	H1	H	0.499940097776	0.778513941760	0.133087766420
275	H1	H	0.278520221831	0.133090181832	0.999937199281
276	H1	H	0.278521187996	0.633090181832	0.499927054550
277	H1	H	0.778520221831	0.133090181832	0.499939131611
278	H1	H	0.778521187996	0.633090181832	0.999919808313
279	H1	H	0.721479778169	0.133090181832	0.000069080791
280	H1	H	0.721478812004	0.633090181832	0.500069080791
281	H1	H	0.221478812004	0.133090181832	0.500067148461
282	H1	H	0.221479778169	0.633090181832	0.000062800719
283	H1	H	0.721479778169	0.866909818168	0.999932851539
284	H1	H	0.721479778169	0.366909818168	0.499932851539
285	H1	H	0.221477845839	0.866909818168	0.499932851539
286	H1	H	0.221481227416	0.366909818168	0.999928020715
287	H1	H	0.278522154161	0.866909818168	0.000071013121
288	H1	H	0.278517806419	0.366910784333	0.500060868389
289	H1	H	0.778522154161	0.866909818168	0.500075843945
290	H1	H	0.778516840254	0.366910784333	0.000054105235
291	H1	H	0.133090181832	0.000062800719	0.721479778169
292	H1	H	0.133090181832	0.500071979285	0.221477845839
293	H1	H	0.633091147997	0.000060868389	0.221476879674
294	H1	H	0.633090181832	0.500073911615	0.721481227416
295	H1	H	0.133090181832	0.999928986879	0.278518772584
296	H1	H	0.133090181832	0.499940097776	0.778522154161
297	H1	H	0.633090181832	0.999925122220	0.778521187996
298	H1	H	0.633090181832	0.499937199281	0.278522154161
299	H1	H	0.866909818168	0.000063766884	0.278520221831
300	H1	H	0.866909818168	0.500074877780	0.778521187996
301	H1	H	0.366909818168	0.000061834554	0.778520221831
302	H1	H	0.366909818168	0.500068114626	0.278521187996
303	H1	H	0.866909818168	0.999928986879	0.721479778169
304	H1	H	0.866909818168	0.499939131611	0.221478812004
305	H1	H	0.366909818168	0.999928020715	0.221478812004
306	H1	H	0.366909818168	0.499939131611	0.721481227416
307	H2	H	0.416491951846	0.583522057545	0.916476010125
308	H2	H	0.583485826361	0.416490985681	0.916450889838
309	H2	H	0.416535912350	0.416462966899	0.083542830090
310	H2	H	0.583589206006	0.583358775676	0.083564085718
311	H2	H	0.916463933064	0.583469884640	0.416461034569
312	H2	H	0.916474077796	0.416492918011	0.583523989875
313	H2	H	0.083478097042	0.583524956039	0.583547177832
314	H2	H	0.083468918475	0.416468763889	0.416455237580
315	H2	H	0.583492106433	0.916470213136	0.416470213136
316	H2	H	0.416479874785	0.916482773280	0.583514811308
317	H2	H	0.416459102240	0.083442832023	0.416443160519
318	H2	H	0.583534134606	0.083457807579	0.583551042492
319	H2	H	0.083551042492	0.083537999266	0.083455875249
320	H2	H	0.916456203745	0.916471179301	0.083484860196
321	H2	H	0.916459102240	0.083519159050	0.916507893567
322	H2	H	0.083518192885	0.916479874785	0.916479874785
323	O1	O	0.170470135843	0.000000000000	0.095300090820
324	O1	O	0.170470135843	0.500000000000	0.595300090820

325	O1	O	0.670470135843	0.000000000000	0.595300090820
326	O1	O	0.670470135843	0.500000000000	0.095300090820
327	O1	O	0.829529864157	0.000000000000	0.095300090820
328	O1	O	0.829529864157	0.500000000000	0.595300090820
329	O1	O	0.329529864157	0.000000000000	0.595300090820
330	O1	O	0.329529864157	0.500000000000	0.095300090820
331	O1	O	0.829529864157	0.000000000000	0.904699909180
332	O1	O	0.829529864157	0.500000000000	0.404699909180
333	O1	O	0.329529864157	0.000000000000	0.404699909180
334	O1	O	0.329529864157	0.500000000000	0.904699909180
335	O1	O	0.170470135843	0.000000000000	0.904699909180
336	O1	O	0.170470135843	0.500000000000	0.404699909180
337	O1	O	0.670470135843	0.000000000000	0.404699909180
338	O1	O	0.670470135843	0.500000000000	0.904699909180
339	O1	O	0.095300090820	0.170470135843	0.000000000000
340	O1	O	0.095300090820	0.670470135843	0.500000000000
341	O1	O	0.595300090820	0.170470135843	0.500000000000
342	O1	O	0.595300090820	0.670470135843	0.000000000000
343	O1	O	0.095300090820	0.829529864157	0.000000000000
344	O1	O	0.095300090820	0.329529864157	0.500000000000
345	O1	O	0.595300090820	0.829529864157	0.500000000000
346	O1	O	0.595300090820	0.329529864157	0.000000000000
347	O1	O	0.904699909180	0.829529864157	0.000000000000
348	O1	O	0.904699909180	0.329529864157	0.500000000000
349	O1	O	0.404699909180	0.829529864157	0.500000000000
350	O1	O	0.404699909180	0.329529864157	0.000000000000
351	O1	O	0.904699909180	0.170470135843	0.000000000000
352	O1	O	0.904699909180	0.670470135843	0.500000000000
353	O1	O	0.404699909180	0.170470135843	0.500000000000
354	O1	O	0.404699909180	0.670470135843	0.000000000000
355	O1	O	0.000000000000	0.095300090820	0.170470135843
356	O1	O	0.000000000000	0.595300090820	0.670470135843
357	O1	O	0.500000000000	0.095300090820	0.670470135843
358	O1	O	0.500000000000	0.595300090820	0.170470135843
359	O1	O	0.000000000000	0.095300090820	0.829529864157
360	O1	O	0.000000000000	0.595300090820	0.329529864157
361	O1	O	0.500000000000	0.095300090820	0.329529864157
362	O1	O	0.500000000000	0.595300090820	0.829529864157
363	O1	O	0.000000000000	0.904699909180	0.829529864157
364	O1	O	0.000000000000	0.404699909180	0.329529864157
365	O1	O	0.500000000000	0.904699909180	0.329529864157
366	O1	O	0.500000000000	0.404699909180	0.829529864157
367	O1	O	0.000000000000	0.904699909180	0.170470135843
368	O1	O	0.000000000000	0.404699909180	0.670470135843
369	O1	O	0.500000000000	0.904699909180	0.670470135843
370	O1	O	0.500000000000	0.404699909180	0.170470135843
371	O1	O	0.000000000000	0.170470135843	0.904699909180
372	O1	O	0.000000000000	0.670470135843	0.404699909180
373	O1	O	0.500000000000	0.170470135843	0.404699909180
374	O1	O	0.500000000000	0.670470135843	0.904699909180
375	O1	O	0.000000000000	0.829529864157	0.904699909180
376	O1	O	0.000000000000	0.329529864157	0.404699909180
377	O1	O	0.500000000000	0.829529864157	0.404699909180
378	O1	O	0.500000000000	0.329529864157	0.904699909180

379	O1	O	0.000000000000	0.829529864157	0.095300090820
380	O1	O	0.000000000000	0.329529864157	0.595300090820
381	O1	O	0.500000000000	0.829529864157	0.595300090820
382	O1	O	0.500000000000	0.329529864157	0.095300090820
383	O1	O	0.000000000000	0.170470135843	0.095300090820
384	O1	O	0.000000000000	0.670470135843	0.595300090820
385	O1	O	0.500000000000	0.170470135843	0.595300090820
386	O1	O	0.500000000000	0.670470135843	0.095300090820
387	O1	O	0.170470135843	0.095300090820	0.000000000000
388	O1	O	0.170470135843	0.595300090820	0.500000000000
389	O1	O	0.670470135843	0.095300090820	0.500000000000
390	O1	O	0.670470135843	0.595300090820	0.000000000000
391	O1	O	0.829529864157	0.095300090820	0.000000000000
392	O1	O	0.829529864157	0.595300090820	0.500000000000
393	O1	O	0.329529864157	0.095300090820	0.500000000000
394	O1	O	0.329529864157	0.595300090820	0.000000000000
395	O1	O	0.829529864157	0.904699909180	0.000000000000
396	O1	O	0.829529864157	0.404699909180	0.500000000000
397	O1	O	0.329529864157	0.904699909180	0.500000000000
398	O1	O	0.329529864157	0.404699909180	0.000000000000
399	O1	O	0.170470135843	0.904699909180	0.000000000000
400	O1	O	0.170470135843	0.404699909180	0.500000000000
401	O1	O	0.670470135843	0.904699909180	0.500000000000
402	O1	O	0.670470135843	0.404699909180	0.000000000000
403	O1	O	0.095300090820	0.000000000000	0.829529864157
404	O1	O	0.095300090820	0.500000000000	0.329529864157
405	O1	O	0.595300090820	0.000000000000	0.329529864157
406	O1	O	0.595300090820	0.500000000000	0.829529864157
407	O1	O	0.095300090820	0.000000000000	0.170470135843
408	O1	O	0.095300090820	0.500000000000	0.670470135843
409	O1	O	0.595300090820	0.000000000000	0.670470135843
410	O1	O	0.595300090820	0.500000000000	0.170470135843
411	O1	O	0.904699909180	0.000000000000	0.170470135843
412	O1	O	0.904699909180	0.500000000000	0.670470135843
413	O1	O	0.404699909180	0.000000000000	0.670470135843
414	O1	O	0.404699909180	0.500000000000	0.170470135843
415	O1	O	0.904699909180	0.000000000000	0.829529864157
416	O1	O	0.904699909180	0.500000000000	0.329529864157
417	O1	O	0.404699909180	0.000000000000	0.329529864157
418	O1	O	0.404699909180	0.500000000000	0.829529864157
419	O2	O	0.056099882128	0.943900117872	0.943900117872
420	O2	O	0.056099882128	0.443900117872	0.443900117872
421	O2	O	0.556099882128	0.943900117872	0.443900117872
422	O2	O	0.556099882128	0.443900117872	0.943900117872
423	O2	O	0.943900117872	0.056099882128	0.943900117872
424	O2	O	0.943900117872	0.556099882128	0.443900117872
425	O2	O	0.443900117872	0.056099882128	0.443900117872
426	O2	O	0.443900117872	0.556099882128	0.943900117872
427	O2	O	0.943900117872	0.943900117872	0.056099882128
428	O2	O	0.943900117872	0.443900117872	0.556099882128
429	O2	O	0.443900117872	0.943900117872	0.556099882128
430	O2	O	0.443900117872	0.443900117872	0.056099882128
431	O2	O	0.056099882128	0.056099882128	0.056099882128
432	O2	O	0.056099882128	0.556099882128	0.556099882128

433	O2	O	0.556099882128	0.056099882128	0.556099882128
434	O2	O	0.556099882128	0.556099882128	0.056099882128
435	O1	O	0.943900117872	0.056099882128	0.056099882128
436	O1	O	0.943900117872	0.556099882128	0.556099882128
437	O1	O	0.443900117872	0.056099882128	0.556099882128
438	O1	O	0.443900117872	0.556099882128	0.056099882128
439	O1	O	0.056099882128	0.943900117872	0.056099882128
440	O1	O	0.056099882128	0.443900117872	0.556099882128
441	O1	O	0.556099882128	0.943900117872	0.556099882128
442	O1	O	0.556099882128	0.443900117872	0.056099882128
443	O1	O	0.943900117872	0.943900117872	0.943900117872
444	O1	O	0.943900117872	0.443900117872	0.443900117872
445	O1	O	0.443900117872	0.943900117872	0.443900117872
446	O1	O	0.443900117872	0.443900117872	0.943900117872
447	O1	O	0.056099882128	0.056099882128	0.943900117872
448	O1	O	0.056099882128	0.556099882128	0.443900117872
449	O1	O	0.556099882128	0.056099882128	0.443900117872
450	O1	O	0.556099882128	0.556099882128	0.943900117872
451	Zr1	Zr	0.119889953817	0.000000000000	0.000000000000
452	Zr1	Zr	0.119889953817	0.500000000000	0.500000000000
453	Zr1	Zr	0.619889953817	0.000000000000	0.500000000000
454	Zr1	Zr	0.619889953817	0.500000000000	0.000000000000
455	Zr1	Zr	0.880110046183	0.000000000000	0.000000000000
456	Zr1	Zr	0.880110046183	0.500000000000	0.500000000000
457	Zr1	Zr	0.380110046183	0.000000000000	0.500000000000
458	Zr1	Zr	0.380110046183	0.500000000000	0.000000000000
459	Zr1	Zr	0.000000000000	0.119889953817	0.000000000000
460	Zr1	Zr	0.000000000000	0.619889953817	0.500000000000
461	Zr1	Zr	0.500000000000	0.119889953817	0.500000000000
462	Zr1	Zr	0.500000000000	0.619889953817	0.000000000000
463	Zr1	Zr	0.000000000000	0.880110046183	0.000000000000
464	Zr1	Zr	0.000000000000	0.380110046183	0.500000000000
465	Zr1	Zr	0.500000000000	0.880110046183	0.500000000000
466	Zr1	Zr	0.500000000000	0.380110046183	0.000000000000
467	Zr1	Zr	0.000000000000	0.000000000000	0.119889953817
468	Zr1	Zr	0.000000000000	0.500000000000	0.619889953817
469	Zr1	Zr	0.500000000000	0.000000000000	0.619889953817
470	Zr1	Zr	0.500000000000	0.500000000000	0.119889953817
471	Zr1	Zr	0.000000000000	0.000000000000	0.880110046183
472	Zr1	Zr	0.000000000000	0.500000000000	0.380110046183
473	Zr1	Zr	0.500000000000	0.000000000000	0.380110046183
474	Zr1	Zr	0.500000000000	0.500000000000	0.880110046183
475					
