# Supplementary material for: Defect-induced tuning of polarity-dependent adsorption in hydrophobic–hydrophilic UiO-66
Source: Commun Chem. 2022 Oct 7;5:120. doi: 10.1038/s42004-022-00742-z (PMC9814431; doi:10.1038/s42004-022-00742-z)
Supplement: Supplementary file 5 — Supplementary Data 2 [file 42004_2022_742_MOESM5_ESM.pdf]

```
data_UiO-66_1

_cell_length_a                    41.4008
_cell_length_b                    41.4008
_cell_length_c                    41.4008
_cell_angle_alpha                 90.0000
_cell_angle_beta                  90.0000
_cell_angle_gamma                 90.0000

_symmetry_equiv_pos_as_xyz 'x,y,z'

loop_
_atom_site_label
_atom_site_type_symbol
_atom_site_fract_x
_atom_site_fract_y
_atom_site_fract_z
C2   C     0.13375   0.00000   0.09220
C2   C     0.13375   0.25000   0.34220
C2   C     0.38375   0.00000   0.34220
C2   C     0.38375   0.25000   0.09220
C2   C     0.36625   0.00000   0.09220
C2   C     0.36625   0.25000   0.34220
C2   C     0.11625   0.00000   0.34220
C2   C     0.11625   0.25000   0.09220
C2   C     0.36625   0.00000   0.40780
C2   C     0.36625   0.25000   0.15780
C2   C     0.11625   0.00000   0.15780
C2   C     0.11625   0.25000   0.40780
C2   C     0.13375   0.00000   0.40780
C2   C     0.13375   0.25000   0.15780
C2   C     0.38375   0.00000   0.15780
C2   C     0.38375   0.25000   0.40780
C2   C     0.09220   0.13375   0.00000
C2   C     0.09220   0.38375   0.25000
C2   C     0.34220   0.13375   0.25000
C2   C     0.34220   0.38375   0.00000
C2   C     0.09220   0.36625   0.00000
C2   C     0.09220   0.11625   0.25000
C2   C     0.34220   0.36625   0.25000
C2   C     0.34220   0.11625   0.00000
C2   C     0.40780   0.36625   0.00000
C2   C     0.40780   0.11625   0.25000
C2   C     0.15780   0.36625   0.25000
C2   C     0.15780   0.11625   0.00000
C2   C     0.40780   0.13375   0.00000
C2   C     0.40780   0.38375   0.25000
C2   C     0.15780   0.13375   0.25000
C2   C     0.15780   0.38375   0.00000
C2   C     0.00000   0.09220   0.13375
C2   C     0.00000   0.34220   0.38375
C2   C     0.25000   0.09220   0.38375
C2   C     0.25000   0.34220   0.13375
C2   C     0.00000   0.09220   0.36625
```

55	C2	C	0.00000	0.34220	0.11625
56	C2	C	0.25000	0.09220	0.11625
57	C2	C	0.25000	0.34220	0.36625
58	C2	C	0.00000	0.40780	0.36625
59	C2	C	0.00000	0.15780	0.11625
60	C2	C	0.25000	0.40780	0.11625
61	C2	C	0.25000	0.15780	0.36625
62	C2	C	0.00000	0.40780	0.13375
63	C2	C	0.00000	0.15780	0.38375
64	C2	C	0.25000	0.40780	0.38375
65	C2	C	0.25000	0.15780	0.13375
66	C2	C	0.00000	0.13375	0.40780
67	C2	C	0.00000	0.38375	0.15780
68	C2	C	0.25000	0.13375	0.15780
69	C2	C	0.25000	0.38375	0.40780
70	C2	C	0.00000	0.36625	0.40780
71	C2	C	0.00000	0.11625	0.15780
72	C2	C	0.25000	0.36625	0.15780
73	C2	C	0.25000	0.11625	0.40780
74	C2	C	0.00000	0.36625	0.09220
75	C2	C	0.00000	0.11625	0.34220
76	C2	C	0.25000	0.36625	0.34220
77	C2	C	0.25000	0.11625	0.09220
78	C2	C	0.00000	0.13375	0.09220
79	C2	C	0.00000	0.38375	0.34220
80	C2	C	0.25000	0.13375	0.34220
81	C2	C	0.25000	0.38375	0.09220
82	C2	C	0.13375	0.09220	0.00000
83	C2	C	0.13375	0.34220	0.25000
84	C2	C	0.38375	0.09220	0.25000
85	C2	C	0.38375	0.34220	0.00000
86	C2	C	0.36625	0.09220	0.00000
87	C2	C	0.36625	0.34220	0.25000
88	C2	C	0.11625	0.09220	0.25000
89	C2	C	0.11625	0.34220	0.00000
90	C2	C	0.36625	0.40780	0.00000
91	C2	C	0.36625	0.15780	0.25000
92	C2	C	0.11625	0.40780	0.25000
93	C2	C	0.11625	0.15780	0.00000
94	C2	C	0.13375	0.40780	0.00000
95	C2	C	0.13375	0.15780	0.25000
96	C2	C	0.38375	0.40780	0.25000
97	C2	C	0.38375	0.15780	0.00000
98	C2	C	0.09220	0.00000	0.36625
99	C2	C	0.09220	0.25000	0.11625
100	C2	C	0.34220	0.00000	0.11625
101	C2	C	0.34220	0.25000	0.36625
102	C2	C	0.09220	0.00000	0.13375
103	C2	C	0.09220	0.25000	0.38375
104	C2	C	0.34220	0.00000	0.38375
105	C2	C	0.34220	0.25000	0.13375
106	C2	C	0.40780	0.00000	0.13375
107	C2	C	0.40780	0.25000	0.38375
108	C2	C	0.15780	0.00000	0.38375

109	C2	C	0.15780	0.25000	0.13375
110	C2	C	0.40780	0.00000	0.36625
111	C2	C	0.40780	0.25000	0.11625
112	C2	C	0.15780	0.00000	0.11625
113	C2	C	0.15780	0.25000	0.36625
114	C1	C	0.07680	0.00000	0.42320
115	C1	C	0.07680	0.25000	0.17320
116	C1	C	0.32680	0.00000	0.17320
117	C1	C	0.32680	0.25000	0.42320
118	C1	C	0.42320	0.00000	0.42320
119	C1	C	0.42320	0.25000	0.17320
120	C1	C	0.17320	0.00000	0.17320
121	C1	C	0.17320	0.25000	0.42320
122	C1	C	0.42320	0.00000	0.07680
123	C1	C	0.42320	0.25000	0.32680
124	C1	C	0.17320	0.00000	0.32680
125	C1	C	0.17320	0.25000	0.07680
126	C1	C	0.07680	0.00000	0.07680
127	C1	C	0.07680	0.25000	0.32680
128	C1	C	0.32680	0.00000	0.32680
129	C1	C	0.32680	0.25000	0.07680
130	C1	C	0.42320	0.07680	0.00000
131	C1	C	0.42320	0.32680	0.25000
132	C1	C	0.17320	0.07680	0.25000
133	C1	C	0.17320	0.32680	0.00000
134	C1	C	0.42320	0.42320	0.00000
135	C1	C	0.42320	0.17320	0.25000
136	C1	C	0.17320	0.42320	0.25000
137	C1	C	0.17320	0.17320	0.00000
138	C1	C	0.07680	0.42320	0.00000
139	C1	C	0.07680	0.17320	0.25000
140	C1	C	0.32680	0.42320	0.25000
141	C1	C	0.32680	0.17320	0.00000
142	C1	C	0.07680	0.07680	0.00000
143	C1	C	0.07680	0.32680	0.25000
144	C1	C	0.32680	0.07680	0.25000
145	C1	C	0.32680	0.32680	0.00000
146	C1	C	0.00000	0.42320	0.07680
147	C1	C	0.00000	0.17320	0.32680
148	C1	C	0.25000	0.42320	0.32680
149	C1	C	0.25000	0.17320	0.07680
150	C1	C	0.00000	0.42320	0.42320
151	C1	C	0.00000	0.17320	0.17320
152	C1	C	0.25000	0.42320	0.17320
153	C1	C	0.25000	0.17320	0.42320
154	C1	C	0.00000	0.07680	0.42320
155	C1	C	0.00000	0.32680	0.17320
156	C1	C	0.25000	0.07680	0.17320
157	C1	C	0.25000	0.32680	0.42320
158	C1	C	0.00000	0.07680	0.07680
159	C1	C	0.00000	0.32680	0.32680
160	C1	C	0.25000	0.07680	0.32680
161	C1	C	0.25000	0.32680	0.07680
162	C3	C	0.10250	0.00000	0.10250

163	C3	C	0.10250	0.25000	0.35250
164	C3	C	0.35250	0.00000	0.35250
165	C3	C	0.35250	0.25000	0.10250
166	C3	C	0.39750	0.00000	0.10250
167	C3	C	0.39750	0.25000	0.35250
168	C3	C	0.14750	0.00000	0.35250
169	C3	C	0.14750	0.25000	0.10250
170	C3	C	0.39750	0.00000	0.39750
171	C3	C	0.39750	0.25000	0.14750
172	C3	C	0.14750	0.00000	0.14750
173	C3	C	0.14750	0.25000	0.39750
174	C3	C	0.10250	0.00000	0.39750
175	C3	C	0.10250	0.25000	0.14750
176	C3	C	0.35250	0.00000	0.14750
177	C3	C	0.35250	0.25000	0.39750
178	C3	C	0.10250	0.10250	0.00000
179	C3	C	0.10250	0.35250	0.25000
180	C3	C	0.35250	0.10250	0.25000
181	C3	C	0.35250	0.35250	0.00000
182	C3	C	0.10250	0.39750	0.00000
183	C3	C	0.10250	0.14750	0.25000
184	C3	C	0.35250	0.39750	0.25000
185	C3	C	0.35250	0.14750	0.00000
186	C3	C	0.39750	0.39750	0.00000
187	C3	C	0.39750	0.14750	0.25000
188	C3	C	0.14750	0.39750	0.25000
189	C3	C	0.14750	0.14750	0.00000
190	C3	C	0.39750	0.10250	0.00000
191	C3	C	0.39750	0.35250	0.25000
192	C3	C	0.14750	0.10250	0.25000
193	C3	C	0.14750	0.35250	0.00000
194	C3	C	0.00000	0.10250	0.10250
195	C3	C	0.00000	0.35250	0.35250
196	C3	C	0.25000	0.10250	0.35250
197	C3	C	0.25000	0.35250	0.10250
198	C3	C	0.00000	0.10250	0.39750
199	C3	C	0.00000	0.35250	0.14750
200	C3	C	0.25000	0.10250	0.14750
201	C3	C	0.25000	0.35250	0.39750
202	C3	C	0.00000	0.39750	0.39750
203	C3	C	0.00000	0.14750	0.14750
204	C3	C	0.25000	0.39750	0.14750
205	C3	C	0.25000	0.14750	0.39750
206	C3	C	0.00000	0.39750	0.10250
207	C3	C	0.00000	0.14750	0.35250
208	C3	C	0.25000	0.39750	0.35250
209	C3	C	0.25000	0.14750	0.10250
210	H1	H	0.13926	0.49997	0.06655
211	H1	H	0.13926	0.24997	0.31655
212	H1	H	0.38926	0.49997	0.31655
213	H1	H	0.38926	0.24997	0.06655
214	H1	H	0.36074	0.00003	0.06655
215	H1	H	0.36074	0.25004	0.31655
216	H1	H	0.11074	0.00003	0.31655

217	H1	H	0.11074	0.25004	0.06655
218	H1	H	0.36074	0.49996	0.43345
219	H1	H	0.36074	0.24997	0.18345
220	H1	H	0.11074	0.49997	0.18345
221	H1	H	0.11074	0.24997	0.43346
222	H1	H	0.13926	0.00003	0.43345
223	H1	H	0.13926	0.25003	0.18346
224	H1	H	0.38926	0.00003	0.18345
225	H1	H	0.38926	0.25004	0.43346
226	H1	H	0.06655	0.13926	0.49996
227	H1	H	0.06655	0.38926	0.24997
228	H1	H	0.31655	0.13926	0.24996
229	H1	H	0.31654	0.38926	0.49997
230	H1	H	0.06655	0.36074	0.00003
231	H1	H	0.06655	0.11074	0.25004
232	H1	H	0.31655	0.36074	0.25003
233	H1	H	0.31655	0.11074	0.00004
234	H1	H	0.43345	0.36074	0.49996
235	H1	H	0.43345	0.11074	0.24997
236	H1	H	0.18345	0.36074	0.24996
237	H1	H	0.18345	0.11074	0.49997
238	H1	H	0.43345	0.13926	0.00003
239	H1	H	0.43345	0.38926	0.25004
240	H1	H	0.18345	0.13926	0.25003
241	H1	H	0.18345	0.38926	0.00004
242	H1	H	0.49997	0.06655	0.13926
243	H1	H	0.49996	0.31655	0.38926
244	H1	H	0.24997	0.06655	0.38926
245	H1	H	0.24996	0.31655	0.13926
246	H1	H	0.00003	0.06655	0.36074
247	H1	H	0.00003	0.31655	0.11074
248	H1	H	0.25003	0.06655	0.11074
249	H1	H	0.25004	0.31655	0.36074
250	H1	H	0.49997	0.43345	0.36074
251	H1	H	0.49997	0.18345	0.11074
252	H1	H	0.24997	0.43345	0.11074
253	H1	H	0.24997	0.18345	0.36074
254	H1	H	0.00003	0.43345	0.13926
255	H1	H	0.00003	0.18345	0.38926
256	H1	H	0.25004	0.43345	0.38926
257	H1	H	0.25003	0.18346	0.13926
258	H1	H	0.00003	0.13926	0.43345
259	H1	H	0.00003	0.38926	0.18345
260	H1	H	0.25003	0.13926	0.18345
261	H1	H	0.25004	0.38926	0.43345
262	H1	H	0.49996	0.36074	0.43345
263	H1	H	0.49997	0.11074	0.18345
264	H1	H	0.24997	0.36074	0.18345
265	H1	H	0.24997	0.11074	0.43345
266	H1	H	0.00003	0.36074	0.06655
267	H1	H	0.00003	0.11074	0.31655
268	H1	H	0.25004	0.36074	0.31655
269	H1	H	0.25003	0.11074	0.06654
270	H1	H	0.49997	0.13926	0.06655

271	H1	H	0.49997	0.38926	0.31655
272	H1	H	0.24997	0.13926	0.31655
273	H1	H	0.24997	0.38926	0.06654
274	H1	H	0.13926	0.06655	0.49997
275	H1	H	0.13926	0.31655	0.24996
276	H1	H	0.38926	0.06655	0.24997
277	H1	H	0.38926	0.31655	0.49996
278	H1	H	0.36074	0.06655	0.00003
279	H1	H	0.36074	0.31655	0.25003
280	H1	H	0.11074	0.06655	0.25003
281	H1	H	0.11074	0.31655	0.00003
282	H1	H	0.36074	0.43345	0.49997
283	H1	H	0.36074	0.18345	0.24997
284	H1	H	0.11074	0.43345	0.24997
285	H1	H	0.11074	0.18345	0.49996
286	H1	H	0.13926	0.43345	0.00004
287	H1	H	0.13926	0.18346	0.25003
288	H1	H	0.38926	0.43345	0.25004
289	H1	H	0.38926	0.18346	0.00003
290	H1	H	0.06655	0.00003	0.36074
291	H1	H	0.06655	0.25004	0.11074
292	H1	H	0.31655	0.00003	0.11074
293	H1	H	0.31655	0.25004	0.36074
294	H1	H	0.06655	0.49996	0.13926
295	H1	H	0.06655	0.24997	0.38926
296	H1	H	0.31655	0.49996	0.38926
297	H1	H	0.31655	0.24997	0.13926
298	H1	H	0.43345	0.00003	0.13926
299	H1	H	0.43345	0.25004	0.38926
300	H1	H	0.18345	0.00003	0.38926
301	H1	H	0.18345	0.25003	0.13926
302	H1	H	0.43345	0.49996	0.36074
303	H1	H	0.43345	0.24997	0.11074
304	H1	H	0.18345	0.49996	0.11074
305	H1	H	0.18345	0.24997	0.36074
306	H2	H	0.20825	0.29176	0.45824
307	H2	H	0.29174	0.20825	0.45823
308	H2	H	0.20827	0.20823	0.04177
309	H2	H	0.29179	0.29168	0.04178
310	H2	H	0.45823	0.29173	0.20823
311	H2	H	0.45824	0.20825	0.29176
312	H2	H	0.04174	0.29176	0.29177
313	H2	H	0.04173	0.20823	0.20823
314	H2	H	0.29175	0.45824	0.20824
315	H2	H	0.20824	0.45824	0.29176
316	H2	H	0.20823	0.04172	0.20822
317	H2	H	0.29177	0.04173	0.29178
318	H2	H	0.04178	0.04177	0.04173
319	H2	H	0.45823	0.45824	0.04174
320	H2	H	0.45823	0.04176	0.45825
321	H2	H	0.04176	0.45824	0.45824
322	O1	O	0.08524	0.00000	0.04765
323	O1	O	0.08524	0.25000	0.29765
324	O1	O	0.33524	0.00000	0.29765

325	O1	O	0.33524	0.25000	0.04765
326	O1	O	0.41476	0.00000	0.04765
327	O1	O	0.41476	0.25000	0.29765
328	O1	O	0.16476	0.00000	0.29765
329	O1	O	0.16476	0.25000	0.04765
330	O1	O	0.41476	0.00000	0.45235
331	O1	O	0.41476	0.25000	0.20235
332	O1	O	0.16476	0.00000	0.20235
333	O1	O	0.16476	0.25000	0.45235
334	O1	O	0.08524	0.00000	0.45235
335	O1	O	0.08524	0.25000	0.20235
336	O1	O	0.33524	0.00000	0.20235
337	O1	O	0.33524	0.25000	0.45235
338	O1	O	0.04765	0.08524	0.00000
339	O1	O	0.04765	0.33524	0.25000
340	O1	O	0.29765	0.08524	0.25000
341	O1	O	0.29765	0.33524	0.00000
342	O1	O	0.04765	0.41476	0.00000
343	O1	O	0.04765	0.16476	0.25000
344	O1	O	0.29765	0.41476	0.25000
345	O1	O	0.29765	0.16476	0.00000
346	O1	O	0.45235	0.41476	0.00000
347	O1	O	0.45235	0.16476	0.25000
348	O1	O	0.20235	0.41476	0.25000
349	O1	O	0.20235	0.16476	0.00000
350	O1	O	0.45235	0.08524	0.00000
351	O1	O	0.45235	0.33524	0.25000
352	O1	O	0.20235	0.08524	0.25000
353	O1	O	0.20235	0.33524	0.00000
354	O1	O	0.00000	0.04765	0.08524
355	O1	O	0.00000	0.29765	0.33524
356	O1	O	0.25000	0.04765	0.33524
357	O1	O	0.25000	0.29765	0.08524
358	O1	O	0.00000	0.04765	0.41476
359	O1	O	0.00000	0.29765	0.16476
360	O1	O	0.25000	0.04765	0.16476
361	O1	O	0.25000	0.29765	0.41476
362	O1	O	0.00000	0.45235	0.41476
363	O1	O	0.00000	0.20235	0.16476
364	O1	O	0.25000	0.45235	0.16476
365	O1	O	0.25000	0.20235	0.41476
366	O1	O	0.00000	0.45235	0.08524
367	O1	O	0.00000	0.20235	0.33524
368	O1	O	0.25000	0.45235	0.33524
369	O1	O	0.25000	0.20235	0.08524
370	O1	O	0.00000	0.08524	0.45235
371	O1	O	0.00000	0.33524	0.20235
372	O1	O	0.25000	0.08524	0.20235
373	O1	O	0.25000	0.33524	0.45235
374	O1	O	0.00000	0.41476	0.45235
375	O1	O	0.00000	0.16476	0.20235
376	O1	O	0.25000	0.41476	0.20235
377	O1	O	0.25000	0.16476	0.45235
378	O1	O	0.00000	0.41476	0.04765

379	O1	O	0.00000	0.16476	0.29765
380	O1	O	0.25000	0.41476	0.29765
381	O1	O	0.25000	0.16476	0.04765
382	O1	O	0.00000	0.08524	0.04765
383	O1	O	0.00000	0.33524	0.29765
384	O1	O	0.25000	0.08524	0.29765
385	O1	O	0.25000	0.33524	0.04765
386	O1	O	0.08524	0.04765	0.00000
387	O1	O	0.08524	0.29765	0.25000
388	O1	O	0.33524	0.04765	0.25000
389	O1	O	0.33524	0.29765	0.00000
390	O1	O	0.41476	0.04765	0.00000
391	O1	O	0.41476	0.29765	0.25000
392	O1	O	0.16476	0.04765	0.25000
393	O1	O	0.16476	0.29765	0.00000
394	O1	O	0.41476	0.45235	0.00000
395	O1	O	0.41476	0.20235	0.25000
396	O1	O	0.16476	0.45235	0.25000
397	O1	O	0.16476	0.20235	0.00000
398	O1	O	0.08524	0.45235	0.00000
399	O1	O	0.08524	0.20235	0.25000
400	O1	O	0.33524	0.45235	0.25000
401	O1	O	0.33524	0.20235	0.00000
402	O1	O	0.04765	0.00000	0.41476
403	O1	O	0.04765	0.25000	0.16476
404	O1	O	0.29765	0.00000	0.16476
405	O1	O	0.29765	0.25000	0.41476
406	O1	O	0.04765	0.00000	0.08524
407	O1	O	0.04765	0.25000	0.33524
408	O1	O	0.29765	0.00000	0.33524
409	O1	O	0.29765	0.25000	0.08524
410	O1	O	0.45235	0.00000	0.08524
411	O1	O	0.45235	0.25000	0.33524
412	O1	O	0.20235	0.00000	0.33524
413	O1	O	0.20235	0.25000	0.08524
414	O1	O	0.45235	0.00000	0.41476
415	O1	O	0.45235	0.25000	0.16476
416	O1	O	0.20235	0.00000	0.16476
417	O1	O	0.20235	0.25000	0.41476
418	O2	O	0.02805	0.47195	0.47195
419	O2	O	0.02805	0.22195	0.22195
420	O2	O	0.27805	0.47195	0.22195
421	O2	O	0.27805	0.22195	0.47195
422	O2	O	0.47195	0.02805	0.47195
423	O2	O	0.47195	0.27805	0.22195
424	O2	O	0.22195	0.02805	0.22195
425	O2	O	0.22195	0.27805	0.47195
426	O2	O	0.47195	0.47195	0.02805
427	O2	O	0.47195	0.22195	0.27805
428	O2	O	0.22195	0.47195	0.27805
429	O2	O	0.22195	0.22195	0.02805
430	O2	O	0.02805	0.02805	0.02805
431	O2	O	0.02805	0.27805	0.27805
432	O2	O	0.27805	0.02805	0.27805

433	O2	O	0.27805	0.27805	0.02805
434	O1	O	0.47195	0.02805	0.02805
435	O1	O	0.47195	0.27805	0.27805
436	O1	O	0.22195	0.02805	0.27805
437	O1	O	0.22195	0.27805	0.02805
438	O1	O	0.02805	0.47195	0.02805
439	O1	O	0.02805	0.22195	0.27805
440	O1	O	0.27805	0.47195	0.27805
441	O1	O	0.27805	0.22195	0.02805
442	O1	O	0.47195	0.47195	0.47195
443	O1	O	0.47195	0.22195	0.22195
444	O1	O	0.22195	0.47195	0.22195
445	O1	O	0.22195	0.22195	0.47195
446	O1	O	0.02805	0.02805	0.47195
447	O1	O	0.02805	0.27805	0.22195
448	O1	O	0.27805	0.02805	0.22195
449	O1	O	0.27805	0.27805	0.47195
450	Zr1	Zr	0.05994	0.00000	0.00000
451	Zr1	Zr	0.05994	0.25000	0.25000
452	Zr1	Zr	0.30994	0.00000	0.25000
453	Zr1	Zr	0.30994	0.25000	0.00000
454	Zr1	Zr	0.44006	0.00000	0.00000
455	Zr1	Zr	0.44006	0.25000	0.25000
456	Zr1	Zr	0.19006	0.00000	0.25000
457	Zr1	Zr	0.19006	0.25000	0.00000
458	Zr1	Zr	0.00000	0.05994	0.00000
459	Zr1	Zr	0.00000	0.30994	0.25000
460	Zr1	Zr	0.25000	0.05994	0.25000
461	Zr1	Zr	0.25000	0.30994	0.00000
462	Zr1	Zr	0.00000	0.44006	0.00000
463	Zr1	Zr	0.00000	0.19006	0.25000
464	Zr1	Zr	0.25000	0.44006	0.25000
465	Zr1	Zr	0.25000	0.19006	0.00000
466	Zr1	Zr	0.00000	0.00000	0.05994
467	Zr1	Zr	0.00000	0.25000	0.30994
468	Zr1	Zr	0.25000	0.00000	0.30994
469	Zr1	Zr	0.25000	0.25000	0.05994
470	Zr1	Zr	0.00000	0.00000	0.44006
471	Zr1	Zr	0.00000	0.25000	0.19006
472	Zr1	Zr	0.25000	0.00000	0.19006
473	Zr1	Zr	0.25000	0.25000	0.44006
474	C2	C	0.13375	0.00000	0.59220
475	C2	C	0.13375	0.25000	0.84220
476	C2	C	0.38375	0.00000	0.84220
477	C2	C	0.38375	0.25000	0.59220
478	C2	C	0.36625	0.00000	0.59220
479	C2	C	0.36625	0.25000	0.84220
480	C2	C	0.11625	0.00000	0.84220
481	C2	C	0.11625	0.25000	0.59220
482	C2	C	0.36625	0.00000	0.90780
483	C2	C	0.36625	0.25000	0.65780
484	C2	C	0.11625	0.00000	0.65780
485	C2	C	0.11625	0.25000	0.90780
486	C2	C	0.13375	0.00000	0.90780

487	C2	C	0.13375	0.25000	0.65780
488	C2	C	0.38375	0.00000	0.65780
489	C2	C	0.38375	0.25000	0.90780
490	C2	C	0.09220	0.13375	0.50000
491	C2	C	0.09220	0.38375	0.75000
492	C2	C	0.34220	0.13375	0.75000
493	C2	C	0.34220	0.38375	0.50000
494	C2	C	0.09220	0.36625	0.50000
495	C2	C	0.09220	0.11625	0.75000
496	C2	C	0.34220	0.36625	0.75000
497	C2	C	0.34220	0.11625	0.50000
498	C2	C	0.40780	0.36625	0.50000
499	C2	C	0.40780	0.11625	0.75000
500	C2	C	0.15780	0.36625	0.75000
501	C2	C	0.15780	0.11625	0.50000
502	C2	C	0.40780	0.13375	0.50000
503	C2	C	0.40780	0.38375	0.75000
504	C2	C	0.15780	0.13375	0.75000
505	C2	C	0.15780	0.38375	0.50000
506	C2	C	0.00000	0.09220	0.63375
507	C2	C	0.00000	0.34220	0.88375
508	C2	C	0.25000	0.09220	0.88375
509	C2	C	0.25000	0.34220	0.63375
510	C2	C	0.00000	0.09220	0.86625
511	C2	C	0.00000	0.34220	0.61625
512	C2	C	0.25000	0.09220	0.61625
513	C2	C	0.25000	0.34220	0.86625
514	C2	C	0.00000	0.40780	0.86625
515	C2	C	0.00000	0.15780	0.61625
516	C2	C	0.25000	0.40780	0.61625
517	C2	C	0.25000	0.15780	0.86625
518	C2	C	0.00000	0.40780	0.63375
519	C2	C	0.00000	0.15780	0.88375
520	C2	C	0.25000	0.40780	0.88375
521	C2	C	0.25000	0.15780	0.63375
522	C2	C	0.00000	0.13375	0.90780
523	C2	C	0.00000	0.38375	0.65780
524	C2	C	0.25000	0.13375	0.65780
525	C2	C	0.25000	0.38375	0.90780
526	C2	C	0.00000	0.36625	0.90780
527	C2	C	0.00000	0.11625	0.65780
528	C2	C	0.25000	0.36625	0.65780
529	C2	C	0.25000	0.11625	0.90780
530	C2	C	0.00000	0.36625	0.59220
531	C2	C	0.00000	0.11625	0.84220
532	C2	C	0.25000	0.36625	0.84220
533	C2	C	0.25000	0.11625	0.59220
534	C2	C	0.00000	0.13375	0.59220
535	C2	C	0.00000	0.38375	0.84220
536	C2	C	0.25000	0.13375	0.84220
537	C2	C	0.25000	0.38375	0.59220
538	C2	C	0.13375	0.09220	0.50000
539	C2	C	0.13375	0.34220	0.75000
540	C2	C	0.38375	0.09220	0.75000

541	C2	C	0.38375	0.34220	0.50000
542	C2	C	0.36625	0.09220	0.50000
543	C2	C	0.36625	0.34220	0.75000
544	C2	C	0.11625	0.09220	0.75000
545	C2	C	0.11625	0.34220	0.50000
546	C2	C	0.36625	0.40780	0.50000
547	C2	C	0.36625	0.15780	0.75000
548	C2	C	0.11625	0.40780	0.75000
549	C2	C	0.11625	0.15780	0.50000
550	C2	C	0.13375	0.40780	0.50000
551	C2	C	0.13375	0.15780	0.75000
552	C2	C	0.38375	0.40780	0.75000
553	C2	C	0.38375	0.15780	0.50000
554	C2	C	0.09220	0.00000	0.86625
555	C2	C	0.09220	0.25000	0.61625
556	C2	C	0.34220	0.00000	0.61625
557	C2	C	0.34220	0.25000	0.86625
558	C2	C	0.09220	0.00000	0.63375
559	C2	C	0.09220	0.25000	0.88375
560	C2	C	0.34220	0.00000	0.88375
561	C2	C	0.34220	0.25000	0.63375
562	C2	C	0.40780	0.00000	0.63375
563	C2	C	0.40780	0.25000	0.88375
564	C2	C	0.15780	0.00000	0.88375
565	C2	C	0.15780	0.25000	0.63375
566	C2	C	0.40780	0.00000	0.86625
567	C2	C	0.40780	0.25000	0.61625
568	C2	C	0.15780	0.00000	0.61625
569	C2	C	0.15780	0.25000	0.86625
570	C1	C	0.07680	0.00000	0.92320
571	C1	C	0.07680	0.25000	0.67320
572	C1	C	0.32680	0.00000	0.67320
573	C1	C	0.32680	0.25000	0.92320
574	C1	C	0.42320	0.00000	0.92320
575	C1	C	0.42320	0.25000	0.67320
576	C1	C	0.17320	0.00000	0.67320
577	C1	C	0.17320	0.25000	0.92320
578	C1	C	0.42320	0.00000	0.57680
579	C1	C	0.42320	0.25000	0.82680
580	C1	C	0.17320	0.00000	0.82680
581	C1	C	0.17320	0.25000	0.57680
582	C1	C	0.07680	0.00000	0.57680
583	C1	C	0.07680	0.25000	0.82680
584	C1	C	0.32680	0.00000	0.82680
585	C1	C	0.32680	0.25000	0.57680
586	C1	C	0.42320	0.07680	0.50000
587	C1	C	0.42320	0.32680	0.75000
588	C1	C	0.17320	0.07680	0.75000
589	C1	C	0.17320	0.32680	0.50000
590	C1	C	0.42320	0.42320	0.50000
591	C1	C	0.42320	0.17320	0.75000
592	C1	C	0.17320	0.42320	0.75000
593	C1	C	0.17320	0.17320	0.50000
594	C1	C	0.07680	0.42320	0.50000

595	C1	C	0.07680	0.17320	0.75000
596	C1	C	0.32680	0.42320	0.75000
597	C1	C	0.32680	0.17320	0.50000
598	C1	C	0.07680	0.07680	0.50000
599	C1	C	0.07680	0.32680	0.75000
600	C1	C	0.32680	0.07680	0.75000
601	C1	C	0.32680	0.32680	0.50000
602	C1	C	0.00000	0.42320	0.57680
603	C1	C	0.00000	0.17320	0.82680
604	C1	C	0.25000	0.42320	0.82680
605	C1	C	0.25000	0.17320	0.57680
606	C1	C	0.00000	0.42320	0.92320
607	C1	C	0.00000	0.17320	0.67320
608	C1	C	0.25000	0.42320	0.67320
609	C1	C	0.25000	0.17320	0.92320
610	C1	C	0.00000	0.07680	0.92320
611	C1	C	0.00000	0.32680	0.67320
612	C1	C	0.25000	0.07680	0.67320
613	C1	C	0.25000	0.32680	0.92320
614	C1	C	0.00000	0.07680	0.57680
615	C1	C	0.00000	0.32680	0.82680
616	C1	C	0.25000	0.07680	0.82680
617	C1	C	0.25000	0.32680	0.57680
618	C3	C	0.10250	0.00000	0.60250
619	C3	C	0.10250	0.25000	0.85250
620	C3	C	0.35250	0.00000	0.85250
621	C3	C	0.35250	0.25000	0.60250
622	C3	C	0.39750	0.00000	0.60250
623	C3	C	0.39750	0.25000	0.85250
624	C3	C	0.14750	0.00000	0.85250
625	C3	C	0.14750	0.25000	0.60250
626	C3	C	0.39750	0.00000	0.89750
627	C3	C	0.39750	0.25000	0.64750
628	C3	C	0.14750	0.00000	0.64750
629	C3	C	0.14750	0.25000	0.89750
630	C3	C	0.10250	0.00000	0.89750
631	C3	C	0.10250	0.25000	0.64750
632	C3	C	0.35250	0.00000	0.64750
633	C3	C	0.35250	0.25000	0.89750
634	C3	C	0.10250	0.10250	0.50000
635	C3	C	0.10250	0.35250	0.75000
636	C3	C	0.35250	0.10250	0.75000
637	C3	C	0.35250	0.35250	0.50000
638	C3	C	0.10250	0.39750	0.50000
639	C3	C	0.10250	0.14750	0.75000
640	C3	C	0.35250	0.39750	0.75000
641	C3	C	0.35250	0.14750	0.50000
642	C3	C	0.39750	0.39750	0.50000
643	C3	C	0.39750	0.14750	0.75000
644	C3	C	0.14750	0.39750	0.75000
645	C3	C	0.14750	0.14750	0.50000
646	C3	C	0.39750	0.10250	0.50000
647	C3	C	0.39750	0.35250	0.75000
648	C3	C	0.14750	0.10250	0.75000

649	C3	C	0.14750	0.35250	0.50000
650	C3	C	0.00000	0.10250	0.60250
651	C3	C	0.00000	0.35250	0.85250
652	C3	C	0.25000	0.10250	0.85250
653	C3	C	0.25000	0.35250	0.60250
654	C3	C	0.00000	0.10250	0.89750
655	C3	C	0.00000	0.35250	0.64750
656	C3	C	0.25000	0.10250	0.64750
657	C3	C	0.25000	0.35250	0.89750
658	C3	C	0.00000	0.39750	0.89750
659	C3	C	0.00000	0.14750	0.64750
660	C3	C	0.25000	0.39750	0.64750
661	C3	C	0.25000	0.14750	0.89750
662	C3	C	0.00000	0.39750	0.60250
663	C3	C	0.00000	0.14750	0.85250
664	C3	C	0.25000	0.39750	0.85250
665	C3	C	0.25000	0.14750	0.60250
666	H1	H	0.13926	0.49997	0.56655
667	H1	H	0.13926	0.24997	0.81655
668	H1	H	0.38926	0.49997	0.81655
669	H1	H	0.38926	0.24997	0.56655
670	H1	H	0.36074	0.00003	0.56655
671	H1	H	0.36074	0.25004	0.81655
672	H1	H	0.11074	0.00003	0.81655
673	H1	H	0.11074	0.25004	0.56655
674	H1	H	0.36074	0.49996	0.93345
675	H1	H	0.36074	0.24997	0.68345
676	H1	H	0.11074	0.49997	0.68345
677	H1	H	0.11074	0.24997	0.93346
678	H1	H	0.13926	0.00003	0.93345
679	H1	H	0.13926	0.25003	0.68346
680	H1	H	0.38926	0.00003	0.68345
681	H1	H	0.38926	0.25004	0.93346
682	H1	H	0.06655	0.13926	0.99996
683	H1	H	0.06655	0.38926	0.74997
684	H1	H	0.31655	0.13926	0.74996
685	H1	H	0.31654	0.38926	0.99997
686	H1	H	0.06655	0.36074	0.50003
687	H1	H	0.06655	0.11074	0.75004
688	H1	H	0.31655	0.36074	0.75003
689	H1	H	0.31655	0.11074	0.50004
690	H1	H	0.43345	0.36074	0.99996
691	H1	H	0.43345	0.11074	0.74997
692	H1	H	0.18345	0.36074	0.74996
693	H1	H	0.18345	0.11074	0.99997
694	H1	H	0.43345	0.13926	0.50003
695	H1	H	0.43345	0.38926	0.75004
696	H1	H	0.18345	0.13926	0.75003
697	H1	H	0.18345	0.38926	0.50004
698	H1	H	0.49997	0.06655	0.63926
699	H1	H	0.49996	0.31655	0.88926
700	H1	H	0.24997	0.06655	0.88926
701	H1	H	0.24996	0.31655	0.63926
702	H1	H	0.00003	0.06655	0.86074

703	H1	H	0.00003	0.31655	0.61074
704	H1	H	0.25003	0.06655	0.61074
705	H1	H	0.25004	0.31655	0.86074
706	H1	H	0.49997	0.43345	0.86074
707	H1	H	0.49997	0.18345	0.61074
708	H1	H	0.24997	0.43345	0.61074
709	H1	H	0.24997	0.18345	0.86074
710	H1	H	0.00003	0.43345	0.63926
711	H1	H	0.00003	0.18345	0.88926
712	H1	H	0.25004	0.43345	0.88926
713	H1	H	0.25003	0.18346	0.63926
714	H1	H	0.00003	0.13926	0.93345
715	H1	H	0.00003	0.38926	0.68345
716	H1	H	0.25003	0.13926	0.68345
717	H1	H	0.25004	0.38926	0.93345
718	H1	H	0.49996	0.36074	0.93345
719	H1	H	0.49997	0.11074	0.68345
720	H1	H	0.24997	0.36074	0.68345
721	H1	H	0.24997	0.11074	0.93345
722	H1	H	0.00003	0.36074	0.56655
723	H1	H	0.00003	0.11074	0.81655
724	H1	H	0.25004	0.36074	0.81655
725	H1	H	0.25003	0.11074	0.56654
726	H1	H	0.49997	0.13926	0.56655
727	H1	H	0.49997	0.38926	0.81655
728	H1	H	0.24997	0.13926	0.81655
729	H1	H	0.24997	0.38926	0.56654
730	H1	H	0.13926	0.06655	0.99997
731	H1	H	0.13926	0.31655	0.74996
732	H1	H	0.38926	0.06655	0.74997
733	H1	H	0.38926	0.31655	0.99996
734	H1	H	0.36074	0.06655	0.50003
735	H1	H	0.36074	0.31655	0.75003
736	H1	H	0.11074	0.06655	0.75003
737	H1	H	0.11074	0.31655	0.50003
738	H1	H	0.36074	0.43345	0.99997
739	H1	H	0.36074	0.18345	0.74997
740	H1	H	0.11074	0.43345	0.74997
741	H1	H	0.11074	0.18345	0.99996
742	H1	H	0.13926	0.43345	0.50004
743	H1	H	0.13926	0.18346	0.75003
744	H1	H	0.38926	0.43345	0.75004
745	H1	H	0.38926	0.18346	0.50003
746	H1	H	0.06655	0.00003	0.86074
747	H1	H	0.06655	0.25004	0.61074
748	H1	H	0.31655	0.00003	0.61074
749	H1	H	0.31655	0.25004	0.86074
750	H1	H	0.06655	0.49996	0.63926
751	H1	H	0.06655	0.24997	0.88926
752	H1	H	0.31655	0.49996	0.88926
753	H1	H	0.31655	0.24997	0.63926
754	H1	H	0.43345	0.00003	0.63926
755	H1	H	0.43345	0.25004	0.88926
756	H1	H	0.18345	0.00003	0.88926

757	H1	H	0.18345	0.25003	0.63926
758	H1	H	0.43345	0.49996	0.86074
759	H1	H	0.43345	0.24997	0.61074
760	H1	H	0.18345	0.49996	0.61074
761	H1	H	0.18345	0.24997	0.86074
762	H2	H	0.20825	0.29176	0.95824
763	H2	H	0.29174	0.20825	0.95823
764	H2	H	0.20827	0.20823	0.54177
765	H2	H	0.29179	0.29168	0.54178
766	H2	H	0.45823	0.29173	0.70823
767	H2	H	0.45824	0.20825	0.79176
768	H2	H	0.04174	0.29176	0.79177
769	H2	H	0.04173	0.20823	0.70823
770	H2	H	0.29175	0.45824	0.70824
771	H2	H	0.20824	0.45824	0.79176
772	H2	H	0.20823	0.04172	0.70822
773	H2	H	0.29177	0.04173	0.79178
774	H2	H	0.04178	0.04177	0.54173
775	H2	H	0.45823	0.45824	0.54174
776	H2	H	0.45823	0.04176	0.95825
777	H2	H	0.04176	0.45824	0.95824
778	O1	O	0.08524	0.00000	0.54765
779	O1	O	0.08524	0.25000	0.79765
780	O1	O	0.33524	0.00000	0.79765
781	O1	O	0.33524	0.25000	0.54765
782	O1	O	0.41476	0.00000	0.54765
783	O1	O	0.41476	0.25000	0.79765
784	O1	O	0.16476	0.00000	0.79765
785	O1	O	0.16476	0.25000	0.54765
786	O1	O	0.41476	0.00000	0.95235
787	O1	O	0.41476	0.25000	0.70235
788	O1	O	0.16476	0.00000	0.70235
789	O1	O	0.16476	0.25000	0.95235
790	O1	O	0.08524	0.00000	0.95235
791	O1	O	0.08524	0.25000	0.70235
792	O1	O	0.33524	0.00000	0.70235
793	O1	O	0.33524	0.25000	0.95235
794	O1	O	0.04765	0.08524	0.50000
795	O1	O	0.04765	0.33524	0.75000
796	O1	O	0.29765	0.08524	0.75000
797	O1	O	0.29765	0.33524	0.50000
798	O1	O	0.04765	0.41476	0.50000
799	O1	O	0.04765	0.16476	0.75000
800	O1	O	0.29765	0.41476	0.75000
801	O1	O	0.29765	0.16476	0.50000
802	O1	O	0.45235	0.41476	0.50000
803	O1	O	0.45235	0.16476	0.75000
804	O1	O	0.20235	0.41476	0.75000
805	O1	O	0.20235	0.16476	0.50000
806	O1	O	0.45235	0.08524	0.50000
807	O1	O	0.45235	0.33524	0.75000
808	O1	O	0.20235	0.08524	0.75000
809	O1	O	0.20235	0.33524	0.50000
810	O1	O	0.00000	0.04765	0.58524

811	O1	O	0.00000	0.29765	0.83524
812	O1	O	0.25000	0.04765	0.83524
813	O1	O	0.25000	0.29765	0.58524
814	O1	O	0.00000	0.04765	0.91476
815	O1	O	0.00000	0.29765	0.66476
816	O1	O	0.25000	0.04765	0.66476
817	O1	O	0.25000	0.29765	0.91476
818	O1	O	0.00000	0.45235	0.91476
819	O1	O	0.00000	0.20235	0.66476
820	O1	O	0.25000	0.45235	0.66476
821	O1	O	0.25000	0.20235	0.91476
822	O1	O	0.00000	0.45235	0.58524
823	O1	O	0.00000	0.20235	0.83524
824	O1	O	0.25000	0.45235	0.83524
825	O1	O	0.25000	0.20235	0.58524
826	O1	O	0.00000	0.08524	0.95235
827	O1	O	0.00000	0.33524	0.70235
828	O1	O	0.25000	0.08524	0.70235
829	O1	O	0.25000	0.33524	0.95235
830	O1	O	0.00000	0.41476	0.95235
831	O1	O	0.00000	0.16476	0.70235
832	O1	O	0.25000	0.41476	0.70235
833	O1	O	0.25000	0.16476	0.95235
834	O1	O	0.00000	0.41476	0.54765
835	O1	O	0.00000	0.16476	0.79765
836	O1	O	0.25000	0.41476	0.79765
837	O1	O	0.25000	0.16476	0.54765
838	O1	O	0.00000	0.08524	0.54765
839	O1	O	0.00000	0.33524	0.79765
840	O1	O	0.25000	0.08524	0.79765
841	O1	O	0.25000	0.33524	0.54765
842	O1	O	0.08524	0.04765	0.50000
843	O1	O	0.08524	0.29765	0.75000
844	O1	O	0.33524	0.04765	0.75000
845	O1	O	0.33524	0.29765	0.50000
846	O1	O	0.41476	0.04765	0.50000
847	O1	O	0.41476	0.29765	0.75000
848	O1	O	0.16476	0.04765	0.75000
849	O1	O	0.16476	0.29765	0.50000
850	O1	O	0.41476	0.45235	0.50000
851	O1	O	0.41476	0.20235	0.75000
852	O1	O	0.16476	0.45235	0.75000
853	O1	O	0.16476	0.20235	0.50000
854	O1	O	0.08524	0.45235	0.50000
855	O1	O	0.08524	0.20235	0.75000
856	O1	O	0.33524	0.45235	0.75000
857	O1	O	0.33524	0.20235	0.50000
858	O1	O	0.04765	0.00000	0.91476
859	O1	O	0.04765	0.25000	0.66476
860	O1	O	0.29765	0.00000	0.66476
861	O1	O	0.29765	0.25000	0.91476
862	O1	O	0.04765	0.00000	0.58524
863	O1	O	0.04765	0.25000	0.83524
864	O1	O	0.29765	0.00000	0.83524

865	O1	O	0.29765	0.25000	0.58524
866	O1	O	0.45235	0.00000	0.58524
867	O1	O	0.45235	0.25000	0.83524
868	O1	O	0.20235	0.00000	0.83524
869	O1	O	0.20235	0.25000	0.58524
870	O1	O	0.45235	0.00000	0.91476
871	O1	O	0.45235	0.25000	0.66476
872	O1	O	0.20235	0.00000	0.66476
873	O1	O	0.20235	0.25000	0.91476
874	O2	O	0.02805	0.47195	0.97195
875	O2	O	0.02805	0.22195	0.72195
876	O2	O	0.27805	0.47195	0.72195
877	O2	O	0.27805	0.22195	0.97195
878	O2	O	0.47195	0.02805	0.97195
879	O2	O	0.47195	0.27805	0.72195
880	O2	O	0.22195	0.02805	0.72195
881	O2	O	0.22195	0.27805	0.97195
882	O2	O	0.47195	0.47195	0.52805
883	O2	O	0.47195	0.22195	0.77805
884	O2	O	0.22195	0.47195	0.77805
885	O2	O	0.22195	0.22195	0.52805
886	O2	O	0.02805	0.02805	0.52805
887	O2	O	0.02805	0.27805	0.77805
888	O2	O	0.27805	0.02805	0.77805
889	O2	O	0.27805	0.27805	0.52805
890	O1	O	0.47195	0.02805	0.52805
891	O1	O	0.47195	0.27805	0.77805
892	O1	O	0.22195	0.02805	0.77805
893	O1	O	0.22195	0.27805	0.52805
894	O1	O	0.02805	0.47195	0.52805
895	O1	O	0.02805	0.22195	0.77805
896	O1	O	0.27805	0.47195	0.77805
897	O1	O	0.27805	0.22195	0.52805
898	O1	O	0.47195	0.47195	0.97195
899	O1	O	0.47195	0.22195	0.72195
900	O1	O	0.22195	0.47195	0.72195
901	O1	O	0.22195	0.22195	0.97195
902	O1	O	0.02805	0.02805	0.97195
903	O1	O	0.02805	0.27805	0.72195
904	O1	O	0.27805	0.02805	0.72195
905	O1	O	0.27805	0.27805	0.97195
906	Zr1	Zr	0.05994	0.00000	0.50000
907	Zr1	Zr	0.05994	0.25000	0.75000
908	Zr1	Zr	0.30994	0.00000	0.75000
909	Zr1	Zr	0.30994	0.25000	0.50000
910	Zr1	Zr	0.44006	0.00000	0.50000
911	Zr1	Zr	0.44006	0.25000	0.75000
912	Zr1	Zr	0.19006	0.00000	0.75000
913	Zr1	Zr	0.19006	0.25000	0.50000
914	Zr1	Zr	0.00000	0.05994	0.50000
915	Zr1	Zr	0.00000	0.30994	0.75000
916	Zr1	Zr	0.25000	0.05994	0.75000
917	Zr1	Zr	0.25000	0.30994	0.50000
918	Zr1	Zr	0.00000	0.44006	0.50000

919	Zr1	Zr	0.00000	0.19006	0.75000
920	Zr1	Zr	0.25000	0.44006	0.75000
921	Zr1	Zr	0.25000	0.19006	0.50000
922	Zr1	Zr	0.00000	0.00000	0.55994
923	Zr1	Zr	0.00000	0.25000	0.80994
924	Zr1	Zr	0.25000	0.00000	0.80994
925	Zr1	Zr	0.25000	0.25000	0.55994
926	Zr1	Zr	0.00000	0.00000	0.94006
927	Zr1	Zr	0.00000	0.25000	0.69006
928	Zr1	Zr	0.25000	0.00000	0.69006
929	Zr1	Zr	0.25000	0.25000	0.94006
930	C2	C	0.13375	0.50000	0.09220
931	C2	C	0.13375	0.75000	0.34220
932	C2	C	0.38375	0.50000	0.34220
933	C2	C	0.38375	0.75000	0.09220
934	C2	C	0.36625	0.50000	0.09220
935	C2	C	0.36625	0.75000	0.34220
936	C2	C	0.11625	0.50000	0.34220
937	C2	C	0.11625	0.75000	0.09220
938	C2	C	0.36625	0.50000	0.40780
939	C2	C	0.36625	0.75000	0.15780
940	C2	C	0.11625	0.50000	0.15780
941	C2	C	0.11625	0.75000	0.40780
942	C2	C	0.13375	0.50000	0.40780
943	C2	C	0.13375	0.75000	0.15780
944	C2	C	0.38375	0.50000	0.15780
945	C2	C	0.38375	0.75000	0.40780
946	C2	C	0.09220	0.63375	0.00000
947	C2	C	0.09220	0.88375	0.25000
948	C2	C	0.34220	0.63375	0.25000
949	C2	C	0.34220	0.88375	0.00000
950	C2	C	0.09220	0.86625	0.00000
951	C2	C	0.09220	0.61625	0.25000
952	C2	C	0.34220	0.86625	0.25000
953	C2	C	0.34220	0.61625	0.00000
954	C2	C	0.40780	0.86625	0.00000
955	C2	C	0.40780	0.61625	0.25000
956	C2	C	0.15780	0.86625	0.25000
957	C2	C	0.15780	0.61625	0.00000
958	C2	C	0.40780	0.63375	0.00000
959	C2	C	0.40780	0.88375	0.25000
960	C2	C	0.15780	0.63375	0.25000
961	C2	C	0.15780	0.88375	0.00000
962	C2	C	0.00000	0.59220	0.13375
963	C2	C	0.00000	0.84220	0.38375
964	C2	C	0.25000	0.59220	0.38375
965	C2	C	0.25000	0.84220	0.13375
966	C2	C	0.00000	0.59220	0.36625
967	C2	C	0.00000	0.84220	0.11625
968	C2	C	0.25000	0.59220	0.11625
969	C2	C	0.25000	0.84220	0.36625
970	C2	C	0.00000	0.90780	0.36625
971	C2	C	0.00000	0.65780	0.11625
972	C2	C	0.25000	0.90780	0.11625

973	C2	C	0.25000	0.65780	0.36625
974	C2	C	0.00000	0.90780	0.13375
975	C2	C	0.00000	0.65780	0.38375
976	C2	C	0.25000	0.90780	0.38375
977	C2	C	0.25000	0.65780	0.13375
978	C2	C	0.00000	0.63375	0.40780
979	C2	C	0.00000	0.88375	0.15780
980	C2	C	0.25000	0.63375	0.15780
981	C2	C	0.25000	0.88375	0.40780
982	C2	C	0.00000	0.86625	0.40780
983	C2	C	0.00000	0.61625	0.15780
984	C2	C	0.25000	0.86625	0.15780
985	C2	C	0.25000	0.61625	0.40780
986	C2	C	0.00000	0.86625	0.09220
987	C2	C	0.00000	0.61625	0.34220
988	C2	C	0.25000	0.86625	0.34220
989	C2	C	0.25000	0.61625	0.09220
990	C2	C	0.00000	0.63375	0.09220
991	C2	C	0.00000	0.88375	0.34220
992	C2	C	0.25000	0.63375	0.34220
993	C2	C	0.25000	0.88375	0.09220
994	C2	C	0.13375	0.59220	0.00000
995	C2	C	0.13375	0.84220	0.25000
996	C2	C	0.38375	0.59220	0.25000
997	C2	C	0.38375	0.84220	0.00000
998	C2	C	0.36625	0.59220	0.00000
999	C2	C	0.36625	0.84220	0.25000
1000	C2	C	0.11625	0.59220	0.25000
1001	C2	C	0.11625	0.84220	0.00000
1002	C2	C	0.36625	0.90780	0.00000
1003	C2	C	0.36625	0.65780	0.25000
1004	C2	C	0.11625	0.90780	0.25000
1005	C2	C	0.11625	0.65780	0.00000
1006	C2	C	0.13375	0.90780	0.00000
1007	C2	C	0.13375	0.65780	0.25000
1008	C2	C	0.38375	0.90780	0.25000
1009	C2	C	0.38375	0.65780	0.00000
1010	C2	C	0.09220	0.50000	0.36625
1011	C2	C	0.09220	0.75000	0.11625
1012	C2	C	0.34220	0.50000	0.11625
1013	C2	C	0.34220	0.75000	0.36625
1014	C2	C	0.09220	0.50000	0.13375
1015	C2	C	0.09220	0.75000	0.38375
1016	C2	C	0.34220	0.50000	0.38375
1017	C2	C	0.34220	0.75000	0.13375
1018	C2	C	0.40780	0.50000	0.13375
1019	C2	C	0.40780	0.75000	0.38375
1020	C2	C	0.15780	0.50000	0.38375
1021	C2	C	0.15780	0.75000	0.13375
1022	C2	C	0.40780	0.50000	0.36625
1023	C2	C	0.40780	0.75000	0.11625
1024	C2	C	0.15780	0.50000	0.11625
1025	C2	C	0.15780	0.75000	0.36625
1026	C1	C	0.07680	0.50000	0.42320

1027	C1	C	0.07680	0.75000	0.17320
1028	C1	C	0.32680	0.50000	0.17320
1029	C1	C	0.32680	0.75000	0.42320
1030	C1	C	0.42320	0.50000	0.42320
1031	C1	C	0.42320	0.75000	0.17320
1032	C1	C	0.17320	0.50000	0.17320
1033	C1	C	0.17320	0.75000	0.42320
1034	C1	C	0.42320	0.50000	0.07680
1035	C1	C	0.42320	0.75000	0.32680
1036	C1	C	0.17320	0.50000	0.32680
1037	C1	C	0.17320	0.75000	0.07680
1038	C1	C	0.07680	0.50000	0.07680
1039	C1	C	0.07680	0.75000	0.32680
1040	C1	C	0.32680	0.50000	0.32680
1041	C1	C	0.32680	0.75000	0.07680
1042	C1	C	0.42320	0.57680	0.00000
1043	C1	C	0.42320	0.82680	0.25000
1044	C1	C	0.17320	0.57680	0.25000
1045	C1	C	0.17320	0.82680	0.00000
1046	C1	C	0.42320	0.92320	0.00000
1047	C1	C	0.42320	0.67320	0.25000
1048	C1	C	0.17320	0.92320	0.25000
1049	C1	C	0.17320	0.67320	0.00000
1050	C1	C	0.07680	0.92320	0.00000
1051	C1	C	0.07680	0.67320	0.25000
1052	C1	C	0.32680	0.92320	0.25000
1053	C1	C	0.32680	0.67320	0.00000
1054	C1	C	0.07680	0.57680	0.00000
1055	C1	C	0.07680	0.82680	0.25000
1056	C1	C	0.32680	0.57680	0.25000
1057	C1	C	0.32680	0.82680	0.00000
1058	C1	C	0.00000	0.92320	0.07680
1059	C1	C	0.00000	0.67320	0.32680
1060	C1	C	0.25000	0.92320	0.32680
1061	C1	C	0.25000	0.67320	0.07680
1062	C1	C	0.00000	0.92320	0.42320
1063	C1	C	0.00000	0.67320	0.17320
1064	C1	C	0.25000	0.92320	0.17320
1065	C1	C	0.25000	0.67320	0.42320
1066	C1	C	0.00000	0.57680	0.42320
1067	C1	C	0.00000	0.82680	0.17320
1068	C1	C	0.25000	0.57680	0.17320
1069	C1	C	0.25000	0.82680	0.42320
1070	C1	C	0.00000	0.57680	0.07680
1071	C1	C	0.00000	0.82680	0.32680
1072	C1	C	0.25000	0.57680	0.32680
1073	C1	C	0.25000	0.82680	0.07680
1074	C3	C	0.10250	0.50000	0.10250
1075	C3	C	0.10250	0.75000	0.35250
1076	C3	C	0.35250	0.50000	0.35250
1077	C3	C	0.35250	0.75000	0.10250
1078	C3	C	0.39750	0.50000	0.10250
1079	C3	C	0.39750	0.75000	0.35250
1080	C3	C	0.14750	0.50000	0.35250

1081	C3	C	0.14750	0.75000	0.10250
1082	C3	C	0.39750	0.50000	0.39750
1083	C3	C	0.39750	0.75000	0.14750
1084	C3	C	0.14750	0.50000	0.14750
1085	C3	C	0.14750	0.75000	0.39750
1086	C3	C	0.10250	0.50000	0.39750
1087	C3	C	0.10250	0.75000	0.14750
1088	C3	C	0.35250	0.50000	0.14750
1089	C3	C	0.35250	0.75000	0.39750
1090	C3	C	0.10250	0.60250	0.00000
1091	C3	C	0.10250	0.85250	0.25000
1092	C3	C	0.35250	0.60250	0.25000
1093	C3	C	0.35250	0.85250	0.00000
1094	C3	C	0.10250	0.89750	0.00000
1095	C3	C	0.10250	0.64750	0.25000
1096	C3	C	0.35250	0.89750	0.25000
1097	C3	C	0.35250	0.64750	0.00000
1098	C3	C	0.39750	0.89750	0.00000
1099	C3	C	0.39750	0.64750	0.25000
1100	C3	C	0.14750	0.89750	0.25000
1101	C3	C	0.14750	0.64750	0.00000
1102	C3	C	0.39750	0.60250	0.00000
1103	C3	C	0.39750	0.85250	0.25000
1104	C3	C	0.14750	0.60250	0.25000
1105	C3	C	0.14750	0.85250	0.00000
1106	C3	C	0.00000	0.60250	0.10250
1107	C3	C	0.00000	0.85250	0.35250
1108	C3	C	0.25000	0.60250	0.35250
1109	C3	C	0.25000	0.85250	0.10250
1110	C3	C	0.00000	0.60250	0.39750
1111	C3	C	0.00000	0.85250	0.14750
1112	C3	C	0.25000	0.60250	0.14750
1113	C3	C	0.25000	0.85250	0.39750
1114	C3	C	0.00000	0.89750	0.39750
1115	C3	C	0.00000	0.64750	0.14750
1116	C3	C	0.25000	0.89750	0.14750
1117	C3	C	0.25000	0.64750	0.39750
1118	C3	C	0.00000	0.89750	0.10250
1119	C3	C	0.00000	0.64750	0.35250
1120	C3	C	0.25000	0.89750	0.35250
1121	C3	C	0.25000	0.64750	0.10250
1122	H1	H	0.13926	0.99997	0.06655
1123	H1	H	0.13926	0.74997	0.31655
1124	H1	H	0.38926	0.99997	0.31655
1125	H1	H	0.38926	0.74997	0.06655
1126	H1	H	0.36074	0.50003	0.06655
1127	H1	H	0.36074	0.75004	0.31655
1128	H1	H	0.11074	0.50003	0.31655
1129	H1	H	0.11074	0.75004	0.06655
1130	H1	H	0.36074	0.99996	0.43345
1131	H1	H	0.36074	0.74997	0.18345
1132	H1	H	0.11074	0.99997	0.18345
1133	H1	H	0.11074	0.74997	0.43346
1134	H1	H	0.13926	0.50003	0.43345

1135	H1	H	0.13926	0.75003	0.18346
1136	H1	H	0.38926	0.50003	0.18345
1137	H1	H	0.38926	0.75004	0.43346
1138	H1	H	0.06655	0.63926	0.49996
1139	H1	H	0.06655	0.88926	0.24997
1140	H1	H	0.31655	0.63926	0.24996
1141	H1	H	0.31654	0.88926	0.49997
1142	H1	H	0.06655	0.86074	0.00003
1143	H1	H	0.06655	0.61074	0.25004
1144	H1	H	0.31655	0.86074	0.25003
1145	H1	H	0.31655	0.61074	0.00004
1146	H1	H	0.43345	0.86074	0.49996
1147	H1	H	0.43345	0.61074	0.24997
1148	H1	H	0.18345	0.86074	0.24996
1149	H1	H	0.18345	0.61074	0.49997
1150	H1	H	0.43345	0.63926	0.00003
1151	H1	H	0.43345	0.88926	0.25004
1152	H1	H	0.18345	0.63926	0.25003
1153	H1	H	0.18345	0.88926	0.00004
1154	H1	H	0.49997	0.56655	0.13926
1155	H1	H	0.49996	0.81655	0.38926
1156	H1	H	0.24997	0.56655	0.38926
1157	H1	H	0.24996	0.81655	0.13926
1158	H1	H	0.00003	0.56655	0.36074
1159	H1	H	0.00003	0.81655	0.11074
1160	H1	H	0.25003	0.56655	0.11074
1161	H1	H	0.25004	0.81655	0.36074
1162	H1	H	0.49997	0.93345	0.36074
1163	H1	H	0.49997	0.68345	0.11074
1164	H1	H	0.24997	0.93345	0.11074
1165	H1	H	0.24997	0.68345	0.36074
1166	H1	H	0.00003	0.93345	0.13926
1167	H1	H	0.00003	0.68345	0.38926
1168	H1	H	0.25004	0.93345	0.38926
1169	H1	H	0.25003	0.68346	0.13926
1170	H1	H	0.00003	0.63926	0.43345
1171	H1	H	0.00003	0.88926	0.18345
1172	H1	H	0.25003	0.63926	0.18345
1173	H1	H	0.25004	0.88926	0.43345
1174	H1	H	0.49996	0.86074	0.43345
1175	H1	H	0.49997	0.61074	0.18345
1176	H1	H	0.24997	0.86074	0.18345
1177	H1	H	0.24997	0.61074	0.43345
1178	H1	H	0.00003	0.86074	0.06655
1179	H1	H	0.00003	0.61074	0.31655
1180	H1	H	0.25004	0.86074	0.31655
1181	H1	H	0.25003	0.61074	0.06654
1182	H1	H	0.49997	0.63926	0.06655
1183	H1	H	0.49997	0.88926	0.31655
1184	H1	H	0.24997	0.63926	0.31655
1185	H1	H	0.24997	0.88926	0.06654
1186	H1	H	0.13926	0.56655	0.49997
1187	H1	H	0.13926	0.81655	0.24996
1188	H1	H	0.38926	0.56655	0.24997

1189	H1	H	0.38926	0.81655	0.49996
1190	H1	H	0.36074	0.56655	0.00003
1191	H1	H	0.36074	0.81655	0.25003
1192	H1	H	0.11074	0.56655	0.25003
1193	H1	H	0.11074	0.81655	0.00003
1194	H1	H	0.36074	0.93345	0.49997
1195	H1	H	0.36074	0.68345	0.24997
1196	H1	H	0.11074	0.93345	0.24997
1197	H1	H	0.11074	0.68345	0.49996
1198	H1	H	0.13926	0.93345	0.00004
1199	H1	H	0.13926	0.68346	0.25003
1200	H1	H	0.38926	0.93345	0.25004
1201	H1	H	0.38926	0.68346	0.00003
1202	H1	H	0.06655	0.50003	0.36074
1203	H1	H	0.06655	0.75004	0.11074
1204	H1	H	0.31655	0.50003	0.11074
1205	H1	H	0.31655	0.75004	0.36074
1206	H1	H	0.06655	0.99996	0.13926
1207	H1	H	0.06655	0.74997	0.38926
1208	H1	H	0.31655	0.99996	0.38926
1209	H1	H	0.31655	0.74997	0.13926
1210	H1	H	0.43345	0.50003	0.13926
1211	H1	H	0.43345	0.75004	0.38926
1212	H1	H	0.18345	0.50003	0.38926
1213	H1	H	0.18345	0.75003	0.13926
1214	H1	H	0.43345	0.99996	0.36074
1215	H1	H	0.43345	0.74997	0.11074
1216	H1	H	0.18345	0.99996	0.11074
1217	H1	H	0.18345	0.74997	0.36074
1218	H2	H	0.20825	0.79176	0.45824
1219	H2	H	0.29174	0.70825	0.45823
1220	H2	H	0.20827	0.70823	0.04177
1221	H2	H	0.29179	0.79168	0.04178
1222	H2	H	0.45823	0.79173	0.20823
1223	H2	H	0.45824	0.70825	0.29176
1224	H2	H	0.04174	0.79176	0.29177
1225	H2	H	0.04173	0.70823	0.20823
1226	H2	H	0.29175	0.95824	0.20824
1227	H2	H	0.20824	0.95824	0.29176
1228	H2	H	0.20823	0.54172	0.20822
1229	H2	H	0.29177	0.54173	0.29178
1230	H2	H	0.04178	0.54177	0.04173
1231	H2	H	0.45823	0.95824	0.04174
1232	H2	H	0.45823	0.54176	0.45825
1233	H2	H	0.04176	0.95824	0.45824
1234	O1	O	0.08524	0.50000	0.04765
1235	O1	O	0.08524	0.75000	0.29765
1236	O1	O	0.33524	0.50000	0.29765
1237	O1	O	0.33524	0.75000	0.04765
1238	O1	O	0.41476	0.50000	0.04765
1239	O1	O	0.41476	0.75000	0.29765
1240	O1	O	0.16476	0.50000	0.29765
1241	O1	O	0.16476	0.75000	0.04765
1242	O1	O	0.41476	0.50000	0.45235

1243	O1	O	0.41476	0.75000	0.20235
1244	O1	O	0.16476	0.50000	0.20235
1245	O1	O	0.16476	0.75000	0.45235
1246	O1	O	0.08524	0.50000	0.45235
1247	O1	O	0.08524	0.75000	0.20235
1248	O1	O	0.33524	0.50000	0.20235
1249	O1	O	0.33524	0.75000	0.45235
1250	O1	O	0.04765	0.58524	0.00000
1251	O1	O	0.04765	0.83524	0.25000
1252	O1	O	0.29765	0.58524	0.25000
1253	O1	O	0.29765	0.83524	0.00000
1254	O1	O	0.04765	0.91476	0.00000
1255	O1	O	0.04765	0.66476	0.25000
1256	O1	O	0.29765	0.91476	0.25000
1257	O1	O	0.29765	0.66476	0.00000
1258	O1	O	0.45235	0.91476	0.00000
1259	O1	O	0.45235	0.66476	0.25000
1260	O1	O	0.20235	0.91476	0.25000
1261	O1	O	0.20235	0.66476	0.00000
1262	O1	O	0.45235	0.58524	0.00000
1263	O1	O	0.45235	0.83524	0.25000
1264	O1	O	0.20235	0.58524	0.25000
1265	O1	O	0.20235	0.83524	0.00000
1266	O1	O	0.00000	0.54765	0.08524
1267	O1	O	0.00000	0.79765	0.33524
1268	O1	O	0.25000	0.54765	0.33524
1269	O1	O	0.25000	0.79765	0.08524
1270	O1	O	0.00000	0.54765	0.41476
1271	O1	O	0.00000	0.79765	0.16476
1272	O1	O	0.25000	0.54765	0.16476
1273	O1	O	0.25000	0.79765	0.41476
1274	O1	O	0.00000	0.95235	0.41476
1275	O1	O	0.00000	0.70235	0.16476
1276	O1	O	0.25000	0.95235	0.16476
1277	O1	O	0.25000	0.70235	0.41476
1278	O1	O	0.00000	0.95235	0.08524
1279	O1	O	0.00000	0.70235	0.33524
1280	O1	O	0.25000	0.95235	0.33524
1281	O1	O	0.25000	0.70235	0.08524
1282	O1	O	0.00000	0.58524	0.45235
1283	O1	O	0.00000	0.83524	0.20235
1284	O1	O	0.25000	0.58524	0.20235
1285	O1	O	0.25000	0.83524	0.45235
1286	O1	O	0.00000	0.91476	0.45235
1287	O1	O	0.00000	0.66476	0.20235
1288	O1	O	0.25000	0.91476	0.20235
1289	O1	O	0.25000	0.66476	0.45235
1290	O1	O	0.00000	0.91476	0.04765
1291	O1	O	0.00000	0.66476	0.29765
1292	O1	O	0.25000	0.91476	0.29765
1293	O1	O	0.25000	0.66476	0.04765
1294	O1	O	0.00000	0.58524	0.04765
1295	O1	O	0.00000	0.83524	0.29765
1296	O1	O	0.25000	0.58524	0.29765

1297	O1	O	0.25000	0.83524	0.04765
1298	O1	O	0.08524	0.54765	0.00000
1299	O1	O	0.08524	0.79765	0.25000
1300	O1	O	0.33524	0.54765	0.25000
1301	O1	O	0.33524	0.79765	0.00000
1302	O1	O	0.41476	0.54765	0.00000
1303	O1	O	0.41476	0.79765	0.25000
1304	O1	O	0.16476	0.54765	0.25000
1305	O1	O	0.16476	0.79765	0.00000
1306	O1	O	0.41476	0.95235	0.00000
1307	O1	O	0.41476	0.70235	0.25000
1308	O1	O	0.16476	0.95235	0.25000
1309	O1	O	0.16476	0.70235	0.00000
1310	O1	O	0.08524	0.95235	0.00000
1311	O1	O	0.08524	0.70235	0.25000
1312	O1	O	0.33524	0.95235	0.25000
1313	O1	O	0.33524	0.70235	0.00000
1314	O1	O	0.04765	0.50000	0.41476
1315	O1	O	0.04765	0.75000	0.16476
1316	O1	O	0.29765	0.50000	0.16476
1317	O1	O	0.29765	0.75000	0.41476
1318	O1	O	0.04765	0.50000	0.08524
1319	O1	O	0.04765	0.75000	0.33524
1320	O1	O	0.29765	0.50000	0.33524
1321	O1	O	0.29765	0.75000	0.08524
1322	O1	O	0.45235	0.50000	0.08524
1323	O1	O	0.45235	0.75000	0.33524
1324	O1	O	0.20235	0.50000	0.33524
1325	O1	O	0.20235	0.75000	0.08524
1326	O1	O	0.45235	0.50000	0.41476
1327	O1	O	0.45235	0.75000	0.16476
1328	O1	O	0.20235	0.50000	0.16476
1329	O1	O	0.20235	0.75000	0.41476
1330	O2	O	0.02805	0.97195	0.47195
1331	O2	O	0.02805	0.72195	0.22195
1332	O2	O	0.27805	0.97195	0.22195
1333	O2	O	0.27805	0.72195	0.47195
1334	O2	O	0.47195	0.52805	0.47195
1335	O2	O	0.47195	0.77805	0.22195
1336	O2	O	0.22195	0.52805	0.22195
1337	O2	O	0.22195	0.77805	0.47195
1338	O2	O	0.47195	0.97195	0.02805
1339	O2	O	0.47195	0.72195	0.27805
1340	O2	O	0.22195	0.97195	0.27805
1341	O2	O	0.22195	0.72195	0.02805
1342	O2	O	0.02805	0.52805	0.02805
1343	O2	O	0.02805	0.77805	0.27805
1344	O2	O	0.27805	0.52805	0.27805
1345	O2	O	0.27805	0.77805	0.02805
1346	O1	O	0.47195	0.52805	0.02805
1347	O1	O	0.47195	0.77805	0.27805
1348	O1	O	0.22195	0.52805	0.27805
1349	O1	O	0.22195	0.77805	0.02805
1350	O1	O	0.02805	0.97195	0.02805

1351	O1	O	0.02805	0.72195	0.27805
1352	O1	O	0.27805	0.97195	0.27805
1353	O1	O	0.27805	0.72195	0.02805
1354	O1	O	0.47195	0.97195	0.47195
1355	O1	O	0.47195	0.72195	0.22195
1356	O1	O	0.22195	0.97195	0.22195
1357	O1	O	0.22195	0.72195	0.47195
1358	O1	O	0.02805	0.52805	0.47195
1359	O1	O	0.02805	0.77805	0.22195
1360	O1	O	0.27805	0.52805	0.22195
1361	O1	O	0.27805	0.77805	0.47195
1362	Zr1	Zr	0.05994	0.50000	0.00000
1363	Zr1	Zr	0.05994	0.75000	0.25000
1364	Zr1	Zr	0.30994	0.50000	0.25000
1365	Zr1	Zr	0.30994	0.75000	0.00000
1366	Zr1	Zr	0.44006	0.50000	0.00000
1367	Zr1	Zr	0.44006	0.75000	0.25000
1368	Zr1	Zr	0.19006	0.50000	0.25000
1369	Zr1	Zr	0.19006	0.75000	0.00000
1370	Zr1	Zr	0.00000	0.55994	0.00000
1371	Zr1	Zr	0.00000	0.80994	0.25000
1372	Zr1	Zr	0.25000	0.55994	0.25000
1373	Zr1	Zr	0.25000	0.80994	0.00000
1374	Zr1	Zr	0.00000	0.94006	0.00000
1375	Zr1	Zr	0.00000	0.69006	0.25000
1376	Zr1	Zr	0.25000	0.94006	0.25000
1377	Zr1	Zr	0.25000	0.69006	0.00000
1378	Zr1	Zr	0.00000	0.50000	0.05994
1379	Zr1	Zr	0.00000	0.75000	0.30994
1380	Zr1	Zr	0.25000	0.50000	0.30994
1381	Zr1	Zr	0.25000	0.75000	0.05994
1382	Zr1	Zr	0.00000	0.50000	0.44006
1383	Zr1	Zr	0.00000	0.75000	0.19006
1384	Zr1	Zr	0.25000	0.50000	0.19006
1385	Zr1	Zr	0.25000	0.75000	0.44006
1386	C2	C	0.13375	0.50000	0.59220
1387	C2	C	0.13375	0.75000	0.84220
1388	C2	C	0.38375	0.50000	0.84220
1389	C2	C	0.38375	0.75000	0.59220
1390	C2	C	0.36625	0.50000	0.59220
1391	C2	C	0.36625	0.75000	0.84220
1392	C2	C	0.11625	0.50000	0.84220
1393	C2	C	0.11625	0.75000	0.59220
1394	C2	C	0.36625	0.50000	0.90780
1395	C2	C	0.36625	0.75000	0.65780
1396	C2	C	0.11625	0.50000	0.65780
1397	C2	C	0.11625	0.75000	0.90780
1398	C2	C	0.13375	0.50000	0.90780
1399	C2	C	0.13375	0.75000	0.65780
1400	C2	C	0.38375	0.50000	0.65780
1401	C2	C	0.38375	0.75000	0.90780
1402	C2	C	0.09220	0.63375	0.50000
1403	C2	C	0.09220	0.88375	0.75000
1404	C2	C	0.34220	0.63375	0.75000

1405	C2	C	0.34220	0.88375	0.50000
1406	C2	C	0.09220	0.86625	0.50000
1407	C2	C	0.09220	0.61625	0.75000
1408	C2	C	0.34220	0.86625	0.75000
1409	C2	C	0.40780	0.86625	0.50000
1410	C2	C	0.40780	0.61625	0.75000
1411	C2	C	0.15780	0.86625	0.75000
1412	C2	C	0.15780	0.61625	0.50000
1413	C2	C	0.40780	0.88375	0.75000
1414	C2	C	0.15780	0.63375	0.75000
1415	C2	C	0.15780	0.88375	0.50000
1416	C2	C	0.00000	0.59220	0.63375
1417	C2	C	0.00000	0.84220	0.88375
1418	C2	C	0.25000	0.59220	0.88375
1419	C2	C	0.25000	0.84220	0.63375
1420	C2	C	0.00000	0.59220	0.86625
1421	C2	C	0.00000	0.84220	0.61625
1422	C2	C	0.25000	0.59220	0.61625
1423	C2	C	0.25000	0.84220	0.86625
1424	C2	C	0.00000	0.90780	0.86625
1425	C2	C	0.00000	0.65780	0.61625
1426	C2	C	0.25000	0.90780	0.61625
1427	C2	C	0.25000	0.65780	0.86625
1428	C2	C	0.00000	0.90780	0.63375
1429	C2	C	0.00000	0.65780	0.88375
1430	C2	C	0.25000	0.90780	0.88375
1431	C2	C	0.25000	0.65780	0.63375
1432	C2	C	0.00000	0.63375	0.90780
1433	C2	C	0.00000	0.88375	0.65780
1434	C2	C	0.25000	0.63375	0.65780
1435	C2	C	0.25000	0.88375	0.90780
1436	C2	C	0.00000	0.86625	0.90780
1437	C2	C	0.00000	0.61625	0.65780
1438	C2	C	0.25000	0.86625	0.65780
1439	C2	C	0.25000	0.61625	0.90780
1440	C2	C	0.00000	0.86625	0.59220
1441	C2	C	0.00000	0.61625	0.84220
1442	C2	C	0.25000	0.86625	0.84220
1443	C2	C	0.25000	0.61625	0.59220
1444	C2	C	0.00000	0.63375	0.59220
1445	C2	C	0.00000	0.88375	0.84220
1446	C2	C	0.25000	0.63375	0.84220
1447	C2	C	0.25000	0.88375	0.59220
1448	C2	C	0.13375	0.59220	0.50000
1449	C2	C	0.13375	0.84220	0.75000
1450	C2	C	0.38375	0.59220	0.75000
1451	C2	C	0.38375	0.84220	0.50000
1452	C2	C	0.36625	0.84220	0.75000
1453	C2	C	0.11625	0.59220	0.75000
1454	C2	C	0.11625	0.84220	0.50000
1455	C2	C	0.36625	0.90780	0.50000
1456	C2	C	0.36625	0.65780	0.75000
1457	C2	C	0.11625	0.90780	0.75000
1458	C2	C	0.11625	0.65780	0.50000

1459	C2	C	0.13375	0.90780	0.50000
1460	C2	C	0.13375	0.65780	0.75000
1461	C2	C	0.38375	0.90780	0.75000
1462	C2	C	0.09220	0.50000	0.86625
1463	C2	C	0.09220	0.75000	0.61625
1464	C2	C	0.34220	0.50000	0.61625
1465	C2	C	0.34220	0.75000	0.86625
1466	C2	C	0.09220	0.50000	0.63375
1467	C2	C	0.09220	0.75000	0.88375
1468	C2	C	0.34220	0.50000	0.88375
1469	C2	C	0.34220	0.75000	0.63375
1470	C2	C	0.40780	0.50000	0.63375
1471	C2	C	0.40780	0.75000	0.88375
1472	C2	C	0.15780	0.50000	0.88375
1473	C2	C	0.15780	0.75000	0.63375
1474	C2	C	0.40780	0.50000	0.86625
1475	C2	C	0.40780	0.75000	0.61625
1476	C2	C	0.15780	0.50000	0.61625
1477	C2	C	0.15780	0.75000	0.86625
1478	C1	C	0.07680	0.50000	0.92320
1479	C1	C	0.07680	0.75000	0.67320
1480	C1	C	0.32680	0.50000	0.67320
1481	C1	C	0.32680	0.75000	0.92320
1482	C1	C	0.42320	0.50000	0.92320
1483	C1	C	0.42320	0.75000	0.67320
1484	C1	C	0.17320	0.50000	0.67320
1485	C1	C	0.17320	0.75000	0.92320
1486	C1	C	0.42320	0.50000	0.57680
1487	C1	C	0.42320	0.75000	0.82680
1488	C1	C	0.17320	0.50000	0.82680
1489	C1	C	0.17320	0.75000	0.57680
1490	C1	C	0.07680	0.50000	0.57680
1491	C1	C	0.07680	0.75000	0.82680
1492	C1	C	0.32680	0.50000	0.82680
1493	C1	C	0.32680	0.75000	0.57680
1494	C1	C	0.42320	0.82680	0.75000
1495	C1	C	0.17320	0.57680	0.75000
1496	C1	C	0.17320	0.82680	0.50000
1497	C1	C	0.42320	0.92320	0.50000
1498	C1	C	0.42320	0.67320	0.75000
1499	C1	C	0.17320	0.92320	0.75000
1500	C1	C	0.17320	0.67320	0.50000
1501	C1	C	0.07680	0.92320	0.50000
1502	C1	C	0.07680	0.67320	0.75000
1503	C1	C	0.32680	0.92320	0.75000
1504	C1	C	0.07680	0.57680	0.50000
1505	C1	C	0.07680	0.82680	0.75000
1506	C1	C	0.32680	0.57680	0.75000
1507	C1	C	0.32680	0.82680	0.50000
1508	C1	C	0.00000	0.92320	0.57680
1509	C1	C	0.00000	0.67320	0.82680
1510	C1	C	0.25000	0.92320	0.82680
1511	C1	C	0.25000	0.67320	0.57680
1512	C1	C	0.00000	0.92320	0.92320

1513	C1	C	0.00000	0.67320	0.67320
1514	C1	C	0.25000	0.92320	0.67320
1515	C1	C	0.25000	0.67320	0.92320
1516	C1	C	0.00000	0.57680	0.92320
1517	C1	C	0.00000	0.82680	0.67320
1518	C1	C	0.25000	0.57680	0.67320
1519	C1	C	0.25000	0.82680	0.92320
1520	C1	C	0.00000	0.57680	0.57680
1521	C1	C	0.00000	0.82680	0.82680
1522	C1	C	0.25000	0.57680	0.82680
1523	C1	C	0.25000	0.82680	0.57680
1524	C3	C	0.10250	0.50000	0.60250
1525	C3	C	0.10250	0.75000	0.85250
1526	C3	C	0.35250	0.50000	0.85250
1527	C3	C	0.35250	0.75000	0.60250
1528	C3	C	0.39750	0.50000	0.60250
1529	C3	C	0.39750	0.75000	0.85250
1530	C3	C	0.14750	0.50000	0.85250
1531	C3	C	0.14750	0.75000	0.60250
1532	C3	C	0.39750	0.50000	0.89750
1533	C3	C	0.39750	0.75000	0.64750
1534	C3	C	0.14750	0.50000	0.64750
1535	C3	C	0.14750	0.75000	0.89750
1536	C3	C	0.10250	0.50000	0.89750
1537	C3	C	0.10250	0.75000	0.64750
1538	C3	C	0.35250	0.50000	0.64750
1539	C3	C	0.35250	0.75000	0.89750
1540	C3	C	0.10250	0.60250	0.50000
1541	C3	C	0.10250	0.85250	0.75000
1542	C3	C	0.35250	0.60250	0.75000
1543	C3	C	0.35250	0.85250	0.50000
1544	C3	C	0.10250	0.89750	0.50000
1545	C3	C	0.10250	0.64750	0.75000
1546	C3	C	0.35250	0.89750	0.75000
1547	C3	C	0.39750	0.89750	0.50000
1548	C3	C	0.39750	0.64750	0.75000
1549	C3	C	0.14750	0.89750	0.75000
1550	C3	C	0.14750	0.64750	0.50000
1551	C3	C	0.39750	0.85250	0.75000
1552	C3	C	0.14750	0.60250	0.75000
1553	C3	C	0.14750	0.85250	0.50000
1554	C3	C	0.00000	0.60250	0.60250
1555	C3	C	0.00000	0.85250	0.85250
1556	C3	C	0.25000	0.60250	0.85250
1557	C3	C	0.25000	0.85250	0.60250
1558	C3	C	0.00000	0.60250	0.89750
1559	C3	C	0.00000	0.85250	0.64750
1560	C3	C	0.25000	0.60250	0.64750
1561	C3	C	0.25000	0.85250	0.89750
1562	C3	C	0.00000	0.89750	0.89750
1563	C3	C	0.00000	0.64750	0.64750
1564	C3	C	0.25000	0.89750	0.64750
1565	C3	C	0.25000	0.64750	0.89750
1566	C3	C	0.00000	0.89750	0.60250

1567	C3	C	0.00000	0.64750	0.85250
1568	C3	C	0.25000	0.89750	0.85250
1569	C3	C	0.25000	0.64750	0.60250
1570	H1	H	0.13926	0.99997	0.56655
1571	H1	H	0.13926	0.74997	0.81655
1572	H1	H	0.38926	0.99997	0.81655
1573	H1	H	0.38926	0.74997	0.56655
1574	H1	H	0.36074	0.50003	0.56655
1575	H1	H	0.36074	0.75004	0.81655
1576	H1	H	0.11074	0.50003	0.81655
1577	H1	H	0.11074	0.75004	0.56655
1578	H1	H	0.36074	0.99996	0.93345
1579	H1	H	0.36074	0.74997	0.68345
1580	H1	H	0.11074	0.99997	0.68345
1581	H1	H	0.11074	0.74997	0.93346
1582	H1	H	0.13926	0.50003	0.93345
1583	H1	H	0.13926	0.75003	0.68346
1584	H1	H	0.38926	0.50003	0.68345
1585	H1	H	0.38926	0.75004	0.93346
1586	H1	H	0.06655	0.63926	0.99996
1587	H1	H	0.06655	0.88926	0.74997
1588	H1	H	0.31655	0.63926	0.74996
1589	H1	H	0.31654	0.88926	0.99997
1590	H1	H	0.06655	0.86074	0.50003
1591	H1	H	0.06655	0.61074	0.75004
1592	H1	H	0.31655	0.86074	0.75003
1593	H1	H	0.43345	0.86074	0.99996
1594	H1	H	0.43345	0.61074	0.74997
1595	H1	H	0.18345	0.86074	0.74996
1596	H1	H	0.18345	0.61074	0.99997
1597	H1	H	0.43345	0.88926	0.75004
1598	H1	H	0.18345	0.63926	0.75003
1599	H1	H	0.18345	0.88926	0.50004
1600	H1	H	0.49997	0.56655	0.63926
1601	H1	H	0.49996	0.81655	0.88926
1602	H1	H	0.24997	0.56655	0.88926
1603	H1	H	0.24996	0.81655	0.63926
1604	H1	H	0.00003	0.56655	0.86074
1605	H1	H	0.00003	0.81655	0.61074
1606	H1	H	0.25003	0.56655	0.61074
1607	H1	H	0.25004	0.81655	0.86074
1608	H1	H	0.49997	0.93345	0.86074
1609	H1	H	0.49997	0.68345	0.61074
1610	H1	H	0.24997	0.93345	0.61074
1611	H1	H	0.24997	0.68345	0.86074
1612	H1	H	0.00003	0.93345	0.63926
1613	H1	H	0.00003	0.68345	0.88926
1614	H1	H	0.25004	0.93345	0.88926
1615	H1	H	0.25003	0.68346	0.63926
1616	H1	H	0.00003	0.63926	0.93345
1617	H1	H	0.00003	0.88926	0.68345
1618	H1	H	0.25003	0.63926	0.68345
1619	H1	H	0.25004	0.88926	0.93345
1620	H1	H	0.49996	0.86074	0.93345

1621	H1	H	0.49997	0.61074	0.68345
1622	H1	H	0.24997	0.86074	0.68345
1623	H1	H	0.24997	0.61074	0.93345
1624	H1	H	0.00003	0.86074	0.56655
1625	H1	H	0.00003	0.61074	0.81655
1626	H1	H	0.25004	0.86074	0.81655
1627	H1	H	0.25003	0.61074	0.56654
1628	H1	H	0.49997	0.63926	0.56655
1629	H1	H	0.49997	0.88926	0.81655
1630	H1	H	0.24997	0.63926	0.81655
1631	H1	H	0.24997	0.88926	0.56654
1632	H1	H	0.13926	0.56655	0.99997
1633	H1	H	0.13926	0.81655	0.74996
1634	H1	H	0.38926	0.56655	0.74997
1635	H1	H	0.38926	0.81655	0.99996
1636	H1	H	0.36074	0.81655	0.75003
1637	H1	H	0.11074	0.56655	0.75003
1638	H1	H	0.11074	0.81655	0.50003
1639	H1	H	0.36074	0.93345	0.99997
1640	H1	H	0.36074	0.68345	0.74997
1641	H1	H	0.11074	0.93345	0.74997
1642	H1	H	0.11074	0.68345	0.99996
1643	H1	H	0.13926	0.93345	0.50004
1644	H1	H	0.13926	0.68346	0.75003
1645	H1	H	0.38926	0.93345	0.75004
1646	H1	H	0.06655	0.50003	0.86074
1647	H1	H	0.06655	0.75004	0.61074
1648	H1	H	0.31655	0.50003	0.61074
1649	H1	H	0.31655	0.75004	0.86074
1650	H1	H	0.06655	0.99996	0.63926
1651	H1	H	0.06655	0.74997	0.88926
1652	H1	H	0.31655	0.99996	0.88926
1653	H1	H	0.31655	0.74997	0.63926
1654	H1	H	0.43345	0.50003	0.63926
1655	H1	H	0.43345	0.75004	0.88926
1656	H1	H	0.18345	0.50003	0.88926
1657	H1	H	0.18345	0.75003	0.63926
1658	H1	H	0.43345	0.99996	0.86074
1659	H1	H	0.43345	0.74997	0.61074
1660	H1	H	0.18345	0.99996	0.61074
1661	H1	H	0.18345	0.74997	0.86074
1662	H2	H	0.20825	0.79176	0.95824
1663	H2	H	0.29174	0.70825	0.95823
1664	H2	H	0.20827	0.70823	0.54177
1665	H2	H	0.29179	0.79168	0.54178
1666	H2	H	0.45823	0.79173	0.70823
1667	H2	H	0.45824	0.70825	0.79176
1668	H2	H	0.04174	0.79176	0.79177
1669	H2	H	0.04173	0.70823	0.70823
1670	H2	H	0.29175	0.95824	0.70824
1671	H2	H	0.20824	0.95824	0.79176
1672	H2	H	0.20823	0.54172	0.70822
1673	H2	H	0.29177	0.54173	0.79178
1674	H2	H	0.04178	0.54177	0.54173

1675	H2	H	0.45823	0.95824	0.54174
1676	H2	H	0.45823	0.54176	0.95825
1677	H2	H	0.04176	0.95824	0.95824
1678	O1	O	0.08524	0.50000	0.54765
1679	O1	O	0.08524	0.75000	0.79765
1680	O1	O	0.33524	0.50000	0.79765
1681	O1	O	0.33524	0.75000	0.54765
1682	O1	O	0.41476	0.50000	0.54765
1683	O1	O	0.41476	0.75000	0.79765
1684	O1	O	0.16476	0.50000	0.79765
1685	O1	O	0.16476	0.75000	0.54765
1686	O1	O	0.41476	0.50000	0.95235
1687	O1	O	0.41476	0.75000	0.70235
1688	O1	O	0.16476	0.50000	0.70235
1689	O1	O	0.16476	0.75000	0.95235
1690	O1	O	0.08524	0.50000	0.95235
1691	O1	O	0.08524	0.75000	0.70235
1692	O1	O	0.33524	0.50000	0.70235
1693	O1	O	0.33524	0.75000	0.95235
1694	O1	O	0.04765	0.58524	0.50000
1695	O1	O	0.04765	0.83524	0.75000
1696	O1	O	0.29765	0.58524	0.75000
1697	O1	O	0.29765	0.83524	0.50000
1698	O1	O	0.04765	0.91476	0.50000
1699	O1	O	0.04765	0.66476	0.75000
1700	O1	O	0.29765	0.91476	0.75000
1701	O3	O	0.29765	0.66476	0.50000
1702	O1	O	0.45235	0.91476	0.50000
1703	O1	O	0.45235	0.66476	0.75000
1704	O1	O	0.20235	0.91476	0.75000
1705	O1	O	0.20235	0.66476	0.50000
1706	O3	O	0.45235	0.58524	0.50000
1707	O1	O	0.45235	0.83524	0.75000
1708	O1	O	0.20235	0.58524	0.75000
1709	O1	O	0.20235	0.83524	0.50000
1710	O1	O	0.00000	0.54765	0.58524
1711	O1	O	0.00000	0.79765	0.83524
1712	O1	O	0.25000	0.54765	0.83524
1713	O1	O	0.25000	0.79765	0.58524
1714	O1	O	0.00000	0.54765	0.91476
1715	O1	O	0.00000	0.79765	0.66476
1716	O1	O	0.25000	0.54765	0.66476
1717	O1	O	0.25000	0.79765	0.91476
1718	O1	O	0.00000	0.95235	0.91476
1719	O1	O	0.00000	0.70235	0.66476
1720	O1	O	0.25000	0.95235	0.66476
1721	O1	O	0.25000	0.70235	0.91476
1722	O1	O	0.00000	0.95235	0.58524
1723	O1	O	0.00000	0.70235	0.83524
1724	O1	O	0.25000	0.95235	0.83524
1725	O1	O	0.25000	0.70235	0.58524
1726	O1	O	0.00000	0.58524	0.95235
1727	O1	O	0.00000	0.83524	0.70235
1728	O1	O	0.25000	0.58524	0.70235

1729	O1	O	0.25000	0.83524	0.95235
1730	O1	O	0.00000	0.91476	0.95235
1731	O1	O	0.00000	0.66476	0.70235
1732	O1	O	0.25000	0.91476	0.70235
1733	O1	O	0.25000	0.66476	0.95235
1734	O1	O	0.00000	0.91476	0.54765
1735	O1	O	0.00000	0.66476	0.79765
1736	O1	O	0.25000	0.91476	0.79765
1737	O1	O	0.25000	0.66476	0.54765
1738	O1	O	0.00000	0.58524	0.54765
1739	O1	O	0.00000	0.83524	0.79765
1740	O1	O	0.25000	0.58524	0.79765
1741	O1	O	0.25000	0.83524	0.54765
1742	O1	O	0.08524	0.54765	0.50000
1743	O1	O	0.08524	0.79765	0.75000
1744	O1	O	0.33524	0.54765	0.75000
1745	O1	O	0.33524	0.79765	0.50000
1746	O3	O	0.41476	0.54765	0.50000
1747	O1	O	0.41476	0.79765	0.75000
1748	O1	O	0.16476	0.54765	0.75000
1749	O1	O	0.16476	0.79765	0.50000
1750	O1	O	0.41476	0.95235	0.50000
1751	O1	O	0.41476	0.70235	0.75000
1752	O1	O	0.16476	0.95235	0.75000
1753	O1	O	0.16476	0.70235	0.50000
1754	O1	O	0.08524	0.95235	0.50000
1755	O1	O	0.08524	0.70235	0.75000
1756	O1	O	0.33524	0.95235	0.75000
1757	O3	O	0.33524	0.70235	0.50000
1758	O1	O	0.04765	0.50000	0.91476
1759	O1	O	0.04765	0.75000	0.66476
1760	O1	O	0.29765	0.50000	0.66476
1761	O1	O	0.29765	0.75000	0.91476
1762	O1	O	0.04765	0.50000	0.58524
1763	O1	O	0.04765	0.75000	0.83524
1764	O1	O	0.29765	0.50000	0.83524
1765	O1	O	0.29765	0.75000	0.58524
1766	O1	O	0.45235	0.50000	0.58524
1767	O1	O	0.45235	0.75000	0.83524
1768	O1	O	0.20235	0.50000	0.83524
1769	O1	O	0.20235	0.75000	0.58524
1770	O1	O	0.45235	0.50000	0.91476
1771	O1	O	0.45235	0.75000	0.66476
1772	O1	O	0.20235	0.50000	0.66476
1773	O1	O	0.20235	0.75000	0.91476
1774	O2	O	0.02805	0.97195	0.97195
1775	O2	O	0.02805	0.72195	0.72195
1776	O2	O	0.27805	0.97195	0.72195
1777	O2	O	0.27805	0.72195	0.97195
1778	O2	O	0.47195	0.52805	0.97195
1779	O2	O	0.47195	0.77805	0.72195
1780	O2	O	0.22195	0.52805	0.72195
1781	O2	O	0.22195	0.77805	0.97195
1782	O2	O	0.47195	0.97195	0.52805

1783	O2	O	0.47195	0.72195	0.77805
1784	O2	O	0.22195	0.97195	0.77805
1785	O2	O	0.22195	0.72195	0.52805
1786	O2	O	0.02805	0.52805	0.52805
1787	O2	O	0.02805	0.77805	0.77805
1788	O2	O	0.27805	0.52805	0.77805
1789	O2	O	0.27805	0.77805	0.52805
1790	O1	O	0.47195	0.52805	0.52805
1791	O1	O	0.47195	0.77805	0.77805
1792	O1	O	0.22195	0.52805	0.77805
1793	O1	O	0.22195	0.77805	0.52805
1794	O1	O	0.02805	0.97195	0.52805
1795	O1	O	0.02805	0.72195	0.77805
1796	O1	O	0.27805	0.97195	0.77805
1797	O1	O	0.27805	0.72195	0.52805
1798	O1	O	0.47195	0.97195	0.97195
1799	O1	O	0.47195	0.72195	0.72195
1800	O1	O	0.22195	0.97195	0.72195
1801	O1	O	0.22195	0.72195	0.97195
1802	O1	O	0.02805	0.52805	0.97195
1803	O1	O	0.02805	0.77805	0.72195
1804	O1	O	0.27805	0.52805	0.72195
1805	O1	O	0.27805	0.77805	0.97195
1806	Zr1	Zr	0.05994	0.50000	0.50000
1807	Zr1	Zr	0.05994	0.75000	0.75000
1808	Zr1	Zr	0.30994	0.50000	0.75000
1809	Zr1	Zr	0.30994	0.75000	0.50000
1810	Zr1	Zr	0.44006	0.50000	0.50000
1811	Zr1	Zr	0.44006	0.75000	0.75000
1812	Zr1	Zr	0.19006	0.50000	0.75000
1813	Zr1	Zr	0.19006	0.75000	0.50000
1814	Zr1	Zr	0.00000	0.55994	0.50000
1815	Zr1	Zr	0.00000	0.80994	0.75000
1816	Zr1	Zr	0.25000	0.55994	0.75000
1817	Zr1	Zr	0.25000	0.80994	0.50000
1818	Zr1	Zr	0.00000	0.94006	0.50000
1819	Zr1	Zr	0.00000	0.69006	0.75000
1820	Zr1	Zr	0.25000	0.94006	0.75000
1821	Zr1	Zr	0.25000	0.69006	0.50000
1822	Zr1	Zr	0.00000	0.50000	0.55994
1823	Zr1	Zr	0.00000	0.75000	0.80994
1824	Zr1	Zr	0.25000	0.50000	0.80994
1825	Zr1	Zr	0.25000	0.75000	0.55994
1826	Zr1	Zr	0.00000	0.50000	0.94006
1827	Zr1	Zr	0.00000	0.75000	0.69006
1828	Zr1	Zr	0.25000	0.50000	0.69006
1829	Zr1	Zr	0.25000	0.75000	0.94006
1830	C2	C	0.63375	0.00000	0.09220
1831	C2	C	0.63375	0.25000	0.34220
1832	C2	C	0.88375	0.00000	0.34220
1833	C2	C	0.88375	0.25000	0.09220
1834	C2	C	0.86625	0.00000	0.09220
1835	C2	C	0.86625	0.25000	0.34220
1836	C2	C	0.61625	0.00000	0.34220

1837	C2	C	0.61625	0.25000	0.09220
1838	C2	C	0.86625	0.00000	0.40780
1839	C2	C	0.86625	0.25000	0.15780
1840	C2	C	0.61625	0.00000	0.15780
1841	C2	C	0.61625	0.25000	0.40780
1842	C2	C	0.63375	0.00000	0.40780
1843	C2	C	0.63375	0.25000	0.15780
1844	C2	C	0.88375	0.00000	0.15780
1845	C2	C	0.88375	0.25000	0.40780
1846	C2	C	0.59220	0.13375	0.00000
1847	C2	C	0.59220	0.38375	0.25000
1848	C2	C	0.84220	0.13375	0.25000
1849	C2	C	0.84220	0.38375	0.00000
1850	C2	C	0.59220	0.36625	0.00000
1851	C2	C	0.59220	0.11625	0.25000
1852	C2	C	0.84220	0.36625	0.25000
1853	C2	C	0.84220	0.11625	0.00000
1854	C2	C	0.90780	0.36625	0.00000
1855	C2	C	0.90780	0.11625	0.25000
1856	C2	C	0.65780	0.36625	0.25000
1857	C2	C	0.65780	0.11625	0.00000
1858	C2	C	0.90780	0.13375	0.00000
1859	C2	C	0.90780	0.38375	0.25000
1860	C2	C	0.65780	0.13375	0.25000
1861	C2	C	0.65780	0.38375	0.00000
1862	C2	C	0.50000	0.09220	0.13375
1863	C2	C	0.50000	0.34220	0.38375
1864	C2	C	0.75000	0.09220	0.38375
1865	C2	C	0.75000	0.34220	0.13375
1866	C2	C	0.50000	0.09220	0.36625
1867	C2	C	0.50000	0.34220	0.11625
1868	C2	C	0.75000	0.09220	0.11625
1869	C2	C	0.75000	0.34220	0.36625
1870	C2	C	0.50000	0.40780	0.36625
1871	C2	C	0.50000	0.15780	0.11625
1872	C2	C	0.75000	0.40780	0.11625
1873	C2	C	0.75000	0.15780	0.36625
1874	C2	C	0.50000	0.40780	0.13375
1875	C2	C	0.50000	0.15780	0.38375
1876	C2	C	0.75000	0.40780	0.38375
1877	C2	C	0.75000	0.15780	0.13375
1878	C2	C	0.50000	0.13375	0.40780
1879	C2	C	0.50000	0.38375	0.15780
1880	C2	C	0.75000	0.13375	0.15780
1881	C2	C	0.75000	0.38375	0.40780
1882	C2	C	0.50000	0.36625	0.40780
1883	C2	C	0.50000	0.11625	0.15780
1884	C2	C	0.75000	0.36625	0.15780
1885	C2	C	0.75000	0.11625	0.40780
1886	C2	C	0.50000	0.36625	0.09220
1887	C2	C	0.50000	0.11625	0.34220
1888	C2	C	0.75000	0.36625	0.34220
1889	C2	C	0.75000	0.11625	0.09220
1890	C2	C	0.50000	0.13375	0.09220

1891	C2	C	0.50000	0.38375	0.34220
1892	C2	C	0.75000	0.13375	0.34220
1893	C2	C	0.75000	0.38375	0.09220
1894	C2	C	0.63375	0.09220	0.00000
1895	C2	C	0.63375	0.34220	0.25000
1896	C2	C	0.88375	0.09220	0.25000
1897	C2	C	0.88375	0.34220	0.00000
1898	C2	C	0.86625	0.09220	0.00000
1899	C2	C	0.86625	0.34220	0.25000
1900	C2	C	0.61625	0.09220	0.25000
1901	C2	C	0.61625	0.34220	0.00000
1902	C2	C	0.86625	0.40780	0.00000
1903	C2	C	0.86625	0.15780	0.25000
1904	C2	C	0.61625	0.40780	0.25000
1905	C2	C	0.61625	0.15780	0.00000
1906	C2	C	0.63375	0.40780	0.00000
1907	C2	C	0.63375	0.15780	0.25000
1908	C2	C	0.88375	0.40780	0.25000
1909	C2	C	0.88375	0.15780	0.00000
1910	C2	C	0.59220	0.00000	0.36625
1911	C2	C	0.59220	0.25000	0.11625
1912	C2	C	0.84220	0.00000	0.11625
1913	C2	C	0.84220	0.25000	0.36625
1914	C2	C	0.59220	0.00000	0.13375
1915	C2	C	0.59220	0.25000	0.38375
1916	C2	C	0.84220	0.00000	0.38375
1917	C2	C	0.84220	0.25000	0.13375
1918	C2	C	0.90780	0.00000	0.13375
1919	C2	C	0.90780	0.25000	0.38375
1920	C2	C	0.65780	0.00000	0.38375
1921	C2	C	0.65780	0.25000	0.13375
1922	C2	C	0.90780	0.00000	0.36625
1923	C2	C	0.90780	0.25000	0.11625
1924	C2	C	0.65780	0.00000	0.11625
1925	C2	C	0.65780	0.25000	0.36625
1926	C1	C	0.57680	0.00000	0.42320
1927	C1	C	0.57680	0.25000	0.17320
1928	C1	C	0.82680	0.00000	0.17320
1929	C1	C	0.82680	0.25000	0.42320
1930	C1	C	0.92320	0.00000	0.42320
1931	C1	C	0.92320	0.25000	0.17320
1932	C1	C	0.67320	0.00000	0.17320
1933	C1	C	0.67320	0.25000	0.42320
1934	C1	C	0.92320	0.00000	0.07680
1935	C1	C	0.92320	0.25000	0.32680
1936	C1	C	0.67320	0.00000	0.32680
1937	C1	C	0.67320	0.25000	0.07680
1938	C1	C	0.57680	0.00000	0.07680
1939	C1	C	0.57680	0.25000	0.32680
1940	C1	C	0.82680	0.00000	0.32680
1941	C1	C	0.82680	0.25000	0.07680
1942	C1	C	0.92320	0.07680	0.00000
1943	C1	C	0.92320	0.32680	0.25000
1944	C1	C	0.67320	0.07680	0.25000

1945	C1	C	0.67320	0.32680	0.00000
1946	C1	C	0.92320	0.42320	0.00000
1947	C1	C	0.92320	0.17320	0.25000
1948	C1	C	0.67320	0.42320	0.25000
1949	C1	C	0.67320	0.17320	0.00000
1950	C1	C	0.57680	0.42320	0.00000
1951	C1	C	0.57680	0.17320	0.25000
1952	C1	C	0.82680	0.42320	0.25000
1953	C1	C	0.82680	0.17320	0.00000
1954	C1	C	0.57680	0.07680	0.00000
1955	C1	C	0.57680	0.32680	0.25000
1956	C1	C	0.82680	0.07680	0.25000
1957	C1	C	0.82680	0.32680	0.00000
1958	C1	C	0.50000	0.42320	0.07680
1959	C1	C	0.50000	0.17320	0.32680
1960	C1	C	0.75000	0.42320	0.32680
1961	C1	C	0.75000	0.17320	0.07680
1962	C1	C	0.50000	0.42320	0.42320
1963	C1	C	0.50000	0.17320	0.17320
1964	C1	C	0.75000	0.42320	0.17320
1965	C1	C	0.75000	0.17320	0.42320
1966	C1	C	0.50000	0.07680	0.42320
1967	C1	C	0.50000	0.32680	0.17320
1968	C1	C	0.75000	0.07680	0.17320
1969	C1	C	0.75000	0.32680	0.42320
1970	C1	C	0.50000	0.07680	0.07680
1971	C1	C	0.50000	0.32680	0.32680
1972	C1	C	0.75000	0.07680	0.32680
1973	C1	C	0.75000	0.32680	0.07680
1974	C3	C	0.60250	0.00000	0.10250
1975	C3	C	0.60250	0.25000	0.35250
1976	C3	C	0.85250	0.00000	0.35250
1977	C3	C	0.85250	0.25000	0.10250
1978	C3	C	0.89750	0.00000	0.10250
1979	C3	C	0.89750	0.25000	0.35250
1980	C3	C	0.64750	0.00000	0.35250
1981	C3	C	0.64750	0.25000	0.10250
1982	C3	C	0.89750	0.00000	0.39750
1983	C3	C	0.89750	0.25000	0.14750
1984	C3	C	0.64750	0.00000	0.14750
1985	C3	C	0.64750	0.25000	0.39750
1986	C3	C	0.60250	0.00000	0.39750
1987	C3	C	0.60250	0.25000	0.14750
1988	C3	C	0.85250	0.00000	0.14750
1989	C3	C	0.85250	0.25000	0.39750
1990	C3	C	0.60250	0.10250	0.00000
1991	C3	C	0.60250	0.35250	0.25000
1992	C3	C	0.85250	0.10250	0.25000
1993	C3	C	0.85250	0.35250	0.00000
1994	C3	C	0.60250	0.39750	0.00000
1995	C3	C	0.60250	0.14750	0.25000
1996	C3	C	0.85250	0.39750	0.25000
1997	C3	C	0.85250	0.14750	0.00000
1998	C3	C	0.89750	0.39750	0.00000

1999	C3	C	0.89750	0.14750	0.25000
2000	C3	C	0.64750	0.39750	0.25000
2001	C3	C	0.64750	0.14750	0.00000
2002	C3	C	0.89750	0.10250	0.00000
2003	C3	C	0.89750	0.35250	0.25000
2004	C3	C	0.64750	0.10250	0.25000
2005	C3	C	0.64750	0.35250	0.00000
2006	C3	C	0.50000	0.10250	0.10250
2007	C3	C	0.50000	0.35250	0.35250
2008	C3	C	0.75000	0.10250	0.35250
2009	C3	C	0.75000	0.35250	0.10250
2010	C3	C	0.50000	0.10250	0.39750
2011	C3	C	0.50000	0.35250	0.14750
2012	C3	C	0.75000	0.10250	0.14750
2013	C3	C	0.75000	0.35250	0.39750
2014	C3	C	0.50000	0.39750	0.39750
2015	C3	C	0.50000	0.14750	0.14750
2016	C3	C	0.75000	0.39750	0.14750
2017	C3	C	0.75000	0.14750	0.39750
2018	C3	C	0.50000	0.39750	0.10250
2019	C3	C	0.50000	0.14750	0.35250
2020	C3	C	0.75000	0.39750	0.35250
2021	C3	C	0.75000	0.14750	0.10250
2022	H1	H	0.63926	0.49997	0.06655
2023	H1	H	0.63926	0.24997	0.31655
2024	H1	H	0.88926	0.49997	0.31655
2025	H1	H	0.88926	0.24997	0.06655
2026	H1	H	0.86074	0.00003	0.06655
2027	H1	H	0.86074	0.25004	0.31655
2028	H1	H	0.61074	0.00003	0.31655
2029	H1	H	0.61074	0.25004	0.06655
2030	H1	H	0.86074	0.49996	0.43345
2031	H1	H	0.86074	0.24997	0.18345
2032	H1	H	0.61074	0.49997	0.18345
2033	H1	H	0.61074	0.24997	0.43346
2034	H1	H	0.63926	0.00003	0.43345
2035	H1	H	0.63926	0.25003	0.18346
2036	H1	H	0.88926	0.00003	0.18345
2037	H1	H	0.88926	0.25004	0.43346
2038	H1	H	0.56655	0.13926	0.49996
2039	H1	H	0.56655	0.38926	0.24997
2040	H1	H	0.81655	0.13926	0.24996
2041	H1	H	0.81654	0.38926	0.49997
2042	H1	H	0.56655	0.36074	0.00003
2043	H1	H	0.56655	0.11074	0.25004
2044	H1	H	0.81655	0.36074	0.25003
2045	H1	H	0.81655	0.11074	0.00004
2046	H1	H	0.93345	0.36074	0.49996
2047	H1	H	0.93345	0.11074	0.24997
2048	H1	H	0.68345	0.36074	0.24996
2049	H1	H	0.68345	0.11074	0.49997
2050	H1	H	0.93345	0.13926	0.00003
2051	H1	H	0.93345	0.38926	0.25004
2052	H1	H	0.68345	0.13926	0.25003

2053	H1	H	0.68345	0.38926	0.00004
2054	H1	H	0.99997	0.06655	0.13926
2055	H1	H	0.99996	0.31655	0.38926
2056	H1	H	0.74997	0.06655	0.38926
2057	H1	H	0.74996	0.31655	0.13926
2058	H1	H	0.50003	0.06655	0.36074
2059	H1	H	0.50003	0.31655	0.11074
2060	H1	H	0.75003	0.06655	0.11074
2061	H1	H	0.75004	0.31655	0.36074
2062	H1	H	0.99997	0.43345	0.36074
2063	H1	H	0.99997	0.18345	0.11074
2064	H1	H	0.74997	0.43345	0.11074
2065	H1	H	0.74997	0.18345	0.36074
2066	H1	H	0.50003	0.43345	0.13926
2067	H1	H	0.50003	0.18345	0.38926
2068	H1	H	0.75004	0.43345	0.38926
2069	H1	H	0.75003	0.18346	0.13926
2070	H1	H	0.50003	0.13926	0.43345
2071	H1	H	0.50003	0.38926	0.18345
2072	H1	H	0.75003	0.13926	0.18345
2073	H1	H	0.75004	0.38926	0.43345
2074	H1	H	0.99996	0.36074	0.43345
2075	H1	H	0.99997	0.11074	0.18345
2076	H1	H	0.74997	0.36074	0.18345
2077	H1	H	0.74997	0.11074	0.43345
2078	H1	H	0.50003	0.36074	0.06655
2079	H1	H	0.50003	0.11074	0.31655
2080	H1	H	0.75004	0.36074	0.31655
2081	H1	H	0.75003	0.11074	0.06654
2082	H1	H	0.99997	0.13926	0.06655
2083	H1	H	0.99997	0.38926	0.31655
2084	H1	H	0.74997	0.13926	0.31655
2085	H1	H	0.74997	0.38926	0.06654
2086	H1	H	0.63926	0.06655	0.49997
2087	H1	H	0.63926	0.31655	0.24996
2088	H1	H	0.88926	0.06655	0.24997
2089	H1	H	0.88926	0.31655	0.49996
2090	H1	H	0.86074	0.06655	0.00003
2091	H1	H	0.86074	0.31655	0.25003
2092	H1	H	0.61074	0.06655	0.25003
2093	H1	H	0.61074	0.31655	0.00003
2094	H1	H	0.86074	0.43345	0.49997
2095	H1	H	0.86074	0.18345	0.24997
2096	H1	H	0.61074	0.43345	0.24997
2097	H1	H	0.61074	0.18345	0.49996
2098	H1	H	0.63926	0.43345	0.00004
2099	H1	H	0.63926	0.18346	0.25003
2100	H1	H	0.88926	0.43345	0.25004
2101	H1	H	0.88926	0.18346	0.00003
2102	H1	H	0.56655	0.00003	0.36074
2103	H1	H	0.56655	0.25004	0.11074
2104	H1	H	0.81655	0.00003	0.11074
2105	H1	H	0.81655	0.25004	0.36074
2106	H1	H	0.56655	0.49996	0.13926

2107	H1	H	0.56655	0.24997	0.38926
2108	H1	H	0.81655	0.49996	0.38926
2109	H1	H	0.81655	0.24997	0.13926
2110	H1	H	0.93345	0.00003	0.13926
2111	H1	H	0.93345	0.25004	0.38926
2112	H1	H	0.68345	0.00003	0.38926
2113	H1	H	0.68345	0.25003	0.13926
2114	H1	H	0.93345	0.49996	0.36074
2115	H1	H	0.93345	0.24997	0.11074
2116	H1	H	0.68345	0.49996	0.11074
2117	H1	H	0.68345	0.24997	0.36074
2118	H2	H	0.70825	0.29176	0.45824
2119	H2	H	0.79174	0.20825	0.45823
2120	H2	H	0.70827	0.20823	0.04177
2121	H2	H	0.79179	0.29168	0.04178
2122	H2	H	0.95823	0.29173	0.20823
2123	H2	H	0.95824	0.20825	0.29176
2124	H2	H	0.54174	0.29176	0.29177
2125	H2	H	0.54173	0.20823	0.20823
2126	H2	H	0.79175	0.45824	0.20824
2127	H2	H	0.70824	0.45824	0.29176
2128	H2	H	0.70823	0.04172	0.20822
2129	H2	H	0.79177	0.04173	0.29178
2130	H2	H	0.54178	0.04177	0.04173
2131	H2	H	0.95823	0.45824	0.04174
2132	H2	H	0.95823	0.04176	0.45825
2133	H2	H	0.54176	0.45824	0.45824
2134	O1	O	0.58524	0.00000	0.04765
2135	O1	O	0.58524	0.25000	0.29765
2136	O1	O	0.83524	0.00000	0.29765
2137	O1	O	0.83524	0.25000	0.04765
2138	O1	O	0.91476	0.00000	0.04765
2139	O1	O	0.91476	0.25000	0.29765
2140	O1	O	0.66476	0.00000	0.29765
2141	O1	O	0.66476	0.25000	0.04765
2142	O1	O	0.91476	0.00000	0.45235
2143	O1	O	0.91476	0.25000	0.20235
2144	O1	O	0.66476	0.00000	0.20235
2145	O1	O	0.66476	0.25000	0.45235
2146	O1	O	0.58524	0.00000	0.45235
2147	O1	O	0.58524	0.25000	0.20235
2148	O1	O	0.83524	0.00000	0.20235
2149	O1	O	0.83524	0.25000	0.45235
2150	O1	O	0.54765	0.08524	0.00000
2151	O1	O	0.54765	0.33524	0.25000
2152	O1	O	0.79765	0.08524	0.25000
2153	O1	O	0.79765	0.33524	0.00000
2154	O1	O	0.54765	0.41476	0.00000
2155	O1	O	0.54765	0.16476	0.25000
2156	O1	O	0.79765	0.41476	0.25000
2157	O1	O	0.79765	0.16476	0.00000
2158	O1	O	0.95235	0.41476	0.00000
2159	O1	O	0.95235	0.16476	0.25000
2160	O1	O	0.70235	0.41476	0.25000

2161	O1	O	0.70235	0.16476	0.00000
2162	O1	O	0.95235	0.08524	0.00000
2163	O1	O	0.95235	0.33524	0.25000
2164	O1	O	0.70235	0.08524	0.25000
2165	O1	O	0.70235	0.33524	0.00000
2166	O1	O	0.50000	0.04765	0.08524
2167	O1	O	0.50000	0.29765	0.33524
2168	O1	O	0.75000	0.04765	0.33524
2169	O1	O	0.75000	0.29765	0.08524
2170	O1	O	0.50000	0.04765	0.41476
2171	O1	O	0.50000	0.29765	0.16476
2172	O1	O	0.75000	0.04765	0.16476
2173	O1	O	0.75000	0.29765	0.41476
2174	O1	O	0.50000	0.45235	0.41476
2175	O1	O	0.50000	0.20235	0.16476
2176	O1	O	0.75000	0.45235	0.16476
2177	O1	O	0.75000	0.20235	0.41476
2178	O1	O	0.50000	0.45235	0.08524
2179	O1	O	0.50000	0.20235	0.33524
2180	O1	O	0.75000	0.45235	0.33524
2181	O1	O	0.75000	0.20235	0.08524
2182	O1	O	0.50000	0.08524	0.45235
2183	O1	O	0.50000	0.33524	0.20235
2184	O1	O	0.75000	0.08524	0.20235
2185	O1	O	0.75000	0.33524	0.45235
2186	O1	O	0.50000	0.41476	0.45235
2187	O1	O	0.50000	0.16476	0.20235
2188	O1	O	0.75000	0.41476	0.20235
2189	O1	O	0.75000	0.16476	0.45235
2190	O1	O	0.50000	0.41476	0.04765
2191	O1	O	0.50000	0.16476	0.29765
2192	O1	O	0.75000	0.41476	0.29765
2193	O1	O	0.75000	0.16476	0.04765
2194	O1	O	0.50000	0.08524	0.04765
2195	O1	O	0.50000	0.33524	0.29765
2196	O1	O	0.75000	0.08524	0.29765
2197	O1	O	0.75000	0.33524	0.04765
2198	O1	O	0.58524	0.04765	0.00000
2199	O1	O	0.58524	0.29765	0.25000
2200	O1	O	0.83524	0.04765	0.25000
2201	O1	O	0.83524	0.29765	0.00000
2202	O1	O	0.91476	0.04765	0.00000
2203	O1	O	0.91476	0.29765	0.25000
2204	O1	O	0.66476	0.04765	0.25000
2205	O1	O	0.66476	0.29765	0.00000
2206	O1	O	0.91476	0.45235	0.00000
2207	O1	O	0.91476	0.20235	0.25000
2208	O1	O	0.66476	0.45235	0.25000
2209	O1	O	0.66476	0.20235	0.00000
2210	O1	O	0.58524	0.45235	0.00000
2211	O1	O	0.58524	0.20235	0.25000
2212	O1	O	0.83524	0.45235	0.25000
2213	O1	O	0.83524	0.20235	0.00000
2214	O1	O	0.54765	0.00000	0.41476

2215	O1	O	0.54765	0.25000	0.16476
2216	O1	O	0.79765	0.00000	0.16476
2217	O1	O	0.79765	0.25000	0.41476
2218	O1	O	0.54765	0.00000	0.08524
2219	O1	O	0.54765	0.25000	0.33524
2220	O1	O	0.79765	0.00000	0.33524
2221	O1	O	0.79765	0.25000	0.08524
2222	O1	O	0.95235	0.00000	0.08524
2223	O1	O	0.95235	0.25000	0.33524
2224	O1	O	0.70235	0.00000	0.33524
2225	O1	O	0.70235	0.25000	0.08524
2226	O1	O	0.95235	0.00000	0.41476
2227	O1	O	0.95235	0.25000	0.16476
2228	O1	O	0.70235	0.00000	0.16476
2229	O1	O	0.70235	0.25000	0.41476
2230	O2	O	0.52805	0.47195	0.47195
2231	O2	O	0.52805	0.22195	0.22195
2232	O2	O	0.77805	0.47195	0.22195
2233	O2	O	0.77805	0.22195	0.47195
2234	O2	O	0.97195	0.02805	0.47195
2235	O2	O	0.97195	0.27805	0.22195
2236	O2	O	0.72195	0.02805	0.22195
2237	O2	O	0.72195	0.27805	0.47195
2238	O2	O	0.97195	0.47195	0.02805
2239	O2	O	0.97195	0.22195	0.27805
2240	O2	O	0.72195	0.47195	0.27805
2241	O2	O	0.72195	0.22195	0.02805
2242	O2	O	0.52805	0.02805	0.02805
2243	O2	O	0.52805	0.27805	0.27805
2244	O2	O	0.77805	0.02805	0.27805
2245	O2	O	0.77805	0.27805	0.02805
2246	O1	O	0.97195	0.02805	0.02805
2247	O1	O	0.97195	0.27805	0.27805
2248	O1	O	0.72195	0.02805	0.27805
2249	O1	O	0.72195	0.27805	0.02805
2250	O1	O	0.52805	0.47195	0.02805
2251	O1	O	0.52805	0.22195	0.27805
2252	O1	O	0.77805	0.47195	0.27805
2253	O1	O	0.77805	0.22195	0.02805
2254	O1	O	0.97195	0.47195	0.47195
2255	O1	O	0.97195	0.22195	0.22195
2256	O1	O	0.72195	0.47195	0.22195
2257	O1	O	0.72195	0.22195	0.47195
2258	O1	O	0.52805	0.02805	0.47195
2259	O1	O	0.52805	0.27805	0.22195
2260	O1	O	0.77805	0.02805	0.22195
2261	O1	O	0.77805	0.27805	0.47195
2262	Zr1	Zr	0.55994	0.00000	0.00000
2263	Zr1	Zr	0.55994	0.25000	0.25000
2264	Zr1	Zr	0.80994	0.00000	0.25000
2265	Zr1	Zr	0.80994	0.25000	0.00000
2266	Zr1	Zr	0.94006	0.00000	0.00000
2267	Zr1	Zr	0.94006	0.25000	0.25000
2268	Zr1	Zr	0.69006	0.00000	0.25000

2269	Zr1	Zr	0.69006	0.25000	0.00000
2270	Zr1	Zr	0.50000	0.05994	0.00000
2271	Zr1	Zr	0.50000	0.30994	0.25000
2272	Zr1	Zr	0.75000	0.05994	0.25000
2273	Zr1	Zr	0.75000	0.30994	0.00000
2274	Zr1	Zr	0.50000	0.44006	0.00000
2275	Zr1	Zr	0.50000	0.19006	0.25000
2276	Zr1	Zr	0.75000	0.44006	0.25000
2277	Zr1	Zr	0.75000	0.19006	0.00000
2278	Zr1	Zr	0.50000	0.00000	0.05994
2279	Zr1	Zr	0.50000	0.25000	0.30994
2280	Zr1	Zr	0.75000	0.00000	0.30994
2281	Zr1	Zr	0.75000	0.25000	0.05994
2282	Zr1	Zr	0.50000	0.00000	0.44006
2283	Zr1	Zr	0.50000	0.25000	0.19006
2284	Zr1	Zr	0.75000	0.00000	0.19006
2285	Zr1	Zr	0.75000	0.25000	0.44006
2286	C2	C	0.63375	0.00000	0.59220
2287	C2	C	0.63375	0.25000	0.84220
2288	C2	C	0.88375	0.00000	0.84220
2289	C2	C	0.88375	0.25000	0.59220
2290	C2	C	0.86625	0.00000	0.59220
2291	C2	C	0.86625	0.25000	0.84220
2292	C2	C	0.61625	0.00000	0.84220
2293	C2	C	0.61625	0.25000	0.59220
2294	C2	C	0.86625	0.00000	0.90780
2295	C2	C	0.86625	0.25000	0.65780
2296	C2	C	0.61625	0.00000	0.65780
2297	C2	C	0.61625	0.25000	0.90780
2298	C2	C	0.63375	0.00000	0.90780
2299	C2	C	0.63375	0.25000	0.65780
2300	C2	C	0.88375	0.00000	0.65780
2301	C2	C	0.88375	0.25000	0.90780
2302	C2	C	0.59220	0.13375	0.50000
2303	C2	C	0.59220	0.38375	0.75000
2304	C2	C	0.84220	0.13375	0.75000
2305	C2	C	0.84220	0.38375	0.50000
2306	C2	C	0.59220	0.36625	0.50000
2307	C2	C	0.59220	0.11625	0.75000
2308	C2	C	0.84220	0.36625	0.75000
2309	C2	C	0.84220	0.11625	0.50000
2310	C2	C	0.90780	0.36625	0.50000
2311	C2	C	0.90780	0.11625	0.75000
2312	C2	C	0.65780	0.36625	0.75000
2313	C2	C	0.65780	0.11625	0.50000
2314	C2	C	0.90780	0.13375	0.50000
2315	C2	C	0.90780	0.38375	0.75000
2316	C2	C	0.65780	0.13375	0.75000
2317	C2	C	0.65780	0.38375	0.50000
2318	C2	C	0.50000	0.09220	0.63375
2319	C2	C	0.50000	0.34220	0.88375
2320	C2	C	0.75000	0.09220	0.88375
2321	C2	C	0.75000	0.34220	0.63375
2322	C2	C	0.50000	0.09220	0.86625

2323	C2	C	0.50000	0.34220	0.61625
2324	C2	C	0.75000	0.09220	0.61625
2325	C2	C	0.75000	0.34220	0.86625
2326	C2	C	0.50000	0.40780	0.86625
2327	C2	C	0.50000	0.15780	0.61625
2328	C2	C	0.75000	0.40780	0.61625
2329	C2	C	0.75000	0.15780	0.86625
2330	C2	C	0.50000	0.40780	0.63375
2331	C2	C	0.50000	0.15780	0.88375
2332	C2	C	0.75000	0.40780	0.88375
2333	C2	C	0.75000	0.15780	0.63375
2334	C2	C	0.50000	0.13375	0.90780
2335	C2	C	0.50000	0.38375	0.65780
2336	C2	C	0.75000	0.13375	0.65780
2337	C2	C	0.75000	0.38375	0.90780
2338	C2	C	0.50000	0.36625	0.90780
2339	C2	C	0.50000	0.11625	0.65780
2340	C2	C	0.75000	0.36625	0.65780
2341	C2	C	0.75000	0.11625	0.90780
2342	C2	C	0.50000	0.36625	0.59220
2343	C2	C	0.50000	0.11625	0.84220
2344	C2	C	0.75000	0.36625	0.84220
2345	C2	C	0.75000	0.11625	0.59220
2346	C2	C	0.50000	0.13375	0.59220
2347	C2	C	0.50000	0.38375	0.84220
2348	C2	C	0.75000	0.13375	0.84220
2349	C2	C	0.75000	0.38375	0.59220
2350	C2	C	0.63375	0.09220	0.50000
2351	C2	C	0.63375	0.34220	0.75000
2352	C2	C	0.88375	0.09220	0.75000
2353	C2	C	0.88375	0.34220	0.50000
2354	C2	C	0.86625	0.09220	0.50000
2355	C2	C	0.86625	0.34220	0.75000
2356	C2	C	0.61625	0.09220	0.75000
2357	C2	C	0.61625	0.34220	0.50000
2358	C2	C	0.86625	0.40780	0.50000
2359	C2	C	0.86625	0.15780	0.75000
2360	C2	C	0.61625	0.40780	0.75000
2361	C2	C	0.61625	0.15780	0.50000
2362	C2	C	0.63375	0.40780	0.50000
2363	C2	C	0.63375	0.15780	0.75000
2364	C2	C	0.88375	0.40780	0.75000
2365	C2	C	0.88375	0.15780	0.50000
2366	C2	C	0.59220	0.00000	0.86625
2367	C2	C	0.59220	0.25000	0.61625
2368	C2	C	0.84220	0.00000	0.61625
2369	C2	C	0.84220	0.25000	0.86625
2370	C2	C	0.59220	0.00000	0.63375
2371	C2	C	0.59220	0.25000	0.88375
2372	C2	C	0.84220	0.00000	0.88375
2373	C2	C	0.84220	0.25000	0.63375
2374	C2	C	0.90780	0.00000	0.63375
2375	C2	C	0.90780	0.25000	0.88375
2376	C2	C	0.65780	0.00000	0.88375

2377	C2	C	0.65780	0.25000	0.63375
2378	C2	C	0.90780	0.00000	0.86625
2379	C2	C	0.90780	0.25000	0.61625
2380	C2	C	0.65780	0.00000	0.61625
2381	C2	C	0.65780	0.25000	0.86625
2382	C1	C	0.57680	0.00000	0.92320
2383	C1	C	0.57680	0.25000	0.67320
2384	C1	C	0.82680	0.00000	0.67320
2385	C1	C	0.82680	0.25000	0.92320
2386	C1	C	0.92320	0.00000	0.92320
2387	C1	C	0.92320	0.25000	0.67320
2388	C1	C	0.67320	0.00000	0.67320
2389	C1	C	0.67320	0.25000	0.92320
2390	C1	C	0.92320	0.00000	0.57680
2391	C1	C	0.92320	0.25000	0.82680
2392	C1	C	0.67320	0.00000	0.82680
2393	C1	C	0.67320	0.25000	0.57680
2394	C1	C	0.57680	0.00000	0.57680
2395	C1	C	0.57680	0.25000	0.82680
2396	C1	C	0.82680	0.00000	0.82680
2397	C1	C	0.82680	0.25000	0.57680
2398	C1	C	0.92320	0.07680	0.50000
2399	C1	C	0.92320	0.32680	0.75000
2400	C1	C	0.67320	0.07680	0.75000
2401	C1	C	0.67320	0.32680	0.50000
2402	C1	C	0.92320	0.42320	0.50000
2403	C1	C	0.92320	0.17320	0.75000
2404	C1	C	0.67320	0.42320	0.75000
2405	C1	C	0.67320	0.17320	0.50000
2406	C1	C	0.57680	0.42320	0.50000
2407	C1	C	0.57680	0.17320	0.75000
2408	C1	C	0.82680	0.42320	0.75000
2409	C1	C	0.82680	0.17320	0.50000
2410	C1	C	0.57680	0.07680	0.50000
2411	C1	C	0.57680	0.32680	0.75000
2412	C1	C	0.82680	0.07680	0.75000
2413	C1	C	0.82680	0.32680	0.50000
2414	C1	C	0.50000	0.42320	0.57680
2415	C1	C	0.50000	0.17320	0.82680
2416	C1	C	0.75000	0.42320	0.82680
2417	C1	C	0.75000	0.17320	0.57680
2418	C1	C	0.50000	0.42320	0.92320
2419	C1	C	0.50000	0.17320	0.67320
2420	C1	C	0.75000	0.42320	0.67320
2421	C1	C	0.75000	0.17320	0.92320
2422	C1	C	0.50000	0.07680	0.92320
2423	C1	C	0.50000	0.32680	0.67320
2424	C1	C	0.75000	0.07680	0.67320
2425	C1	C	0.75000	0.32680	0.92320
2426	C1	C	0.50000	0.07680	0.57680
2427	C1	C	0.50000	0.32680	0.82680
2428	C1	C	0.75000	0.07680	0.82680
2429	C1	C	0.75000	0.32680	0.57680
2430	C3	C	0.60250	0.00000	0.60250

2431	C3	C	0.60250	0.25000	0.85250
2432	C3	C	0.85250	0.00000	0.85250
2433	C3	C	0.85250	0.25000	0.60250
2434	C3	C	0.89750	0.00000	0.60250
2435	C3	C	0.89750	0.25000	0.85250
2436	C3	C	0.64750	0.00000	0.85250
2437	C3	C	0.64750	0.25000	0.60250
2438	C3	C	0.89750	0.00000	0.89750
2439	C3	C	0.89750	0.25000	0.64750
2440	C3	C	0.64750	0.00000	0.64750
2441	C3	C	0.64750	0.25000	0.89750
2442	C3	C	0.60250	0.00000	0.89750
2443	C3	C	0.60250	0.25000	0.64750
2444	C3	C	0.85250	0.00000	0.64750
2445	C3	C	0.85250	0.25000	0.89750
2446	C3	C	0.60250	0.10250	0.50000
2447	C3	C	0.60250	0.35250	0.75000
2448	C3	C	0.85250	0.10250	0.75000
2449	C3	C	0.85250	0.35250	0.50000
2450	C3	C	0.60250	0.39750	0.50000
2451	C3	C	0.60250	0.14750	0.75000
2452	C3	C	0.85250	0.39750	0.75000
2453	C3	C	0.85250	0.14750	0.50000
2454	C3	C	0.89750	0.39750	0.50000
2455	C3	C	0.89750	0.14750	0.75000
2456	C3	C	0.64750	0.39750	0.75000
2457	C3	C	0.64750	0.14750	0.50000
2458	C3	C	0.89750	0.10250	0.50000
2459	C3	C	0.89750	0.35250	0.75000
2460	C3	C	0.64750	0.10250	0.75000
2461	C3	C	0.64750	0.35250	0.50000
2462	C3	C	0.50000	0.10250	0.60250
2463	C3	C	0.50000	0.35250	0.85250
2464	C3	C	0.75000	0.10250	0.85250
2465	C3	C	0.75000	0.35250	0.60250
2466	C3	C	0.50000	0.10250	0.89750
2467	C3	C	0.50000	0.35250	0.64750
2468	C3	C	0.75000	0.10250	0.64750
2469	C3	C	0.75000	0.35250	0.89750
2470	C3	C	0.50000	0.39750	0.89750
2471	C3	C	0.50000	0.14750	0.64750
2472	C3	C	0.75000	0.39750	0.64750
2473	C3	C	0.75000	0.14750	0.89750
2474	C3	C	0.50000	0.39750	0.60250
2475	C3	C	0.50000	0.14750	0.85250
2476	C3	C	0.75000	0.39750	0.85250
2477	C3	C	0.75000	0.14750	0.60250
2478	H1	H	0.63926	0.49997	0.56655
2479	H1	H	0.63926	0.24997	0.81655
2480	H1	H	0.88926	0.49997	0.81655
2481	H1	H	0.88926	0.24997	0.56655
2482	H1	H	0.86074	0.00003	0.56655
2483	H1	H	0.86074	0.25004	0.81655
2484	H1	H	0.61074	0.00003	0.81655

2485	H1	H	0.61074	0.25004	0.56655
2486	H1	H	0.86074	0.49996	0.93345
2487	H1	H	0.86074	0.24997	0.68345
2488	H1	H	0.61074	0.49997	0.68345
2489	H1	H	0.61074	0.24997	0.93346
2490	H1	H	0.63926	0.00003	0.93345
2491	H1	H	0.63926	0.25003	0.68346
2492	H1	H	0.88926	0.00003	0.68345
2493	H1	H	0.88926	0.25004	0.93346
2494	H1	H	0.56655	0.13926	0.99996
2495	H1	H	0.56655	0.38926	0.74997
2496	H1	H	0.81655	0.13926	0.74996
2497	H1	H	0.81654	0.38926	0.99997
2498	H1	H	0.56655	0.36074	0.50003
2499	H1	H	0.56655	0.11074	0.75004
2500	H1	H	0.81655	0.36074	0.75003
2501	H1	H	0.81655	0.11074	0.50004
2502	H1	H	0.93345	0.36074	0.99996
2503	H1	H	0.93345	0.11074	0.74997
2504	H1	H	0.68345	0.36074	0.74996
2505	H1	H	0.68345	0.11074	0.99997
2506	H1	H	0.93345	0.13926	0.50003
2507	H1	H	0.93345	0.38926	0.75004
2508	H1	H	0.68345	0.13926	0.75003
2509	H1	H	0.68345	0.38926	0.50004
2510	H1	H	0.99997	0.06655	0.63926
2511	H1	H	0.99996	0.31655	0.88926
2512	H1	H	0.74997	0.06655	0.88926
2513	H1	H	0.74996	0.31655	0.63926
2514	H1	H	0.50003	0.06655	0.86074
2515	H1	H	0.50003	0.31655	0.61074
2516	H1	H	0.75003	0.06655	0.61074
2517	H1	H	0.75004	0.31655	0.86074
2518	H1	H	0.99997	0.43345	0.86074
2519	H1	H	0.99997	0.18345	0.61074
2520	H1	H	0.74997	0.43345	0.61074
2521	H1	H	0.74997	0.18345	0.86074
2522	H1	H	0.50003	0.43345	0.63926
2523	H1	H	0.50003	0.18345	0.88926
2524	H1	H	0.75004	0.43345	0.88926
2525	H1	H	0.75003	0.18346	0.63926
2526	H1	H	0.50003	0.13926	0.93345
2527	H1	H	0.50003	0.38926	0.68345
2528	H1	H	0.75003	0.13926	0.68345
2529	H1	H	0.75004	0.38926	0.93345
2530	H1	H	0.99996	0.36074	0.93345
2531	H1	H	0.99997	0.11074	0.68345
2532	H1	H	0.74997	0.36074	0.68345
2533	H1	H	0.74997	0.11074	0.93345
2534	H1	H	0.50003	0.36074	0.56655
2535	H1	H	0.50003	0.11074	0.81655
2536	H1	H	0.75004	0.36074	0.81655
2537	H1	H	0.75003	0.11074	0.56654
2538	H1	H	0.99997	0.13926	0.56655

2539	H1	H	0.99997	0.38926	0.81655
2540	H1	H	0.74997	0.13926	0.81655
2541	H1	H	0.74997	0.38926	0.56654
2542	H1	H	0.63926	0.06655	0.99997
2543	H1	H	0.63926	0.31655	0.74996
2544	H1	H	0.88926	0.06655	0.74997
2545	H1	H	0.88926	0.31655	0.99996
2546	H1	H	0.86074	0.06655	0.50003
2547	H1	H	0.86074	0.31655	0.75003
2548	H1	H	0.61074	0.06655	0.75003
2549	H1	H	0.61074	0.31655	0.50003
2550	H1	H	0.86074	0.43345	0.99997
2551	H1	H	0.86074	0.18345	0.74997
2552	H1	H	0.61074	0.43345	0.74997
2553	H1	H	0.61074	0.18345	0.99996
2554	H1	H	0.63926	0.43345	0.50004
2555	H1	H	0.63926	0.18346	0.75003
2556	H1	H	0.88926	0.43345	0.75004
2557	H1	H	0.88926	0.18346	0.50003
2558	H1	H	0.56655	0.00003	0.86074
2559	H1	H	0.56655	0.25004	0.61074
2560	H1	H	0.81655	0.00003	0.61074
2561	H1	H	0.81655	0.25004	0.86074
2562	H1	H	0.56655	0.49996	0.63926
2563	H1	H	0.56655	0.24997	0.88926
2564	H1	H	0.81655	0.49996	0.88926
2565	H1	H	0.81655	0.24997	0.63926
2566	H1	H	0.93345	0.00003	0.63926
2567	H1	H	0.93345	0.25004	0.88926
2568	H1	H	0.68345	0.00003	0.88926
2569	H1	H	0.68345	0.25003	0.63926
2570	H1	H	0.93345	0.49996	0.86074
2571	H1	H	0.93345	0.24997	0.61074
2572	H1	H	0.68345	0.49996	0.61074
2573	H1	H	0.68345	0.24997	0.86074
2574	H2	H	0.70825	0.29176	0.95824
2575	H2	H	0.79174	0.20825	0.95823
2576	H2	H	0.70827	0.20823	0.54177
2577	H2	H	0.79179	0.29168	0.54178
2578	H2	H	0.95823	0.29173	0.70823
2579	H2	H	0.95824	0.20825	0.79176
2580	H2	H	0.54174	0.29176	0.79177
2581	H2	H	0.54173	0.20823	0.70823
2582	H2	H	0.79175	0.45824	0.70824
2583	H2	H	0.70824	0.45824	0.79176
2584	H2	H	0.70823	0.04172	0.70822
2585	H2	H	0.79177	0.04173	0.79178
2586	H2	H	0.54178	0.04177	0.54173
2587	H2	H	0.95823	0.45824	0.54174
2588	H2	H	0.95823	0.04176	0.95825
2589	H2	H	0.54176	0.45824	0.95824
2590	O1	O	0.58524	0.00000	0.54765
2591	O1	O	0.58524	0.25000	0.79765
2592	O1	O	0.83524	0.00000	0.79765

2593	O1	O	0.83524	0.25000	0.54765
2594	O1	O	0.91476	0.00000	0.54765
2595	O1	O	0.91476	0.25000	0.79765
2596	O1	O	0.66476	0.00000	0.79765
2597	O1	O	0.66476	0.25000	0.54765
2598	O1	O	0.91476	0.00000	0.95235
2599	O1	O	0.91476	0.25000	0.70235
2600	O1	O	0.66476	0.00000	0.70235
2601	O1	O	0.66476	0.25000	0.95235
2602	O1	O	0.58524	0.00000	0.95235
2603	O1	O	0.58524	0.25000	0.70235
2604	O1	O	0.83524	0.00000	0.70235
2605	O1	O	0.83524	0.25000	0.95235
2606	O1	O	0.54765	0.08524	0.50000
2607	O1	O	0.54765	0.33524	0.75000
2608	O1	O	0.79765	0.08524	0.75000
2609	O1	O	0.79765	0.33524	0.50000
2610	O1	O	0.54765	0.41476	0.50000
2611	O1	O	0.54765	0.16476	0.75000
2612	O1	O	0.79765	0.41476	0.75000
2613	O1	O	0.79765	0.16476	0.50000
2614	O1	O	0.95235	0.41476	0.50000
2615	O1	O	0.95235	0.16476	0.75000
2616	O1	O	0.70235	0.41476	0.75000
2617	O1	O	0.70235	0.16476	0.50000
2618	O1	O	0.95235	0.08524	0.50000
2619	O1	O	0.95235	0.33524	0.75000
2620	O1	O	0.70235	0.08524	0.75000
2621	O1	O	0.70235	0.33524	0.50000
2622	O1	O	0.50000	0.04765	0.58524
2623	O1	O	0.50000	0.29765	0.83524
2624	O1	O	0.75000	0.04765	0.83524
2625	O1	O	0.75000	0.29765	0.58524
2626	O1	O	0.50000	0.04765	0.91476
2627	O1	O	0.50000	0.29765	0.66476
2628	O1	O	0.75000	0.04765	0.66476
2629	O1	O	0.75000	0.29765	0.91476
2630	O1	O	0.50000	0.45235	0.91476
2631	O1	O	0.50000	0.20235	0.66476
2632	O1	O	0.75000	0.45235	0.66476
2633	O1	O	0.75000	0.20235	0.91476
2634	O1	O	0.50000	0.45235	0.58524
2635	O1	O	0.50000	0.20235	0.83524
2636	O1	O	0.75000	0.45235	0.83524
2637	O1	O	0.75000	0.20235	0.58524
2638	O1	O	0.50000	0.08524	0.95235
2639	O1	O	0.50000	0.33524	0.70235
2640	O1	O	0.75000	0.08524	0.70235
2641	O1	O	0.75000	0.33524	0.95235
2642	O1	O	0.50000	0.41476	0.95235
2643	O1	O	0.50000	0.16476	0.70235
2644	O1	O	0.75000	0.41476	0.70235
2645	O1	O	0.75000	0.16476	0.95235
2646	O1	O	0.50000	0.41476	0.54765

2647	O1	O	0.50000	0.16476	0.79765
2648	O1	O	0.75000	0.41476	0.79765
2649	O1	O	0.75000	0.16476	0.54765
2650	O1	O	0.50000	0.08524	0.54765
2651	O1	O	0.50000	0.33524	0.79765
2652	O1	O	0.75000	0.08524	0.79765
2653	O1	O	0.75000	0.33524	0.54765
2654	O1	O	0.58524	0.04765	0.50000
2655	O1	O	0.58524	0.29765	0.75000
2656	O1	O	0.83524	0.04765	0.75000
2657	O1	O	0.83524	0.29765	0.50000
2658	O1	O	0.91476	0.04765	0.50000
2659	O1	O	0.91476	0.29765	0.75000
2660	O1	O	0.66476	0.04765	0.75000
2661	O1	O	0.66476	0.29765	0.50000
2662	O1	O	0.91476	0.45235	0.50000
2663	O1	O	0.91476	0.20235	0.75000
2664	O1	O	0.66476	0.45235	0.75000
2665	O1	O	0.66476	0.20235	0.50000
2666	O1	O	0.58524	0.45235	0.50000
2667	O1	O	0.58524	0.20235	0.75000
2668	O1	O	0.83524	0.45235	0.75000
2669	O1	O	0.83524	0.20235	0.50000
2670	O1	O	0.54765	0.00000	0.91476
2671	O1	O	0.54765	0.25000	0.66476
2672	O1	O	0.79765	0.00000	0.66476
2673	O1	O	0.79765	0.25000	0.91476
2674	O1	O	0.54765	0.00000	0.58524
2675	O1	O	0.54765	0.25000	0.83524
2676	O1	O	0.79765	0.00000	0.83524
2677	O1	O	0.79765	0.25000	0.58524
2678	O1	O	0.95235	0.00000	0.58524
2679	O1	O	0.95235	0.25000	0.83524
2680	O1	O	0.70235	0.00000	0.83524
2681	O1	O	0.70235	0.25000	0.58524
2682	O1	O	0.95235	0.00000	0.91476
2683	O1	O	0.95235	0.25000	0.66476
2684	O1	O	0.70235	0.00000	0.66476
2685	O1	O	0.70235	0.25000	0.91476
2686	O2	O	0.52805	0.47195	0.97195
2687	O2	O	0.52805	0.22195	0.72195
2688	O2	O	0.77805	0.47195	0.72195
2689	O2	O	0.77805	0.22195	0.97195
2690	O2	O	0.97195	0.02805	0.97195
2691	O2	O	0.97195	0.27805	0.72195
2692	O2	O	0.72195	0.02805	0.72195
2693	O2	O	0.72195	0.27805	0.97195
2694	O2	O	0.97195	0.47195	0.52805
2695	O2	O	0.97195	0.22195	0.77805
2696	O2	O	0.72195	0.47195	0.77805
2697	O2	O	0.72195	0.22195	0.52805
2698	O2	O	0.52805	0.02805	0.52805
2699	O2	O	0.52805	0.27805	0.77805
2700	O2	O	0.77805	0.02805	0.77805

2701	O2	O	0.77805	0.27805	0.52805
2702	O1	O	0.97195	0.02805	0.52805
2703	O1	O	0.97195	0.27805	0.77805
2704	O1	O	0.72195	0.02805	0.77805
2705	O1	O	0.72195	0.27805	0.52805
2706	O1	O	0.52805	0.47195	0.52805
2707	O1	O	0.52805	0.22195	0.77805
2708	O1	O	0.77805	0.47195	0.77805
2709	O1	O	0.77805	0.22195	0.52805
2710	O1	O	0.97195	0.47195	0.97195
2711	O1	O	0.97195	0.22195	0.72195
2712	O1	O	0.72195	0.47195	0.72195
2713	O1	O	0.72195	0.22195	0.97195
2714	O1	O	0.52805	0.02805	0.97195
2715	O1	O	0.52805	0.27805	0.72195
2716	O1	O	0.77805	0.02805	0.72195
2717	O1	O	0.77805	0.27805	0.97195
2718	Zr1	Zr	0.55994	0.00000	0.50000
2719	Zr1	Zr	0.55994	0.25000	0.75000
2720	Zr1	Zr	0.80994	0.00000	0.75000
2721	Zr1	Zr	0.80994	0.25000	0.50000
2722	Zr1	Zr	0.94006	0.00000	0.50000
2723	Zr1	Zr	0.94006	0.25000	0.75000
2724	Zr1	Zr	0.69006	0.00000	0.75000
2725	Zr1	Zr	0.69006	0.25000	0.50000
2726	Zr1	Zr	0.50000	0.05994	0.50000
2727	Zr1	Zr	0.50000	0.30994	0.75000
2728	Zr1	Zr	0.75000	0.05994	0.75000
2729	Zr1	Zr	0.75000	0.30994	0.50000
2730	Zr1	Zr	0.50000	0.44006	0.50000
2731	Zr1	Zr	0.50000	0.19006	0.75000
2732	Zr1	Zr	0.75000	0.44006	0.75000
2733	Zr1	Zr	0.75000	0.19006	0.50000
2734	Zr1	Zr	0.50000	0.00000	0.55994
2735	Zr1	Zr	0.50000	0.25000	0.80994
2736	Zr1	Zr	0.75000	0.00000	0.80994
2737	Zr1	Zr	0.75000	0.25000	0.55994
2738	Zr1	Zr	0.50000	0.00000	0.94006
2739	Zr1	Zr	0.50000	0.25000	0.69006
2740	Zr1	Zr	0.75000	0.00000	0.69006
2741	Zr1	Zr	0.75000	0.25000	0.94006
2742	C2	C	0.63375	0.50000	0.09220
2743	C2	C	0.63375	0.75000	0.34220
2744	C2	C	0.88375	0.50000	0.34220
2745	C2	C	0.88375	0.75000	0.09220
2746	C2	C	0.86625	0.50000	0.09220
2747	C2	C	0.86625	0.75000	0.34220
2748	C2	C	0.61625	0.50000	0.34220
2749	C2	C	0.61625	0.75000	0.09220
2750	C2	C	0.86625	0.50000	0.40780
2751	C2	C	0.86625	0.75000	0.15780
2752	C2	C	0.61625	0.50000	0.15780
2753	C2	C	0.61625	0.75000	0.40780
2754	C2	C	0.63375	0.50000	0.40780

2755	C2	C	0.63375	0.75000	0.15780
2756	C2	C	0.88375	0.50000	0.15780
2757	C2	C	0.88375	0.75000	0.40780
2758	C2	C	0.59220	0.63375	0.00000
2759	C2	C	0.59220	0.88375	0.25000
2760	C2	C	0.84220	0.63375	0.25000
2761	C2	C	0.84220	0.88375	0.00000
2762	C2	C	0.59220	0.86625	0.00000
2763	C2	C	0.59220	0.61625	0.25000
2764	C2	C	0.84220	0.86625	0.25000
2765	C2	C	0.84220	0.61625	0.00000
2766	C2	C	0.90780	0.86625	0.00000
2767	C2	C	0.90780	0.61625	0.25000
2768	C2	C	0.65780	0.86625	0.25000
2769	C2	C	0.65780	0.61625	0.00000
2770	C2	C	0.90780	0.63375	0.00000
2771	C2	C	0.90780	0.88375	0.25000
2772	C2	C	0.65780	0.63375	0.25000
2773	C2	C	0.65780	0.88375	0.00000
2774	C2	C	0.50000	0.59220	0.13375
2775	C2	C	0.50000	0.84220	0.38375
2776	C2	C	0.75000	0.59220	0.38375
2777	C2	C	0.75000	0.84220	0.13375
2778	C2	C	0.50000	0.59220	0.36625
2779	C2	C	0.50000	0.84220	0.11625
2780	C2	C	0.75000	0.59220	0.11625
2781	C2	C	0.75000	0.84220	0.36625
2782	C2	C	0.50000	0.90780	0.36625
2783	C2	C	0.50000	0.65780	0.11625
2784	C2	C	0.75000	0.90780	0.11625
2785	C2	C	0.75000	0.65780	0.36625
2786	C2	C	0.50000	0.90780	0.13375
2787	C2	C	0.50000	0.65780	0.38375
2788	C2	C	0.75000	0.90780	0.38375
2789	C2	C	0.75000	0.65780	0.13375
2790	C2	C	0.50000	0.63375	0.40780
2791	C2	C	0.50000	0.88375	0.15780
2792	C2	C	0.75000	0.63375	0.15780
2793	C2	C	0.75000	0.88375	0.40780
2794	C2	C	0.50000	0.86625	0.40780
2795	C2	C	0.50000	0.61625	0.15780
2796	C2	C	0.75000	0.86625	0.15780
2797	C2	C	0.75000	0.61625	0.40780
2798	C2	C	0.50000	0.86625	0.09220
2799	C2	C	0.50000	0.61625	0.34220
2800	C2	C	0.75000	0.86625	0.34220
2801	C2	C	0.75000	0.61625	0.09220
2802	C2	C	0.50000	0.63375	0.09220
2803	C2	C	0.50000	0.88375	0.34220
2804	C2	C	0.75000	0.63375	0.34220
2805	C2	C	0.75000	0.88375	0.09220
2806	C2	C	0.63375	0.59220	0.00000
2807	C2	C	0.63375	0.84220	0.25000
2808	C2	C	0.88375	0.59220	0.25000

2809	C2	C	0.88375	0.84220	0.00000
2810	C2	C	0.86625	0.59220	0.00000
2811	C2	C	0.86625	0.84220	0.25000
2812	C2	C	0.61625	0.59220	0.25000
2813	C2	C	0.61625	0.84220	0.00000
2814	C2	C	0.86625	0.90780	0.00000
2815	C2	C	0.86625	0.65780	0.25000
2816	C2	C	0.61625	0.90780	0.25000
2817	C2	C	0.61625	0.65780	0.00000
2818	C2	C	0.63375	0.90780	0.00000
2819	C2	C	0.63375	0.65780	0.25000
2820	C2	C	0.88375	0.90780	0.25000
2821	C2	C	0.88375	0.65780	0.00000
2822	C2	C	0.59220	0.50000	0.36625
2823	C2	C	0.59220	0.75000	0.11625
2824	C2	C	0.84220	0.50000	0.11625
2825	C2	C	0.84220	0.75000	0.36625
2826	C2	C	0.59220	0.50000	0.13375
2827	C2	C	0.59220	0.75000	0.38375
2828	C2	C	0.84220	0.50000	0.38375
2829	C2	C	0.84220	0.75000	0.13375
2830	C2	C	0.90780	0.50000	0.13375
2831	C2	C	0.90780	0.75000	0.38375
2832	C2	C	0.65780	0.50000	0.38375
2833	C2	C	0.65780	0.75000	0.13375
2834	C2	C	0.90780	0.50000	0.36625
2835	C2	C	0.90780	0.75000	0.11625
2836	C2	C	0.65780	0.50000	0.11625
2837	C2	C	0.65780	0.75000	0.36625
2838	C1	C	0.57680	0.50000	0.42320
2839	C1	C	0.57680	0.75000	0.17320
2840	C1	C	0.82680	0.50000	0.17320
2841	C1	C	0.82680	0.75000	0.42320
2842	C1	C	0.92320	0.50000	0.42320
2843	C1	C	0.92320	0.75000	0.17320
2844	C1	C	0.67320	0.50000	0.17320
2845	C1	C	0.67320	0.75000	0.42320
2846	C1	C	0.92320	0.50000	0.07680
2847	C1	C	0.92320	0.75000	0.32680
2848	C1	C	0.67320	0.50000	0.32680
2849	C1	C	0.67320	0.75000	0.07680
2850	C1	C	0.57680	0.50000	0.07680
2851	C1	C	0.57680	0.75000	0.32680
2852	C1	C	0.82680	0.50000	0.32680
2853	C1	C	0.82680	0.75000	0.07680
2854	C1	C	0.92320	0.57680	0.00000
2855	C1	C	0.92320	0.82680	0.25000
2856	C1	C	0.67320	0.57680	0.25000
2857	C1	C	0.67320	0.82680	0.00000
2858	C1	C	0.92320	0.92320	0.00000
2859	C1	C	0.92320	0.67320	0.25000
2860	C1	C	0.67320	0.92320	0.25000
2861	C1	C	0.67320	0.67320	0.00000
2862	C1	C	0.57680	0.92320	0.00000

2863	C1	C	0.57680	0.67320	0.25000
2864	C1	C	0.82680	0.92320	0.25000
2865	C1	C	0.82680	0.67320	0.00000
2866	C1	C	0.57680	0.57680	0.00000
2867	C1	C	0.57680	0.82680	0.25000
2868	C1	C	0.82680	0.57680	0.25000
2869	C1	C	0.82680	0.82680	0.00000
2870	C1	C	0.50000	0.92320	0.07680
2871	C1	C	0.50000	0.67320	0.32680
2872	C1	C	0.75000	0.92320	0.32680
2873	C1	C	0.75000	0.67320	0.07680
2874	C1	C	0.50000	0.92320	0.42320
2875	C1	C	0.50000	0.67320	0.17320
2876	C1	C	0.75000	0.92320	0.17320
2877	C1	C	0.75000	0.67320	0.42320
2878	C1	C	0.50000	0.57680	0.42320
2879	C1	C	0.50000	0.82680	0.17320
2880	C1	C	0.75000	0.57680	0.17320
2881	C1	C	0.75000	0.82680	0.42320
2882	C1	C	0.50000	0.57680	0.07680
2883	C1	C	0.50000	0.82680	0.32680
2884	C1	C	0.75000	0.57680	0.32680
2885	C1	C	0.75000	0.82680	0.07680
2886	C3	C	0.60250	0.50000	0.10250
2887	C3	C	0.60250	0.75000	0.35250
2888	C3	C	0.85250	0.50000	0.35250
2889	C3	C	0.85250	0.75000	0.10250
2890	C3	C	0.89750	0.50000	0.10250
2891	C3	C	0.89750	0.75000	0.35250
2892	C3	C	0.64750	0.50000	0.35250
2893	C3	C	0.64750	0.75000	0.10250
2894	C3	C	0.89750	0.50000	0.39750
2895	C3	C	0.89750	0.75000	0.14750
2896	C3	C	0.64750	0.50000	0.14750
2897	C3	C	0.64750	0.75000	0.39750
2898	C3	C	0.60250	0.50000	0.39750
2899	C3	C	0.60250	0.75000	0.14750
2900	C3	C	0.85250	0.50000	0.14750
2901	C3	C	0.85250	0.75000	0.39750
2902	C3	C	0.60250	0.60250	0.00000
2903	C3	C	0.60250	0.85250	0.25000
2904	C3	C	0.85250	0.60250	0.25000
2905	C3	C	0.85250	0.85250	0.00000
2906	C3	C	0.60250	0.89750	0.00000
2907	C3	C	0.60250	0.64750	0.25000
2908	C3	C	0.85250	0.89750	0.25000
2909	C3	C	0.85250	0.64750	0.00000
2910	C3	C	0.89750	0.89750	0.00000
2911	C3	C	0.89750	0.64750	0.25000
2912	C3	C	0.64750	0.89750	0.25000
2913	C3	C	0.64750	0.64750	0.00000
2914	C3	C	0.89750	0.60250	0.00000
2915	C3	C	0.89750	0.85250	0.25000
2916	C3	C	0.64750	0.60250	0.25000

2917	C3	C	0.64750	0.85250	0.00000
2918	C3	C	0.50000	0.60250	0.10250
2919	C3	C	0.50000	0.85250	0.35250
2920	C3	C	0.75000	0.60250	0.35250
2921	C3	C	0.75000	0.85250	0.10250
2922	C3	C	0.50000	0.60250	0.39750
2923	C3	C	0.50000	0.85250	0.14750
2924	C3	C	0.75000	0.60250	0.14750
2925	C3	C	0.75000	0.85250	0.39750
2926	C3	C	0.50000	0.89750	0.39750
2927	C3	C	0.50000	0.64750	0.14750
2928	C3	C	0.75000	0.89750	0.14750
2929	C3	C	0.75000	0.64750	0.39750
2930	C3	C	0.50000	0.89750	0.10250
2931	C3	C	0.50000	0.64750	0.35250
2932	C3	C	0.75000	0.89750	0.35250
2933	C3	C	0.75000	0.64750	0.10250
2934	H1	H	0.63926	0.99997	0.06655
2935	H1	H	0.63926	0.74997	0.31655
2936	H1	H	0.88926	0.99997	0.31655
2937	H1	H	0.88926	0.74997	0.06655
2938	H1	H	0.86074	0.50003	0.06655
2939	H1	H	0.86074	0.75004	0.31655
2940	H1	H	0.61074	0.50003	0.31655
2941	H1	H	0.61074	0.75004	0.06655
2942	H1	H	0.86074	0.99996	0.43345
2943	H1	H	0.86074	0.74997	0.18345
2944	H1	H	0.61074	0.99997	0.18345
2945	H1	H	0.61074	0.74997	0.43346
2946	H1	H	0.63926	0.50003	0.43345
2947	H1	H	0.63926	0.75003	0.18346
2948	H1	H	0.88926	0.50003	0.18345
2949	H1	H	0.88926	0.75004	0.43346
2950	H1	H	0.56655	0.63926	0.49996
2951	H1	H	0.56655	0.88926	0.24997
2952	H1	H	0.81655	0.63926	0.24996
2953	H1	H	0.81654	0.88926	0.49997
2954	H1	H	0.56655	0.86074	0.00003
2955	H1	H	0.56655	0.61074	0.25004
2956	H1	H	0.81655	0.86074	0.25003
2957	H1	H	0.81655	0.61074	0.00004
2958	H1	H	0.93345	0.86074	0.49996
2959	H1	H	0.93345	0.61074	0.24997
2960	H1	H	0.68345	0.86074	0.24996
2961	H1	H	0.68345	0.61074	0.49997
2962	H1	H	0.93345	0.63926	0.00003
2963	H1	H	0.93345	0.88926	0.25004
2964	H1	H	0.68345	0.63926	0.25003
2965	H1	H	0.68345	0.88926	0.00004
2966	H1	H	0.99997	0.56655	0.13926
2967	H1	H	0.99996	0.81655	0.38926
2968	H1	H	0.74997	0.56655	0.38926
2969	H1	H	0.74996	0.81655	0.13926
2970	H1	H	0.50003	0.56655	0.36074

2971	H1	H	0.50003	0.81655	0.11074
2972	H1	H	0.75003	0.56655	0.11074
2973	H1	H	0.75004	0.81655	0.36074
2974	H1	H	0.99997	0.93345	0.36074
2975	H1	H	0.99997	0.68345	0.11074
2976	H1	H	0.74997	0.93345	0.11074
2977	H1	H	0.74997	0.68345	0.36074
2978	H1	H	0.50003	0.93345	0.13926
2979	H1	H	0.50003	0.68345	0.38926
2980	H1	H	0.75004	0.93345	0.38926
2981	H1	H	0.75003	0.68346	0.13926
2982	H1	H	0.50003	0.63926	0.43345
2983	H1	H	0.50003	0.88926	0.18345
2984	H1	H	0.75003	0.63926	0.18345
2985	H1	H	0.75004	0.88926	0.43345
2986	H1	H	0.99996	0.86074	0.43345
2987	H1	H	0.99997	0.61074	0.18345
2988	H1	H	0.74997	0.86074	0.18345
2989	H1	H	0.74997	0.61074	0.43345
2990	H1	H	0.50003	0.86074	0.06655
2991	H1	H	0.50003	0.61074	0.31655
2992	H1	H	0.75004	0.86074	0.31655
2993	H1	H	0.75003	0.61074	0.06654
2994	H1	H	0.99997	0.63926	0.06655
2995	H1	H	0.99997	0.88926	0.31655
2996	H1	H	0.74997	0.63926	0.31655
2997	H1	H	0.74997	0.88926	0.06654
2998	H1	H	0.63926	0.56655	0.49997
2999	H1	H	0.63926	0.81655	0.24996
3000	H1	H	0.88926	0.56655	0.24997
3001	H1	H	0.88926	0.81655	0.49996
3002	H1	H	0.86074	0.56655	0.00003
3003	H1	H	0.86074	0.81655	0.25003
3004	H1	H	0.61074	0.56655	0.25003
3005	H1	H	0.61074	0.81655	0.00003
3006	H1	H	0.86074	0.93345	0.49997
3007	H1	H	0.86074	0.68345	0.24997
3008	H1	H	0.61074	0.93345	0.24997
3009	H1	H	0.61074	0.68345	0.49996
3010	H1	H	0.63926	0.93345	0.00004
3011	H1	H	0.63926	0.68346	0.25003
3012	H1	H	0.88926	0.93345	0.25004
3013	H1	H	0.88926	0.68346	0.00003
3014	H1	H	0.56655	0.50003	0.36074
3015	H1	H	0.56655	0.75004	0.11074
3016	H1	H	0.81655	0.50003	0.11074
3017	H1	H	0.81655	0.75004	0.36074
3018	H1	H	0.56655	0.99996	0.13926
3019	H1	H	0.56655	0.74997	0.38926
3020	H1	H	0.81655	0.99996	0.38926
3021	H1	H	0.81655	0.74997	0.13926
3022	H1	H	0.93345	0.50003	0.13926
3023	H1	H	0.93345	0.75004	0.38926
3024	H1	H	0.68345	0.50003	0.38926

3025	H1	H	0.68345	0.75003	0.13926
3026	H1	H	0.93345	0.99996	0.36074
3027	H1	H	0.93345	0.74997	0.11074
3028	H1	H	0.68345	0.99996	0.11074
3029	H1	H	0.68345	0.74997	0.36074
3030	H2	H	0.70825	0.79176	0.45824
3031	H2	H	0.79174	0.70825	0.45823
3032	H2	H	0.70827	0.70823	0.04177
3033	H2	H	0.79179	0.79168	0.04178
3034	H2	H	0.95823	0.79173	0.20823
3035	H2	H	0.95824	0.70825	0.29176
3036	H2	H	0.54174	0.79176	0.29177
3037	H2	H	0.54173	0.70823	0.20823
3038	H2	H	0.79175	0.95824	0.20824
3039	H2	H	0.70824	0.95824	0.29176
3040	H2	H	0.70823	0.54172	0.20822
3041	H2	H	0.79177	0.54173	0.29178
3042	H2	H	0.54178	0.54177	0.04173
3043	H2	H	0.95823	0.95824	0.04174
3044	H2	H	0.95823	0.54176	0.45825
3045	H2	H	0.54176	0.95824	0.45824
3046	O1	O	0.58524	0.50000	0.04765
3047	O1	O	0.58524	0.75000	0.29765
3048	O1	O	0.83524	0.50000	0.29765
3049	O1	O	0.83524	0.75000	0.04765
3050	O1	O	0.91476	0.50000	0.04765
3051	O1	O	0.91476	0.75000	0.29765
3052	O1	O	0.66476	0.50000	0.29765
3053	O1	O	0.66476	0.75000	0.04765
3054	O1	O	0.91476	0.50000	0.45235
3055	O1	O	0.91476	0.75000	0.20235
3056	O1	O	0.66476	0.50000	0.20235
3057	O1	O	0.66476	0.75000	0.45235
3058	O1	O	0.58524	0.50000	0.45235
3059	O1	O	0.58524	0.75000	0.20235
3060	O1	O	0.83524	0.50000	0.20235
3061	O1	O	0.83524	0.75000	0.45235
3062	O1	O	0.54765	0.58524	0.00000
3063	O1	O	0.54765	0.83524	0.25000
3064	O1	O	0.79765	0.58524	0.25000
3065	O1	O	0.79765	0.83524	0.00000
3066	O1	O	0.54765	0.91476	0.00000
3067	O1	O	0.54765	0.66476	0.25000
3068	O1	O	0.79765	0.91476	0.25000
3069	O1	O	0.79765	0.66476	0.00000
3070	O1	O	0.95235	0.91476	0.00000
3071	O1	O	0.95235	0.66476	0.25000
3072	O1	O	0.70235	0.91476	0.25000
3073	O1	O	0.70235	0.66476	0.00000
3074	O1	O	0.95235	0.58524	0.00000
3075	O1	O	0.95235	0.83524	0.25000
3076	O1	O	0.70235	0.58524	0.25000
3077	O1	O	0.70235	0.83524	0.00000
3078	O1	O	0.50000	0.54765	0.08524

3079	O1	O	0.50000	0.79765	0.33524
3080	O1	O	0.75000	0.54765	0.33524
3081	O1	O	0.75000	0.79765	0.08524
3082	O1	O	0.50000	0.54765	0.41476
3083	O1	O	0.50000	0.79765	0.16476
3084	O1	O	0.75000	0.54765	0.16476
3085	O1	O	0.75000	0.79765	0.41476
3086	O1	O	0.50000	0.95235	0.41476
3087	O1	O	0.50000	0.70235	0.16476
3088	O1	O	0.75000	0.95235	0.16476
3089	O1	O	0.75000	0.70235	0.41476
3090	O1	O	0.50000	0.95235	0.08524
3091	O1	O	0.50000	0.70235	0.33524
3092	O1	O	0.75000	0.95235	0.33524
3093	O1	O	0.75000	0.70235	0.08524
3094	O1	O	0.50000	0.58524	0.45235
3095	O1	O	0.50000	0.83524	0.20235
3096	O1	O	0.75000	0.58524	0.20235
3097	O1	O	0.75000	0.83524	0.45235
3098	O1	O	0.50000	0.91476	0.45235
3099	O1	O	0.50000	0.66476	0.20235
3100	O1	O	0.75000	0.91476	0.20235
3101	O1	O	0.75000	0.66476	0.45235
3102	O1	O	0.50000	0.91476	0.04765
3103	O1	O	0.50000	0.66476	0.29765
3104	O1	O	0.75000	0.91476	0.29765
3105	O1	O	0.75000	0.66476	0.04765
3106	O1	O	0.50000	0.58524	0.04765
3107	O1	O	0.50000	0.83524	0.29765
3108	O1	O	0.75000	0.58524	0.29765
3109	O1	O	0.75000	0.83524	0.04765
3110	O1	O	0.58524	0.54765	0.00000
3111	O1	O	0.58524	0.79765	0.25000
3112	O1	O	0.83524	0.54765	0.25000
3113	O1	O	0.83524	0.79765	0.00000
3114	O1	O	0.91476	0.54765	0.00000
3115	O1	O	0.91476	0.79765	0.25000
3116	O1	O	0.66476	0.54765	0.25000
3117	O1	O	0.66476	0.79765	0.00000
3118	O1	O	0.91476	0.95235	0.00000
3119	O1	O	0.91476	0.70235	0.25000
3120	O1	O	0.66476	0.95235	0.25000
3121	O1	O	0.66476	0.70235	0.00000
3122	O1	O	0.58524	0.95235	0.00000
3123	O1	O	0.58524	0.70235	0.25000
3124	O1	O	0.83524	0.95235	0.25000
3125	O1	O	0.83524	0.70235	0.00000
3126	O1	O	0.54765	0.50000	0.41476
3127	O1	O	0.54765	0.75000	0.16476
3128	O1	O	0.79765	0.50000	0.16476
3129	O1	O	0.79765	0.75000	0.41476
3130	O1	O	0.54765	0.50000	0.08524
3131	O1	O	0.54765	0.75000	0.33524
3132	O1	O	0.79765	0.50000	0.33524

3133	O1	O	0.79765	0.75000	0.08524
3134	O1	O	0.95235	0.50000	0.08524
3135	O1	O	0.95235	0.75000	0.33524
3136	O1	O	0.70235	0.50000	0.33524
3137	O1	O	0.70235	0.75000	0.08524
3138	O1	O	0.95235	0.50000	0.41476
3139	O1	O	0.95235	0.75000	0.16476
3140	O1	O	0.70235	0.50000	0.16476
3141	O1	O	0.70235	0.75000	0.41476
3142	O2	O	0.52805	0.97195	0.47195
3143	O2	O	0.52805	0.72195	0.22195
3144	O2	O	0.77805	0.97195	0.22195
3145	O2	O	0.77805	0.72195	0.47195
3146	O2	O	0.97195	0.52805	0.47195
3147	O2	O	0.97195	0.77805	0.22195
3148	O2	O	0.72195	0.52805	0.22195
3149	O2	O	0.72195	0.77805	0.47195
3150	O2	O	0.97195	0.97195	0.02805
3151	O2	O	0.97195	0.72195	0.27805
3152	O2	O	0.72195	0.97195	0.27805
3153	O2	O	0.72195	0.72195	0.02805
3154	O2	O	0.52805	0.52805	0.02805
3155	O2	O	0.52805	0.77805	0.27805
3156	O2	O	0.77805	0.52805	0.27805
3157	O2	O	0.77805	0.77805	0.02805
3158	O1	O	0.97195	0.52805	0.02805
3159	O1	O	0.97195	0.77805	0.27805
3160	O1	O	0.72195	0.52805	0.27805
3161	O1	O	0.72195	0.77805	0.02805
3162	O1	O	0.52805	0.97195	0.02805
3163	O1	O	0.52805	0.72195	0.27805
3164	O1	O	0.77805	0.97195	0.27805
3165	O1	O	0.77805	0.72195	0.02805
3166	O1	O	0.97195	0.97195	0.47195
3167	O1	O	0.97195	0.72195	0.22195
3168	O1	O	0.72195	0.97195	0.22195
3169	O1	O	0.72195	0.72195	0.47195
3170	O1	O	0.52805	0.52805	0.47195
3171	O1	O	0.52805	0.77805	0.22195
3172	O1	O	0.77805	0.52805	0.22195
3173	O1	O	0.77805	0.77805	0.47195
3174	Zr1	Zr	0.55994	0.50000	0.00000
3175	Zr1	Zr	0.55994	0.75000	0.25000
3176	Zr1	Zr	0.80994	0.50000	0.25000
3177	Zr1	Zr	0.80994	0.75000	0.00000
3178	Zr1	Zr	0.94006	0.50000	0.00000
3179	Zr1	Zr	0.94006	0.75000	0.25000
3180	Zr1	Zr	0.69006	0.50000	0.25000
3181	Zr1	Zr	0.69006	0.75000	0.00000
3182	Zr1	Zr	0.50000	0.55994	0.00000
3183	Zr1	Zr	0.50000	0.80994	0.25000
3184	Zr1	Zr	0.75000	0.55994	0.25000
3185	Zr1	Zr	0.75000	0.80994	0.00000
3186	Zr1	Zr	0.50000	0.94006	0.00000

3187	Zr1	Zr	0.50000	0.69006	0.25000
3188	Zr1	Zr	0.75000	0.94006	0.25000
3189	Zr1	Zr	0.75000	0.69006	0.00000
3190	Zr1	Zr	0.50000	0.50000	0.05994
3191	Zr1	Zr	0.50000	0.75000	0.30994
3192	Zr1	Zr	0.75000	0.50000	0.30994
3193	Zr1	Zr	0.75000	0.75000	0.05994
3194	Zr1	Zr	0.50000	0.50000	0.44006
3195	Zr1	Zr	0.50000	0.75000	0.19006
3196	Zr1	Zr	0.75000	0.50000	0.19006
3197	Zr1	Zr	0.75000	0.75000	0.44006
3198	C2	C	0.63375	0.50000	0.59220
3199	C2	C	0.63375	0.75000	0.84220
3200	C2	C	0.88375	0.50000	0.84220
3201	C2	C	0.88375	0.75000	0.59220
3202	C2	C	0.86625	0.50000	0.59220
3203	C2	C	0.86625	0.75000	0.84220
3204	C2	C	0.61625	0.50000	0.84220
3205	C2	C	0.61625	0.75000	0.59220
3206	C2	C	0.86625	0.50000	0.90780
3207	C2	C	0.86625	0.75000	0.65780
3208	C2	C	0.61625	0.50000	0.65780
3209	C2	C	0.61625	0.75000	0.90780
3210	C2	C	0.63375	0.50000	0.90780
3211	C2	C	0.63375	0.75000	0.65780
3212	C2	C	0.88375	0.50000	0.65780
3213	C2	C	0.88375	0.75000	0.90780
3214	C2	C	0.59220	0.63375	0.50000
3215	C2	C	0.59220	0.88375	0.75000
3216	C2	C	0.84220	0.63375	0.75000
3217	C2	C	0.84220	0.88375	0.50000
3218	C2	C	0.59220	0.86625	0.50000
3219	C2	C	0.59220	0.61625	0.75000
3220	C2	C	0.84220	0.86625	0.75000
3221	C2	C	0.84220	0.61625	0.50000
3222	C2	C	0.90780	0.86625	0.50000
3223	C2	C	0.90780	0.61625	0.75000
3224	C2	C	0.65780	0.86625	0.75000
3225	C2	C	0.65780	0.61625	0.50000
3226	C2	C	0.90780	0.63375	0.50000
3227	C2	C	0.90780	0.88375	0.75000
3228	C2	C	0.65780	0.63375	0.75000
3229	C2	C	0.65780	0.88375	0.50000
3230	C2	C	0.50000	0.59220	0.63375
3231	C2	C	0.50000	0.84220	0.88375
3232	C2	C	0.75000	0.59220	0.88375
3233	C2	C	0.75000	0.84220	0.63375
3234	C2	C	0.50000	0.59220	0.86625
3235	C2	C	0.50000	0.84220	0.61625
3236	C2	C	0.75000	0.59220	0.61625
3237	C2	C	0.75000	0.84220	0.86625
3238	C2	C	0.50000	0.90780	0.86625
3239	C2	C	0.50000	0.65780	0.61625
3240	C2	C	0.75000	0.90780	0.61625

3241	C2	C	0.75000	0.65780	0.86625
3242	C2	C	0.50000	0.90780	0.63375
3243	C2	C	0.50000	0.65780	0.88375
3244	C2	C	0.75000	0.90780	0.88375
3245	C2	C	0.75000	0.65780	0.63375
3246	C2	C	0.50000	0.63375	0.90780
3247	C2	C	0.50000	0.88375	0.65780
3248	C2	C	0.75000	0.63375	0.65780
3249	C2	C	0.75000	0.88375	0.90780
3250	C2	C	0.50000	0.86625	0.90780
3251	C2	C	0.50000	0.61625	0.65780
3252	C2	C	0.75000	0.86625	0.65780
3253	C2	C	0.75000	0.61625	0.90780
3254	C2	C	0.50000	0.86625	0.59220
3255	C2	C	0.50000	0.61625	0.84220
3256	C2	C	0.75000	0.86625	0.84220
3257	C2	C	0.75000	0.61625	0.59220
3258	C2	C	0.50000	0.63375	0.59220
3259	C2	C	0.50000	0.88375	0.84220
3260	C2	C	0.75000	0.63375	0.84220
3261	C2	C	0.75000	0.88375	0.59220
3262	C2	C	0.63375	0.59220	0.50000
3263	C2	C	0.63375	0.84220	0.75000
3264	C2	C	0.88375	0.59220	0.75000
3265	C2	C	0.88375	0.84220	0.50000
3266	C2	C	0.86625	0.59220	0.50000
3267	C2	C	0.86625	0.84220	0.75000
3268	C2	C	0.61625	0.59220	0.75000
3269	C2	C	0.61625	0.84220	0.50000
3270	C2	C	0.86625	0.90780	0.50000
3271	C2	C	0.86625	0.65780	0.75000
3272	C2	C	0.61625	0.90780	0.75000
3273	C2	C	0.61625	0.65780	0.50000
3274	C2	C	0.63375	0.90780	0.50000
3275	C2	C	0.63375	0.65780	0.75000
3276	C2	C	0.88375	0.90780	0.75000
3277	C2	C	0.88375	0.65780	0.50000
3278	C2	C	0.59220	0.50000	0.86625
3279	C2	C	0.59220	0.75000	0.61625
3280	C2	C	0.84220	0.50000	0.61625
3281	C2	C	0.84220	0.75000	0.86625
3282	C2	C	0.59220	0.50000	0.63375
3283	C2	C	0.59220	0.75000	0.88375
3284	C2	C	0.84220	0.50000	0.88375
3285	C2	C	0.84220	0.75000	0.63375
3286	C2	C	0.90780	0.50000	0.63375
3287	C2	C	0.90780	0.75000	0.88375
3288	C2	C	0.65780	0.50000	0.88375
3289	C2	C	0.65780	0.75000	0.63375
3290	C2	C	0.90780	0.50000	0.86625
3291	C2	C	0.90780	0.75000	0.61625
3292	C2	C	0.65780	0.50000	0.61625
3293	C2	C	0.65780	0.75000	0.86625
3294	C1	C	0.57680	0.50000	0.92320

3295	C1	C	0.57680	0.75000	0.67320
3296	C1	C	0.82680	0.50000	0.67320
3297	C1	C	0.82680	0.75000	0.92320
3298	C1	C	0.92320	0.50000	0.92320
3299	C1	C	0.92320	0.75000	0.67320
3300	C1	C	0.67320	0.50000	0.67320
3301	C1	C	0.67320	0.75000	0.92320
3302	C1	C	0.92320	0.50000	0.57680
3303	C1	C	0.92320	0.75000	0.82680
3304	C1	C	0.67320	0.50000	0.82680
3305	C1	C	0.67320	0.75000	0.57680
3306	C1	C	0.57680	0.50000	0.57680
3307	C1	C	0.57680	0.75000	0.82680
3308	C1	C	0.82680	0.50000	0.82680
3309	C1	C	0.82680	0.75000	0.57680
3310	C1	C	0.92320	0.57680	0.50000
3311	C1	C	0.92320	0.82680	0.75000
3312	C1	C	0.67320	0.57680	0.75000
3313	C1	C	0.67320	0.82680	0.50000
3314	C1	C	0.92320	0.92320	0.50000
3315	C1	C	0.92320	0.67320	0.75000
3316	C1	C	0.67320	0.92320	0.75000
3317	C1	C	0.67320	0.67320	0.50000
3318	C1	C	0.57680	0.92320	0.50000
3319	C1	C	0.57680	0.67320	0.75000
3320	C1	C	0.82680	0.92320	0.75000
3321	C1	C	0.82680	0.67320	0.50000
3322	C1	C	0.57680	0.57680	0.50000
3323	C1	C	0.57680	0.82680	0.75000
3324	C1	C	0.82680	0.57680	0.75000
3325	C1	C	0.82680	0.82680	0.50000
3326	C1	C	0.50000	0.92320	0.57680
3327	C1	C	0.50000	0.67320	0.82680
3328	C1	C	0.75000	0.92320	0.82680
3329	C1	C	0.75000	0.67320	0.57680
3330	C1	C	0.50000	0.92320	0.92320
3331	C1	C	0.50000	0.67320	0.67320
3332	C1	C	0.75000	0.92320	0.67320
3333	C1	C	0.75000	0.67320	0.92320
3334	C1	C	0.50000	0.57680	0.92320
3335	C1	C	0.50000	0.82680	0.67320
3336	C1	C	0.75000	0.57680	0.67320
3337	C1	C	0.75000	0.82680	0.92320
3338	C1	C	0.50000	0.57680	0.57680
3339	C1	C	0.50000	0.82680	0.82680
3340	C1	C	0.75000	0.57680	0.82680
3341	C1	C	0.75000	0.82680	0.57680
3342	C3	C	0.60250	0.50000	0.60250
3343	C3	C	0.60250	0.75000	0.85250
3344	C3	C	0.85250	0.50000	0.85250
3345	C3	C	0.85250	0.75000	0.60250
3346	C3	C	0.89750	0.50000	0.60250
3347	C3	C	0.89750	0.75000	0.85250
3348	C3	C	0.64750	0.50000	0.85250

3349	C3	C	0.64750	0.75000	0.60250
3350	C3	C	0.89750	0.50000	0.89750
3351	C3	C	0.89750	0.75000	0.64750
3352	C3	C	0.64750	0.50000	0.64750
3353	C3	C	0.64750	0.75000	0.89750
3354	C3	C	0.60250	0.50000	0.89750
3355	C3	C	0.60250	0.75000	0.64750
3356	C3	C	0.85250	0.50000	0.64750
3357	C3	C	0.85250	0.75000	0.89750
3358	C3	C	0.60250	0.60250	0.50000
3359	C3	C	0.60250	0.85250	0.75000
3360	C3	C	0.85250	0.60250	0.75000
3361	C3	C	0.85250	0.85250	0.50000
3362	C3	C	0.60250	0.89750	0.50000
3363	C3	C	0.60250	0.64750	0.75000
3364	C3	C	0.85250	0.89750	0.75000
3365	C3	C	0.85250	0.64750	0.50000
3366	C3	C	0.89750	0.89750	0.50000
3367	C3	C	0.89750	0.64750	0.75000
3368	C3	C	0.64750	0.89750	0.75000
3369	C3	C	0.64750	0.64750	0.50000
3370	C3	C	0.89750	0.60250	0.50000
3371	C3	C	0.89750	0.85250	0.75000
3372	C3	C	0.64750	0.60250	0.75000
3373	C3	C	0.64750	0.85250	0.50000
3374	C3	C	0.50000	0.60250	0.60250
3375	C3	C	0.50000	0.85250	0.85250
3376	C3	C	0.75000	0.60250	0.85250
3377	C3	C	0.75000	0.85250	0.60250
3378	C3	C	0.50000	0.60250	0.89750
3379	C3	C	0.50000	0.85250	0.64750
3380	C3	C	0.75000	0.60250	0.64750
3381	C3	C	0.75000	0.85250	0.89750
3382	C3	C	0.50000	0.89750	0.89750
3383	C3	C	0.50000	0.64750	0.64750
3384	C3	C	0.75000	0.89750	0.64750
3385	C3	C	0.75000	0.64750	0.89750
3386	C3	C	0.50000	0.89750	0.60250
3387	C3	C	0.50000	0.64750	0.85250
3388	C3	C	0.75000	0.89750	0.85250
3389	C3	C	0.75000	0.64750	0.60250
3390	H1	H	0.63926	0.99997	0.56655
3391	H1	H	0.63926	0.74997	0.81655
3392	H1	H	0.88926	0.99997	0.81655
3393	H1	H	0.88926	0.74997	0.56655
3394	H1	H	0.86074	0.50003	0.56655
3395	H1	H	0.86074	0.75004	0.81655
3396	H1	H	0.61074	0.50003	0.81655
3397	H1	H	0.61074	0.75004	0.56655
3398	H1	H	0.86074	0.99996	0.93345
3399	H1	H	0.86074	0.74997	0.68345
3400	H1	H	0.61074	0.99997	0.68345
3401	H1	H	0.61074	0.74997	0.93346
3402	H1	H	0.63926	0.50003	0.93345

3403	H1	H	0.63926	0.75003	0.68346
3404	H1	H	0.88926	0.50003	0.68345
3405	H1	H	0.88926	0.75004	0.93346
3406	H1	H	0.56655	0.63926	0.99996
3407	H1	H	0.56655	0.88926	0.74997
3408	H1	H	0.81655	0.63926	0.74996
3409	H1	H	0.81654	0.88926	0.99997
3410	H1	H	0.56655	0.86074	0.50003
3411	H1	H	0.56655	0.61074	0.75004
3412	H1	H	0.81655	0.86074	0.75003
3413	H1	H	0.81655	0.61074	0.50004
3414	H1	H	0.93345	0.86074	0.99996
3415	H1	H	0.93345	0.61074	0.74997
3416	H1	H	0.68345	0.86074	0.74996
3417	H1	H	0.68345	0.61074	0.99997
3418	H1	H	0.93345	0.63926	0.50003
3419	H1	H	0.93345	0.88926	0.75004
3420	H1	H	0.68345	0.63926	0.75003
3421	H1	H	0.68345	0.88926	0.50004
3422	H1	H	0.99997	0.56655	0.63926
3423	H1	H	0.99996	0.81655	0.88926
3424	H1	H	0.74997	0.56655	0.88926
3425	H1	H	0.74996	0.81655	0.63926
3426	H1	H	0.50003	0.56655	0.86074
3427	H1	H	0.50003	0.81655	0.61074
3428	H1	H	0.75003	0.56655	0.61074
3429	H1	H	0.75004	0.81655	0.86074
3430	H1	H	0.99997	0.93345	0.86074
3431	H1	H	0.99997	0.68345	0.61074
3432	H1	H	0.74997	0.93345	0.61074
3433	H1	H	0.74997	0.68345	0.86074
3434	H1	H	0.50003	0.93345	0.63926
3435	H1	H	0.50003	0.68345	0.88926
3436	H1	H	0.75004	0.93345	0.88926
3437	H1	H	0.75003	0.68346	0.63926
3438	H1	H	0.50003	0.63926	0.93345
3439	H1	H	0.50003	0.88926	0.68345
3440	H1	H	0.75003	0.63926	0.68345
3441	H1	H	0.75004	0.88926	0.93345
3442	H1	H	0.99996	0.86074	0.93345
3443	H1	H	0.99997	0.61074	0.68345
3444	H1	H	0.74997	0.86074	0.68345
3445	H1	H	0.74997	0.61074	0.93345
3446	H1	H	0.50003	0.86074	0.56655
3447	H1	H	0.50003	0.61074	0.81655
3448	H1	H	0.75004	0.86074	0.81655
3449	H1	H	0.75003	0.61074	0.56654
3450	H1	H	0.99997	0.63926	0.56655
3451	H1	H	0.99997	0.88926	0.81655
3452	H1	H	0.74997	0.63926	0.81655
3453	H1	H	0.74997	0.88926	0.56654
3454	H1	H	0.63926	0.56655	0.99997
3455	H1	H	0.63926	0.81655	0.74996
3456	H1	H	0.88926	0.56655	0.74997

3457	H1	H	0.88926	0.81655	0.99996
3458	H1	H	0.86074	0.56655	0.50003
3459	H1	H	0.86074	0.81655	0.75003
3460	H1	H	0.61074	0.56655	0.75003
3461	H1	H	0.61074	0.81655	0.50003
3462	H1	H	0.86074	0.93345	0.99997
3463	H1	H	0.86074	0.68345	0.74997
3464	H1	H	0.61074	0.93345	0.74997
3465	H1	H	0.61074	0.68345	0.99996
3466	H1	H	0.63926	0.93345	0.50004
3467	H1	H	0.63926	0.68346	0.75003
3468	H1	H	0.88926	0.93345	0.75004
3469	H1	H	0.88926	0.68346	0.50003
3470	H1	H	0.56655	0.50003	0.86074
3471	H1	H	0.56655	0.75004	0.61074
3472	H1	H	0.81655	0.50003	0.61074
3473	H1	H	0.81655	0.75004	0.86074
3474	H1	H	0.56655	0.99996	0.63926
3475	H1	H	0.56655	0.74997	0.88926
3476	H1	H	0.81655	0.99996	0.88926
3477	H1	H	0.81655	0.74997	0.63926
3478	H1	H	0.93345	0.50003	0.63926
3479	H1	H	0.93345	0.75004	0.88926
3480	H1	H	0.68345	0.50003	0.88926
3481	H1	H	0.68345	0.75003	0.63926
3482	H1	H	0.93345	0.99996	0.86074
3483	H1	H	0.93345	0.74997	0.61074
3484	H1	H	0.68345	0.99996	0.61074
3485	H1	H	0.68345	0.74997	0.86074
3486	H2	H	0.70825	0.79176	0.95824
3487	H2	H	0.79174	0.70825	0.95823
3488	H2	H	0.70827	0.70823	0.54177
3489	H2	H	0.79179	0.79168	0.54178
3490	H2	H	0.95823	0.79173	0.70823
3491	H2	H	0.95824	0.70825	0.79176
3492	H2	H	0.54174	0.79176	0.79177
3493	H2	H	0.54173	0.70823	0.70823
3494	H2	H	0.79175	0.95824	0.70824
3495	H2	H	0.70824	0.95824	0.79176
3496	H2	H	0.70823	0.54172	0.70822
3497	H2	H	0.79177	0.54173	0.79178
3498	H2	H	0.54178	0.54177	0.54173
3499	H2	H	0.95823	0.95824	0.54174
3500	H2	H	0.95823	0.54176	0.95825
3501	H2	H	0.54176	0.95824	0.95824
3502	O1	O	0.58524	0.50000	0.54765
3503	O1	O	0.58524	0.75000	0.79765
3504	O1	O	0.83524	0.50000	0.79765
3505	O1	O	0.83524	0.75000	0.54765
3506	O1	O	0.91476	0.50000	0.54765
3507	O1	O	0.91476	0.75000	0.79765
3508	O1	O	0.66476	0.50000	0.79765
3509	O1	O	0.66476	0.75000	0.54765
3510	O1	O	0.91476	0.50000	0.95235

3511	O1	O	0.91476	0.75000	0.70235
3512	O1	O	0.66476	0.50000	0.70235
3513	O1	O	0.66476	0.75000	0.95235
3514	O1	O	0.58524	0.50000	0.95235
3515	O1	O	0.58524	0.75000	0.70235
3516	O1	O	0.83524	0.50000	0.70235
3517	O1	O	0.83524	0.75000	0.95235
3518	O1	O	0.54765	0.58524	0.50000
3519	O1	O	0.54765	0.83524	0.75000
3520	O1	O	0.79765	0.58524	0.75000
3521	O1	O	0.79765	0.83524	0.50000
3522	O1	O	0.54765	0.91476	0.50000
3523	O1	O	0.54765	0.66476	0.75000
3524	O1	O	0.79765	0.91476	0.75000
3525	O1	O	0.79765	0.66476	0.50000
3526	O1	O	0.95235	0.91476	0.50000
3527	O1	O	0.95235	0.66476	0.75000
3528	O1	O	0.70235	0.91476	0.75000
3529	O1	O	0.70235	0.66476	0.50000
3530	O1	O	0.95235	0.58524	0.50000
3531	O1	O	0.95235	0.83524	0.75000
3532	O1	O	0.70235	0.58524	0.75000
3533	O1	O	0.70235	0.83524	0.50000
3534	O1	O	0.50000	0.54765	0.58524
3535	O1	O	0.50000	0.79765	0.83524
3536	O1	O	0.75000	0.54765	0.83524
3537	O1	O	0.75000	0.79765	0.58524
3538	O1	O	0.50000	0.54765	0.91476
3539	O1	O	0.50000	0.79765	0.66476
3540	O1	O	0.75000	0.54765	0.66476
3541	O1	O	0.75000	0.79765	0.91476
3542	O1	O	0.50000	0.95235	0.91476
3543	O1	O	0.50000	0.70235	0.66476
3544	O1	O	0.75000	0.95235	0.66476
3545	O1	O	0.75000	0.70235	0.91476
3546	O1	O	0.50000	0.95235	0.58524
3547	O1	O	0.50000	0.70235	0.83524
3548	O1	O	0.75000	0.95235	0.83524
3549	O1	O	0.75000	0.70235	0.58524
3550	O1	O	0.50000	0.58524	0.95235
3551	O1	O	0.50000	0.83524	0.70235
3552	O1	O	0.75000	0.58524	0.70235
3553	O1	O	0.75000	0.83524	0.95235
3554	O1	O	0.50000	0.91476	0.95235
3555	O1	O	0.50000	0.66476	0.70235
3556	O1	O	0.75000	0.91476	0.70235
3557	O1	O	0.75000	0.66476	0.95235
3558	O1	O	0.50000	0.91476	0.54765
3559	O1	O	0.50000	0.66476	0.79765
3560	O1	O	0.75000	0.91476	0.79765
3561	O1	O	0.75000	0.66476	0.54765
3562	O1	O	0.50000	0.58524	0.54765
3563	O1	O	0.50000	0.83524	0.79765
3564	O1	O	0.75000	0.58524	0.79765

3565	O1	O	0.75000	0.83524	0.54765
3566	O1	O	0.58524	0.54765	0.50000
3567	O1	O	0.58524	0.79765	0.75000
3568	O1	O	0.83524	0.54765	0.75000
3569	O1	O	0.83524	0.79765	0.50000
3570	O1	O	0.91476	0.54765	0.50000
3571	O1	O	0.91476	0.79765	0.75000
3572	O1	O	0.66476	0.54765	0.75000
3573	O1	O	0.66476	0.79765	0.50000
3574	O1	O	0.91476	0.95235	0.50000
3575	O1	O	0.91476	0.70235	0.75000
3576	O1	O	0.66476	0.95235	0.75000
3577	O1	O	0.66476	0.70235	0.50000
3578	O1	O	0.58524	0.95235	0.50000
3579	O1	O	0.58524	0.70235	0.75000
3580	O1	O	0.83524	0.95235	0.75000
3581	O1	O	0.83524	0.70235	0.50000
3582	O1	O	0.54765	0.50000	0.91476
3583	O1	O	0.54765	0.75000	0.66476
3584	O1	O	0.79765	0.50000	0.66476
3585	O1	O	0.79765	0.75000	0.91476
3586	O1	O	0.54765	0.50000	0.58524
3587	O1	O	0.54765	0.75000	0.83524
3588	O1	O	0.79765	0.50000	0.83524
3589	O1	O	0.79765	0.75000	0.58524
3590	O1	O	0.95235	0.50000	0.58524
3591	O1	O	0.95235	0.75000	0.83524
3592	O1	O	0.70235	0.50000	0.83524
3593	O1	O	0.70235	0.75000	0.58524
3594	O1	O	0.95235	0.50000	0.91476
3595	O1	O	0.95235	0.75000	0.66476
3596	O1	O	0.70235	0.50000	0.66476
3597	O1	O	0.70235	0.75000	0.91476
3598	O2	O	0.52805	0.97195	0.97195
3599	O2	O	0.52805	0.72195	0.72195
3600	O2	O	0.77805	0.97195	0.72195
3601	O2	O	0.77805	0.72195	0.97195
3602	O2	O	0.97195	0.52805	0.97195
3603	O2	O	0.97195	0.77805	0.72195
3604	O2	O	0.72195	0.52805	0.72195
3605	O2	O	0.72195	0.77805	0.97195
3606	O2	O	0.97195	0.97195	0.52805
3607	O2	O	0.97195	0.72195	0.77805
3608	O2	O	0.72195	0.97195	0.77805
3609	O2	O	0.72195	0.72195	0.52805
3610	O2	O	0.52805	0.52805	0.52805
3611	O2	O	0.52805	0.77805	0.77805
3612	O2	O	0.77805	0.52805	0.77805
3613	O2	O	0.77805	0.77805	0.52805
3614	O1	O	0.97195	0.52805	0.52805
3615	O1	O	0.97195	0.77805	0.77805
3616	O1	O	0.72195	0.52805	0.77805
3617	O1	O	0.72195	0.77805	0.52805
3618	O1	O	0.52805	0.97195	0.52805

3619	O1	O	0.52805	0.72195	0.77805
3620	O1	O	0.77805	0.97195	0.77805
3621	O1	O	0.77805	0.72195	0.52805
3622	O1	O	0.97195	0.97195	0.97195
3623	O1	O	0.97195	0.72195	0.72195
3624	O1	O	0.72195	0.97195	0.72195
3625	O1	O	0.72195	0.72195	0.97195
3626	O1	O	0.52805	0.52805	0.97195
3627	O1	O	0.52805	0.77805	0.72195
3628	O1	O	0.77805	0.52805	0.72195
3629	O1	O	0.77805	0.77805	0.97195
3630	Zr1	Zr	0.55994	0.50000	0.50000
3631	Zr1	Zr	0.55994	0.75000	0.75000
3632	Zr1	Zr	0.80994	0.50000	0.75000
3633	Zr1	Zr	0.80994	0.75000	0.50000
3634	Zr1	Zr	0.94006	0.50000	0.50000
3635	Zr1	Zr	0.94006	0.75000	0.75000
3636	Zr1	Zr	0.69006	0.50000	0.75000
3637	Zr1	Zr	0.69006	0.75000	0.50000
3638	Zr1	Zr	0.50000	0.55994	0.50000
3639	Zr1	Zr	0.50000	0.80994	0.75000
3640	Zr1	Zr	0.75000	0.55994	0.75000
3641	Zr1	Zr	0.75000	0.80994	0.50000
3642	Zr1	Zr	0.50000	0.94006	0.50000
3643	Zr1	Zr	0.50000	0.69006	0.75000
3644	Zr1	Zr	0.75000	0.94006	0.75000
3645	Zr1	Zr	0.75000	0.69006	0.50000
3646	Zr1	Zr	0.50000	0.50000	0.55994
3647	Zr1	Zr	0.50000	0.75000	0.80994
3648	Zr1	Zr	0.75000	0.50000	0.80994
3649	Zr1	Zr	0.75000	0.75000	0.55994
3650	Zr1	Zr	0.50000	0.50000	0.94006
3651	Zr1	Zr	0.50000	0.75000	0.69006
3652	Zr1	Zr	0.75000	0.50000	0.69006
3653	Zr1	Zr	0.75000	0.75000	0.94006
3654	H3	H	0.45705	0.60274	0.49978
3655	H3	H	0.39442	0.54974	0.49979
3656	H3	H	0.35697	0.70270	0.50002
3657	H3	H	0.28977	0.64489	0.49991
