# Supplementary material for: Defect-induced tuning of polarity-dependent adsorption in hydrophobic–hydrophilic UiO-66
Source: Commun Chem. 2022 Oct 7;5:120. doi: 10.1038/s42004-022-00742-z (PMC9814431; doi:10.1038/s42004-022-00742-z)
Supplement: Supplementary file 6 — Supplementary Data 3 [file 42004_2022_742_MOESM6_ESM.pdf]

```
data_UiO-66_2

_cell_length_a                    41.4008
_cell_length_b                    41.4008
_cell_length_c                    41.4008
_cell_angle_alpha                 90.0000
_cell_angle_beta                  90.0000
_cell_angle_gamma                 90.0000


_symmetry_equiv_pos_as_xyz 'x,y,z'

loop_
_atom_site_label
_atom_site_type_symbol
_atom_site_fract_x
_atom_site_fract_y
_atom_site_fract_z
C2  C     0.13375   0.00000   0.09220
C2  C     0.13375   0.25000   0.34220
C2  C     0.38375   0.00000   0.34220
C2  C     0.38375   0.25000   0.09220
C2  C     0.36625   0.00000   0.09220
C2  C     0.36625   0.25000   0.34220
C2  C     0.11625   0.00000   0.34220
C2  C     0.11625   0.25000   0.09220
C2  C     0.36625   0.00000   0.40780
C2  C     0.36625   0.25000   0.15780
C2  C     0.11625   0.00000   0.15780
C2  C     0.11625   0.25000   0.40780
C2  C     0.13375   0.00000   0.40780
C2  C     0.13375   0.25000   0.15780
C2  C     0.38375   0.00000   0.15780
C2  C     0.38375   0.25000   0.40780
C2  C     0.09220   0.13375   0.00000
C2  C     0.09220   0.38375   0.25000
C2  C     0.34220   0.13375   0.25000
C2  C     0.34220   0.38375   0.00000
C2  C     0.09220   0.36625   0.00000
C2  C     0.09220   0.11625   0.25000
C2  C     0.34220   0.36625   0.25000
C2  C     0.34220   0.11625   0.00000
C2  C     0.40780   0.36625   0.00000
C2  C     0.40780   0.11625   0.25000
C2  C     0.15780   0.36625   0.25000
C2  C     0.15780   0.11625   0.00000
C2  C     0.40780   0.13375   0.00000
C2  C     0.40780   0.38375   0.25000
C2  C     0.15780   0.13375   0.25000
C2  C     0.15780   0.38375   0.00000
C2  C     0.00000   0.09220   0.13375
C2  C     0.00000   0.34220   0.38375
C2  C     0.25000   0.09220   0.38375
C2  C     0.25000   0.34220   0.13375
```

55	C2	C	0.00000	0.09220	0.36625
56	C2	C	0.00000	0.34220	0.11625
57	C2	C	0.25000	0.09220	0.11625
58	C2	C	0.25000	0.34220	0.36625
59	C2	C	0.00000	0.40780	0.36625
60	C2	C	0.00000	0.15780	0.11625
61	C2	C	0.25000	0.40780	0.11625
62	C2	C	0.25000	0.15780	0.36625
63	C2	C	0.00000	0.40780	0.13375
64	C2	C	0.00000	0.15780	0.38375
65	C2	C	0.25000	0.40780	0.38375
66	C2	C	0.25000	0.15780	0.13375
67	C2	C	0.00000	0.13375	0.40780
68	C2	C	0.00000	0.38375	0.15780
69	C2	C	0.25000	0.13375	0.15780
70	C2	C	0.25000	0.38375	0.40780
71	C2	C	0.00000	0.36625	0.40780
72	C2	C	0.00000	0.11625	0.15780
73	C2	C	0.25000	0.36625	0.15780
74	C2	C	0.25000	0.11625	0.40780
75	C2	C	0.00000	0.36625	0.09220
76	C2	C	0.00000	0.11625	0.34220
77	C2	C	0.25000	0.36625	0.34220
78	C2	C	0.25000	0.11625	0.09220
79	C2	C	0.00000	0.13375	0.09220
80	C2	C	0.00000	0.38375	0.34220
81	C2	C	0.25000	0.13375	0.34220
82	C2	C	0.25000	0.38375	0.09220
83	C2	C	0.13375	0.09220	0.00000
84	C2	C	0.13375	0.34220	0.25000
85	C2	C	0.38375	0.09220	0.25000
86	C2	C	0.38375	0.34220	0.00000
87	C2	C	0.36625	0.09220	0.00000
88	C2	C	0.36625	0.34220	0.25000
89	C2	C	0.11625	0.09220	0.25000
90	C2	C	0.11625	0.34220	0.00000
91	C2	C	0.36625	0.40780	0.00000
92	C2	C	0.36625	0.15780	0.25000
93	C2	C	0.11625	0.40780	0.25000
94	C2	C	0.11625	0.15780	0.00000
95	C2	C	0.13375	0.40780	0.00000
96	C2	C	0.13375	0.15780	0.25000
97	C2	C	0.38375	0.40780	0.25000
98	C2	C	0.38375	0.15780	0.00000
99	C2	C	0.09220	0.00000	0.36625
100	C2	C	0.09220	0.25000	0.11625
101	C2	C	0.34220	0.00000	0.11625
102	C2	C	0.34220	0.25000	0.36625
103	C2	C	0.09220	0.00000	0.13375
104	C2	C	0.09220	0.25000	0.38375
105	C2	C	0.34220	0.00000	0.38375
106	C2	C	0.34220	0.25000	0.13375
107	C2	C	0.40780	0.00000	0.13375
108	C2	C	0.40780	0.25000	0.38375

109	C2	C	0.15780	0.00000	0.38375
110	C2	C	0.15780	0.25000	0.13375
111	C2	C	0.40780	0.00000	0.36625
112	C2	C	0.40780	0.25000	0.11625
113	C2	C	0.15780	0.00000	0.11625
114	C2	C	0.15780	0.25000	0.36625
115	C1	C	0.07680	0.00000	0.42320
116	C1	C	0.07680	0.25000	0.17320
117	C1	C	0.32680	0.00000	0.17320
118	C1	C	0.32680	0.25000	0.42320
119	C1	C	0.42320	0.00000	0.42320
120	C1	C	0.42320	0.25000	0.17320
121	C1	C	0.17320	0.00000	0.17320
122	C1	C	0.17320	0.25000	0.42320
123	C1	C	0.42320	0.00000	0.07680
124	C1	C	0.42320	0.25000	0.32680
125	C1	C	0.17320	0.00000	0.32680
126	C1	C	0.17320	0.25000	0.07680
127	C1	C	0.07680	0.00000	0.07680
128	C1	C	0.07680	0.25000	0.32680
129	C1	C	0.32680	0.00000	0.32680
130	C1	C	0.32680	0.25000	0.07680
131	C1	C	0.42320	0.07680	0.00000
132	C1	C	0.42320	0.32680	0.25000
133	C1	C	0.17320	0.07680	0.25000
134	C1	C	0.17320	0.32680	0.00000
135	C1	C	0.42320	0.42320	0.00000
136	C1	C	0.42320	0.17320	0.25000
137	C1	C	0.17320	0.42320	0.25000
138	C1	C	0.17320	0.17320	0.00000
139	C1	C	0.07680	0.42320	0.00000
140	C1	C	0.07680	0.17320	0.25000
141	C1	C	0.32680	0.42320	0.25000
142	C1	C	0.32680	0.17320	0.00000
143	C1	C	0.07680	0.07680	0.00000
144	C1	C	0.07680	0.32680	0.25000
145	C1	C	0.32680	0.07680	0.25000
146	C1	C	0.32680	0.32680	0.00000
147	C1	C	0.00000	0.42320	0.07680
148	C1	C	0.00000	0.17320	0.32680
149	C1	C	0.25000	0.42320	0.32680
150	C1	C	0.25000	0.17320	0.07680
151	C1	C	0.00000	0.42320	0.42320
152	C1	C	0.00000	0.17320	0.17320
153	C1	C	0.25000	0.42320	0.17320
154	C1	C	0.25000	0.17320	0.42320
155	C1	C	0.00000	0.07680	0.42320
156	C1	C	0.00000	0.32680	0.17320
157	C1	C	0.25000	0.07680	0.17320
158	C1	C	0.25000	0.32680	0.42320
159	C1	C	0.00000	0.07680	0.07680
160	C1	C	0.00000	0.32680	0.32680
161	C1	C	0.25000	0.07680	0.32680
162	C1	C	0.25000	0.32680	0.07680

163	C3	C	0.10250	0.00000	0.10250
164	C3	C	0.10250	0.25000	0.35250
165	C3	C	0.35250	0.00000	0.35250
166	C3	C	0.35250	0.25000	0.10250
167	C3	C	0.39750	0.00000	0.10250
168	C3	C	0.39750	0.25000	0.35250
169	C3	C	0.14750	0.00000	0.35250
170	C3	C	0.14750	0.25000	0.10250
171	C3	C	0.39750	0.00000	0.39750
172	C3	C	0.39750	0.25000	0.14750
173	C3	C	0.14750	0.00000	0.14750
174	C3	C	0.14750	0.25000	0.39750
175	C3	C	0.10250	0.00000	0.39750
176	C3	C	0.10250	0.25000	0.14750
177	C3	C	0.35250	0.00000	0.14750
178	C3	C	0.35250	0.25000	0.39750
179	C3	C	0.10250	0.10250	0.00000
180	C3	C	0.10250	0.35250	0.25000
181	C3	C	0.35250	0.10250	0.25000
182	C3	C	0.35250	0.35250	0.00000
183	C3	C	0.10250	0.39750	0.00000
184	C3	C	0.10250	0.14750	0.25000
185	C3	C	0.35250	0.39750	0.25000
186	C3	C	0.35250	0.14750	0.00000
187	C3	C	0.39750	0.39750	0.00000
188	C3	C	0.39750	0.14750	0.25000
189	C3	C	0.14750	0.39750	0.25000
190	C3	C	0.14750	0.14750	0.00000
191	C3	C	0.39750	0.10250	0.00000
192	C3	C	0.39750	0.35250	0.25000
193	C3	C	0.14750	0.10250	0.25000
194	C3	C	0.14750	0.35250	0.00000
195	C3	C	0.00000	0.10250	0.10250
196	C3	C	0.00000	0.35250	0.35250
197	C3	C	0.25000	0.10250	0.35250
198	C3	C	0.25000	0.35250	0.10250
199	C3	C	0.00000	0.10250	0.39750
200	C3	C	0.00000	0.35250	0.14750
201	C3	C	0.25000	0.10250	0.14750
202	C3	C	0.25000	0.35250	0.39750
203	C3	C	0.00000	0.39750	0.39750
204	C3	C	0.00000	0.14750	0.14750
205	C3	C	0.25000	0.39750	0.14750
206	C3	C	0.25000	0.14750	0.39750
207	C3	C	0.00000	0.39750	0.10250
208	C3	C	0.00000	0.14750	0.35250
209	C3	C	0.25000	0.39750	0.35250
210	C3	C	0.25000	0.14750	0.10250
211	H1	H	0.13926	0.49997	0.06655
212	H1	H	0.13926	0.24997	0.31655
213	H1	H	0.38926	0.49997	0.31655
214	H1	H	0.38926	0.24997	0.06655
215	H1	H	0.36074	0.00003	0.06655
216	H1	H	0.36074	0.25004	0.31655

217	H1	H	0.11074	0.00003	0.31655
218	H1	H	0.11074	0.25004	0.06655
219	H1	H	0.36074	0.49996	0.43345
220	H1	H	0.36074	0.24997	0.18345
221	H1	H	0.11074	0.49997	0.18345
222	H1	H	0.11074	0.24997	0.43346
223	H1	H	0.13926	0.00003	0.43345
224	H1	H	0.13926	0.25003	0.18346
225	H1	H	0.38926	0.00003	0.18345
226	H1	H	0.38926	0.25004	0.43346
227	H1	H	0.06655	0.13926	0.49996
228	H1	H	0.06655	0.38926	0.24997
229	H1	H	0.31655	0.13926	0.24996
230	H1	H	0.31654	0.38926	0.49997
231	H1	H	0.06655	0.36074	0.00003
232	H1	H	0.06655	0.11074	0.25004
233	H1	H	0.31655	0.36074	0.25003
234	H1	H	0.31655	0.11074	0.00004
235	H1	H	0.43345	0.36074	0.49996
236	H1	H	0.43345	0.11074	0.24997
237	H1	H	0.18345	0.36074	0.24996
238	H1	H	0.18345	0.11074	0.49997
239	H1	H	0.43345	0.13926	0.00003
240	H1	H	0.43345	0.38926	0.25004
241	H1	H	0.18345	0.13926	0.25003
242	H1	H	0.18345	0.38926	0.00004
243	H1	H	0.49997	0.06655	0.13926
244	H1	H	0.49996	0.31655	0.38926
245	H1	H	0.24997	0.06655	0.38926
246	H1	H	0.24996	0.31655	0.13926
247	H1	H	0.00003	0.06655	0.36074
248	H1	H	0.00003	0.31655	0.11074
249	H1	H	0.25003	0.06655	0.11074
250	H1	H	0.25004	0.31655	0.36074
251	H1	H	0.49997	0.43345	0.36074
252	H1	H	0.49997	0.18345	0.11074
253	H1	H	0.24997	0.43345	0.11074
254	H1	H	0.24997	0.18345	0.36074
255	H1	H	0.00003	0.43345	0.13926
256	H1	H	0.00003	0.18345	0.38926
257	H1	H	0.25004	0.43345	0.38926
258	H1	H	0.25003	0.18346	0.13926
259	H1	H	0.00003	0.13926	0.43345
260	H1	H	0.00003	0.38926	0.18345
261	H1	H	0.25003	0.13926	0.18345
262	H1	H	0.25004	0.38926	0.43345
263	H1	H	0.49996	0.36074	0.43345
264	H1	H	0.49997	0.11074	0.18345
265	H1	H	0.24997	0.36074	0.18345
266	H1	H	0.24997	0.11074	0.43345
267	H1	H	0.00003	0.36074	0.06655
268	H1	H	0.00003	0.11074	0.31655
269	H1	H	0.25004	0.36074	0.31655
270	H1	H	0.25003	0.11074	0.06654

271	H1	H	0.49997	0.13926	0.06655
272	H1	H	0.49997	0.38926	0.31655
273	H1	H	0.24997	0.13926	0.31655
274	H1	H	0.24997	0.38926	0.06654
275	H1	H	0.13926	0.06655	0.49997
276	H1	H	0.13926	0.31655	0.24996
277	H1	H	0.38926	0.06655	0.24997
278	H1	H	0.38926	0.31655	0.49996
279	H1	H	0.36074	0.06655	0.00003
280	H1	H	0.36074	0.31655	0.25003
281	H1	H	0.11074	0.06655	0.25003
282	H1	H	0.11074	0.31655	0.00003
283	H1	H	0.36074	0.43345	0.49997
284	H1	H	0.36074	0.18345	0.24997
285	H1	H	0.11074	0.43345	0.24997
286	H1	H	0.11074	0.18345	0.49996
287	H1	H	0.13926	0.43345	0.00004
288	H1	H	0.13926	0.18346	0.25003
289	H1	H	0.38926	0.43345	0.25004
290	H1	H	0.38926	0.18346	0.00003
291	H1	H	0.06655	0.00003	0.36074
292	H1	H	0.06655	0.25004	0.11074
293	H1	H	0.31655	0.00003	0.11074
294	H1	H	0.31655	0.25004	0.36074
295	H1	H	0.06655	0.49996	0.13926
296	H1	H	0.06655	0.24997	0.38926
297	H1	H	0.31655	0.49996	0.38926
298	H1	H	0.31655	0.24997	0.13926
299	H1	H	0.43345	0.00003	0.13926
300	H1	H	0.43345	0.25004	0.38926
301	H1	H	0.18345	0.00003	0.38926
302	H1	H	0.18345	0.25003	0.13926
303	H1	H	0.43345	0.49996	0.36074
304	H1	H	0.43345	0.24997	0.11074
305	H1	H	0.18345	0.49996	0.11074
306	H1	H	0.18345	0.24997	0.36074
307	H2	H	0.20825	0.29176	0.45824
308	H2	H	0.29174	0.20825	0.45823
309	H2	H	0.20827	0.20823	0.04177
310	H2	H	0.29179	0.29168	0.04178
311	H2	H	0.45823	0.29173	0.20823
312	H2	H	0.45824	0.20825	0.29176
313	H2	H	0.04174	0.29176	0.29177
314	H2	H	0.04173	0.20823	0.20823
315	H2	H	0.29175	0.45824	0.20824
316	H2	H	0.20824	0.45824	0.29176
317	H2	H	0.20823	0.04172	0.20822
318	H2	H	0.29177	0.04173	0.29178
319	H2	H	0.04178	0.04177	0.04173
320	H2	H	0.45823	0.45824	0.04174
321	H2	H	0.45823	0.04176	0.45825
322	H2	H	0.04176	0.45824	0.45824
323	O1	O	0.08524	0.00000	0.04765
324	O1	O	0.08524	0.25000	0.29765

325	O1	O	0.33524	0.00000	0.29765
326	O1	O	0.33524	0.25000	0.04765
327	O1	O	0.41476	0.00000	0.04765
328	O1	O	0.41476	0.25000	0.29765
329	O1	O	0.16476	0.00000	0.29765
330	O1	O	0.16476	0.25000	0.04765
331	O1	O	0.41476	0.00000	0.45235
332	O1	O	0.41476	0.25000	0.20235
333	O1	O	0.16476	0.00000	0.20235
334	O1	O	0.16476	0.25000	0.45235
335	O1	O	0.08524	0.00000	0.45235
336	O1	O	0.08524	0.25000	0.20235
337	O1	O	0.33524	0.00000	0.20235
338	O1	O	0.33524	0.25000	0.45235
339	O1	O	0.04765	0.08524	0.00000
340	O1	O	0.04765	0.33524	0.25000
341	O1	O	0.29765	0.08524	0.25000
342	O1	O	0.29765	0.33524	0.00000
343	O1	O	0.04765	0.41476	0.00000
344	O1	O	0.04765	0.16476	0.25000
345	O1	O	0.29765	0.41476	0.25000
346	O1	O	0.29765	0.16476	0.00000
347	O1	O	0.45235	0.41476	0.00000
348	O1	O	0.45235	0.16476	0.25000
349	O1	O	0.20235	0.41476	0.25000
350	O1	O	0.20235	0.16476	0.00000
351	O1	O	0.45235	0.08524	0.00000
352	O1	O	0.45235	0.33524	0.25000
353	O1	O	0.20235	0.08524	0.25000
354	O1	O	0.20235	0.33524	0.00000
355	O1	O	0.00000	0.04765	0.08524
356	O1	O	0.00000	0.29765	0.33524
357	O1	O	0.25000	0.04765	0.33524
358	O1	O	0.25000	0.29765	0.08524
359	O1	O	0.00000	0.04765	0.41476
360	O1	O	0.00000	0.29765	0.16476
361	O1	O	0.25000	0.04765	0.16476
362	O1	O	0.25000	0.29765	0.41476
363	O1	O	0.00000	0.45235	0.41476
364	O1	O	0.00000	0.20235	0.16476
365	O1	O	0.25000	0.45235	0.16476
366	O1	O	0.25000	0.20235	0.41476
367	O1	O	0.00000	0.45235	0.08524
368	O1	O	0.00000	0.20235	0.33524
369	O1	O	0.25000	0.45235	0.33524
370	O1	O	0.25000	0.20235	0.08524
371	O1	O	0.00000	0.08524	0.45235
372	O1	O	0.00000	0.33524	0.20235
373	O1	O	0.25000	0.08524	0.20235
374	O1	O	0.25000	0.33524	0.45235
375	O1	O	0.00000	0.41476	0.45235
376	O1	O	0.00000	0.16476	0.20235
377	O1	O	0.25000	0.41476	0.20235
378	O1	O	0.25000	0.16476	0.45235

379	O1	O	0.00000	0.41476	0.04765
380	O1	O	0.00000	0.16476	0.29765
381	O1	O	0.25000	0.41476	0.29765
382	O1	O	0.25000	0.16476	0.04765
383	O1	O	0.00000	0.08524	0.04765
384	O1	O	0.00000	0.33524	0.29765
385	O1	O	0.25000	0.08524	0.29765
386	O1	O	0.25000	0.33524	0.04765
387	O1	O	0.08524	0.04765	0.00000
388	O1	O	0.08524	0.29765	0.25000
389	O1	O	0.33524	0.04765	0.25000
390	O1	O	0.33524	0.29765	0.00000
391	O1	O	0.41476	0.04765	0.00000
392	O1	O	0.41476	0.29765	0.25000
393	O1	O	0.16476	0.04765	0.25000
394	O1	O	0.16476	0.29765	0.00000
395	O1	O	0.41476	0.45235	0.00000
396	O1	O	0.41476	0.20235	0.25000
397	O1	O	0.16476	0.45235	0.25000
398	O1	O	0.16476	0.20235	0.00000
399	O1	O	0.08524	0.45235	0.00000
400	O1	O	0.08524	0.20235	0.25000
401	O1	O	0.33524	0.45235	0.25000
402	O1	O	0.33524	0.20235	0.00000
403	O1	O	0.04765	0.00000	0.41476
404	O1	O	0.04765	0.25000	0.16476
405	O1	O	0.29765	0.00000	0.16476
406	O1	O	0.29765	0.25000	0.41476
407	O1	O	0.04765	0.00000	0.08524
408	O1	O	0.04765	0.25000	0.33524
409	O1	O	0.29765	0.00000	0.33524
410	O1	O	0.29765	0.25000	0.08524
411	O1	O	0.45235	0.00000	0.08524
412	O1	O	0.45235	0.25000	0.33524
413	O1	O	0.20235	0.00000	0.33524
414	O1	O	0.20235	0.25000	0.08524
415	O1	O	0.45235	0.00000	0.41476
416	O1	O	0.45235	0.25000	0.16476
417	O1	O	0.20235	0.00000	0.16476
418	O1	O	0.20235	0.25000	0.41476
419	O2	O	0.02805	0.47195	0.47195
420	O2	O	0.02805	0.22195	0.22195
421	O2	O	0.27805	0.47195	0.22195
422	O2	O	0.27805	0.22195	0.47195
423	O2	O	0.47195	0.02805	0.47195
424	O2	O	0.47195	0.27805	0.22195
425	O2	O	0.22195	0.02805	0.22195
426	O2	O	0.22195	0.27805	0.47195
427	O2	O	0.47195	0.47195	0.02805
428	O2	O	0.47195	0.22195	0.27805
429	O2	O	0.22195	0.47195	0.27805
430	O2	O	0.22195	0.22195	0.02805
431	O2	O	0.02805	0.02805	0.02805
432	O2	O	0.02805	0.27805	0.27805

433	O2	O	0.27805	0.02805	0.27805
434	O2	O	0.27805	0.27805	0.02805
435	O1	O	0.47195	0.02805	0.02805
436	O1	O	0.47195	0.27805	0.27805
437	O1	O	0.22195	0.02805	0.27805
438	O1	O	0.22195	0.27805	0.02805
439	O1	O	0.02805	0.47195	0.02805
440	O1	O	0.02805	0.22195	0.27805
441	O1	O	0.27805	0.47195	0.27805
442	O1	O	0.27805	0.22195	0.02805
443	O1	O	0.47195	0.47195	0.47195
444	O1	O	0.47195	0.22195	0.22195
445	O1	O	0.22195	0.47195	0.22195
446	O1	O	0.22195	0.22195	0.47195
447	O1	O	0.02805	0.02805	0.47195
448	O1	O	0.02805	0.27805	0.22195
449	O1	O	0.27805	0.02805	0.22195
450	O1	O	0.27805	0.27805	0.47195
451	Zr1	Zr	0.05994	0.00000	0.00000
452	Zr1	Zr	0.05994	0.25000	0.25000
453	Zr1	Zr	0.30994	0.00000	0.25000
454	Zr1	Zr	0.30994	0.25000	0.00000
455	Zr1	Zr	0.44006	0.00000	0.00000
456	Zr1	Zr	0.44006	0.25000	0.25000
457	Zr1	Zr	0.19006	0.00000	0.25000
458	Zr1	Zr	0.19006	0.25000	0.00000
459	Zr1	Zr	0.00000	0.05994	0.00000
460	Zr1	Zr	0.00000	0.30994	0.25000
461	Zr1	Zr	0.25000	0.05994	0.25000
462	Zr1	Zr	0.25000	0.30994	0.00000
463	Zr1	Zr	0.00000	0.44006	0.00000
464	Zr1	Zr	0.00000	0.19006	0.25000
465	Zr1	Zr	0.25000	0.44006	0.25000
466	Zr1	Zr	0.25000	0.19006	0.00000
467	Zr1	Zr	0.00000	0.00000	0.05994
468	Zr1	Zr	0.00000	0.25000	0.30994
469	Zr1	Zr	0.25000	0.00000	0.30994
470	Zr1	Zr	0.25000	0.25000	0.05994
471	Zr1	Zr	0.00000	0.00000	0.44006
472	Zr1	Zr	0.00000	0.25000	0.19006
473	Zr1	Zr	0.25000	0.00000	0.19006
474	Zr1	Zr	0.25000	0.25000	0.44006
475	C2	C	0.13375	0.00000	0.59220
476	C2	C	0.13375	0.25000	0.84220
477	C2	C	0.38375	0.00000	0.84220
478	C2	C	0.38375	0.25000	0.59220
479	C2	C	0.36625	0.00000	0.59220
480	C2	C	0.36625	0.25000	0.84220
481	C2	C	0.11625	0.00000	0.84220
482	C2	C	0.11625	0.25000	0.59220
483	C2	C	0.36625	0.00000	0.90780
484	C2	C	0.36625	0.25000	0.65780
485	C2	C	0.11625	0.00000	0.65780
486	C2	C	0.11625	0.25000	0.90780

487	C2	C	0.13375	0.00000	0.90780
488	C2	C	0.13375	0.25000	0.65780
489	C2	C	0.38375	0.00000	0.65780
490	C2	C	0.38375	0.25000	0.90780
491	C2	C	0.09220	0.13375	0.50000
492	C2	C	0.09220	0.38375	0.75000
493	C2	C	0.34220	0.13375	0.75000
494	C2	C	0.34220	0.38375	0.50000
495	C2	C	0.09220	0.36625	0.50000
496	C2	C	0.09220	0.11625	0.75000
497	C2	C	0.34220	0.36625	0.75000
498	C2	C	0.34220	0.11625	0.50000
499	C2	C	0.40780	0.36625	0.50000
500	C2	C	0.40780	0.11625	0.75000
501	C2	C	0.15780	0.36625	0.75000
502	C2	C	0.15780	0.11625	0.50000
503	C2	C	0.40780	0.13375	0.50000
504	C2	C	0.40780	0.38375	0.75000
505	C2	C	0.15780	0.13375	0.75000
506	C2	C	0.15780	0.38375	0.50000
507	C2	C	0.00000	0.09220	0.63375
508	C2	C	0.00000	0.34220	0.88375
509	C2	C	0.25000	0.09220	0.88375
510	C2	C	0.25000	0.34220	0.63375
511	C2	C	0.00000	0.09220	0.86625
512	C2	C	0.00000	0.34220	0.61625
513	C2	C	0.25000	0.09220	0.61625
514	C2	C	0.25000	0.34220	0.86625
515	C2	C	0.00000	0.40780	0.86625
516	C2	C	0.00000	0.15780	0.61625
517	C2	C	0.25000	0.40780	0.61625
518	C2	C	0.25000	0.15780	0.86625
519	C2	C	0.00000	0.40780	0.63375
520	C2	C	0.00000	0.15780	0.88375
521	C2	C	0.25000	0.40780	0.88375
522	C2	C	0.25000	0.15780	0.63375
523	C2	C	0.00000	0.13375	0.90780
524	C2	C	0.00000	0.38375	0.65780
525	C2	C	0.25000	0.13375	0.65780
526	C2	C	0.25000	0.38375	0.90780
527	C2	C	0.00000	0.36625	0.90780
528	C2	C	0.00000	0.11625	0.65780
529	C2	C	0.25000	0.36625	0.65780
530	C2	C	0.25000	0.11625	0.90780
531	C2	C	0.00000	0.36625	0.59220
532	C2	C	0.00000	0.11625	0.84220
533	C2	C	0.25000	0.36625	0.84220
534	C2	C	0.25000	0.11625	0.59220
535	C2	C	0.00000	0.13375	0.59220
536	C2	C	0.00000	0.38375	0.84220
537	C2	C	0.25000	0.13375	0.84220
538	C2	C	0.25000	0.38375	0.59220
539	C2	C	0.13375	0.09220	0.50000
540	C2	C	0.13375	0.34220	0.75000

541	C2	C	0.38375	0.09220	0.75000
542	C2	C	0.38375	0.34220	0.50000
543	C2	C	0.36625	0.09220	0.50000
544	C2	C	0.36625	0.34220	0.75000
545	C2	C	0.11625	0.09220	0.75000
546	C2	C	0.11625	0.34220	0.50000
547	C2	C	0.36625	0.40780	0.50000
548	C2	C	0.36625	0.15780	0.75000
549	C2	C	0.11625	0.40780	0.75000
550	C2	C	0.11625	0.15780	0.50000
551	C2	C	0.13375	0.40780	0.50000
552	C2	C	0.13375	0.15780	0.75000
553	C2	C	0.38375	0.40780	0.75000
554	C2	C	0.38375	0.15780	0.50000
555	C2	C	0.09220	0.00000	0.86625
556	C2	C	0.09220	0.25000	0.61625
557	C2	C	0.34220	0.00000	0.61625
558	C2	C	0.34220	0.25000	0.86625
559	C2	C	0.09220	0.00000	0.63375
560	C2	C	0.09220	0.25000	0.88375
561	C2	C	0.34220	0.00000	0.88375
562	C2	C	0.34220	0.25000	0.63375
563	C2	C	0.40780	0.00000	0.63375
564	C2	C	0.40780	0.25000	0.88375
565	C2	C	0.15780	0.00000	0.88375
566	C2	C	0.15780	0.25000	0.63375
567	C2	C	0.40780	0.00000	0.86625
568	C2	C	0.40780	0.25000	0.61625
569	C2	C	0.15780	0.00000	0.61625
570	C2	C	0.15780	0.25000	0.86625
571	C1	C	0.07680	0.00000	0.92320
572	C1	C	0.07680	0.25000	0.67320
573	C1	C	0.32680	0.00000	0.67320
574	C1	C	0.32680	0.25000	0.92320
575	C1	C	0.42320	0.00000	0.92320
576	C1	C	0.42320	0.25000	0.67320
577	C1	C	0.17320	0.00000	0.67320
578	C1	C	0.17320	0.25000	0.92320
579	C1	C	0.42320	0.00000	0.57680
580	C1	C	0.42320	0.25000	0.82680
581	C1	C	0.17320	0.00000	0.82680
582	C1	C	0.17320	0.25000	0.57680
583	C1	C	0.07680	0.00000	0.57680
584	C1	C	0.07680	0.25000	0.82680
585	C1	C	0.32680	0.00000	0.82680
586	C1	C	0.32680	0.25000	0.57680
587	C1	C	0.42320	0.07680	0.50000
588	C1	C	0.42320	0.32680	0.75000
589	C1	C	0.17320	0.07680	0.75000
590	C1	C	0.17320	0.32680	0.50000
591	C1	C	0.42320	0.42320	0.50000
592	C1	C	0.42320	0.17320	0.75000
593	C1	C	0.17320	0.42320	0.75000
594	C1	C	0.17320	0.17320	0.50000

595	C1	C	0.07680	0.42320	0.50000
596	C1	C	0.07680	0.17320	0.75000
597	C1	C	0.32680	0.42320	0.75000
598	C1	C	0.32680	0.17320	0.50000
599	C1	C	0.07680	0.07680	0.50000
600	C1	C	0.07680	0.32680	0.75000
601	C1	C	0.32680	0.07680	0.75000
602	C1	C	0.32680	0.32680	0.50000
603	C1	C	0.00000	0.42320	0.57680
604	C1	C	0.00000	0.17320	0.82680
605	C1	C	0.25000	0.42320	0.82680
606	C1	C	0.25000	0.17320	0.57680
607	C1	C	0.00000	0.42320	0.92320
608	C1	C	0.00000	0.17320	0.67320
609	C1	C	0.25000	0.42320	0.67320
610	C1	C	0.25000	0.17320	0.92320
611	C1	C	0.00000	0.07680	0.92320
612	C1	C	0.00000	0.32680	0.67320
613	C1	C	0.25000	0.07680	0.67320
614	C1	C	0.25000	0.32680	0.92320
615	C1	C	0.00000	0.07680	0.57680
616	C1	C	0.00000	0.32680	0.82680
617	C1	C	0.25000	0.07680	0.82680
618	C1	C	0.25000	0.32680	0.57680
619	C3	C	0.10250	0.00000	0.60250
620	C3	C	0.10250	0.25000	0.85250
621	C3	C	0.35250	0.00000	0.85250
622	C3	C	0.35250	0.25000	0.60250
623	C3	C	0.39750	0.00000	0.60250
624	C3	C	0.39750	0.25000	0.85250
625	C3	C	0.14750	0.00000	0.85250
626	C3	C	0.14750	0.25000	0.60250
627	C3	C	0.39750	0.00000	0.89750
628	C3	C	0.39750	0.25000	0.64750
629	C3	C	0.14750	0.00000	0.64750
630	C3	C	0.14750	0.25000	0.89750
631	C3	C	0.10250	0.00000	0.89750
632	C3	C	0.10250	0.25000	0.64750
633	C3	C	0.35250	0.00000	0.64750
634	C3	C	0.35250	0.25000	0.89750
635	C3	C	0.10250	0.10250	0.50000
636	C3	C	0.10250	0.35250	0.75000
637	C3	C	0.35250	0.10250	0.75000
638	C3	C	0.35250	0.35250	0.50000
639	C3	C	0.10250	0.39750	0.50000
640	C3	C	0.10250	0.14750	0.75000
641	C3	C	0.35250	0.39750	0.75000
642	C3	C	0.35250	0.14750	0.50000
643	C3	C	0.39750	0.39750	0.50000
644	C3	C	0.39750	0.14750	0.75000
645	C3	C	0.14750	0.39750	0.75000
646	C3	C	0.14750	0.14750	0.50000
647	C3	C	0.39750	0.10250	0.50000
648	C3	C	0.39750	0.35250	0.75000

649	C3	C	0.14750	0.10250	0.75000
650	C3	C	0.14750	0.35250	0.50000
651	C3	C	0.00000	0.10250	0.60250
652	C3	C	0.00000	0.35250	0.85250
653	C3	C	0.25000	0.10250	0.85250
654	C3	C	0.25000	0.35250	0.60250
655	C3	C	0.00000	0.10250	0.89750
656	C3	C	0.00000	0.35250	0.64750
657	C3	C	0.25000	0.10250	0.64750
658	C3	C	0.25000	0.35250	0.89750
659	C3	C	0.00000	0.39750	0.89750
660	C3	C	0.00000	0.14750	0.64750
661	C3	C	0.25000	0.39750	0.64750
662	C3	C	0.25000	0.14750	0.89750
663	C3	C	0.00000	0.39750	0.60250
664	C3	C	0.00000	0.14750	0.85250
665	C3	C	0.25000	0.39750	0.85250
666	C3	C	0.25000	0.14750	0.60250
667	H1	H	0.13926	0.49997	0.56655
668	H1	H	0.13926	0.24997	0.81655
669	H1	H	0.38926	0.49997	0.81655
670	H1	H	0.38926	0.24997	0.56655
671	H1	H	0.36074	0.00003	0.56655
672	H1	H	0.36074	0.25004	0.81655
673	H1	H	0.11074	0.00003	0.81655
674	H1	H	0.11074	0.25004	0.56655
675	H1	H	0.36074	0.49996	0.93345
676	H1	H	0.36074	0.24997	0.68345
677	H1	H	0.11074	0.49997	0.68345
678	H1	H	0.11074	0.24997	0.93346
679	H1	H	0.13926	0.00003	0.93345
680	H1	H	0.13926	0.25003	0.68346
681	H1	H	0.38926	0.00003	0.68345
682	H1	H	0.38926	0.25004	0.93346
683	H1	H	0.06655	0.13926	0.99996
684	H1	H	0.06655	0.38926	0.74997
685	H1	H	0.31655	0.13926	0.74996
686	H1	H	0.31654	0.38926	0.99997
687	H1	H	0.06655	0.36074	0.50003
688	H1	H	0.06655	0.11074	0.75004
689	H1	H	0.31655	0.36074	0.75003
690	H1	H	0.31655	0.11074	0.50004
691	H1	H	0.43345	0.36074	0.99996
692	H1	H	0.43345	0.11074	0.74997
693	H1	H	0.18345	0.36074	0.74996
694	H1	H	0.18345	0.11074	0.99997
695	H1	H	0.43345	0.13926	0.50003
696	H1	H	0.43345	0.38926	0.75004
697	H1	H	0.18345	0.13926	0.75003
698	H1	H	0.18345	0.38926	0.50004
699	H1	H	0.49997	0.06655	0.63926
700	H1	H	0.49996	0.31655	0.88926
701	H1	H	0.24997	0.06655	0.88926
702	H1	H	0.24996	0.31655	0.63926

703	H1	H	0.00003	0.06655	0.86074
704	H1	H	0.00003	0.31655	0.61074
705	H1	H	0.25003	0.06655	0.61074
706	H1	H	0.25004	0.31655	0.86074
707	H1	H	0.49997	0.43345	0.86074
708	H1	H	0.49997	0.18345	0.61074
709	H1	H	0.24997	0.43345	0.61074
710	H1	H	0.24997	0.18345	0.86074
711	H1	H	0.00003	0.43345	0.63926
712	H1	H	0.00003	0.18345	0.88926
713	H1	H	0.25004	0.43345	0.88926
714	H1	H	0.25003	0.18346	0.63926
715	H1	H	0.00003	0.13926	0.93345
716	H1	H	0.00003	0.38926	0.68345
717	H1	H	0.25003	0.13926	0.68345
718	H1	H	0.25004	0.38926	0.93345
719	H1	H	0.49996	0.36074	0.93345
720	H1	H	0.49997	0.11074	0.68345
721	H1	H	0.24997	0.36074	0.68345
722	H1	H	0.24997	0.11074	0.93345
723	H1	H	0.00003	0.36074	0.56655
724	H1	H	0.00003	0.11074	0.81655
725	H1	H	0.25004	0.36074	0.81655
726	H1	H	0.25003	0.11074	0.56654
727	H1	H	0.49997	0.13926	0.56655
728	H1	H	0.49997	0.38926	0.81655
729	H1	H	0.24997	0.13926	0.81655
730	H1	H	0.24997	0.38926	0.56654
731	H1	H	0.13926	0.06655	0.99997
732	H1	H	0.13926	0.31655	0.74996
733	H1	H	0.38926	0.06655	0.74997
734	H1	H	0.38926	0.31655	0.99996
735	H1	H	0.36074	0.06655	0.50003
736	H1	H	0.36074	0.31655	0.75003
737	H1	H	0.11074	0.06655	0.75003
738	H1	H	0.11074	0.31655	0.50003
739	H1	H	0.36074	0.43345	0.99997
740	H1	H	0.36074	0.18345	0.74997
741	H1	H	0.11074	0.43345	0.74997
742	H1	H	0.11074	0.18345	0.99996
743	H1	H	0.13926	0.43345	0.50004
744	H1	H	0.13926	0.18346	0.75003
745	H1	H	0.38926	0.43345	0.75004
746	H1	H	0.38926	0.18346	0.50003
747	H1	H	0.06655	0.00003	0.86074
748	H1	H	0.06655	0.25004	0.61074
749	H1	H	0.31655	0.00003	0.61074
750	H1	H	0.31655	0.25004	0.86074
751	H1	H	0.06655	0.49996	0.63926
752	H1	H	0.06655	0.24997	0.88926
753	H1	H	0.31655	0.49996	0.88926
754	H1	H	0.31655	0.24997	0.63926
755	H1	H	0.43345	0.00003	0.63926
756	H1	H	0.43345	0.25004	0.88926

757	H1	H	0.18345	0.00003	0.88926
758	H1	H	0.18345	0.25003	0.63926
759	H1	H	0.43345	0.49996	0.86074
760	H1	H	0.43345	0.24997	0.61074
761	H1	H	0.18345	0.49996	0.61074
762	H1	H	0.18345	0.24997	0.86074
763	H2	H	0.20825	0.29176	0.95824
764	H2	H	0.29174	0.20825	0.95823
765	H2	H	0.20827	0.20823	0.54177
766	H2	H	0.29179	0.29168	0.54178
767	H2	H	0.45823	0.29173	0.70823
768	H2	H	0.45824	0.20825	0.79176
769	H2	H	0.04174	0.29176	0.79177
770	H2	H	0.04173	0.20823	0.70823
771	H2	H	0.29175	0.45824	0.70824
772	H2	H	0.20824	0.45824	0.79176
773	H2	H	0.20823	0.04172	0.70822
774	H2	H	0.29177	0.04173	0.79178
775	H2	H	0.04178	0.04177	0.54173
776	H2	H	0.45823	0.45824	0.54174
777	H2	H	0.45823	0.04176	0.95825
778	H2	H	0.04176	0.45824	0.95824
779	O1	O	0.08524	0.00000	0.54765
780	O1	O	0.08524	0.25000	0.79765
781	O1	O	0.33524	0.00000	0.79765
782	O1	O	0.33524	0.25000	0.54765
783	O1	O	0.41476	0.00000	0.54765
784	O1	O	0.41476	0.25000	0.79765
785	O1	O	0.16476	0.00000	0.79765
786	O1	O	0.16476	0.25000	0.54765
787	O1	O	0.41476	0.00000	0.95235
788	O1	O	0.41476	0.25000	0.70235
789	O1	O	0.16476	0.00000	0.70235
790	O1	O	0.16476	0.25000	0.95235
791	O1	O	0.08524	0.00000	0.95235
792	O1	O	0.08524	0.25000	0.70235
793	O1	O	0.33524	0.00000	0.70235
794	O1	O	0.33524	0.25000	0.95235
795	O1	O	0.04765	0.08524	0.50000
796	O1	O	0.04765	0.33524	0.75000
797	O1	O	0.29765	0.08524	0.75000
798	O1	O	0.29765	0.33524	0.50000
799	O1	O	0.04765	0.41476	0.50000
800	O1	O	0.04765	0.16476	0.75000
801	O1	O	0.29765	0.41476	0.75000
802	O1	O	0.29765	0.16476	0.50000
803	O1	O	0.45235	0.41476	0.50000
804	O1	O	0.45235	0.16476	0.75000
805	O1	O	0.20235	0.41476	0.75000
806	O1	O	0.20235	0.16476	0.50000
807	O1	O	0.45235	0.08524	0.50000
808	O1	O	0.45235	0.33524	0.75000
809	O1	O	0.20235	0.08524	0.75000
810	O1	O	0.20235	0.33524	0.50000

811	O1	O	0.00000	0.04765	0.58524
812	O1	O	0.00000	0.29765	0.83524
813	O1	O	0.25000	0.04765	0.83524
814	O1	O	0.25000	0.29765	0.58524
815	O1	O	0.00000	0.04765	0.91476
816	O1	O	0.00000	0.29765	0.66476
817	O1	O	0.25000	0.04765	0.66476
818	O1	O	0.25000	0.29765	0.91476
819	O1	O	0.00000	0.45235	0.91476
820	O1	O	0.00000	0.20235	0.66476
821	O1	O	0.25000	0.45235	0.66476
822	O1	O	0.25000	0.20235	0.91476
823	O1	O	0.00000	0.45235	0.58524
824	O1	O	0.00000	0.20235	0.83524
825	O1	O	0.25000	0.45235	0.83524
826	O1	O	0.25000	0.20235	0.58524
827	O1	O	0.00000	0.08524	0.95235
828	O1	O	0.00000	0.33524	0.70235
829	O1	O	0.25000	0.08524	0.70235
830	O1	O	0.25000	0.33524	0.95235
831	O1	O	0.00000	0.41476	0.95235
832	O1	O	0.00000	0.16476	0.70235
833	O1	O	0.25000	0.41476	0.70235
834	O1	O	0.25000	0.16476	0.95235
835	O1	O	0.00000	0.41476	0.54765
836	O1	O	0.00000	0.16476	0.79765
837	O1	O	0.25000	0.41476	0.79765
838	O1	O	0.25000	0.16476	0.54765
839	O1	O	0.00000	0.08524	0.54765
840	O1	O	0.00000	0.33524	0.79765
841	O1	O	0.25000	0.08524	0.79765
842	O1	O	0.25000	0.33524	0.54765
843	O1	O	0.08524	0.04765	0.50000
844	O1	O	0.08524	0.29765	0.75000
845	O1	O	0.33524	0.04765	0.75000
846	O1	O	0.33524	0.29765	0.50000
847	O1	O	0.41476	0.04765	0.50000
848	O1	O	0.41476	0.29765	0.75000
849	O1	O	0.16476	0.04765	0.75000
850	O1	O	0.16476	0.29765	0.50000
851	O1	O	0.41476	0.45235	0.50000
852	O1	O	0.41476	0.20235	0.75000
853	O1	O	0.16476	0.45235	0.75000
854	O1	O	0.16476	0.20235	0.50000
855	O1	O	0.08524	0.45235	0.50000
856	O1	O	0.08524	0.20235	0.75000
857	O1	O	0.33524	0.45235	0.75000
858	O1	O	0.33524	0.20235	0.50000
859	O1	O	0.04765	0.00000	0.91476
860	O1	O	0.04765	0.25000	0.66476
861	O1	O	0.29765	0.00000	0.66476
862	O1	O	0.29765	0.25000	0.91476
863	O1	O	0.04765	0.00000	0.58524
864	O1	O	0.04765	0.25000	0.83524

865	O1	O	0.29765	0.00000	0.83524
866	O1	O	0.29765	0.25000	0.58524
867	O1	O	0.45235	0.00000	0.58524
868	O1	O	0.45235	0.25000	0.83524
869	O1	O	0.20235	0.00000	0.83524
870	O1	O	0.20235	0.25000	0.58524
871	O1	O	0.45235	0.00000	0.91476
872	O1	O	0.45235	0.25000	0.66476
873	O1	O	0.20235	0.00000	0.66476
874	O1	O	0.20235	0.25000	0.91476
875	O2	O	0.02805	0.47195	0.97195
876	O2	O	0.02805	0.22195	0.72195
877	O2	O	0.27805	0.47195	0.72195
878	O2	O	0.27805	0.22195	0.97195
879	O2	O	0.47195	0.02805	0.97195
880	O2	O	0.47195	0.27805	0.72195
881	O2	O	0.22195	0.02805	0.72195
882	O2	O	0.22195	0.27805	0.97195
883	O2	O	0.47195	0.47195	0.52805
884	O2	O	0.47195	0.22195	0.77805
885	O2	O	0.22195	0.47195	0.77805
886	O2	O	0.22195	0.22195	0.52805
887	O2	O	0.02805	0.02805	0.52805
888	O2	O	0.02805	0.27805	0.77805
889	O2	O	0.27805	0.02805	0.77805
890	O2	O	0.27805	0.27805	0.52805
891	O1	O	0.47195	0.02805	0.52805
892	O1	O	0.47195	0.27805	0.77805
893	O1	O	0.22195	0.02805	0.77805
894	O1	O	0.22195	0.27805	0.52805
895	O1	O	0.02805	0.47195	0.52805
896	O1	O	0.02805	0.22195	0.77805
897	O1	O	0.27805	0.47195	0.77805
898	O1	O	0.27805	0.22195	0.52805
899	O1	O	0.47195	0.47195	0.97195
900	O1	O	0.47195	0.22195	0.72195
901	O1	O	0.22195	0.47195	0.72195
902	O1	O	0.22195	0.22195	0.97195
903	O1	O	0.02805	0.02805	0.97195
904	O1	O	0.02805	0.27805	0.72195
905	O1	O	0.27805	0.02805	0.72195
906	O1	O	0.27805	0.27805	0.97195
907	Zr1	Zr	0.05994	0.00000	0.50000
908	Zr1	Zr	0.05994	0.25000	0.75000
909	Zr1	Zr	0.30994	0.00000	0.75000
910	Zr1	Zr	0.30994	0.25000	0.50000
911	Zr1	Zr	0.44006	0.00000	0.50000
912	Zr1	Zr	0.44006	0.25000	0.75000
913	Zr1	Zr	0.19006	0.00000	0.75000
914	Zr1	Zr	0.19006	0.25000	0.50000
915	Zr1	Zr	0.00000	0.05994	0.50000
916	Zr1	Zr	0.00000	0.30994	0.75000
917	Zr1	Zr	0.25000	0.05994	0.75000
918	Zr1	Zr	0.25000	0.30994	0.50000

919	Zr1	Zr	0.00000	0.44006	0.50000
920	Zr1	Zr	0.00000	0.19006	0.75000
921	Zr1	Zr	0.25000	0.44006	0.75000
922	Zr1	Zr	0.25000	0.19006	0.50000
923	Zr1	Zr	0.00000	0.00000	0.55994
924	Zr1	Zr	0.00000	0.25000	0.80994
925	Zr1	Zr	0.25000	0.00000	0.80994
926	Zr1	Zr	0.25000	0.25000	0.55994
927	Zr1	Zr	0.00000	0.00000	0.94006
928	Zr1	Zr	0.00000	0.25000	0.69006
929	Zr1	Zr	0.25000	0.00000	0.69006
930	Zr1	Zr	0.25000	0.25000	0.94006
931	C2	C	0.13375	0.50000	0.09220
932	C2	C	0.13375	0.75000	0.34220
933	C2	C	0.38375	0.50000	0.34220
934	C2	C	0.38375	0.75000	0.09220
935	C2	C	0.36625	0.50000	0.09220
936	C2	C	0.36625	0.75000	0.34220
937	C2	C	0.11625	0.50000	0.34220
938	C2	C	0.11625	0.75000	0.09220
939	C2	C	0.36625	0.50000	0.40780
940	C2	C	0.36625	0.75000	0.15780
941	C2	C	0.11625	0.50000	0.15780
942	C2	C	0.11625	0.75000	0.40780
943	C2	C	0.13375	0.50000	0.40780
944	C2	C	0.13375	0.75000	0.15780
945	C2	C	0.38375	0.50000	0.15780
946	C2	C	0.38375	0.75000	0.40780
947	C2	C	0.09220	0.63375	0.00000
948	C2	C	0.09220	0.88375	0.25000
949	C2	C	0.34220	0.63375	0.25000
950	C2	C	0.34220	0.88375	0.00000
951	C2	C	0.09220	0.86625	0.00000
952	C2	C	0.09220	0.61625	0.25000
953	C2	C	0.34220	0.86625	0.25000
954	C2	C	0.34220	0.61625	0.00000
955	C2	C	0.40780	0.86625	0.00000
956	C2	C	0.40780	0.61625	0.25000
957	C2	C	0.15780	0.86625	0.25000
958	C2	C	0.15780	0.61625	0.00000
959	C2	C	0.40780	0.63375	0.00000
960	C2	C	0.40780	0.88375	0.25000
961	C2	C	0.15780	0.63375	0.25000
962	C2	C	0.15780	0.88375	0.00000
963	C2	C	0.00000	0.59220	0.13375
964	C2	C	0.00000	0.84220	0.38375
965	C2	C	0.25000	0.59220	0.38375
966	C2	C	0.25000	0.84220	0.13375
967	C2	C	0.00000	0.59220	0.36625
968	C2	C	0.00000	0.84220	0.11625
969	C2	C	0.25000	0.59220	0.11625
970	C2	C	0.25000	0.84220	0.36625
971	C2	C	0.00000	0.90780	0.36625
972	C2	C	0.00000	0.65780	0.11625

973	C2	C	0.25000	0.90780	0.11625
974	C2	C	0.25000	0.65780	0.36625
975	C2	C	0.00000	0.90780	0.13375
976	C2	C	0.00000	0.65780	0.38375
977	C2	C	0.25000	0.90780	0.38375
978	C2	C	0.25000	0.65780	0.13375
979	C2	C	0.00000	0.63375	0.40780
980	C2	C	0.00000	0.88375	0.15780
981	C2	C	0.25000	0.63375	0.15780
982	C2	C	0.25000	0.88375	0.40780
983	C2	C	0.00000	0.86625	0.40780
984	C2	C	0.00000	0.61625	0.15780
985	C2	C	0.25000	0.86625	0.15780
986	C2	C	0.25000	0.61625	0.40780
987	C2	C	0.00000	0.86625	0.09220
988	C2	C	0.00000	0.61625	0.34220
989	C2	C	0.25000	0.86625	0.34220
990	C2	C	0.25000	0.61625	0.09220
991	C2	C	0.00000	0.63375	0.09220
992	C2	C	0.00000	0.88375	0.34220
993	C2	C	0.25000	0.63375	0.34220
994	C2	C	0.25000	0.88375	0.09220
995	C2	C	0.13375	0.59220	0.00000
996	C2	C	0.13375	0.84220	0.25000
997	C2	C	0.38375	0.59220	0.25000
998	C2	C	0.38375	0.84220	0.00000
999	C2	C	0.36625	0.59220	0.00000
1000	C2	C	0.36625	0.84220	0.25000
1001	C2	C	0.11625	0.59220	0.25000
1002	C2	C	0.11625	0.84220	0.00000
1003	C2	C	0.36625	0.90780	0.00000
1004	C2	C	0.36625	0.65780	0.25000
1005	C2	C	0.11625	0.90780	0.25000
1006	C2	C	0.11625	0.65780	0.00000
1007	C2	C	0.13375	0.90780	0.00000
1008	C2	C	0.13375	0.65780	0.25000
1009	C2	C	0.38375	0.90780	0.25000
1010	C2	C	0.38375	0.65780	0.00000
1011	C2	C	0.09220	0.50000	0.36625
1012	C2	C	0.09220	0.75000	0.11625
1013	C2	C	0.34220	0.50000	0.11625
1014	C2	C	0.34220	0.75000	0.36625
1015	C2	C	0.09220	0.50000	0.13375
1016	C2	C	0.09220	0.75000	0.38375
1017	C2	C	0.34220	0.50000	0.38375
1018	C2	C	0.34220	0.75000	0.13375
1019	C2	C	0.40780	0.50000	0.13375
1020	C2	C	0.40780	0.75000	0.38375
1021	C2	C	0.15780	0.50000	0.38375
1022	C2	C	0.15780	0.75000	0.13375
1023	C2	C	0.40780	0.50000	0.36625
1024	C2	C	0.40780	0.75000	0.11625
1025	C2	C	0.15780	0.50000	0.11625
1026	C2	C	0.15780	0.75000	0.36625

1027	C1	C	0.07680	0.50000	0.42320
1028	C1	C	0.07680	0.75000	0.17320
1029	C1	C	0.32680	0.50000	0.17320
1030	C1	C	0.32680	0.75000	0.42320
1031	C1	C	0.42320	0.50000	0.42320
1032	C1	C	0.42320	0.75000	0.17320
1033	C1	C	0.17320	0.50000	0.17320
1034	C1	C	0.17320	0.75000	0.42320
1035	C1	C	0.42320	0.50000	0.07680
1036	C1	C	0.42320	0.75000	0.32680
1037	C1	C	0.17320	0.50000	0.32680
1038	C1	C	0.17320	0.75000	0.07680
1039	C1	C	0.07680	0.50000	0.07680
1040	C1	C	0.07680	0.75000	0.32680
1041	C1	C	0.32680	0.50000	0.32680
1042	C1	C	0.32680	0.75000	0.07680
1043	C1	C	0.42320	0.57680	0.00000
1044	C1	C	0.42320	0.82680	0.25000
1045	C1	C	0.17320	0.57680	0.25000
1046	C1	C	0.17320	0.82680	0.00000
1047	C1	C	0.42320	0.92320	0.00000
1048	C1	C	0.42320	0.67320	0.25000
1049	C1	C	0.17320	0.92320	0.25000
1050	C1	C	0.17320	0.67320	0.00000
1051	C1	C	0.07680	0.92320	0.00000
1052	C1	C	0.07680	0.67320	0.25000
1053	C1	C	0.32680	0.92320	0.25000
1054	C1	C	0.32680	0.67320	0.00000
1055	C1	C	0.07680	0.57680	0.00000
1056	C1	C	0.07680	0.82680	0.25000
1057	C1	C	0.32680	0.57680	0.25000
1058	C1	C	0.32680	0.82680	0.00000
1059	C1	C	0.00000	0.92320	0.07680
1060	C1	C	0.00000	0.67320	0.32680
1061	C1	C	0.25000	0.92320	0.32680
1062	C1	C	0.25000	0.67320	0.07680
1063	C1	C	0.00000	0.92320	0.42320
1064	C1	C	0.00000	0.67320	0.17320
1065	C1	C	0.25000	0.92320	0.17320
1066	C1	C	0.25000	0.67320	0.42320
1067	C1	C	0.00000	0.57680	0.42320
1068	C1	C	0.00000	0.82680	0.17320
1069	C1	C	0.25000	0.57680	0.17320
1070	C1	C	0.25000	0.82680	0.42320
1071	C1	C	0.00000	0.57680	0.07680
1072	C1	C	0.00000	0.82680	0.32680
1073	C1	C	0.25000	0.57680	0.32680
1074	C1	C	0.25000	0.82680	0.07680
1075	C3	C	0.10250	0.50000	0.10250
1076	C3	C	0.10250	0.75000	0.35250
1077	C3	C	0.35250	0.50000	0.35250
1078	C3	C	0.35250	0.75000	0.10250
1079	C3	C	0.39750	0.50000	0.10250
1080	C3	C	0.39750	0.75000	0.35250

1081	C3	C	0.14750	0.50000	0.35250
1082	C3	C	0.14750	0.75000	0.10250
1083	C3	C	0.39750	0.50000	0.39750
1084	C3	C	0.39750	0.75000	0.14750
1085	C3	C	0.14750	0.50000	0.14750
1086	C3	C	0.14750	0.75000	0.39750
1087	C3	C	0.10250	0.50000	0.39750
1088	C3	C	0.10250	0.75000	0.14750
1089	C3	C	0.35250	0.50000	0.14750
1090	C3	C	0.35250	0.75000	0.39750
1091	C3	C	0.10250	0.60250	0.00000
1092	C3	C	0.10250	0.85250	0.25000
1093	C3	C	0.35250	0.60250	0.25000
1094	C3	C	0.35250	0.85250	0.00000
1095	C3	C	0.10250	0.89750	0.00000
1096	C3	C	0.10250	0.64750	0.25000
1097	C3	C	0.35250	0.89750	0.25000
1098	C3	C	0.35250	0.64750	0.00000
1099	C3	C	0.39750	0.89750	0.00000
1100	C3	C	0.39750	0.64750	0.25000
1101	C3	C	0.14750	0.89750	0.25000
1102	C3	C	0.14750	0.64750	0.00000
1103	C3	C	0.39750	0.60250	0.00000
1104	C3	C	0.39750	0.85250	0.25000
1105	C3	C	0.14750	0.60250	0.25000
1106	C3	C	0.14750	0.85250	0.00000
1107	C3	C	0.00000	0.60250	0.10250
1108	C3	C	0.00000	0.85250	0.35250
1109	C3	C	0.25000	0.60250	0.35250
1110	C3	C	0.25000	0.85250	0.10250
1111	C3	C	0.00000	0.60250	0.39750
1112	C3	C	0.00000	0.85250	0.14750
1113	C3	C	0.25000	0.60250	0.14750
1114	C3	C	0.25000	0.85250	0.39750
1115	C3	C	0.00000	0.89750	0.39750
1116	C3	C	0.00000	0.64750	0.14750
1117	C3	C	0.25000	0.89750	0.14750
1118	C3	C	0.25000	0.64750	0.39750
1119	C3	C	0.00000	0.89750	0.10250
1120	C3	C	0.00000	0.64750	0.35250
1121	C3	C	0.25000	0.89750	0.35250
1122	C3	C	0.25000	0.64750	0.10250
1123	H1	H	0.13926	0.99997	0.06655
1124	H1	H	0.13926	0.74997	0.31655
1125	H1	H	0.38926	0.99997	0.31655
1126	H1	H	0.38926	0.74997	0.06655
1127	H1	H	0.36074	0.50003	0.06655
1128	H1	H	0.36074	0.75004	0.31655
1129	H1	H	0.11074	0.50003	0.31655
1130	H1	H	0.11074	0.75004	0.06655
1131	H1	H	0.36074	0.99996	0.43345
1132	H1	H	0.36074	0.74997	0.18345
1133	H1	H	0.11074	0.99997	0.18345
1134	H1	H	0.11074	0.74997	0.43346

1135	H1	H	0.13926	0.50003	0.43345
1136	H1	H	0.13926	0.75003	0.18346
1137	H1	H	0.38926	0.50003	0.18345
1138	H1	H	0.38926	0.75004	0.43346
1139	H1	H	0.06655	0.63926	0.49996
1140	H1	H	0.06655	0.88926	0.24997
1141	H1	H	0.31655	0.63926	0.24996
1142	H1	H	0.31654	0.88926	0.49997
1143	H1	H	0.06655	0.86074	0.00003
1144	H1	H	0.06655	0.61074	0.25004
1145	H1	H	0.31655	0.86074	0.25003
1146	H1	H	0.31655	0.61074	0.00004
1147	H1	H	0.43345	0.86074	0.49996
1148	H1	H	0.43345	0.61074	0.24997
1149	H1	H	0.18345	0.86074	0.24996
1150	H1	H	0.18345	0.61074	0.49997
1151	H1	H	0.43345	0.63926	0.00003
1152	H1	H	0.43345	0.88926	0.25004
1153	H1	H	0.18345	0.63926	0.25003
1154	H1	H	0.18345	0.88926	0.00004
1155	H1	H	0.49997	0.56655	0.13926
1156	H1	H	0.49996	0.81655	0.38926
1157	H1	H	0.24997	0.56655	0.38926
1158	H1	H	0.24996	0.81655	0.13926
1159	H1	H	0.00003	0.56655	0.36074
1160	H1	H	0.00003	0.81655	0.11074
1161	H1	H	0.25003	0.56655	0.11074
1162	H1	H	0.25004	0.81655	0.36074
1163	H1	H	0.49997	0.93345	0.36074
1164	H1	H	0.49997	0.68345	0.11074
1165	H1	H	0.24997	0.93345	0.11074
1166	H1	H	0.24997	0.68345	0.36074
1167	H1	H	0.00003	0.93345	0.13926
1168	H1	H	0.00003	0.68345	0.38926
1169	H1	H	0.25004	0.93345	0.38926
1170	H1	H	0.25003	0.68346	0.13926
1171	H1	H	0.00003	0.63926	0.43345
1172	H1	H	0.00003	0.88926	0.18345
1173	H1	H	0.25003	0.63926	0.18345
1174	H1	H	0.25004	0.88926	0.43345
1175	H1	H	0.49996	0.86074	0.43345
1176	H1	H	0.49997	0.61074	0.18345
1177	H1	H	0.24997	0.86074	0.18345
1178	H1	H	0.24997	0.61074	0.43345
1179	H1	H	0.00003	0.86074	0.06655
1180	H1	H	0.00003	0.61074	0.31655
1181	H1	H	0.25004	0.86074	0.31655
1182	H1	H	0.25003	0.61074	0.06654
1183	H1	H	0.49997	0.63926	0.06655
1184	H1	H	0.49997	0.88926	0.31655
1185	H1	H	0.24997	0.63926	0.31655
1186	H1	H	0.24997	0.88926	0.06654
1187	H1	H	0.13926	0.56655	0.49997
1188	H1	H	0.13926	0.81655	0.24996

1189	H1	H	0.38926	0.56655	0.24997
1190	H1	H	0.38926	0.81655	0.49996
1191	H1	H	0.36074	0.56655	0.00003
1192	H1	H	0.36074	0.81655	0.25003
1193	H1	H	0.11074	0.56655	0.25003
1194	H1	H	0.11074	0.81655	0.00003
1195	H1	H	0.36074	0.93345	0.49997
1196	H1	H	0.36074	0.68345	0.24997
1197	H1	H	0.11074	0.93345	0.24997
1198	H1	H	0.11074	0.68345	0.49996
1199	H1	H	0.13926	0.93345	0.00004
1200	H1	H	0.13926	0.68346	0.25003
1201	H1	H	0.38926	0.93345	0.25004
1202	H1	H	0.38926	0.68346	0.00003
1203	H1	H	0.06655	0.50003	0.36074
1204	H1	H	0.06655	0.75004	0.11074
1205	H1	H	0.31655	0.50003	0.11074
1206	H1	H	0.31655	0.75004	0.36074
1207	H1	H	0.06655	0.99996	0.13926
1208	H1	H	0.06655	0.74997	0.38926
1209	H1	H	0.31655	0.99996	0.38926
1210	H1	H	0.31655	0.74997	0.13926
1211	H1	H	0.43345	0.50003	0.13926
1212	H1	H	0.43345	0.75004	0.38926
1213	H1	H	0.18345	0.50003	0.38926
1214	H1	H	0.18345	0.75003	0.13926
1215	H1	H	0.43345	0.99996	0.36074
1216	H1	H	0.43345	0.74997	0.11074
1217	H1	H	0.18345	0.99996	0.11074
1218	H1	H	0.18345	0.74997	0.36074
1219	H2	H	0.20825	0.79176	0.45824
1220	H2	H	0.29174	0.70825	0.45823
1221	H2	H	0.20827	0.70823	0.04177
1222	H2	H	0.29179	0.79168	0.04178
1223	H2	H	0.45823	0.79173	0.20823
1224	H2	H	0.45824	0.70825	0.29176
1225	H2	H	0.04174	0.79176	0.29177
1226	H2	H	0.04173	0.70823	0.20823
1227	H2	H	0.29175	0.95824	0.20824
1228	H2	H	0.20824	0.95824	0.29176
1229	H2	H	0.20823	0.54172	0.20822
1230	H2	H	0.29177	0.54173	0.29178
1231	H2	H	0.04178	0.54177	0.04173
1232	H2	H	0.45823	0.95824	0.04174
1233	H2	H	0.45823	0.54176	0.45825
1234	H2	H	0.04176	0.95824	0.45824
1235	O1	O	0.08524	0.50000	0.04765
1236	O1	O	0.08524	0.75000	0.29765
1237	O1	O	0.33524	0.50000	0.29765
1238	O1	O	0.33524	0.75000	0.04765
1239	O1	O	0.41476	0.50000	0.04765
1240	O1	O	0.41476	0.75000	0.29765
1241	O1	O	0.16476	0.50000	0.29765
1242	O1	O	0.16476	0.75000	0.04765

1243	O1	O	0.41476	0.50000	0.45235
1244	O1	O	0.41476	0.75000	0.20235
1245	O1	O	0.16476	0.50000	0.20235
1246	O1	O	0.16476	0.75000	0.45235
1247	O1	O	0.08524	0.50000	0.45235
1248	O1	O	0.08524	0.75000	0.20235
1249	O1	O	0.33524	0.50000	0.20235
1250	O1	O	0.33524	0.75000	0.45235
1251	O1	O	0.04765	0.58524	0.00000
1252	O1	O	0.04765	0.83524	0.25000
1253	O1	O	0.29765	0.58524	0.25000
1254	O1	O	0.29765	0.83524	0.00000
1255	O1	O	0.04765	0.91476	0.00000
1256	O1	O	0.04765	0.66476	0.25000
1257	O1	O	0.29765	0.91476	0.25000
1258	O1	O	0.29765	0.66476	0.00000
1259	O1	O	0.45235	0.91476	0.00000
1260	O1	O	0.45235	0.66476	0.25000
1261	O1	O	0.20235	0.91476	0.25000
1262	O1	O	0.20235	0.66476	0.00000
1263	O1	O	0.45235	0.58524	0.00000
1264	O1	O	0.45235	0.83524	0.25000
1265	O1	O	0.20235	0.58524	0.25000
1266	O1	O	0.20235	0.83524	0.00000
1267	O1	O	0.00000	0.54765	0.08524
1268	O1	O	0.00000	0.79765	0.33524
1269	O1	O	0.25000	0.54765	0.33524
1270	O1	O	0.25000	0.79765	0.08524
1271	O1	O	0.00000	0.54765	0.41476
1272	O1	O	0.00000	0.79765	0.16476
1273	O1	O	0.25000	0.54765	0.16476
1274	O1	O	0.25000	0.79765	0.41476
1275	O1	O	0.00000	0.95235	0.41476
1276	O1	O	0.00000	0.70235	0.16476
1277	O1	O	0.25000	0.95235	0.16476
1278	O1	O	0.25000	0.70235	0.41476
1279	O1	O	0.00000	0.95235	0.08524
1280	O1	O	0.00000	0.70235	0.33524
1281	O1	O	0.25000	0.95235	0.33524
1282	O1	O	0.25000	0.70235	0.08524
1283	O1	O	0.00000	0.58524	0.45235
1284	O1	O	0.00000	0.83524	0.20235
1285	O1	O	0.25000	0.58524	0.20235
1286	O1	O	0.25000	0.83524	0.45235
1287	O1	O	0.00000	0.91476	0.45235
1288	O1	O	0.00000	0.66476	0.20235
1289	O1	O	0.25000	0.91476	0.20235
1290	O1	O	0.25000	0.66476	0.45235
1291	O1	O	0.00000	0.91476	0.04765
1292	O1	O	0.00000	0.66476	0.29765
1293	O1	O	0.25000	0.91476	0.29765
1294	O1	O	0.25000	0.66476	0.04765
1295	O1	O	0.00000	0.58524	0.04765
1296	O1	O	0.00000	0.83524	0.29765

1297	O1	O	0.25000	0.58524	0.29765
1298	O1	O	0.25000	0.83524	0.04765
1299	O1	O	0.08524	0.54765	0.00000
1300	O1	O	0.08524	0.79765	0.25000
1301	O1	O	0.33524	0.54765	0.25000
1302	O1	O	0.33524	0.79765	0.00000
1303	O1	O	0.41476	0.54765	0.00000
1304	O1	O	0.41476	0.79765	0.25000
1305	O1	O	0.16476	0.54765	0.25000
1306	O1	O	0.16476	0.79765	0.00000
1307	O1	O	0.41476	0.95235	0.00000
1308	O1	O	0.41476	0.70235	0.25000
1309	O1	O	0.16476	0.95235	0.25000
1310	O1	O	0.16476	0.70235	0.00000
1311	O1	O	0.08524	0.95235	0.00000
1312	O1	O	0.08524	0.70235	0.25000
1313	O1	O	0.33524	0.95235	0.25000
1314	O1	O	0.33524	0.70235	0.00000
1315	O1	O	0.04765	0.50000	0.41476
1316	O1	O	0.04765	0.75000	0.16476
1317	O1	O	0.29765	0.50000	0.16476
1318	O1	O	0.29765	0.75000	0.41476
1319	O1	O	0.04765	0.50000	0.08524
1320	O1	O	0.04765	0.75000	0.33524
1321	O1	O	0.29765	0.50000	0.33524
1322	O1	O	0.29765	0.75000	0.08524
1323	O1	O	0.45235	0.50000	0.08524
1324	O1	O	0.45235	0.75000	0.33524
1325	O1	O	0.20235	0.50000	0.33524
1326	O1	O	0.20235	0.75000	0.08524
1327	O1	O	0.45235	0.50000	0.41476
1328	O1	O	0.45235	0.75000	0.16476
1329	O1	O	0.20235	0.50000	0.16476
1330	O1	O	0.20235	0.75000	0.41476
1331	O2	O	0.02805	0.97195	0.47195
1332	O2	O	0.02805	0.72195	0.22195
1333	O2	O	0.27805	0.97195	0.22195
1334	O2	O	0.27805	0.72195	0.47195
1335	O2	O	0.47195	0.52805	0.47195
1336	O2	O	0.47195	0.77805	0.22195
1337	O2	O	0.22195	0.52805	0.22195
1338	O2	O	0.22195	0.77805	0.47195
1339	O2	O	0.47195	0.97195	0.02805
1340	O2	O	0.47195	0.72195	0.27805
1341	O2	O	0.22195	0.97195	0.27805
1342	O2	O	0.22195	0.72195	0.02805
1343	O2	O	0.02805	0.52805	0.02805
1344	O2	O	0.02805	0.77805	0.27805
1345	O2	O	0.27805	0.52805	0.27805
1346	O2	O	0.27805	0.77805	0.02805
1347	O1	O	0.47195	0.52805	0.02805
1348	O1	O	0.47195	0.77805	0.27805
1349	O1	O	0.22195	0.52805	0.27805
1350	O1	O	0.22195	0.77805	0.02805

1351	O1	O	0.02805	0.97195	0.02805
1352	O1	O	0.02805	0.72195	0.27805
1353	O1	O	0.27805	0.97195	0.27805
1354	O1	O	0.27805	0.72195	0.02805
1355	O1	O	0.47195	0.97195	0.47195
1356	O1	O	0.47195	0.72195	0.22195
1357	O1	O	0.22195	0.97195	0.22195
1358	O1	O	0.22195	0.72195	0.47195
1359	O1	O	0.02805	0.52805	0.47195
1360	O1	O	0.02805	0.77805	0.22195
1361	O1	O	0.27805	0.52805	0.22195
1362	O1	O	0.27805	0.77805	0.47195
1363	Zr1	Zr	0.05994	0.50000	0.00000
1364	Zr1	Zr	0.05994	0.75000	0.25000
1365	Zr1	Zr	0.30994	0.50000	0.25000
1366	Zr1	Zr	0.30994	0.75000	0.00000
1367	Zr1	Zr	0.44006	0.50000	0.00000
1368	Zr1	Zr	0.44006	0.75000	0.25000
1369	Zr1	Zr	0.19006	0.50000	0.25000
1370	Zr1	Zr	0.19006	0.75000	0.00000
1371	Zr1	Zr	0.00000	0.55994	0.00000
1372	Zr1	Zr	0.00000	0.80994	0.25000
1373	Zr1	Zr	0.25000	0.55994	0.25000
1374	Zr1	Zr	0.25000	0.80994	0.00000
1375	Zr1	Zr	0.00000	0.94006	0.00000
1376	Zr1	Zr	0.00000	0.69006	0.25000
1377	Zr1	Zr	0.25000	0.94006	0.25000
1378	Zr1	Zr	0.25000	0.69006	0.00000
1379	Zr1	Zr	0.00000	0.50000	0.05994
1380	Zr1	Zr	0.00000	0.75000	0.30994
1381	Zr1	Zr	0.25000	0.50000	0.30994
1382	Zr1	Zr	0.25000	0.75000	0.05994
1383	Zr1	Zr	0.00000	0.50000	0.44006
1384	Zr1	Zr	0.00000	0.75000	0.19006
1385	Zr1	Zr	0.25000	0.50000	0.19006
1386	Zr1	Zr	0.25000	0.75000	0.44006
1387	C2	C	0.13375	0.50000	0.59220
1388	C2	C	0.13375	0.75000	0.84220
1389	C2	C	0.38375	0.50000	0.84220
1390	C2	C	0.38375	0.75000	0.59220
1391	C2	C	0.36625	0.50000	0.59220
1392	C2	C	0.36625	0.75000	0.84220
1393	C2	C	0.11625	0.50000	0.84220
1394	C2	C	0.11625	0.75000	0.59220
1395	C2	C	0.36625	0.50000	0.90780
1396	C2	C	0.36625	0.75000	0.65780
1397	C2	C	0.11625	0.50000	0.65780
1398	C2	C	0.11625	0.75000	0.90780
1399	C2	C	0.13375	0.50000	0.90780
1400	C2	C	0.13375	0.75000	0.65780
1401	C2	C	0.38375	0.50000	0.65780
1402	C2	C	0.38375	0.75000	0.90780
1403	C2	C	0.09220	0.63375	0.50000
1404	C2	C	0.09220	0.88375	0.75000

1405	C2	C	0.34220	0.63375	0.75000
1406	C2	C	0.34220	0.88375	0.50000
1407	C2	C	0.09220	0.86625	0.50000
1408	C2	C	0.09220	0.61625	0.75000
1409	C2	C	0.34220	0.86625	0.75000
1410	C2	C	0.40780	0.86625	0.50000
1411	C2	C	0.40780	0.61625	0.75000
1412	C2	C	0.15780	0.86625	0.75000
1413	C2	C	0.15780	0.61625	0.50000
1414	C2	C	0.40780	0.88375	0.75000
1415	C2	C	0.15780	0.63375	0.75000
1416	C2	C	0.15780	0.88375	0.50000
1417	C2	C	0.00000	0.59220	0.63375
1418	C2	C	0.00000	0.84220	0.88375
1419	C2	C	0.25000	0.59220	0.88375
1420	C2	C	0.25000	0.84220	0.63375
1421	C2	C	0.00000	0.59220	0.86625
1422	C2	C	0.00000	0.84220	0.61625
1423	C2	C	0.25000	0.59220	0.61625
1424	C2	C	0.25000	0.84220	0.86625
1425	C2	C	0.00000	0.90780	0.86625
1426	C2	C	0.00000	0.65780	0.61625
1427	C2	C	0.25000	0.90780	0.61625
1428	C2	C	0.25000	0.65780	0.86625
1429	C2	C	0.00000	0.90780	0.63375
1430	C2	C	0.00000	0.65780	0.88375
1431	C2	C	0.25000	0.90780	0.88375
1432	C2	C	0.25000	0.65780	0.63375
1433	C2	C	0.00000	0.63375	0.90780
1434	C2	C	0.00000	0.88375	0.65780
1435	C2	C	0.25000	0.63375	0.65780
1436	C2	C	0.25000	0.88375	0.90780
1437	C2	C	0.00000	0.86625	0.90780
1438	C2	C	0.00000	0.61625	0.65780
1439	C2	C	0.25000	0.86625	0.65780
1440	C2	C	0.25000	0.61625	0.90780
1441	C2	C	0.00000	0.86625	0.59220
1442	C2	C	0.00000	0.61625	0.84220
1443	C2	C	0.25000	0.86625	0.84220
1444	C2	C	0.25000	0.61625	0.59220
1445	C2	C	0.00000	0.63375	0.59220
1446	C2	C	0.00000	0.88375	0.84220
1447	C2	C	0.25000	0.63375	0.84220
1448	C2	C	0.25000	0.88375	0.59220
1449	C2	C	0.13375	0.59220	0.50000
1450	C2	C	0.13375	0.84220	0.75000
1451	C2	C	0.38375	0.59220	0.75000
1452	C2	C	0.38375	0.84220	0.50000
1453	C2	C	0.36625	0.84220	0.75000
1454	C2	C	0.11625	0.59220	0.75000
1455	C2	C	0.11625	0.84220	0.50000
1456	C2	C	0.36625	0.90780	0.50000
1457	C2	C	0.36625	0.65780	0.75000
1458	C2	C	0.11625	0.90780	0.75000

1459	C2	C	0.11625	0.65780	0.50000
1460	C2	C	0.13375	0.90780	0.50000
1461	C2	C	0.13375	0.65780	0.75000
1462	C2	C	0.38375	0.90780	0.75000
1463	C2	C	0.09220	0.50000	0.86625
1464	C2	C	0.09220	0.75000	0.61625
1465	C2	C	0.34220	0.50000	0.61625
1466	C2	C	0.34220	0.75000	0.86625
1467	C2	C	0.09220	0.50000	0.63375
1468	C2	C	0.09220	0.75000	0.88375
1469	C2	C	0.34220	0.50000	0.88375
1470	C2	C	0.34220	0.75000	0.63375
1471	C2	C	0.40780	0.50000	0.63375
1472	C2	C	0.40780	0.75000	0.88375
1473	C2	C	0.15780	0.50000	0.88375
1474	C2	C	0.15780	0.75000	0.63375
1475	C2	C	0.40780	0.50000	0.86625
1476	C2	C	0.40780	0.75000	0.61625
1477	C2	C	0.15780	0.50000	0.61625
1478	C2	C	0.15780	0.75000	0.86625
1479	C1	C	0.07680	0.50000	0.92320
1480	C1	C	0.07680	0.75000	0.67320
1481	C1	C	0.32680	0.50000	0.67320
1482	C1	C	0.32680	0.75000	0.92320
1483	C1	C	0.42320	0.50000	0.92320
1484	C1	C	0.42320	0.75000	0.67320
1485	C1	C	0.17320	0.50000	0.67320
1486	C1	C	0.17320	0.75000	0.92320
1487	C1	C	0.42320	0.50000	0.57680
1488	C1	C	0.42320	0.75000	0.82680
1489	C1	C	0.17320	0.50000	0.82680
1490	C1	C	0.17320	0.75000	0.57680
1491	C1	C	0.07680	0.50000	0.57680
1492	C1	C	0.07680	0.75000	0.82680
1493	C1	C	0.32680	0.50000	0.82680
1494	C1	C	0.32680	0.75000	0.57680
1495	C1	C	0.42320	0.82680	0.75000
1496	C1	C	0.17320	0.57680	0.75000
1497	C1	C	0.17320	0.82680	0.50000
1498	C1	C	0.42320	0.92320	0.50000
1499	C1	C	0.42320	0.67320	0.75000
1500	C1	C	0.17320	0.92320	0.75000
1501	C1	C	0.17320	0.67320	0.50000
1502	C1	C	0.07680	0.92320	0.50000
1503	C1	C	0.07680	0.67320	0.75000
1504	C1	C	0.32680	0.92320	0.75000
1505	C1	C	0.07680	0.57680	0.50000
1506	C1	C	0.07680	0.82680	0.75000
1507	C1	C	0.32680	0.57680	0.75000
1508	C1	C	0.32680	0.82680	0.50000
1509	C1	C	0.00000	0.92320	0.57680
1510	C1	C	0.00000	0.67320	0.82680
1511	C1	C	0.25000	0.92320	0.82680
1512	C1	C	0.25000	0.67320	0.57680

1513	C1	C	0.00000	0.92320	0.92320
1514	C1	C	0.00000	0.67320	0.67320
1515	C1	C	0.25000	0.92320	0.67320
1516	C1	C	0.25000	0.67320	0.92320
1517	C1	C	0.00000	0.57680	0.92320
1518	C1	C	0.00000	0.82680	0.67320
1519	C1	C	0.25000	0.57680	0.67320
1520	C1	C	0.25000	0.82680	0.92320
1521	C1	C	0.00000	0.57680	0.57680
1522	C1	C	0.00000	0.82680	0.82680
1523	C1	C	0.25000	0.57680	0.82680
1524	C1	C	0.25000	0.82680	0.57680
1525	C3	C	0.10250	0.50000	0.60250
1526	C3	C	0.10250	0.75000	0.85250
1527	C3	C	0.35250	0.50000	0.85250
1528	C3	C	0.35250	0.75000	0.60250
1529	C3	C	0.39750	0.50000	0.60250
1530	C3	C	0.39750	0.75000	0.85250
1531	C3	C	0.14750	0.50000	0.85250
1532	C3	C	0.14750	0.75000	0.60250
1533	C3	C	0.39750	0.50000	0.89750
1534	C3	C	0.39750	0.75000	0.64750
1535	C3	C	0.14750	0.50000	0.64750
1536	C3	C	0.14750	0.75000	0.89750
1537	C3	C	0.10250	0.50000	0.89750
1538	C3	C	0.10250	0.75000	0.64750
1539	C3	C	0.35250	0.50000	0.64750
1540	C3	C	0.35250	0.75000	0.89750
1541	C3	C	0.10250	0.60250	0.50000
1542	C3	C	0.10250	0.85250	0.75000
1543	C3	C	0.35250	0.60250	0.75000
1544	C3	C	0.35250	0.85250	0.50000
1545	C3	C	0.10250	0.89750	0.50000
1546	C3	C	0.10250	0.64750	0.75000
1547	C3	C	0.35250	0.89750	0.75000
1548	C3	C	0.39750	0.89750	0.50000
1549	C3	C	0.39750	0.64750	0.75000
1550	C3	C	0.14750	0.89750	0.75000
1551	C3	C	0.14750	0.64750	0.50000
1552	C3	C	0.39750	0.85250	0.75000
1553	C3	C	0.14750	0.60250	0.75000
1554	C3	C	0.14750	0.85250	0.50000
1555	C3	C	0.00000	0.60250	0.60250
1556	C3	C	0.00000	0.85250	0.85250
1557	C3	C	0.25000	0.60250	0.85250
1558	C3	C	0.25000	0.85250	0.60250
1559	C3	C	0.00000	0.60250	0.89750
1560	C3	C	0.00000	0.85250	0.64750
1561	C3	C	0.25000	0.60250	0.64750
1562	C3	C	0.25000	0.85250	0.89750
1563	C3	C	0.00000	0.89750	0.89750
1564	C3	C	0.00000	0.64750	0.64750
1565	C3	C	0.25000	0.89750	0.64750
1566	C3	C	0.25000	0.64750	0.89750

1567	C3	C	0.00000	0.89750	0.60250
1568	C3	C	0.00000	0.64750	0.85250
1569	C3	C	0.25000	0.89750	0.85250
1570	C3	C	0.25000	0.64750	0.60250
1571	H1	H	0.13926	0.99997	0.56655
1572	H1	H	0.13926	0.74997	0.81655
1573	H1	H	0.38926	0.99997	0.81655
1574	H1	H	0.38926	0.74997	0.56655
1575	H1	H	0.36074	0.50003	0.56655
1576	H1	H	0.36074	0.75004	0.81655
1577	H1	H	0.11074	0.50003	0.81655
1578	H1	H	0.11074	0.75004	0.56655
1579	H1	H	0.36074	0.99996	0.93345
1580	H1	H	0.36074	0.74997	0.68345
1581	H1	H	0.11074	0.99997	0.68345
1582	H1	H	0.11074	0.74997	0.93346
1583	H1	H	0.13926	0.50003	0.93345
1584	H1	H	0.13926	0.75003	0.68346
1585	H1	H	0.38926	0.50003	0.68345
1586	H1	H	0.38926	0.75004	0.93346
1587	H1	H	0.06655	0.63926	0.99996
1588	H1	H	0.06655	0.88926	0.74997
1589	H1	H	0.31655	0.63926	0.74996
1590	H1	H	0.31654	0.88926	0.99997
1591	H1	H	0.06655	0.86074	0.50003
1592	H1	H	0.06655	0.61074	0.75004
1593	H1	H	0.31655	0.86074	0.75003
1594	H1	H	0.43345	0.86074	0.99996
1595	H1	H	0.43345	0.61074	0.74997
1596	H1	H	0.18345	0.86074	0.74996
1597	H1	H	0.18345	0.61074	0.99997
1598	H1	H	0.43345	0.88926	0.75004
1599	H1	H	0.18345	0.63926	0.75003
1600	H1	H	0.18345	0.88926	0.50004
1601	H1	H	0.49997	0.56655	0.63926
1602	H1	H	0.49996	0.81655	0.88926
1603	H1	H	0.24997	0.56655	0.88926
1604	H1	H	0.24996	0.81655	0.63926
1605	H1	H	0.00003	0.56655	0.86074
1606	H1	H	0.00003	0.81655	0.61074
1607	H1	H	0.25003	0.56655	0.61074
1608	H1	H	0.25004	0.81655	0.86074
1609	H1	H	0.49997	0.93345	0.86074
1610	H1	H	0.49997	0.68345	0.61074
1611	H1	H	0.24997	0.93345	0.61074
1612	H1	H	0.24997	0.68345	0.86074
1613	H1	H	0.00003	0.93345	0.63926
1614	H1	H	0.00003	0.68345	0.88926
1615	H1	H	0.25004	0.93345	0.88926
1616	H1	H	0.25003	0.68346	0.63926
1617	H1	H	0.00003	0.63926	0.93345
1618	H1	H	0.00003	0.88926	0.68345
1619	H1	H	0.25003	0.63926	0.68345
1620	H1	H	0.25004	0.88926	0.93345

1621	H1	H	0.49996	0.86074	0.93345
1622	H1	H	0.49997	0.61074	0.68345
1623	H1	H	0.24997	0.86074	0.68345
1624	H1	H	0.24997	0.61074	0.93345
1625	H1	H	0.00003	0.86074	0.56655
1626	H1	H	0.00003	0.61074	0.81655
1627	H1	H	0.25004	0.86074	0.81655
1628	H1	H	0.25003	0.61074	0.56654
1629	H1	H	0.49997	0.63926	0.56655
1630	H1	H	0.49997	0.88926	0.81655
1631	H1	H	0.24997	0.63926	0.81655
1632	H1	H	0.24997	0.88926	0.56654
1633	H1	H	0.13926	0.56655	0.99997
1634	H1	H	0.13926	0.81655	0.74996
1635	H1	H	0.38926	0.56655	0.74997
1636	H1	H	0.38926	0.81655	0.99996
1637	H1	H	0.36074	0.81655	0.75003
1638	H1	H	0.11074	0.56655	0.75003
1639	H1	H	0.11074	0.81655	0.50003
1640	H1	H	0.36074	0.93345	0.99997
1641	H1	H	0.36074	0.68345	0.74997
1642	H1	H	0.11074	0.93345	0.74997
1643	H1	H	0.11074	0.68345	0.99996
1644	H1	H	0.13926	0.93345	0.50004
1645	H1	H	0.13926	0.68346	0.75003
1646	H1	H	0.38926	0.93345	0.75004
1647	H1	H	0.06655	0.50003	0.86074
1648	H1	H	0.06655	0.75004	0.61074
1649	H1	H	0.31655	0.50003	0.61074
1650	H1	H	0.31655	0.75004	0.86074
1651	H1	H	0.06655	0.99996	0.63926
1652	H1	H	0.06655	0.74997	0.88926
1653	H1	H	0.31655	0.99996	0.88926
1654	H1	H	0.31655	0.74997	0.63926
1655	H1	H	0.43345	0.50003	0.63926
1656	H1	H	0.43345	0.75004	0.88926
1657	H1	H	0.18345	0.50003	0.88926
1658	H1	H	0.18345	0.75003	0.63926
1659	H1	H	0.43345	0.99996	0.86074
1660	H1	H	0.43345	0.74997	0.61074
1661	H1	H	0.18345	0.99996	0.61074
1662	H1	H	0.18345	0.74997	0.86074
1663	H2	H	0.20825	0.79176	0.95824
1664	H2	H	0.29174	0.70825	0.95823
1665	H2	H	0.20827	0.70823	0.54177
1666	H2	H	0.29179	0.79168	0.54178
1667	H2	H	0.45823	0.79173	0.70823
1668	H2	H	0.45824	0.70825	0.79176
1669	H2	H	0.04174	0.79176	0.79177
1670	H2	H	0.04173	0.70823	0.70823
1671	H2	H	0.29175	0.95824	0.70824
1672	H2	H	0.20824	0.95824	0.79176
1673	H2	H	0.20823	0.54172	0.70822
1674	H2	H	0.29177	0.54173	0.79178

1675	H2	H	0.04178	0.54177	0.54173
1676	H2	H	0.45823	0.95824	0.54174
1677	H2	H	0.45823	0.54176	0.95825
1678	H2	H	0.04176	0.95824	0.95824
1679	O1	O	0.08524	0.50000	0.54765
1680	O1	O	0.08524	0.75000	0.79765
1681	O1	O	0.33524	0.50000	0.79765
1682	O1	O	0.33524	0.75000	0.54765
1683	O1	O	0.41476	0.50000	0.54765
1684	O1	O	0.41476	0.75000	0.79765
1685	O1	O	0.16476	0.50000	0.79765
1686	O1	O	0.16476	0.75000	0.54765
1687	O1	O	0.41476	0.50000	0.95235
1688	O1	O	0.41476	0.75000	0.70235
1689	O1	O	0.16476	0.50000	0.70235
1690	O1	O	0.16476	0.75000	0.95235
1691	O1	O	0.08524	0.50000	0.95235
1692	O1	O	0.08524	0.75000	0.70235
1693	O1	O	0.33524	0.50000	0.70235
1694	O1	O	0.33524	0.75000	0.95235
1695	O1	O	0.04765	0.58524	0.50000
1696	O1	O	0.04765	0.83524	0.75000
1697	O1	O	0.29765	0.58524	0.75000
1698	O1	O	0.29765	0.83524	0.50000
1699	O1	O	0.04765	0.91476	0.50000
1700	O1	O	0.04765	0.66476	0.75000
1701	O1	O	0.29765	0.91476	0.75000
1702	O3	O	0.29765	0.66476	0.50000
1703	O1	O	0.45235	0.91476	0.50000
1704	O1	O	0.45235	0.66476	0.75000
1705	O1	O	0.20235	0.91476	0.75000
1706	O1	O	0.20235	0.66476	0.50000
1707	O3	O	0.45235	0.58524	0.50000
1708	O1	O	0.45235	0.83524	0.75000
1709	O1	O	0.20235	0.58524	0.75000
1710	O1	O	0.20235	0.83524	0.50000
1711	O1	O	0.00000	0.54765	0.58524
1712	O1	O	0.00000	0.79765	0.83524
1713	O1	O	0.25000	0.54765	0.83524
1714	O1	O	0.25000	0.79765	0.58524
1715	O1	O	0.00000	0.54765	0.91476
1716	O1	O	0.00000	0.79765	0.66476
1717	O1	O	0.25000	0.54765	0.66476
1718	O1	O	0.25000	0.79765	0.91476
1719	O1	O	0.00000	0.95235	0.91476
1720	O1	O	0.00000	0.70235	0.66476
1721	O1	O	0.25000	0.95235	0.66476
1722	O1	O	0.25000	0.70235	0.91476
1723	O1	O	0.00000	0.95235	0.58524
1724	O1	O	0.00000	0.70235	0.83524
1725	O1	O	0.25000	0.95235	0.83524
1726	O1	O	0.25000	0.70235	0.58524
1727	O1	O	0.00000	0.58524	0.95235
1728	O1	O	0.00000	0.83524	0.70235

1729	O1	O	0.25000	0.58524	0.70235
1730	O1	O	0.25000	0.83524	0.95235
1731	O1	O	0.00000	0.91476	0.95235
1732	O1	O	0.00000	0.66476	0.70235
1733	O1	O	0.25000	0.91476	0.70235
1734	O1	O	0.25000	0.66476	0.95235
1735	O1	O	0.00000	0.91476	0.54765
1736	O1	O	0.00000	0.66476	0.79765
1737	O1	O	0.25000	0.91476	0.79765
1738	O1	O	0.25000	0.66476	0.54765
1739	O1	O	0.00000	0.58524	0.54765
1740	O1	O	0.00000	0.83524	0.79765
1741	O1	O	0.25000	0.58524	0.79765
1742	O1	O	0.25000	0.83524	0.54765
1743	O1	O	0.08524	0.54765	0.50000
1744	O1	O	0.08524	0.79765	0.75000
1745	O1	O	0.33524	0.54765	0.75000
1746	O1	O	0.33524	0.79765	0.50000
1747	O3	O	0.41476	0.54765	0.50000
1748	O1	O	0.41476	0.79765	0.75000
1749	O1	O	0.16476	0.54765	0.75000
1750	O1	O	0.16476	0.79765	0.50000
1751	O1	O	0.41476	0.95235	0.50000
1752	O1	O	0.41476	0.70235	0.75000
1753	O1	O	0.16476	0.95235	0.75000
1754	O1	O	0.16476	0.70235	0.50000
1755	O1	O	0.08524	0.95235	0.50000
1756	O1	O	0.08524	0.70235	0.75000
1757	O1	O	0.33524	0.95235	0.75000
1758	O3	O	0.33524	0.70235	0.50000
1759	O1	O	0.04765	0.50000	0.91476
1760	O1	O	0.04765	0.75000	0.66476
1761	O1	O	0.29765	0.50000	0.66476
1762	O1	O	0.29765	0.75000	0.91476
1763	O1	O	0.04765	0.50000	0.58524
1764	O1	O	0.04765	0.75000	0.83524
1765	O1	O	0.29765	0.50000	0.83524
1766	O1	O	0.29765	0.75000	0.58524
1767	O1	O	0.45235	0.50000	0.58524
1768	O1	O	0.45235	0.75000	0.83524
1769	O1	O	0.20235	0.50000	0.83524
1770	O1	O	0.20235	0.75000	0.58524
1771	O1	O	0.45235	0.50000	0.91476
1772	O1	O	0.45235	0.75000	0.66476
1773	O1	O	0.20235	0.50000	0.66476
1774	O1	O	0.20235	0.75000	0.91476
1775	O2	O	0.02805	0.97195	0.97195
1776	O2	O	0.02805	0.72195	0.72195
1777	O2	O	0.27805	0.97195	0.72195
1778	O2	O	0.27805	0.72195	0.97195
1779	O2	O	0.47195	0.52805	0.97195
1780	O2	O	0.47195	0.77805	0.72195
1781	O2	O	0.22195	0.52805	0.72195
1782	O2	O	0.22195	0.77805	0.97195

1783	O2	O	0.47195	0.97195	0.52805
1784	O2	O	0.47195	0.72195	0.77805
1785	O2	O	0.22195	0.97195	0.77805
1786	O2	O	0.22195	0.72195	0.52805
1787	O2	O	0.02805	0.52805	0.52805
1788	O2	O	0.02805	0.77805	0.77805
1789	O2	O	0.27805	0.52805	0.77805
1790	O2	O	0.27805	0.77805	0.52805
1791	O1	O	0.47195	0.52805	0.52805
1792	O1	O	0.47195	0.77805	0.77805
1793	O1	O	0.22195	0.52805	0.77805
1794	O1	O	0.22195	0.77805	0.52805
1795	O1	O	0.02805	0.97195	0.52805
1796	O1	O	0.02805	0.72195	0.77805
1797	O1	O	0.27805	0.97195	0.77805
1798	O1	O	0.27805	0.72195	0.52805
1799	O1	O	0.47195	0.97195	0.97195
1800	O1	O	0.47195	0.72195	0.72195
1801	O1	O	0.22195	0.97195	0.72195
1802	O1	O	0.22195	0.72195	0.97195
1803	O1	O	0.02805	0.52805	0.97195
1804	O1	O	0.02805	0.77805	0.72195
1805	O1	O	0.27805	0.52805	0.72195
1806	O1	O	0.27805	0.77805	0.97195
1807	Zr1	Zr	0.05994	0.50000	0.50000
1808	Zr1	Zr	0.05994	0.75000	0.75000
1809	Zr1	Zr	0.30994	0.50000	0.75000
1810	Zr1	Zr	0.30994	0.75000	0.50000
1811	Zr1	Zr	0.44006	0.50000	0.50000
1812	Zr1	Zr	0.44006	0.75000	0.75000
1813	Zr1	Zr	0.19006	0.50000	0.75000
1814	Zr1	Zr	0.19006	0.75000	0.50000
1815	Zr1	Zr	0.00000	0.55994	0.50000
1816	Zr1	Zr	0.00000	0.80994	0.75000
1817	Zr1	Zr	0.25000	0.55994	0.75000
1818	Zr1	Zr	0.25000	0.80994	0.50000
1819	Zr1	Zr	0.00000	0.94006	0.50000
1820	Zr1	Zr	0.00000	0.69006	0.75000
1821	Zr1	Zr	0.25000	0.94006	0.75000
1822	Zr1	Zr	0.25000	0.69006	0.50000
1823	Zr1	Zr	0.00000	0.50000	0.55994
1824	Zr1	Zr	0.00000	0.75000	0.80994
1825	Zr1	Zr	0.25000	0.50000	0.80994
1826	Zr1	Zr	0.25000	0.75000	0.55994
1827	Zr1	Zr	0.00000	0.50000	0.94006
1828	Zr1	Zr	0.00000	0.75000	0.69006
1829	Zr1	Zr	0.25000	0.50000	0.69006
1830	Zr1	Zr	0.25000	0.75000	0.94006
1831	C2	C	0.63375	0.00000	0.09220
1832	C2	C	0.63375	0.25000	0.34220
1833	C2	C	0.88375	0.00000	0.34220
1834	C2	C	0.88375	0.25000	0.09220
1835	C2	C	0.86625	0.00000	0.09220
1836	C2	C	0.86625	0.25000	0.34220

1837	C2	C	0.61625	0.00000	0.34220
1838	C2	C	0.61625	0.25000	0.09220
1839	C2	C	0.86625	0.00000	0.40780
1840	C2	C	0.86625	0.25000	0.15780
1841	C2	C	0.61625	0.00000	0.15780
1842	C2	C	0.61625	0.25000	0.40780
1843	C2	C	0.63375	0.00000	0.40780
1844	C2	C	0.63375	0.25000	0.15780
1845	C2	C	0.88375	0.00000	0.15780
1846	C2	C	0.88375	0.25000	0.40780
1847	C2	C	0.59220	0.13375	0.00000
1848	C2	C	0.84220	0.13375	0.25000
1849	C2	C	0.84220	0.38375	0.00000
1850	C2	C	0.59220	0.36625	0.00000
1851	C2	C	0.59220	0.11625	0.25000
1852	C2	C	0.84220	0.36625	0.25000
1853	C2	C	0.84220	0.11625	0.00000
1854	C2	C	0.90780	0.36625	0.00000
1855	C2	C	0.90780	0.11625	0.25000
1856	C2	C	0.65780	0.11625	0.00000
1857	C2	C	0.90780	0.13375	0.00000
1858	C2	C	0.90780	0.38375	0.25000
1859	C2	C	0.65780	0.13375	0.25000
1860	C2	C	0.65780	0.38375	0.00000
1861	C2	C	0.50000	0.09220	0.13375
1862	C2	C	0.50000	0.34220	0.38375
1863	C2	C	0.75000	0.09220	0.38375
1864	C2	C	0.75000	0.34220	0.13375
1865	C2	C	0.50000	0.09220	0.36625
1866	C2	C	0.50000	0.34220	0.11625
1867	C2	C	0.75000	0.09220	0.11625
1868	C2	C	0.75000	0.34220	0.36625
1869	C2	C	0.50000	0.40780	0.36625
1870	C2	C	0.50000	0.15780	0.11625
1871	C2	C	0.75000	0.40780	0.11625
1872	C2	C	0.75000	0.15780	0.36625
1873	C2	C	0.50000	0.40780	0.13375
1874	C2	C	0.50000	0.15780	0.38375
1875	C2	C	0.75000	0.40780	0.38375
1876	C2	C	0.75000	0.15780	0.13375
1877	C2	C	0.50000	0.13375	0.40780
1878	C2	C	0.50000	0.38375	0.15780
1879	C2	C	0.75000	0.13375	0.15780
1880	C2	C	0.75000	0.38375	0.40780
1881	C2	C	0.50000	0.36625	0.40780
1882	C2	C	0.50000	0.11625	0.15780
1883	C2	C	0.75000	0.36625	0.15780
1884	C2	C	0.75000	0.11625	0.40780
1885	C2	C	0.50000	0.36625	0.09220
1886	C2	C	0.50000	0.11625	0.34220
1887	C2	C	0.75000	0.36625	0.34220
1888	C2	C	0.75000	0.11625	0.09220
1889	C2	C	0.50000	0.13375	0.09220
1890	C2	C	0.50000	0.38375	0.34220

1891	C2	C	0.75000	0.13375	0.34220
1892	C2	C	0.75000	0.38375	0.09220
1893	C2	C	0.63375	0.09220	0.00000
1894	C2	C	0.88375	0.09220	0.25000
1895	C2	C	0.88375	0.34220	0.00000
1896	C2	C	0.86625	0.09220	0.00000
1897	C2	C	0.86625	0.34220	0.25000
1898	C2	C	0.61625	0.09220	0.25000
1899	C2	C	0.61625	0.34220	0.00000
1900	C2	C	0.86625	0.40780	0.00000
1901	C2	C	0.86625	0.15780	0.25000
1902	C2	C	0.61625	0.15780	0.00000
1903	C2	C	0.63375	0.40780	0.00000
1904	C2	C	0.63375	0.15780	0.25000
1905	C2	C	0.88375	0.40780	0.25000
1906	C2	C	0.88375	0.15780	0.00000
1907	C2	C	0.59220	0.00000	0.36625
1908	C2	C	0.59220	0.25000	0.11625
1909	C2	C	0.84220	0.00000	0.11625
1910	C2	C	0.84220	0.25000	0.36625
1911	C2	C	0.59220	0.00000	0.13375
1912	C2	C	0.59220	0.25000	0.38375
1913	C2	C	0.84220	0.00000	0.38375
1914	C2	C	0.84220	0.25000	0.13375
1915	C2	C	0.90780	0.00000	0.13375
1916	C2	C	0.90780	0.25000	0.38375
1917	C2	C	0.65780	0.00000	0.38375
1918	C2	C	0.65780	0.25000	0.13375
1919	C2	C	0.90780	0.00000	0.36625
1920	C2	C	0.90780	0.25000	0.11625
1921	C2	C	0.65780	0.00000	0.11625
1922	C2	C	0.65780	0.25000	0.36625
1923	C1	C	0.57680	0.00000	0.42320
1924	C1	C	0.57680	0.25000	0.17320
1925	C1	C	0.82680	0.00000	0.17320
1926	C1	C	0.82680	0.25000	0.42320
1927	C1	C	0.92320	0.00000	0.42320
1928	C1	C	0.92320	0.25000	0.17320
1929	C1	C	0.67320	0.00000	0.17320
1930	C1	C	0.67320	0.25000	0.42320
1931	C1	C	0.92320	0.00000	0.07680
1932	C1	C	0.92320	0.25000	0.32680
1933	C1	C	0.67320	0.00000	0.32680
1934	C1	C	0.67320	0.25000	0.07680
1935	C1	C	0.57680	0.00000	0.07680
1936	C1	C	0.57680	0.25000	0.32680
1937	C1	C	0.82680	0.00000	0.32680
1938	C1	C	0.82680	0.25000	0.07680
1939	C1	C	0.92320	0.07680	0.00000
1940	C1	C	0.92320	0.32680	0.25000
1941	C1	C	0.67320	0.07680	0.25000
1942	C1	C	0.67320	0.32680	0.00000
1943	C1	C	0.92320	0.42320	0.00000
1944	C1	C	0.92320	0.17320	0.25000

1945	C1	C	0.67320	0.17320	0.00000
1946	C1	C	0.57680	0.42320	0.00000
1947	C1	C	0.57680	0.17320	0.25000
1948	C1	C	0.82680	0.42320	0.25000
1949	C1	C	0.82680	0.17320	0.00000
1950	C1	C	0.57680	0.07680	0.00000
1951	C1	C	0.82680	0.07680	0.25000
1952	C1	C	0.82680	0.32680	0.00000
1953	C1	C	0.50000	0.42320	0.07680
1954	C1	C	0.50000	0.17320	0.32680
1955	C1	C	0.75000	0.42320	0.32680
1956	C1	C	0.75000	0.17320	0.07680
1957	C1	C	0.50000	0.42320	0.42320
1958	C1	C	0.50000	0.17320	0.17320
1959	C1	C	0.75000	0.42320	0.17320
1960	C1	C	0.75000	0.17320	0.42320
1961	C1	C	0.50000	0.07680	0.42320
1962	C1	C	0.50000	0.32680	0.17320
1963	C1	C	0.75000	0.07680	0.17320
1964	C1	C	0.75000	0.32680	0.42320
1965	C1	C	0.50000	0.07680	0.07680
1966	C1	C	0.50000	0.32680	0.32680
1967	C1	C	0.75000	0.07680	0.32680
1968	C1	C	0.75000	0.32680	0.07680
1969	C3	C	0.60250	0.00000	0.10250
1970	C3	C	0.60250	0.25000	0.35250
1971	C3	C	0.85250	0.00000	0.35250
1972	C3	C	0.85250	0.25000	0.10250
1973	C3	C	0.89750	0.00000	0.10250
1974	C3	C	0.89750	0.25000	0.35250
1975	C3	C	0.64750	0.00000	0.35250
1976	C3	C	0.64750	0.25000	0.10250
1977	C3	C	0.89750	0.00000	0.39750
1978	C3	C	0.89750	0.25000	0.14750
1979	C3	C	0.64750	0.00000	0.14750
1980	C3	C	0.64750	0.25000	0.39750
1981	C3	C	0.60250	0.00000	0.39750
1982	C3	C	0.60250	0.25000	0.14750
1983	C3	C	0.85250	0.00000	0.14750
1984	C3	C	0.85250	0.25000	0.39750
1985	C3	C	0.60250	0.10250	0.00000
1986	C3	C	0.85250	0.10250	0.25000
1987	C3	C	0.85250	0.35250	0.00000
1988	C3	C	0.60250	0.39750	0.00000
1989	C3	C	0.60250	0.14750	0.25000
1990	C3	C	0.85250	0.39750	0.25000
1991	C3	C	0.85250	0.14750	0.00000
1992	C3	C	0.89750	0.39750	0.00000
1993	C3	C	0.89750	0.14750	0.25000
1994	C3	C	0.64750	0.14750	0.00000
1995	C3	C	0.89750	0.10250	0.00000
1996	C3	C	0.89750	0.35250	0.25000
1997	C3	C	0.64750	0.10250	0.25000
1998	C3	C	0.64750	0.35250	0.00000

1999	C3	C	0.50000	0.10250	0.10250
2000	C3	C	0.50000	0.35250	0.35250
2001	C3	C	0.75000	0.10250	0.35250
2002	C3	C	0.75000	0.35250	0.10250
2003	C3	C	0.50000	0.10250	0.39750
2004	C3	C	0.50000	0.35250	0.14750
2005	C3	C	0.75000	0.10250	0.14750
2006	C3	C	0.75000	0.35250	0.39750
2007	C3	C	0.50000	0.39750	0.39750
2008	C3	C	0.50000	0.14750	0.14750
2009	C3	C	0.75000	0.39750	0.14750
2010	C3	C	0.75000	0.14750	0.39750
2011	C3	C	0.50000	0.39750	0.10250
2012	C3	C	0.50000	0.14750	0.35250
2013	C3	C	0.75000	0.39750	0.35250
2014	C3	C	0.75000	0.14750	0.10250
2015	H1	H	0.63926	0.49997	0.06655
2016	H1	H	0.63926	0.24997	0.31655
2017	H1	H	0.88926	0.49997	0.31655
2018	H1	H	0.88926	0.24997	0.06655
2019	H1	H	0.86074	0.00003	0.06655
2020	H1	H	0.86074	0.25004	0.31655
2021	H1	H	0.61074	0.00003	0.31655
2022	H1	H	0.61074	0.25004	0.06655
2023	H1	H	0.86074	0.49996	0.43345
2024	H1	H	0.86074	0.24997	0.18345
2025	H1	H	0.61074	0.49997	0.18345
2026	H1	H	0.61074	0.24997	0.43346
2027	H1	H	0.63926	0.00003	0.43345
2028	H1	H	0.63926	0.25003	0.18346
2029	H1	H	0.88926	0.00003	0.18345
2030	H1	H	0.88926	0.25004	0.43346
2031	H1	H	0.56655	0.13926	0.49996
2032	H1	H	0.81655	0.13926	0.24996
2033	H1	H	0.81654	0.38926	0.49997
2034	H1	H	0.56655	0.36074	0.00003
2035	H1	H	0.56655	0.11074	0.25004
2036	H1	H	0.81655	0.36074	0.25003
2037	H1	H	0.81655	0.11074	0.00004
2038	H1	H	0.93345	0.36074	0.49996
2039	H1	H	0.93345	0.11074	0.24997
2040	H1	H	0.68345	0.11074	0.49997
2041	H1	H	0.93345	0.13926	0.00003
2042	H1	H	0.93345	0.38926	0.25004
2043	H1	H	0.68345	0.13926	0.25003
2044	H1	H	0.68345	0.38926	0.00004
2045	H1	H	0.99997	0.06655	0.13926
2046	H1	H	0.99996	0.31655	0.38926
2047	H1	H	0.74997	0.06655	0.38926
2048	H1	H	0.74996	0.31655	0.13926
2049	H1	H	0.50003	0.06655	0.36074
2050	H1	H	0.50003	0.31655	0.11074
2051	H1	H	0.75003	0.06655	0.11074
2052	H1	H	0.75004	0.31655	0.36074

2053	H1	H	0.99997	0.43345	0.36074
2054	H1	H	0.99997	0.18345	0.11074
2055	H1	H	0.74997	0.43345	0.11074
2056	H1	H	0.74997	0.18345	0.36074
2057	H1	H	0.50003	0.43345	0.13926
2058	H1	H	0.50003	0.18345	0.38926
2059	H1	H	0.75004	0.43345	0.38926
2060	H1	H	0.75003	0.18346	0.13926
2061	H1	H	0.50003	0.13926	0.43345
2062	H1	H	0.50003	0.38926	0.18345
2063	H1	H	0.75003	0.13926	0.18345
2064	H1	H	0.75004	0.38926	0.43345
2065	H1	H	0.99996	0.36074	0.43345
2066	H1	H	0.99997	0.11074	0.18345
2067	H1	H	0.74997	0.36074	0.18345
2068	H1	H	0.74997	0.11074	0.43345
2069	H1	H	0.50003	0.36074	0.06655
2070	H1	H	0.50003	0.11074	0.31655
2071	H1	H	0.75004	0.36074	0.31655
2072	H1	H	0.75003	0.11074	0.06654
2073	H1	H	0.99997	0.13926	0.06655
2074	H1	H	0.99997	0.38926	0.31655
2075	H1	H	0.74997	0.13926	0.31655
2076	H1	H	0.74997	0.38926	0.06654
2077	H1	H	0.63926	0.06655	0.49997
2078	H1	H	0.88926	0.06655	0.24997
2079	H1	H	0.88926	0.31655	0.49996
2080	H1	H	0.86074	0.06655	0.00003
2081	H1	H	0.86074	0.31655	0.25003
2082	H1	H	0.61074	0.06655	0.25003
2083	H1	H	0.61074	0.31655	0.00003
2084	H1	H	0.86074	0.43345	0.49997
2085	H1	H	0.86074	0.18345	0.24997
2086	H1	H	0.61074	0.18345	0.49996
2087	H1	H	0.63926	0.43345	0.00004
2088	H1	H	0.63926	0.18346	0.25003
2089	H1	H	0.88926	0.43345	0.25004
2090	H1	H	0.88926	0.18346	0.00003
2091	H1	H	0.56655	0.00003	0.36074
2092	H1	H	0.56655	0.25004	0.11074
2093	H1	H	0.81655	0.00003	0.11074
2094	H1	H	0.81655	0.25004	0.36074
2095	H1	H	0.56655	0.49996	0.13926
2096	H1	H	0.56655	0.24997	0.38926
2097	H1	H	0.81655	0.49996	0.38926
2098	H1	H	0.81655	0.24997	0.13926
2099	H1	H	0.93345	0.00003	0.13926
2100	H1	H	0.93345	0.25004	0.38926
2101	H1	H	0.68345	0.00003	0.38926
2102	H1	H	0.68345	0.25003	0.13926
2103	H1	H	0.93345	0.49996	0.36074
2104	H1	H	0.93345	0.24997	0.11074
2105	H1	H	0.68345	0.49996	0.11074
2106	H1	H	0.68345	0.24997	0.36074

2107	H2	H	0.70825	0.29176	0.45824
2108	H2	H	0.79174	0.20825	0.45823
2109	H2	H	0.70827	0.20823	0.04177
2110	H2	H	0.79179	0.29168	0.04178
2111	H2	H	0.95823	0.29173	0.20823
2112	H2	H	0.95824	0.20825	0.29176
2113	H2	H	0.54174	0.29176	0.29177
2114	H2	H	0.54173	0.20823	0.20823
2115	H2	H	0.79175	0.45824	0.20824
2116	H2	H	0.70824	0.45824	0.29176
2117	H2	H	0.70823	0.04172	0.20822
2118	H2	H	0.79177	0.04173	0.29178
2119	H2	H	0.54178	0.04177	0.04173
2120	H2	H	0.95823	0.45824	0.04174
2121	H2	H	0.95823	0.04176	0.45825
2122	H2	H	0.54176	0.45824	0.45824
2123	O1	O	0.58524	0.00000	0.04765
2124	O1	O	0.58524	0.25000	0.29765
2125	O1	O	0.83524	0.00000	0.29765
2126	O1	O	0.83524	0.25000	0.04765
2127	O1	O	0.91476	0.00000	0.04765
2128	O1	O	0.91476	0.25000	0.29765
2129	O1	O	0.66476	0.00000	0.29765
2130	O1	O	0.66476	0.25000	0.04765
2131	O1	O	0.91476	0.00000	0.45235
2132	O1	O	0.91476	0.25000	0.20235
2133	O1	O	0.66476	0.00000	0.20235
2134	O1	O	0.66476	0.25000	0.45235
2135	O1	O	0.58524	0.00000	0.45235
2136	O1	O	0.58524	0.25000	0.20235
2137	O1	O	0.83524	0.00000	0.20235
2138	O1	O	0.83524	0.25000	0.45235
2139	O1	O	0.54765	0.08524	0.00000
2140	O3	O	0.54765	0.33524	0.25000
2141	O1	O	0.79765	0.08524	0.25000
2142	O1	O	0.79765	0.33524	0.00000
2143	O1	O	0.54765	0.41476	0.00000
2144	O1	O	0.54765	0.16476	0.25000
2145	O1	O	0.79765	0.41476	0.25000
2146	O1	O	0.79765	0.16476	0.00000
2147	O1	O	0.95235	0.41476	0.00000
2148	O1	O	0.95235	0.16476	0.25000
2149	O3	O	0.70235	0.41476	0.25000
2150	O1	O	0.70235	0.16476	0.00000
2151	O1	O	0.95235	0.08524	0.00000
2152	O1	O	0.95235	0.33524	0.25000
2153	O1	O	0.70235	0.08524	0.25000
2154	O1	O	0.70235	0.33524	0.00000
2155	O1	O	0.50000	0.04765	0.08524
2156	O1	O	0.50000	0.29765	0.33524
2157	O1	O	0.75000	0.04765	0.33524
2158	O1	O	0.75000	0.29765	0.08524
2159	O1	O	0.50000	0.04765	0.41476
2160	O1	O	0.50000	0.29765	0.16476

2161	O1	O	0.75000	0.04765	0.16476
2162	O1	O	0.75000	0.29765	0.41476
2163	O1	O	0.50000	0.45235	0.41476
2164	O1	O	0.50000	0.20235	0.16476
2165	O1	O	0.75000	0.45235	0.16476
2166	O1	O	0.75000	0.20235	0.41476
2167	O1	O	0.50000	0.45235	0.08524
2168	O1	O	0.50000	0.20235	0.33524
2169	O1	O	0.75000	0.45235	0.33524
2170	O1	O	0.75000	0.20235	0.08524
2171	O1	O	0.50000	0.08524	0.45235
2172	O1	O	0.50000	0.33524	0.20235
2173	O1	O	0.75000	0.08524	0.20235
2174	O1	O	0.75000	0.33524	0.45235
2175	O1	O	0.50000	0.41476	0.45235
2176	O1	O	0.50000	0.16476	0.20235
2177	O1	O	0.75000	0.41476	0.20235
2178	O1	O	0.75000	0.16476	0.45235
2179	O1	O	0.50000	0.41476	0.04765
2180	O1	O	0.50000	0.16476	0.29765
2181	O1	O	0.75000	0.41476	0.29765
2182	O1	O	0.75000	0.16476	0.04765
2183	O1	O	0.50000	0.08524	0.04765
2184	O1	O	0.50000	0.33524	0.29765
2185	O1	O	0.75000	0.08524	0.29765
2186	O1	O	0.75000	0.33524	0.04765
2187	O1	O	0.58524	0.04765	0.00000
2188	O3	O	0.58524	0.29765	0.25000
2189	O1	O	0.83524	0.04765	0.25000
2190	O1	O	0.83524	0.29765	0.00000
2191	O1	O	0.91476	0.04765	0.00000
2192	O1	O	0.91476	0.29765	0.25000
2193	O1	O	0.66476	0.04765	0.25000
2194	O1	O	0.66476	0.29765	0.00000
2195	O1	O	0.91476	0.45235	0.00000
2196	O1	O	0.91476	0.20235	0.25000
2197	O3	O	0.66476	0.45235	0.25000
2198	O1	O	0.66476	0.20235	0.00000
2199	O1	O	0.58524	0.45235	0.00000
2200	O1	O	0.58524	0.20235	0.25000
2201	O1	O	0.83524	0.45235	0.25000
2202	O1	O	0.83524	0.20235	0.00000
2203	O1	O	0.54765	0.00000	0.41476
2204	O1	O	0.54765	0.25000	0.16476
2205	O1	O	0.79765	0.00000	0.16476
2206	O1	O	0.79765	0.25000	0.41476
2207	O1	O	0.54765	0.00000	0.08524
2208	O1	O	0.54765	0.25000	0.33524
2209	O1	O	0.79765	0.00000	0.33524
2210	O1	O	0.79765	0.25000	0.08524
2211	O1	O	0.95235	0.00000	0.08524
2212	O1	O	0.95235	0.25000	0.33524
2213	O1	O	0.70235	0.00000	0.33524
2214	O1	O	0.70235	0.25000	0.08524

2215	O1	O	0.95235	0.00000	0.41476
2216	O1	O	0.95235	0.25000	0.16476
2217	O1	O	0.70235	0.00000	0.16476
2218	O1	O	0.70235	0.25000	0.41476
2219	O2	O	0.52805	0.47195	0.47195
2220	O2	O	0.52805	0.22195	0.22195
2221	O2	O	0.77805	0.47195	0.22195
2222	O2	O	0.77805	0.22195	0.47195
2223	O2	O	0.97195	0.02805	0.47195
2224	O2	O	0.97195	0.27805	0.22195
2225	O2	O	0.72195	0.02805	0.22195
2226	O2	O	0.72195	0.27805	0.47195
2227	O2	O	0.97195	0.47195	0.02805
2228	O2	O	0.97195	0.22195	0.27805
2229	O2	O	0.72195	0.47195	0.27805
2230	O2	O	0.72195	0.22195	0.02805
2231	O2	O	0.52805	0.02805	0.02805
2232	O2	O	0.52805	0.27805	0.27805
2233	O2	O	0.77805	0.02805	0.27805
2234	O2	O	0.77805	0.27805	0.02805
2235	O1	O	0.97195	0.02805	0.02805
2236	O1	O	0.97195	0.27805	0.27805
2237	O1	O	0.72195	0.02805	0.27805
2238	O1	O	0.72195	0.27805	0.02805
2239	O1	O	0.52805	0.47195	0.02805
2240	O1	O	0.52805	0.22195	0.27805
2241	O1	O	0.77805	0.47195	0.27805
2242	O1	O	0.77805	0.22195	0.02805
2243	O1	O	0.97195	0.47195	0.47195
2244	O1	O	0.97195	0.22195	0.22195
2245	O1	O	0.72195	0.47195	0.22195
2246	O1	O	0.72195	0.22195	0.47195
2247	O1	O	0.52805	0.02805	0.47195
2248	O1	O	0.52805	0.27805	0.22195
2249	O1	O	0.77805	0.02805	0.22195
2250	O1	O	0.77805	0.27805	0.47195
2251	Zr1	Zr	0.55994	0.00000	0.00000
2252	Zr1	Zr	0.55994	0.25000	0.25000
2253	Zr1	Zr	0.80994	0.00000	0.25000
2254	Zr1	Zr	0.80994	0.25000	0.00000
2255	Zr1	Zr	0.94006	0.00000	0.00000
2256	Zr1	Zr	0.94006	0.25000	0.25000
2257	Zr1	Zr	0.69006	0.00000	0.25000
2258	Zr1	Zr	0.69006	0.25000	0.00000
2259	Zr1	Zr	0.50000	0.05994	0.00000
2260	Zr1	Zr	0.50000	0.30994	0.25000
2261	Zr1	Zr	0.75000	0.05994	0.25000
2262	Zr1	Zr	0.75000	0.30994	0.00000
2263	Zr1	Zr	0.50000	0.44006	0.00000
2264	Zr1	Zr	0.50000	0.19006	0.25000
2265	Zr1	Zr	0.75000	0.44006	0.25000
2266	Zr1	Zr	0.75000	0.19006	0.00000
2267	Zr1	Zr	0.50000	0.00000	0.05994
2268	Zr1	Zr	0.50000	0.25000	0.30994

2269	Zr1	Zr	0.75000	0.00000	0.30994
2270	Zr1	Zr	0.75000	0.25000	0.05994
2271	Zr1	Zr	0.50000	0.00000	0.44006
2272	Zr1	Zr	0.50000	0.25000	0.19006
2273	Zr1	Zr	0.75000	0.00000	0.19006
2274	Zr1	Zr	0.75000	0.25000	0.44006
2275	C2	C	0.63375	0.00000	0.59220
2276	C2	C	0.63375	0.25000	0.84220
2277	C2	C	0.88375	0.00000	0.84220
2278	C2	C	0.88375	0.25000	0.59220
2279	C2	C	0.86625	0.00000	0.59220
2280	C2	C	0.86625	0.25000	0.84220
2281	C2	C	0.61625	0.00000	0.84220
2282	C2	C	0.61625	0.25000	0.59220
2283	C2	C	0.86625	0.00000	0.90780
2284	C2	C	0.86625	0.25000	0.65780
2285	C2	C	0.61625	0.00000	0.65780
2286	C2	C	0.61625	0.25000	0.90780
2287	C2	C	0.63375	0.00000	0.90780
2288	C2	C	0.63375	0.25000	0.65780
2289	C2	C	0.88375	0.00000	0.65780
2290	C2	C	0.88375	0.25000	0.90780
2291	C2	C	0.59220	0.13375	0.50000
2292	C2	C	0.59220	0.38375	0.75000
2293	C2	C	0.84220	0.13375	0.75000
2294	C2	C	0.84220	0.38375	0.50000
2295	C2	C	0.59220	0.36625	0.50000
2296	C2	C	0.59220	0.11625	0.75000
2297	C2	C	0.84220	0.36625	0.75000
2298	C2	C	0.84220	0.11625	0.50000
2299	C2	C	0.90780	0.36625	0.50000
2300	C2	C	0.90780	0.11625	0.75000
2301	C2	C	0.65780	0.36625	0.75000
2302	C2	C	0.65780	0.11625	0.50000
2303	C2	C	0.90780	0.13375	0.50000
2304	C2	C	0.90780	0.38375	0.75000
2305	C2	C	0.65780	0.13375	0.75000
2306	C2	C	0.65780	0.38375	0.50000
2307	C2	C	0.50000	0.09220	0.63375
2308	C2	C	0.50000	0.34220	0.88375
2309	C2	C	0.75000	0.09220	0.88375
2310	C2	C	0.75000	0.34220	0.63375
2311	C2	C	0.50000	0.09220	0.86625
2312	C2	C	0.50000	0.34220	0.61625
2313	C2	C	0.75000	0.09220	0.61625
2314	C2	C	0.75000	0.34220	0.86625
2315	C2	C	0.50000	0.40780	0.86625
2316	C2	C	0.50000	0.15780	0.61625
2317	C2	C	0.75000	0.40780	0.61625
2318	C2	C	0.75000	0.15780	0.86625
2319	C2	C	0.50000	0.40780	0.63375
2320	C2	C	0.50000	0.15780	0.88375
2321	C2	C	0.75000	0.40780	0.88375
2322	C2	C	0.75000	0.15780	0.63375

2323	C2	C	0.50000	0.13375	0.90780
2324	C2	C	0.50000	0.38375	0.65780
2325	C2	C	0.75000	0.13375	0.65780
2326	C2	C	0.75000	0.38375	0.90780
2327	C2	C	0.50000	0.36625	0.90780
2328	C2	C	0.50000	0.11625	0.65780
2329	C2	C	0.75000	0.36625	0.65780
2330	C2	C	0.75000	0.11625	0.90780
2331	C2	C	0.50000	0.36625	0.59220
2332	C2	C	0.50000	0.11625	0.84220
2333	C2	C	0.75000	0.36625	0.84220
2334	C2	C	0.75000	0.11625	0.59220
2335	C2	C	0.50000	0.13375	0.59220
2336	C2	C	0.50000	0.38375	0.84220
2337	C2	C	0.75000	0.13375	0.84220
2338	C2	C	0.75000	0.38375	0.59220
2339	C2	C	0.63375	0.09220	0.50000
2340	C2	C	0.63375	0.34220	0.75000
2341	C2	C	0.88375	0.09220	0.75000
2342	C2	C	0.88375	0.34220	0.50000
2343	C2	C	0.86625	0.09220	0.50000
2344	C2	C	0.86625	0.34220	0.75000
2345	C2	C	0.61625	0.09220	0.75000
2346	C2	C	0.61625	0.34220	0.50000
2347	C2	C	0.86625	0.40780	0.50000
2348	C2	C	0.86625	0.15780	0.75000
2349	C2	C	0.61625	0.40780	0.75000
2350	C2	C	0.61625	0.15780	0.50000
2351	C2	C	0.63375	0.40780	0.50000
2352	C2	C	0.63375	0.15780	0.75000
2353	C2	C	0.88375	0.40780	0.75000
2354	C2	C	0.88375	0.15780	0.50000
2355	C2	C	0.59220	0.00000	0.86625
2356	C2	C	0.59220	0.25000	0.61625
2357	C2	C	0.84220	0.00000	0.61625
2358	C2	C	0.84220	0.25000	0.86625
2359	C2	C	0.59220	0.00000	0.63375
2360	C2	C	0.59220	0.25000	0.88375
2361	C2	C	0.84220	0.00000	0.88375
2362	C2	C	0.84220	0.25000	0.63375
2363	C2	C	0.90780	0.00000	0.63375
2364	C2	C	0.90780	0.25000	0.88375
2365	C2	C	0.65780	0.00000	0.88375
2366	C2	C	0.65780	0.25000	0.63375
2367	C2	C	0.90780	0.00000	0.86625
2368	C2	C	0.90780	0.25000	0.61625
2369	C2	C	0.65780	0.00000	0.61625
2370	C2	C	0.65780	0.25000	0.86625
2371	C1	C	0.57680	0.00000	0.92320
2372	C1	C	0.57680	0.25000	0.67320
2373	C1	C	0.82680	0.00000	0.67320
2374	C1	C	0.82680	0.25000	0.92320
2375	C1	C	0.92320	0.00000	0.92320
2376	C1	C	0.92320	0.25000	0.67320

2377	C1	C	0.67320	0.00000	0.67320
2378	C1	C	0.67320	0.25000	0.92320
2379	C1	C	0.92320	0.00000	0.57680
2380	C1	C	0.92320	0.25000	0.82680
2381	C1	C	0.67320	0.00000	0.82680
2382	C1	C	0.67320	0.25000	0.57680
2383	C1	C	0.57680	0.00000	0.57680
2384	C1	C	0.57680	0.25000	0.82680
2385	C1	C	0.82680	0.00000	0.82680
2386	C1	C	0.82680	0.25000	0.57680
2387	C1	C	0.92320	0.07680	0.50000
2388	C1	C	0.92320	0.32680	0.75000
2389	C1	C	0.67320	0.07680	0.75000
2390	C1	C	0.67320	0.32680	0.50000
2391	C1	C	0.92320	0.42320	0.50000
2392	C1	C	0.92320	0.17320	0.75000
2393	C1	C	0.67320	0.42320	0.75000
2394	C1	C	0.67320	0.17320	0.50000
2395	C1	C	0.57680	0.42320	0.50000
2396	C1	C	0.57680	0.17320	0.75000
2397	C1	C	0.82680	0.42320	0.75000
2398	C1	C	0.82680	0.17320	0.50000
2399	C1	C	0.57680	0.07680	0.50000
2400	C1	C	0.57680	0.32680	0.75000
2401	C1	C	0.82680	0.07680	0.75000
2402	C1	C	0.82680	0.32680	0.50000
2403	C1	C	0.50000	0.42320	0.57680
2404	C1	C	0.50000	0.17320	0.82680
2405	C1	C	0.75000	0.42320	0.82680
2406	C1	C	0.75000	0.17320	0.57680
2407	C1	C	0.50000	0.42320	0.92320
2408	C1	C	0.50000	0.17320	0.67320
2409	C1	C	0.75000	0.42320	0.67320
2410	C1	C	0.75000	0.17320	0.92320
2411	C1	C	0.50000	0.07680	0.92320
2412	C1	C	0.50000	0.32680	0.67320
2413	C1	C	0.75000	0.07680	0.67320
2414	C1	C	0.75000	0.32680	0.92320
2415	C1	C	0.50000	0.07680	0.57680
2416	C1	C	0.50000	0.32680	0.82680
2417	C1	C	0.75000	0.07680	0.82680
2418	C1	C	0.75000	0.32680	0.57680
2419	C3	C	0.60250	0.00000	0.60250
2420	C3	C	0.60250	0.25000	0.85250
2421	C3	C	0.85250	0.00000	0.85250
2422	C3	C	0.85250	0.25000	0.60250
2423	C3	C	0.89750	0.00000	0.60250
2424	C3	C	0.89750	0.25000	0.85250
2425	C3	C	0.64750	0.00000	0.85250
2426	C3	C	0.64750	0.25000	0.60250
2427	C3	C	0.89750	0.00000	0.89750
2428	C3	C	0.89750	0.25000	0.64750
2429	C3	C	0.64750	0.00000	0.64750
2430	C3	C	0.64750	0.25000	0.89750

2431	C3	C	0.60250	0.00000	0.89750
2432	C3	C	0.60250	0.25000	0.64750
2433	C3	C	0.85250	0.00000	0.64750
2434	C3	C	0.85250	0.25000	0.89750
2435	C3	C	0.60250	0.10250	0.50000
2436	C3	C	0.60250	0.35250	0.75000
2437	C3	C	0.85250	0.10250	0.75000
2438	C3	C	0.85250	0.35250	0.50000
2439	C3	C	0.60250	0.39750	0.50000
2440	C3	C	0.60250	0.14750	0.75000
2441	C3	C	0.85250	0.39750	0.75000
2442	C3	C	0.85250	0.14750	0.50000
2443	C3	C	0.89750	0.39750	0.50000
2444	C3	C	0.89750	0.14750	0.75000
2445	C3	C	0.64750	0.39750	0.75000
2446	C3	C	0.64750	0.14750	0.50000
2447	C3	C	0.89750	0.10250	0.50000
2448	C3	C	0.89750	0.35250	0.75000
2449	C3	C	0.64750	0.10250	0.75000
2450	C3	C	0.64750	0.35250	0.50000
2451	C3	C	0.50000	0.10250	0.60250
2452	C3	C	0.50000	0.35250	0.85250
2453	C3	C	0.75000	0.10250	0.85250
2454	C3	C	0.75000	0.35250	0.60250
2455	C3	C	0.50000	0.10250	0.89750
2456	C3	C	0.50000	0.35250	0.64750
2457	C3	C	0.75000	0.10250	0.64750
2458	C3	C	0.75000	0.35250	0.89750
2459	C3	C	0.50000	0.39750	0.89750
2460	C3	C	0.50000	0.14750	0.64750
2461	C3	C	0.75000	0.39750	0.64750
2462	C3	C	0.75000	0.14750	0.89750
2463	C3	C	0.50000	0.39750	0.60250
2464	C3	C	0.50000	0.14750	0.85250
2465	C3	C	0.75000	0.39750	0.85250
2466	C3	C	0.75000	0.14750	0.60250
2467	H1	H	0.63926	0.49997	0.56655
2468	H1	H	0.63926	0.24997	0.81655
2469	H1	H	0.88926	0.49997	0.81655
2470	H1	H	0.88926	0.24997	0.56655
2471	H1	H	0.86074	0.00003	0.56655
2472	H1	H	0.86074	0.25004	0.81655
2473	H1	H	0.61074	0.00003	0.81655
2474	H1	H	0.61074	0.25004	0.56655
2475	H1	H	0.86074	0.49996	0.93345
2476	H1	H	0.86074	0.24997	0.68345
2477	H1	H	0.61074	0.49997	0.68345
2478	H1	H	0.61074	0.24997	0.93346
2479	H1	H	0.63926	0.00003	0.93345
2480	H1	H	0.63926	0.25003	0.68346
2481	H1	H	0.88926	0.00003	0.68345
2482	H1	H	0.88926	0.25004	0.93346
2483	H1	H	0.56655	0.13926	0.99996
2484	H1	H	0.56655	0.38926	0.74997

2485	H1	H	0.81655	0.13926	0.74996
2486	H1	H	0.81654	0.38926	0.99997
2487	H1	H	0.56655	0.36074	0.50003
2488	H1	H	0.56655	0.11074	0.75004
2489	H1	H	0.81655	0.36074	0.75003
2490	H1	H	0.81655	0.11074	0.50004
2491	H1	H	0.93345	0.36074	0.99996
2492	H1	H	0.93345	0.11074	0.74997
2493	H1	H	0.68345	0.36074	0.74996
2494	H1	H	0.68345	0.11074	0.99997
2495	H1	H	0.93345	0.13926	0.50003
2496	H1	H	0.93345	0.38926	0.75004
2497	H1	H	0.68345	0.13926	0.75003
2498	H1	H	0.68345	0.38926	0.50004
2499	H1	H	0.99997	0.06655	0.63926
2500	H1	H	0.99996	0.31655	0.88926
2501	H1	H	0.74997	0.06655	0.88926
2502	H1	H	0.74996	0.31655	0.63926
2503	H1	H	0.50003	0.06655	0.86074
2504	H1	H	0.50003	0.31655	0.61074
2505	H1	H	0.75003	0.06655	0.61074
2506	H1	H	0.75004	0.31655	0.86074
2507	H1	H	0.99997	0.43345	0.86074
2508	H1	H	0.99997	0.18345	0.61074
2509	H1	H	0.74997	0.43345	0.61074
2510	H1	H	0.74997	0.18345	0.86074
2511	H1	H	0.50003	0.43345	0.63926
2512	H1	H	0.50003	0.18345	0.88926
2513	H1	H	0.75004	0.43345	0.88926
2514	H1	H	0.75003	0.18346	0.63926
2515	H1	H	0.50003	0.13926	0.93345
2516	H1	H	0.50003	0.38926	0.68345
2517	H1	H	0.75003	0.13926	0.68345
2518	H1	H	0.75004	0.38926	0.93345
2519	H1	H	0.99996	0.36074	0.93345
2520	H1	H	0.99997	0.11074	0.68345
2521	H1	H	0.74997	0.36074	0.68345
2522	H1	H	0.74997	0.11074	0.93345
2523	H1	H	0.50003	0.36074	0.56655
2524	H1	H	0.50003	0.11074	0.81655
2525	H1	H	0.75004	0.36074	0.81655
2526	H1	H	0.75003	0.11074	0.56654
2527	H1	H	0.99997	0.13926	0.56655
2528	H1	H	0.99997	0.38926	0.81655
2529	H1	H	0.74997	0.13926	0.81655
2530	H1	H	0.74997	0.38926	0.56654
2531	H1	H	0.63926	0.06655	0.99997
2532	H1	H	0.63926	0.31655	0.74996
2533	H1	H	0.88926	0.06655	0.74997
2534	H1	H	0.88926	0.31655	0.99996
2535	H1	H	0.86074	0.06655	0.50003
2536	H1	H	0.86074	0.31655	0.75003
2537	H1	H	0.61074	0.06655	0.75003
2538	H1	H	0.61074	0.31655	0.50003

2539	H1	H	0.86074	0.43345	0.99997
2540	H1	H	0.86074	0.18345	0.74997
2541	H1	H	0.61074	0.43345	0.74997
2542	H1	H	0.61074	0.18345	0.99996
2543	H1	H	0.63926	0.43345	0.50004
2544	H1	H	0.63926	0.18346	0.75003
2545	H1	H	0.88926	0.43345	0.75004
2546	H1	H	0.88926	0.18346	0.50003
2547	H1	H	0.56655	0.00003	0.86074
2548	H1	H	0.56655	0.25004	0.61074
2549	H1	H	0.81655	0.00003	0.61074
2550	H1	H	0.81655	0.25004	0.86074
2551	H1	H	0.56655	0.49996	0.63926
2552	H1	H	0.56655	0.24997	0.88926
2553	H1	H	0.81655	0.49996	0.88926
2554	H1	H	0.81655	0.24997	0.63926
2555	H1	H	0.93345	0.00003	0.63926
2556	H1	H	0.93345	0.25004	0.88926
2557	H1	H	0.68345	0.00003	0.88926
2558	H1	H	0.68345	0.25003	0.63926
2559	H1	H	0.93345	0.49996	0.86074
2560	H1	H	0.93345	0.24997	0.61074
2561	H1	H	0.68345	0.49996	0.61074
2562	H1	H	0.68345	0.24997	0.86074
2563	H2	H	0.70825	0.29176	0.95824
2564	H2	H	0.79174	0.20825	0.95823
2565	H2	H	0.70827	0.20823	0.54177
2566	H2	H	0.79179	0.29168	0.54178
2567	H2	H	0.95823	0.29173	0.70823
2568	H2	H	0.95824	0.20825	0.79176
2569	H2	H	0.54174	0.29176	0.79177
2570	H2	H	0.54173	0.20823	0.70823
2571	H2	H	0.79175	0.45824	0.70824
2572	H2	H	0.70824	0.45824	0.79176
2573	H2	H	0.70823	0.04172	0.70822
2574	H2	H	0.79177	0.04173	0.79178
2575	H2	H	0.54178	0.04177	0.54173
2576	H2	H	0.95823	0.45824	0.54174
2577	H2	H	0.95823	0.04176	0.95825
2578	H2	H	0.54176	0.45824	0.95824
2579	O1	O	0.58524	0.00000	0.54765
2580	O1	O	0.58524	0.25000	0.79765
2581	O1	O	0.83524	0.00000	0.79765
2582	O1	O	0.83524	0.25000	0.54765
2583	O1	O	0.91476	0.00000	0.54765
2584	O1	O	0.91476	0.25000	0.79765
2585	O1	O	0.66476	0.00000	0.79765
2586	O1	O	0.66476	0.25000	0.54765
2587	O1	O	0.91476	0.00000	0.95235
2588	O1	O	0.91476	0.25000	0.70235
2589	O1	O	0.66476	0.00000	0.70235
2590	O1	O	0.66476	0.25000	0.95235
2591	O1	O	0.58524	0.00000	0.95235
2592	O1	O	0.58524	0.25000	0.70235

2593	O1	O	0.83524	0.00000	0.70235
2594	O1	O	0.83524	0.25000	0.95235
2595	O1	O	0.54765	0.08524	0.50000
2596	O1	O	0.54765	0.33524	0.75000
2597	O1	O	0.79765	0.08524	0.75000
2598	O1	O	0.79765	0.33524	0.50000
2599	O1	O	0.54765	0.41476	0.50000
2600	O1	O	0.54765	0.16476	0.75000
2601	O1	O	0.79765	0.41476	0.75000
2602	O1	O	0.79765	0.16476	0.50000
2603	O1	O	0.95235	0.41476	0.50000
2604	O1	O	0.95235	0.16476	0.75000
2605	O1	O	0.70235	0.41476	0.75000
2606	O1	O	0.70235	0.16476	0.50000
2607	O1	O	0.95235	0.08524	0.50000
2608	O1	O	0.95235	0.33524	0.75000
2609	O1	O	0.70235	0.08524	0.75000
2610	O1	O	0.70235	0.33524	0.50000
2611	O1	O	0.50000	0.04765	0.58524
2612	O1	O	0.50000	0.29765	0.83524
2613	O1	O	0.75000	0.04765	0.83524
2614	O1	O	0.75000	0.29765	0.58524
2615	O1	O	0.50000	0.04765	0.91476
2616	O1	O	0.50000	0.29765	0.66476
2617	O1	O	0.75000	0.04765	0.66476
2618	O1	O	0.75000	0.29765	0.91476
2619	O1	O	0.50000	0.45235	0.91476
2620	O1	O	0.50000	0.20235	0.66476
2621	O1	O	0.75000	0.45235	0.66476
2622	O1	O	0.75000	0.20235	0.91476
2623	O1	O	0.50000	0.45235	0.58524
2624	O1	O	0.50000	0.20235	0.83524
2625	O1	O	0.75000	0.45235	0.83524
2626	O1	O	0.75000	0.20235	0.58524
2627	O1	O	0.50000	0.08524	0.95235
2628	O1	O	0.50000	0.33524	0.70235
2629	O1	O	0.75000	0.08524	0.70235
2630	O1	O	0.75000	0.33524	0.95235
2631	O1	O	0.50000	0.41476	0.95235
2632	O1	O	0.50000	0.16476	0.70235
2633	O1	O	0.75000	0.41476	0.70235
2634	O1	O	0.75000	0.16476	0.95235
2635	O1	O	0.50000	0.41476	0.54765
2636	O1	O	0.50000	0.16476	0.79765
2637	O1	O	0.75000	0.41476	0.79765
2638	O1	O	0.75000	0.16476	0.54765
2639	O1	O	0.50000	0.08524	0.54765
2640	O1	O	0.50000	0.33524	0.79765
2641	O1	O	0.75000	0.08524	0.79765
2642	O1	O	0.75000	0.33524	0.54765
2643	O1	O	0.58524	0.04765	0.50000
2644	O1	O	0.58524	0.29765	0.75000
2645	O1	O	0.83524	0.04765	0.75000
2646	O1	O	0.83524	0.29765	0.50000

2647	O1	O	0.91476	0.04765	0.50000
2648	O1	O	0.91476	0.29765	0.75000
2649	O1	O	0.66476	0.04765	0.75000
2650	O1	O	0.66476	0.29765	0.50000
2651	O1	O	0.91476	0.45235	0.50000
2652	O1	O	0.91476	0.20235	0.75000
2653	O1	O	0.66476	0.45235	0.75000
2654	O1	O	0.66476	0.20235	0.50000
2655	O1	O	0.58524	0.45235	0.50000
2656	O1	O	0.58524	0.20235	0.75000
2657	O1	O	0.83524	0.45235	0.75000
2658	O1	O	0.83524	0.20235	0.50000
2659	O1	O	0.54765	0.00000	0.91476
2660	O1	O	0.54765	0.25000	0.66476
2661	O1	O	0.79765	0.00000	0.66476
2662	O1	O	0.79765	0.25000	0.91476
2663	O1	O	0.54765	0.00000	0.58524
2664	O1	O	0.54765	0.25000	0.83524
2665	O1	O	0.79765	0.00000	0.83524
2666	O1	O	0.79765	0.25000	0.58524
2667	O1	O	0.95235	0.00000	0.58524
2668	O1	O	0.95235	0.25000	0.83524
2669	O1	O	0.70235	0.00000	0.83524
2670	O1	O	0.70235	0.25000	0.58524
2671	O1	O	0.95235	0.00000	0.91476
2672	O1	O	0.95235	0.25000	0.66476
2673	O1	O	0.70235	0.00000	0.66476
2674	O1	O	0.70235	0.25000	0.91476
2675	O2	O	0.52805	0.47195	0.97195
2676	O2	O	0.52805	0.22195	0.72195
2677	O2	O	0.77805	0.47195	0.72195
2678	O2	O	0.77805	0.22195	0.97195
2679	O2	O	0.97195	0.02805	0.97195
2680	O2	O	0.97195	0.27805	0.72195
2681	O2	O	0.72195	0.02805	0.72195
2682	O2	O	0.72195	0.27805	0.97195
2683	O2	O	0.97195	0.47195	0.52805
2684	O2	O	0.97195	0.22195	0.77805
2685	O2	O	0.72195	0.47195	0.77805
2686	O2	O	0.72195	0.22195	0.52805
2687	O2	O	0.52805	0.02805	0.52805
2688	O2	O	0.52805	0.27805	0.77805
2689	O2	O	0.77805	0.02805	0.77805
2690	O2	O	0.77805	0.27805	0.52805
2691	O1	O	0.97195	0.02805	0.52805
2692	O1	O	0.97195	0.27805	0.77805
2693	O1	O	0.72195	0.02805	0.77805
2694	O1	O	0.72195	0.27805	0.52805
2695	O1	O	0.52805	0.47195	0.52805
2696	O1	O	0.52805	0.22195	0.77805
2697	O1	O	0.77805	0.47195	0.77805
2698	O1	O	0.77805	0.22195	0.52805
2699	O1	O	0.97195	0.47195	0.97195
2700	O1	O	0.97195	0.22195	0.72195

2701	O1	O	0.72195	0.47195	0.72195
2702	O1	O	0.72195	0.22195	0.97195
2703	O1	O	0.52805	0.02805	0.97195
2704	O1	O	0.52805	0.27805	0.72195
2705	O1	O	0.77805	0.02805	0.72195
2706	O1	O	0.77805	0.27805	0.97195
2707	Zr1	Zr	0.55994	0.00000	0.50000
2708	Zr1	Zr	0.55994	0.25000	0.75000
2709	Zr1	Zr	0.80994	0.00000	0.75000
2710	Zr1	Zr	0.80994	0.25000	0.50000
2711	Zr1	Zr	0.94006	0.00000	0.50000
2712	Zr1	Zr	0.94006	0.25000	0.75000
2713	Zr1	Zr	0.69006	0.00000	0.75000
2714	Zr1	Zr	0.69006	0.25000	0.50000
2715	Zr1	Zr	0.50000	0.05994	0.50000
2716	Zr1	Zr	0.50000	0.30994	0.75000
2717	Zr1	Zr	0.75000	0.05994	0.75000
2718	Zr1	Zr	0.75000	0.30994	0.50000
2719	Zr1	Zr	0.50000	0.44006	0.50000
2720	Zr1	Zr	0.50000	0.19006	0.75000
2721	Zr1	Zr	0.75000	0.44006	0.75000
2722	Zr1	Zr	0.75000	0.19006	0.50000
2723	Zr1	Zr	0.50000	0.00000	0.55994
2724	Zr1	Zr	0.50000	0.25000	0.80994
2725	Zr1	Zr	0.75000	0.00000	0.80994
2726	Zr1	Zr	0.75000	0.25000	0.55994
2727	Zr1	Zr	0.50000	0.00000	0.94006
2728	Zr1	Zr	0.50000	0.25000	0.69006
2729	Zr1	Zr	0.75000	0.00000	0.69006
2730	Zr1	Zr	0.75000	0.25000	0.94006
2731	C2	C	0.63375	0.50000	0.09220
2732	C2	C	0.63375	0.75000	0.34220
2733	C2	C	0.88375	0.50000	0.34220
2734	C2	C	0.88375	0.75000	0.09220
2735	C2	C	0.86625	0.50000	0.09220
2736	C2	C	0.86625	0.75000	0.34220
2737	C2	C	0.61625	0.50000	0.34220
2738	C2	C	0.61625	0.75000	0.09220
2739	C2	C	0.86625	0.50000	0.40780
2740	C2	C	0.86625	0.75000	0.15780
2741	C2	C	0.61625	0.50000	0.15780
2742	C2	C	0.61625	0.75000	0.40780
2743	C2	C	0.63375	0.50000	0.40780
2744	C2	C	0.63375	0.75000	0.15780
2745	C2	C	0.88375	0.50000	0.15780
2746	C2	C	0.88375	0.75000	0.40780
2747	C2	C	0.59220	0.63375	0.00000
2748	C2	C	0.59220	0.88375	0.25000
2749	C2	C	0.84220	0.63375	0.25000
2750	C2	C	0.84220	0.88375	0.00000
2751	C2	C	0.59220	0.86625	0.00000
2752	C2	C	0.59220	0.61625	0.25000
2753	C2	C	0.84220	0.86625	0.25000
2754	C2	C	0.84220	0.61625	0.00000

2755	C2	C	0.90780	0.86625	0.00000
2756	C2	C	0.90780	0.61625	0.25000
2757	C2	C	0.65780	0.86625	0.25000
2758	C2	C	0.65780	0.61625	0.00000
2759	C2	C	0.90780	0.63375	0.00000
2760	C2	C	0.90780	0.88375	0.25000
2761	C2	C	0.65780	0.63375	0.25000
2762	C2	C	0.65780	0.88375	0.00000
2763	C2	C	0.50000	0.59220	0.13375
2764	C2	C	0.50000	0.84220	0.38375
2765	C2	C	0.75000	0.59220	0.38375
2766	C2	C	0.75000	0.84220	0.13375
2767	C2	C	0.50000	0.59220	0.36625
2768	C2	C	0.50000	0.84220	0.11625
2769	C2	C	0.75000	0.59220	0.11625
2770	C2	C	0.75000	0.84220	0.36625
2771	C2	C	0.50000	0.90780	0.36625
2772	C2	C	0.50000	0.65780	0.11625
2773	C2	C	0.75000	0.90780	0.11625
2774	C2	C	0.75000	0.65780	0.36625
2775	C2	C	0.50000	0.90780	0.13375
2776	C2	C	0.50000	0.65780	0.38375
2777	C2	C	0.75000	0.90780	0.38375
2778	C2	C	0.75000	0.65780	0.13375
2779	C2	C	0.50000	0.63375	0.40780
2780	C2	C	0.50000	0.88375	0.15780
2781	C2	C	0.75000	0.63375	0.15780
2782	C2	C	0.75000	0.88375	0.40780
2783	C2	C	0.50000	0.86625	0.40780
2784	C2	C	0.50000	0.61625	0.15780
2785	C2	C	0.75000	0.86625	0.15780
2786	C2	C	0.75000	0.61625	0.40780
2787	C2	C	0.50000	0.86625	0.09220
2788	C2	C	0.50000	0.61625	0.34220
2789	C2	C	0.75000	0.86625	0.34220
2790	C2	C	0.75000	0.61625	0.09220
2791	C2	C	0.50000	0.63375	0.09220
2792	C2	C	0.50000	0.88375	0.34220
2793	C2	C	0.75000	0.63375	0.34220
2794	C2	C	0.75000	0.88375	0.09220
2795	C2	C	0.63375	0.59220	0.00000
2796	C2	C	0.63375	0.84220	0.25000
2797	C2	C	0.88375	0.59220	0.25000
2798	C2	C	0.88375	0.84220	0.00000
2799	C2	C	0.86625	0.59220	0.00000
2800	C2	C	0.86625	0.84220	0.25000
2801	C2	C	0.61625	0.59220	0.25000
2802	C2	C	0.61625	0.84220	0.00000
2803	C2	C	0.86625	0.90780	0.00000
2804	C2	C	0.86625	0.65780	0.25000
2805	C2	C	0.61625	0.90780	0.25000
2806	C2	C	0.61625	0.65780	0.00000
2807	C2	C	0.63375	0.90780	0.00000
2808	C2	C	0.63375	0.65780	0.25000

2809	C2	C	0.88375	0.90780	0.25000
2810	C2	C	0.88375	0.65780	0.00000
2811	C2	C	0.59220	0.50000	0.36625
2812	C2	C	0.59220	0.75000	0.11625
2813	C2	C	0.84220	0.50000	0.11625
2814	C2	C	0.84220	0.75000	0.36625
2815	C2	C	0.59220	0.50000	0.13375
2816	C2	C	0.59220	0.75000	0.38375
2817	C2	C	0.84220	0.50000	0.38375
2818	C2	C	0.84220	0.75000	0.13375
2819	C2	C	0.90780	0.50000	0.13375
2820	C2	C	0.90780	0.75000	0.38375
2821	C2	C	0.65780	0.50000	0.38375
2822	C2	C	0.65780	0.75000	0.13375
2823	C2	C	0.90780	0.50000	0.36625
2824	C2	C	0.90780	0.75000	0.11625
2825	C2	C	0.65780	0.50000	0.11625
2826	C2	C	0.65780	0.75000	0.36625
2827	C1	C	0.57680	0.50000	0.42320
2828	C1	C	0.57680	0.75000	0.17320
2829	C1	C	0.82680	0.50000	0.17320
2830	C1	C	0.82680	0.75000	0.42320
2831	C1	C	0.92320	0.50000	0.42320
2832	C1	C	0.92320	0.75000	0.17320
2833	C1	C	0.67320	0.50000	0.17320
2834	C1	C	0.67320	0.75000	0.42320
2835	C1	C	0.92320	0.50000	0.07680
2836	C1	C	0.92320	0.75000	0.32680
2837	C1	C	0.67320	0.50000	0.32680
2838	C1	C	0.67320	0.75000	0.07680
2839	C1	C	0.57680	0.50000	0.07680
2840	C1	C	0.57680	0.75000	0.32680
2841	C1	C	0.82680	0.50000	0.32680
2842	C1	C	0.82680	0.75000	0.07680
2843	C1	C	0.92320	0.57680	0.00000
2844	C1	C	0.92320	0.82680	0.25000
2845	C1	C	0.67320	0.57680	0.25000
2846	C1	C	0.67320	0.82680	0.00000
2847	C1	C	0.92320	0.92320	0.00000
2848	C1	C	0.92320	0.67320	0.25000
2849	C1	C	0.67320	0.92320	0.25000
2850	C1	C	0.67320	0.67320	0.00000
2851	C1	C	0.57680	0.92320	0.00000
2852	C1	C	0.57680	0.67320	0.25000
2853	C1	C	0.82680	0.92320	0.25000
2854	C1	C	0.82680	0.67320	0.00000
2855	C1	C	0.57680	0.57680	0.00000
2856	C1	C	0.57680	0.82680	0.25000
2857	C1	C	0.82680	0.57680	0.25000
2858	C1	C	0.82680	0.82680	0.00000
2859	C1	C	0.50000	0.92320	0.07680
2860	C1	C	0.50000	0.67320	0.32680
2861	C1	C	0.75000	0.92320	0.32680
2862	C1	C	0.75000	0.67320	0.07680

2863	C1	C	0.50000	0.92320	0.42320
2864	C1	C	0.50000	0.67320	0.17320
2865	C1	C	0.75000	0.92320	0.17320
2866	C1	C	0.75000	0.67320	0.42320
2867	C1	C	0.50000	0.57680	0.42320
2868	C1	C	0.50000	0.82680	0.17320
2869	C1	C	0.75000	0.57680	0.17320
2870	C1	C	0.75000	0.82680	0.42320
2871	C1	C	0.50000	0.57680	0.07680
2872	C1	C	0.50000	0.82680	0.32680
2873	C1	C	0.75000	0.57680	0.32680
2874	C1	C	0.75000	0.82680	0.07680
2875	C3	C	0.60250	0.50000	0.10250
2876	C3	C	0.60250	0.75000	0.35250
2877	C3	C	0.85250	0.50000	0.35250
2878	C3	C	0.85250	0.75000	0.10250
2879	C3	C	0.89750	0.50000	0.10250
2880	C3	C	0.89750	0.75000	0.35250
2881	C3	C	0.64750	0.50000	0.35250
2882	C3	C	0.64750	0.75000	0.10250
2883	C3	C	0.89750	0.50000	0.39750
2884	C3	C	0.89750	0.75000	0.14750
2885	C3	C	0.64750	0.50000	0.14750
2886	C3	C	0.64750	0.75000	0.39750
2887	C3	C	0.60250	0.50000	0.39750
2888	C3	C	0.60250	0.75000	0.14750
2889	C3	C	0.85250	0.50000	0.14750
2890	C3	C	0.85250	0.75000	0.39750
2891	C3	C	0.60250	0.60250	0.00000
2892	C3	C	0.60250	0.85250	0.25000
2893	C3	C	0.85250	0.60250	0.25000
2894	C3	C	0.85250	0.85250	0.00000
2895	C3	C	0.60250	0.89750	0.00000
2896	C3	C	0.60250	0.64750	0.25000
2897	C3	C	0.85250	0.89750	0.25000
2898	C3	C	0.85250	0.64750	0.00000
2899	C3	C	0.89750	0.89750	0.00000
2900	C3	C	0.89750	0.64750	0.25000
2901	C3	C	0.64750	0.89750	0.25000
2902	C3	C	0.64750	0.64750	0.00000
2903	C3	C	0.89750	0.60250	0.00000
2904	C3	C	0.89750	0.85250	0.25000
2905	C3	C	0.64750	0.60250	0.25000
2906	C3	C	0.64750	0.85250	0.00000
2907	C3	C	0.50000	0.60250	0.10250
2908	C3	C	0.50000	0.85250	0.35250
2909	C3	C	0.75000	0.60250	0.35250
2910	C3	C	0.75000	0.85250	0.10250
2911	C3	C	0.50000	0.60250	0.39750
2912	C3	C	0.50000	0.85250	0.14750
2913	C3	C	0.75000	0.60250	0.14750
2914	C3	C	0.75000	0.85250	0.39750
2915	C3	C	0.50000	0.89750	0.39750
2916	C3	C	0.50000	0.64750	0.14750

2917	C3	C	0.75000	0.89750	0.14750
2918	C3	C	0.75000	0.64750	0.39750
2919	C3	C	0.50000	0.89750	0.10250
2920	C3	C	0.50000	0.64750	0.35250
2921	C3	C	0.75000	0.89750	0.35250
2922	C3	C	0.75000	0.64750	0.10250
2923	H1	H	0.63926	0.99997	0.06655
2924	H1	H	0.63926	0.74997	0.31655
2925	H1	H	0.88926	0.99997	0.31655
2926	H1	H	0.88926	0.74997	0.06655
2927	H1	H	0.86074	0.50003	0.06655
2928	H1	H	0.86074	0.75004	0.31655
2929	H1	H	0.61074	0.50003	0.31655
2930	H1	H	0.61074	0.75004	0.06655
2931	H1	H	0.86074	0.99996	0.43345
2932	H1	H	0.86074	0.74997	0.18345
2933	H1	H	0.61074	0.99997	0.18345
2934	H1	H	0.61074	0.74997	0.43346
2935	H1	H	0.63926	0.50003	0.43345
2936	H1	H	0.63926	0.75003	0.18346
2937	H1	H	0.88926	0.50003	0.18345
2938	H1	H	0.88926	0.75004	0.43346
2939	H1	H	0.56655	0.63926	0.49996
2940	H1	H	0.56655	0.88926	0.24997
2941	H1	H	0.81655	0.63926	0.24996
2942	H1	H	0.81654	0.88926	0.49997
2943	H1	H	0.56655	0.86074	0.00003
2944	H1	H	0.56655	0.61074	0.25004
2945	H1	H	0.81655	0.86074	0.25003
2946	H1	H	0.81655	0.61074	0.00004
2947	H1	H	0.93345	0.86074	0.49996
2948	H1	H	0.93345	0.61074	0.24997
2949	H1	H	0.68345	0.86074	0.24996
2950	H1	H	0.68345	0.61074	0.49997
2951	H1	H	0.93345	0.63926	0.00003
2952	H1	H	0.93345	0.88926	0.25004
2953	H1	H	0.68345	0.63926	0.25003
2954	H1	H	0.68345	0.88926	0.00004
2955	H1	H	0.99997	0.56655	0.13926
2956	H1	H	0.99996	0.81655	0.38926
2957	H1	H	0.74997	0.56655	0.38926
2958	H1	H	0.74996	0.81655	0.13926
2959	H1	H	0.50003	0.56655	0.36074
2960	H1	H	0.50003	0.81655	0.11074
2961	H1	H	0.75003	0.56655	0.11074
2962	H1	H	0.75004	0.81655	0.36074
2963	H1	H	0.99997	0.93345	0.36074
2964	H1	H	0.99997	0.68345	0.11074
2965	H1	H	0.74997	0.93345	0.11074
2966	H1	H	0.74997	0.68345	0.36074
2967	H1	H	0.50003	0.93345	0.13926
2968	H1	H	0.50003	0.68345	0.38926
2969	H1	H	0.75004	0.93345	0.38926
2970	H1	H	0.75003	0.68346	0.13926

2971	H1	H	0.50003	0.63926	0.43345
2972	H1	H	0.50003	0.88926	0.18345
2973	H1	H	0.75003	0.63926	0.18345
2974	H1	H	0.75004	0.88926	0.43345
2975	H1	H	0.99996	0.86074	0.43345
2976	H1	H	0.99997	0.61074	0.18345
2977	H1	H	0.74997	0.86074	0.18345
2978	H1	H	0.74997	0.61074	0.43345
2979	H1	H	0.50003	0.86074	0.06655
2980	H1	H	0.50003	0.61074	0.31655
2981	H1	H	0.75004	0.86074	0.31655
2982	H1	H	0.75003	0.61074	0.06654
2983	H1	H	0.99997	0.63926	0.06655
2984	H1	H	0.99997	0.88926	0.31655
2985	H1	H	0.74997	0.63926	0.31655
2986	H1	H	0.74997	0.88926	0.06654
2987	H1	H	0.63926	0.56655	0.49997
2988	H1	H	0.63926	0.81655	0.24996
2989	H1	H	0.88926	0.56655	0.24997
2990	H1	H	0.88926	0.81655	0.49996
2991	H1	H	0.86074	0.56655	0.00003
2992	H1	H	0.86074	0.81655	0.25003
2993	H1	H	0.61074	0.56655	0.25003
2994	H1	H	0.61074	0.81655	0.00003
2995	H1	H	0.86074	0.93345	0.49997
2996	H1	H	0.86074	0.68345	0.24997
2997	H1	H	0.61074	0.93345	0.24997
2998	H1	H	0.61074	0.68345	0.49996
2999	H1	H	0.63926	0.93345	0.00004
3000	H1	H	0.63926	0.68346	0.25003
3001	H1	H	0.88926	0.93345	0.25004
3002	H1	H	0.88926	0.68346	0.00003
3003	H1	H	0.56655	0.50003	0.36074
3004	H1	H	0.56655	0.75004	0.11074
3005	H1	H	0.81655	0.50003	0.11074
3006	H1	H	0.81655	0.75004	0.36074
3007	H1	H	0.56655	0.99996	0.13926
3008	H1	H	0.56655	0.74997	0.38926
3009	H1	H	0.81655	0.99996	0.38926
3010	H1	H	0.81655	0.74997	0.13926
3011	H1	H	0.93345	0.50003	0.13926
3012	H1	H	0.93345	0.75004	0.38926
3013	H1	H	0.68345	0.50003	0.38926
3014	H1	H	0.68345	0.75003	0.13926
3015	H1	H	0.93345	0.99996	0.36074
3016	H1	H	0.93345	0.74997	0.11074
3017	H1	H	0.68345	0.99996	0.11074
3018	H1	H	0.68345	0.74997	0.36074
3019	H2	H	0.70825	0.79176	0.45824
3020	H2	H	0.79174	0.70825	0.45823
3021	H2	H	0.70827	0.70823	0.04177
3022	H2	H	0.79179	0.79168	0.04178
3023	H2	H	0.95823	0.79173	0.20823
3024	H2	H	0.95824	0.70825	0.29176

3025	H2	H	0.54174	0.79176	0.29177
3026	H2	H	0.54173	0.70823	0.20823
3027	H2	H	0.79175	0.95824	0.20824
3028	H2	H	0.70824	0.95824	0.29176
3029	H2	H	0.70823	0.54172	0.20822
3030	H2	H	0.79177	0.54173	0.29178
3031	H2	H	0.54178	0.54177	0.04173
3032	H2	H	0.95823	0.95824	0.04174
3033	H2	H	0.95823	0.54176	0.45825
3034	H2	H	0.54176	0.95824	0.45824
3035	O1	O	0.58524	0.50000	0.04765
3036	O1	O	0.58524	0.75000	0.29765
3037	O1	O	0.83524	0.50000	0.29765
3038	O1	O	0.83524	0.75000	0.04765
3039	O1	O	0.91476	0.50000	0.04765
3040	O1	O	0.91476	0.75000	0.29765
3041	O1	O	0.66476	0.50000	0.29765
3042	O1	O	0.66476	0.75000	0.04765
3043	O1	O	0.91476	0.50000	0.45235
3044	O1	O	0.91476	0.75000	0.20235
3045	O1	O	0.66476	0.50000	0.20235
3046	O1	O	0.66476	0.75000	0.45235
3047	O1	O	0.58524	0.50000	0.45235
3048	O1	O	0.58524	0.75000	0.20235
3049	O1	O	0.83524	0.50000	0.20235
3050	O1	O	0.83524	0.75000	0.45235
3051	O1	O	0.54765	0.58524	0.00000
3052	O1	O	0.54765	0.83524	0.25000
3053	O1	O	0.79765	0.58524	0.25000
3054	O1	O	0.79765	0.83524	0.00000
3055	O1	O	0.54765	0.91476	0.00000
3056	O1	O	0.54765	0.66476	0.25000
3057	O1	O	0.79765	0.91476	0.25000
3058	O1	O	0.79765	0.66476	0.00000
3059	O1	O	0.95235	0.91476	0.00000
3060	O1	O	0.95235	0.66476	0.25000
3061	O1	O	0.70235	0.91476	0.25000
3062	O1	O	0.70235	0.66476	0.00000
3063	O1	O	0.95235	0.58524	0.00000
3064	O1	O	0.95235	0.83524	0.25000
3065	O1	O	0.70235	0.58524	0.25000
3066	O1	O	0.70235	0.83524	0.00000
3067	O1	O	0.50000	0.54765	0.08524
3068	O1	O	0.50000	0.79765	0.33524
3069	O1	O	0.75000	0.54765	0.33524
3070	O1	O	0.75000	0.79765	0.08524
3071	O1	O	0.50000	0.54765	0.41476
3072	O1	O	0.50000	0.79765	0.16476
3073	O1	O	0.75000	0.54765	0.16476
3074	O1	O	0.75000	0.79765	0.41476
3075	O1	O	0.50000	0.95235	0.41476
3076	O1	O	0.50000	0.70235	0.16476
3077	O1	O	0.75000	0.95235	0.16476
3078	O1	O	0.75000	0.70235	0.41476

3079	O1	O	0.50000	0.95235	0.08524
3080	O1	O	0.50000	0.70235	0.33524
3081	O1	O	0.75000	0.95235	0.33524
3082	O1	O	0.75000	0.70235	0.08524
3083	O1	O	0.50000	0.58524	0.45235
3084	O1	O	0.50000	0.83524	0.20235
3085	O1	O	0.75000	0.58524	0.20235
3086	O1	O	0.75000	0.83524	0.45235
3087	O1	O	0.50000	0.91476	0.45235
3088	O1	O	0.50000	0.66476	0.20235
3089	O1	O	0.75000	0.91476	0.20235
3090	O1	O	0.75000	0.66476	0.45235
3091	O1	O	0.50000	0.91476	0.04765
3092	O1	O	0.50000	0.66476	0.29765
3093	O1	O	0.75000	0.91476	0.29765
3094	O1	O	0.75000	0.66476	0.04765
3095	O1	O	0.50000	0.58524	0.04765
3096	O1	O	0.50000	0.83524	0.29765
3097	O1	O	0.75000	0.58524	0.29765
3098	O1	O	0.75000	0.83524	0.04765
3099	O1	O	0.58524	0.54765	0.00000
3100	O1	O	0.58524	0.79765	0.25000
3101	O1	O	0.83524	0.54765	0.25000
3102	O1	O	0.83524	0.79765	0.00000
3103	O1	O	0.91476	0.54765	0.00000
3104	O1	O	0.91476	0.79765	0.25000
3105	O1	O	0.66476	0.54765	0.25000
3106	O1	O	0.66476	0.79765	0.00000
3107	O1	O	0.91476	0.95235	0.00000
3108	O1	O	0.91476	0.70235	0.25000
3109	O1	O	0.66476	0.95235	0.25000
3110	O1	O	0.66476	0.70235	0.00000
3111	O1	O	0.58524	0.95235	0.00000
3112	O1	O	0.58524	0.70235	0.25000
3113	O1	O	0.83524	0.95235	0.25000
3114	O1	O	0.83524	0.70235	0.00000
3115	O1	O	0.54765	0.50000	0.41476
3116	O1	O	0.54765	0.75000	0.16476
3117	O1	O	0.79765	0.50000	0.16476
3118	O1	O	0.79765	0.75000	0.41476
3119	O1	O	0.54765	0.50000	0.08524
3120	O1	O	0.54765	0.75000	0.33524
3121	O1	O	0.79765	0.50000	0.33524
3122	O1	O	0.79765	0.75000	0.08524
3123	O1	O	0.95235	0.50000	0.08524
3124	O1	O	0.95235	0.75000	0.33524
3125	O1	O	0.70235	0.50000	0.33524
3126	O1	O	0.70235	0.75000	0.08524
3127	O1	O	0.95235	0.50000	0.41476
3128	O1	O	0.95235	0.75000	0.16476
3129	O1	O	0.70235	0.50000	0.16476
3130	O1	O	0.70235	0.75000	0.41476
3131	O2	O	0.52805	0.97195	0.47195
3132	O2	O	0.52805	0.72195	0.22195

3133	O2	O	0.77805	0.97195	0.22195
3134	O2	O	0.77805	0.72195	0.47195
3135	O2	O	0.97195	0.52805	0.47195
3136	O2	O	0.97195	0.77805	0.22195
3137	O2	O	0.72195	0.52805	0.22195
3138	O2	O	0.72195	0.77805	0.47195
3139	O2	O	0.97195	0.97195	0.02805
3140	O2	O	0.97195	0.72195	0.27805
3141	O2	O	0.72195	0.97195	0.27805
3142	O2	O	0.72195	0.72195	0.02805
3143	O2	O	0.52805	0.52805	0.02805
3144	O2	O	0.52805	0.77805	0.27805
3145	O2	O	0.77805	0.52805	0.27805
3146	O2	O	0.77805	0.77805	0.02805
3147	O1	O	0.97195	0.52805	0.02805
3148	O1	O	0.97195	0.77805	0.27805
3149	O1	O	0.72195	0.52805	0.27805
3150	O1	O	0.72195	0.77805	0.02805
3151	O1	O	0.52805	0.97195	0.02805
3152	O1	O	0.52805	0.72195	0.27805
3153	O1	O	0.77805	0.97195	0.27805
3154	O1	O	0.77805	0.72195	0.02805
3155	O1	O	0.97195	0.97195	0.47195
3156	O1	O	0.97195	0.72195	0.22195
3157	O1	O	0.72195	0.97195	0.22195
3158	O1	O	0.72195	0.72195	0.47195
3159	O1	O	0.52805	0.52805	0.47195
3160	O1	O	0.52805	0.77805	0.22195
3161	O1	O	0.77805	0.52805	0.22195
3162	O1	O	0.77805	0.77805	0.47195
3163	Zr1	Zr	0.55994	0.50000	0.00000
3164	Zr1	Zr	0.55994	0.75000	0.25000
3165	Zr1	Zr	0.80994	0.50000	0.25000
3166	Zr1	Zr	0.80994	0.75000	0.00000
3167	Zr1	Zr	0.94006	0.50000	0.00000
3168	Zr1	Zr	0.94006	0.75000	0.25000
3169	Zr1	Zr	0.69006	0.50000	0.25000
3170	Zr1	Zr	0.69006	0.75000	0.00000
3171	Zr1	Zr	0.50000	0.55994	0.00000
3172	Zr1	Zr	0.50000	0.80994	0.25000
3173	Zr1	Zr	0.75000	0.55994	0.25000
3174	Zr1	Zr	0.75000	0.80994	0.00000
3175	Zr1	Zr	0.50000	0.94006	0.00000
3176	Zr1	Zr	0.50000	0.69006	0.25000
3177	Zr1	Zr	0.75000	0.94006	0.25000
3178	Zr1	Zr	0.75000	0.69006	0.00000
3179	Zr1	Zr	0.50000	0.50000	0.05994
3180	Zr1	Zr	0.50000	0.75000	0.30994
3181	Zr1	Zr	0.75000	0.50000	0.30994
3182	Zr1	Zr	0.75000	0.75000	0.05994
3183	Zr1	Zr	0.50000	0.50000	0.44006
3184	Zr1	Zr	0.50000	0.75000	0.19006
3185	Zr1	Zr	0.75000	0.50000	0.19006
3186	Zr1	Zr	0.75000	0.75000	0.44006

3187	C2	C	0.63375	0.50000	0.59220
3188	C2	C	0.63375	0.75000	0.84220
3189	C2	C	0.88375	0.50000	0.84220
3190	C2	C	0.88375	0.75000	0.59220
3191	C2	C	0.86625	0.50000	0.59220
3192	C2	C	0.86625	0.75000	0.84220
3193	C2	C	0.61625	0.50000	0.84220
3194	C2	C	0.61625	0.75000	0.59220
3195	C2	C	0.86625	0.50000	0.90780
3196	C2	C	0.86625	0.75000	0.65780
3197	C2	C	0.61625	0.50000	0.65780
3198	C2	C	0.61625	0.75000	0.90780
3199	C2	C	0.63375	0.50000	0.90780
3200	C2	C	0.63375	0.75000	0.65780
3201	C2	C	0.88375	0.50000	0.65780
3202	C2	C	0.88375	0.75000	0.90780
3203	C2	C	0.59220	0.63375	0.50000
3204	C2	C	0.59220	0.88375	0.75000
3205	C2	C	0.84220	0.63375	0.75000
3206	C2	C	0.84220	0.88375	0.50000
3207	C2	C	0.59220	0.86625	0.50000
3208	C2	C	0.59220	0.61625	0.75000
3209	C2	C	0.84220	0.86625	0.75000
3210	C2	C	0.84220	0.61625	0.50000
3211	C2	C	0.90780	0.86625	0.50000
3212	C2	C	0.90780	0.61625	0.75000
3213	C2	C	0.65780	0.86625	0.75000
3214	C2	C	0.65780	0.61625	0.50000
3215	C2	C	0.90780	0.63375	0.50000
3216	C2	C	0.90780	0.88375	0.75000
3217	C2	C	0.65780	0.63375	0.75000
3218	C2	C	0.65780	0.88375	0.50000
3219	C2	C	0.50000	0.59220	0.63375
3220	C2	C	0.50000	0.84220	0.88375
3221	C2	C	0.75000	0.59220	0.88375
3222	C2	C	0.75000	0.84220	0.63375
3223	C2	C	0.50000	0.59220	0.86625
3224	C2	C	0.50000	0.84220	0.61625
3225	C2	C	0.75000	0.59220	0.61625
3226	C2	C	0.75000	0.84220	0.86625
3227	C2	C	0.50000	0.90780	0.86625
3228	C2	C	0.50000	0.65780	0.61625
3229	C2	C	0.75000	0.90780	0.61625
3230	C2	C	0.75000	0.65780	0.86625
3231	C2	C	0.50000	0.90780	0.63375
3232	C2	C	0.50000	0.65780	0.88375
3233	C2	C	0.75000	0.90780	0.88375
3234	C2	C	0.75000	0.65780	0.63375
3235	C2	C	0.50000	0.63375	0.90780
3236	C2	C	0.50000	0.88375	0.65780
3237	C2	C	0.75000	0.63375	0.65780
3238	C2	C	0.75000	0.88375	0.90780
3239	C2	C	0.50000	0.86625	0.90780
3240	C2	C	0.50000	0.61625	0.65780

3241	C2	C	0.75000	0.86625	0.65780
3242	C2	C	0.75000	0.61625	0.90780
3243	C2	C	0.50000	0.86625	0.59220
3244	C2	C	0.50000	0.61625	0.84220
3245	C2	C	0.75000	0.86625	0.84220
3246	C2	C	0.75000	0.61625	0.59220
3247	C2	C	0.50000	0.63375	0.59220
3248	C2	C	0.50000	0.88375	0.84220
3249	C2	C	0.75000	0.63375	0.84220
3250	C2	C	0.75000	0.88375	0.59220
3251	C2	C	0.63375	0.59220	0.50000
3252	C2	C	0.63375	0.84220	0.75000
3253	C2	C	0.88375	0.59220	0.75000
3254	C2	C	0.88375	0.84220	0.50000
3255	C2	C	0.86625	0.59220	0.50000
3256	C2	C	0.86625	0.84220	0.75000
3257	C2	C	0.61625	0.59220	0.75000
3258	C2	C	0.61625	0.84220	0.50000
3259	C2	C	0.86625	0.90780	0.50000
3260	C2	C	0.86625	0.65780	0.75000
3261	C2	C	0.61625	0.90780	0.75000
3262	C2	C	0.61625	0.65780	0.50000
3263	C2	C	0.63375	0.90780	0.50000
3264	C2	C	0.63375	0.65780	0.75000
3265	C2	C	0.88375	0.90780	0.75000
3266	C2	C	0.88375	0.65780	0.50000
3267	C2	C	0.59220	0.50000	0.86625
3268	C2	C	0.59220	0.75000	0.61625
3269	C2	C	0.84220	0.50000	0.61625
3270	C2	C	0.84220	0.75000	0.86625
3271	C2	C	0.59220	0.50000	0.63375
3272	C2	C	0.59220	0.75000	0.88375
3273	C2	C	0.84220	0.50000	0.88375
3274	C2	C	0.84220	0.75000	0.63375
3275	C2	C	0.90780	0.50000	0.63375
3276	C2	C	0.90780	0.75000	0.88375
3277	C2	C	0.65780	0.50000	0.88375
3278	C2	C	0.65780	0.75000	0.63375
3279	C2	C	0.90780	0.50000	0.86625
3280	C2	C	0.90780	0.75000	0.61625
3281	C2	C	0.65780	0.50000	0.61625
3282	C2	C	0.65780	0.75000	0.86625
3283	C1	C	0.57680	0.50000	0.92320
3284	C1	C	0.57680	0.75000	0.67320
3285	C1	C	0.82680	0.50000	0.67320
3286	C1	C	0.82680	0.75000	0.92320
3287	C1	C	0.92320	0.50000	0.92320
3288	C1	C	0.92320	0.75000	0.67320
3289	C1	C	0.67320	0.50000	0.67320
3290	C1	C	0.67320	0.75000	0.92320
3291	C1	C	0.92320	0.50000	0.57680
3292	C1	C	0.92320	0.75000	0.82680
3293	C1	C	0.67320	0.50000	0.82680
3294	C1	C	0.67320	0.75000	0.57680

3295	C1	C	0.57680	0.50000	0.57680
3296	C1	C	0.57680	0.75000	0.82680
3297	C1	C	0.82680	0.50000	0.82680
3298	C1	C	0.82680	0.75000	0.57680
3299	C1	C	0.92320	0.57680	0.50000
3300	C1	C	0.92320	0.82680	0.75000
3301	C1	C	0.67320	0.57680	0.75000
3302	C1	C	0.67320	0.82680	0.50000
3303	C1	C	0.92320	0.92320	0.50000
3304	C1	C	0.92320	0.67320	0.75000
3305	C1	C	0.67320	0.92320	0.75000
3306	C1	C	0.67320	0.67320	0.50000
3307	C1	C	0.57680	0.92320	0.50000
3308	C1	C	0.57680	0.67320	0.75000
3309	C1	C	0.82680	0.92320	0.75000
3310	C1	C	0.82680	0.67320	0.50000
3311	C1	C	0.57680	0.57680	0.50000
3312	C1	C	0.57680	0.82680	0.75000
3313	C1	C	0.82680	0.57680	0.75000
3314	C1	C	0.82680	0.82680	0.50000
3315	C1	C	0.50000	0.92320	0.57680
3316	C1	C	0.50000	0.67320	0.82680
3317	C1	C	0.75000	0.92320	0.82680
3318	C1	C	0.75000	0.67320	0.57680
3319	C1	C	0.50000	0.92320	0.92320
3320	C1	C	0.50000	0.67320	0.67320
3321	C1	C	0.75000	0.92320	0.67320
3322	C1	C	0.75000	0.67320	0.92320
3323	C1	C	0.50000	0.57680	0.92320
3324	C1	C	0.50000	0.82680	0.67320
3325	C1	C	0.75000	0.57680	0.67320
3326	C1	C	0.75000	0.82680	0.92320
3327	C1	C	0.50000	0.57680	0.57680
3328	C1	C	0.50000	0.82680	0.82680
3329	C1	C	0.75000	0.57680	0.82680
3330	C1	C	0.75000	0.82680	0.57680
3331	C3	C	0.60250	0.50000	0.60250
3332	C3	C	0.60250	0.75000	0.85250
3333	C3	C	0.85250	0.50000	0.85250
3334	C3	C	0.85250	0.75000	0.60250
3335	C3	C	0.89750	0.50000	0.60250
3336	C3	C	0.89750	0.75000	0.85250
3337	C3	C	0.64750	0.50000	0.85250
3338	C3	C	0.64750	0.75000	0.60250
3339	C3	C	0.89750	0.50000	0.89750
3340	C3	C	0.89750	0.75000	0.64750
3341	C3	C	0.64750	0.50000	0.64750
3342	C3	C	0.64750	0.75000	0.89750
3343	C3	C	0.60250	0.50000	0.89750
3344	C3	C	0.60250	0.75000	0.64750
3345	C3	C	0.85250	0.50000	0.64750
3346	C3	C	0.85250	0.75000	0.89750
3347	C3	C	0.60250	0.60250	0.50000
3348	C3	C	0.60250	0.85250	0.75000

3349	C3	C	0.85250	0.60250	0.75000
3350	C3	C	0.85250	0.85250	0.50000
3351	C3	C	0.60250	0.89750	0.50000
3352	C3	C	0.60250	0.64750	0.75000
3353	C3	C	0.85250	0.89750	0.75000
3354	C3	C	0.85250	0.64750	0.50000
3355	C3	C	0.89750	0.89750	0.50000
3356	C3	C	0.89750	0.64750	0.75000
3357	C3	C	0.64750	0.89750	0.75000
3358	C3	C	0.64750	0.64750	0.50000
3359	C3	C	0.89750	0.60250	0.50000
3360	C3	C	0.89750	0.85250	0.75000
3361	C3	C	0.64750	0.60250	0.75000
3362	C3	C	0.64750	0.85250	0.50000
3363	C3	C	0.50000	0.60250	0.60250
3364	C3	C	0.50000	0.85250	0.85250
3365	C3	C	0.75000	0.60250	0.85250
3366	C3	C	0.75000	0.85250	0.60250
3367	C3	C	0.50000	0.60250	0.89750
3368	C3	C	0.50000	0.85250	0.64750
3369	C3	C	0.75000	0.60250	0.64750
3370	C3	C	0.75000	0.85250	0.89750
3371	C3	C	0.50000	0.89750	0.89750
3372	C3	C	0.50000	0.64750	0.64750
3373	C3	C	0.75000	0.89750	0.64750
3374	C3	C	0.75000	0.64750	0.89750
3375	C3	C	0.50000	0.89750	0.60250
3376	C3	C	0.50000	0.64750	0.85250
3377	C3	C	0.75000	0.89750	0.85250
3378	C3	C	0.75000	0.64750	0.60250
3379	H1	H	0.63926	0.99997	0.56655
3380	H1	H	0.63926	0.74997	0.81655
3381	H1	H	0.88926	0.99997	0.81655
3382	H1	H	0.88926	0.74997	0.56655
3383	H1	H	0.86074	0.50003	0.56655
3384	H1	H	0.86074	0.75004	0.81655
3385	H1	H	0.61074	0.50003	0.81655
3386	H1	H	0.61074	0.75004	0.56655
3387	H1	H	0.86074	0.99996	0.93345
3388	H1	H	0.86074	0.74997	0.68345
3389	H1	H	0.61074	0.99997	0.68345
3390	H1	H	0.61074	0.74997	0.93346
3391	H1	H	0.63926	0.50003	0.93345
3392	H1	H	0.63926	0.75003	0.68346
3393	H1	H	0.88926	0.50003	0.68345
3394	H1	H	0.88926	0.75004	0.93346
3395	H1	H	0.56655	0.63926	0.99996
3396	H1	H	0.56655	0.88926	0.74997
3397	H1	H	0.81655	0.63926	0.74996
3398	H1	H	0.81654	0.88926	0.99997
3399	H1	H	0.56655	0.86074	0.50003
3400	H1	H	0.56655	0.61074	0.75004
3401	H1	H	0.81655	0.86074	0.75003
3402	H1	H	0.81655	0.61074	0.50004

3403	H1	H	0.93345	0.86074	0.99996
3404	H1	H	0.93345	0.61074	0.74997
3405	H1	H	0.68345	0.86074	0.74996
3406	H1	H	0.68345	0.61074	0.99997
3407	H1	H	0.93345	0.63926	0.50003
3408	H1	H	0.93345	0.88926	0.75004
3409	H1	H	0.68345	0.63926	0.75003
3410	H1	H	0.68345	0.88926	0.50004
3411	H1	H	0.99997	0.56655	0.63926
3412	H1	H	0.99996	0.81655	0.88926
3413	H1	H	0.74997	0.56655	0.88926
3414	H1	H	0.74996	0.81655	0.63926
3415	H1	H	0.50003	0.56655	0.86074
3416	H1	H	0.50003	0.81655	0.61074
3417	H1	H	0.75003	0.56655	0.61074
3418	H1	H	0.75004	0.81655	0.86074
3419	H1	H	0.99997	0.93345	0.86074
3420	H1	H	0.99997	0.68345	0.61074
3421	H1	H	0.74997	0.93345	0.61074
3422	H1	H	0.74997	0.68345	0.86074
3423	H1	H	0.50003	0.93345	0.63926
3424	H1	H	0.50003	0.68345	0.88926
3425	H1	H	0.75004	0.93345	0.88926
3426	H1	H	0.75003	0.68346	0.63926
3427	H1	H	0.50003	0.63926	0.93345
3428	H1	H	0.50003	0.88926	0.68345
3429	H1	H	0.75003	0.63926	0.68345
3430	H1	H	0.75004	0.88926	0.93345
3431	H1	H	0.99996	0.86074	0.93345
3432	H1	H	0.99997	0.61074	0.68345
3433	H1	H	0.74997	0.86074	0.68345
3434	H1	H	0.74997	0.61074	0.93345
3435	H1	H	0.50003	0.86074	0.56655
3436	H1	H	0.50003	0.61074	0.81655
3437	H1	H	0.75004	0.86074	0.81655
3438	H1	H	0.75003	0.61074	0.56654
3439	H1	H	0.99997	0.63926	0.56655
3440	H1	H	0.99997	0.88926	0.81655
3441	H1	H	0.74997	0.63926	0.81655
3442	H1	H	0.74997	0.88926	0.56654
3443	H1	H	0.63926	0.56655	0.99997
3444	H1	H	0.63926	0.81655	0.74996
3445	H1	H	0.88926	0.56655	0.74997
3446	H1	H	0.88926	0.81655	0.99996
3447	H1	H	0.86074	0.56655	0.50003
3448	H1	H	0.86074	0.81655	0.75003
3449	H1	H	0.61074	0.56655	0.75003
3450	H1	H	0.61074	0.81655	0.50003
3451	H1	H	0.86074	0.93345	0.99997
3452	H1	H	0.86074	0.68345	0.74997
3453	H1	H	0.61074	0.93345	0.74997
3454	H1	H	0.61074	0.68345	0.99996
3455	H1	H	0.63926	0.93345	0.50004
3456	H1	H	0.63926	0.68346	0.75003

3457	H1	H	0.88926	0.93345	0.75004
3458	H1	H	0.88926	0.68346	0.50003
3459	H1	H	0.56655	0.50003	0.86074
3460	H1	H	0.56655	0.75004	0.61074
3461	H1	H	0.81655	0.50003	0.61074
3462	H1	H	0.81655	0.75004	0.86074
3463	H1	H	0.56655	0.99996	0.63926
3464	H1	H	0.56655	0.74997	0.88926
3465	H1	H	0.81655	0.99996	0.88926
3466	H1	H	0.81655	0.74997	0.63926
3467	H1	H	0.93345	0.50003	0.63926
3468	H1	H	0.93345	0.75004	0.88926
3469	H1	H	0.68345	0.50003	0.88926
3470	H1	H	0.68345	0.75003	0.63926
3471	H1	H	0.93345	0.99996	0.86074
3472	H1	H	0.93345	0.74997	0.61074
3473	H1	H	0.68345	0.99996	0.61074
3474	H1	H	0.68345	0.74997	0.86074
3475	H2	H	0.70825	0.79176	0.95824
3476	H2	H	0.79174	0.70825	0.95823
3477	H2	H	0.70827	0.70823	0.54177
3478	H2	H	0.79179	0.79168	0.54178
3479	H2	H	0.95823	0.79173	0.70823
3480	H2	H	0.95824	0.70825	0.79176
3481	H2	H	0.54174	0.79176	0.79177
3482	H2	H	0.54173	0.70823	0.70823
3483	H2	H	0.79175	0.95824	0.70824
3484	H2	H	0.70824	0.95824	0.79176
3485	H2	H	0.70823	0.54172	0.70822
3486	H2	H	0.79177	0.54173	0.79178
3487	H2	H	0.54178	0.54177	0.54173
3488	H2	H	0.95823	0.95824	0.54174
3489	H2	H	0.95823	0.54176	0.95825
3490	H2	H	0.54176	0.95824	0.95824
3491	O1	O	0.58524	0.50000	0.54765
3492	O1	O	0.58524	0.75000	0.79765
3493	O1	O	0.83524	0.50000	0.79765
3494	O1	O	0.83524	0.75000	0.54765
3495	O1	O	0.91476	0.50000	0.54765
3496	O1	O	0.91476	0.75000	0.79765
3497	O1	O	0.66476	0.50000	0.79765
3498	O1	O	0.66476	0.75000	0.54765
3499	O1	O	0.91476	0.50000	0.95235
3500	O1	O	0.91476	0.75000	0.70235
3501	O1	O	0.66476	0.50000	0.70235
3502	O1	O	0.66476	0.75000	0.95235
3503	O1	O	0.58524	0.50000	0.95235
3504	O1	O	0.58524	0.75000	0.70235
3505	O1	O	0.83524	0.50000	0.70235
3506	O1	O	0.83524	0.75000	0.95235
3507	O1	O	0.54765	0.58524	0.50000
3508	O1	O	0.54765	0.83524	0.75000
3509	O1	O	0.79765	0.58524	0.75000
3510	O1	O	0.79765	0.83524	0.50000

3511	O1	O	0.54765	0.91476	0.50000
3512	O1	O	0.54765	0.66476	0.75000
3513	O1	O	0.79765	0.91476	0.75000
3514	O1	O	0.79765	0.66476	0.50000
3515	O1	O	0.95235	0.91476	0.50000
3516	O1	O	0.95235	0.66476	0.75000
3517	O1	O	0.70235	0.91476	0.75000
3518	O1	O	0.70235	0.66476	0.50000
3519	O1	O	0.95235	0.58524	0.50000
3520	O1	O	0.95235	0.83524	0.75000
3521	O1	O	0.70235	0.58524	0.75000
3522	O1	O	0.70235	0.83524	0.50000
3523	O1	O	0.50000	0.54765	0.58524
3524	O1	O	0.50000	0.79765	0.83524
3525	O1	O	0.75000	0.54765	0.83524
3526	O1	O	0.75000	0.79765	0.58524
3527	O1	O	0.50000	0.54765	0.91476
3528	O1	O	0.50000	0.79765	0.66476
3529	O1	O	0.75000	0.54765	0.66476
3530	O1	O	0.75000	0.79765	0.91476
3531	O1	O	0.50000	0.95235	0.91476
3532	O1	O	0.50000	0.70235	0.66476
3533	O1	O	0.75000	0.95235	0.66476
3534	O1	O	0.75000	0.70235	0.91476
3535	O1	O	0.50000	0.95235	0.58524
3536	O1	O	0.50000	0.70235	0.83524
3537	O1	O	0.75000	0.95235	0.83524
3538	O1	O	0.75000	0.70235	0.58524
3539	O1	O	0.50000	0.58524	0.95235
3540	O1	O	0.50000	0.83524	0.70235
3541	O1	O	0.75000	0.58524	0.70235
3542	O1	O	0.75000	0.83524	0.95235
3543	O1	O	0.50000	0.91476	0.95235
3544	O1	O	0.50000	0.66476	0.70235
3545	O1	O	0.75000	0.91476	0.70235
3546	O1	O	0.75000	0.66476	0.95235
3547	O1	O	0.50000	0.91476	0.54765
3548	O1	O	0.50000	0.66476	0.79765
3549	O1	O	0.75000	0.91476	0.79765
3550	O1	O	0.75000	0.66476	0.54765
3551	O1	O	0.50000	0.58524	0.54765
3552	O1	O	0.50000	0.83524	0.79765
3553	O1	O	0.75000	0.58524	0.79765
3554	O1	O	0.75000	0.83524	0.54765
3555	O1	O	0.58524	0.54765	0.50000
3556	O1	O	0.58524	0.79765	0.75000
3557	O1	O	0.83524	0.54765	0.75000
3558	O1	O	0.83524	0.79765	0.50000
3559	O1	O	0.91476	0.54765	0.50000
3560	O1	O	0.91476	0.79765	0.75000
3561	O1	O	0.66476	0.54765	0.75000
3562	O1	O	0.66476	0.79765	0.50000
3563	O1	O	0.91476	0.95235	0.50000
3564	O1	O	0.91476	0.70235	0.75000

3565	O1	O	0.66476	0.95235	0.75000
3566	O1	O	0.66476	0.70235	0.50000
3567	O1	O	0.58524	0.95235	0.50000
3568	O1	O	0.58524	0.70235	0.75000
3569	O1	O	0.83524	0.95235	0.75000
3570	O1	O	0.83524	0.70235	0.50000
3571	O1	O	0.54765	0.50000	0.91476
3572	O1	O	0.54765	0.75000	0.66476
3573	O1	O	0.79765	0.50000	0.66476
3574	O1	O	0.79765	0.75000	0.91476
3575	O1	O	0.54765	0.50000	0.58524
3576	O1	O	0.54765	0.75000	0.83524
3577	O1	O	0.79765	0.50000	0.83524
3578	O1	O	0.79765	0.75000	0.58524
3579	O1	O	0.95235	0.50000	0.58524
3580	O1	O	0.95235	0.75000	0.83524
3581	O1	O	0.70235	0.50000	0.83524
3582	O1	O	0.70235	0.75000	0.58524
3583	O1	O	0.95235	0.50000	0.91476
3584	O1	O	0.95235	0.75000	0.66476
3585	O1	O	0.70235	0.50000	0.66476
3586	O1	O	0.70235	0.75000	0.91476
3587	O2	O	0.52805	0.97195	0.97195
3588	O2	O	0.52805	0.72195	0.72195
3589	O2	O	0.77805	0.97195	0.72195
3590	O2	O	0.77805	0.72195	0.97195
3591	O2	O	0.97195	0.52805	0.97195
3592	O2	O	0.97195	0.77805	0.72195
3593	O2	O	0.72195	0.52805	0.72195
3594	O2	O	0.72195	0.77805	0.97195
3595	O2	O	0.97195	0.97195	0.52805
3596	O2	O	0.97195	0.72195	0.77805
3597	O2	O	0.72195	0.97195	0.77805
3598	O2	O	0.72195	0.72195	0.52805
3599	O2	O	0.52805	0.52805	0.52805
3600	O2	O	0.52805	0.77805	0.77805
3601	O2	O	0.77805	0.52805	0.77805
3602	O2	O	0.77805	0.77805	0.52805
3603	O1	O	0.97195	0.52805	0.52805
3604	O1	O	0.97195	0.77805	0.77805
3605	O1	O	0.72195	0.52805	0.77805
3606	O1	O	0.72195	0.77805	0.52805
3607	O1	O	0.52805	0.97195	0.52805
3608	O1	O	0.52805	0.72195	0.77805
3609	O1	O	0.77805	0.97195	0.77805
3610	O1	O	0.77805	0.72195	0.52805
3611	O1	O	0.97195	0.97195	0.97195
3612	O1	O	0.97195	0.72195	0.72195
3613	O1	O	0.72195	0.97195	0.72195
3614	O1	O	0.72195	0.72195	0.97195
3615	O1	O	0.52805	0.52805	0.97195
3616	O1	O	0.52805	0.77805	0.72195
3617	O1	O	0.77805	0.52805	0.72195
3618	O1	O	0.77805	0.77805	0.97195

3619	Zr1	Zr	0.55994	0.50000	0.50000
3620	Zr1	Zr	0.55994	0.75000	0.75000
3621	Zr1	Zr	0.80994	0.50000	0.75000
3622	Zr1	Zr	0.80994	0.75000	0.50000
3623	Zr1	Zr	0.94006	0.50000	0.50000
3624	Zr1	Zr	0.94006	0.75000	0.75000
3625	Zr1	Zr	0.69006	0.50000	0.75000
3626	Zr1	Zr	0.69006	0.75000	0.50000
3627	Zr1	Zr	0.50000	0.55994	0.50000
3628	Zr1	Zr	0.50000	0.80994	0.75000
3629	Zr1	Zr	0.75000	0.55994	0.75000
3630	Zr1	Zr	0.75000	0.80994	0.50000
3631	Zr1	Zr	0.50000	0.94006	0.50000
3632	Zr1	Zr	0.50000	0.69006	0.75000
3633	Zr1	Zr	0.75000	0.94006	0.75000
3634	Zr1	Zr	0.75000	0.69006	0.50000
3635	Zr1	Zr	0.50000	0.50000	0.55994
3636	Zr1	Zr	0.50000	0.75000	0.80994
3637	Zr1	Zr	0.75000	0.50000	0.80994
3638	Zr1	Zr	0.75000	0.75000	0.55994
3639	Zr1	Zr	0.50000	0.50000	0.94006
3640	Zr1	Zr	0.50000	0.75000	0.69006
3641	Zr1	Zr	0.75000	0.50000	0.69006
3642	Zr1	Zr	0.75000	0.75000	0.94006
3643	H3	H	0.45705	0.60274	0.49978
3644	H3	H	0.39442	0.54974	0.49979
3645	H3	H	0.35697	0.70270	0.50002
3646	H3	H	0.28977	0.64489	0.49991
3647	H3	H	0.64386	0.45601	0.24981
3648	H3	H	0.70804	0.39699	0.24968
3649	H3	H	0.54730	0.35566	0.25047
3650	H3	H	0.60740	0.29773	0.25004
