# Supplementary material for: Defect-induced tuning of polarity-dependent adsorption in hydrophobic–hydrophilic UiO-66
Source: Commun Chem. 2022 Oct 7;5:120. doi: 10.1038/s42004-022-00742-z (PMC9814431; doi:10.1038/s42004-022-00742-z)
Supplement: Supplementary file 7 — Supplementary Data 4 [file 42004_2022_742_MOESM7_ESM.pdf]

```
data_UiO-66_8

_cell_length_a                    41.4008
_cell_length_b                    41.4008
_cell_length_c                    41.4008
_cell_angle_alpha                 90.0000
_cell_angle_beta                  90.0000
_cell_angle_gamma                 90.0000


_symmetry_equiv_pos_as_xyz 'x,y,z'

loop_
_atom_site_label
_atom_site_type_symbol
_atom_site_fract_x
_atom_site_fract_y
_atom_site_fract_z
C2   C     0.13375   0.00000   0.09220
C2   C     0.13375   0.25000   0.34220
C2   C     0.38375   0.00000   0.34220
C2   C     0.38375   0.25000   0.09220
C2   C     0.36625   0.00000   0.09220
C2   C     0.36625   0.25000   0.34220
C2   C     0.11625   0.00000   0.34220
C2   C     0.11625   0.25000   0.09220
C2   C     0.36625   0.00000   0.40780
C2   C     0.36625   0.25000   0.15780
C2   C     0.11625   0.00000   0.15780
C2   C     0.11625   0.25000   0.40780
C2   C     0.13375   0.00000   0.40780
C2   C     0.13375   0.25000   0.15780
C2   C     0.38375   0.00000   0.15780
C2   C     0.38375   0.25000   0.40780
C2   C     0.09220   0.38375   0.25000
C2   C     0.34220   0.13375   0.25000
C2   C     0.34220   0.38375   0.00000
C2   C     0.09220   0.36625   0.00000
C2   C     0.09220   0.11625   0.25000
C2   C     0.34220   0.36625   0.25000
C2   C     0.34220   0.11625   0.00000
C2   C     0.40780   0.36625   0.00000
C2   C     0.40780   0.11625   0.25000
C2   C     0.15780   0.36625   0.25000
C2   C     0.40780   0.13375   0.00000
C2   C     0.40780   0.38375   0.25000
C2   C     0.15780   0.13375   0.25000
C2   C     0.15780   0.38375   0.00000
C2   C     0.00000   0.09220   0.13375
C2   C     0.00000   0.34220   0.38375
C2   C     0.25000   0.09220   0.38375
C2   C     0.25000   0.34220   0.13375
C2   C     0.00000   0.09220   0.36625
C2   C     0.00000   0.34220   0.11625
```

| | | | | | |
|---|---|---|---|---|---|
| 55 | C2 | C | 0.25000 | 0.09220 | 0.11625 |
| 56 | C2 | C | 0.25000 | 0.34220 | 0.36625 |
| 57 | C2 | C | 0.00000 | 0.40780 | 0.36625 |
| 58 | C2 | C | 0.00000 | 0.15780 | 0.11625 |
| 59 | C2 | C | 0.25000 | 0.40780 | 0.11625 |
| 60 | C2 | C | 0.25000 | 0.15780 | 0.36625 |
| 61 | C2 | C | 0.00000 | 0.40780 | 0.13375 |
| 62 | C2 | C | 0.00000 | 0.15780 | 0.38375 |
| 63 | C2 | C | 0.25000 | 0.40780 | 0.38375 |
| 64 | C2 | C | 0.25000 | 0.15780 | 0.13375 |
| 65 | C2 | C | 0.00000 | 0.13375 | 0.40780 |
| 66 | C2 | C | 0.00000 | 0.38375 | 0.15780 |
| 67 | C2 | C | 0.25000 | 0.13375 | 0.15780 |
| 68 | C2 | C | 0.25000 | 0.38375 | 0.40780 |
| 69 | C2 | C | 0.00000 | 0.36625 | 0.40780 |
| 70 | C2 | C | 0.00000 | 0.11625 | 0.15780 |
| 71 | C2 | C | 0.25000 | 0.36625 | 0.15780 |
| 72 | C2 | C | 0.25000 | 0.11625 | 0.40780 |
| 73 | C2 | C | 0.00000 | 0.36625 | 0.09220 |
| 74 | C2 | C | 0.00000 | 0.11625 | 0.34220 |
| 75 | C2 | C | 0.25000 | 0.36625 | 0.34220 |
| 76 | C2 | C | 0.25000 | 0.11625 | 0.09220 |
| 77 | C2 | C | 0.00000 | 0.13375 | 0.09220 |
| 78 | C2 | C | 0.00000 | 0.38375 | 0.34220 |
| 79 | C2 | C | 0.25000 | 0.13375 | 0.34220 |
| 80 | C2 | C | 0.25000 | 0.38375 | 0.09220 |
| 81 | C2 | C | 0.13375 | 0.34220 | 0.25000 |
| 82 | C2 | C | 0.38375 | 0.09220 | 0.25000 |
| 83 | C2 | C | 0.38375 | 0.34220 | 0.00000 |
| 84 | C2 | C | 0.36625 | 0.09220 | 0.00000 |
| 85 | C2 | C | 0.36625 | 0.34220 | 0.25000 |
| 86 | C2 | C | 0.11625 | 0.09220 | 0.25000 |
| 87 | C2 | C | 0.11625 | 0.34220 | 0.00000 |
| 88 | C2 | C | 0.36625 | 0.40780 | 0.00000 |
| 89 | C2 | C | 0.36625 | 0.15780 | 0.25000 |
| 90 | C2 | C | 0.11625 | 0.40780 | 0.25000 |
| 91 | C2 | C | 0.13375 | 0.40780 | 0.00000 |
| 92 | C2 | C | 0.13375 | 0.15780 | 0.25000 |
| 93 | C2 | C | 0.38375 | 0.40780 | 0.25000 |
| 94 | C2 | C | 0.38375 | 0.15780 | 0.00000 |
| 95 | C2 | C | 0.09220 | 0.00000 | 0.36625 |
| 96 | C2 | C | 0.09220 | 0.25000 | 0.11625 |
| 97 | C2 | C | 0.34220 | 0.00000 | 0.11625 |
| 98 | C2 | C | 0.34220 | 0.25000 | 0.36625 |
| 99 | C2 | C | 0.09220 | 0.00000 | 0.13375 |
| 100 | C2 | C | 0.09220 | 0.25000 | 0.38375 |
| 101 | C2 | C | 0.34220 | 0.00000 | 0.38375 |
| 102 | C2 | C | 0.34220 | 0.25000 | 0.13375 |
| 103 | C2 | C | 0.40780 | 0.00000 | 0.13375 |
| 104 | C2 | C | 0.40780 | 0.25000 | 0.38375 |
| 105 | C2 | C | 0.15780 | 0.00000 | 0.38375 |
| 106 | C2 | C | 0.15780 | 0.25000 | 0.13375 |
| 107 | C2 | C | 0.40780 | 0.00000 | 0.36625 |
| 108 | C2 | C | 0.40780 | 0.25000 | 0.11625 |

| | | | | | |
|---|---|---|---|---|---|
| 109 | C2 | C | 0.15780 | 0.00000 | 0.11625 |
| 110 | C2 | C | 0.15780 | 0.25000 | 0.36625 |
| 111 | C1 | C | 0.07680 | 0.00000 | 0.42320 |
| 112 | C1 | C | 0.07680 | 0.25000 | 0.17320 |
| 113 | C1 | C | 0.32680 | 0.00000 | 0.17320 |
| 114 | C1 | C | 0.32680 | 0.25000 | 0.42320 |
| 115 | C1 | C | 0.42320 | 0.00000 | 0.42320 |
| 116 | C1 | C | 0.42320 | 0.25000 | 0.17320 |
| 117 | C1 | C | 0.17320 | 0.00000 | 0.17320 |
| 118 | C1 | C | 0.17320 | 0.25000 | 0.42320 |
| 119 | C1 | C | 0.42320 | 0.00000 | 0.07680 |
| 120 | C1 | C | 0.42320 | 0.25000 | 0.32680 |
| 121 | C1 | C | 0.17320 | 0.00000 | 0.32680 |
| 122 | C1 | C | 0.17320 | 0.25000 | 0.07680 |
| 123 | C1 | C | 0.07680 | 0.00000 | 0.07680 |
| 124 | C1 | C | 0.07680 | 0.25000 | 0.32680 |
| 125 | C1 | C | 0.32680 | 0.00000 | 0.32680 |
| 126 | C1 | C | 0.32680 | 0.25000 | 0.07680 |
| 127 | C1 | C | 0.42320 | 0.07680 | 0.00000 |
| 128 | C1 | C | 0.42320 | 0.32680 | 0.25000 |
| 129 | C1 | C | 0.17320 | 0.07680 | 0.25000 |
| 130 | C1 | C | 0.17320 | 0.32680 | 0.00000 |
| 131 | C1 | C | 0.42320 | 0.42320 | 0.00000 |
| 132 | C1 | C | 0.42320 | 0.17320 | 0.25000 |
| 133 | C1 | C | 0.17320 | 0.42320 | 0.25000 |
| 134 | C1 | C | 0.07680 | 0.42320 | 0.00000 |
| 135 | C1 | C | 0.07680 | 0.17320 | 0.25000 |
| 136 | C1 | C | 0.32680 | 0.42320 | 0.25000 |
| 137 | C1 | C | 0.32680 | 0.17320 | 0.00000 |
| 138 | C1 | C | 0.07680 | 0.32680 | 0.25000 |
| 139 | C1 | C | 0.32680 | 0.07680 | 0.25000 |
| 140 | C1 | C | 0.32680 | 0.32680 | 0.00000 |
| 141 | C1 | C | 0.00000 | 0.42320 | 0.07680 |
| 142 | C1 | C | 0.00000 | 0.17320 | 0.32680 |
| 143 | C1 | C | 0.25000 | 0.42320 | 0.32680 |
| 144 | C1 | C | 0.25000 | 0.17320 | 0.07680 |
| 145 | C1 | C | 0.00000 | 0.42320 | 0.42320 |
| 146 | C1 | C | 0.00000 | 0.17320 | 0.17320 |
| 147 | C1 | C | 0.25000 | 0.42320 | 0.17320 |
| 148 | C1 | C | 0.25000 | 0.17320 | 0.42320 |
| 149 | C1 | C | 0.00000 | 0.07680 | 0.42320 |
| 150 | C1 | C | 0.00000 | 0.32680 | 0.17320 |
| 151 | C1 | C | 0.25000 | 0.07680 | 0.17320 |
| 152 | C1 | C | 0.25000 | 0.32680 | 0.42320 |
| 153 | C1 | C | 0.00000 | 0.07680 | 0.07680 |
| 154 | C1 | C | 0.00000 | 0.32680 | 0.32680 |
| 155 | C1 | C | 0.25000 | 0.07680 | 0.32680 |
| 156 | C1 | C | 0.25000 | 0.32680 | 0.07680 |
| 157 | C3 | C | 0.10250 | 0.00000 | 0.10250 |
| 158 | C3 | C | 0.10250 | 0.25000 | 0.35250 |
| 159 | C3 | C | 0.35250 | 0.00000 | 0.35250 |
| 160 | C3 | C | 0.35250 | 0.25000 | 0.10250 |
| 161 | C3 | C | 0.39750 | 0.00000 | 0.10250 |
| 162 | C3 | C | 0.39750 | 0.25000 | 0.35250 |

| | | | | | |
|---|---|---|---|---|---|
| 163 | C3 | C | 0.14750 | 0.00000 | 0.35250 |
| 164 | C3 | C | 0.14750 | 0.25000 | 0.10250 |
| 165 | C3 | C | 0.39750 | 0.00000 | 0.39750 |
| 166 | C3 | C | 0.39750 | 0.25000 | 0.14750 |
| 167 | C3 | C | 0.14750 | 0.00000 | 0.14750 |
| 168 | C3 | C | 0.14750 | 0.25000 | 0.39750 |
| 169 | C3 | C | 0.10250 | 0.00000 | 0.39750 |
| 170 | C3 | C | 0.10250 | 0.25000 | 0.14750 |
| 171 | C3 | C | 0.35250 | 0.00000 | 0.14750 |
| 172 | C3 | C | 0.35250 | 0.25000 | 0.39750 |
| 173 | C3 | C | 0.10250 | 0.35250 | 0.25000 |
| 174 | C3 | C | 0.35250 | 0.10250 | 0.25000 |
| 175 | C3 | C | 0.35250 | 0.35250 | 0.00000 |
| 176 | C3 | C | 0.10250 | 0.39750 | 0.00000 |
| 177 | C3 | C | 0.10250 | 0.14750 | 0.25000 |
| 178 | C3 | C | 0.35250 | 0.39750 | 0.25000 |
| 179 | C3 | C | 0.35250 | 0.14750 | 0.00000 |
| 180 | C3 | C | 0.39750 | 0.39750 | 0.00000 |
| 181 | C3 | C | 0.39750 | 0.14750 | 0.25000 |
| 182 | C3 | C | 0.14750 | 0.39750 | 0.25000 |
| 183 | C3 | C | 0.39750 | 0.10250 | 0.00000 |
| 184 | C3 | C | 0.39750 | 0.35250 | 0.25000 |
| 185 | C3 | C | 0.14750 | 0.10250 | 0.25000 |
| 186 | C3 | C | 0.14750 | 0.35250 | 0.00000 |
| 187 | C3 | C | 0.00000 | 0.10250 | 0.10250 |
| 188 | C3 | C | 0.00000 | 0.35250 | 0.35250 |
| 189 | C3 | C | 0.25000 | 0.10250 | 0.35250 |
| 190 | C3 | C | 0.25000 | 0.35250 | 0.10250 |
| 191 | C3 | C | 0.00000 | 0.10250 | 0.39750 |
| 192 | C3 | C | 0.00000 | 0.35250 | 0.14750 |
| 193 | C3 | C | 0.25000 | 0.10250 | 0.14750 |
| 194 | C3 | C | 0.25000 | 0.35250 | 0.39750 |
| 195 | C3 | C | 0.00000 | 0.39750 | 0.39750 |
| 196 | C3 | C | 0.00000 | 0.14750 | 0.14750 |
| 197 | C3 | C | 0.25000 | 0.39750 | 0.14750 |
| 198 | C3 | C | 0.25000 | 0.14750 | 0.39750 |
| 199 | C3 | C | 0.00000 | 0.39750 | 0.10250 |
| 200 | C3 | C | 0.00000 | 0.14750 | 0.35250 |
| 201 | C3 | C | 0.25000 | 0.39750 | 0.35250 |
| 202 | C3 | C | 0.25000 | 0.14750 | 0.10250 |
| 203 | H1 | H | 0.13926 | 0.49997 | 0.06655 |
| 204 | H1 | H | 0.13926 | 0.24997 | 0.31655 |
| 205 | H1 | H | 0.38926 | 0.49997 | 0.31655 |
| 206 | H1 | H | 0.38926 | 0.24997 | 0.06655 |
| 207 | H1 | H | 0.36074 | 0.00003 | 0.06655 |
| 208 | H1 | H | 0.36074 | 0.25004 | 0.31655 |
| 209 | H1 | H | 0.11074 | 0.00003 | 0.31655 |
| 210 | H1 | H | 0.11074 | 0.25004 | 0.06655 |
| 211 | H1 | H | 0.36074 | 0.49996 | 0.43345 |
| 212 | H1 | H | 0.36074 | 0.24997 | 0.18345 |
| 213 | H1 | H | 0.11074 | 0.49997 | 0.18345 |
| 214 | H1 | H | 0.11074 | 0.24997 | 0.43346 |
| 215 | H1 | H | 0.13926 | 0.00003 | 0.43345 |
| 216 | H1 | H | 0.13926 | 0.25003 | 0.18346 |

| | | | | | |
|---|---|---|---|---|---|
| 217 | H1 | H | 0.38926 | 0.00003 | 0.18345 |
| 218 | H1 | H | 0.38926 | 0.25004 | 0.43346 |
| 219 | H1 | H | 0.06655 | 0.13926 | 0.49996 |
| 220 | H1 | H | 0.06655 | 0.38926 | 0.24997 |
| 221 | H1 | H | 0.31655 | 0.13926 | 0.24996 |
| 222 | H1 | H | 0.31654 | 0.38926 | 0.49997 |
| 223 | H1 | H | 0.06655 | 0.36074 | 0.00003 |
| 224 | H1 | H | 0.06655 | 0.11074 | 0.25004 |
| 225 | H1 | H | 0.31655 | 0.36074 | 0.25003 |
| 226 | H1 | H | 0.31655 | 0.11074 | 0.00004 |
| 227 | H1 | H | 0.43345 | 0.36074 | 0.49996 |
| 228 | H1 | H | 0.43345 | 0.11074 | 0.24997 |
| 229 | H1 | H | 0.18345 | 0.36074 | 0.24996 |
| 230 | H1 | H | 0.18345 | 0.11074 | 0.49997 |
| 231 | H1 | H | 0.43345 | 0.13926 | 0.00003 |
| 232 | H1 | H | 0.43345 | 0.38926 | 0.25004 |
| 233 | H1 | H | 0.18345 | 0.13926 | 0.25003 |
| 234 | H1 | H | 0.18345 | 0.38926 | 0.00004 |
| 235 | H1 | H | 0.49997 | 0.06655 | 0.13926 |
| 236 | H1 | H | 0.49996 | 0.31655 | 0.38926 |
| 237 | H1 | H | 0.24997 | 0.06655 | 0.38926 |
| 238 | H1 | H | 0.24996 | 0.31655 | 0.13926 |
| 239 | H1 | H | 0.00003 | 0.06655 | 0.36074 |
| 240 | H1 | H | 0.00003 | 0.31655 | 0.11074 |
| 241 | H1 | H | 0.25003 | 0.06655 | 0.11074 |
| 242 | H1 | H | 0.25004 | 0.31655 | 0.36074 |
| 243 | H1 | H | 0.49997 | 0.43345 | 0.36074 |
| 244 | H1 | H | 0.49997 | 0.18345 | 0.11074 |
| 245 | H1 | H | 0.24997 | 0.43345 | 0.11074 |
| 246 | H1 | H | 0.24997 | 0.18345 | 0.36074 |
| 247 | H1 | H | 0.00003 | 0.43345 | 0.13926 |
| 248 | H1 | H | 0.00003 | 0.18345 | 0.38926 |
| 249 | H1 | H | 0.25004 | 0.43345 | 0.38926 |
| 250 | H1 | H | 0.25003 | 0.18346 | 0.13926 |
| 251 | H1 | H | 0.00003 | 0.13926 | 0.43345 |
| 252 | H1 | H | 0.00003 | 0.38926 | 0.18345 |
| 253 | H1 | H | 0.25003 | 0.13926 | 0.18345 |
| 254 | H1 | H | 0.25004 | 0.38926 | 0.43345 |
| 255 | H1 | H | 0.49996 | 0.36074 | 0.43345 |
| 256 | H1 | H | 0.49997 | 0.11074 | 0.18345 |
| 257 | H1 | H | 0.24997 | 0.36074 | 0.18345 |
| 258 | H1 | H | 0.24997 | 0.11074 | 0.43345 |
| 259 | H1 | H | 0.00003 | 0.36074 | 0.06655 |
| 260 | H1 | H | 0.00003 | 0.11074 | 0.31655 |
| 261 | H1 | H | 0.25004 | 0.36074 | 0.31655 |
| 262 | H1 | H | 0.25003 | 0.11074 | 0.06654 |
| 263 | H1 | H | 0.49997 | 0.13926 | 0.06655 |
| 264 | H1 | H | 0.49997 | 0.38926 | 0.31655 |
| 265 | H1 | H | 0.24997 | 0.13926 | 0.31655 |
| 266 | H1 | H | 0.24997 | 0.38926 | 0.06654 |
| 267 | H1 | H | 0.13926 | 0.06655 | 0.49997 |
| 268 | H1 | H | 0.13926 | 0.31655 | 0.24996 |
| 269 | H1 | H | 0.38926 | 0.06655 | 0.24997 |
| 270 | H1 | H | 0.38926 | 0.31655 | 0.49996 |

| | | | | | |
|---|---|---|---|---|---|
| 271 | H1 | H | 0.36074 | 0.06655 | 0.00003 |
| 272 | H1 | H | 0.36074 | 0.31655 | 0.25003 |
| 273 | H1 | H | 0.11074 | 0.06655 | 0.25003 |
| 274 | H1 | H | 0.11074 | 0.31655 | 0.00003 |
| 275 | H1 | H | 0.36074 | 0.43345 | 0.49997 |
| 276 | H1 | H | 0.36074 | 0.18345 | 0.24997 |
| 277 | H1 | H | 0.11074 | 0.43345 | 0.24997 |
| 278 | H1 | H | 0.11074 | 0.18345 | 0.49996 |
| 279 | H1 | H | 0.13926 | 0.43345 | 0.00004 |
| 280 | H1 | H | 0.13926 | 0.18346 | 0.25003 |
| 281 | H1 | H | 0.38926 | 0.43345 | 0.25004 |
| 282 | H1 | H | 0.38926 | 0.18346 | 0.00003 |
| 283 | H1 | H | 0.06655 | 0.00003 | 0.36074 |
| 284 | H1 | H | 0.06655 | 0.25004 | 0.11074 |
| 285 | H1 | H | 0.31655 | 0.00003 | 0.11074 |
| 286 | H1 | H | 0.31655 | 0.25004 | 0.36074 |
| 287 | H1 | H | 0.06655 | 0.49996 | 0.13926 |
| 288 | H1 | H | 0.06655 | 0.24997 | 0.38926 |
| 289 | H1 | H | 0.31655 | 0.49996 | 0.38926 |
| 290 | H1 | H | 0.31655 | 0.24997 | 0.13926 |
| 291 | H1 | H | 0.43345 | 0.00003 | 0.13926 |
| 292 | H1 | H | 0.43345 | 0.25004 | 0.38926 |
| 293 | H1 | H | 0.18345 | 0.00003 | 0.38926 |
| 294 | H1 | H | 0.18345 | 0.25003 | 0.13926 |
| 295 | H1 | H | 0.43345 | 0.49996 | 0.36074 |
| 296 | H1 | H | 0.43345 | 0.24997 | 0.11074 |
| 297 | H1 | H | 0.18345 | 0.49996 | 0.11074 |
| 298 | H1 | H | 0.18345 | 0.24997 | 0.36074 |
| 299 | H2 | H | 0.20825 | 0.29176 | 0.45824 |
| 300 | H2 | H | 0.29174 | 0.20825 | 0.45823 |
| 301 | H2 | H | 0.20827 | 0.20823 | 0.04177 |
| 302 | H2 | H | 0.29179 | 0.29168 | 0.04178 |
| 303 | H2 | H | 0.45823 | 0.29173 | 0.20823 |
| 304 | H2 | H | 0.45824 | 0.20825 | 0.29176 |
| 305 | H2 | H | 0.04174 | 0.29176 | 0.29177 |
| 306 | H2 | H | 0.04173 | 0.20823 | 0.20823 |
| 307 | H2 | H | 0.29175 | 0.45824 | 0.20824 |
| 308 | H2 | H | 0.20824 | 0.45824 | 0.29176 |
| 309 | H2 | H | 0.20823 | 0.04172 | 0.20822 |
| 310 | H2 | H | 0.29177 | 0.04173 | 0.29178 |
| 311 | H2 | H | 0.04178 | 0.04177 | 0.04173 |
| 312 | H2 | H | 0.45823 | 0.45824 | 0.04174 |
| 313 | H2 | H | 0.45823 | 0.04176 | 0.45825 |
| 314 | H2 | H | 0.04176 | 0.45824 | 0.45824 |
| 315 | O1 | O | 0.08524 | 0.00000 | 0.04765 |
| 316 | O1 | O | 0.08524 | 0.25000 | 0.29765 |
| 317 | O1 | O | 0.33524 | 0.00000 | 0.29765 |
| 318 | O1 | O | 0.33524 | 0.25000 | 0.04765 |
| 319 | O1 | O | 0.41476 | 0.00000 | 0.04765 |
| 320 | O1 | O | 0.41476 | 0.25000 | 0.29765 |
| 321 | O1 | O | 0.16476 | 0.00000 | 0.29765 |
| 322 | O1 | O | 0.16476 | 0.25000 | 0.04765 |
| 323 | O1 | O | 0.41476 | 0.00000 | 0.45235 |
| 324 | O1 | O | 0.41476 | 0.25000 | 0.20235 |

| | | | | | |
|---|---|---|---|---|---|
| 325 | O1 | O | 0.16476 | 0.00000 | 0.20235 |
| 326 | O1 | O | 0.16476 | 0.25000 | 0.45235 |
| 327 | O1 | O | 0.08524 | 0.00000 | 0.45235 |
| 328 | O1 | O | 0.08524 | 0.25000 | 0.20235 |
| 329 | O1 | O | 0.33524 | 0.00000 | 0.20235 |
| 330 | O1 | O | 0.33524 | 0.25000 | 0.45235 |
| 331 | O3 | O | 0.04765 | 0.08524 | 0.00000 |
| 332 | O1 | O | 0.04765 | 0.33524 | 0.25000 |
| 333 | O1 | O | 0.29765 | 0.08524 | 0.25000 |
| 334 | O1 | O | 0.29765 | 0.33524 | 0.00000 |
| 335 | O1 | O | 0.04765 | 0.41476 | 0.00000 |
| 336 | O1 | O | 0.04765 | 0.16476 | 0.25000 |
| 337 | O1 | O | 0.29765 | 0.41476 | 0.25000 |
| 338 | O1 | O | 0.29765 | 0.16476 | 0.00000 |
| 339 | O1 | O | 0.45235 | 0.41476 | 0.00000 |
| 340 | O1 | O | 0.45235 | 0.16476 | 0.25000 |
| 341 | O1 | O | 0.20235 | 0.41476 | 0.25000 |
| 342 | O3 | O | 0.20235 | 0.16476 | 0.00000 |
| 343 | O1 | O | 0.45235 | 0.08524 | 0.00000 |
| 344 | O1 | O | 0.45235 | 0.33524 | 0.25000 |
| 345 | O1 | O | 0.20235 | 0.08524 | 0.25000 |
| 346 | O1 | O | 0.20235 | 0.33524 | 0.00000 |
| 347 | O1 | O | 0.00000 | 0.04765 | 0.08524 |
| 348 | O1 | O | 0.00000 | 0.29765 | 0.33524 |
| 349 | O1 | O | 0.25000 | 0.04765 | 0.33524 |
| 350 | O1 | O | 0.25000 | 0.29765 | 0.08524 |
| 351 | O1 | O | 0.00000 | 0.04765 | 0.41476 |
| 352 | O1 | O | 0.00000 | 0.29765 | 0.16476 |
| 353 | O1 | O | 0.25000 | 0.04765 | 0.16476 |
| 354 | O1 | O | 0.25000 | 0.29765 | 0.41476 |
| 355 | O1 | O | 0.00000 | 0.45235 | 0.41476 |
| 356 | O1 | O | 0.00000 | 0.20235 | 0.16476 |
| 357 | O1 | O | 0.25000 | 0.45235 | 0.16476 |
| 358 | O1 | O | 0.25000 | 0.20235 | 0.41476 |
| 359 | O1 | O | 0.00000 | 0.45235 | 0.08524 |
| 360 | O1 | O | 0.00000 | 0.20235 | 0.33524 |
| 361 | O1 | O | 0.25000 | 0.45235 | 0.33524 |
| 362 | O1 | O | 0.25000 | 0.20235 | 0.08524 |
| 363 | O1 | O | 0.00000 | 0.08524 | 0.45235 |
| 364 | O1 | O | 0.00000 | 0.33524 | 0.20235 |
| 365 | O1 | O | 0.25000 | 0.08524 | 0.20235 |
| 366 | O1 | O | 0.25000 | 0.33524 | 0.45235 |
| 367 | O1 | O | 0.00000 | 0.41476 | 0.45235 |
| 368 | O1 | O | 0.00000 | 0.16476 | 0.20235 |
| 369 | O1 | O | 0.25000 | 0.41476 | 0.20235 |
| 370 | O1 | O | 0.25000 | 0.16476 | 0.45235 |
| 371 | O1 | O | 0.00000 | 0.41476 | 0.04765 |
| 372 | O1 | O | 0.00000 | 0.16476 | 0.29765 |
| 373 | O1 | O | 0.25000 | 0.41476 | 0.29765 |
| 374 | O1 | O | 0.25000 | 0.16476 | 0.04765 |
| 375 | O1 | O | 0.00000 | 0.08524 | 0.04765 |
| 376 | O1 | O | 0.00000 | 0.33524 | 0.29765 |
| 377 | O1 | O | 0.25000 | 0.08524 | 0.29765 |
| 378 | O1 | O | 0.25000 | 0.33524 | 0.04765 |

| | | | | | |
|---|---|---|---|---|---|
| 379 | O3 | O | 0.08524 | 0.04765 | 0.00000 |
| 380 | O1 | O | 0.08524 | 0.29765 | 0.25000 |
| 381 | O1 | O | 0.33524 | 0.04765 | 0.25000 |
| 382 | O1 | O | 0.33524 | 0.29765 | 0.00000 |
| 383 | O1 | O | 0.41476 | 0.04765 | 0.00000 |
| 384 | O1 | O | 0.41476 | 0.29765 | 0.25000 |
| 385 | O1 | O | 0.16476 | 0.04765 | 0.25000 |
| 386 | O1 | O | 0.16476 | 0.29765 | 0.00000 |
| 387 | O1 | O | 0.41476 | 0.45235 | 0.00000 |
| 388 | O1 | O | 0.41476 | 0.20235 | 0.25000 |
| 389 | O1 | O | 0.16476 | 0.45235 | 0.25000 |
| 390 | O3 | O | 0.16476 | 0.20235 | 0.00000 |
| 391 | O1 | O | 0.08524 | 0.45235 | 0.00000 |
| 392 | O1 | O | 0.08524 | 0.20235 | 0.25000 |
| 393 | O1 | O | 0.33524 | 0.45235 | 0.25000 |
| 394 | O1 | O | 0.33524 | 0.20235 | 0.00000 |
| 395 | O1 | O | 0.04765 | 0.00000 | 0.41476 |
| 396 | O1 | O | 0.04765 | 0.25000 | 0.16476 |
| 397 | O1 | O | 0.29765 | 0.00000 | 0.16476 |
| 398 | O1 | O | 0.29765 | 0.25000 | 0.41476 |
| 399 | O1 | O | 0.04765 | 0.00000 | 0.08524 |
| 400 | O1 | O | 0.04765 | 0.25000 | 0.33524 |
| 401 | O1 | O | 0.29765 | 0.00000 | 0.33524 |
| 402 | O1 | O | 0.29765 | 0.25000 | 0.08524 |
| 403 | O1 | O | 0.45235 | 0.00000 | 0.08524 |
| 404 | O1 | O | 0.45235 | 0.25000 | 0.33524 |
| 405 | O1 | O | 0.20235 | 0.00000 | 0.33524 |
| 406 | O1 | O | 0.20235 | 0.25000 | 0.08524 |
| 407 | O1 | O | 0.45235 | 0.00000 | 0.41476 |
| 408 | O1 | O | 0.45235 | 0.25000 | 0.16476 |
| 409 | O1 | O | 0.20235 | 0.00000 | 0.16476 |
| 410 | O1 | O | 0.20235 | 0.25000 | 0.41476 |
| 411 | O2 | O | 0.02805 | 0.47195 | 0.47195 |
| 412 | O2 | O | 0.02805 | 0.22195 | 0.22195 |
| 413 | O2 | O | 0.27805 | 0.47195 | 0.22195 |
| 414 | O2 | O | 0.27805 | 0.22195 | 0.47195 |
| 415 | O2 | O | 0.47195 | 0.02805 | 0.47195 |
| 416 | O2 | O | 0.47195 | 0.27805 | 0.22195 |
| 417 | O2 | O | 0.22195 | 0.02805 | 0.22195 |
| 418 | O2 | O | 0.22195 | 0.27805 | 0.47195 |
| 419 | O2 | O | 0.47195 | 0.47195 | 0.02805 |
| 420 | O2 | O | 0.47195 | 0.22195 | 0.27805 |
| 421 | O2 | O | 0.22195 | 0.47195 | 0.27805 |
| 422 | O2 | O | 0.22195 | 0.22195 | 0.02805 |
| 423 | O2 | O | 0.02805 | 0.02805 | 0.02805 |
| 424 | O2 | O | 0.02805 | 0.27805 | 0.27805 |
| 425 | O2 | O | 0.27805 | 0.02805 | 0.27805 |
| 426 | O2 | O | 0.27805 | 0.27805 | 0.02805 |
| 427 | O1 | O | 0.47195 | 0.02805 | 0.02805 |
| 428 | O1 | O | 0.47195 | 0.27805 | 0.27805 |
| 429 | O1 | O | 0.22195 | 0.02805 | 0.27805 |
| 430 | O1 | O | 0.22195 | 0.27805 | 0.02805 |
| 431 | O1 | O | 0.02805 | 0.47195 | 0.02805 |
| 432 | O1 | O | 0.02805 | 0.22195 | 0.27805 |

| | | | | | |
|---|---|---|---|---|---|
| 433 | O1 | O | 0.27805 | 0.47195 | 0.27805 |
| 434 | O1 | O | 0.27805 | 0.22195 | 0.02805 |
| 435 | O1 | O | 0.47195 | 0.47195 | 0.47195 |
| 436 | O1 | O | 0.47195 | 0.22195 | 0.22195 |
| 437 | O1 | O | 0.22195 | 0.47195 | 0.22195 |
| 438 | O1 | O | 0.22195 | 0.22195 | 0.47195 |
| 439 | O1 | O | 0.02805 | 0.02805 | 0.47195 |
| 440 | O1 | O | 0.02805 | 0.27805 | 0.22195 |
| 441 | O1 | O | 0.27805 | 0.02805 | 0.22195 |
| 442 | O1 | O | 0.27805 | 0.27805 | 0.47195 |
| 443 | Zr1 | Zr | 0.05994 | 0.00000 | 0.00000 |
| 444 | Zr1 | Zr | 0.05994 | 0.25000 | 0.25000 |
| 445 | Zr1 | Zr | 0.30994 | 0.00000 | 0.25000 |
| 446 | Zr1 | Zr | 0.30994 | 0.25000 | 0.00000 |
| 447 | Zr1 | Zr | 0.44006 | 0.00000 | 0.00000 |
| 448 | Zr1 | Zr | 0.44006 | 0.25000 | 0.25000 |
| 449 | Zr1 | Zr | 0.19006 | 0.00000 | 0.25000 |
| 450 | Zr1 | Zr | 0.19006 | 0.25000 | 0.00000 |
| 451 | Zr1 | Zr | 0.00000 | 0.05994 | 0.00000 |
| 452 | Zr1 | Zr | 0.00000 | 0.30994 | 0.25000 |
| 453 | Zr1 | Zr | 0.25000 | 0.05994 | 0.25000 |
| 454 | Zr1 | Zr | 0.25000 | 0.30994 | 0.00000 |
| 455 | Zr1 | Zr | 0.00000 | 0.44006 | 0.00000 |
| 456 | Zr1 | Zr | 0.00000 | 0.19006 | 0.25000 |
| 457 | Zr1 | Zr | 0.25000 | 0.44006 | 0.25000 |
| 458 | Zr1 | Zr | 0.25000 | 0.19006 | 0.00000 |
| 459 | Zr1 | Zr | 0.00000 | 0.00000 | 0.05994 |
| 460 | Zr1 | Zr | 0.00000 | 0.25000 | 0.30994 |
| 461 | Zr1 | Zr | 0.25000 | 0.00000 | 0.30994 |
| 462 | Zr1 | Zr | 0.25000 | 0.25000 | 0.05994 |
| 463 | Zr1 | Zr | 0.00000 | 0.00000 | 0.44006 |
| 464 | Zr1 | Zr | 0.00000 | 0.25000 | 0.19006 |
| 465 | Zr1 | Zr | 0.25000 | 0.00000 | 0.19006 |
| 466 | Zr1 | Zr | 0.25000 | 0.25000 | 0.44006 |
| 467 | C2 | C | 0.13375 | 0.00000 | 0.59220 |
| 468 | C2 | C | 0.13375 | 0.25000 | 0.84220 |
| 469 | C2 | C | 0.38375 | 0.00000 | 0.84220 |
| 470 | C2 | C | 0.38375 | 0.25000 | 0.59220 |
| 471 | C2 | C | 0.36625 | 0.00000 | 0.59220 |
| 472 | C2 | C | 0.36625 | 0.25000 | 0.84220 |
| 473 | C2 | C | 0.11625 | 0.00000 | 0.84220 |
| 474 | C2 | C | 0.11625 | 0.25000 | 0.59220 |
| 475 | C2 | C | 0.36625 | 0.00000 | 0.90780 |
| 476 | C2 | C | 0.36625 | 0.25000 | 0.65780 |
| 477 | C2 | C | 0.11625 | 0.00000 | 0.65780 |
| 478 | C2 | C | 0.11625 | 0.25000 | 0.90780 |
| 479 | C2 | C | 0.13375 | 0.00000 | 0.90780 |
| 480 | C2 | C | 0.13375 | 0.25000 | 0.65780 |
| 481 | C2 | C | 0.38375 | 0.00000 | 0.65780 |
| 482 | C2 | C | 0.38375 | 0.25000 | 0.90780 |
| 483 | C2 | C | 0.09220 | 0.13375 | 0.50000 |
| 484 | C2 | C | 0.34220 | 0.13375 | 0.75000 |
| 485 | C2 | C | 0.34220 | 0.38375 | 0.50000 |
| 486 | C2 | C | 0.09220 | 0.36625 | 0.50000 |

| | | | | | |
|---|---|---|---|---|---|
| 487 | C2 | C | 0.09220 | 0.11625 | 0.75000 |
| 488 | C2 | C | 0.34220 | 0.36625 | 0.75000 |
| 489 | C2 | C | 0.34220 | 0.11625 | 0.50000 |
| 490 | C2 | C | 0.40780 | 0.36625 | 0.50000 |
| 491 | C2 | C | 0.40780 | 0.11625 | 0.75000 |
| 492 | C2 | C | 0.15780 | 0.11625 | 0.50000 |
| 493 | C2 | C | 0.40780 | 0.13375 | 0.50000 |
| 494 | C2 | C | 0.40780 | 0.38375 | 0.75000 |
| 495 | C2 | C | 0.15780 | 0.13375 | 0.75000 |
| 496 | C2 | C | 0.15780 | 0.38375 | 0.50000 |
| 497 | C2 | C | 0.00000 | 0.09220 | 0.63375 |
| 498 | C2 | C | 0.00000 | 0.34220 | 0.88375 |
| 499 | C2 | C | 0.25000 | 0.09220 | 0.88375 |
| 500 | C2 | C | 0.25000 | 0.34220 | 0.63375 |
| 501 | C2 | C | 0.00000 | 0.09220 | 0.86625 |
| 502 | C2 | C | 0.00000 | 0.34220 | 0.61625 |
| 503 | C2 | C | 0.25000 | 0.09220 | 0.61625 |
| 504 | C2 | C | 0.25000 | 0.34220 | 0.86625 |
| 505 | C2 | C | 0.00000 | 0.40780 | 0.86625 |
| 506 | C2 | C | 0.00000 | 0.15780 | 0.61625 |
| 507 | C2 | C | 0.25000 | 0.40780 | 0.61625 |
| 508 | C2 | C | 0.25000 | 0.15780 | 0.86625 |
| 509 | C2 | C | 0.00000 | 0.40780 | 0.63375 |
| 510 | C2 | C | 0.00000 | 0.15780 | 0.88375 |
| 511 | C2 | C | 0.25000 | 0.40780 | 0.88375 |
| 512 | C2 | C | 0.25000 | 0.15780 | 0.63375 |
| 513 | C2 | C | 0.00000 | 0.13375 | 0.90780 |
| 514 | C2 | C | 0.00000 | 0.38375 | 0.65780 |
| 515 | C2 | C | 0.25000 | 0.13375 | 0.65780 |
| 516 | C2 | C | 0.25000 | 0.38375 | 0.90780 |
| 517 | C2 | C | 0.00000 | 0.36625 | 0.90780 |
| 518 | C2 | C | 0.00000 | 0.11625 | 0.65780 |
| 519 | C2 | C | 0.25000 | 0.36625 | 0.65780 |
| 520 | C2 | C | 0.25000 | 0.11625 | 0.90780 |
| 521 | C2 | C | 0.00000 | 0.36625 | 0.59220 |
| 522 | C2 | C | 0.00000 | 0.11625 | 0.84220 |
| 523 | C2 | C | 0.25000 | 0.36625 | 0.84220 |
| 524 | C2 | C | 0.25000 | 0.11625 | 0.59220 |
| 525 | C2 | C | 0.00000 | 0.13375 | 0.59220 |
| 526 | C2 | C | 0.00000 | 0.38375 | 0.84220 |
| 527 | C2 | C | 0.25000 | 0.13375 | 0.84220 |
| 528 | C2 | C | 0.25000 | 0.38375 | 0.59220 |
| 529 | C2 | C | 0.13375 | 0.09220 | 0.50000 |
| 530 | C2 | C | 0.38375 | 0.09220 | 0.75000 |
| 531 | C2 | C | 0.38375 | 0.34220 | 0.50000 |
| 532 | C2 | C | 0.36625 | 0.09220 | 0.50000 |
| 533 | C2 | C | 0.36625 | 0.34220 | 0.75000 |
| 534 | C2 | C | 0.11625 | 0.09220 | 0.75000 |
| 535 | C2 | C | 0.11625 | 0.34220 | 0.50000 |
| 536 | C2 | C | 0.36625 | 0.40780 | 0.50000 |
| 537 | C2 | C | 0.36625 | 0.15780 | 0.75000 |
| 538 | C2 | C | 0.11625 | 0.15780 | 0.50000 |
| 539 | C2 | C | 0.13375 | 0.40780 | 0.50000 |
| 540 | C2 | C | 0.13375 | 0.15780 | 0.75000 |

| | | | | | |
|---|---|---|---|---|---|
| 541 | C2 | C | 0.38375 | 0.40780 | 0.75000 |
| 542 | C2 | C | 0.38375 | 0.15780 | 0.50000 |
| 543 | C2 | C | 0.09220 | 0.00000 | 0.86625 |
| 544 | C2 | C | 0.09220 | 0.25000 | 0.61625 |
| 545 | C2 | C | 0.34220 | 0.00000 | 0.61625 |
| 546 | C2 | C | 0.34220 | 0.25000 | 0.86625 |
| 547 | C2 | C | 0.09220 | 0.00000 | 0.63375 |
| 548 | C2 | C | 0.09220 | 0.25000 | 0.88375 |
| 549 | C2 | C | 0.34220 | 0.00000 | 0.88375 |
| 550 | C2 | C | 0.34220 | 0.25000 | 0.63375 |
| 551 | C2 | C | 0.40780 | 0.00000 | 0.63375 |
| 552 | C2 | C | 0.40780 | 0.25000 | 0.88375 |
| 553 | C2 | C | 0.15780 | 0.00000 | 0.88375 |
| 554 | C2 | C | 0.15780 | 0.25000 | 0.63375 |
| 555 | C2 | C | 0.40780 | 0.00000 | 0.86625 |
| 556 | C2 | C | 0.40780 | 0.25000 | 0.61625 |
| 557 | C2 | C | 0.15780 | 0.00000 | 0.61625 |
| 558 | C2 | C | 0.15780 | 0.25000 | 0.86625 |
| 559 | C1 | C | 0.07680 | 0.00000 | 0.92320 |
| 560 | C1 | C | 0.07680 | 0.25000 | 0.67320 |
| 561 | C1 | C | 0.32680 | 0.00000 | 0.67320 |
| 562 | C1 | C | 0.32680 | 0.25000 | 0.92320 |
| 563 | C1 | C | 0.42320 | 0.00000 | 0.92320 |
| 564 | C1 | C | 0.42320 | 0.25000 | 0.67320 |
| 565 | C1 | C | 0.17320 | 0.00000 | 0.67320 |
| 566 | C1 | C | 0.17320 | 0.25000 | 0.92320 |
| 567 | C1 | C | 0.42320 | 0.00000 | 0.57680 |
| 568 | C1 | C | 0.42320 | 0.25000 | 0.82680 |
| 569 | C1 | C | 0.17320 | 0.00000 | 0.82680 |
| 570 | C1 | C | 0.17320 | 0.25000 | 0.57680 |
| 571 | C1 | C | 0.07680 | 0.00000 | 0.57680 |
| 572 | C1 | C | 0.07680 | 0.25000 | 0.82680 |
| 573 | C1 | C | 0.32680 | 0.00000 | 0.82680 |
| 574 | C1 | C | 0.32680 | 0.25000 | 0.57680 |
| 575 | C1 | C | 0.42320 | 0.07680 | 0.50000 |
| 576 | C1 | C | 0.42320 | 0.32680 | 0.75000 |
| 577 | C1 | C | 0.17320 | 0.07680 | 0.75000 |
| 578 | C1 | C | 0.17320 | 0.32680 | 0.50000 |
| 579 | C1 | C | 0.42320 | 0.42320 | 0.50000 |
| 580 | C1 | C | 0.42320 | 0.17320 | 0.75000 |
| 581 | C1 | C | 0.17320 | 0.17320 | 0.50000 |
| 582 | C1 | C | 0.07680 | 0.42320 | 0.50000 |
| 583 | C1 | C | 0.07680 | 0.17320 | 0.75000 |
| 584 | C1 | C | 0.32680 | 0.42320 | 0.75000 |
| 585 | C1 | C | 0.32680 | 0.17320 | 0.50000 |
| 586 | C1 | C | 0.07680 | 0.07680 | 0.50000 |
| 587 | C1 | C | 0.32680 | 0.07680 | 0.75000 |
| 588 | C1 | C | 0.32680 | 0.32680 | 0.50000 |
| 589 | C1 | C | 0.00000 | 0.42320 | 0.57680 |
| 590 | C1 | C | 0.00000 | 0.17320 | 0.82680 |
| 591 | C1 | C | 0.25000 | 0.42320 | 0.82680 |
| 592 | C1 | C | 0.25000 | 0.17320 | 0.57680 |
| 593 | C1 | C | 0.00000 | 0.42320 | 0.92320 |
| 594 | C1 | C | 0.00000 | 0.17320 | 0.67320 |

| | | | | | |
|---|---|---|---|---|---|
| 595 | C1 | C | 0.25000 | 0.42320 | 0.67320 |
| 596 | C1 | C | 0.25000 | 0.17320 | 0.92320 |
| 597 | C1 | C | 0.00000 | 0.07680 | 0.92320 |
| 598 | C1 | C | 0.00000 | 0.32680 | 0.67320 |
| 599 | C1 | C | 0.25000 | 0.07680 | 0.67320 |
| 600 | C1 | C | 0.25000 | 0.32680 | 0.92320 |
| 601 | C1 | C | 0.00000 | 0.07680 | 0.57680 |
| 602 | C1 | C | 0.00000 | 0.32680 | 0.82680 |
| 603 | C1 | C | 0.25000 | 0.07680 | 0.82680 |
| 604 | C1 | C | 0.25000 | 0.32680 | 0.57680 |
| 605 | C3 | C | 0.10250 | 0.00000 | 0.60250 |
| 606 | C3 | C | 0.10250 | 0.25000 | 0.85250 |
| 607 | C3 | C | 0.35250 | 0.00000 | 0.85250 |
| 608 | C3 | C | 0.35250 | 0.25000 | 0.60250 |
| 609 | C3 | C | 0.39750 | 0.00000 | 0.60250 |
| 610 | C3 | C | 0.39750 | 0.25000 | 0.85250 |
| 611 | C3 | C | 0.14750 | 0.00000 | 0.85250 |
| 612 | C3 | C | 0.14750 | 0.25000 | 0.60250 |
| 613 | C3 | C | 0.39750 | 0.00000 | 0.89750 |
| 614 | C3 | C | 0.39750 | 0.25000 | 0.64750 |
| 615 | C3 | C | 0.14750 | 0.00000 | 0.64750 |
| 616 | C3 | C | 0.14750 | 0.25000 | 0.89750 |
| 617 | C3 | C | 0.10250 | 0.00000 | 0.89750 |
| 618 | C3 | C | 0.10250 | 0.25000 | 0.64750 |
| 619 | C3 | C | 0.35250 | 0.00000 | 0.64750 |
| 620 | C3 | C | 0.35250 | 0.25000 | 0.89750 |
| 621 | C3 | C | 0.10250 | 0.10250 | 0.50000 |
| 622 | C3 | C | 0.35250 | 0.10250 | 0.75000 |
| 623 | C3 | C | 0.35250 | 0.35250 | 0.50000 |
| 624 | C3 | C | 0.10250 | 0.39750 | 0.50000 |
| 625 | C3 | C | 0.10250 | 0.14750 | 0.75000 |
| 626 | C3 | C | 0.35250 | 0.39750 | 0.75000 |
| 627 | C3 | C | 0.35250 | 0.14750 | 0.50000 |
| 628 | C3 | C | 0.39750 | 0.39750 | 0.50000 |
| 629 | C3 | C | 0.39750 | 0.14750 | 0.75000 |
| 630 | C3 | C | 0.14750 | 0.14750 | 0.50000 |
| 631 | C3 | C | 0.39750 | 0.10250 | 0.50000 |
| 632 | C3 | C | 0.39750 | 0.35250 | 0.75000 |
| 633 | C3 | C | 0.14750 | 0.10250 | 0.75000 |
| 634 | C3 | C | 0.14750 | 0.35250 | 0.50000 |
| 635 | C3 | C | 0.00000 | 0.10250 | 0.60250 |
| 636 | C3 | C | 0.00000 | 0.35250 | 0.85250 |
| 637 | C3 | C | 0.25000 | 0.10250 | 0.85250 |
| 638 | C3 | C | 0.25000 | 0.35250 | 0.60250 |
| 639 | C3 | C | 0.00000 | 0.10250 | 0.89750 |
| 640 | C3 | C | 0.00000 | 0.35250 | 0.64750 |
| 641 | C3 | C | 0.25000 | 0.10250 | 0.64750 |
| 642 | C3 | C | 0.25000 | 0.35250 | 0.89750 |
| 643 | C3 | C | 0.00000 | 0.39750 | 0.89750 |
| 644 | C3 | C | 0.00000 | 0.14750 | 0.64750 |
| 645 | C3 | C | 0.25000 | 0.39750 | 0.64750 |
| 646 | C3 | C | 0.25000 | 0.14750 | 0.89750 |
| 647 | C3 | C | 0.00000 | 0.39750 | 0.60250 |
| 648 | C3 | C | 0.00000 | 0.14750 | 0.85250 |

| | | | | | |
|---|---|---|---|---|---|
| 649 | C3 | C | 0.25000 | 0.39750 | 0.85250 |
| 650 | C3 | C | 0.25000 | 0.14750 | 0.60250 |
| 651 | H1 | H | 0.13926 | 0.49997 | 0.56655 |
| 652 | H1 | H | 0.13926 | 0.24997 | 0.81655 |
| 653 | H1 | H | 0.38926 | 0.49997 | 0.81655 |
| 654 | H1 | H | 0.38926 | 0.24997 | 0.56655 |
| 655 | H1 | H | 0.36074 | 0.00003 | 0.56655 |
| 656 | H1 | H | 0.36074 | 0.25004 | 0.81655 |
| 657 | H1 | H | 0.11074 | 0.00003 | 0.81655 |
| 658 | H1 | H | 0.11074 | 0.25004 | 0.56655 |
| 659 | H1 | H | 0.36074 | 0.49996 | 0.93345 |
| 660 | H1 | H | 0.36074 | 0.24997 | 0.68345 |
| 661 | H1 | H | 0.11074 | 0.49997 | 0.68345 |
| 662 | H1 | H | 0.11074 | 0.24997 | 0.93346 |
| 663 | H1 | H | 0.13926 | 0.00003 | 0.93345 |
| 664 | H1 | H | 0.13926 | 0.25003 | 0.68346 |
| 665 | H1 | H | 0.38926 | 0.00003 | 0.68345 |
| 666 | H1 | H | 0.38926 | 0.25004 | 0.93346 |
| 667 | H1 | H | 0.31655 | 0.13926 | 0.74996 |
| 668 | H1 | H | 0.31654 | 0.38926 | 0.99997 |
| 669 | H1 | H | 0.06655 | 0.36074 | 0.50003 |
| 670 | H1 | H | 0.06655 | 0.11074 | 0.75004 |
| 671 | H1 | H | 0.31655 | 0.36074 | 0.75003 |
| 672 | H1 | H | 0.31655 | 0.11074 | 0.50004 |
| 673 | H1 | H | 0.43345 | 0.36074 | 0.99996 |
| 674 | H1 | H | 0.43345 | 0.11074 | 0.74997 |
| 675 | H1 | H | 0.43345 | 0.13926 | 0.50003 |
| 676 | H1 | H | 0.43345 | 0.38926 | 0.75004 |
| 677 | H1 | H | 0.18345 | 0.13926 | 0.75003 |
| 678 | H1 | H | 0.18345 | 0.38926 | 0.50004 |
| 679 | H1 | H | 0.49997 | 0.06655 | 0.63926 |
| 680 | H1 | H | 0.49996 | 0.31655 | 0.88926 |
| 681 | H1 | H | 0.24997 | 0.06655 | 0.88926 |
| 682 | H1 | H | 0.24996 | 0.31655 | 0.63926 |
| 683 | H1 | H | 0.00003 | 0.06655 | 0.86074 |
| 684 | H1 | H | 0.00003 | 0.31655 | 0.61074 |
| 685 | H1 | H | 0.25003 | 0.06655 | 0.61074 |
| 686 | H1 | H | 0.25004 | 0.31655 | 0.86074 |
| 687 | H1 | H | 0.49997 | 0.43345 | 0.86074 |
| 688 | H1 | H | 0.49997 | 0.18345 | 0.61074 |
| 689 | H1 | H | 0.24997 | 0.43345 | 0.61074 |
| 690 | H1 | H | 0.24997 | 0.18345 | 0.86074 |
| 691 | H1 | H | 0.00003 | 0.43345 | 0.63926 |
| 692 | H1 | H | 0.00003 | 0.18345 | 0.88926 |
| 693 | H1 | H | 0.25004 | 0.43345 | 0.88926 |
| 694 | H1 | H | 0.25003 | 0.18346 | 0.63926 |
| 695 | H1 | H | 0.00003 | 0.13926 | 0.93345 |
| 696 | H1 | H | 0.00003 | 0.38926 | 0.68345 |
| 697 | H1 | H | 0.25003 | 0.13926 | 0.68345 |
| 698 | H1 | H | 0.25004 | 0.38926 | 0.93345 |
| 699 | H1 | H | 0.49996 | 0.36074 | 0.93345 |
| 700 | H1 | H | 0.49997 | 0.11074 | 0.68345 |
| 701 | H1 | H | 0.24997 | 0.36074 | 0.68345 |
| 702 | H1 | H | 0.24997 | 0.11074 | 0.93345 |

| | | | | | |
|---|---|---|---|---|---|
| 703 | H1 | H | 0.00003 | 0.36074 | 0.56655 |
| 704 | H1 | H | 0.00003 | 0.11074 | 0.81655 |
| 705 | H1 | H | 0.25004 | 0.36074 | 0.81655 |
| 706 | H1 | H | 0.25003 | 0.11074 | 0.56654 |
| 707 | H1 | H | 0.49997 | 0.13926 | 0.56655 |
| 708 | H1 | H | 0.49997 | 0.38926 | 0.81655 |
| 709 | H1 | H | 0.24997 | 0.13926 | 0.81655 |
| 710 | H1 | H | 0.24997 | 0.38926 | 0.56654 |
| 711 | H1 | H | 0.38926 | 0.06655 | 0.74997 |
| 712 | H1 | H | 0.38926 | 0.31655 | 0.99996 |
| 713 | H1 | H | 0.36074 | 0.06655 | 0.50003 |
| 714 | H1 | H | 0.36074 | 0.31655 | 0.75003 |
| 715 | H1 | H | 0.11074 | 0.06655 | 0.75003 |
| 716 | H1 | H | 0.11074 | 0.31655 | 0.50003 |
| 717 | H1 | H | 0.36074 | 0.43345 | 0.99997 |
| 718 | H1 | H | 0.36074 | 0.18345 | 0.74997 |
| 719 | H1 | H | 0.13926 | 0.43345 | 0.50004 |
| 720 | H1 | H | 0.13926 | 0.18346 | 0.75003 |
| 721 | H1 | H | 0.38926 | 0.43345 | 0.75004 |
| 722 | H1 | H | 0.38926 | 0.18346 | 0.50003 |
| 723 | H1 | H | 0.06655 | 0.00003 | 0.86074 |
| 724 | H1 | H | 0.06655 | 0.25004 | 0.61074 |
| 725 | H1 | H | 0.31655 | 0.00003 | 0.61074 |
| 726 | H1 | H | 0.31655 | 0.25004 | 0.86074 |
| 727 | H1 | H | 0.06655 | 0.49996 | 0.63926 |
| 728 | H1 | H | 0.06655 | 0.24997 | 0.88926 |
| 729 | H1 | H | 0.31655 | 0.49996 | 0.88926 |
| 730 | H1 | H | 0.31655 | 0.24997 | 0.63926 |
| 731 | H1 | H | 0.43345 | 0.00003 | 0.63926 |
| 732 | H1 | H | 0.43345 | 0.25004 | 0.88926 |
| 733 | H1 | H | 0.18345 | 0.00003 | 0.88926 |
| 734 | H1 | H | 0.18345 | 0.25003 | 0.63926 |
| 735 | H1 | H | 0.43345 | 0.49996 | 0.86074 |
| 736 | H1 | H | 0.43345 | 0.24997 | 0.61074 |
| 737 | H1 | H | 0.18345 | 0.49996 | 0.61074 |
| 738 | H1 | H | 0.18345 | 0.24997 | 0.86074 |
| 739 | H2 | H | 0.20825 | 0.29176 | 0.95824 |
| 740 | H2 | H | 0.29174 | 0.20825 | 0.95823 |
| 741 | H2 | H | 0.20827 | 0.20823 | 0.54177 |
| 742 | H2 | H | 0.29179 | 0.29168 | 0.54178 |
| 743 | H2 | H | 0.45823 | 0.29173 | 0.70823 |
| 744 | H2 | H | 0.45824 | 0.20825 | 0.79176 |
| 745 | H2 | H | 0.04174 | 0.29176 | 0.79177 |
| 746 | H2 | H | 0.04173 | 0.20823 | 0.70823 |
| 747 | H2 | H | 0.29175 | 0.45824 | 0.70824 |
| 748 | H2 | H | 0.20824 | 0.45824 | 0.79176 |
| 749 | H2 | H | 0.20823 | 0.04172 | 0.70822 |
| 750 | H2 | H | 0.29177 | 0.04173 | 0.79178 |
| 751 | H2 | H | 0.04178 | 0.04177 | 0.54173 |
| 752 | H2 | H | 0.45823 | 0.45824 | 0.54174 |
| 753 | H2 | H | 0.45823 | 0.04176 | 0.95825 |
| 754 | H2 | H | 0.04176 | 0.45824 | 0.95824 |
| 755 | O1 | O | 0.08524 | 0.00000 | 0.54765 |
| 756 | O1 | O | 0.08524 | 0.25000 | 0.79765 |

| | | | | | |
|---|---|---|---|---|---|
| 757 | O1 | O | 0.33524 | 0.00000 | 0.79765 |
| 758 | O1 | O | 0.33524 | 0.25000 | 0.54765 |
| 759 | O1 | O | 0.41476 | 0.00000 | 0.54765 |
| 760 | O1 | O | 0.41476 | 0.25000 | 0.79765 |
| 761 | O1 | O | 0.16476 | 0.00000 | 0.79765 |
| 762 | O1 | O | 0.16476 | 0.25000 | 0.54765 |
| 763 | O1 | O | 0.41476 | 0.00000 | 0.95235 |
| 764 | O1 | O | 0.41476 | 0.25000 | 0.70235 |
| 765 | O1 | O | 0.16476 | 0.00000 | 0.70235 |
| 766 | O1 | O | 0.16476 | 0.25000 | 0.95235 |
| 767 | O1 | O | 0.08524 | 0.00000 | 0.95235 |
| 768 | O1 | O | 0.08524 | 0.25000 | 0.70235 |
| 769 | O1 | O | 0.33524 | 0.00000 | 0.70235 |
| 770 | O1 | O | 0.33524 | 0.25000 | 0.95235 |
| 771 | O1 | O | 0.04765 | 0.08524 | 0.50000 |
| 772 | O3 | O | 0.04765 | 0.33524 | 0.75000 |
| 773 | O1 | O | 0.29765 | 0.08524 | 0.75000 |
| 774 | O1 | O | 0.29765 | 0.33524 | 0.50000 |
| 775 | O1 | O | 0.04765 | 0.41476 | 0.50000 |
| 776 | O1 | O | 0.04765 | 0.16476 | 0.75000 |
| 777 | O1 | O | 0.29765 | 0.41476 | 0.75000 |
| 778 | O1 | O | 0.29765 | 0.16476 | 0.50000 |
| 779 | O1 | O | 0.45235 | 0.41476 | 0.50000 |
| 780 | O1 | O | 0.45235 | 0.16476 | 0.75000 |
| 781 | O3 | O | 0.20235 | 0.41476 | 0.75000 |
| 782 | O1 | O | 0.20235 | 0.16476 | 0.50000 |
| 783 | O1 | O | 0.45235 | 0.08524 | 0.50000 |
| 784 | O1 | O | 0.45235 | 0.33524 | 0.75000 |
| 785 | O1 | O | 0.20235 | 0.08524 | 0.75000 |
| 786 | O1 | O | 0.20235 | 0.33524 | 0.50000 |
| 787 | O1 | O | 0.00000 | 0.04765 | 0.58524 |
| 788 | O1 | O | 0.00000 | 0.29765 | 0.83524 |
| 789 | O1 | O | 0.25000 | 0.04765 | 0.83524 |
| 790 | O1 | O | 0.25000 | 0.29765 | 0.58524 |
| 791 | O1 | O | 0.00000 | 0.04765 | 0.91476 |
| 792 | O1 | O | 0.00000 | 0.29765 | 0.66476 |
| 793 | O1 | O | 0.25000 | 0.04765 | 0.66476 |
| 794 | O1 | O | 0.25000 | 0.29765 | 0.91476 |
| 795 | O1 | O | 0.00000 | 0.45235 | 0.91476 |
| 796 | O1 | O | 0.00000 | 0.20235 | 0.66476 |
| 797 | O1 | O | 0.25000 | 0.45235 | 0.66476 |
| 798 | O1 | O | 0.25000 | 0.20235 | 0.91476 |
| 799 | O1 | O | 0.00000 | 0.45235 | 0.58524 |
| 800 | O1 | O | 0.00000 | 0.20235 | 0.83524 |
| 801 | O1 | O | 0.25000 | 0.45235 | 0.83524 |
| 802 | O1 | O | 0.25000 | 0.20235 | 0.58524 |
| 803 | O1 | O | 0.00000 | 0.08524 | 0.95235 |
| 804 | O1 | O | 0.00000 | 0.33524 | 0.70235 |
| 805 | O1 | O | 0.25000 | 0.08524 | 0.70235 |
| 806 | O1 | O | 0.25000 | 0.33524 | 0.95235 |
| 807 | O1 | O | 0.00000 | 0.41476 | 0.95235 |
| 808 | O1 | O | 0.00000 | 0.16476 | 0.70235 |
| 809 | O1 | O | 0.25000 | 0.41476 | 0.70235 |
| 810 | O1 | O | 0.25000 | 0.16476 | 0.95235 |

| | | | | | |
|---|---|---|---|---|---|
| 811 | O1 | O | 0.00000 | 0.41476 | 0.54765 |
| 812 | O1 | O | 0.00000 | 0.16476 | 0.79765 |
| 813 | O1 | O | 0.25000 | 0.41476 | 0.79765 |
| 814 | O1 | O | 0.25000 | 0.16476 | 0.54765 |
| 815 | O1 | O | 0.00000 | 0.08524 | 0.54765 |
| 816 | O1 | O | 0.00000 | 0.33524 | 0.79765 |
| 817 | O1 | O | 0.25000 | 0.08524 | 0.79765 |
| 818 | O1 | O | 0.25000 | 0.33524 | 0.54765 |
| 819 | O1 | O | 0.08524 | 0.04765 | 0.50000 |
| 820 | O3 | O | 0.08524 | 0.29765 | 0.75000 |
| 821 | O1 | O | 0.33524 | 0.04765 | 0.75000 |
| 822 | O1 | O | 0.33524 | 0.29765 | 0.50000 |
| 823 | O1 | O | 0.41476 | 0.04765 | 0.50000 |
| 824 | O1 | O | 0.41476 | 0.29765 | 0.75000 |
| 825 | O1 | O | 0.16476 | 0.04765 | 0.75000 |
| 826 | O1 | O | 0.16476 | 0.29765 | 0.50000 |
| 827 | O1 | O | 0.41476 | 0.45235 | 0.50000 |
| 828 | O1 | O | 0.41476 | 0.20235 | 0.75000 |
| 829 | O3 | O | 0.16476 | 0.45235 | 0.75000 |
| 830 | O1 | O | 0.16476 | 0.20235 | 0.50000 |
| 831 | O1 | O | 0.08524 | 0.45235 | 0.50000 |
| 832 | O1 | O | 0.08524 | 0.20235 | 0.75000 |
| 833 | O1 | O | 0.33524 | 0.45235 | 0.75000 |
| 834 | O1 | O | 0.33524 | 0.20235 | 0.50000 |
| 835 | O1 | O | 0.04765 | 0.00000 | 0.91476 |
| 836 | O1 | O | 0.04765 | 0.25000 | 0.66476 |
| 837 | O1 | O | 0.29765 | 0.00000 | 0.66476 |
| 838 | O1 | O | 0.29765 | 0.25000 | 0.91476 |
| 839 | O1 | O | 0.04765 | 0.00000 | 0.58524 |
| 840 | O1 | O | 0.04765 | 0.25000 | 0.83524 |
| 841 | O1 | O | 0.29765 | 0.00000 | 0.83524 |
| 842 | O1 | O | 0.29765 | 0.25000 | 0.58524 |
| 843 | O1 | O | 0.45235 | 0.00000 | 0.58524 |
| 844 | O1 | O | 0.45235 | 0.25000 | 0.83524 |
| 845 | O1 | O | 0.20235 | 0.00000 | 0.83524 |
| 846 | O1 | O | 0.20235 | 0.25000 | 0.58524 |
| 847 | O1 | O | 0.45235 | 0.00000 | 0.91476 |
| 848 | O1 | O | 0.45235 | 0.25000 | 0.66476 |
| 849 | O1 | O | 0.20235 | 0.00000 | 0.66476 |
| 850 | O1 | O | 0.20235 | 0.25000 | 0.91476 |
| 851 | O2 | O | 0.02805 | 0.47195 | 0.97195 |
| 852 | O2 | O | 0.02805 | 0.22195 | 0.72195 |
| 853 | O2 | O | 0.27805 | 0.47195 | 0.72195 |
| 854 | O2 | O | 0.27805 | 0.22195 | 0.97195 |
| 855 | O2 | O | 0.47195 | 0.02805 | 0.97195 |
| 856 | O2 | O | 0.47195 | 0.27805 | 0.72195 |
| 857 | O2 | O | 0.22195 | 0.02805 | 0.72195 |
| 858 | O2 | O | 0.22195 | 0.27805 | 0.97195 |
| 859 | O2 | O | 0.47195 | 0.47195 | 0.52805 |
| 860 | O2 | O | 0.47195 | 0.22195 | 0.77805 |
| 861 | O2 | O | 0.22195 | 0.47195 | 0.77805 |
| 862 | O2 | O | 0.22195 | 0.22195 | 0.52805 |
| 863 | O2 | O | 0.02805 | 0.02805 | 0.52805 |
| 864 | O2 | O | 0.02805 | 0.27805 | 0.77805 |

| | | | | | |
|---|---|---|---|---|---|
| 865 | O2 | O | 0.27805 | 0.02805 | 0.77805 |
| 866 | O2 | O | 0.27805 | 0.27805 | 0.52805 |
| 867 | O1 | O | 0.47195 | 0.02805 | 0.52805 |
| 868 | O1 | O | 0.47195 | 0.27805 | 0.77805 |
| 869 | O1 | O | 0.22195 | 0.02805 | 0.77805 |
| 870 | O1 | O | 0.22195 | 0.27805 | 0.52805 |
| 871 | O1 | O | 0.02805 | 0.47195 | 0.52805 |
| 872 | O1 | O | 0.02805 | 0.22195 | 0.77805 |
| 873 | O1 | O | 0.27805 | 0.47195 | 0.77805 |
| 874 | O1 | O | 0.27805 | 0.22195 | 0.52805 |
| 875 | O1 | O | 0.47195 | 0.47195 | 0.97195 |
| 876 | O1 | O | 0.47195 | 0.22195 | 0.72195 |
| 877 | O1 | O | 0.22195 | 0.47195 | 0.72195 |
| 878 | O1 | O | 0.22195 | 0.22195 | 0.97195 |
| 879 | O1 | O | 0.02805 | 0.02805 | 0.97195 |
| 880 | O1 | O | 0.02805 | 0.27805 | 0.72195 |
| 881 | O1 | O | 0.27805 | 0.02805 | 0.72195 |
| 882 | O1 | O | 0.27805 | 0.27805 | 0.97195 |
| 883 | Zr1 | Zr | 0.05994 | 0.00000 | 0.50000 |
| 884 | Zr1 | Zr | 0.05994 | 0.25000 | 0.75000 |
| 885 | Zr1 | Zr | 0.30994 | 0.00000 | 0.75000 |
| 886 | Zr1 | Zr | 0.30994 | 0.25000 | 0.50000 |
| 887 | Zr1 | Zr | 0.44006 | 0.00000 | 0.50000 |
| 888 | Zr1 | Zr | 0.44006 | 0.25000 | 0.75000 |
| 889 | Zr1 | Zr | 0.19006 | 0.00000 | 0.75000 |
| 890 | Zr1 | Zr | 0.19006 | 0.25000 | 0.50000 |
| 891 | Zr1 | Zr | 0.00000 | 0.05994 | 0.50000 |
| 892 | Zr1 | Zr | 0.00000 | 0.30994 | 0.75000 |
| 893 | Zr1 | Zr | 0.25000 | 0.05994 | 0.75000 |
| 894 | Zr1 | Zr | 0.25000 | 0.30994 | 0.50000 |
| 895 | Zr1 | Zr | 0.00000 | 0.44006 | 0.50000 |
| 896 | Zr1 | Zr | 0.00000 | 0.19006 | 0.75000 |
| 897 | Zr1 | Zr | 0.25000 | 0.44006 | 0.75000 |
| 898 | Zr1 | Zr | 0.25000 | 0.19006 | 0.50000 |
| 899 | Zr1 | Zr | 0.00000 | 0.00000 | 0.55994 |
| 900 | Zr1 | Zr | 0.00000 | 0.25000 | 0.80994 |
| 901 | Zr1 | Zr | 0.25000 | 0.00000 | 0.80994 |
| 902 | Zr1 | Zr | 0.25000 | 0.25000 | 0.55994 |
| 903 | Zr1 | Zr | 0.00000 | 0.00000 | 0.94006 |
| 904 | Zr1 | Zr | 0.00000 | 0.25000 | 0.69006 |
| 905 | Zr1 | Zr | 0.25000 | 0.00000 | 0.69006 |
| 906 | Zr1 | Zr | 0.25000 | 0.25000 | 0.94006 |
| 907 | C2 | C | 0.13375 | 0.50000 | 0.09220 |
| 908 | C2 | C | 0.13375 | 0.75000 | 0.34220 |
| 909 | C2 | C | 0.38375 | 0.50000 | 0.34220 |
| 910 | C2 | C | 0.38375 | 0.75000 | 0.09220 |
| 911 | C2 | C | 0.36625 | 0.50000 | 0.09220 |
| 912 | C2 | C | 0.36625 | 0.75000 | 0.34220 |
| 913 | C2 | C | 0.11625 | 0.50000 | 0.34220 |
| 914 | C2 | C | 0.11625 | 0.75000 | 0.09220 |
| 915 | C2 | C | 0.36625 | 0.50000 | 0.40780 |
| 916 | C2 | C | 0.36625 | 0.75000 | 0.15780 |
| 917 | C2 | C | 0.11625 | 0.50000 | 0.15780 |
| 918 | C2 | C | 0.11625 | 0.75000 | 0.40780 |

| | | | | | |
|---|---|---|---|---|---|
| 919 | C2 | C | 0.13375 | 0.50000 | 0.40780 |
| 920 | C2 | C | 0.13375 | 0.75000 | 0.15780 |
| 921 | C2 | C | 0.38375 | 0.50000 | 0.15780 |
| 922 | C2 | C | 0.38375 | 0.75000 | 0.40780 |
| 923 | C2 | C | 0.09220 | 0.63375 | 0.00000 |
| 924 | C2 | C | 0.09220 | 0.88375 | 0.25000 |
| 925 | C2 | C | 0.34220 | 0.63375 | 0.25000 |
| 926 | C2 | C | 0.34220 | 0.88375 | 0.00000 |
| 927 | C2 | C | 0.09220 | 0.86625 | 0.00000 |
| 928 | C2 | C | 0.09220 | 0.61625 | 0.25000 |
| 929 | C2 | C | 0.34220 | 0.86625 | 0.25000 |
| 930 | C2 | C | 0.34220 | 0.61625 | 0.00000 |
| 931 | C2 | C | 0.40780 | 0.86625 | 0.00000 |
| 932 | C2 | C | 0.40780 | 0.61625 | 0.25000 |
| 933 | C2 | C | 0.15780 | 0.86625 | 0.25000 |
| 934 | C2 | C | 0.15780 | 0.61625 | 0.00000 |
| 935 | C2 | C | 0.40780 | 0.63375 | 0.00000 |
| 936 | C2 | C | 0.40780 | 0.88375 | 0.25000 |
| 937 | C2 | C | 0.15780 | 0.63375 | 0.25000 |
| 938 | C2 | C | 0.15780 | 0.88375 | 0.00000 |
| 939 | C2 | C | 0.00000 | 0.59220 | 0.13375 |
| 940 | C2 | C | 0.00000 | 0.84220 | 0.38375 |
| 941 | C2 | C | 0.25000 | 0.59220 | 0.38375 |
| 942 | C2 | C | 0.25000 | 0.84220 | 0.13375 |
| 943 | C2 | C | 0.00000 | 0.59220 | 0.36625 |
| 944 | C2 | C | 0.00000 | 0.84220 | 0.11625 |
| 945 | C2 | C | 0.25000 | 0.59220 | 0.11625 |
| 946 | C2 | C | 0.25000 | 0.84220 | 0.36625 |
| 947 | C2 | C | 0.00000 | 0.90780 | 0.36625 |
| 948 | C2 | C | 0.00000 | 0.65780 | 0.11625 |
| 949 | C2 | C | 0.25000 | 0.90780 | 0.11625 |
| 950 | C2 | C | 0.25000 | 0.65780 | 0.36625 |
| 951 | C2 | C | 0.00000 | 0.90780 | 0.13375 |
| 952 | C2 | C | 0.00000 | 0.65780 | 0.38375 |
| 953 | C2 | C | 0.25000 | 0.90780 | 0.38375 |
| 954 | C2 | C | 0.25000 | 0.65780 | 0.13375 |
| 955 | C2 | C | 0.00000 | 0.63375 | 0.40780 |
| 956 | C2 | C | 0.00000 | 0.88375 | 0.15780 |
| 957 | C2 | C | 0.25000 | 0.63375 | 0.15780 |
| 958 | C2 | C | 0.25000 | 0.88375 | 0.40780 |
| 959 | C2 | C | 0.00000 | 0.86625 | 0.40780 |
| 960 | C2 | C | 0.00000 | 0.61625 | 0.15780 |
| 961 | C2 | C | 0.25000 | 0.86625 | 0.15780 |
| 962 | C2 | C | 0.25000 | 0.61625 | 0.40780 |
| 963 | C2 | C | 0.00000 | 0.86625 | 0.09220 |
| 964 | C2 | C | 0.00000 | 0.61625 | 0.34220 |
| 965 | C2 | C | 0.25000 | 0.86625 | 0.34220 |
| 966 | C2 | C | 0.25000 | 0.61625 | 0.09220 |
| 967 | C2 | C | 0.00000 | 0.63375 | 0.09220 |
| 968 | C2 | C | 0.00000 | 0.88375 | 0.34220 |
| 969 | C2 | C | 0.25000 | 0.63375 | 0.34220 |
| 970 | C2 | C | 0.25000 | 0.88375 | 0.09220 |
| 971 | C2 | C | 0.13375 | 0.59220 | 0.00000 |
| 972 | C2 | C | 0.13375 | 0.84220 | 0.25000 |

| | | | | | |
|---|---|---|---|---|---|
| 973 | C2 | C | 0.38375 | 0.59220 | 0.25000 |
| 974 | C2 | C | 0.38375 | 0.84220 | 0.00000 |
| 975 | C2 | C | 0.36625 | 0.59220 | 0.00000 |
| 976 | C2 | C | 0.36625 | 0.84220 | 0.25000 |
| 977 | C2 | C | 0.11625 | 0.59220 | 0.25000 |
| 978 | C2 | C | 0.11625 | 0.84220 | 0.00000 |
| 979 | C2 | C | 0.36625 | 0.90780 | 0.00000 |
| 980 | C2 | C | 0.36625 | 0.65780 | 0.25000 |
| 981 | C2 | C | 0.11625 | 0.90780 | 0.25000 |
| 982 | C2 | C | 0.11625 | 0.65780 | 0.00000 |
| 983 | C2 | C | 0.13375 | 0.90780 | 0.00000 |
| 984 | C2 | C | 0.13375 | 0.65780 | 0.25000 |
| 985 | C2 | C | 0.38375 | 0.90780 | 0.25000 |
| 986 | C2 | C | 0.38375 | 0.65780 | 0.00000 |
| 987 | C2 | C | 0.09220 | 0.50000 | 0.36625 |
| 988 | C2 | C | 0.09220 | 0.75000 | 0.11625 |
| 989 | C2 | C | 0.34220 | 0.50000 | 0.11625 |
| 990 | C2 | C | 0.34220 | 0.75000 | 0.36625 |
| 991 | C2 | C | 0.09220 | 0.50000 | 0.13375 |
| 992 | C2 | C | 0.09220 | 0.75000 | 0.38375 |
| 993 | C2 | C | 0.34220 | 0.50000 | 0.38375 |
| 994 | C2 | C | 0.34220 | 0.75000 | 0.13375 |
| 995 | C2 | C | 0.40780 | 0.50000 | 0.13375 |
| 996 | C2 | C | 0.40780 | 0.75000 | 0.38375 |
| 997 | C2 | C | 0.15780 | 0.50000 | 0.38375 |
| 998 | C2 | C | 0.15780 | 0.75000 | 0.13375 |
| 999 | C2 | C | 0.40780 | 0.50000 | 0.36625 |
| 1000 | C2 | C | 0.40780 | 0.75000 | 0.11625 |
| 1001 | C2 | C | 0.15780 | 0.50000 | 0.11625 |
| 1002 | C2 | C | 0.15780 | 0.75000 | 0.36625 |
| 1003 | C1 | C | 0.07680 | 0.50000 | 0.42320 |
| 1004 | C1 | C | 0.07680 | 0.75000 | 0.17320 |
| 1005 | C1 | C | 0.32680 | 0.50000 | 0.17320 |
| 1006 | C1 | C | 0.32680 | 0.75000 | 0.42320 |
| 1007 | C1 | C | 0.42320 | 0.50000 | 0.42320 |
| 1008 | C1 | C | 0.42320 | 0.75000 | 0.17320 |
| 1009 | C1 | C | 0.17320 | 0.50000 | 0.17320 |
| 1010 | C1 | C | 0.17320 | 0.75000 | 0.42320 |
| 1011 | C1 | C | 0.42320 | 0.50000 | 0.07680 |
| 1012 | C1 | C | 0.42320 | 0.75000 | 0.32680 |
| 1013 | C1 | C | 0.17320 | 0.50000 | 0.32680 |
| 1014 | C1 | C | 0.17320 | 0.75000 | 0.07680 |
| 1015 | C1 | C | 0.07680 | 0.50000 | 0.07680 |
| 1016 | C1 | C | 0.07680 | 0.75000 | 0.32680 |
| 1017 | C1 | C | 0.32680 | 0.50000 | 0.32680 |
| 1018 | C1 | C | 0.32680 | 0.75000 | 0.07680 |
| 1019 | C1 | C | 0.42320 | 0.57680 | 0.00000 |
| 1020 | C1 | C | 0.42320 | 0.82680 | 0.25000 |
| 1021 | C1 | C | 0.17320 | 0.57680 | 0.25000 |
| 1022 | C1 | C | 0.17320 | 0.82680 | 0.00000 |
| 1023 | C1 | C | 0.42320 | 0.92320 | 0.00000 |
| 1024 | C1 | C | 0.42320 | 0.67320 | 0.25000 |
| 1025 | C1 | C | 0.17320 | 0.92320 | 0.25000 |
| 1026 | C1 | C | 0.17320 | 0.67320 | 0.00000 |

| | | | | | |
|---|---|---|---|---|---|
| 1027 | C1 | C | 0.07680 | 0.92320 | 0.00000 |
| 1028 | C1 | C | 0.07680 | 0.67320 | 0.25000 |
| 1029 | C1 | C | 0.32680 | 0.92320 | 0.25000 |
| 1030 | C1 | C | 0.32680 | 0.67320 | 0.00000 |
| 1031 | C1 | C | 0.07680 | 0.57680 | 0.00000 |
| 1032 | C1 | C | 0.07680 | 0.82680 | 0.25000 |
| 1033 | C1 | C | 0.32680 | 0.57680 | 0.25000 |
| 1034 | C1 | C | 0.32680 | 0.82680 | 0.00000 |
| 1035 | C1 | C | 0.00000 | 0.92320 | 0.07680 |
| 1036 | C1 | C | 0.00000 | 0.67320 | 0.32680 |
| 1037 | C1 | C | 0.25000 | 0.92320 | 0.32680 |
| 1038 | C1 | C | 0.25000 | 0.67320 | 0.07680 |
| 1039 | C1 | C | 0.00000 | 0.92320 | 0.42320 |
| 1040 | C1 | C | 0.00000 | 0.67320 | 0.17320 |
| 1041 | C1 | C | 0.25000 | 0.92320 | 0.17320 |
| 1042 | C1 | C | 0.25000 | 0.67320 | 0.42320 |
| 1043 | C1 | C | 0.00000 | 0.57680 | 0.42320 |
| 1044 | C1 | C | 0.00000 | 0.82680 | 0.17320 |
| 1045 | C1 | C | 0.25000 | 0.57680 | 0.17320 |
| 1046 | C1 | C | 0.25000 | 0.82680 | 0.42320 |
| 1047 | C1 | C | 0.00000 | 0.57680 | 0.07680 |
| 1048 | C1 | C | 0.00000 | 0.82680 | 0.32680 |
| 1049 | C1 | C | 0.25000 | 0.57680 | 0.32680 |
| 1050 | C1 | C | 0.25000 | 0.82680 | 0.07680 |
| 1051 | C3 | C | 0.10250 | 0.50000 | 0.10250 |
| 1052 | C3 | C | 0.10250 | 0.75000 | 0.35250 |
| 1053 | C3 | C | 0.35250 | 0.50000 | 0.35250 |
| 1054 | C3 | C | 0.35250 | 0.75000 | 0.10250 |
| 1055 | C3 | C | 0.39750 | 0.50000 | 0.10250 |
| 1056 | C3 | C | 0.39750 | 0.75000 | 0.35250 |
| 1057 | C3 | C | 0.14750 | 0.50000 | 0.35250 |
| 1058 | C3 | C | 0.14750 | 0.75000 | 0.10250 |
| 1059 | C3 | C | 0.39750 | 0.50000 | 0.39750 |
| 1060 | C3 | C | 0.39750 | 0.75000 | 0.14750 |
| 1061 | C3 | C | 0.14750 | 0.50000 | 0.14750 |
| 1062 | C3 | C | 0.14750 | 0.75000 | 0.39750 |
| 1063 | C3 | C | 0.10250 | 0.50000 | 0.39750 |
| 1064 | C3 | C | 0.10250 | 0.75000 | 0.14750 |
| 1065 | C3 | C | 0.35250 | 0.50000 | 0.14750 |
| 1066 | C3 | C | 0.35250 | 0.75000 | 0.39750 |
| 1067 | C3 | C | 0.10250 | 0.60250 | 0.00000 |
| 1068 | C3 | C | 0.10250 | 0.85250 | 0.25000 |
| 1069 | C3 | C | 0.35250 | 0.60250 | 0.25000 |
| 1070 | C3 | C | 0.35250 | 0.85250 | 0.00000 |
| 1071 | C3 | C | 0.10250 | 0.89750 | 0.00000 |
| 1072 | C3 | C | 0.10250 | 0.64750 | 0.25000 |
| 1073 | C3 | C | 0.35250 | 0.89750 | 0.25000 |
| 1074 | C3 | C | 0.35250 | 0.64750 | 0.00000 |
| 1075 | C3 | C | 0.39750 | 0.89750 | 0.00000 |
| 1076 | C3 | C | 0.39750 | 0.64750 | 0.25000 |
| 1077 | C3 | C | 0.14750 | 0.89750 | 0.25000 |
| 1078 | C3 | C | 0.14750 | 0.64750 | 0.00000 |
| 1079 | C3 | C | 0.39750 | 0.60250 | 0.00000 |
| 1080 | C3 | C | 0.39750 | 0.85250 | 0.25000 |

| | | | | | |
|---|---|---|---|---|---|
| 1081 | C3 | C | 0.14750 | 0.60250 | 0.25000 |
| 1082 | C3 | C | 0.14750 | 0.85250 | 0.00000 |
| 1083 | C3 | C | 0.00000 | 0.60250 | 0.10250 |
| 1084 | C3 | C | 0.00000 | 0.85250 | 0.35250 |
| 1085 | C3 | C | 0.25000 | 0.60250 | 0.35250 |
| 1086 | C3 | C | 0.25000 | 0.85250 | 0.10250 |
| 1087 | C3 | C | 0.00000 | 0.60250 | 0.39750 |
| 1088 | C3 | C | 0.00000 | 0.85250 | 0.14750 |
| 1089 | C3 | C | 0.25000 | 0.60250 | 0.14750 |
| 1090 | C3 | C | 0.25000 | 0.85250 | 0.39750 |
| 1091 | C3 | C | 0.00000 | 0.89750 | 0.39750 |
| 1092 | C3 | C | 0.00000 | 0.64750 | 0.14750 |
| 1093 | C3 | C | 0.25000 | 0.89750 | 0.14750 |
| 1094 | C3 | C | 0.25000 | 0.64750 | 0.39750 |
| 1095 | C3 | C | 0.00000 | 0.89750 | 0.10250 |
| 1096 | C3 | C | 0.00000 | 0.64750 | 0.35250 |
| 1097 | C3 | C | 0.25000 | 0.89750 | 0.35250 |
| 1098 | C3 | C | 0.25000 | 0.64750 | 0.10250 |
| 1099 | H1 | H | 0.13926 | 0.99997 | 0.06655 |
| 1100 | H1 | H | 0.13926 | 0.74997 | 0.31655 |
| 1101 | H1 | H | 0.38926 | 0.99997 | 0.31655 |
| 1102 | H1 | H | 0.38926 | 0.74997 | 0.06655 |
| 1103 | H1 | H | 0.36074 | 0.50003 | 0.06655 |
| 1104 | H1 | H | 0.36074 | 0.75004 | 0.31655 |
| 1105 | H1 | H | 0.11074 | 0.50003 | 0.31655 |
| 1106 | H1 | H | 0.11074 | 0.75004 | 0.06655 |
| 1107 | H1 | H | 0.36074 | 0.99996 | 0.43345 |
| 1108 | H1 | H | 0.36074 | 0.74997 | 0.18345 |
| 1109 | H1 | H | 0.11074 | 0.99997 | 0.18345 |
| 1110 | H1 | H | 0.11074 | 0.74997 | 0.43346 |
| 1111 | H1 | H | 0.13926 | 0.50003 | 0.43345 |
| 1112 | H1 | H | 0.13926 | 0.75003 | 0.18346 |
| 1113 | H1 | H | 0.38926 | 0.50003 | 0.18345 |
| 1114 | H1 | H | 0.38926 | 0.75004 | 0.43346 |
| 1115 | H1 | H | 0.06655 | 0.63926 | 0.49996 |
| 1116 | H1 | H | 0.06655 | 0.88926 | 0.24997 |
| 1117 | H1 | H | 0.31655 | 0.63926 | 0.24996 |
| 1118 | H1 | H | 0.31654 | 0.88926 | 0.49997 |
| 1119 | H1 | H | 0.06655 | 0.86074 | 0.00003 |
| 1120 | H1 | H | 0.06655 | 0.61074 | 0.25004 |
| 1121 | H1 | H | 0.31655 | 0.86074 | 0.25003 |
| 1122 | H1 | H | 0.31655 | 0.61074 | 0.00004 |
| 1123 | H1 | H | 0.43345 | 0.86074 | 0.49996 |
| 1124 | H1 | H | 0.43345 | 0.61074 | 0.24997 |
| 1125 | H1 | H | 0.18345 | 0.86074 | 0.24996 |
| 1126 | H1 | H | 0.18345 | 0.61074 | 0.49997 |
| 1127 | H1 | H | 0.43345 | 0.63926 | 0.00003 |
| 1128 | H1 | H | 0.43345 | 0.88926 | 0.25004 |
| 1129 | H1 | H | 0.18345 | 0.63926 | 0.25003 |
| 1130 | H1 | H | 0.18345 | 0.88926 | 0.00004 |
| 1131 | H1 | H | 0.49997 | 0.56655 | 0.13926 |
| 1132 | H1 | H | 0.49996 | 0.81655 | 0.38926 |
| 1133 | H1 | H | 0.24997 | 0.56655 | 0.38926 |
| 1134 | H1 | H | 0.24996 | 0.81655 | 0.13926 |

| | | | | | |
|---|---|---|---|---|---|
| 1135 | H1 | H | 0.00003 | 0.56655 | 0.36074 |
| 1136 | H1 | H | 0.00003 | 0.81655 | 0.11074 |
| 1137 | H1 | H | 0.25003 | 0.56655 | 0.11074 |
| 1138 | H1 | H | 0.25004 | 0.81655 | 0.36074 |
| 1139 | H1 | H | 0.49997 | 0.93345 | 0.36074 |
| 1140 | H1 | H | 0.49997 | 0.68345 | 0.11074 |
| 1141 | H1 | H | 0.24997 | 0.93345 | 0.11074 |
| 1142 | H1 | H | 0.24997 | 0.68345 | 0.36074 |
| 1143 | H1 | H | 0.00003 | 0.93345 | 0.13926 |
| 1144 | H1 | H | 0.00003 | 0.68345 | 0.38926 |
| 1145 | H1 | H | 0.25004 | 0.93345 | 0.38926 |
| 1146 | H1 | H | 0.25003 | 0.68346 | 0.13926 |
| 1147 | H1 | H | 0.00003 | 0.63926 | 0.43345 |
| 1148 | H1 | H | 0.00003 | 0.88926 | 0.18345 |
| 1149 | H1 | H | 0.25003 | 0.63926 | 0.18345 |
| 1150 | H1 | H | 0.25004 | 0.88926 | 0.43345 |
| 1151 | H1 | H | 0.49996 | 0.86074 | 0.43345 |
| 1152 | H1 | H | 0.49997 | 0.61074 | 0.18345 |
| 1153 | H1 | H | 0.24997 | 0.86074 | 0.18345 |
| 1154 | H1 | H | 0.24997 | 0.61074 | 0.43345 |
| 1155 | H1 | H | 0.00003 | 0.86074 | 0.06655 |
| 1156 | H1 | H | 0.00003 | 0.61074 | 0.31655 |
| 1157 | H1 | H | 0.25004 | 0.86074 | 0.31655 |
| 1158 | H1 | H | 0.25003 | 0.61074 | 0.06654 |
| 1159 | H1 | H | 0.49997 | 0.63926 | 0.06655 |
| 1160 | H1 | H | 0.49997 | 0.88926 | 0.31655 |
| 1161 | H1 | H | 0.24997 | 0.63926 | 0.31655 |
| 1162 | H1 | H | 0.24997 | 0.88926 | 0.06654 |
| 1163 | H1 | H | 0.13926 | 0.56655 | 0.49997 |
| 1164 | H1 | H | 0.13926 | 0.81655 | 0.24996 |
| 1165 | H1 | H | 0.38926 | 0.56655 | 0.24997 |
| 1166 | H1 | H | 0.38926 | 0.81655 | 0.49996 |
| 1167 | H1 | H | 0.36074 | 0.56655 | 0.00003 |
| 1168 | H1 | H | 0.36074 | 0.81655 | 0.25003 |
| 1169 | H1 | H | 0.11074 | 0.56655 | 0.25003 |
| 1170 | H1 | H | 0.11074 | 0.81655 | 0.00003 |
| 1171 | H1 | H | 0.36074 | 0.93345 | 0.49997 |
| 1172 | H1 | H | 0.36074 | 0.68345 | 0.24997 |
| 1173 | H1 | H | 0.11074 | 0.93345 | 0.24997 |
| 1174 | H1 | H | 0.11074 | 0.68345 | 0.49996 |
| 1175 | H1 | H | 0.13926 | 0.93345 | 0.00004 |
| 1176 | H1 | H | 0.13926 | 0.68346 | 0.25003 |
| 1177 | H1 | H | 0.38926 | 0.93345 | 0.25004 |
| 1178 | H1 | H | 0.38926 | 0.68346 | 0.00003 |
| 1179 | H1 | H | 0.06655 | 0.50003 | 0.36074 |
| 1180 | H1 | H | 0.06655 | 0.75004 | 0.11074 |
| 1181 | H1 | H | 0.31655 | 0.50003 | 0.11074 |
| 1182 | H1 | H | 0.31655 | 0.75004 | 0.36074 |
| 1183 | H1 | H | 0.06655 | 0.99996 | 0.13926 |
| 1184 | H1 | H | 0.06655 | 0.74997 | 0.38926 |
| 1185 | H1 | H | 0.31655 | 0.99996 | 0.38926 |
| 1186 | H1 | H | 0.31655 | 0.74997 | 0.13926 |
| 1187 | H1 | H | 0.43345 | 0.50003 | 0.13926 |
| 1188 | H1 | H | 0.43345 | 0.75004 | 0.38926 |

| | | | | | |
|---|---|---|---|---|---|
| 1189 | H1 | H | 0.18345 | 0.50003 | 0.38926 |
| 1190 | H1 | H | 0.18345 | 0.75003 | 0.13926 |
| 1191 | H1 | H | 0.43345 | 0.99996 | 0.36074 |
| 1192 | H1 | H | 0.43345 | 0.74997 | 0.11074 |
| 1193 | H1 | H | 0.18345 | 0.99996 | 0.11074 |
| 1194 | H1 | H | 0.18345 | 0.74997 | 0.36074 |
| 1195 | H2 | H | 0.20825 | 0.79176 | 0.45824 |
| 1196 | H2 | H | 0.29174 | 0.70825 | 0.45823 |
| 1197 | H2 | H | 0.20827 | 0.70823 | 0.04177 |
| 1198 | H2 | H | 0.29179 | 0.79168 | 0.04178 |
| 1199 | H2 | H | 0.45823 | 0.79173 | 0.20823 |
| 1200 | H2 | H | 0.45824 | 0.70825 | 0.29176 |
| 1201 | H2 | H | 0.04174 | 0.79176 | 0.29177 |
| 1202 | H2 | H | 0.04173 | 0.70823 | 0.20823 |
| 1203 | H2 | H | 0.29175 | 0.95824 | 0.20824 |
| 1204 | H2 | H | 0.20824 | 0.95824 | 0.29176 |
| 1205 | H2 | H | 0.20823 | 0.54172 | 0.20822 |
| 1206 | H2 | H | 0.29177 | 0.54173 | 0.29178 |
| 1207 | H2 | H | 0.04178 | 0.54177 | 0.04173 |
| 1208 | H2 | H | 0.45823 | 0.95824 | 0.04174 |
| 1209 | H2 | H | 0.45823 | 0.54176 | 0.45825 |
| 1210 | H2 | H | 0.04176 | 0.95824 | 0.45824 |
| 1211 | O1 | O | 0.08524 | 0.50000 | 0.04765 |
| 1212 | O1 | O | 0.08524 | 0.75000 | 0.29765 |
| 1213 | O1 | O | 0.33524 | 0.50000 | 0.29765 |
| 1214 | O1 | O | 0.33524 | 0.75000 | 0.04765 |
| 1215 | O1 | O | 0.41476 | 0.50000 | 0.04765 |
| 1216 | O1 | O | 0.41476 | 0.75000 | 0.29765 |
| 1217 | O1 | O | 0.16476 | 0.50000 | 0.29765 |
| 1218 | O1 | O | 0.16476 | 0.75000 | 0.04765 |
| 1219 | O1 | O | 0.41476 | 0.50000 | 0.45235 |
| 1220 | O1 | O | 0.41476 | 0.75000 | 0.20235 |
| 1221 | O1 | O | 0.16476 | 0.50000 | 0.20235 |
| 1222 | O1 | O | 0.16476 | 0.75000 | 0.45235 |
| 1223 | O1 | O | 0.08524 | 0.50000 | 0.45235 |
| 1224 | O1 | O | 0.08524 | 0.75000 | 0.20235 |
| 1225 | O1 | O | 0.33524 | 0.50000 | 0.20235 |
| 1226 | O1 | O | 0.33524 | 0.75000 | 0.45235 |
| 1227 | O1 | O | 0.04765 | 0.58524 | 0.00000 |
| 1228 | O1 | O | 0.04765 | 0.83524 | 0.25000 |
| 1229 | O1 | O | 0.29765 | 0.58524 | 0.25000 |
| 1230 | O1 | O | 0.29765 | 0.83524 | 0.00000 |
| 1231 | O1 | O | 0.04765 | 0.91476 | 0.00000 |
| 1232 | O1 | O | 0.04765 | 0.66476 | 0.25000 |
| 1233 | O1 | O | 0.29765 | 0.91476 | 0.25000 |
| 1234 | O1 | O | 0.29765 | 0.66476 | 0.00000 |
| 1235 | O1 | O | 0.45235 | 0.91476 | 0.00000 |
| 1236 | O1 | O | 0.45235 | 0.66476 | 0.25000 |
| 1237 | O1 | O | 0.20235 | 0.91476 | 0.25000 |
| 1238 | O1 | O | 0.20235 | 0.66476 | 0.00000 |
| 1239 | O1 | O | 0.45235 | 0.58524 | 0.00000 |
| 1240 | O1 | O | 0.45235 | 0.83524 | 0.25000 |
| 1241 | O1 | O | 0.20235 | 0.58524 | 0.25000 |
| 1242 | O1 | O | 0.20235 | 0.83524 | 0.00000 |

| | | | | | |
|---|---|---|---|---|---|
| 1243 | O1 | O | 0.00000 | 0.54765 | 0.08524 |
| 1244 | O1 | O | 0.00000 | 0.79765 | 0.33524 |
| 1245 | O1 | O | 0.25000 | 0.54765 | 0.33524 |
| 1246 | O1 | O | 0.25000 | 0.79765 | 0.08524 |
| 1247 | O1 | O | 0.00000 | 0.54765 | 0.41476 |
| 1248 | O1 | O | 0.00000 | 0.79765 | 0.16476 |
| 1249 | O1 | O | 0.25000 | 0.54765 | 0.16476 |
| 1250 | O1 | O | 0.25000 | 0.79765 | 0.41476 |
| 1251 | O1 | O | 0.00000 | 0.95235 | 0.41476 |
| 1252 | O1 | O | 0.00000 | 0.70235 | 0.16476 |
| 1253 | O1 | O | 0.25000 | 0.95235 | 0.16476 |
| 1254 | O1 | O | 0.25000 | 0.70235 | 0.41476 |
| 1255 | O1 | O | 0.00000 | 0.95235 | 0.08524 |
| 1256 | O1 | O | 0.00000 | 0.70235 | 0.33524 |
| 1257 | O1 | O | 0.25000 | 0.95235 | 0.33524 |
| 1258 | O1 | O | 0.25000 | 0.70235 | 0.08524 |
| 1259 | O1 | O | 0.00000 | 0.58524 | 0.45235 |
| 1260 | O1 | O | 0.00000 | 0.83524 | 0.20235 |
| 1261 | O1 | O | 0.25000 | 0.58524 | 0.20235 |
| 1262 | O1 | O | 0.25000 | 0.83524 | 0.45235 |
| 1263 | O1 | O | 0.00000 | 0.91476 | 0.45235 |
| 1264 | O1 | O | 0.00000 | 0.66476 | 0.20235 |
| 1265 | O1 | O | 0.25000 | 0.91476 | 0.20235 |
| 1266 | O1 | O | 0.25000 | 0.66476 | 0.45235 |
| 1267 | O1 | O | 0.00000 | 0.91476 | 0.04765 |
| 1268 | O1 | O | 0.00000 | 0.66476 | 0.29765 |
| 1269 | O1 | O | 0.25000 | 0.91476 | 0.29765 |
| 1270 | O1 | O | 0.25000 | 0.66476 | 0.04765 |
| 1271 | O1 | O | 0.00000 | 0.58524 | 0.04765 |
| 1272 | O1 | O | 0.00000 | 0.83524 | 0.29765 |
| 1273 | O1 | O | 0.25000 | 0.58524 | 0.29765 |
| 1274 | O1 | O | 0.25000 | 0.83524 | 0.04765 |
| 1275 | O1 | O | 0.08524 | 0.54765 | 0.00000 |
| 1276 | O1 | O | 0.08524 | 0.79765 | 0.25000 |
| 1277 | O1 | O | 0.33524 | 0.54765 | 0.25000 |
| 1278 | O1 | O | 0.33524 | 0.79765 | 0.00000 |
| 1279 | O1 | O | 0.41476 | 0.54765 | 0.00000 |
| 1280 | O1 | O | 0.41476 | 0.79765 | 0.25000 |
| 1281 | O1 | O | 0.16476 | 0.54765 | 0.25000 |
| 1282 | O1 | O | 0.16476 | 0.79765 | 0.00000 |
| 1283 | O1 | O | 0.41476 | 0.95235 | 0.00000 |
| 1284 | O1 | O | 0.41476 | 0.70235 | 0.25000 |
| 1285 | O1 | O | 0.16476 | 0.95235 | 0.25000 |
| 1286 | O1 | O | 0.16476 | 0.70235 | 0.00000 |
| 1287 | O1 | O | 0.08524 | 0.95235 | 0.00000 |
| 1288 | O1 | O | 0.08524 | 0.70235 | 0.25000 |
| 1289 | O1 | O | 0.33524 | 0.95235 | 0.25000 |
| 1290 | O1 | O | 0.33524 | 0.70235 | 0.00000 |
| 1291 | O1 | O | 0.04765 | 0.50000 | 0.41476 |
| 1292 | O1 | O | 0.04765 | 0.75000 | 0.16476 |
| 1293 | O1 | O | 0.29765 | 0.50000 | 0.16476 |
| 1294 | O1 | O | 0.29765 | 0.75000 | 0.41476 |
| 1295 | O1 | O | 0.04765 | 0.50000 | 0.08524 |
| 1296 | O1 | O | 0.04765 | 0.75000 | 0.33524 |

| | | | | | |
|---|---|---|---|---|---|
| 1297 | O1 | O | 0.29765 | 0.50000 | 0.33524 |
| 1298 | O1 | O | 0.29765 | 0.75000 | 0.08524 |
| 1299 | O1 | O | 0.45235 | 0.50000 | 0.08524 |
| 1300 | O1 | O | 0.45235 | 0.75000 | 0.33524 |
| 1301 | O1 | O | 0.20235 | 0.50000 | 0.33524 |
| 1302 | O1 | O | 0.20235 | 0.75000 | 0.08524 |
| 1303 | O1 | O | 0.45235 | 0.50000 | 0.41476 |
| 1304 | O1 | O | 0.45235 | 0.75000 | 0.16476 |
| 1305 | O1 | O | 0.20235 | 0.50000 | 0.16476 |
| 1306 | O1 | O | 0.20235 | 0.75000 | 0.41476 |
| 1307 | O2 | O | 0.02805 | 0.97195 | 0.47195 |
| 1308 | O2 | O | 0.02805 | 0.72195 | 0.22195 |
| 1309 | O2 | O | 0.27805 | 0.97195 | 0.22195 |
| 1310 | O2 | O | 0.27805 | 0.72195 | 0.47195 |
| 1311 | O2 | O | 0.47195 | 0.52805 | 0.47195 |
| 1312 | O2 | O | 0.47195 | 0.77805 | 0.22195 |
| 1313 | O2 | O | 0.22195 | 0.52805 | 0.22195 |
| 1314 | O2 | O | 0.22195 | 0.77805 | 0.47195 |
| 1315 | O2 | O | 0.47195 | 0.97195 | 0.02805 |
| 1316 | O2 | O | 0.47195 | 0.72195 | 0.27805 |
| 1317 | O2 | O | 0.22195 | 0.97195 | 0.27805 |
| 1318 | O2 | O | 0.22195 | 0.72195 | 0.02805 |
| 1319 | O2 | O | 0.02805 | 0.52805 | 0.02805 |
| 1320 | O2 | O | 0.02805 | 0.77805 | 0.27805 |
| 1321 | O2 | O | 0.27805 | 0.52805 | 0.27805 |
| 1322 | O2 | O | 0.27805 | 0.77805 | 0.02805 |
| 1323 | O1 | O | 0.47195 | 0.52805 | 0.02805 |
| 1324 | O1 | O | 0.47195 | 0.77805 | 0.27805 |
| 1325 | O1 | O | 0.22195 | 0.52805 | 0.27805 |
| 1326 | O1 | O | 0.22195 | 0.77805 | 0.02805 |
| 1327 | O1 | O | 0.02805 | 0.97195 | 0.02805 |
| 1328 | O1 | O | 0.02805 | 0.72195 | 0.27805 |
| 1329 | O1 | O | 0.27805 | 0.97195 | 0.27805 |
| 1330 | O1 | O | 0.27805 | 0.72195 | 0.02805 |
| 1331 | O1 | O | 0.47195 | 0.97195 | 0.47195 |
| 1332 | O1 | O | 0.47195 | 0.72195 | 0.22195 |
| 1333 | O1 | O | 0.22195 | 0.97195 | 0.22195 |
| 1334 | O1 | O | 0.22195 | 0.72195 | 0.47195 |
| 1335 | O1 | O | 0.02805 | 0.52805 | 0.47195 |
| 1336 | O1 | O | 0.02805 | 0.77805 | 0.22195 |
| 1337 | O1 | O | 0.27805 | 0.52805 | 0.22195 |
| 1338 | O1 | O | 0.27805 | 0.77805 | 0.47195 |
| 1339 | Zr1 | Zr | 0.05994 | 0.50000 | 0.00000 |
| 1340 | Zr1 | Zr | 0.05994 | 0.75000 | 0.25000 |
| 1341 | Zr1 | Zr | 0.30994 | 0.50000 | 0.25000 |
| 1342 | Zr1 | Zr | 0.30994 | 0.75000 | 0.00000 |
| 1343 | Zr1 | Zr | 0.44006 | 0.50000 | 0.00000 |
| 1344 | Zr1 | Zr | 0.44006 | 0.75000 | 0.25000 |
| 1345 | Zr1 | Zr | 0.19006 | 0.50000 | 0.25000 |
| 1346 | Zr1 | Zr | 0.19006 | 0.75000 | 0.00000 |
| 1347 | Zr1 | Zr | 0.00000 | 0.55994 | 0.00000 |
| 1348 | Zr1 | Zr | 0.00000 | 0.80994 | 0.25000 |
| 1349 | Zr1 | Zr | 0.25000 | 0.55994 | 0.25000 |
| 1350 | Zr1 | Zr | 0.25000 | 0.80994 | 0.00000 |

| | | | | | |
|---|---|---|---|---|---|
| 1351 | Zr1 | Zr | 0.00000 | 0.94006 | 0.00000 |
| 1352 | Zr1 | Zr | 0.00000 | 0.69006 | 0.25000 |
| 1353 | Zr1 | Zr | 0.25000 | 0.94006 | 0.25000 |
| 1354 | Zr1 | Zr | 0.25000 | 0.69006 | 0.00000 |
| 1355 | Zr1 | Zr | 0.00000 | 0.50000 | 0.05994 |
| 1356 | Zr1 | Zr | 0.00000 | 0.75000 | 0.30994 |
| 1357 | Zr1 | Zr | 0.25000 | 0.50000 | 0.30994 |
| 1358 | Zr1 | Zr | 0.25000 | 0.75000 | 0.05994 |
| 1359 | Zr1 | Zr | 0.00000 | 0.50000 | 0.44006 |
| 1360 | Zr1 | Zr | 0.00000 | 0.75000 | 0.19006 |
| 1361 | Zr1 | Zr | 0.25000 | 0.50000 | 0.19006 |
| 1362 | Zr1 | Zr | 0.25000 | 0.75000 | 0.44006 |
| 1363 | C2 | C | 0.13375 | 0.50000 | 0.59220 |
| 1364 | C2 | C | 0.13375 | 0.75000 | 0.84220 |
| 1365 | C2 | C | 0.38375 | 0.50000 | 0.84220 |
| 1366 | C2 | C | 0.38375 | 0.75000 | 0.59220 |
| 1367 | C2 | C | 0.36625 | 0.50000 | 0.59220 |
| 1368 | C2 | C | 0.36625 | 0.75000 | 0.84220 |
| 1369 | C2 | C | 0.11625 | 0.50000 | 0.84220 |
| 1370 | C2 | C | 0.11625 | 0.75000 | 0.59220 |
| 1371 | C2 | C | 0.36625 | 0.50000 | 0.90780 |
| 1372 | C2 | C | 0.36625 | 0.75000 | 0.65780 |
| 1373 | C2 | C | 0.11625 | 0.50000 | 0.65780 |
| 1374 | C2 | C | 0.11625 | 0.75000 | 0.90780 |
| 1375 | C2 | C | 0.13375 | 0.50000 | 0.90780 |
| 1376 | C2 | C | 0.13375 | 0.75000 | 0.65780 |
| 1377 | C2 | C | 0.38375 | 0.50000 | 0.65780 |
| 1378 | C2 | C | 0.38375 | 0.75000 | 0.90780 |
| 1379 | C2 | C | 0.09220 | 0.63375 | 0.50000 |
| 1380 | C2 | C | 0.34220 | 0.63375 | 0.75000 |
| 1381 | C2 | C | 0.34220 | 0.88375 | 0.50000 |
| 1382 | C2 | C | 0.09220 | 0.86625 | 0.50000 |
| 1383 | C2 | C | 0.09220 | 0.61625 | 0.75000 |
| 1384 | C2 | C | 0.34220 | 0.86625 | 0.75000 |
| 1385 | C2 | C | 0.40780 | 0.86625 | 0.50000 |
| 1386 | C2 | C | 0.40780 | 0.61625 | 0.75000 |
| 1387 | C2 | C | 0.15780 | 0.61625 | 0.50000 |
| 1388 | C2 | C | 0.40780 | 0.88375 | 0.75000 |
| 1389 | C2 | C | 0.15780 | 0.63375 | 0.75000 |
| 1390 | C2 | C | 0.15780 | 0.88375 | 0.50000 |
| 1391 | C2 | C | 0.00000 | 0.59220 | 0.63375 |
| 1392 | C2 | C | 0.00000 | 0.84220 | 0.88375 |
| 1393 | C2 | C | 0.25000 | 0.59220 | 0.88375 |
| 1394 | C2 | C | 0.25000 | 0.84220 | 0.63375 |
| 1395 | C2 | C | 0.00000 | 0.59220 | 0.86625 |
| 1396 | C2 | C | 0.00000 | 0.84220 | 0.61625 |
| 1397 | C2 | C | 0.25000 | 0.59220 | 0.61625 |
| 1398 | C2 | C | 0.25000 | 0.84220 | 0.86625 |
| 1399 | C2 | C | 0.00000 | 0.90780 | 0.86625 |
| 1400 | C2 | C | 0.00000 | 0.65780 | 0.61625 |
| 1401 | C2 | C | 0.25000 | 0.90780 | 0.61625 |
| 1402 | C2 | C | 0.25000 | 0.65780 | 0.86625 |
| 1403 | C2 | C | 0.00000 | 0.90780 | 0.63375 |
| 1404 | C2 | C | 0.00000 | 0.65780 | 0.88375 |

| | | | | | |
|---|---|---|---|---|---|
| 1405 | C2 | C | 0.25000 | 0.90780 | 0.88375 |
| 1406 | C2 | C | 0.25000 | 0.65780 | 0.63375 |
| 1407 | C2 | C | 0.00000 | 0.63375 | 0.90780 |
| 1408 | C2 | C | 0.00000 | 0.88375 | 0.65780 |
| 1409 | C2 | C | 0.25000 | 0.63375 | 0.65780 |
| 1410 | C2 | C | 0.25000 | 0.88375 | 0.90780 |
| 1411 | C2 | C | 0.00000 | 0.86625 | 0.90780 |
| 1412 | C2 | C | 0.00000 | 0.61625 | 0.65780 |
| 1413 | C2 | C | 0.25000 | 0.86625 | 0.65780 |
| 1414 | C2 | C | 0.25000 | 0.61625 | 0.90780 |
| 1415 | C2 | C | 0.00000 | 0.86625 | 0.59220 |
| 1416 | C2 | C | 0.00000 | 0.61625 | 0.84220 |
| 1417 | C2 | C | 0.25000 | 0.86625 | 0.84220 |
| 1418 | C2 | C | 0.25000 | 0.61625 | 0.59220 |
| 1419 | C2 | C | 0.00000 | 0.63375 | 0.59220 |
| 1420 | C2 | C | 0.00000 | 0.88375 | 0.84220 |
| 1421 | C2 | C | 0.25000 | 0.63375 | 0.84220 |
| 1422 | C2 | C | 0.25000 | 0.88375 | 0.59220 |
| 1423 | C2 | C | 0.13375 | 0.59220 | 0.50000 |
| 1424 | C2 | C | 0.38375 | 0.59220 | 0.75000 |
| 1425 | C2 | C | 0.38375 | 0.84220 | 0.50000 |
| 1426 | C2 | C | 0.36625 | 0.84220 | 0.75000 |
| 1427 | C2 | C | 0.11625 | 0.59220 | 0.75000 |
| 1428 | C2 | C | 0.11625 | 0.84220 | 0.50000 |
| 1429 | C2 | C | 0.36625 | 0.90780 | 0.50000 |
| 1430 | C2 | C | 0.36625 | 0.65780 | 0.75000 |
| 1431 | C2 | C | 0.11625 | 0.65780 | 0.50000 |
| 1432 | C2 | C | 0.13375 | 0.90780 | 0.50000 |
| 1433 | C2 | C | 0.13375 | 0.65780 | 0.75000 |
| 1434 | C2 | C | 0.38375 | 0.90780 | 0.75000 |
| 1435 | C2 | C | 0.09220 | 0.50000 | 0.86625 |
| 1436 | C2 | C | 0.09220 | 0.75000 | 0.61625 |
| 1437 | C2 | C | 0.34220 | 0.50000 | 0.61625 |
| 1438 | C2 | C | 0.34220 | 0.75000 | 0.86625 |
| 1439 | C2 | C | 0.09220 | 0.50000 | 0.63375 |
| 1440 | C2 | C | 0.09220 | 0.75000 | 0.88375 |
| 1441 | C2 | C | 0.34220 | 0.50000 | 0.88375 |
| 1442 | C2 | C | 0.34220 | 0.75000 | 0.63375 |
| 1443 | C2 | C | 0.40780 | 0.50000 | 0.63375 |
| 1444 | C2 | C | 0.40780 | 0.75000 | 0.88375 |
| 1445 | C2 | C | 0.15780 | 0.50000 | 0.88375 |
| 1446 | C2 | C | 0.15780 | 0.75000 | 0.63375 |
| 1447 | C2 | C | 0.40780 | 0.50000 | 0.86625 |
| 1448 | C2 | C | 0.40780 | 0.75000 | 0.61625 |
| 1449 | C2 | C | 0.15780 | 0.50000 | 0.61625 |
| 1450 | C2 | C | 0.15780 | 0.75000 | 0.86625 |
| 1451 | C1 | C | 0.07680 | 0.50000 | 0.92320 |
| 1452 | C1 | C | 0.07680 | 0.75000 | 0.67320 |
| 1453 | C1 | C | 0.32680 | 0.50000 | 0.67320 |
| 1454 | C1 | C | 0.32680 | 0.75000 | 0.92320 |
| 1455 | C1 | C | 0.42320 | 0.50000 | 0.92320 |
| 1456 | C1 | C | 0.42320 | 0.75000 | 0.67320 |
| 1457 | C1 | C | 0.17320 | 0.50000 | 0.67320 |
| 1458 | C1 | C | 0.17320 | 0.75000 | 0.92320 |

| | | | | | |
|---|---|---|---|---|---|
| 1459 | C1 | C | 0.42320 | 0.50000 | 0.57680 |
| 1460 | C1 | C | 0.42320 | 0.75000 | 0.82680 |
| 1461 | C1 | C | 0.17320 | 0.50000 | 0.82680 |
| 1462 | C1 | C | 0.17320 | 0.75000 | 0.57680 |
| 1463 | C1 | C | 0.07680 | 0.50000 | 0.57680 |
| 1464 | C1 | C | 0.07680 | 0.75000 | 0.82680 |
| 1465 | C1 | C | 0.32680 | 0.50000 | 0.82680 |
| 1466 | C1 | C | 0.32680 | 0.75000 | 0.57680 |
| 1467 | C1 | C | 0.42320 | 0.82680 | 0.75000 |
| 1468 | C1 | C | 0.17320 | 0.57680 | 0.75000 |
| 1469 | C1 | C | 0.17320 | 0.82680 | 0.50000 |
| 1470 | C1 | C | 0.42320 | 0.92320 | 0.50000 |
| 1471 | C1 | C | 0.42320 | 0.67320 | 0.75000 |
| 1472 | C1 | C | 0.17320 | 0.67320 | 0.50000 |
| 1473 | C1 | C | 0.07680 | 0.92320 | 0.50000 |
| 1474 | C1 | C | 0.07680 | 0.67320 | 0.75000 |
| 1475 | C1 | C | 0.32680 | 0.92320 | 0.75000 |
| 1476 | C1 | C | 0.07680 | 0.57680 | 0.50000 |
| 1477 | C1 | C | 0.32680 | 0.57680 | 0.75000 |
| 1478 | C1 | C | 0.32680 | 0.82680 | 0.50000 |
| 1479 | C1 | C | 0.00000 | 0.92320 | 0.57680 |
| 1480 | C1 | C | 0.00000 | 0.67320 | 0.82680 |
| 1481 | C1 | C | 0.25000 | 0.92320 | 0.82680 |
| 1482 | C1 | C | 0.25000 | 0.67320 | 0.57680 |
| 1483 | C1 | C | 0.00000 | 0.92320 | 0.92320 |
| 1484 | C1 | C | 0.00000 | 0.67320 | 0.67320 |
| 1485 | C1 | C | 0.25000 | 0.92320 | 0.67320 |
| 1486 | C1 | C | 0.25000 | 0.67320 | 0.92320 |
| 1487 | C1 | C | 0.00000 | 0.57680 | 0.92320 |
| 1488 | C1 | C | 0.00000 | 0.82680 | 0.67320 |
| 1489 | C1 | C | 0.25000 | 0.57680 | 0.67320 |
| 1490 | C1 | C | 0.25000 | 0.82680 | 0.92320 |
| 1491 | C1 | C | 0.00000 | 0.57680 | 0.57680 |
| 1492 | C1 | C | 0.00000 | 0.82680 | 0.82680 |
| 1493 | C1 | C | 0.25000 | 0.57680 | 0.82680 |
| 1494 | C1 | C | 0.25000 | 0.82680 | 0.57680 |
| 1495 | C3 | C | 0.10250 | 0.50000 | 0.60250 |
| 1496 | C3 | C | 0.10250 | 0.75000 | 0.85250 |
| 1497 | C3 | C | 0.35250 | 0.50000 | 0.85250 |
| 1498 | C3 | C | 0.35250 | 0.75000 | 0.60250 |
| 1499 | C3 | C | 0.39750 | 0.50000 | 0.60250 |
| 1500 | C3 | C | 0.39750 | 0.75000 | 0.85250 |
| 1501 | C3 | C | 0.14750 | 0.50000 | 0.85250 |
| 1502 | C3 | C | 0.14750 | 0.75000 | 0.60250 |
| 1503 | C3 | C | 0.39750 | 0.50000 | 0.89750 |
| 1504 | C3 | C | 0.39750 | 0.75000 | 0.64750 |
| 1505 | C3 | C | 0.14750 | 0.50000 | 0.64750 |
| 1506 | C3 | C | 0.14750 | 0.75000 | 0.89750 |
| 1507 | C3 | C | 0.10250 | 0.50000 | 0.89750 |
| 1508 | C3 | C | 0.10250 | 0.75000 | 0.64750 |
| 1509 | C3 | C | 0.35250 | 0.50000 | 0.64750 |
| 1510 | C3 | C | 0.35250 | 0.75000 | 0.89750 |
| 1511 | C3 | C | 0.10250 | 0.60250 | 0.50000 |
| 1512 | C3 | C | 0.35250 | 0.60250 | 0.75000 |

| | | | | | |
|---|---|---|---|---|---|
| 1513 | C3 | C | 0.35250 | 0.85250 | 0.50000 |
| 1514 | C3 | C | 0.10250 | 0.89750 | 0.50000 |
| 1515 | C3 | C | 0.10250 | 0.64750 | 0.75000 |
| 1516 | C3 | C | 0.35250 | 0.89750 | 0.75000 |
| 1517 | C3 | C | 0.39750 | 0.89750 | 0.50000 |
| 1518 | C3 | C | 0.39750 | 0.64750 | 0.75000 |
| 1519 | C3 | C | 0.14750 | 0.64750 | 0.50000 |
| 1520 | C3 | C | 0.39750 | 0.85250 | 0.75000 |
| 1521 | C3 | C | 0.14750 | 0.60250 | 0.75000 |
| 1522 | C3 | C | 0.14750 | 0.85250 | 0.50000 |
| 1523 | C3 | C | 0.00000 | 0.60250 | 0.60250 |
| 1524 | C3 | C | 0.00000 | 0.85250 | 0.85250 |
| 1525 | C3 | C | 0.25000 | 0.60250 | 0.85250 |
| 1526 | C3 | C | 0.25000 | 0.85250 | 0.60250 |
| 1527 | C3 | C | 0.00000 | 0.60250 | 0.89750 |
| 1528 | C3 | C | 0.00000 | 0.85250 | 0.64750 |
| 1529 | C3 | C | 0.25000 | 0.60250 | 0.64750 |
| 1530 | C3 | C | 0.25000 | 0.85250 | 0.89750 |
| 1531 | C3 | C | 0.00000 | 0.89750 | 0.89750 |
| 1532 | C3 | C | 0.00000 | 0.64750 | 0.64750 |
| 1533 | C3 | C | 0.25000 | 0.89750 | 0.64750 |
| 1534 | C3 | C | 0.25000 | 0.64750 | 0.89750 |
| 1535 | C3 | C | 0.00000 | 0.89750 | 0.60250 |
| 1536 | C3 | C | 0.00000 | 0.64750 | 0.85250 |
| 1537 | C3 | C | 0.25000 | 0.89750 | 0.85250 |
| 1538 | C3 | C | 0.25000 | 0.64750 | 0.60250 |
| 1539 | H1 | H | 0.13926 | 0.99997 | 0.56655 |
| 1540 | H1 | H | 0.13926 | 0.74997 | 0.81655 |
| 1541 | H1 | H | 0.38926 | 0.99997 | 0.81655 |
| 1542 | H1 | H | 0.38926 | 0.74997 | 0.56655 |
| 1543 | H1 | H | 0.36074 | 0.50003 | 0.56655 |
| 1544 | H1 | H | 0.36074 | 0.75004 | 0.81655 |
| 1545 | H1 | H | 0.11074 | 0.50003 | 0.81655 |
| 1546 | H1 | H | 0.11074 | 0.75004 | 0.56655 |
| 1547 | H1 | H | 0.36074 | 0.99996 | 0.93345 |
| 1548 | H1 | H | 0.36074 | 0.74997 | 0.68345 |
| 1549 | H1 | H | 0.11074 | 0.99997 | 0.68345 |
| 1550 | H1 | H | 0.11074 | 0.74997 | 0.93346 |
| 1551 | H1 | H | 0.13926 | 0.50003 | 0.93345 |
| 1552 | H1 | H | 0.13926 | 0.75003 | 0.68346 |
| 1553 | H1 | H | 0.38926 | 0.50003 | 0.68345 |
| 1554 | H1 | H | 0.38926 | 0.75004 | 0.93346 |
| 1555 | H1 | H | 0.06655 | 0.63926 | 0.99996 |
| 1556 | H1 | H | 0.31655 | 0.63926 | 0.74996 |
| 1557 | H1 | H | 0.31654 | 0.88926 | 0.99997 |
| 1558 | H1 | H | 0.06655 | 0.86074 | 0.50003 |
| 1559 | H1 | H | 0.06655 | 0.61074 | 0.75004 |
| 1560 | H1 | H | 0.31655 | 0.86074 | 0.75003 |
| 1561 | H1 | H | 0.43345 | 0.86074 | 0.99996 |
| 1562 | H1 | H | 0.43345 | 0.61074 | 0.74997 |
| 1563 | H1 | H | 0.18345 | 0.61074 | 0.99997 |
| 1564 | H1 | H | 0.43345 | 0.88926 | 0.75004 |
| 1565 | H1 | H | 0.18345 | 0.63926 | 0.75003 |
| 1566 | H1 | H | 0.18345 | 0.88926 | 0.50004 |

| | | | | | |
|---|---|---|---|---|---|
| 1567 | H1 | H | 0.49997 | 0.56655 | 0.63926 |
| 1568 | H1 | H | 0.49996 | 0.81655 | 0.88926 |
| 1569 | H1 | H | 0.24997 | 0.56655 | 0.88926 |
| 1570 | H1 | H | 0.24996 | 0.81655 | 0.63926 |
| 1571 | H1 | H | 0.00003 | 0.56655 | 0.86074 |
| 1572 | H1 | H | 0.00003 | 0.81655 | 0.61074 |
| 1573 | H1 | H | 0.25003 | 0.56655 | 0.61074 |
| 1574 | H1 | H | 0.25004 | 0.81655 | 0.86074 |
| 1575 | H1 | H | 0.49997 | 0.93345 | 0.86074 |
| 1576 | H1 | H | 0.49997 | 0.68345 | 0.61074 |
| 1577 | H1 | H | 0.24997 | 0.93345 | 0.61074 |
| 1578 | H1 | H | 0.24997 | 0.68345 | 0.86074 |
| 1579 | H1 | H | 0.00003 | 0.93345 | 0.63926 |
| 1580 | H1 | H | 0.00003 | 0.68345 | 0.88926 |
| 1581 | H1 | H | 0.25004 | 0.93345 | 0.88926 |
| 1582 | H1 | H | 0.25003 | 0.68346 | 0.63926 |
| 1583 | H1 | H | 0.00003 | 0.63926 | 0.93345 |
| 1584 | H1 | H | 0.00003 | 0.88926 | 0.68345 |
| 1585 | H1 | H | 0.25003 | 0.63926 | 0.68345 |
| 1586 | H1 | H | 0.25004 | 0.88926 | 0.93345 |
| 1587 | H1 | H | 0.49996 | 0.86074 | 0.93345 |
| 1588 | H1 | H | 0.49997 | 0.61074 | 0.68345 |
| 1589 | H1 | H | 0.24997 | 0.86074 | 0.68345 |
| 1590 | H1 | H | 0.24997 | 0.61074 | 0.93345 |
| 1591 | H1 | H | 0.00003 | 0.86074 | 0.56655 |
| 1592 | H1 | H | 0.00003 | 0.61074 | 0.81655 |
| 1593 | H1 | H | 0.25004 | 0.86074 | 0.81655 |
| 1594 | H1 | H | 0.25003 | 0.61074 | 0.56654 |
| 1595 | H1 | H | 0.49997 | 0.63926 | 0.56655 |
| 1596 | H1 | H | 0.49997 | 0.88926 | 0.81655 |
| 1597 | H1 | H | 0.24997 | 0.63926 | 0.81655 |
| 1598 | H1 | H | 0.24997 | 0.88926 | 0.56654 |
| 1599 | H1 | H | 0.13926 | 0.56655 | 0.99997 |
| 1600 | H1 | H | 0.38926 | 0.56655 | 0.74997 |
| 1601 | H1 | H | 0.38926 | 0.81655 | 0.99996 |
| 1602 | H1 | H | 0.36074 | 0.81655 | 0.75003 |
| 1603 | H1 | H | 0.11074 | 0.56655 | 0.75003 |
| 1604 | H1 | H | 0.11074 | 0.81655 | 0.50003 |
| 1605 | H1 | H | 0.36074 | 0.93345 | 0.99997 |
| 1606 | H1 | H | 0.36074 | 0.68345 | 0.74997 |
| 1607 | H1 | H | 0.11074 | 0.68345 | 0.99996 |
| 1608 | H1 | H | 0.13926 | 0.93345 | 0.50004 |
| 1609 | H1 | H | 0.13926 | 0.68346 | 0.75003 |
| 1610 | H1 | H | 0.38926 | 0.93345 | 0.75004 |
| 1611 | H1 | H | 0.06655 | 0.50003 | 0.86074 |
| 1612 | H1 | H | 0.06655 | 0.75004 | 0.61074 |
| 1613 | H1 | H | 0.31655 | 0.50003 | 0.61074 |
| 1614 | H1 | H | 0.31655 | 0.75004 | 0.86074 |
| 1615 | H1 | H | 0.06655 | 0.99996 | 0.63926 |
| 1616 | H1 | H | 0.06655 | 0.74997 | 0.88926 |
| 1617 | H1 | H | 0.31655 | 0.99996 | 0.88926 |
| 1618 | H1 | H | 0.31655 | 0.74997 | 0.63926 |
| 1619 | H1 | H | 0.43345 | 0.50003 | 0.63926 |
| 1620 | H1 | H | 0.43345 | 0.75004 | 0.88926 |

| | | | | | |
|---|---|---|---|---|---|
| 1621 | H1 | H | 0.18345 | 0.50003 | 0.88926 |
| 1622 | H1 | H | 0.18345 | 0.75003 | 0.63926 |
| 1623 | H1 | H | 0.43345 | 0.99996 | 0.86074 |
| 1624 | H1 | H | 0.43345 | 0.74997 | 0.61074 |
| 1625 | H1 | H | 0.18345 | 0.99996 | 0.61074 |
| 1626 | H1 | H | 0.18345 | 0.74997 | 0.86074 |
| 1627 | H2 | H | 0.20825 | 0.79176 | 0.95824 |
| 1628 | H2 | H | 0.29174 | 0.70825 | 0.95823 |
| 1629 | H2 | H | 0.20827 | 0.70823 | 0.54177 |
| 1630 | H2 | H | 0.29179 | 0.79168 | 0.54178 |
| 1631 | H2 | H | 0.45823 | 0.79173 | 0.70823 |
| 1632 | H2 | H | 0.45824 | 0.70825 | 0.79176 |
| 1633 | H2 | H | 0.04174 | 0.79176 | 0.79177 |
| 1634 | H2 | H | 0.04173 | 0.70823 | 0.70823 |
| 1635 | H2 | H | 0.29175 | 0.95824 | 0.70824 |
| 1636 | H2 | H | 0.20824 | 0.95824 | 0.79176 |
| 1637 | H2 | H | 0.20823 | 0.54172 | 0.70822 |
| 1638 | H2 | H | 0.29177 | 0.54173 | 0.79178 |
| 1639 | H2 | H | 0.04178 | 0.54177 | 0.54173 |
| 1640 | H2 | H | 0.45823 | 0.95824 | 0.54174 |
| 1641 | H2 | H | 0.45823 | 0.54176 | 0.95825 |
| 1642 | H2 | H | 0.04176 | 0.95824 | 0.95824 |
| 1643 | O1 | O | 0.08524 | 0.50000 | 0.54765 |
| 1644 | O1 | O | 0.08524 | 0.75000 | 0.79765 |
| 1645 | O1 | O | 0.33524 | 0.50000 | 0.79765 |
| 1646 | O1 | O | 0.33524 | 0.75000 | 0.54765 |
| 1647 | O1 | O | 0.41476 | 0.50000 | 0.54765 |
| 1648 | O1 | O | 0.41476 | 0.75000 | 0.79765 |
| 1649 | O1 | O | 0.16476 | 0.50000 | 0.79765 |
| 1650 | O1 | O | 0.16476 | 0.75000 | 0.54765 |
| 1651 | O1 | O | 0.41476 | 0.50000 | 0.95235 |
| 1652 | O1 | O | 0.41476 | 0.75000 | 0.70235 |
| 1653 | O1 | O | 0.16476 | 0.50000 | 0.70235 |
| 1654 | O1 | O | 0.16476 | 0.75000 | 0.95235 |
| 1655 | O1 | O | 0.08524 | 0.50000 | 0.95235 |
| 1656 | O1 | O | 0.08524 | 0.75000 | 0.70235 |
| 1657 | O1 | O | 0.33524 | 0.50000 | 0.70235 |
| 1658 | O1 | O | 0.33524 | 0.75000 | 0.95235 |
| 1659 | O1 | O | 0.04765 | 0.58524 | 0.50000 |
| 1660 | O3 | O | 0.04765 | 0.83524 | 0.75000 |
| 1661 | O1 | O | 0.29765 | 0.58524 | 0.75000 |
| 1662 | O1 | O | 0.29765 | 0.83524 | 0.50000 |
| 1663 | O1 | O | 0.04765 | 0.91476 | 0.50000 |
| 1664 | O1 | O | 0.04765 | 0.66476 | 0.75000 |
| 1665 | O1 | O | 0.29765 | 0.91476 | 0.75000 |
| 1666 | O3 | O | 0.29765 | 0.66476 | 0.50000 |
| 1667 | O1 | O | 0.45235 | 0.91476 | 0.50000 |
| 1668 | O1 | O | 0.45235 | 0.66476 | 0.75000 |
| 1669 | O3 | O | 0.20235 | 0.91476 | 0.75000 |
| 1670 | O1 | O | 0.20235 | 0.66476 | 0.50000 |
| 1671 | O3 | O | 0.45235 | 0.58524 | 0.50000 |
| 1672 | O1 | O | 0.45235 | 0.83524 | 0.75000 |
| 1673 | O1 | O | 0.20235 | 0.58524 | 0.75000 |
| 1674 | O1 | O | 0.20235 | 0.83524 | 0.50000 |

| | | | | | |
|---|---|---|---|---|---|
| 1675 | O1 | O | 0.00000 | 0.54765 | 0.58524 |
| 1676 | O1 | O | 0.00000 | 0.79765 | 0.83524 |
| 1677 | O1 | O | 0.25000 | 0.54765 | 0.83524 |
| 1678 | O1 | O | 0.25000 | 0.79765 | 0.58524 |
| 1679 | O1 | O | 0.00000 | 0.54765 | 0.91476 |
| 1680 | O1 | O | 0.00000 | 0.79765 | 0.66476 |
| 1681 | O1 | O | 0.25000 | 0.54765 | 0.66476 |
| 1682 | O1 | O | 0.25000 | 0.79765 | 0.91476 |
| 1683 | O1 | O | 0.00000 | 0.95235 | 0.91476 |
| 1684 | O1 | O | 0.00000 | 0.70235 | 0.66476 |
| 1685 | O1 | O | 0.25000 | 0.95235 | 0.66476 |
| 1686 | O1 | O | 0.25000 | 0.70235 | 0.91476 |
| 1687 | O1 | O | 0.00000 | 0.95235 | 0.58524 |
| 1688 | O1 | O | 0.00000 | 0.70235 | 0.83524 |
| 1689 | O1 | O | 0.25000 | 0.95235 | 0.83524 |
| 1690 | O1 | O | 0.25000 | 0.70235 | 0.58524 |
| 1691 | O1 | O | 0.00000 | 0.58524 | 0.95235 |
| 1692 | O1 | O | 0.00000 | 0.83524 | 0.70235 |
| 1693 | O1 | O | 0.25000 | 0.58524 | 0.70235 |
| 1694 | O1 | O | 0.25000 | 0.83524 | 0.95235 |
| 1695 | O1 | O | 0.00000 | 0.91476 | 0.95235 |
| 1696 | O1 | O | 0.00000 | 0.66476 | 0.70235 |
| 1697 | O1 | O | 0.25000 | 0.91476 | 0.70235 |
| 1698 | O1 | O | 0.25000 | 0.66476 | 0.95235 |
| 1699 | O1 | O | 0.00000 | 0.91476 | 0.54765 |
| 1700 | O1 | O | 0.00000 | 0.66476 | 0.79765 |
| 1701 | O1 | O | 0.25000 | 0.91476 | 0.79765 |
| 1702 | O1 | O | 0.25000 | 0.66476 | 0.54765 |
| 1703 | O1 | O | 0.00000 | 0.58524 | 0.54765 |
| 1704 | O1 | O | 0.00000 | 0.83524 | 0.79765 |
| 1705 | O1 | O | 0.25000 | 0.58524 | 0.79765 |
| 1706 | O1 | O | 0.25000 | 0.83524 | 0.54765 |
| 1707 | O1 | O | 0.08524 | 0.54765 | 0.50000 |
| 1708 | O3 | O | 0.08524 | 0.79765 | 0.75000 |
| 1709 | O1 | O | 0.33524 | 0.54765 | 0.75000 |
| 1710 | O1 | O | 0.33524 | 0.79765 | 0.50000 |
| 1711 | O3 | O | 0.41476 | 0.54765 | 0.50000 |
| 1712 | O1 | O | 0.41476 | 0.79765 | 0.75000 |
| 1713 | O1 | O | 0.16476 | 0.54765 | 0.75000 |
| 1714 | O1 | O | 0.16476 | 0.79765 | 0.50000 |
| 1715 | O1 | O | 0.41476 | 0.95235 | 0.50000 |
| 1716 | O1 | O | 0.41476 | 0.70235 | 0.75000 |
| 1717 | O3 | O | 0.16476 | 0.95235 | 0.75000 |
| 1718 | O1 | O | 0.16476 | 0.70235 | 0.50000 |
| 1719 | O1 | O | 0.08524 | 0.95235 | 0.50000 |
| 1720 | O1 | O | 0.08524 | 0.70235 | 0.75000 |
| 1721 | O1 | O | 0.33524 | 0.95235 | 0.75000 |
| 1722 | O3 | O | 0.33524 | 0.70235 | 0.50000 |
| 1723 | O1 | O | 0.04765 | 0.50000 | 0.91476 |
| 1724 | O1 | O | 0.04765 | 0.75000 | 0.66476 |
| 1725 | O1 | O | 0.29765 | 0.50000 | 0.66476 |
| 1726 | O1 | O | 0.29765 | 0.75000 | 0.91476 |
| 1727 | O1 | O | 0.04765 | 0.50000 | 0.58524 |
| 1728 | O1 | O | 0.04765 | 0.75000 | 0.83524 |

| | | | | | |
|---|---|---|---|---|---|
| 1729 | O1 | O | 0.29765 | 0.50000 | 0.83524 |
| 1730 | O1 | O | 0.29765 | 0.75000 | 0.58524 |
| 1731 | O1 | O | 0.45235 | 0.50000 | 0.58524 |
| 1732 | O1 | O | 0.45235 | 0.75000 | 0.83524 |
| 1733 | O1 | O | 0.20235 | 0.50000 | 0.83524 |
| 1734 | O1 | O | 0.20235 | 0.75000 | 0.58524 |
| 1735 | O1 | O | 0.45235 | 0.50000 | 0.91476 |
| 1736 | O1 | O | 0.45235 | 0.75000 | 0.66476 |
| 1737 | O1 | O | 0.20235 | 0.50000 | 0.66476 |
| 1738 | O1 | O | 0.20235 | 0.75000 | 0.91476 |
| 1739 | O2 | O | 0.02805 | 0.97195 | 0.97195 |
| 1740 | O2 | O | 0.02805 | 0.72195 | 0.72195 |
| 1741 | O2 | O | 0.27805 | 0.97195 | 0.72195 |
| 1742 | O2 | O | 0.27805 | 0.72195 | 0.97195 |
| 1743 | O2 | O | 0.47195 | 0.52805 | 0.97195 |
| 1744 | O2 | O | 0.47195 | 0.77805 | 0.72195 |
| 1745 | O2 | O | 0.22195 | 0.52805 | 0.72195 |
| 1746 | O2 | O | 0.22195 | 0.77805 | 0.97195 |
| 1747 | O2 | O | 0.47195 | 0.97195 | 0.52805 |
| 1748 | O2 | O | 0.47195 | 0.72195 | 0.77805 |
| 1749 | O2 | O | 0.22195 | 0.97195 | 0.77805 |
| 1750 | O2 | O | 0.22195 | 0.72195 | 0.52805 |
| 1751 | O2 | O | 0.02805 | 0.52805 | 0.52805 |
| 1752 | O2 | O | 0.02805 | 0.77805 | 0.77805 |
| 1753 | O2 | O | 0.27805 | 0.52805 | 0.77805 |
| 1754 | O2 | O | 0.27805 | 0.77805 | 0.52805 |
| 1755 | O1 | O | 0.47195 | 0.52805 | 0.52805 |
| 1756 | O1 | O | 0.47195 | 0.77805 | 0.77805 |
| 1757 | O1 | O | 0.22195 | 0.52805 | 0.77805 |
| 1758 | O1 | O | 0.22195 | 0.77805 | 0.52805 |
| 1759 | O1 | O | 0.02805 | 0.97195 | 0.52805 |
| 1760 | O1 | O | 0.02805 | 0.72195 | 0.77805 |
| 1761 | O1 | O | 0.27805 | 0.97195 | 0.77805 |
| 1762 | O1 | O | 0.27805 | 0.72195 | 0.52805 |
| 1763 | O1 | O | 0.47195 | 0.97195 | 0.97195 |
| 1764 | O1 | O | 0.47195 | 0.72195 | 0.72195 |
| 1765 | O1 | O | 0.22195 | 0.97195 | 0.72195 |
| 1766 | O1 | O | 0.22195 | 0.72195 | 0.97195 |
| 1767 | O1 | O | 0.02805 | 0.52805 | 0.97195 |
| 1768 | O1 | O | 0.02805 | 0.77805 | 0.72195 |
| 1769 | O1 | O | 0.27805 | 0.52805 | 0.72195 |
| 1770 | O1 | O | 0.27805 | 0.77805 | 0.97195 |
| 1771 | Zr1 | Zr | 0.05994 | 0.50000 | 0.50000 |
| 1772 | Zr1 | Zr | 0.05994 | 0.75000 | 0.75000 |
| 1773 | Zr1 | Zr | 0.30994 | 0.50000 | 0.75000 |
| 1774 | Zr1 | Zr | 0.30994 | 0.75000 | 0.50000 |
| 1775 | Zr1 | Zr | 0.44006 | 0.50000 | 0.50000 |
| 1776 | Zr1 | Zr | 0.44006 | 0.75000 | 0.75000 |
| 1777 | Zr1 | Zr | 0.19006 | 0.50000 | 0.75000 |
| 1778 | Zr1 | Zr | 0.19006 | 0.75000 | 0.50000 |
| 1779 | Zr1 | Zr | 0.00000 | 0.55994 | 0.50000 |
| 1780 | Zr1 | Zr | 0.00000 | 0.80994 | 0.75000 |
| 1781 | Zr1 | Zr | 0.25000 | 0.55994 | 0.75000 |
| 1782 | Zr1 | Zr | 0.25000 | 0.80994 | 0.50000 |

| | | | | | |
|---|---|---|---|---|---|
| 1783 | Zr1 | Zr | 0.00000 | 0.94006 | 0.50000 |
| 1784 | Zr1 | Zr | 0.00000 | 0.69006 | 0.75000 |
| 1785 | Zr1 | Zr | 0.25000 | 0.94006 | 0.75000 |
| 1786 | Zr1 | Zr | 0.25000 | 0.69006 | 0.50000 |
| 1787 | Zr1 | Zr | 0.00000 | 0.50000 | 0.55994 |
| 1788 | Zr1 | Zr | 0.00000 | 0.75000 | 0.80994 |
| 1789 | Zr1 | Zr | 0.25000 | 0.50000 | 0.80994 |
| 1790 | Zr1 | Zr | 0.25000 | 0.75000 | 0.55994 |
| 1791 | Zr1 | Zr | 0.00000 | 0.50000 | 0.94006 |
| 1792 | Zr1 | Zr | 0.00000 | 0.75000 | 0.69006 |
| 1793 | Zr1 | Zr | 0.25000 | 0.50000 | 0.69006 |
| 1794 | Zr1 | Zr | 0.25000 | 0.75000 | 0.94006 |
| 1795 | C2 | C | 0.63375 | 0.00000 | 0.09220 |
| 1796 | C2 | C | 0.63375 | 0.25000 | 0.34220 |
| 1797 | C2 | C | 0.88375 | 0.00000 | 0.34220 |
| 1798 | C2 | C | 0.88375 | 0.25000 | 0.09220 |
| 1799 | C2 | C | 0.86625 | 0.00000 | 0.09220 |
| 1800 | C2 | C | 0.86625 | 0.25000 | 0.34220 |
| 1801 | C2 | C | 0.61625 | 0.00000 | 0.34220 |
| 1802 | C2 | C | 0.61625 | 0.25000 | 0.09220 |
| 1803 | C2 | C | 0.86625 | 0.00000 | 0.40780 |
| 1804 | C2 | C | 0.86625 | 0.25000 | 0.15780 |
| 1805 | C2 | C | 0.61625 | 0.00000 | 0.15780 |
| 1806 | C2 | C | 0.61625 | 0.25000 | 0.40780 |
| 1807 | C2 | C | 0.63375 | 0.00000 | 0.40780 |
| 1808 | C2 | C | 0.63375 | 0.25000 | 0.15780 |
| 1809 | C2 | C | 0.88375 | 0.00000 | 0.15780 |
| 1810 | C2 | C | 0.88375 | 0.25000 | 0.40780 |
| 1811 | C2 | C | 0.59220 | 0.13375 | 0.00000 |
| 1812 | C2 | C | 0.84220 | 0.13375 | 0.25000 |
| 1813 | C2 | C | 0.84220 | 0.38375 | 0.00000 |
| 1814 | C2 | C | 0.59220 | 0.36625 | 0.00000 |
| 1815 | C2 | C | 0.59220 | 0.11625 | 0.25000 |
| 1816 | C2 | C | 0.84220 | 0.36625 | 0.25000 |
| 1817 | C2 | C | 0.84220 | 0.11625 | 0.00000 |
| 1818 | C2 | C | 0.90780 | 0.36625 | 0.00000 |
| 1819 | C2 | C | 0.90780 | 0.11625 | 0.25000 |
| 1820 | C2 | C | 0.65780 | 0.11625 | 0.00000 |
| 1821 | C2 | C | 0.90780 | 0.13375 | 0.00000 |
| 1822 | C2 | C | 0.90780 | 0.38375 | 0.25000 |
| 1823 | C2 | C | 0.65780 | 0.13375 | 0.25000 |
| 1824 | C2 | C | 0.65780 | 0.38375 | 0.00000 |
| 1825 | C2 | C | 0.50000 | 0.09220 | 0.13375 |
| 1826 | C2 | C | 0.50000 | 0.34220 | 0.38375 |
| 1827 | C2 | C | 0.75000 | 0.09220 | 0.38375 |
| 1828 | C2 | C | 0.75000 | 0.34220 | 0.13375 |
| 1829 | C2 | C | 0.50000 | 0.09220 | 0.36625 |
| 1830 | C2 | C | 0.50000 | 0.34220 | 0.11625 |
| 1831 | C2 | C | 0.75000 | 0.09220 | 0.11625 |
| 1832 | C2 | C | 0.75000 | 0.34220 | 0.36625 |
| 1833 | C2 | C | 0.50000 | 0.40780 | 0.36625 |
| 1834 | C2 | C | 0.50000 | 0.15780 | 0.11625 |
| 1835 | C2 | C | 0.75000 | 0.40780 | 0.11625 |
| 1836 | C2 | C | 0.75000 | 0.15780 | 0.36625 |

| | | | | | |
|---|---|---|---|---|---|
| 1837 | C2 | C | 0.50000 | 0.40780 | 0.13375 |
| 1838 | C2 | C | 0.50000 | 0.15780 | 0.38375 |
| 1839 | C2 | C | 0.75000 | 0.40780 | 0.38375 |
| 1840 | C2 | C | 0.75000 | 0.15780 | 0.13375 |
| 1841 | C2 | C | 0.50000 | 0.13375 | 0.40780 |
| 1842 | C2 | C | 0.50000 | 0.38375 | 0.15780 |
| 1843 | C2 | C | 0.75000 | 0.13375 | 0.15780 |
| 1844 | C2 | C | 0.75000 | 0.38375 | 0.40780 |
| 1845 | C2 | C | 0.50000 | 0.36625 | 0.40780 |
| 1846 | C2 | C | 0.50000 | 0.11625 | 0.15780 |
| 1847 | C2 | C | 0.75000 | 0.36625 | 0.15780 |
| 1848 | C2 | C | 0.75000 | 0.11625 | 0.40780 |
| 1849 | C2 | C | 0.50000 | 0.36625 | 0.09220 |
| 1850 | C2 | C | 0.50000 | 0.11625 | 0.34220 |
| 1851 | C2 | C | 0.75000 | 0.36625 | 0.34220 |
| 1852 | C2 | C | 0.75000 | 0.11625 | 0.09220 |
| 1853 | C2 | C | 0.50000 | 0.13375 | 0.09220 |
| 1854 | C2 | C | 0.50000 | 0.38375 | 0.34220 |
| 1855 | C2 | C | 0.75000 | 0.13375 | 0.34220 |
| 1856 | C2 | C | 0.75000 | 0.38375 | 0.09220 |
| 1857 | C2 | C | 0.63375 | 0.09220 | 0.00000 |
| 1858 | C2 | C | 0.88375 | 0.09220 | 0.25000 |
| 1859 | C2 | C | 0.88375 | 0.34220 | 0.00000 |
| 1860 | C2 | C | 0.86625 | 0.09220 | 0.00000 |
| 1861 | C2 | C | 0.86625 | 0.34220 | 0.25000 |
| 1862 | C2 | C | 0.61625 | 0.09220 | 0.25000 |
| 1863 | C2 | C | 0.61625 | 0.34220 | 0.00000 |
| 1864 | C2 | C | 0.86625 | 0.40780 | 0.00000 |
| 1865 | C2 | C | 0.86625 | 0.15780 | 0.25000 |
| 1866 | C2 | C | 0.61625 | 0.15780 | 0.00000 |
| 1867 | C2 | C | 0.63375 | 0.40780 | 0.00000 |
| 1868 | C2 | C | 0.63375 | 0.15780 | 0.25000 |
| 1869 | C2 | C | 0.88375 | 0.40780 | 0.25000 |
| 1870 | C2 | C | 0.88375 | 0.15780 | 0.00000 |
| 1871 | C2 | C | 0.59220 | 0.00000 | 0.36625 |
| 1872 | C2 | C | 0.59220 | 0.25000 | 0.11625 |
| 1873 | C2 | C | 0.84220 | 0.00000 | 0.11625 |
| 1874 | C2 | C | 0.84220 | 0.25000 | 0.36625 |
| 1875 | C2 | C | 0.59220 | 0.00000 | 0.13375 |
| 1876 | C2 | C | 0.59220 | 0.25000 | 0.38375 |
| 1877 | C2 | C | 0.84220 | 0.00000 | 0.38375 |
| 1878 | C2 | C | 0.84220 | 0.25000 | 0.13375 |
| 1879 | C2 | C | 0.90780 | 0.00000 | 0.13375 |
| 1880 | C2 | C | 0.90780 | 0.25000 | 0.38375 |
| 1881 | C2 | C | 0.65780 | 0.00000 | 0.38375 |
| 1882 | C2 | C | 0.65780 | 0.25000 | 0.13375 |
| 1883 | C2 | C | 0.90780 | 0.00000 | 0.36625 |
| 1884 | C2 | C | 0.90780 | 0.25000 | 0.11625 |
| 1885 | C2 | C | 0.65780 | 0.00000 | 0.11625 |
| 1886 | C2 | C | 0.65780 | 0.25000 | 0.36625 |
| 1887 | C1 | C | 0.57680 | 0.00000 | 0.42320 |
| 1888 | C1 | C | 0.57680 | 0.25000 | 0.17320 |
| 1889 | C1 | C | 0.82680 | 0.00000 | 0.17320 |
| 1890 | C1 | C | 0.82680 | 0.25000 | 0.42320 |

| | | | | | |
|---|---|---|---|---|---|
| 1891 | C1 | C | 0.92320 | 0.00000 | 0.42320 |
| 1892 | C1 | C | 0.92320 | 0.25000 | 0.17320 |
| 1893 | C1 | C | 0.67320 | 0.00000 | 0.17320 |
| 1894 | C1 | C | 0.67320 | 0.25000 | 0.42320 |
| 1895 | C1 | C | 0.92320 | 0.00000 | 0.07680 |
| 1896 | C1 | C | 0.92320 | 0.25000 | 0.32680 |
| 1897 | C1 | C | 0.67320 | 0.00000 | 0.32680 |
| 1898 | C1 | C | 0.67320 | 0.25000 | 0.07680 |
| 1899 | C1 | C | 0.57680 | 0.00000 | 0.07680 |
| 1900 | C1 | C | 0.57680 | 0.25000 | 0.32680 |
| 1901 | C1 | C | 0.82680 | 0.00000 | 0.32680 |
| 1902 | C1 | C | 0.82680 | 0.25000 | 0.07680 |
| 1903 | C1 | C | 0.92320 | 0.07680 | 0.00000 |
| 1904 | C1 | C | 0.92320 | 0.32680 | 0.25000 |
| 1905 | C1 | C | 0.67320 | 0.07680 | 0.25000 |
| 1906 | C1 | C | 0.67320 | 0.32680 | 0.00000 |
| 1907 | C1 | C | 0.92320 | 0.42320 | 0.00000 |
| 1908 | C1 | C | 0.92320 | 0.17320 | 0.25000 |
| 1909 | C1 | C | 0.67320 | 0.17320 | 0.00000 |
| 1910 | C1 | C | 0.57680 | 0.42320 | 0.00000 |
| 1911 | C1 | C | 0.57680 | 0.17320 | 0.25000 |
| 1912 | C1 | C | 0.82680 | 0.42320 | 0.25000 |
| 1913 | C1 | C | 0.82680 | 0.17320 | 0.00000 |
| 1914 | C1 | C | 0.57680 | 0.07680 | 0.00000 |
| 1915 | C1 | C | 0.82680 | 0.07680 | 0.25000 |
| 1916 | C1 | C | 0.82680 | 0.32680 | 0.00000 |
| 1917 | C1 | C | 0.50000 | 0.42320 | 0.07680 |
| 1918 | C1 | C | 0.50000 | 0.17320 | 0.32680 |
| 1919 | C1 | C | 0.75000 | 0.42320 | 0.32680 |
| 1920 | C1 | C | 0.75000 | 0.17320 | 0.07680 |
| 1921 | C1 | C | 0.50000 | 0.42320 | 0.42320 |
| 1922 | C1 | C | 0.50000 | 0.17320 | 0.17320 |
| 1923 | C1 | C | 0.75000 | 0.42320 | 0.17320 |
| 1924 | C1 | C | 0.75000 | 0.17320 | 0.42320 |
| 1925 | C1 | C | 0.50000 | 0.07680 | 0.42320 |
| 1926 | C1 | C | 0.50000 | 0.32680 | 0.17320 |
| 1927 | C1 | C | 0.75000 | 0.07680 | 0.17320 |
| 1928 | C1 | C | 0.75000 | 0.32680 | 0.42320 |
| 1929 | C1 | C | 0.50000 | 0.07680 | 0.07680 |
| 1930 | C1 | C | 0.50000 | 0.32680 | 0.32680 |
| 1931 | C1 | C | 0.75000 | 0.07680 | 0.32680 |
| 1932 | C1 | C | 0.75000 | 0.32680 | 0.07680 |
| 1933 | C3 | C | 0.60250 | 0.00000 | 0.10250 |
| 1934 | C3 | C | 0.60250 | 0.25000 | 0.35250 |
| 1935 | C3 | C | 0.85250 | 0.00000 | 0.35250 |
| 1936 | C3 | C | 0.85250 | 0.25000 | 0.10250 |
| 1937 | C3 | C | 0.89750 | 0.00000 | 0.10250 |
| 1938 | C3 | C | 0.89750 | 0.25000 | 0.35250 |
| 1939 | C3 | C | 0.64750 | 0.00000 | 0.35250 |
| 1940 | C3 | C | 0.64750 | 0.25000 | 0.10250 |
| 1941 | C3 | C | 0.89750 | 0.00000 | 0.39750 |
| 1942 | C3 | C | 0.89750 | 0.25000 | 0.14750 |
| 1943 | C3 | C | 0.64750 | 0.00000 | 0.14750 |
| 1944 | C3 | C | 0.64750 | 0.25000 | 0.39750 |

| | | | | | |
|---|---|---|---|---|---|
| 1945 | C3 | C | 0.60250 | 0.00000 | 0.39750 |
| 1946 | C3 | C | 0.60250 | 0.25000 | 0.14750 |
| 1947 | C3 | C | 0.85250 | 0.00000 | 0.14750 |
| 1948 | C3 | C | 0.85250 | 0.25000 | 0.39750 |
| 1949 | C3 | C | 0.60250 | 0.10250 | 0.00000 |
| 1950 | C3 | C | 0.85250 | 0.10250 | 0.25000 |
| 1951 | C3 | C | 0.85250 | 0.35250 | 0.00000 |
| 1952 | C3 | C | 0.60250 | 0.39750 | 0.00000 |
| 1953 | C3 | C | 0.60250 | 0.14750 | 0.25000 |
| 1954 | C3 | C | 0.85250 | 0.39750 | 0.25000 |
| 1955 | C3 | C | 0.85250 | 0.14750 | 0.00000 |
| 1956 | C3 | C | 0.89750 | 0.39750 | 0.00000 |
| 1957 | C3 | C | 0.89750 | 0.14750 | 0.25000 |
| 1958 | C3 | C | 0.64750 | 0.14750 | 0.00000 |
| 1959 | C3 | C | 0.89750 | 0.10250 | 0.00000 |
| 1960 | C3 | C | 0.89750 | 0.35250 | 0.25000 |
| 1961 | C3 | C | 0.64750 | 0.10250 | 0.25000 |
| 1962 | C3 | C | 0.64750 | 0.35250 | 0.00000 |
| 1963 | C3 | C | 0.50000 | 0.10250 | 0.10250 |
| 1964 | C3 | C | 0.50000 | 0.35250 | 0.35250 |
| 1965 | C3 | C | 0.75000 | 0.10250 | 0.35250 |
| 1966 | C3 | C | 0.75000 | 0.35250 | 0.10250 |
| 1967 | C3 | C | 0.50000 | 0.10250 | 0.39750 |
| 1968 | C3 | C | 0.50000 | 0.35250 | 0.14750 |
| 1969 | C3 | C | 0.75000 | 0.10250 | 0.14750 |
| 1970 | C3 | C | 0.75000 | 0.35250 | 0.39750 |
| 1971 | C3 | C | 0.50000 | 0.39750 | 0.39750 |
| 1972 | C3 | C | 0.50000 | 0.14750 | 0.14750 |
| 1973 | C3 | C | 0.75000 | 0.39750 | 0.14750 |
| 1974 | C3 | C | 0.75000 | 0.14750 | 0.39750 |
| 1975 | C3 | C | 0.50000 | 0.39750 | 0.10250 |
| 1976 | C3 | C | 0.50000 | 0.14750 | 0.35250 |
| 1977 | C3 | C | 0.75000 | 0.39750 | 0.35250 |
| 1978 | C3 | C | 0.75000 | 0.14750 | 0.10250 |
| 1979 | H1 | H | 0.63926 | 0.49997 | 0.06655 |
| 1980 | H1 | H | 0.63926 | 0.24997 | 0.31655 |
| 1981 | H1 | H | 0.88926 | 0.49997 | 0.31655 |
| 1982 | H1 | H | 0.88926 | 0.24997 | 0.06655 |
| 1983 | H1 | H | 0.86074 | 0.00003 | 0.06655 |
| 1984 | H1 | H | 0.86074 | 0.25004 | 0.31655 |
| 1985 | H1 | H | 0.61074 | 0.00003 | 0.31655 |
| 1986 | H1 | H | 0.61074 | 0.25004 | 0.06655 |
| 1987 | H1 | H | 0.86074 | 0.49996 | 0.43345 |
| 1988 | H1 | H | 0.86074 | 0.24997 | 0.18345 |
| 1989 | H1 | H | 0.61074 | 0.49997 | 0.18345 |
| 1990 | H1 | H | 0.61074 | 0.24997 | 0.43346 |
| 1991 | H1 | H | 0.63926 | 0.00003 | 0.43345 |
| 1992 | H1 | H | 0.63926 | 0.25003 | 0.18346 |
| 1993 | H1 | H | 0.88926 | 0.00003 | 0.18345 |
| 1994 | H1 | H | 0.88926 | 0.25004 | 0.43346 |
| 1995 | H1 | H | 0.56655 | 0.13926 | 0.49996 |
| 1996 | H1 | H | 0.81655 | 0.13926 | 0.24996 |
| 1997 | H1 | H | 0.81654 | 0.38926 | 0.49997 |
| 1998 | H1 | H | 0.56655 | 0.36074 | 0.00003 |

| | | | | | |
|---|---|---|---|---|---|
| 1999 | H1 | H | 0.56655 | 0.11074 | 0.25004 |
| 2000 | H1 | H | 0.81655 | 0.36074 | 0.25003 |
| 2001 | H1 | H | 0.81655 | 0.11074 | 0.00004 |
| 2002 | H1 | H | 0.93345 | 0.36074 | 0.49996 |
| 2003 | H1 | H | 0.93345 | 0.11074 | 0.24997 |
| 2004 | H1 | H | 0.68345 | 0.11074 | 0.49997 |
| 2005 | H1 | H | 0.93345 | 0.13926 | 0.00003 |
| 2006 | H1 | H | 0.93345 | 0.38926 | 0.25004 |
| 2007 | H1 | H | 0.68345 | 0.13926 | 0.25003 |
| 2008 | H1 | H | 0.68345 | 0.38926 | 0.00004 |
| 2009 | H1 | H | 0.99997 | 0.06655 | 0.13926 |
| 2010 | H1 | H | 0.99996 | 0.31655 | 0.38926 |
| 2011 | H1 | H | 0.74997 | 0.06655 | 0.38926 |
| 2012 | H1 | H | 0.74996 | 0.31655 | 0.13926 |
| 2013 | H1 | H | 0.50003 | 0.06655 | 0.36074 |
| 2014 | H1 | H | 0.50003 | 0.31655 | 0.11074 |
| 2015 | H1 | H | 0.75003 | 0.06655 | 0.11074 |
| 2016 | H1 | H | 0.75004 | 0.31655 | 0.36074 |
| 2017 | H1 | H | 0.99997 | 0.43345 | 0.36074 |
| 2018 | H1 | H | 0.99997 | 0.18345 | 0.11074 |
| 2019 | H1 | H | 0.74997 | 0.43345 | 0.11074 |
| 2020 | H1 | H | 0.74997 | 0.18345 | 0.36074 |
| 2021 | H1 | H | 0.50003 | 0.43345 | 0.13926 |
| 2022 | H1 | H | 0.50003 | 0.18345 | 0.38926 |
| 2023 | H1 | H | 0.75004 | 0.43345 | 0.38926 |
| 2024 | H1 | H | 0.75003 | 0.18346 | 0.13926 |
| 2025 | H1 | H | 0.50003 | 0.13926 | 0.43345 |
| 2026 | H1 | H | 0.50003 | 0.38926 | 0.18345 |
| 2027 | H1 | H | 0.75003 | 0.13926 | 0.18345 |
| 2028 | H1 | H | 0.75004 | 0.38926 | 0.43345 |
| 2029 | H1 | H | 0.99996 | 0.36074 | 0.43345 |
| 2030 | H1 | H | 0.99997 | 0.11074 | 0.18345 |
| 2031 | H1 | H | 0.74997 | 0.36074 | 0.18345 |
| 2032 | H1 | H | 0.74997 | 0.11074 | 0.43345 |
| 2033 | H1 | H | 0.50003 | 0.36074 | 0.06655 |
| 2034 | H1 | H | 0.50003 | 0.11074 | 0.31655 |
| 2035 | H1 | H | 0.75004 | 0.36074 | 0.31655 |
| 2036 | H1 | H | 0.75003 | 0.11074 | 0.06654 |
| 2037 | H1 | H | 0.99997 | 0.13926 | 0.06655 |
| 2038 | H1 | H | 0.99997 | 0.38926 | 0.31655 |
| 2039 | H1 | H | 0.74997 | 0.13926 | 0.31655 |
| 2040 | H1 | H | 0.74997 | 0.38926 | 0.06654 |
| 2041 | H1 | H | 0.63926 | 0.06655 | 0.49997 |
| 2042 | H1 | H | 0.88926 | 0.06655 | 0.24997 |
| 2043 | H1 | H | 0.88926 | 0.31655 | 0.49996 |
| 2044 | H1 | H | 0.86074 | 0.06655 | 0.00003 |
| 2045 | H1 | H | 0.86074 | 0.31655 | 0.25003 |
| 2046 | H1 | H | 0.61074 | 0.06655 | 0.25003 |
| 2047 | H1 | H | 0.61074 | 0.31655 | 0.00003 |
| 2048 | H1 | H | 0.86074 | 0.43345 | 0.49997 |
| 2049 | H1 | H | 0.86074 | 0.18345 | 0.24997 |
| 2050 | H1 | H | 0.61074 | 0.18345 | 0.49996 |
| 2051 | H1 | H | 0.63926 | 0.43345 | 0.00004 |
| 2052 | H1 | H | 0.63926 | 0.18346 | 0.25003 |

| | | | | | |
|---|---|---|---|---|---|
| 2053 | H1 | H | 0.88926 | 0.43345 | 0.25004 |
| 2054 | H1 | H | 0.88926 | 0.18346 | 0.00003 |
| 2055 | H1 | H | 0.56655 | 0.00003 | 0.36074 |
| 2056 | H1 | H | 0.56655 | 0.25004 | 0.11074 |
| 2057 | H1 | H | 0.81655 | 0.00003 | 0.11074 |
| 2058 | H1 | H | 0.81655 | 0.25004 | 0.36074 |
| 2059 | H1 | H | 0.56655 | 0.49996 | 0.13926 |
| 2060 | H1 | H | 0.56655 | 0.24997 | 0.38926 |
| 2061 | H1 | H | 0.81655 | 0.49996 | 0.38926 |
| 2062 | H1 | H | 0.81655 | 0.24997 | 0.13926 |
| 2063 | H1 | H | 0.93345 | 0.00003 | 0.13926 |
| 2064 | H1 | H | 0.93345 | 0.25004 | 0.38926 |
| 2065 | H1 | H | 0.68345 | 0.00003 | 0.38926 |
| 2066 | H1 | H | 0.68345 | 0.25003 | 0.13926 |
| 2067 | H1 | H | 0.93345 | 0.49996 | 0.36074 |
| 2068 | H1 | H | 0.93345 | 0.24997 | 0.11074 |
| 2069 | H1 | H | 0.68345 | 0.49996 | 0.11074 |
| 2070 | H1 | H | 0.68345 | 0.24997 | 0.36074 |
| 2071 | H2 | H | 0.70825 | 0.29176 | 0.45824 |
| 2072 | H2 | H | 0.79174 | 0.20825 | 0.45823 |
| 2073 | H2 | H | 0.70827 | 0.20823 | 0.04177 |
| 2074 | H2 | H | 0.79179 | 0.29168 | 0.04178 |
| 2075 | H2 | H | 0.95823 | 0.29173 | 0.20823 |
| 2076 | H2 | H | 0.95824 | 0.20825 | 0.29176 |
| 2077 | H2 | H | 0.54174 | 0.29176 | 0.29177 |
| 2078 | H2 | H | 0.54173 | 0.20823 | 0.20823 |
| 2079 | H2 | H | 0.79175 | 0.45824 | 0.20824 |
| 2080 | H2 | H | 0.70824 | 0.45824 | 0.29176 |
| 2081 | H2 | H | 0.70823 | 0.04172 | 0.20822 |
| 2082 | H2 | H | 0.79177 | 0.04173 | 0.29178 |
| 2083 | H2 | H | 0.54178 | 0.04177 | 0.04173 |
| 2084 | H2 | H | 0.95823 | 0.45824 | 0.04174 |
| 2085 | H2 | H | 0.95823 | 0.04176 | 0.45825 |
| 2086 | H2 | H | 0.54176 | 0.45824 | 0.45824 |
| 2087 | O1 | O | 0.58524 | 0.00000 | 0.04765 |
| 2088 | O1 | O | 0.58524 | 0.25000 | 0.29765 |
| 2089 | O1 | O | 0.83524 | 0.00000 | 0.29765 |
| 2090 | O1 | O | 0.83524 | 0.25000 | 0.04765 |
| 2091 | O1 | O | 0.91476 | 0.00000 | 0.04765 |
| 2092 | O1 | O | 0.91476 | 0.25000 | 0.29765 |
| 2093 | O1 | O | 0.66476 | 0.00000 | 0.29765 |
| 2094 | O1 | O | 0.66476 | 0.25000 | 0.04765 |
| 2095 | O1 | O | 0.91476 | 0.00000 | 0.45235 |
| 2096 | O1 | O | 0.91476 | 0.25000 | 0.20235 |
| 2097 | O1 | O | 0.66476 | 0.00000 | 0.20235 |
| 2098 | O1 | O | 0.66476 | 0.25000 | 0.45235 |
| 2099 | O1 | O | 0.58524 | 0.00000 | 0.45235 |
| 2100 | O1 | O | 0.58524 | 0.25000 | 0.20235 |
| 2101 | O1 | O | 0.83524 | 0.00000 | 0.20235 |
| 2102 | O1 | O | 0.83524 | 0.25000 | 0.45235 |
| 2103 | O1 | O | 0.54765 | 0.08524 | 0.00000 |
| 2104 | O3 | O | 0.54765 | 0.33524 | 0.25000 |
| 2105 | O1 | O | 0.79765 | 0.08524 | 0.25000 |
| 2106 | O1 | O | 0.79765 | 0.33524 | 0.00000 |

| | | | | | |
|---|---|---|---|---|---|
| 2107 | O1 | O | 0.54765 | 0.41476 | 0.00000 |
| 2108 | O1 | O | 0.54765 | 0.16476 | 0.25000 |
| 2109 | O1 | O | 0.79765 | 0.41476 | 0.25000 |
| 2110 | O1 | O | 0.79765 | 0.16476 | 0.00000 |
| 2111 | O1 | O | 0.95235 | 0.41476 | 0.00000 |
| 2112 | O1 | O | 0.95235 | 0.16476 | 0.25000 |
| 2113 | O3 | O | 0.70235 | 0.41476 | 0.25000 |
| 2114 | O1 | O | 0.70235 | 0.16476 | 0.00000 |
| 2115 | O1 | O | 0.95235 | 0.08524 | 0.00000 |
| 2116 | O1 | O | 0.95235 | 0.33524 | 0.25000 |
| 2117 | O1 | O | 0.70235 | 0.08524 | 0.25000 |
| 2118 | O1 | O | 0.70235 | 0.33524 | 0.00000 |
| 2119 | O1 | O | 0.50000 | 0.04765 | 0.08524 |
| 2120 | O1 | O | 0.50000 | 0.29765 | 0.33524 |
| 2121 | O1 | O | 0.75000 | 0.04765 | 0.33524 |
| 2122 | O1 | O | 0.75000 | 0.29765 | 0.08524 |
| 2123 | O1 | O | 0.50000 | 0.04765 | 0.41476 |
| 2124 | O1 | O | 0.50000 | 0.29765 | 0.16476 |
| 2125 | O1 | O | 0.75000 | 0.04765 | 0.16476 |
| 2126 | O1 | O | 0.75000 | 0.29765 | 0.41476 |
| 2127 | O1 | O | 0.50000 | 0.45235 | 0.41476 |
| 2128 | O1 | O | 0.50000 | 0.20235 | 0.16476 |
| 2129 | O1 | O | 0.75000 | 0.45235 | 0.16476 |
| 2130 | O1 | O | 0.75000 | 0.20235 | 0.41476 |
| 2131 | O1 | O | 0.50000 | 0.45235 | 0.08524 |
| 2132 | O1 | O | 0.50000 | 0.20235 | 0.33524 |
| 2133 | O1 | O | 0.75000 | 0.45235 | 0.33524 |
| 2134 | O1 | O | 0.75000 | 0.20235 | 0.08524 |
| 2135 | O1 | O | 0.50000 | 0.08524 | 0.45235 |
| 2136 | O1 | O | 0.50000 | 0.33524 | 0.20235 |
| 2137 | O1 | O | 0.75000 | 0.08524 | 0.20235 |
| 2138 | O1 | O | 0.75000 | 0.33524 | 0.45235 |
| 2139 | O1 | O | 0.50000 | 0.41476 | 0.45235 |
| 2140 | O1 | O | 0.50000 | 0.16476 | 0.20235 |
| 2141 | O1 | O | 0.75000 | 0.41476 | 0.20235 |
| 2142 | O1 | O | 0.75000 | 0.16476 | 0.45235 |
| 2143 | O1 | O | 0.50000 | 0.41476 | 0.04765 |
| 2144 | O1 | O | 0.50000 | 0.16476 | 0.29765 |
| 2145 | O1 | O | 0.75000 | 0.41476 | 0.29765 |
| 2146 | O1 | O | 0.75000 | 0.16476 | 0.04765 |
| 2147 | O1 | O | 0.50000 | 0.08524 | 0.04765 |
| 2148 | O1 | O | 0.50000 | 0.33524 | 0.29765 |
| 2149 | O1 | O | 0.75000 | 0.08524 | 0.29765 |
| 2150 | O1 | O | 0.75000 | 0.33524 | 0.04765 |
| 2151 | O1 | O | 0.58524 | 0.04765 | 0.00000 |
| 2152 | O3 | O | 0.58524 | 0.29765 | 0.25000 |
| 2153 | O1 | O | 0.83524 | 0.04765 | 0.25000 |
| 2154 | O1 | O | 0.83524 | 0.29765 | 0.00000 |
| 2155 | O1 | O | 0.91476 | 0.04765 | 0.00000 |
| 2156 | O1 | O | 0.91476 | 0.29765 | 0.25000 |
| 2157 | O1 | O | 0.66476 | 0.04765 | 0.25000 |
| 2158 | O1 | O | 0.66476 | 0.29765 | 0.00000 |
| 2159 | O1 | O | 0.91476 | 0.45235 | 0.00000 |
| 2160 | O1 | O | 0.91476 | 0.20235 | 0.25000 |

| | | | | | |
|---|---|---|---|---|---|
| 2161 | O3 | O | 0.66476 | 0.45235 | 0.25000 |
| 2162 | O1 | O | 0.66476 | 0.20235 | 0.00000 |
| 2163 | O1 | O | 0.58524 | 0.45235 | 0.00000 |
| 2164 | O1 | O | 0.58524 | 0.20235 | 0.25000 |
| 2165 | O1 | O | 0.83524 | 0.45235 | 0.25000 |
| 2166 | O1 | O | 0.83524 | 0.20235 | 0.00000 |
| 2167 | O1 | O | 0.54765 | 0.00000 | 0.41476 |
| 2168 | O1 | O | 0.54765 | 0.25000 | 0.16476 |
| 2169 | O1 | O | 0.79765 | 0.00000 | 0.16476 |
| 2170 | O1 | O | 0.79765 | 0.25000 | 0.41476 |
| 2171 | O1 | O | 0.54765 | 0.00000 | 0.08524 |
| 2172 | O1 | O | 0.54765 | 0.25000 | 0.33524 |
| 2173 | O1 | O | 0.79765 | 0.00000 | 0.33524 |
| 2174 | O1 | O | 0.79765 | 0.25000 | 0.08524 |
| 2175 | O1 | O | 0.95235 | 0.00000 | 0.08524 |
| 2176 | O1 | O | 0.95235 | 0.25000 | 0.33524 |
| 2177 | O1 | O | 0.70235 | 0.00000 | 0.33524 |
| 2178 | O1 | O | 0.70235 | 0.25000 | 0.08524 |
| 2179 | O1 | O | 0.95235 | 0.00000 | 0.41476 |
| 2180 | O1 | O | 0.95235 | 0.25000 | 0.16476 |
| 2181 | O1 | O | 0.70235 | 0.00000 | 0.16476 |
| 2182 | O1 | O | 0.70235 | 0.25000 | 0.41476 |
| 2183 | O2 | O | 0.52805 | 0.47195 | 0.47195 |
| 2184 | O2 | O | 0.52805 | 0.22195 | 0.22195 |
| 2185 | O2 | O | 0.77805 | 0.47195 | 0.22195 |
| 2186 | O2 | O | 0.77805 | 0.22195 | 0.47195 |
| 2187 | O2 | O | 0.97195 | 0.02805 | 0.47195 |
| 2188 | O2 | O | 0.97195 | 0.27805 | 0.22195 |
| 2189 | O2 | O | 0.72195 | 0.02805 | 0.22195 |
| 2190 | O2 | O | 0.72195 | 0.27805 | 0.47195 |
| 2191 | O2 | O | 0.97195 | 0.47195 | 0.02805 |
| 2192 | O2 | O | 0.97195 | 0.22195 | 0.27805 |
| 2193 | O2 | O | 0.72195 | 0.47195 | 0.27805 |
| 2194 | O2 | O | 0.72195 | 0.22195 | 0.02805 |
| 2195 | O2 | O | 0.52805 | 0.02805 | 0.02805 |
| 2196 | O2 | O | 0.52805 | 0.27805 | 0.27805 |
| 2197 | O2 | O | 0.77805 | 0.02805 | 0.27805 |
| 2198 | O2 | O | 0.77805 | 0.27805 | 0.02805 |
| 2199 | O1 | O | 0.97195 | 0.02805 | 0.02805 |
| 2200 | O1 | O | 0.97195 | 0.27805 | 0.27805 |
| 2201 | O1 | O | 0.72195 | 0.02805 | 0.27805 |
| 2202 | O1 | O | 0.72195 | 0.27805 | 0.02805 |
| 2203 | O1 | O | 0.52805 | 0.47195 | 0.02805 |
| 2204 | O1 | O | 0.52805 | 0.22195 | 0.27805 |
| 2205 | O1 | O | 0.77805 | 0.47195 | 0.27805 |
| 2206 | O1 | O | 0.77805 | 0.22195 | 0.02805 |
| 2207 | O1 | O | 0.97195 | 0.47195 | 0.47195 |
| 2208 | O1 | O | 0.97195 | 0.22195 | 0.22195 |
| 2209 | O1 | O | 0.72195 | 0.47195 | 0.22195 |
| 2210 | O1 | O | 0.72195 | 0.22195 | 0.47195 |
| 2211 | O1 | O | 0.52805 | 0.02805 | 0.47195 |
| 2212 | O1 | O | 0.52805 | 0.27805 | 0.22195 |
| 2213 | O1 | O | 0.77805 | 0.02805 | 0.22195 |
| 2214 | O1 | O | 0.77805 | 0.27805 | 0.47195 |

| | | | | | |
|---|---|---|---|---|---|
| 2215 | Zr1 | Zr | 0.55994 | 0.00000 | 0.00000 |
| 2216 | Zr1 | Zr | 0.55994 | 0.25000 | 0.25000 |
| 2217 | Zr1 | Zr | 0.80994 | 0.00000 | 0.25000 |
| 2218 | Zr1 | Zr | 0.80994 | 0.25000 | 0.00000 |
| 2219 | Zr1 | Zr | 0.94006 | 0.00000 | 0.00000 |
| 2220 | Zr1 | Zr | 0.94006 | 0.25000 | 0.25000 |
| 2221 | Zr1 | Zr | 0.69006 | 0.00000 | 0.25000 |
| 2222 | Zr1 | Zr | 0.69006 | 0.25000 | 0.00000 |
| 2223 | Zr1 | Zr | 0.50000 | 0.05994 | 0.00000 |
| 2224 | Zr1 | Zr | 0.50000 | 0.30994 | 0.25000 |
| 2225 | Zr1 | Zr | 0.75000 | 0.05994 | 0.25000 |
| 2226 | Zr1 | Zr | 0.75000 | 0.30994 | 0.00000 |
| 2227 | Zr1 | Zr | 0.50000 | 0.44006 | 0.00000 |
| 2228 | Zr1 | Zr | 0.50000 | 0.19006 | 0.25000 |
| 2229 | Zr1 | Zr | 0.75000 | 0.44006 | 0.25000 |
| 2230 | Zr1 | Zr | 0.75000 | 0.19006 | 0.00000 |
| 2231 | Zr1 | Zr | 0.50000 | 0.00000 | 0.05994 |
| 2232 | Zr1 | Zr | 0.50000 | 0.25000 | 0.30994 |
| 2233 | Zr1 | Zr | 0.75000 | 0.00000 | 0.30994 |
| 2234 | Zr1 | Zr | 0.75000 | 0.25000 | 0.05994 |
| 2235 | Zr1 | Zr | 0.50000 | 0.00000 | 0.44006 |
| 2236 | Zr1 | Zr | 0.50000 | 0.25000 | 0.19006 |
| 2237 | Zr1 | Zr | 0.75000 | 0.00000 | 0.19006 |
| 2238 | Zr1 | Zr | 0.75000 | 0.25000 | 0.44006 |
| 2239 | C2 | C | 0.63375 | 0.00000 | 0.59220 |
| 2240 | C2 | C | 0.63375 | 0.25000 | 0.84220 |
| 2241 | C2 | C | 0.88375 | 0.00000 | 0.84220 |
| 2242 | C2 | C | 0.88375 | 0.25000 | 0.59220 |
| 2243 | C2 | C | 0.86625 | 0.00000 | 0.59220 |
| 2244 | C2 | C | 0.86625 | 0.25000 | 0.84220 |
| 2245 | C2 | C | 0.61625 | 0.00000 | 0.84220 |
| 2246 | C2 | C | 0.61625 | 0.25000 | 0.59220 |
| 2247 | C2 | C | 0.86625 | 0.00000 | 0.90780 |
| 2248 | C2 | C | 0.86625 | 0.25000 | 0.65780 |
| 2249 | C2 | C | 0.61625 | 0.00000 | 0.65780 |
| 2250 | C2 | C | 0.61625 | 0.25000 | 0.90780 |
| 2251 | C2 | C | 0.63375 | 0.00000 | 0.90780 |
| 2252 | C2 | C | 0.63375 | 0.25000 | 0.65780 |
| 2253 | C2 | C | 0.88375 | 0.00000 | 0.65780 |
| 2254 | C2 | C | 0.88375 | 0.25000 | 0.90780 |
| 2255 | C2 | C | 0.59220 | 0.13375 | 0.50000 |
| 2256 | C2 | C | 0.59220 | 0.38375 | 0.75000 |
| 2257 | C2 | C | 0.84220 | 0.13375 | 0.75000 |
| 2258 | C2 | C | 0.84220 | 0.38375 | 0.50000 |
| 2259 | C2 | C | 0.59220 | 0.36625 | 0.50000 |
| 2260 | C2 | C | 0.59220 | 0.11625 | 0.75000 |
| 2261 | C2 | C | 0.84220 | 0.36625 | 0.75000 |
| 2262 | C2 | C | 0.90780 | 0.36625 | 0.50000 |
| 2263 | C2 | C | 0.90780 | 0.11625 | 0.75000 |
| 2264 | C2 | C | 0.65780 | 0.36625 | 0.75000 |
| 2265 | C2 | C | 0.65780 | 0.11625 | 0.50000 |
| 2266 | C2 | C | 0.90780 | 0.38375 | 0.75000 |
| 2267 | C2 | C | 0.65780 | 0.13375 | 0.75000 |
| 2268 | C2 | C | 0.65780 | 0.38375 | 0.50000 |

| | | | | | |
|---|---|---|---|---|---|
| 2269 | C2 | C | 0.50000 | 0.09220 | 0.63375 |
| 2270 | C2 | C | 0.50000 | 0.34220 | 0.88375 |
| 2271 | C2 | C | 0.75000 | 0.09220 | 0.88375 |
| 2272 | C2 | C | 0.75000 | 0.34220 | 0.63375 |
| 2273 | C2 | C | 0.50000 | 0.09220 | 0.86625 |
| 2274 | C2 | C | 0.50000 | 0.34220 | 0.61625 |
| 2275 | C2 | C | 0.75000 | 0.09220 | 0.61625 |
| 2276 | C2 | C | 0.75000 | 0.34220 | 0.86625 |
| 2277 | C2 | C | 0.50000 | 0.40780 | 0.86625 |
| 2278 | C2 | C | 0.50000 | 0.15780 | 0.61625 |
| 2279 | C2 | C | 0.75000 | 0.40780 | 0.61625 |
| 2280 | C2 | C | 0.75000 | 0.15780 | 0.86625 |
| 2281 | C2 | C | 0.50000 | 0.40780 | 0.63375 |
| 2282 | C2 | C | 0.50000 | 0.15780 | 0.88375 |
| 2283 | C2 | C | 0.75000 | 0.40780 | 0.88375 |
| 2284 | C2 | C | 0.75000 | 0.15780 | 0.63375 |
| 2285 | C2 | C | 0.50000 | 0.13375 | 0.90780 |
| 2286 | C2 | C | 0.50000 | 0.38375 | 0.65780 |
| 2287 | C2 | C | 0.75000 | 0.13375 | 0.65780 |
| 2288 | C2 | C | 0.75000 | 0.38375 | 0.90780 |
| 2289 | C2 | C | 0.50000 | 0.36625 | 0.90780 |
| 2290 | C2 | C | 0.50000 | 0.11625 | 0.65780 |
| 2291 | C2 | C | 0.75000 | 0.36625 | 0.65780 |
| 2292 | C2 | C | 0.75000 | 0.11625 | 0.90780 |
| 2293 | C2 | C | 0.50000 | 0.36625 | 0.59220 |
| 2294 | C2 | C | 0.50000 | 0.11625 | 0.84220 |
| 2295 | C2 | C | 0.75000 | 0.36625 | 0.84220 |
| 2296 | C2 | C | 0.75000 | 0.11625 | 0.59220 |
| 2297 | C2 | C | 0.50000 | 0.13375 | 0.59220 |
| 2298 | C2 | C | 0.50000 | 0.38375 | 0.84220 |
| 2299 | C2 | C | 0.75000 | 0.13375 | 0.84220 |
| 2300 | C2 | C | 0.75000 | 0.38375 | 0.59220 |
| 2301 | C2 | C | 0.63375 | 0.09220 | 0.50000 |
| 2302 | C2 | C | 0.63375 | 0.34220 | 0.75000 |
| 2303 | C2 | C | 0.88375 | 0.09220 | 0.75000 |
| 2304 | C2 | C | 0.88375 | 0.34220 | 0.50000 |
| 2305 | C2 | C | 0.86625 | 0.34220 | 0.75000 |
| 2306 | C2 | C | 0.61625 | 0.09220 | 0.75000 |
| 2307 | C2 | C | 0.61625 | 0.34220 | 0.50000 |
| 2308 | C2 | C | 0.86625 | 0.40780 | 0.50000 |
| 2309 | C2 | C | 0.86625 | 0.15780 | 0.75000 |
| 2310 | C2 | C | 0.61625 | 0.40780 | 0.75000 |
| 2311 | C2 | C | 0.61625 | 0.15780 | 0.50000 |
| 2312 | C2 | C | 0.63375 | 0.40780 | 0.50000 |
| 2313 | C2 | C | 0.63375 | 0.15780 | 0.75000 |
| 2314 | C2 | C | 0.88375 | 0.40780 | 0.75000 |
| 2315 | C2 | C | 0.59220 | 0.00000 | 0.86625 |
| 2316 | C2 | C | 0.59220 | 0.25000 | 0.61625 |
| 2317 | C2 | C | 0.84220 | 0.00000 | 0.61625 |
| 2318 | C2 | C | 0.84220 | 0.25000 | 0.86625 |
| 2319 | C2 | C | 0.59220 | 0.00000 | 0.63375 |
| 2320 | C2 | C | 0.59220 | 0.25000 | 0.88375 |
| 2321 | C2 | C | 0.84220 | 0.00000 | 0.88375 |
| 2322 | C2 | C | 0.84220 | 0.25000 | 0.63375 |

| | | | | | |
|---|---|---|---|---|---|
| 2323 | C2 | C | 0.90780 | 0.00000 | 0.63375 |
| 2324 | C2 | C | 0.90780 | 0.25000 | 0.88375 |
| 2325 | C2 | C | 0.65780 | 0.00000 | 0.88375 |
| 2326 | C2 | C | 0.65780 | 0.25000 | 0.63375 |
| 2327 | C2 | C | 0.90780 | 0.00000 | 0.86625 |
| 2328 | C2 | C | 0.90780 | 0.25000 | 0.61625 |
| 2329 | C2 | C | 0.65780 | 0.00000 | 0.61625 |
| 2330 | C2 | C | 0.65780 | 0.25000 | 0.86625 |
| 2331 | C1 | C | 0.57680 | 0.00000 | 0.92320 |
| 2332 | C1 | C | 0.57680 | 0.25000 | 0.67320 |
| 2333 | C1 | C | 0.82680 | 0.00000 | 0.67320 |
| 2334 | C1 | C | 0.82680 | 0.25000 | 0.92320 |
| 2335 | C1 | C | 0.92320 | 0.00000 | 0.92320 |
| 2336 | C1 | C | 0.92320 | 0.25000 | 0.67320 |
| 2337 | C1 | C | 0.67320 | 0.00000 | 0.67320 |
| 2338 | C1 | C | 0.67320 | 0.25000 | 0.92320 |
| 2339 | C1 | C | 0.92320 | 0.00000 | 0.57680 |
| 2340 | C1 | C | 0.92320 | 0.25000 | 0.82680 |
| 2341 | C1 | C | 0.67320 | 0.00000 | 0.82680 |
| 2342 | C1 | C | 0.67320 | 0.25000 | 0.57680 |
| 2343 | C1 | C | 0.57680 | 0.00000 | 0.57680 |
| 2344 | C1 | C | 0.57680 | 0.25000 | 0.82680 |
| 2345 | C1 | C | 0.82680 | 0.00000 | 0.82680 |
| 2346 | C1 | C | 0.82680 | 0.25000 | 0.57680 |
| 2347 | C1 | C | 0.92320 | 0.32680 | 0.75000 |
| 2348 | C1 | C | 0.67320 | 0.07680 | 0.75000 |
| 2349 | C1 | C | 0.67320 | 0.32680 | 0.50000 |
| 2350 | C1 | C | 0.92320 | 0.42320 | 0.50000 |
| 2351 | C1 | C | 0.92320 | 0.17320 | 0.75000 |
| 2352 | C1 | C | 0.67320 | 0.42320 | 0.75000 |
| 2353 | C1 | C | 0.67320 | 0.17320 | 0.50000 |
| 2354 | C1 | C | 0.57680 | 0.42320 | 0.50000 |
| 2355 | C1 | C | 0.57680 | 0.17320 | 0.75000 |
| 2356 | C1 | C | 0.82680 | 0.42320 | 0.75000 |
| 2357 | C1 | C | 0.57680 | 0.07680 | 0.50000 |
| 2358 | C1 | C | 0.57680 | 0.32680 | 0.75000 |
| 2359 | C1 | C | 0.82680 | 0.07680 | 0.75000 |
| 2360 | C1 | C | 0.82680 | 0.32680 | 0.50000 |
| 2361 | C1 | C | 0.50000 | 0.42320 | 0.57680 |
| 2362 | C1 | C | 0.50000 | 0.17320 | 0.82680 |
| 2363 | C1 | C | 0.75000 | 0.42320 | 0.82680 |
| 2364 | C1 | C | 0.75000 | 0.17320 | 0.57680 |
| 2365 | C1 | C | 0.50000 | 0.42320 | 0.92320 |
| 2366 | C1 | C | 0.50000 | 0.17320 | 0.67320 |
| 2367 | C1 | C | 0.75000 | 0.42320 | 0.67320 |
| 2368 | C1 | C | 0.75000 | 0.17320 | 0.92320 |
| 2369 | C1 | C | 0.50000 | 0.07680 | 0.92320 |
| 2370 | C1 | C | 0.50000 | 0.32680 | 0.67320 |
| 2371 | C1 | C | 0.75000 | 0.07680 | 0.67320 |
| 2372 | C1 | C | 0.75000 | 0.32680 | 0.92320 |
| 2373 | C1 | C | 0.50000 | 0.07680 | 0.57680 |
| 2374 | C1 | C | 0.50000 | 0.32680 | 0.82680 |
| 2375 | C1 | C | 0.75000 | 0.07680 | 0.82680 |
| 2376 | C1 | C | 0.75000 | 0.32680 | 0.57680 |

| | | | | | |
|---|---|---|---|---|---|
| 2377 | C3 | C | 0.60250 | 0.00000 | 0.60250 |
| 2378 | C3 | C | 0.60250 | 0.25000 | 0.85250 |
| 2379 | C3 | C | 0.85250 | 0.00000 | 0.85250 |
| 2380 | C3 | C | 0.85250 | 0.25000 | 0.60250 |
| 2381 | C3 | C | 0.89750 | 0.00000 | 0.60250 |
| 2382 | C3 | C | 0.89750 | 0.25000 | 0.85250 |
| 2383 | C3 | C | 0.64750 | 0.00000 | 0.85250 |
| 2384 | C3 | C | 0.64750 | 0.25000 | 0.60250 |
| 2385 | C3 | C | 0.89750 | 0.00000 | 0.89750 |
| 2386 | C3 | C | 0.89750 | 0.25000 | 0.64750 |
| 2387 | C3 | C | 0.64750 | 0.00000 | 0.64750 |
| 2388 | C3 | C | 0.64750 | 0.25000 | 0.89750 |
| 2389 | C3 | C | 0.60250 | 0.00000 | 0.89750 |
| 2390 | C3 | C | 0.60250 | 0.25000 | 0.64750 |
| 2391 | C3 | C | 0.85250 | 0.00000 | 0.64750 |
| 2392 | C3 | C | 0.85250 | 0.25000 | 0.89750 |
| 2393 | C3 | C | 0.60250 | 0.10250 | 0.50000 |
| 2394 | C3 | C | 0.60250 | 0.35250 | 0.75000 |
| 2395 | C3 | C | 0.85250 | 0.10250 | 0.75000 |
| 2396 | C3 | C | 0.85250 | 0.35250 | 0.50000 |
| 2397 | C3 | C | 0.60250 | 0.39750 | 0.50000 |
| 2398 | C3 | C | 0.60250 | 0.14750 | 0.75000 |
| 2399 | C3 | C | 0.85250 | 0.39750 | 0.75000 |
| 2400 | C3 | C | 0.89750 | 0.39750 | 0.50000 |
| 2401 | C3 | C | 0.89750 | 0.14750 | 0.75000 |
| 2402 | C3 | C | 0.64750 | 0.39750 | 0.75000 |
| 2403 | C3 | C | 0.64750 | 0.14750 | 0.50000 |
| 2404 | C3 | C | 0.89750 | 0.35250 | 0.75000 |
| 2405 | C3 | C | 0.64750 | 0.10250 | 0.75000 |
| 2406 | C3 | C | 0.64750 | 0.35250 | 0.50000 |
| 2407 | C3 | C | 0.50000 | 0.10250 | 0.60250 |
| 2408 | C3 | C | 0.50000 | 0.35250 | 0.85250 |
| 2409 | C3 | C | 0.75000 | 0.10250 | 0.85250 |
| 2410 | C3 | C | 0.75000 | 0.35250 | 0.60250 |
| 2411 | C3 | C | 0.50000 | 0.10250 | 0.89750 |
| 2412 | C3 | C | 0.50000 | 0.35250 | 0.64750 |
| 2413 | C3 | C | 0.75000 | 0.10250 | 0.64750 |
| 2414 | C3 | C | 0.75000 | 0.35250 | 0.89750 |
| 2415 | C3 | C | 0.50000 | 0.39750 | 0.89750 |
| 2416 | C3 | C | 0.50000 | 0.14750 | 0.64750 |
| 2417 | C3 | C | 0.75000 | 0.39750 | 0.64750 |
| 2418 | C3 | C | 0.75000 | 0.14750 | 0.89750 |
| 2419 | C3 | C | 0.50000 | 0.39750 | 0.60250 |
| 2420 | C3 | C | 0.50000 | 0.14750 | 0.85250 |
| 2421 | C3 | C | 0.75000 | 0.39750 | 0.85250 |
| 2422 | C3 | C | 0.75000 | 0.14750 | 0.60250 |
| 2423 | H1 | H | 0.63926 | 0.49997 | 0.56655 |
| 2424 | H1 | H | 0.63926 | 0.24997 | 0.81655 |
| 2425 | H1 | H | 0.88926 | 0.49997 | 0.81655 |
| 2426 | H1 | H | 0.88926 | 0.24997 | 0.56655 |
| 2427 | H1 | H | 0.86074 | 0.00003 | 0.56655 |
| 2428 | H1 | H | 0.86074 | 0.25004 | 0.81655 |
| 2429 | H1 | H | 0.61074 | 0.00003 | 0.81655 |
| 2430 | H1 | H | 0.61074 | 0.25004 | 0.56655 |

| | | | | | |
|---|---|---|---|---|---|
| 2431 | H1 | H | 0.86074 | 0.49996 | 0.93345 |
| 2432 | H1 | H | 0.86074 | 0.24997 | 0.68345 |
| 2433 | H1 | H | 0.61074 | 0.49997 | 0.68345 |
| 2434 | H1 | H | 0.61074 | 0.24997 | 0.93346 |
| 2435 | H1 | H | 0.63926 | 0.00003 | 0.93345 |
| 2436 | H1 | H | 0.63926 | 0.25003 | 0.68346 |
| 2437 | H1 | H | 0.88926 | 0.00003 | 0.68345 |
| 2438 | H1 | H | 0.88926 | 0.25004 | 0.93346 |
| 2439 | H1 | H | 0.56655 | 0.13926 | 0.99996 |
| 2440 | H1 | H | 0.56655 | 0.38926 | 0.74997 |
| 2441 | H1 | H | 0.81655 | 0.13926 | 0.74996 |
| 2442 | H1 | H | 0.81654 | 0.38926 | 0.99997 |
| 2443 | H1 | H | 0.56655 | 0.36074 | 0.50003 |
| 2444 | H1 | H | 0.56655 | 0.11074 | 0.75004 |
| 2445 | H1 | H | 0.81655 | 0.36074 | 0.75003 |
| 2446 | H1 | H | 0.93345 | 0.36074 | 0.99996 |
| 2447 | H1 | H | 0.93345 | 0.11074 | 0.74997 |
| 2448 | H1 | H | 0.68345 | 0.36074 | 0.74996 |
| 2449 | H1 | H | 0.68345 | 0.11074 | 0.99997 |
| 2450 | H1 | H | 0.93345 | 0.38926 | 0.75004 |
| 2451 | H1 | H | 0.68345 | 0.13926 | 0.75003 |
| 2452 | H1 | H | 0.68345 | 0.38926 | 0.50004 |
| 2453 | H1 | H | 0.99997 | 0.06655 | 0.63926 |
| 2454 | H1 | H | 0.99996 | 0.31655 | 0.88926 |
| 2455 | H1 | H | 0.74997 | 0.06655 | 0.88926 |
| 2456 | H1 | H | 0.74996 | 0.31655 | 0.63926 |
| 2457 | H1 | H | 0.50003 | 0.06655 | 0.86074 |
| 2458 | H1 | H | 0.50003 | 0.31655 | 0.61074 |
| 2459 | H1 | H | 0.75003 | 0.06655 | 0.61074 |
| 2460 | H1 | H | 0.75004 | 0.31655 | 0.86074 |
| 2461 | H1 | H | 0.99997 | 0.43345 | 0.86074 |
| 2462 | H1 | H | 0.99997 | 0.18345 | 0.61074 |
| 2463 | H1 | H | 0.74997 | 0.43345 | 0.61074 |
| 2464 | H1 | H | 0.74997 | 0.18345 | 0.86074 |
| 2465 | H1 | H | 0.50003 | 0.43345 | 0.63926 |
| 2466 | H1 | H | 0.50003 | 0.18345 | 0.88926 |
| 2467 | H1 | H | 0.75004 | 0.43345 | 0.88926 |
| 2468 | H1 | H | 0.75003 | 0.18346 | 0.63926 |
| 2469 | H1 | H | 0.50003 | 0.13926 | 0.93345 |
| 2470 | H1 | H | 0.50003 | 0.38926 | 0.68345 |
| 2471 | H1 | H | 0.75003 | 0.13926 | 0.68345 |
| 2472 | H1 | H | 0.75004 | 0.38926 | 0.93345 |
| 2473 | H1 | H | 0.99996 | 0.36074 | 0.93345 |
| 2474 | H1 | H | 0.99997 | 0.11074 | 0.68345 |
| 2475 | H1 | H | 0.74997 | 0.36074 | 0.68345 |
| 2476 | H1 | H | 0.74997 | 0.11074 | 0.93345 |
| 2477 | H1 | H | 0.50003 | 0.36074 | 0.56655 |
| 2478 | H1 | H | 0.50003 | 0.11074 | 0.81655 |
| 2479 | H1 | H | 0.75004 | 0.36074 | 0.81655 |
| 2480 | H1 | H | 0.75003 | 0.11074 | 0.56654 |
| 2481 | H1 | H | 0.99997 | 0.13926 | 0.56655 |
| 2482 | H1 | H | 0.99997 | 0.38926 | 0.81655 |
| 2483 | H1 | H | 0.74997 | 0.13926 | 0.81655 |
| 2484 | H1 | H | 0.74997 | 0.38926 | 0.56654 |

| | | | | | |
|---|---|---|---|---|---|
| 2485 | H1 | H | 0.63926 | 0.06655 | 0.99997 |
| 2486 | H1 | H | 0.63926 | 0.31655 | 0.74996 |
| 2487 | H1 | H | 0.88926 | 0.06655 | 0.74997 |
| 2488 | H1 | H | 0.88926 | 0.31655 | 0.99996 |
| 2489 | H1 | H | 0.86074 | 0.31655 | 0.75003 |
| 2490 | H1 | H | 0.61074 | 0.06655 | 0.75003 |
| 2491 | H1 | H | 0.61074 | 0.31655 | 0.50003 |
| 2492 | H1 | H | 0.86074 | 0.43345 | 0.99997 |
| 2493 | H1 | H | 0.86074 | 0.18345 | 0.74997 |
| 2494 | H1 | H | 0.61074 | 0.43345 | 0.74997 |
| 2495 | H1 | H | 0.61074 | 0.18345 | 0.99996 |
| 2496 | H1 | H | 0.63926 | 0.43345 | 0.50004 |
| 2497 | H1 | H | 0.63926 | 0.18346 | 0.75003 |
| 2498 | H1 | H | 0.88926 | 0.43345 | 0.75004 |
| 2499 | H1 | H | 0.56655 | 0.00003 | 0.86074 |
| 2500 | H1 | H | 0.56655 | 0.25004 | 0.61074 |
| 2501 | H1 | H | 0.81655 | 0.00003 | 0.61074 |
| 2502 | H1 | H | 0.81655 | 0.25004 | 0.86074 |
| 2503 | H1 | H | 0.56655 | 0.49996 | 0.63926 |
| 2504 | H1 | H | 0.56655 | 0.24997 | 0.88926 |
| 2505 | H1 | H | 0.81655 | 0.49996 | 0.88926 |
| 2506 | H1 | H | 0.81655 | 0.24997 | 0.63926 |
| 2507 | H1 | H | 0.93345 | 0.00003 | 0.63926 |
| 2508 | H1 | H | 0.93345 | 0.25004 | 0.88926 |
| 2509 | H1 | H | 0.68345 | 0.00003 | 0.88926 |
| 2510 | H1 | H | 0.68345 | 0.25003 | 0.63926 |
| 2511 | H1 | H | 0.93345 | 0.49996 | 0.86074 |
| 2512 | H1 | H | 0.93345 | 0.24997 | 0.61074 |
| 2513 | H1 | H | 0.68345 | 0.49996 | 0.61074 |
| 2514 | H1 | H | 0.68345 | 0.24997 | 0.86074 |
| 2515 | H2 | H | 0.70825 | 0.29176 | 0.95824 |
| 2516 | H2 | H | 0.79174 | 0.20825 | 0.95823 |
| 2517 | H2 | H | 0.70827 | 0.20823 | 0.54177 |
| 2518 | H2 | H | 0.79179 | 0.29168 | 0.54178 |
| 2519 | H2 | H | 0.95823 | 0.29173 | 0.70823 |
| 2520 | H2 | H | 0.95824 | 0.20825 | 0.79176 |
| 2521 | H2 | H | 0.54174 | 0.29176 | 0.79177 |
| 2522 | H2 | H | 0.54173 | 0.20823 | 0.70823 |
| 2523 | H2 | H | 0.79175 | 0.45824 | 0.70824 |
| 2524 | H2 | H | 0.70824 | 0.45824 | 0.79176 |
| 2525 | H2 | H | 0.70823 | 0.04172 | 0.70822 |
| 2526 | H2 | H | 0.79177 | 0.04173 | 0.79178 |
| 2527 | H2 | H | 0.54178 | 0.04177 | 0.54173 |
| 2528 | H2 | H | 0.95823 | 0.45824 | 0.54174 |
| 2529 | H2 | H | 0.95823 | 0.04176 | 0.95825 |
| 2530 | H2 | H | 0.54176 | 0.45824 | 0.95824 |
| 2531 | O1 | O | 0.58524 | 0.00000 | 0.54765 |
| 2532 | O1 | O | 0.58524 | 0.25000 | 0.79765 |
| 2533 | O1 | O | 0.83524 | 0.00000 | 0.79765 |
| 2534 | O1 | O | 0.83524 | 0.25000 | 0.54765 |
| 2535 | O1 | O | 0.91476 | 0.00000 | 0.54765 |
| 2536 | O1 | O | 0.91476 | 0.25000 | 0.79765 |
| 2537 | O1 | O | 0.66476 | 0.00000 | 0.79765 |
| 2538 | O1 | O | 0.66476 | 0.25000 | 0.54765 |

| | | | | | |
|---|---|---|---|---|---|
| 2539 | O1 | O | 0.91476 | 0.00000 | 0.95235 |
| 2540 | O1 | O | 0.91476 | 0.25000 | 0.70235 |
| 2541 | O1 | O | 0.66476 | 0.00000 | 0.70235 |
| 2542 | O1 | O | 0.66476 | 0.25000 | 0.95235 |
| 2543 | O1 | O | 0.58524 | 0.00000 | 0.95235 |
| 2544 | O1 | O | 0.58524 | 0.25000 | 0.70235 |
| 2545 | O1 | O | 0.83524 | 0.00000 | 0.70235 |
| 2546 | O1 | O | 0.83524 | 0.25000 | 0.95235 |
| 2547 | O1 | O | 0.54765 | 0.08524 | 0.50000 |
| 2548 | O1 | O | 0.54765 | 0.33524 | 0.75000 |
| 2549 | O1 | O | 0.79765 | 0.08524 | 0.75000 |
| 2550 | O1 | O | 0.79765 | 0.33524 | 0.50000 |
| 2551 | O1 | O | 0.54765 | 0.41476 | 0.50000 |
| 2552 | O1 | O | 0.54765 | 0.16476 | 0.75000 |
| 2553 | O1 | O | 0.79765 | 0.41476 | 0.75000 |
| 2554 | O3 | O | 0.79765 | 0.16476 | 0.50000 |
| 2555 | O1 | O | 0.95235 | 0.41476 | 0.50000 |
| 2556 | O1 | O | 0.95235 | 0.16476 | 0.75000 |
| 2557 | O1 | O | 0.70235 | 0.41476 | 0.75000 |
| 2558 | O1 | O | 0.70235 | 0.16476 | 0.50000 |
| 2559 | O3 | O | 0.95235 | 0.08524 | 0.50000 |
| 2560 | O1 | O | 0.95235 | 0.33524 | 0.75000 |
| 2561 | O1 | O | 0.70235 | 0.08524 | 0.75000 |
| 2562 | O1 | O | 0.70235 | 0.33524 | 0.50000 |
| 2563 | O1 | O | 0.50000 | 0.04765 | 0.58524 |
| 2564 | O1 | O | 0.50000 | 0.29765 | 0.83524 |
| 2565 | O1 | O | 0.75000 | 0.04765 | 0.83524 |
| 2566 | O1 | O | 0.75000 | 0.29765 | 0.58524 |
| 2567 | O1 | O | 0.50000 | 0.04765 | 0.91476 |
| 2568 | O1 | O | 0.50000 | 0.29765 | 0.66476 |
| 2569 | O1 | O | 0.75000 | 0.04765 | 0.66476 |
| 2570 | O1 | O | 0.75000 | 0.29765 | 0.91476 |
| 2571 | O1 | O | 0.50000 | 0.45235 | 0.91476 |
| 2572 | O1 | O | 0.50000 | 0.20235 | 0.66476 |
| 2573 | O1 | O | 0.75000 | 0.45235 | 0.66476 |
| 2574 | O1 | O | 0.75000 | 0.20235 | 0.91476 |
| 2575 | O1 | O | 0.50000 | 0.45235 | 0.58524 |
| 2576 | O1 | O | 0.50000 | 0.20235 | 0.83524 |
| 2577 | O1 | O | 0.75000 | 0.45235 | 0.83524 |
| 2578 | O1 | O | 0.75000 | 0.20235 | 0.58524 |
| 2579 | O1 | O | 0.50000 | 0.08524 | 0.95235 |
| 2580 | O1 | O | 0.50000 | 0.33524 | 0.70235 |
| 2581 | O1 | O | 0.75000 | 0.08524 | 0.70235 |
| 2582 | O1 | O | 0.75000 | 0.33524 | 0.95235 |
| 2583 | O1 | O | 0.50000 | 0.41476 | 0.95235 |
| 2584 | O1 | O | 0.50000 | 0.16476 | 0.70235 |
| 2585 | O1 | O | 0.75000 | 0.41476 | 0.70235 |
| 2586 | O1 | O | 0.75000 | 0.16476 | 0.95235 |
| 2587 | O1 | O | 0.50000 | 0.41476 | 0.54765 |
| 2588 | O1 | O | 0.50000 | 0.16476 | 0.79765 |
| 2589 | O1 | O | 0.75000 | 0.41476 | 0.79765 |
| 2590 | O1 | O | 0.75000 | 0.16476 | 0.54765 |
| 2591 | O1 | O | 0.50000 | 0.08524 | 0.54765 |
| 2592 | O1 | O | 0.50000 | 0.33524 | 0.79765 |

| | | | | | |
|---|---|---|---|---|---|
| 2593 | O1 | O | 0.75000 | 0.08524 | 0.79765 |
| 2594 | O1 | O | 0.75000 | 0.33524 | 0.54765 |
| 2595 | O1 | O | 0.58524 | 0.04765 | 0.50000 |
| 2596 | O1 | O | 0.58524 | 0.29765 | 0.75000 |
| 2597 | O1 | O | 0.83524 | 0.04765 | 0.75000 |
| 2598 | O1 | O | 0.83524 | 0.29765 | 0.50000 |
| 2599 | O3 | O | 0.91476 | 0.04765 | 0.50000 |
| 2600 | O1 | O | 0.91476 | 0.29765 | 0.75000 |
| 2601 | O1 | O | 0.66476 | 0.04765 | 0.75000 |
| 2602 | O1 | O | 0.66476 | 0.29765 | 0.50000 |
| 2603 | O1 | O | 0.91476 | 0.45235 | 0.50000 |
| 2604 | O1 | O | 0.91476 | 0.20235 | 0.75000 |
| 2605 | O1 | O | 0.66476 | 0.45235 | 0.75000 |
| 2606 | O1 | O | 0.66476 | 0.20235 | 0.50000 |
| 2607 | O1 | O | 0.58524 | 0.45235 | 0.50000 |
| 2608 | O1 | O | 0.58524 | 0.20235 | 0.75000 |
| 2609 | O1 | O | 0.83524 | 0.45235 | 0.75000 |
| 2610 | O3 | O | 0.83524 | 0.20235 | 0.50000 |
| 2611 | O1 | O | 0.54765 | 0.00000 | 0.91476 |
| 2612 | O1 | O | 0.54765 | 0.25000 | 0.66476 |
| 2613 | O1 | O | 0.79765 | 0.00000 | 0.66476 |
| 2614 | O1 | O | 0.79765 | 0.25000 | 0.91476 |
| 2615 | O1 | O | 0.54765 | 0.00000 | 0.58524 |
| 2616 | O1 | O | 0.54765 | 0.25000 | 0.83524 |
| 2617 | O1 | O | 0.79765 | 0.00000 | 0.83524 |
| 2618 | O1 | O | 0.79765 | 0.25000 | 0.58524 |
| 2619 | O1 | O | 0.95235 | 0.00000 | 0.58524 |
| 2620 | O1 | O | 0.95235 | 0.25000 | 0.83524 |
| 2621 | O1 | O | 0.70235 | 0.00000 | 0.83524 |
| 2622 | O1 | O | 0.70235 | 0.25000 | 0.58524 |
| 2623 | O1 | O | 0.95235 | 0.00000 | 0.91476 |
| 2624 | O1 | O | 0.95235 | 0.25000 | 0.66476 |
| 2625 | O1 | O | 0.70235 | 0.00000 | 0.66476 |
| 2626 | O1 | O | 0.70235 | 0.25000 | 0.91476 |
| 2627 | O2 | O | 0.52805 | 0.47195 | 0.97195 |
| 2628 | O2 | O | 0.52805 | 0.22195 | 0.72195 |
| 2629 | O2 | O | 0.77805 | 0.47195 | 0.72195 |
| 2630 | O2 | O | 0.77805 | 0.22195 | 0.97195 |
| 2631 | O2 | O | 0.97195 | 0.02805 | 0.97195 |
| 2632 | O2 | O | 0.97195 | 0.27805 | 0.72195 |
| 2633 | O2 | O | 0.72195 | 0.02805 | 0.72195 |
| 2634 | O2 | O | 0.72195 | 0.27805 | 0.97195 |
| 2635 | O2 | O | 0.97195 | 0.47195 | 0.52805 |
| 2636 | O2 | O | 0.97195 | 0.22195 | 0.77805 |
| 2637 | O2 | O | 0.72195 | 0.47195 | 0.77805 |
| 2638 | O2 | O | 0.72195 | 0.22195 | 0.52805 |
| 2639 | O2 | O | 0.52805 | 0.02805 | 0.52805 |
| 2640 | O2 | O | 0.52805 | 0.27805 | 0.77805 |
| 2641 | O2 | O | 0.77805 | 0.02805 | 0.77805 |
| 2642 | O2 | O | 0.77805 | 0.27805 | 0.52805 |
| 2643 | O1 | O | 0.97195 | 0.02805 | 0.52805 |
| 2644 | O1 | O | 0.97195 | 0.27805 | 0.77805 |
| 2645 | O1 | O | 0.72195 | 0.02805 | 0.77805 |
| 2646 | O1 | O | 0.72195 | 0.27805 | 0.52805 |

| | | | | | |
|---|---|---|---|---|---|
| 2647 | O1 | O | 0.52805 | 0.47195 | 0.52805 |
| 2648 | O1 | O | 0.52805 | 0.22195 | 0.77805 |
| 2649 | O1 | O | 0.77805 | 0.47195 | 0.77805 |
| 2650 | O1 | O | 0.77805 | 0.22195 | 0.52805 |
| 2651 | O1 | O | 0.97195 | 0.47195 | 0.97195 |
| 2652 | O1 | O | 0.97195 | 0.22195 | 0.72195 |
| 2653 | O1 | O | 0.72195 | 0.47195 | 0.72195 |
| 2654 | O1 | O | 0.72195 | 0.22195 | 0.97195 |
| 2655 | O1 | O | 0.52805 | 0.02805 | 0.97195 |
| 2656 | O1 | O | 0.52805 | 0.27805 | 0.72195 |
| 2657 | O1 | O | 0.77805 | 0.02805 | 0.72195 |
| 2658 | O1 | O | 0.77805 | 0.27805 | 0.97195 |
| 2659 | Zr1 | Zr | 0.55994 | 0.00000 | 0.50000 |
| 2660 | Zr1 | Zr | 0.55994 | 0.25000 | 0.75000 |
| 2661 | Zr1 | Zr | 0.80994 | 0.00000 | 0.75000 |
| 2662 | Zr1 | Zr | 0.80994 | 0.25000 | 0.50000 |
| 2663 | Zr1 | Zr | 0.94006 | 0.00000 | 0.50000 |
| 2664 | Zr1 | Zr | 0.94006 | 0.25000 | 0.75000 |
| 2665 | Zr1 | Zr | 0.69006 | 0.00000 | 0.75000 |
| 2666 | Zr1 | Zr | 0.69006 | 0.25000 | 0.50000 |
| 2667 | Zr1 | Zr | 0.50000 | 0.05994 | 0.50000 |
| 2668 | Zr1 | Zr | 0.50000 | 0.30994 | 0.75000 |
| 2669 | Zr1 | Zr | 0.75000 | 0.05994 | 0.75000 |
| 2670 | Zr1 | Zr | 0.75000 | 0.30994 | 0.50000 |
| 2671 | Zr1 | Zr | 0.50000 | 0.44006 | 0.50000 |
| 2672 | Zr1 | Zr | 0.50000 | 0.19006 | 0.75000 |
| 2673 | Zr1 | Zr | 0.75000 | 0.44006 | 0.75000 |
| 2674 | Zr1 | Zr | 0.75000 | 0.19006 | 0.50000 |
| 2675 | Zr1 | Zr | 0.50000 | 0.00000 | 0.55994 |
| 2676 | Zr1 | Zr | 0.50000 | 0.25000 | 0.80994 |
| 2677 | Zr1 | Zr | 0.75000 | 0.00000 | 0.80994 |
| 2678 | Zr1 | Zr | 0.75000 | 0.25000 | 0.55994 |
| 2679 | Zr1 | Zr | 0.50000 | 0.00000 | 0.94006 |
| 2680 | Zr1 | Zr | 0.50000 | 0.25000 | 0.69006 |
| 2681 | Zr1 | Zr | 0.75000 | 0.00000 | 0.69006 |
| 2682 | Zr1 | Zr | 0.75000 | 0.25000 | 0.94006 |
| 2683 | C2 | C | 0.63375 | 0.50000 | 0.09220 |
| 2684 | C2 | C | 0.63375 | 0.75000 | 0.34220 |
| 2685 | C2 | C | 0.88375 | 0.50000 | 0.34220 |
| 2686 | C2 | C | 0.88375 | 0.75000 | 0.09220 |
| 2687 | C2 | C | 0.86625 | 0.50000 | 0.09220 |
| 2688 | C2 | C | 0.86625 | 0.75000 | 0.34220 |
| 2689 | C2 | C | 0.61625 | 0.50000 | 0.34220 |
| 2690 | C2 | C | 0.61625 | 0.75000 | 0.09220 |
| 2691 | C2 | C | 0.86625 | 0.50000 | 0.40780 |
| 2692 | C2 | C | 0.86625 | 0.75000 | 0.15780 |
| 2693 | C2 | C | 0.61625 | 0.50000 | 0.15780 |
| 2694 | C2 | C | 0.61625 | 0.75000 | 0.40780 |
| 2695 | C2 | C | 0.63375 | 0.50000 | 0.40780 |
| 2696 | C2 | C | 0.63375 | 0.75000 | 0.15780 |
| 2697 | C2 | C | 0.88375 | 0.50000 | 0.15780 |
| 2698 | C2 | C | 0.88375 | 0.75000 | 0.40780 |
| 2699 | C2 | C | 0.59220 | 0.63375 | 0.00000 |
| 2700 | C2 | C | 0.84220 | 0.63375 | 0.25000 |

| | | | | | |
|---|---|---|---|---|---|
| 2701 | C2 | C | 0.84220 | 0.88375 | 0.00000 |
| 2702 | C2 | C | 0.59220 | 0.86625 | 0.00000 |
| 2703 | C2 | C | 0.59220 | 0.61625 | 0.25000 |
| 2704 | C2 | C | 0.84220 | 0.86625 | 0.25000 |
| 2705 | C2 | C | 0.84220 | 0.61625 | 0.00000 |
| 2706 | C2 | C | 0.90780 | 0.86625 | 0.00000 |
| 2707 | C2 | C | 0.90780 | 0.61625 | 0.25000 |
| 2708 | C2 | C | 0.65780 | 0.61625 | 0.00000 |
| 2709 | C2 | C | 0.90780 | 0.63375 | 0.00000 |
| 2710 | C2 | C | 0.90780 | 0.88375 | 0.25000 |
| 2711 | C2 | C | 0.65780 | 0.63375 | 0.25000 |
| 2712 | C2 | C | 0.65780 | 0.88375 | 0.00000 |
| 2713 | C2 | C | 0.50000 | 0.59220 | 0.13375 |
| 2714 | C2 | C | 0.50000 | 0.84220 | 0.38375 |
| 2715 | C2 | C | 0.75000 | 0.59220 | 0.38375 |
| 2716 | C2 | C | 0.75000 | 0.84220 | 0.13375 |
| 2717 | C2 | C | 0.50000 | 0.59220 | 0.36625 |
| 2718 | C2 | C | 0.50000 | 0.84220 | 0.11625 |
| 2719 | C2 | C | 0.75000 | 0.59220 | 0.11625 |
| 2720 | C2 | C | 0.75000 | 0.84220 | 0.36625 |
| 2721 | C2 | C | 0.50000 | 0.90780 | 0.36625 |
| 2722 | C2 | C | 0.50000 | 0.65780 | 0.11625 |
| 2723 | C2 | C | 0.75000 | 0.90780 | 0.11625 |
| 2724 | C2 | C | 0.75000 | 0.65780 | 0.36625 |
| 2725 | C2 | C | 0.50000 | 0.90780 | 0.13375 |
| 2726 | C2 | C | 0.50000 | 0.65780 | 0.38375 |
| 2727 | C2 | C | 0.75000 | 0.90780 | 0.38375 |
| 2728 | C2 | C | 0.75000 | 0.65780 | 0.13375 |
| 2729 | C2 | C | 0.50000 | 0.63375 | 0.40780 |
| 2730 | C2 | C | 0.50000 | 0.88375 | 0.15780 |
| 2731 | C2 | C | 0.75000 | 0.63375 | 0.15780 |
| 2732 | C2 | C | 0.75000 | 0.88375 | 0.40780 |
| 2733 | C2 | C | 0.50000 | 0.86625 | 0.40780 |
| 2734 | C2 | C | 0.50000 | 0.61625 | 0.15780 |
| 2735 | C2 | C | 0.75000 | 0.86625 | 0.15780 |
| 2736 | C2 | C | 0.75000 | 0.61625 | 0.40780 |
| 2737 | C2 | C | 0.50000 | 0.86625 | 0.09220 |
| 2738 | C2 | C | 0.50000 | 0.61625 | 0.34220 |
| 2739 | C2 | C | 0.75000 | 0.86625 | 0.34220 |
| 2740 | C2 | C | 0.75000 | 0.61625 | 0.09220 |
| 2741 | C2 | C | 0.50000 | 0.63375 | 0.09220 |
| 2742 | C2 | C | 0.50000 | 0.88375 | 0.34220 |
| 2743 | C2 | C | 0.75000 | 0.63375 | 0.34220 |
| 2744 | C2 | C | 0.75000 | 0.88375 | 0.09220 |
| 2745 | C2 | C | 0.63375 | 0.59220 | 0.00000 |
| 2746 | C2 | C | 0.88375 | 0.59220 | 0.25000 |
| 2747 | C2 | C | 0.88375 | 0.84220 | 0.00000 |
| 2748 | C2 | C | 0.86625 | 0.59220 | 0.00000 |
| 2749 | C2 | C | 0.86625 | 0.84220 | 0.25000 |
| 2750 | C2 | C | 0.61625 | 0.59220 | 0.25000 |
| 2751 | C2 | C | 0.61625 | 0.84220 | 0.00000 |
| 2752 | C2 | C | 0.86625 | 0.90780 | 0.00000 |
| 2753 | C2 | C | 0.86625 | 0.65780 | 0.25000 |
| 2754 | C2 | C | 0.61625 | 0.65780 | 0.00000 |

| | | | | | |
|---|---|---|---|---|---|
| 2755 | C2 | C | 0.63375 | 0.90780 | 0.00000 |
| 2756 | C2 | C | 0.63375 | 0.65780 | 0.25000 |
| 2757 | C2 | C | 0.88375 | 0.90780 | 0.25000 |
| 2758 | C2 | C | 0.88375 | 0.65780 | 0.00000 |
| 2759 | C2 | C | 0.59220 | 0.50000 | 0.36625 |
| 2760 | C2 | C | 0.59220 | 0.75000 | 0.11625 |
| 2761 | C2 | C | 0.84220 | 0.50000 | 0.11625 |
| 2762 | C2 | C | 0.84220 | 0.75000 | 0.36625 |
| 2763 | C2 | C | 0.59220 | 0.50000 | 0.13375 |
| 2764 | C2 | C | 0.59220 | 0.75000 | 0.38375 |
| 2765 | C2 | C | 0.84220 | 0.50000 | 0.38375 |
| 2766 | C2 | C | 0.84220 | 0.75000 | 0.13375 |
| 2767 | C2 | C | 0.90780 | 0.50000 | 0.13375 |
| 2768 | C2 | C | 0.90780 | 0.75000 | 0.38375 |
| 2769 | C2 | C | 0.65780 | 0.50000 | 0.38375 |
| 2770 | C2 | C | 0.65780 | 0.75000 | 0.13375 |
| 2771 | C2 | C | 0.90780 | 0.50000 | 0.36625 |
| 2772 | C2 | C | 0.90780 | 0.75000 | 0.11625 |
| 2773 | C2 | C | 0.65780 | 0.50000 | 0.11625 |
| 2774 | C2 | C | 0.65780 | 0.75000 | 0.36625 |
| 2775 | C1 | C | 0.57680 | 0.50000 | 0.42320 |
| 2776 | C1 | C | 0.57680 | 0.75000 | 0.17320 |
| 2777 | C1 | C | 0.82680 | 0.50000 | 0.17320 |
| 2778 | C1 | C | 0.82680 | 0.75000 | 0.42320 |
| 2779 | C1 | C | 0.92320 | 0.50000 | 0.42320 |
| 2780 | C1 | C | 0.92320 | 0.75000 | 0.17320 |
| 2781 | C1 | C | 0.67320 | 0.50000 | 0.17320 |
| 2782 | C1 | C | 0.67320 | 0.75000 | 0.42320 |
| 2783 | C1 | C | 0.92320 | 0.50000 | 0.07680 |
| 2784 | C1 | C | 0.92320 | 0.75000 | 0.32680 |
| 2785 | C1 | C | 0.67320 | 0.50000 | 0.32680 |
| 2786 | C1 | C | 0.67320 | 0.75000 | 0.07680 |
| 2787 | C1 | C | 0.57680 | 0.50000 | 0.07680 |
| 2788 | C1 | C | 0.57680 | 0.75000 | 0.32680 |
| 2789 | C1 | C | 0.82680 | 0.50000 | 0.32680 |
| 2790 | C1 | C | 0.82680 | 0.75000 | 0.07680 |
| 2791 | C1 | C | 0.92320 | 0.57680 | 0.00000 |
| 2792 | C1 | C | 0.92320 | 0.82680 | 0.25000 |
| 2793 | C1 | C | 0.67320 | 0.57680 | 0.25000 |
| 2794 | C1 | C | 0.67320 | 0.82680 | 0.00000 |
| 2795 | C1 | C | 0.92320 | 0.92320 | 0.00000 |
| 2796 | C1 | C | 0.92320 | 0.67320 | 0.25000 |
| 2797 | C1 | C | 0.67320 | 0.67320 | 0.00000 |
| 2798 | C1 | C | 0.57680 | 0.92320 | 0.00000 |
| 2799 | C1 | C | 0.57680 | 0.67320 | 0.25000 |
| 2800 | C1 | C | 0.82680 | 0.92320 | 0.25000 |
| 2801 | C1 | C | 0.82680 | 0.67320 | 0.00000 |
| 2802 | C1 | C | 0.57680 | 0.57680 | 0.00000 |
| 2803 | C1 | C | 0.82680 | 0.57680 | 0.25000 |
| 2804 | C1 | C | 0.82680 | 0.82680 | 0.00000 |
| 2805 | C1 | C | 0.50000 | 0.92320 | 0.07680 |
| 2806 | C1 | C | 0.50000 | 0.67320 | 0.32680 |
| 2807 | C1 | C | 0.75000 | 0.92320 | 0.32680 |
| 2808 | C1 | C | 0.75000 | 0.67320 | 0.07680 |

| | | | | | |
|---|---|---|---|---|---|
| 2809 | C1 | C | 0.50000 | 0.92320 | 0.42320 |
| 2810 | C1 | C | 0.50000 | 0.67320 | 0.17320 |
| 2811 | C1 | C | 0.75000 | 0.92320 | 0.17320 |
| 2812 | C1 | C | 0.75000 | 0.67320 | 0.42320 |
| 2813 | C1 | C | 0.50000 | 0.57680 | 0.42320 |
| 2814 | C1 | C | 0.50000 | 0.82680 | 0.17320 |
| 2815 | C1 | C | 0.75000 | 0.57680 | 0.17320 |
| 2816 | C1 | C | 0.75000 | 0.82680 | 0.42320 |
| 2817 | C1 | C | 0.50000 | 0.57680 | 0.07680 |
| 2818 | C1 | C | 0.50000 | 0.82680 | 0.32680 |
| 2819 | C1 | C | 0.75000 | 0.57680 | 0.32680 |
| 2820 | C1 | C | 0.75000 | 0.82680 | 0.07680 |
| 2821 | C3 | C | 0.60250 | 0.50000 | 0.10250 |
| 2822 | C3 | C | 0.60250 | 0.75000 | 0.35250 |
| 2823 | C3 | C | 0.85250 | 0.50000 | 0.35250 |
| 2824 | C3 | C | 0.85250 | 0.75000 | 0.10250 |
| 2825 | C3 | C | 0.89750 | 0.50000 | 0.10250 |
| 2826 | C3 | C | 0.89750 | 0.75000 | 0.35250 |
| 2827 | C3 | C | 0.64750 | 0.50000 | 0.35250 |
| 2828 | C3 | C | 0.64750 | 0.75000 | 0.10250 |
| 2829 | C3 | C | 0.89750 | 0.50000 | 0.39750 |
| 2830 | C3 | C | 0.89750 | 0.75000 | 0.14750 |
| 2831 | C3 | C | 0.64750 | 0.50000 | 0.14750 |
| 2832 | C3 | C | 0.64750 | 0.75000 | 0.39750 |
| 2833 | C3 | C | 0.60250 | 0.50000 | 0.39750 |
| 2834 | C3 | C | 0.60250 | 0.75000 | 0.14750 |
| 2835 | C3 | C | 0.85250 | 0.50000 | 0.14750 |
| 2836 | C3 | C | 0.85250 | 0.75000 | 0.39750 |
| 2837 | C3 | C | 0.60250 | 0.60250 | 0.00000 |
| 2838 | C3 | C | 0.85250 | 0.60250 | 0.25000 |
| 2839 | C3 | C | 0.85250 | 0.85250 | 0.00000 |
| 2840 | C3 | C | 0.60250 | 0.89750 | 0.00000 |
| 2841 | C3 | C | 0.60250 | 0.64750 | 0.25000 |
| 2842 | C3 | C | 0.85250 | 0.89750 | 0.25000 |
| 2843 | C3 | C | 0.85250 | 0.64750 | 0.00000 |
| 2844 | C3 | C | 0.89750 | 0.89750 | 0.00000 |
| 2845 | C3 | C | 0.89750 | 0.64750 | 0.25000 |
| 2846 | C3 | C | 0.64750 | 0.64750 | 0.00000 |
| 2847 | C3 | C | 0.89750 | 0.60250 | 0.00000 |
| 2848 | C3 | C | 0.89750 | 0.85250 | 0.25000 |
| 2849 | C3 | C | 0.64750 | 0.60250 | 0.25000 |
| 2850 | C3 | C | 0.64750 | 0.85250 | 0.00000 |
| 2851 | C3 | C | 0.50000 | 0.60250 | 0.10250 |
| 2852 | C3 | C | 0.50000 | 0.85250 | 0.35250 |
| 2853 | C3 | C | 0.75000 | 0.60250 | 0.35250 |
| 2854 | C3 | C | 0.75000 | 0.85250 | 0.10250 |
| 2855 | C3 | C | 0.50000 | 0.60250 | 0.39750 |
| 2856 | C3 | C | 0.50000 | 0.85250 | 0.14750 |
| 2857 | C3 | C | 0.75000 | 0.60250 | 0.14750 |
| 2858 | C3 | C | 0.75000 | 0.85250 | 0.39750 |
| 2859 | C3 | C | 0.50000 | 0.89750 | 0.39750 |
| 2860 | C3 | C | 0.50000 | 0.64750 | 0.14750 |
| 2861 | C3 | C | 0.75000 | 0.89750 | 0.14750 |
| 2862 | C3 | C | 0.75000 | 0.64750 | 0.39750 |

| | | | | | |
|---|---|---|---|---|---|
| 2863 | C3 | C | 0.50000 | 0.89750 | 0.10250 |
| 2864 | C3 | C | 0.50000 | 0.64750 | 0.35250 |
| 2865 | C3 | C | 0.75000 | 0.89750 | 0.35250 |
| 2866 | C3 | C | 0.75000 | 0.64750 | 0.10250 |
| 2867 | H1 | H | 0.63926 | 0.99997 | 0.06655 |
| 2868 | H1 | H | 0.63926 | 0.74997 | 0.31655 |
| 2869 | H1 | H | 0.88926 | 0.99997 | 0.31655 |
| 2870 | H1 | H | 0.88926 | 0.74997 | 0.06655 |
| 2871 | H1 | H | 0.86074 | 0.50003 | 0.06655 |
| 2872 | H1 | H | 0.86074 | 0.75004 | 0.31655 |
| 2873 | H1 | H | 0.61074 | 0.50003 | 0.31655 |
| 2874 | H1 | H | 0.61074 | 0.75004 | 0.06655 |
| 2875 | H1 | H | 0.86074 | 0.99996 | 0.43345 |
| 2876 | H1 | H | 0.86074 | 0.74997 | 0.18345 |
| 2877 | H1 | H | 0.61074 | 0.99997 | 0.18345 |
| 2878 | H1 | H | 0.61074 | 0.74997 | 0.43346 |
| 2879 | H1 | H | 0.63926 | 0.50003 | 0.43345 |
| 2880 | H1 | H | 0.63926 | 0.75003 | 0.18346 |
| 2881 | H1 | H | 0.88926 | 0.50003 | 0.18345 |
| 2882 | H1 | H | 0.88926 | 0.75004 | 0.43346 |
| 2883 | H1 | H | 0.56655 | 0.63926 | 0.49996 |
| 2884 | H1 | H | 0.81655 | 0.63926 | 0.24996 |
| 2885 | H1 | H | 0.81654 | 0.88926 | 0.49997 |
| 2886 | H1 | H | 0.56655 | 0.86074 | 0.00003 |
| 2887 | H1 | H | 0.56655 | 0.61074 | 0.25004 |
| 2888 | H1 | H | 0.81655 | 0.86074 | 0.25003 |
| 2889 | H1 | H | 0.81655 | 0.61074 | 0.00004 |
| 2890 | H1 | H | 0.93345 | 0.86074 | 0.49996 |
| 2891 | H1 | H | 0.93345 | 0.61074 | 0.24997 |
| 2892 | H1 | H | 0.68345 | 0.61074 | 0.49997 |
| 2893 | H1 | H | 0.93345 | 0.63926 | 0.00003 |
| 2894 | H1 | H | 0.93345 | 0.88926 | 0.25004 |
| 2895 | H1 | H | 0.68345 | 0.63926 | 0.25003 |
| 2896 | H1 | H | 0.68345 | 0.88926 | 0.00004 |
| 2897 | H1 | H | 0.99997 | 0.56655 | 0.13926 |
| 2898 | H1 | H | 0.99996 | 0.81655 | 0.38926 |
| 2899 | H1 | H | 0.74997 | 0.56655 | 0.38926 |
| 2900 | H1 | H | 0.74996 | 0.81655 | 0.13926 |
| 2901 | H1 | H | 0.50003 | 0.56655 | 0.36074 |
| 2902 | H1 | H | 0.50003 | 0.81655 | 0.11074 |
| 2903 | H1 | H | 0.75003 | 0.56655 | 0.11074 |
| 2904 | H1 | H | 0.75004 | 0.81655 | 0.36074 |
| 2905 | H1 | H | 0.99997 | 0.93345 | 0.36074 |
| 2906 | H1 | H | 0.99997 | 0.68345 | 0.11074 |
| 2907 | H1 | H | 0.74997 | 0.93345 | 0.11074 |
| 2908 | H1 | H | 0.74997 | 0.68345 | 0.36074 |
| 2909 | H1 | H | 0.50003 | 0.93345 | 0.13926 |
| 2910 | H1 | H | 0.50003 | 0.68345 | 0.38926 |
| 2911 | H1 | H | 0.75004 | 0.93345 | 0.38926 |
| 2912 | H1 | H | 0.75003 | 0.68346 | 0.13926 |
| 2913 | H1 | H | 0.50003 | 0.63926 | 0.43345 |
| 2914 | H1 | H | 0.50003 | 0.88926 | 0.18345 |
| 2915 | H1 | H | 0.75003 | 0.63926 | 0.18345 |
| 2916 | H1 | H | 0.75004 | 0.88926 | 0.43345 |

| | | | | | |
|---|---|---|---|---|---|
| 2917 | H1 | H | 0.99996 | 0.86074 | 0.43345 |
| 2918 | H1 | H | 0.99997 | 0.61074 | 0.18345 |
| 2919 | H1 | H | 0.74997 | 0.86074 | 0.18345 |
| 2920 | H1 | H | 0.74997 | 0.61074 | 0.43345 |
| 2921 | H1 | H | 0.50003 | 0.86074 | 0.06655 |
| 2922 | H1 | H | 0.50003 | 0.61074 | 0.31655 |
| 2923 | H1 | H | 0.75004 | 0.86074 | 0.31655 |
| 2924 | H1 | H | 0.75003 | 0.61074 | 0.06654 |
| 2925 | H1 | H | 0.99997 | 0.63926 | 0.06655 |
| 2926 | H1 | H | 0.99997 | 0.88926 | 0.31655 |
| 2927 | H1 | H | 0.74997 | 0.63926 | 0.31655 |
| 2928 | H1 | H | 0.74997 | 0.88926 | 0.06654 |
| 2929 | H1 | H | 0.63926 | 0.56655 | 0.49997 |
| 2930 | H1 | H | 0.88926 | 0.56655 | 0.24997 |
| 2931 | H1 | H | 0.88926 | 0.81655 | 0.49996 |
| 2932 | H1 | H | 0.86074 | 0.56655 | 0.00003 |
| 2933 | H1 | H | 0.86074 | 0.81655 | 0.25003 |
| 2934 | H1 | H | 0.61074 | 0.56655 | 0.25003 |
| 2935 | H1 | H | 0.61074 | 0.81655 | 0.00003 |
| 2936 | H1 | H | 0.86074 | 0.93345 | 0.49997 |
| 2937 | H1 | H | 0.86074 | 0.68345 | 0.24997 |
| 2938 | H1 | H | 0.61074 | 0.68345 | 0.49996 |
| 2939 | H1 | H | 0.63926 | 0.93345 | 0.00004 |
| 2940 | H1 | H | 0.63926 | 0.68346 | 0.25003 |
| 2941 | H1 | H | 0.88926 | 0.93345 | 0.25004 |
| 2942 | H1 | H | 0.88926 | 0.68346 | 0.00003 |
| 2943 | H1 | H | 0.56655 | 0.50003 | 0.36074 |
| 2944 | H1 | H | 0.56655 | 0.75004 | 0.11074 |
| 2945 | H1 | H | 0.81655 | 0.50003 | 0.11074 |
| 2946 | H1 | H | 0.81655 | 0.75004 | 0.36074 |
| 2947 | H1 | H | 0.56655 | 0.99996 | 0.13926 |
| 2948 | H1 | H | 0.56655 | 0.74997 | 0.38926 |
| 2949 | H1 | H | 0.81655 | 0.99996 | 0.38926 |
| 2950 | H1 | H | 0.81655 | 0.74997 | 0.13926 |
| 2951 | H1 | H | 0.93345 | 0.50003 | 0.13926 |
| 2952 | H1 | H | 0.93345 | 0.75004 | 0.38926 |
| 2953 | H1 | H | 0.68345 | 0.50003 | 0.38926 |
| 2954 | H1 | H | 0.68345 | 0.75003 | 0.13926 |
| 2955 | H1 | H | 0.93345 | 0.99996 | 0.36074 |
| 2956 | H1 | H | 0.93345 | 0.74997 | 0.11074 |
| 2957 | H1 | H | 0.68345 | 0.99996 | 0.11074 |
| 2958 | H1 | H | 0.68345 | 0.74997 | 0.36074 |
| 2959 | H2 | H | 0.70825 | 0.79176 | 0.45824 |
| 2960 | H2 | H | 0.79174 | 0.70825 | 0.45823 |
| 2961 | H2 | H | 0.70827 | 0.70823 | 0.04177 |
| 2962 | H2 | H | 0.79179 | 0.79168 | 0.04178 |
| 2963 | H2 | H | 0.95823 | 0.79173 | 0.20823 |
| 2964 | H2 | H | 0.95824 | 0.70825 | 0.29176 |
| 2965 | H2 | H | 0.54174 | 0.79176 | 0.29177 |
| 2966 | H2 | H | 0.54173 | 0.70823 | 0.20823 |
| 2967 | H2 | H | 0.79175 | 0.95824 | 0.20824 |
| 2968 | H2 | H | 0.70824 | 0.95824 | 0.29176 |
| 2969 | H2 | H | 0.70823 | 0.54172 | 0.20822 |
| 2970 | H2 | H | 0.79177 | 0.54173 | 0.29178 |

| | | | | | |
|---|---|---|---|---|---|
| 2971 | H2 | H | 0.54178 | 0.54177 | 0.04173 |
| 2972 | H2 | H | 0.95823 | 0.95824 | 0.04174 |
| 2973 | H2 | H | 0.95823 | 0.54176 | 0.45825 |
| 2974 | H2 | H | 0.54176 | 0.95824 | 0.45824 |
| 2975 | O1 | O | 0.58524 | 0.50000 | 0.04765 |
| 2976 | O1 | O | 0.58524 | 0.75000 | 0.29765 |
| 2977 | O1 | O | 0.83524 | 0.50000 | 0.29765 |
| 2978 | O1 | O | 0.83524 | 0.75000 | 0.04765 |
| 2979 | O1 | O | 0.91476 | 0.50000 | 0.04765 |
| 2980 | O1 | O | 0.91476 | 0.75000 | 0.29765 |
| 2981 | O1 | O | 0.66476 | 0.50000 | 0.29765 |
| 2982 | O1 | O | 0.66476 | 0.75000 | 0.04765 |
| 2983 | O1 | O | 0.91476 | 0.50000 | 0.45235 |
| 2984 | O1 | O | 0.91476 | 0.75000 | 0.20235 |
| 2985 | O1 | O | 0.66476 | 0.50000 | 0.20235 |
| 2986 | O1 | O | 0.66476 | 0.75000 | 0.45235 |
| 2987 | O1 | O | 0.58524 | 0.50000 | 0.45235 |
| 2988 | O1 | O | 0.58524 | 0.75000 | 0.20235 |
| 2989 | O1 | O | 0.83524 | 0.50000 | 0.20235 |
| 2990 | O1 | O | 0.83524 | 0.75000 | 0.45235 |
| 2991 | O1 | O | 0.54765 | 0.58524 | 0.00000 |
| 2992 | O3 | O | 0.54765 | 0.83524 | 0.25000 |
| 2993 | O1 | O | 0.79765 | 0.58524 | 0.25000 |
| 2994 | O1 | O | 0.79765 | 0.83524 | 0.00000 |
| 2995 | O1 | O | 0.54765 | 0.91476 | 0.00000 |
| 2996 | O1 | O | 0.54765 | 0.66476 | 0.25000 |
| 2997 | O1 | O | 0.79765 | 0.91476 | 0.25000 |
| 2998 | O1 | O | 0.79765 | 0.66476 | 0.00000 |
| 2999 | O1 | O | 0.95235 | 0.91476 | 0.00000 |
| 3000 | O1 | O | 0.95235 | 0.66476 | 0.25000 |
| 3001 | O3 | O | 0.70235 | 0.91476 | 0.25000 |
| 3002 | O1 | O | 0.70235 | 0.66476 | 0.00000 |
| 3003 | O1 | O | 0.95235 | 0.58524 | 0.00000 |
| 3004 | O1 | O | 0.95235 | 0.83524 | 0.25000 |
| 3005 | O1 | O | 0.70235 | 0.58524 | 0.25000 |
| 3006 | O1 | O | 0.70235 | 0.83524 | 0.00000 |
| 3007 | O1 | O | 0.50000 | 0.54765 | 0.08524 |
| 3008 | O1 | O | 0.50000 | 0.79765 | 0.33524 |
| 3009 | O1 | O | 0.75000 | 0.54765 | 0.33524 |
| 3010 | O1 | O | 0.75000 | 0.79765 | 0.08524 |
| 3011 | O1 | O | 0.50000 | 0.54765 | 0.41476 |
| 3012 | O1 | O | 0.50000 | 0.79765 | 0.16476 |
| 3013 | O1 | O | 0.75000 | 0.54765 | 0.16476 |
| 3014 | O1 | O | 0.75000 | 0.79765 | 0.41476 |
| 3015 | O1 | O | 0.50000 | 0.95235 | 0.41476 |
| 3016 | O1 | O | 0.50000 | 0.70235 | 0.16476 |
| 3017 | O1 | O | 0.75000 | 0.95235 | 0.16476 |
| 3018 | O1 | O | 0.75000 | 0.70235 | 0.41476 |
| 3019 | O1 | O | 0.50000 | 0.95235 | 0.08524 |
| 3020 | O1 | O | 0.50000 | 0.70235 | 0.33524 |
| 3021 | O1 | O | 0.75000 | 0.95235 | 0.33524 |
| 3022 | O1 | O | 0.75000 | 0.70235 | 0.08524 |
| 3023 | O1 | O | 0.50000 | 0.58524 | 0.45235 |
| 3024 | O1 | O | 0.50000 | 0.83524 | 0.20235 |

| | | | | | |
|---|---|---|---|---|---|
| 3025 | O1 | O | 0.75000 | 0.58524 | 0.20235 |
| 3026 | O1 | O | 0.75000 | 0.83524 | 0.45235 |
| 3027 | O1 | O | 0.50000 | 0.91476 | 0.45235 |
| 3028 | O1 | O | 0.50000 | 0.66476 | 0.20235 |
| 3029 | O1 | O | 0.75000 | 0.91476 | 0.20235 |
| 3030 | O1 | O | 0.75000 | 0.66476 | 0.45235 |
| 3031 | O1 | O | 0.50000 | 0.91476 | 0.04765 |
| 3032 | O1 | O | 0.50000 | 0.66476 | 0.29765 |
| 3033 | O1 | O | 0.75000 | 0.91476 | 0.29765 |
| 3034 | O1 | O | 0.75000 | 0.66476 | 0.04765 |
| 3035 | O1 | O | 0.50000 | 0.58524 | 0.04765 |
| 3036 | O1 | O | 0.50000 | 0.83524 | 0.29765 |
| 3037 | O1 | O | 0.75000 | 0.58524 | 0.29765 |
| 3038 | O1 | O | 0.75000 | 0.83524 | 0.04765 |
| 3039 | O1 | O | 0.58524 | 0.54765 | 0.00000 |
| 3040 | O3 | O | 0.58524 | 0.79765 | 0.25000 |
| 3041 | O1 | O | 0.83524 | 0.54765 | 0.25000 |
| 3042 | O1 | O | 0.83524 | 0.79765 | 0.00000 |
| 3043 | O1 | O | 0.91476 | 0.54765 | 0.00000 |
| 3044 | O1 | O | 0.91476 | 0.79765 | 0.25000 |
| 3045 | O1 | O | 0.66476 | 0.54765 | 0.25000 |
| 3046 | O1 | O | 0.66476 | 0.79765 | 0.00000 |
| 3047 | O1 | O | 0.91476 | 0.95235 | 0.00000 |
| 3048 | O1 | O | 0.91476 | 0.70235 | 0.25000 |
| 3049 | O3 | O | 0.66476 | 0.95235 | 0.25000 |
| 3050 | O1 | O | 0.66476 | 0.70235 | 0.00000 |
| 3051 | O1 | O | 0.58524 | 0.95235 | 0.00000 |
| 3052 | O1 | O | 0.58524 | 0.70235 | 0.25000 |
| 3053 | O1 | O | 0.83524 | 0.95235 | 0.25000 |
| 3054 | O1 | O | 0.83524 | 0.70235 | 0.00000 |
| 3055 | O1 | O | 0.54765 | 0.50000 | 0.41476 |
| 3056 | O1 | O | 0.54765 | 0.75000 | 0.16476 |
| 3057 | O1 | O | 0.79765 | 0.50000 | 0.16476 |
| 3058 | O1 | O | 0.79765 | 0.75000 | 0.41476 |
| 3059 | O1 | O | 0.54765 | 0.50000 | 0.08524 |
| 3060 | O1 | O | 0.54765 | 0.75000 | 0.33524 |
| 3061 | O1 | O | 0.79765 | 0.50000 | 0.33524 |
| 3062 | O1 | O | 0.79765 | 0.75000 | 0.08524 |
| 3063 | O1 | O | 0.95235 | 0.50000 | 0.08524 |
| 3064 | O1 | O | 0.95235 | 0.75000 | 0.33524 |
| 3065 | O1 | O | 0.70235 | 0.50000 | 0.33524 |
| 3066 | O1 | O | 0.70235 | 0.75000 | 0.08524 |
| 3067 | O1 | O | 0.95235 | 0.50000 | 0.41476 |
| 3068 | O1 | O | 0.95235 | 0.75000 | 0.16476 |
| 3069 | O1 | O | 0.70235 | 0.50000 | 0.16476 |
| 3070 | O1 | O | 0.70235 | 0.75000 | 0.41476 |
| 3071 | O2 | O | 0.52805 | 0.97195 | 0.47195 |
| 3072 | O2 | O | 0.52805 | 0.72195 | 0.22195 |
| 3073 | O2 | O | 0.77805 | 0.97195 | 0.22195 |
| 3074 | O2 | O | 0.77805 | 0.72195 | 0.47195 |
| 3075 | O2 | O | 0.97195 | 0.52805 | 0.47195 |
| 3076 | O2 | O | 0.97195 | 0.77805 | 0.22195 |
| 3077 | O2 | O | 0.72195 | 0.52805 | 0.22195 |
| 3078 | O2 | O | 0.72195 | 0.77805 | 0.47195 |

| | | | | | |
|---|---|---|---|---|---|
| 3079 | O2 | O | 0.97195 | 0.97195 | 0.02805 |
| 3080 | O2 | O | 0.97195 | 0.72195 | 0.27805 |
| 3081 | O2 | O | 0.72195 | 0.97195 | 0.27805 |
| 3082 | O2 | O | 0.72195 | 0.72195 | 0.02805 |
| 3083 | O2 | O | 0.52805 | 0.52805 | 0.02805 |
| 3084 | O2 | O | 0.52805 | 0.77805 | 0.27805 |
| 3085 | O2 | O | 0.77805 | 0.52805 | 0.27805 |
| 3086 | O2 | O | 0.77805 | 0.77805 | 0.02805 |
| 3087 | O1 | O | 0.97195 | 0.52805 | 0.02805 |
| 3088 | O1 | O | 0.97195 | 0.77805 | 0.27805 |
| 3089 | O1 | O | 0.72195 | 0.52805 | 0.27805 |
| 3090 | O1 | O | 0.72195 | 0.77805 | 0.02805 |
| 3091 | O1 | O | 0.52805 | 0.97195 | 0.02805 |
| 3092 | O1 | O | 0.52805 | 0.72195 | 0.27805 |
| 3093 | O1 | O | 0.77805 | 0.97195 | 0.27805 |
| 3094 | O1 | O | 0.77805 | 0.72195 | 0.02805 |
| 3095 | O1 | O | 0.97195 | 0.97195 | 0.47195 |
| 3096 | O1 | O | 0.97195 | 0.72195 | 0.22195 |
| 3097 | O1 | O | 0.72195 | 0.97195 | 0.22195 |
| 3098 | O1 | O | 0.72195 | 0.72195 | 0.47195 |
| 3099 | O1 | O | 0.52805 | 0.52805 | 0.47195 |
| 3100 | O1 | O | 0.52805 | 0.77805 | 0.22195 |
| 3101 | O1 | O | 0.77805 | 0.52805 | 0.22195 |
| 3102 | O1 | O | 0.77805 | 0.77805 | 0.47195 |
| 3103 | Zr1 | Zr | 0.55994 | 0.50000 | 0.00000 |
| 3104 | Zr1 | Zr | 0.55994 | 0.75000 | 0.25000 |
| 3105 | Zr1 | Zr | 0.80994 | 0.50000 | 0.25000 |
| 3106 | Zr1 | Zr | 0.80994 | 0.75000 | 0.00000 |
| 3107 | Zr1 | Zr | 0.94006 | 0.50000 | 0.00000 |
| 3108 | Zr1 | Zr | 0.94006 | 0.75000 | 0.25000 |
| 3109 | Zr1 | Zr | 0.69006 | 0.50000 | 0.25000 |
| 3110 | Zr1 | Zr | 0.69006 | 0.75000 | 0.00000 |
| 3111 | Zr1 | Zr | 0.50000 | 0.55994 | 0.00000 |
| 3112 | Zr1 | Zr | 0.50000 | 0.80994 | 0.25000 |
| 3113 | Zr1 | Zr | 0.75000 | 0.55994 | 0.25000 |
| 3114 | Zr1 | Zr | 0.75000 | 0.80994 | 0.00000 |
| 3115 | Zr1 | Zr | 0.50000 | 0.94006 | 0.00000 |
| 3116 | Zr1 | Zr | 0.50000 | 0.69006 | 0.25000 |
| 3117 | Zr1 | Zr | 0.75000 | 0.94006 | 0.25000 |
| 3118 | Zr1 | Zr | 0.75000 | 0.69006 | 0.00000 |
| 3119 | Zr1 | Zr | 0.50000 | 0.50000 | 0.05994 |
| 3120 | Zr1 | Zr | 0.50000 | 0.75000 | 0.30994 |
| 3121 | Zr1 | Zr | 0.75000 | 0.50000 | 0.30994 |
| 3122 | Zr1 | Zr | 0.75000 | 0.75000 | 0.05994 |
| 3123 | Zr1 | Zr | 0.50000 | 0.50000 | 0.44006 |
| 3124 | Zr1 | Zr | 0.50000 | 0.75000 | 0.19006 |
| 3125 | Zr1 | Zr | 0.75000 | 0.50000 | 0.19006 |
| 3126 | Zr1 | Zr | 0.75000 | 0.75000 | 0.44006 |
| 3127 | C2 | C | 0.63375 | 0.50000 | 0.59220 |
| 3128 | C2 | C | 0.63375 | 0.75000 | 0.84220 |
| 3129 | C2 | C | 0.88375 | 0.50000 | 0.84220 |
| 3130 | C2 | C | 0.88375 | 0.75000 | 0.59220 |
| 3131 | C2 | C | 0.86625 | 0.50000 | 0.59220 |
| 3132 | C2 | C | 0.86625 | 0.75000 | 0.84220 |

| | | | | | |
|---|---|---|---|---|---|
| 3133 | C2 | C | 0.61625 | 0.50000 | 0.84220 |
| 3134 | C2 | C | 0.61625 | 0.75000 | 0.59220 |
| 3135 | C2 | C | 0.86625 | 0.50000 | 0.90780 |
| 3136 | C2 | C | 0.86625 | 0.75000 | 0.65780 |
| 3137 | C2 | C | 0.61625 | 0.50000 | 0.65780 |
| 3138 | C2 | C | 0.61625 | 0.75000 | 0.90780 |
| 3139 | C2 | C | 0.63375 | 0.50000 | 0.90780 |
| 3140 | C2 | C | 0.63375 | 0.75000 | 0.65780 |
| 3141 | C2 | C | 0.88375 | 0.50000 | 0.65780 |
| 3142 | C2 | C | 0.88375 | 0.75000 | 0.90780 |
| 3143 | C2 | C | 0.59220 | 0.63375 | 0.50000 |
| 3144 | C2 | C | 0.59220 | 0.88375 | 0.75000 |
| 3145 | C2 | C | 0.84220 | 0.63375 | 0.75000 |
| 3146 | C2 | C | 0.84220 | 0.88375 | 0.50000 |
| 3147 | C2 | C | 0.59220 | 0.86625 | 0.50000 |
| 3148 | C2 | C | 0.59220 | 0.61625 | 0.75000 |
| 3149 | C2 | C | 0.84220 | 0.86625 | 0.75000 |
| 3150 | C2 | C | 0.90780 | 0.86625 | 0.50000 |
| 3151 | C2 | C | 0.90780 | 0.61625 | 0.75000 |
| 3152 | C2 | C | 0.65780 | 0.86625 | 0.75000 |
| 3153 | C2 | C | 0.65780 | 0.61625 | 0.50000 |
| 3154 | C2 | C | 0.90780 | 0.88375 | 0.75000 |
| 3155 | C2 | C | 0.65780 | 0.63375 | 0.75000 |
| 3156 | C2 | C | 0.65780 | 0.88375 | 0.50000 |
| 3157 | C2 | C | 0.50000 | 0.59220 | 0.63375 |
| 3158 | C2 | C | 0.50000 | 0.84220 | 0.88375 |
| 3159 | C2 | C | 0.75000 | 0.59220 | 0.88375 |
| 3160 | C2 | C | 0.75000 | 0.84220 | 0.63375 |
| 3161 | C2 | C | 0.50000 | 0.59220 | 0.86625 |
| 3162 | C2 | C | 0.50000 | 0.84220 | 0.61625 |
| 3163 | C2 | C | 0.75000 | 0.59220 | 0.61625 |
| 3164 | C2 | C | 0.75000 | 0.84220 | 0.86625 |
| 3165 | C2 | C | 0.50000 | 0.90780 | 0.86625 |
| 3166 | C2 | C | 0.50000 | 0.65780 | 0.61625 |
| 3167 | C2 | C | 0.75000 | 0.90780 | 0.61625 |
| 3168 | C2 | C | 0.75000 | 0.65780 | 0.86625 |
| 3169 | C2 | C | 0.50000 | 0.90780 | 0.63375 |
| 3170 | C2 | C | 0.50000 | 0.65780 | 0.88375 |
| 3171 | C2 | C | 0.75000 | 0.90780 | 0.88375 |
| 3172 | C2 | C | 0.75000 | 0.65780 | 0.63375 |
| 3173 | C2 | C | 0.50000 | 0.63375 | 0.90780 |
| 3174 | C2 | C | 0.50000 | 0.88375 | 0.65780 |
| 3175 | C2 | C | 0.75000 | 0.63375 | 0.65780 |
| 3176 | C2 | C | 0.75000 | 0.88375 | 0.90780 |
| 3177 | C2 | C | 0.50000 | 0.86625 | 0.90780 |
| 3178 | C2 | C | 0.50000 | 0.61625 | 0.65780 |
| 3179 | C2 | C | 0.75000 | 0.86625 | 0.65780 |
| 3180 | C2 | C | 0.75000 | 0.61625 | 0.90780 |
| 3181 | C2 | C | 0.50000 | 0.86625 | 0.59220 |
| 3182 | C2 | C | 0.50000 | 0.61625 | 0.84220 |
| 3183 | C2 | C | 0.75000 | 0.86625 | 0.84220 |
| 3184 | C2 | C | 0.75000 | 0.61625 | 0.59220 |
| 3185 | C2 | C | 0.50000 | 0.63375 | 0.59220 |
| 3186 | C2 | C | 0.50000 | 0.88375 | 0.84220 |

| | | | | | |
|---|---|---|---|---|---|
| 3187 | C2 | C | 0.75000 | 0.63375 | 0.84220 |
| 3188 | C2 | C | 0.75000 | 0.88375 | 0.59220 |
| 3189 | C2 | C | 0.63375 | 0.59220 | 0.50000 |
| 3190 | C2 | C | 0.63375 | 0.84220 | 0.75000 |
| 3191 | C2 | C | 0.88375 | 0.59220 | 0.75000 |
| 3192 | C2 | C | 0.88375 | 0.84220 | 0.50000 |
| 3193 | C2 | C | 0.86625 | 0.84220 | 0.75000 |
| 3194 | C2 | C | 0.61625 | 0.59220 | 0.75000 |
| 3195 | C2 | C | 0.61625 | 0.84220 | 0.50000 |
| 3196 | C2 | C | 0.86625 | 0.90780 | 0.50000 |
| 3197 | C2 | C | 0.86625 | 0.65780 | 0.75000 |
| 3198 | C2 | C | 0.61625 | 0.90780 | 0.75000 |
| 3199 | C2 | C | 0.61625 | 0.65780 | 0.50000 |
| 3200 | C2 | C | 0.63375 | 0.90780 | 0.50000 |
| 3201 | C2 | C | 0.63375 | 0.65780 | 0.75000 |
| 3202 | C2 | C | 0.88375 | 0.90780 | 0.75000 |
| 3203 | C2 | C | 0.59220 | 0.50000 | 0.86625 |
| 3204 | C2 | C | 0.59220 | 0.75000 | 0.61625 |
| 3205 | C2 | C | 0.84220 | 0.50000 | 0.61625 |
| 3206 | C2 | C | 0.84220 | 0.75000 | 0.86625 |
| 3207 | C2 | C | 0.59220 | 0.50000 | 0.63375 |
| 3208 | C2 | C | 0.59220 | 0.75000 | 0.88375 |
| 3209 | C2 | C | 0.84220 | 0.50000 | 0.88375 |
| 3210 | C2 | C | 0.84220 | 0.75000 | 0.63375 |
| 3211 | C2 | C | 0.90780 | 0.50000 | 0.63375 |
| 3212 | C2 | C | 0.90780 | 0.75000 | 0.88375 |
| 3213 | C2 | C | 0.65780 | 0.50000 | 0.88375 |
| 3214 | C2 | C | 0.65780 | 0.75000 | 0.63375 |
| 3215 | C2 | C | 0.90780 | 0.50000 | 0.86625 |
| 3216 | C2 | C | 0.90780 | 0.75000 | 0.61625 |
| 3217 | C2 | C | 0.65780 | 0.50000 | 0.61625 |
| 3218 | C2 | C | 0.65780 | 0.75000 | 0.86625 |
| 3219 | C1 | C | 0.57680 | 0.50000 | 0.92320 |
| 3220 | C1 | C | 0.57680 | 0.75000 | 0.67320 |
| 3221 | C1 | C | 0.82680 | 0.50000 | 0.67320 |
| 3222 | C1 | C | 0.82680 | 0.75000 | 0.92320 |
| 3223 | C1 | C | 0.92320 | 0.50000 | 0.92320 |
| 3224 | C1 | C | 0.92320 | 0.75000 | 0.67320 |
| 3225 | C1 | C | 0.67320 | 0.50000 | 0.67320 |
| 3226 | C1 | C | 0.67320 | 0.75000 | 0.92320 |
| 3227 | C1 | C | 0.92320 | 0.50000 | 0.57680 |
| 3228 | C1 | C | 0.92320 | 0.75000 | 0.82680 |
| 3229 | C1 | C | 0.67320 | 0.50000 | 0.82680 |
| 3230 | C1 | C | 0.67320 | 0.75000 | 0.57680 |
| 3231 | C1 | C | 0.57680 | 0.50000 | 0.57680 |
| 3232 | C1 | C | 0.57680 | 0.75000 | 0.82680 |
| 3233 | C1 | C | 0.82680 | 0.50000 | 0.82680 |
| 3234 | C1 | C | 0.82680 | 0.75000 | 0.57680 |
| 3235 | C1 | C | 0.92320 | 0.82680 | 0.75000 |
| 3236 | C1 | C | 0.67320 | 0.57680 | 0.75000 |
| 3237 | C1 | C | 0.67320 | 0.82680 | 0.50000 |
| 3238 | C1 | C | 0.92320 | 0.92320 | 0.50000 |
| 3239 | C1 | C | 0.92320 | 0.67320 | 0.75000 |
| 3240 | C1 | C | 0.67320 | 0.92320 | 0.75000 |

| | | | | | |
|---|---|---|---|---|---|
| 3241 | C1 | C | 0.67320 | 0.67320 | 0.50000 |
| 3242 | C1 | C | 0.57680 | 0.92320 | 0.50000 |
| 3243 | C1 | C | 0.57680 | 0.67320 | 0.75000 |
| 3244 | C1 | C | 0.82680 | 0.92320 | 0.75000 |
| 3245 | C1 | C | 0.57680 | 0.57680 | 0.50000 |
| 3246 | C1 | C | 0.57680 | 0.82680 | 0.75000 |
| 3247 | C1 | C | 0.82680 | 0.57680 | 0.75000 |
| 3248 | C1 | C | 0.82680 | 0.82680 | 0.50000 |
| 3249 | C1 | C | 0.50000 | 0.92320 | 0.57680 |
| 3250 | C1 | C | 0.50000 | 0.67320 | 0.82680 |
| 3251 | C1 | C | 0.75000 | 0.92320 | 0.82680 |
| 3252 | C1 | C | 0.75000 | 0.67320 | 0.57680 |
| 3253 | C1 | C | 0.50000 | 0.92320 | 0.92320 |
| 3254 | C1 | C | 0.50000 | 0.67320 | 0.67320 |
| 3255 | C1 | C | 0.75000 | 0.92320 | 0.67320 |
| 3256 | C1 | C | 0.75000 | 0.67320 | 0.92320 |
| 3257 | C1 | C | 0.50000 | 0.57680 | 0.92320 |
| 3258 | C1 | C | 0.50000 | 0.82680 | 0.67320 |
| 3259 | C1 | C | 0.75000 | 0.57680 | 0.67320 |
| 3260 | C1 | C | 0.75000 | 0.82680 | 0.92320 |
| 3261 | C1 | C | 0.50000 | 0.57680 | 0.57680 |
| 3262 | C1 | C | 0.50000 | 0.82680 | 0.82680 |
| 3263 | C1 | C | 0.75000 | 0.57680 | 0.82680 |
| 3264 | C1 | C | 0.75000 | 0.82680 | 0.57680 |
| 3265 | C3 | C | 0.60250 | 0.50000 | 0.60250 |
| 3266 | C3 | C | 0.60250 | 0.75000 | 0.85250 |
| 3267 | C3 | C | 0.85250 | 0.50000 | 0.85250 |
| 3268 | C3 | C | 0.85250 | 0.75000 | 0.60250 |
| 3269 | C3 | C | 0.89750 | 0.50000 | 0.60250 |
| 3270 | C3 | C | 0.89750 | 0.75000 | 0.85250 |
| 3271 | C3 | C | 0.64750 | 0.50000 | 0.85250 |
| 3272 | C3 | C | 0.64750 | 0.75000 | 0.60250 |
| 3273 | C3 | C | 0.89750 | 0.50000 | 0.89750 |
| 3274 | C3 | C | 0.89750 | 0.75000 | 0.64750 |
| 3275 | C3 | C | 0.64750 | 0.50000 | 0.64750 |
| 3276 | C3 | C | 0.64750 | 0.75000 | 0.89750 |
| 3277 | C3 | C | 0.60250 | 0.50000 | 0.89750 |
| 3278 | C3 | C | 0.60250 | 0.75000 | 0.64750 |
| 3279 | C3 | C | 0.85250 | 0.50000 | 0.64750 |
| 3280 | C3 | C | 0.85250 | 0.75000 | 0.89750 |
| 3281 | C3 | C | 0.60250 | 0.60250 | 0.50000 |
| 3282 | C3 | C | 0.60250 | 0.85250 | 0.75000 |
| 3283 | C3 | C | 0.85250 | 0.60250 | 0.75000 |
| 3284 | C3 | C | 0.85250 | 0.85250 | 0.50000 |
| 3285 | C3 | C | 0.60250 | 0.89750 | 0.50000 |
| 3286 | C3 | C | 0.60250 | 0.64750 | 0.75000 |
| 3287 | C3 | C | 0.85250 | 0.89750 | 0.75000 |
| 3288 | C3 | C | 0.89750 | 0.89750 | 0.50000 |
| 3289 | C3 | C | 0.89750 | 0.64750 | 0.75000 |
| 3290 | C3 | C | 0.64750 | 0.89750 | 0.75000 |
| 3291 | C3 | C | 0.64750 | 0.64750 | 0.50000 |
| 3292 | C3 | C | 0.89750 | 0.85250 | 0.75000 |
| 3293 | C3 | C | 0.64750 | 0.60250 | 0.75000 |
| 3294 | C3 | C | 0.64750 | 0.85250 | 0.50000 |

| | | | | | |
|---|---|---|---|---|---|
| 3295 | C3 | C | 0.50000 | 0.60250 | 0.60250 |
| 3296 | C3 | C | 0.50000 | 0.85250 | 0.85250 |
| 3297 | C3 | C | 0.75000 | 0.60250 | 0.85250 |
| 3298 | C3 | C | 0.75000 | 0.85250 | 0.60250 |
| 3299 | C3 | C | 0.50000 | 0.60250 | 0.89750 |
| 3300 | C3 | C | 0.50000 | 0.85250 | 0.64750 |
| 3301 | C3 | C | 0.75000 | 0.60250 | 0.64750 |
| 3302 | C3 | C | 0.75000 | 0.85250 | 0.89750 |
| 3303 | C3 | C | 0.50000 | 0.89750 | 0.89750 |
| 3304 | C3 | C | 0.50000 | 0.64750 | 0.64750 |
| 3305 | C3 | C | 0.75000 | 0.89750 | 0.64750 |
| 3306 | C3 | C | 0.75000 | 0.64750 | 0.89750 |
| 3307 | C3 | C | 0.50000 | 0.89750 | 0.60250 |
| 3308 | C3 | C | 0.50000 | 0.64750 | 0.85250 |
| 3309 | C3 | C | 0.75000 | 0.89750 | 0.85250 |
| 3310 | C3 | C | 0.75000 | 0.64750 | 0.60250 |
| 3311 | H1 | H | 0.63926 | 0.99997 | 0.56655 |
| 3312 | H1 | H | 0.63926 | 0.74997 | 0.81655 |
| 3313 | H1 | H | 0.88926 | 0.99997 | 0.81655 |
| 3314 | H1 | H | 0.88926 | 0.74997 | 0.56655 |
| 3315 | H1 | H | 0.86074 | 0.50003 | 0.56655 |
| 3316 | H1 | H | 0.86074 | 0.75004 | 0.81655 |
| 3317 | H1 | H | 0.61074 | 0.50003 | 0.81655 |
| 3318 | H1 | H | 0.61074 | 0.75004 | 0.56655 |
| 3319 | H1 | H | 0.86074 | 0.99996 | 0.93345 |
| 3320 | H1 | H | 0.86074 | 0.74997 | 0.68345 |
| 3321 | H1 | H | 0.61074 | 0.99997 | 0.68345 |
| 3322 | H1 | H | 0.61074 | 0.74997 | 0.93346 |
| 3323 | H1 | H | 0.63926 | 0.50003 | 0.93345 |
| 3324 | H1 | H | 0.63926 | 0.75003 | 0.68346 |
| 3325 | H1 | H | 0.88926 | 0.50003 | 0.68345 |
| 3326 | H1 | H | 0.88926 | 0.75004 | 0.93346 |
| 3327 | H1 | H | 0.56655 | 0.63926 | 0.99996 |
| 3328 | H1 | H | 0.56655 | 0.88926 | 0.74997 |
| 3329 | H1 | H | 0.81655 | 0.63926 | 0.74996 |
| 3330 | H1 | H | 0.81654 | 0.88926 | 0.99997 |
| 3331 | H1 | H | 0.56655 | 0.86074 | 0.50003 |
| 3332 | H1 | H | 0.56655 | 0.61074 | 0.75004 |
| 3333 | H1 | H | 0.81655 | 0.86074 | 0.75003 |
| 3334 | H1 | H | 0.93345 | 0.86074 | 0.99996 |
| 3335 | H1 | H | 0.93345 | 0.61074 | 0.74997 |
| 3336 | H1 | H | 0.68345 | 0.86074 | 0.74996 |
| 3337 | H1 | H | 0.68345 | 0.61074 | 0.99997 |
| 3338 | H1 | H | 0.93345 | 0.88926 | 0.75004 |
| 3339 | H1 | H | 0.68345 | 0.63926 | 0.75003 |
| 3340 | H1 | H | 0.68345 | 0.88926 | 0.50004 |
| 3341 | H1 | H | 0.99997 | 0.56655 | 0.63926 |
| 3342 | H1 | H | 0.99996 | 0.81655 | 0.88926 |
| 3343 | H1 | H | 0.74997 | 0.56655 | 0.88926 |
| 3344 | H1 | H | 0.74996 | 0.81655 | 0.63926 |
| 3345 | H1 | H | 0.50003 | 0.56655 | 0.86074 |
| 3346 | H1 | H | 0.50003 | 0.81655 | 0.61074 |
| 3347 | H1 | H | 0.75003 | 0.56655 | 0.61074 |
| 3348 | H1 | H | 0.75004 | 0.81655 | 0.86074 |

| | | | | | |
|---|---|---|---|---|---|
| 3349 | H1 | H | 0.99997 | 0.93345 | 0.86074 |
| 3350 | H1 | H | 0.99997 | 0.68345 | 0.61074 |
| 3351 | H1 | H | 0.74997 | 0.93345 | 0.61074 |
| 3352 | H1 | H | 0.74997 | 0.68345 | 0.86074 |
| 3353 | H1 | H | 0.50003 | 0.93345 | 0.63926 |
| 3354 | H1 | H | 0.50003 | 0.68345 | 0.88926 |
| 3355 | H1 | H | 0.75004 | 0.93345 | 0.88926 |
| 3356 | H1 | H | 0.75003 | 0.68346 | 0.63926 |
| 3357 | H1 | H | 0.50003 | 0.63926 | 0.93345 |
| 3358 | H1 | H | 0.50003 | 0.88926 | 0.68345 |
| 3359 | H1 | H | 0.75003 | 0.63926 | 0.68345 |
| 3360 | H1 | H | 0.75004 | 0.88926 | 0.93345 |
| 3361 | H1 | H | 0.99996 | 0.86074 | 0.93345 |
| 3362 | H1 | H | 0.99997 | 0.61074 | 0.68345 |
| 3363 | H1 | H | 0.74997 | 0.86074 | 0.68345 |
| 3364 | H1 | H | 0.74997 | 0.61074 | 0.93345 |
| 3365 | H1 | H | 0.50003 | 0.86074 | 0.56655 |
| 3366 | H1 | H | 0.50003 | 0.61074 | 0.81655 |
| 3367 | H1 | H | 0.75004 | 0.86074 | 0.81655 |
| 3368 | H1 | H | 0.75003 | 0.61074 | 0.56654 |
| 3369 | H1 | H | 0.99997 | 0.63926 | 0.56655 |
| 3370 | H1 | H | 0.99997 | 0.88926 | 0.81655 |
| 3371 | H1 | H | 0.74997 | 0.63926 | 0.81655 |
| 3372 | H1 | H | 0.74997 | 0.88926 | 0.56654 |
| 3373 | H1 | H | 0.63926 | 0.56655 | 0.99997 |
| 3374 | H1 | H | 0.63926 | 0.81655 | 0.74996 |
| 3375 | H1 | H | 0.88926 | 0.56655 | 0.74997 |
| 3376 | H1 | H | 0.88926 | 0.81655 | 0.99996 |
| 3377 | H1 | H | 0.86074 | 0.81655 | 0.75003 |
| 3378 | H1 | H | 0.61074 | 0.56655 | 0.75003 |
| 3379 | H1 | H | 0.61074 | 0.81655 | 0.50003 |
| 3380 | H1 | H | 0.86074 | 0.93345 | 0.99997 |
| 3381 | H1 | H | 0.86074 | 0.68345 | 0.74997 |
| 3382 | H1 | H | 0.61074 | 0.93345 | 0.74997 |
| 3383 | H1 | H | 0.61074 | 0.68345 | 0.99996 |
| 3384 | H1 | H | 0.63926 | 0.93345 | 0.50004 |
| 3385 | H1 | H | 0.63926 | 0.68346 | 0.75003 |
| 3386 | H1 | H | 0.88926 | 0.93345 | 0.75004 |
| 3387 | H1 | H | 0.56655 | 0.50003 | 0.86074 |
| 3388 | H1 | H | 0.56655 | 0.75004 | 0.61074 |
| 3389 | H1 | H | 0.81655 | 0.50003 | 0.61074 |
| 3390 | H1 | H | 0.81655 | 0.75004 | 0.86074 |
| 3391 | H1 | H | 0.56655 | 0.99996 | 0.63926 |
| 3392 | H1 | H | 0.56655 | 0.74997 | 0.88926 |
| 3393 | H1 | H | 0.81655 | 0.99996 | 0.88926 |
| 3394 | H1 | H | 0.81655 | 0.74997 | 0.63926 |
| 3395 | H1 | H | 0.93345 | 0.50003 | 0.63926 |
| 3396 | H1 | H | 0.93345 | 0.75004 | 0.88926 |
| 3397 | H1 | H | 0.68345 | 0.50003 | 0.88926 |
| 3398 | H1 | H | 0.68345 | 0.75003 | 0.63926 |
| 3399 | H1 | H | 0.93345 | 0.99996 | 0.86074 |
| 3400 | H1 | H | 0.93345 | 0.74997 | 0.61074 |
| 3401 | H1 | H | 0.68345 | 0.99996 | 0.61074 |
| 3402 | H1 | H | 0.68345 | 0.74997 | 0.86074 |

| | | | | | |
|---|---|---|---|---|---|
| 3403 | H2 | H | 0.70825 | 0.79176 | 0.95824 |
| 3404 | H2 | H | 0.79174 | 0.70825 | 0.95823 |
| 3405 | H2 | H | 0.70827 | 0.70823 | 0.54177 |
| 3406 | H2 | H | 0.79179 | 0.79168 | 0.54178 |
| 3407 | H2 | H | 0.95823 | 0.79173 | 0.70823 |
| 3408 | H2 | H | 0.95824 | 0.70825 | 0.79176 |
| 3409 | H2 | H | 0.54174 | 0.79176 | 0.79177 |
| 3410 | H2 | H | 0.54173 | 0.70823 | 0.70823 |
| 3411 | H2 | H | 0.79175 | 0.95824 | 0.70824 |
| 3412 | H2 | H | 0.70824 | 0.95824 | 0.79176 |
| 3413 | H2 | H | 0.70823 | 0.54172 | 0.70822 |
| 3414 | H2 | H | 0.79177 | 0.54173 | 0.79178 |
| 3415 | H2 | H | 0.54178 | 0.54177 | 0.54173 |
| 3416 | H2 | H | 0.95823 | 0.95824 | 0.54174 |
| 3417 | H2 | H | 0.95823 | 0.54176 | 0.95825 |
| 3418 | H2 | H | 0.54176 | 0.95824 | 0.95824 |
| 3419 | O1 | O | 0.58524 | 0.50000 | 0.54765 |
| 3420 | O1 | O | 0.58524 | 0.75000 | 0.79765 |
| 3421 | O1 | O | 0.83524 | 0.50000 | 0.79765 |
| 3422 | O1 | O | 0.83524 | 0.75000 | 0.54765 |
| 3423 | O1 | O | 0.91476 | 0.50000 | 0.54765 |
| 3424 | O1 | O | 0.91476 | 0.75000 | 0.79765 |
| 3425 | O1 | O | 0.66476 | 0.50000 | 0.79765 |
| 3426 | O1 | O | 0.66476 | 0.75000 | 0.54765 |
| 3427 | O1 | O | 0.91476 | 0.50000 | 0.95235 |
| 3428 | O1 | O | 0.91476 | 0.75000 | 0.70235 |
| 3429 | O1 | O | 0.66476 | 0.50000 | 0.70235 |
| 3430 | O1 | O | 0.66476 | 0.75000 | 0.95235 |
| 3431 | O1 | O | 0.58524 | 0.50000 | 0.95235 |
| 3432 | O1 | O | 0.58524 | 0.75000 | 0.70235 |
| 3433 | O1 | O | 0.83524 | 0.50000 | 0.70235 |
| 3434 | O1 | O | 0.83524 | 0.75000 | 0.95235 |
| 3435 | O1 | O | 0.54765 | 0.58524 | 0.50000 |
| 3436 | O1 | O | 0.54765 | 0.83524 | 0.75000 |
| 3437 | O1 | O | 0.79765 | 0.58524 | 0.75000 |
| 3438 | O1 | O | 0.79765 | 0.83524 | 0.50000 |
| 3439 | O1 | O | 0.54765 | 0.91476 | 0.50000 |
| 3440 | O1 | O | 0.54765 | 0.66476 | 0.75000 |
| 3441 | O1 | O | 0.79765 | 0.91476 | 0.75000 |
| 3442 | O3 | O | 0.79765 | 0.66476 | 0.50000 |
| 3443 | O1 | O | 0.95235 | 0.91476 | 0.50000 |
| 3444 | O1 | O | 0.95235 | 0.66476 | 0.75000 |
| 3445 | O1 | O | 0.70235 | 0.91476 | 0.75000 |
| 3446 | O1 | O | 0.70235 | 0.66476 | 0.50000 |
| 3447 | O3 | O | 0.95235 | 0.58524 | 0.50000 |
| 3448 | O1 | O | 0.95235 | 0.83524 | 0.75000 |
| 3449 | O1 | O | 0.70235 | 0.58524 | 0.75000 |
| 3450 | O1 | O | 0.70235 | 0.83524 | 0.50000 |
| 3451 | O1 | O | 0.50000 | 0.54765 | 0.58524 |
| 3452 | O1 | O | 0.50000 | 0.79765 | 0.83524 |
| 3453 | O1 | O | 0.75000 | 0.54765 | 0.83524 |
| 3454 | O1 | O | 0.75000 | 0.79765 | 0.58524 |
| 3455 | O1 | O | 0.50000 | 0.54765 | 0.91476 |
| 3456 | O1 | O | 0.50000 | 0.79765 | 0.66476 |

| | | | | | |
|---|---|---|---|---|---|
| 3457 | O1 | O | 0.75000 | 0.54765 | 0.66476 |
| 3458 | O1 | O | 0.75000 | 0.79765 | 0.91476 |
| 3459 | O1 | O | 0.50000 | 0.95235 | 0.91476 |
| 3460 | O1 | O | 0.50000 | 0.70235 | 0.66476 |
| 3461 | O1 | O | 0.75000 | 0.95235 | 0.66476 |
| 3462 | O1 | O | 0.75000 | 0.70235 | 0.91476 |
| 3463 | O1 | O | 0.50000 | 0.95235 | 0.58524 |
| 3464 | O1 | O | 0.50000 | 0.70235 | 0.83524 |
| 3465 | O1 | O | 0.75000 | 0.95235 | 0.83524 |
| 3466 | O1 | O | 0.75000 | 0.70235 | 0.58524 |
| 3467 | O1 | O | 0.50000 | 0.58524 | 0.95235 |
| 3468 | O1 | O | 0.50000 | 0.83524 | 0.70235 |
| 3469 | O1 | O | 0.75000 | 0.58524 | 0.70235 |
| 3470 | O1 | O | 0.75000 | 0.83524 | 0.95235 |
| 3471 | O1 | O | 0.50000 | 0.91476 | 0.95235 |
| 3472 | O1 | O | 0.50000 | 0.66476 | 0.70235 |
| 3473 | O1 | O | 0.75000 | 0.91476 | 0.70235 |
| 3474 | O1 | O | 0.75000 | 0.66476 | 0.95235 |
| 3475 | O1 | O | 0.50000 | 0.91476 | 0.54765 |
| 3476 | O1 | O | 0.50000 | 0.66476 | 0.79765 |
| 3477 | O1 | O | 0.75000 | 0.91476 | 0.79765 |
| 3478 | O1 | O | 0.75000 | 0.66476 | 0.54765 |
| 3479 | O1 | O | 0.50000 | 0.58524 | 0.54765 |
| 3480 | O1 | O | 0.50000 | 0.83524 | 0.79765 |
| 3481 | O1 | O | 0.75000 | 0.58524 | 0.79765 |
| 3482 | O1 | O | 0.75000 | 0.83524 | 0.54765 |
| 3483 | O1 | O | 0.58524 | 0.54765 | 0.50000 |
| 3484 | O1 | O | 0.58524 | 0.79765 | 0.75000 |
| 3485 | O1 | O | 0.83524 | 0.54765 | 0.75000 |
| 3486 | O1 | O | 0.83524 | 0.79765 | 0.50000 |
| 3487 | O3 | O | 0.91476 | 0.54765 | 0.50000 |
| 3488 | O1 | O | 0.91476 | 0.79765 | 0.75000 |
| 3489 | O1 | O | 0.66476 | 0.54765 | 0.75000 |
| 3490 | O1 | O | 0.66476 | 0.79765 | 0.50000 |
| 3491 | O1 | O | 0.91476 | 0.95235 | 0.50000 |
| 3492 | O1 | O | 0.91476 | 0.70235 | 0.75000 |
| 3493 | O1 | O | 0.66476 | 0.95235 | 0.75000 |
| 3494 | O1 | O | 0.66476 | 0.70235 | 0.50000 |
| 3495 | O1 | O | 0.58524 | 0.95235 | 0.50000 |
| 3496 | O1 | O | 0.58524 | 0.70235 | 0.75000 |
| 3497 | O1 | O | 0.83524 | 0.95235 | 0.75000 |
| 3498 | O3 | O | 0.83524 | 0.70235 | 0.50000 |
| 3499 | O1 | O | 0.54765 | 0.50000 | 0.91476 |
| 3500 | O1 | O | 0.54765 | 0.75000 | 0.66476 |
| 3501 | O1 | O | 0.79765 | 0.50000 | 0.66476 |
| 3502 | O1 | O | 0.79765 | 0.75000 | 0.91476 |
| 3503 | O1 | O | 0.54765 | 0.50000 | 0.58524 |
| 3504 | O1 | O | 0.54765 | 0.75000 | 0.83524 |
| 3505 | O1 | O | 0.79765 | 0.50000 | 0.83524 |
| 3506 | O1 | O | 0.79765 | 0.75000 | 0.58524 |
| 3507 | O1 | O | 0.95235 | 0.50000 | 0.58524 |
| 3508 | O1 | O | 0.95235 | 0.75000 | 0.83524 |
| 3509 | O1 | O | 0.70235 | 0.50000 | 0.83524 |
| 3510 | O1 | O | 0.70235 | 0.75000 | 0.58524 |

| | | | | | |
|---|---|---|---|---|---|
| 3511 | O1 | O | 0.95235 | 0.50000 | 0.91476 |
| 3512 | O1 | O | 0.95235 | 0.75000 | 0.66476 |
| 3513 | O1 | O | 0.70235 | 0.50000 | 0.66476 |
| 3514 | O1 | O | 0.70235 | 0.75000 | 0.91476 |
| 3515 | O2 | O | 0.52805 | 0.97195 | 0.97195 |
| 3516 | O2 | O | 0.52805 | 0.72195 | 0.72195 |
| 3517 | O2 | O | 0.77805 | 0.97195 | 0.72195 |
| 3518 | O2 | O | 0.77805 | 0.72195 | 0.97195 |
| 3519 | O2 | O | 0.97195 | 0.52805 | 0.97195 |
| 3520 | O2 | O | 0.97195 | 0.77805 | 0.72195 |
| 3521 | O2 | O | 0.72195 | 0.52805 | 0.72195 |
| 3522 | O2 | O | 0.72195 | 0.77805 | 0.97195 |
| 3523 | O2 | O | 0.97195 | 0.97195 | 0.52805 |
| 3524 | O2 | O | 0.97195 | 0.72195 | 0.77805 |
| 3525 | O2 | O | 0.72195 | 0.97195 | 0.77805 |
| 3526 | O2 | O | 0.72195 | 0.72195 | 0.52805 |
| 3527 | O2 | O | 0.52805 | 0.52805 | 0.52805 |
| 3528 | O2 | O | 0.52805 | 0.77805 | 0.77805 |
| 3529 | O2 | O | 0.77805 | 0.52805 | 0.77805 |
| 3530 | O2 | O | 0.77805 | 0.77805 | 0.52805 |
| 3531 | O1 | O | 0.97195 | 0.52805 | 0.52805 |
| 3532 | O1 | O | 0.97195 | 0.77805 | 0.77805 |
| 3533 | O1 | O | 0.72195 | 0.52805 | 0.77805 |
| 3534 | O1 | O | 0.72195 | 0.77805 | 0.52805 |
| 3535 | O1 | O | 0.52805 | 0.97195 | 0.52805 |
| 3536 | O1 | O | 0.52805 | 0.72195 | 0.77805 |
| 3537 | O1 | O | 0.77805 | 0.97195 | 0.77805 |
| 3538 | O1 | O | 0.77805 | 0.72195 | 0.52805 |
| 3539 | O1 | O | 0.97195 | 0.97195 | 0.97195 |
| 3540 | O1 | O | 0.97195 | 0.72195 | 0.72195 |
| 3541 | O1 | O | 0.72195 | 0.97195 | 0.72195 |
| 3542 | O1 | O | 0.72195 | 0.72195 | 0.97195 |
| 3543 | O1 | O | 0.52805 | 0.52805 | 0.97195 |
| 3544 | O1 | O | 0.52805 | 0.77805 | 0.72195 |
| 3545 | O1 | O | 0.77805 | 0.52805 | 0.72195 |
| 3546 | O1 | O | 0.77805 | 0.77805 | 0.97195 |
| 3547 | Zr1 | Zr | 0.55994 | 0.50000 | 0.50000 |
| 3548 | Zr1 | Zr | 0.55994 | 0.75000 | 0.75000 |
| 3549 | Zr1 | Zr | 0.80994 | 0.50000 | 0.75000 |
| 3550 | Zr1 | Zr | 0.80994 | 0.75000 | 0.50000 |
| 3551 | Zr1 | Zr | 0.94006 | 0.50000 | 0.50000 |
| 3552 | Zr1 | Zr | 0.94006 | 0.75000 | 0.75000 |
| 3553 | Zr1 | Zr | 0.69006 | 0.50000 | 0.75000 |
| 3554 | Zr1 | Zr | 0.69006 | 0.75000 | 0.50000 |
| 3555 | Zr1 | Zr | 0.50000 | 0.55994 | 0.50000 |
| 3556 | Zr1 | Zr | 0.50000 | 0.80994 | 0.75000 |
| 3557 | Zr1 | Zr | 0.75000 | 0.55994 | 0.75000 |
| 3558 | Zr1 | Zr | 0.75000 | 0.80994 | 0.50000 |
| 3559 | Zr1 | Zr | 0.50000 | 0.94006 | 0.50000 |
| 3560 | Zr1 | Zr | 0.50000 | 0.69006 | 0.75000 |
| 3561 | Zr1 | Zr | 0.75000 | 0.94006 | 0.75000 |
| 3562 | Zr1 | Zr | 0.75000 | 0.69006 | 0.50000 |
| 3563 | Zr1 | Zr | 0.50000 | 0.50000 | 0.55994 |
| 3564 | Zr1 | Zr | 0.50000 | 0.75000 | 0.80994 |

| | | | | | |
|---|---|---|---|---|---|
| 3565 | Zr1 | Zr | 0.75000 | 0.50000 | 0.80994 |
| 3566 | Zr1 | Zr | 0.75000 | 0.75000 | 0.55994 |
| 3567 | Zr1 | Zr | 0.50000 | 0.50000 | 0.94006 |
| 3568 | Zr1 | Zr | 0.50000 | 0.75000 | 0.69006 |
| 3569 | Zr1 | Zr | 0.75000 | 0.50000 | 0.69006 |
| 3570 | Zr1 | Zr | 0.75000 | 0.75000 | 0.94006 |
| 3571 | H3 | H | 0.45705 | 0.60274 | 0.49978 |
| 3572 | H3 | H | 0.39442 | 0.54974 | 0.49979 |
| 3573 | H3 | H | 0.35697 | 0.70270 | 0.50002 |
| 3574 | H3 | H | 0.28977 | 0.64489 | 0.49991 |
| 3575 | H3 | H | 0.64386 | 0.45601 | 0.24981 |
| 3576 | H3 | H | 0.70804 | 0.39699 | 0.24968 |
| 3577 | H3 | H | 0.54730 | 0.35566 | 0.25047 |
| 3578 | H3 | H | 0.60740 | 0.29773 | 0.25004 |
| 3579 | H3 | H | 0.04695 | 0.10657 | 0.99983 |
| 3580 | H3 | H | 0.10864 | 0.04687 | 1.00001 |
| 3581 | H3 | H | 0.14301 | 0.20303 | 1.00119 |
| 3582 | H3 | H | 0.20304 | 0.14279 | 1.00005 |
| 3583 | H3 | H | 0.95291 | 0.60674 | 0.49652 |
| 3584 | H3 | H | 0.89295 | 0.54695 | 0.49832 |
| 3585 | H3 | H | 0.85720 | 0.70306 | 0.49969 |
| 3586 | H3 | H | 0.79694 | 0.64282 | 0.49937 |
| 3587 | H3 | H | 0.10914 | 0.79684 | 0.75005 |
| 3588 | H3 | H | 0.04692 | 0.85681 | 0.75071 |
| 3589 | H3 | H | 0.20306 | 0.89312 | 0.74987 |
| 3590 | H3 | H | 0.14328 | 0.95304 | 0.75074 |
| 3591 | H3 | H | 0.85750 | 0.20308 | 0.49999 |
| 3592 | H3 | H | 0.79696 | 0.14318 | 0.50031 |
| 3593 | H3 | H | 0.95305 | 0.10673 | 0.50030 |
| 3594 | H3 | H | 0.89283 | 0.04696 | 0.49842 |
| 3595 | H3 | H | 0.54704 | 0.85681 | 0.24680 |
| 3596 | H3 | H | 0.60737 | 0.79695 | 0.24979 |
| 3597 | H3 | H | 0.70303 | 0.89281 | 0.25176 |
| 3598 | H3 | H | 0.64294 | 0.95288 | 0.24619 |
| 3599 | H3 | H | 0.10718 | 0.29694 | 0.75132 |
| 3600 | H3 | H | 0.04694 | 0.35699 | 0.74955 |
| 3601 | H3 | H | 0.14241 | 0.45308 | 0.75119 |
| 3602 | H3 | H | 0.20306 | 0.39265 | 0.75001 |
