# Supplementary material for: Defect-induced tuning of polarity-dependent adsorption in hydrophobic–hydrophilic UiO-66
Source: Commun Chem. 2022 Oct 7;5:120. doi: 10.1038/s42004-022-00742-z (PMC9814431; doi:10.1038/s42004-022-00742-z)
Supplement: Supplementary file 8 — Supplementary Data 5 [file 42004_2022_742_MOESM8_ESM.pdf]

```
data_UiO-66_32

_cell_length_a    20.70040
_cell_length_b    20.70040
_cell_length_c    20.70040
_cell_angle_alpha 90
_cell_angle_beta  90
_cell_angle_gamma 90


_symmetry_equiv_pos_as_xyz 'x,y,z'

loop_
_atom_site_label
_atom_site_type_symbol
_atom_site_fract_x
_atom_site_fract_y
_atom_site_fract_z
C2     C      0.267500000000     0.000000000000     0.184400000000
C2     C      0.267500000000     0.500000000000     0.684400000000
C2     C      0.767500000000     0.000000000000     0.684400000000
C2     C      0.767500000000     0.500000000000     0.184400000000
C2     C      0.732500000000     0.000000000000     0.184400000000
C2     C      0.732500000000     0.500000000000     0.684400000000
C2     C      0.232500000000     0.000000000000     0.684400000000
C2     C      0.232500000000     0.500000000000     0.184400000000
C2     C      0.732500000000     0.000000000000     0.815600000000
C2     C      0.732500000000     0.500000000000     0.315600000000
C2     C      0.232500000000     0.000000000000     0.315600000000
C2     C      0.232500000000     0.500000000000     0.815600000000
C2     C      0.267500000000     0.000000000000     0.815600000000
C2     C      0.267500000000     0.500000000000     0.315600000000
C2     C      0.767500000000     0.000000000000     0.315600000000
C2     C      0.767500000000     0.500000000000     0.815600000000
C2     C      0.184400000000     0.267500000000     0.000000000000
C2     C      0.684400000000     0.767500000000     0.000000000000
C2     C      0.184400000000     0.732500000000     0.000000000000
C2     C      0.684400000000     0.232500000000     0.000000000000
C2     C      0.815600000000     0.732500000000     0.000000000000
C2     C      0.315600000000     0.232500000000     0.000000000000
C2     C      0.815600000000     0.267500000000     0.000000000000
C2     C      0.315600000000     0.767500000000     0.000000000000
C2     C      0.000000000000     0.184400000000     0.267500000000
C2     C      0.000000000000     0.684400000000     0.767500000000
C2     C      0.500000000000     0.184400000000     0.767500000000
C2     C      0.500000000000     0.684400000000     0.267500000000
C2     C      0.000000000000     0.184400000000     0.732500000000
C2     C      0.000000000000     0.684400000000     0.232500000000
C2     C      0.500000000000     0.184400000000     0.232500000000
C2     C      0.500000000000     0.684400000000     0.732500000000
C2     C      0.000000000000     0.815600000000     0.732500000000
C2     C      0.000000000000     0.315600000000     0.232500000000
C2     C      0.500000000000     0.815600000000     0.232500000000
C2     C      0.500000000000     0.315600000000     0.732500000000
```

55	C2	C	0.000000000000	0.815600000000	0.267500000000
56	C2	C	0.000000000000	0.315600000000	0.767500000000
57	C2	C	0.500000000000	0.815600000000	0.767500000000
58	C2	C	0.500000000000	0.315600000000	0.267500000000
59	C2	C	0.000000000000	0.267500000000	0.815600000000
60	C2	C	0.000000000000	0.767500000000	0.315600000000
61	C2	C	0.500000000000	0.267500000000	0.315600000000
62	C2	C	0.500000000000	0.767500000000	0.815600000000
63	C2	C	0.000000000000	0.732500000000	0.815600000000
64	C2	C	0.000000000000	0.232500000000	0.315600000000
65	C2	C	0.500000000000	0.732500000000	0.315600000000
66	C2	C	0.500000000000	0.232500000000	0.815600000000
67	C2	C	0.000000000000	0.732500000000	0.184400000000
68	C2	C	0.000000000000	0.232500000000	0.684400000000
69	C2	C	0.500000000000	0.732500000000	0.684400000000
70	C2	C	0.500000000000	0.232500000000	0.184400000000
71	C2	C	0.000000000000	0.267500000000	0.184400000000
72	C2	C	0.000000000000	0.767500000000	0.684400000000
73	C2	C	0.500000000000	0.267500000000	0.684400000000
74	C2	C	0.500000000000	0.767500000000	0.184400000000
75	C2	C	0.267500000000	0.184400000000	0.000000000000
76	C2	C	0.767500000000	0.684400000000	0.000000000000
77	C2	C	0.732500000000	0.184400000000	0.000000000000
78	C2	C	0.232500000000	0.684400000000	0.000000000000
79	C2	C	0.732500000000	0.815600000000	0.000000000000
80	C2	C	0.232500000000	0.315600000000	0.000000000000
81	C2	C	0.267500000000	0.815600000000	0.000000000000
82	C2	C	0.767500000000	0.315600000000	0.000000000000
83	C2	C	0.184400000000	0.000000000000	0.732500000000
84	C2	C	0.184400000000	0.500000000000	0.232500000000
85	C2	C	0.684400000000	0.000000000000	0.232500000000
86	C2	C	0.684400000000	0.500000000000	0.732500000000
87	C2	C	0.184400000000	0.000000000000	0.267500000000
88	C2	C	0.184400000000	0.500000000000	0.767500000000
89	C2	C	0.684400000000	0.000000000000	0.767500000000
90	C2	C	0.684400000000	0.500000000000	0.267500000000
91	C2	C	0.815600000000	0.000000000000	0.267500000000
92	C2	C	0.815600000000	0.500000000000	0.767500000000
93	C2	C	0.315600000000	0.000000000000	0.767500000000
94	C2	C	0.315600000000	0.500000000000	0.267500000000
95	C2	C	0.815600000000	0.000000000000	0.732500000000
96	C2	C	0.815600000000	0.500000000000	0.232500000000
97	C2	C	0.315600000000	0.000000000000	0.232500000000
98	C2	C	0.315600000000	0.500000000000	0.732500000000
99	C1	C	0.153600000000	0.000000000000	0.846400000000
100	C1	C	0.153600000000	0.500000000000	0.346400000000
101	C1	C	0.653600000000	0.000000000000	0.346400000000
102	C1	C	0.653600000000	0.500000000000	0.846400000000
103	C1	C	0.846400000000	0.000000000000	0.846400000000
104	C1	C	0.846400000000	0.500000000000	0.346400000000
105	C1	C	0.346400000000	0.000000000000	0.346400000000
106	C1	C	0.346400000000	0.500000000000	0.846400000000
107	C1	C	0.846400000000	0.000000000000	0.153600000000
108	C1	C	0.846400000000	0.500000000000	0.653600000000

109	C1	C	0.346400000000	0.000000000000	0.653600000000
110	C1	C	0.346400000000	0.500000000000	0.153600000000
111	C1	C	0.153600000000	0.000000000000	0.153600000000
112	C1	C	0.153600000000	0.500000000000	0.653600000000
113	C1	C	0.653600000000	0.000000000000	0.653600000000
114	C1	C	0.653600000000	0.500000000000	0.153600000000
115	C1	C	0.846400000000	0.153600000000	0.000000000000
116	C1	C	0.346400000000	0.653600000000	0.000000000000
117	C1	C	0.846400000000	0.846400000000	0.000000000000
118	C1	C	0.346400000000	0.346400000000	0.000000000000
119	C1	C	0.153600000000	0.846400000000	0.000000000000
120	C1	C	0.653600000000	0.346400000000	0.000000000000
121	C1	C	0.153600000000	0.153600000000	0.000000000000
122	C1	C	0.653600000000	0.653600000000	0.000000000000
123	C1	C	0.000000000000	0.846400000000	0.153600000000
124	C1	C	0.000000000000	0.346400000000	0.653600000000
125	C1	C	0.500000000000	0.846400000000	0.653600000000
126	C1	C	0.500000000000	0.346400000000	0.153600000000
127	C1	C	0.000000000000	0.846400000000	0.846400000000
128	C1	C	0.000000000000	0.346400000000	0.346400000000
129	C1	C	0.500000000000	0.846400000000	0.346400000000
130	C1	C	0.500000000000	0.346400000000	0.846400000000
131	C1	C	0.000000000000	0.153600000000	0.846400000000
132	C1	C	0.000000000000	0.653600000000	0.346400000000
133	C1	C	0.500000000000	0.153600000000	0.346400000000
134	C1	C	0.500000000000	0.653600000000	0.846400000000
135	C1	C	0.000000000000	0.153600000000	0.153600000000
136	C1	C	0.000000000000	0.653600000000	0.653600000000
137	C1	C	0.500000000000	0.153600000000	0.653600000000
138	C1	C	0.500000000000	0.653600000000	0.153600000000
139	C3	C	0.205000000000	0.000000000000	0.205000000000
140	C3	C	0.205000000000	0.500000000000	0.705000000000
141	C3	C	0.705000000000	0.000000000000	0.705000000000
142	C3	C	0.705000000000	0.500000000000	0.205000000000
143	C3	C	0.795000000000	0.000000000000	0.205000000000
144	C3	C	0.795000000000	0.500000000000	0.705000000000
145	C3	C	0.295000000000	0.000000000000	0.705000000000
146	C3	C	0.295000000000	0.500000000000	0.205000000000
147	C3	C	0.795000000000	0.000000000000	0.795000000000
148	C3	C	0.795000000000	0.500000000000	0.295000000000
149	C3	C	0.295000000000	0.000000000000	0.295000000000
150	C3	C	0.295000000000	0.500000000000	0.795000000000
151	C3	C	0.205000000000	0.000000000000	0.795000000000
152	C3	C	0.205000000000	0.500000000000	0.295000000000
153	C3	C	0.705000000000	0.000000000000	0.295000000000
154	C3	C	0.705000000000	0.500000000000	0.795000000000
155	C3	C	0.205000000000	0.205000000000	0.000000000000
156	C3	C	0.705000000000	0.705000000000	0.000000000000
157	C3	C	0.205000000000	0.795000000000	0.000000000000
158	C3	C	0.705000000000	0.295000000000	0.000000000000
159	C3	C	0.795000000000	0.795000000000	0.000000000000
160	C3	C	0.295000000000	0.295000000000	0.000000000000
161	C3	C	0.795000000000	0.205000000000	0.000000000000
162	C3	C	0.295000000000	0.705000000000	0.000000000000

163	C3	C	0.000000000000	0.205000000000	0.205000000000
164	C3	C	0.000000000000	0.705000000000	0.705000000000
165	C3	C	0.500000000000	0.205000000000	0.705000000000
166	C3	C	0.500000000000	0.705000000000	0.205000000000
167	C3	C	0.000000000000	0.205000000000	0.795000000000
168	C3	C	0.000000000000	0.705000000000	0.295000000000
169	C3	C	0.500000000000	0.205000000000	0.295000000000
170	C3	C	0.500000000000	0.705000000000	0.795000000000
171	C3	C	0.000000000000	0.795000000000	0.795000000000
172	C3	C	0.000000000000	0.295000000000	0.295000000000
173	C3	C	0.500000000000	0.795000000000	0.295000000000
174	C3	C	0.500000000000	0.295000000000	0.795000000000
175	C3	C	0.000000000000	0.795000000000	0.205000000000
176	C3	C	0.000000000000	0.295000000000	0.705000000000
177	C3	C	0.500000000000	0.795000000000	0.705000000000
178	C3	C	0.500000000000	0.295000000000	0.205000000000
179	H1	H	0.278520000000	0.999929999903	0.133090000000
180	H1	H	0.278520000000	0.499929999903	0.633090000193
181	H1	H	0.778520000097	0.999929999903	0.633090000193
182	H1	H	0.778520000097	0.499940000193	0.133090000000
183	H1	H	0.721479999903	0.000060000000	0.133090000000
184	H1	H	0.721479999903	0.500070000097	0.633090000193
185	H1	H	0.221480000000	0.000070000000	0.633090000193
186	H1	H	0.221480000000	0.500070000097	0.133090000000
187	H1	H	0.721479999903	0.999929999903	0.866909999807
188	H1	H	0.721479999903	0.499929999903	0.366910000000
189	H1	H	0.221480000000	0.999929999903	0.366910000000
190	H1	H	0.221480000000	0.499940000193	0.866909999807
191	H1	H	0.278520000000	0.000070000000	0.866909999807
192	H1	H	0.278520000000	0.500059999807	0.366910000000
193	H1	H	0.778520000097	0.000070000000	0.366910000000
194	H1	H	0.778520000097	0.500070000097	0.866909999807
195	H1	H	0.133090000000	0.278520000000	0.999929999903
196	H1	H	0.633090000193	0.778509999807	0.999940000193
197	H1	H	0.133090000000	0.721479999903	0.000060000000
198	H1	H	0.633090000193	0.221480000000	0.000070000000
199	H1	H	0.866909999807	0.721479999903	0.999929999903
200	H1	H	0.366910000000	0.221480000000	0.999940000193
201	H1	H	0.866909999807	0.278520000000	0.000060000000
202	H1	H	0.366910000000	0.778520000097	0.000080000000
203	H1	H	0.999929999903	0.133090000000	0.278520000000
204	H1	H	0.999929999903	0.633090000193	0.778520000097
205	H1	H	0.499940000193	0.133090000000	0.778520000097
206	H1	H	0.499929999903	0.633090000193	0.278520000000
207	H1	H	0.000070000000	0.133090000000	0.721479999903
208	H1	H	0.000070000000	0.633090000193	0.221480000000
209	H1	H	0.500059999807	0.133090000000	0.221480000000
210	H1	H	0.500070000097	0.633090000193	0.721479999903
211	H1	H	0.999929999903	0.866909999807	0.721479999903
212	H1	H	0.999929999903	0.366910000000	0.221480000000
213	H1	H	0.499929999903	0.866909999807	0.221480000000
214	H1	H	0.499940000193	0.366910000000	0.721479999903
215	H1	H	0.000070000000	0.866909999807	0.278520000000
216	H1	H	0.000060000000	0.366910000000	0.778520000097

217	H1	H	0.500070000097	0.866909999807	0.778520000097
218	H1	H	0.500059999807	0.366910000000	0.278520000000
219	H1	H	0.000070000000	0.278520000000	0.866909999807
220	H1	H	0.000070000000	0.778520000097	0.366910000000
221	H1	H	0.500070000097	0.278520000000	0.366910000000
222	H1	H	0.500079999903	0.778520000097	0.866909999807
223	H1	H	0.999929999903	0.721479999903	0.866909999807
224	H1	H	0.999929999903	0.221480000000	0.366910000000
225	H1	H	0.499929999903	0.721479999903	0.366910000000
226	H1	H	0.499940000193	0.221480000000	0.866909999807
227	H1	H	0.000060000000	0.721479999903	0.133090000000
228	H1	H	0.000070000000	0.221480000000	0.633090000193
229	H1	H	0.500070000097	0.721479999903	0.633090000193
230	H1	H	0.500070000097	0.221480000000	0.133090000000
231	H1	H	0.999929999903	0.278520000000	0.133090000000
232	H1	H	0.999929999903	0.778520000097	0.633090000193
233	H1	H	0.499940000193	0.278520000000	0.633090000193
234	H1	H	0.499940000193	0.778509999807	0.133090000000
235	H1	H	0.278520000000	0.133090000000	0.999940000193
236	H1	H	0.778520000097	0.633090000193	0.999920000097
237	H1	H	0.721479999903	0.133090000000	0.000070000000
238	H1	H	0.221480000000	0.633090000193	0.000060000000
239	H1	H	0.721479999903	0.866909999807	0.999929999903
240	H1	H	0.221480000000	0.366910000000	0.999929999903
241	H1	H	0.278520000000	0.866909999807	0.000070000000
242	H1	H	0.778520000097	0.366910000000	0.000050000000
243	H1	H	0.133090000000	0.000060000000	0.721479999903
244	H1	H	0.133090000000	0.500070000097	0.221480000000
245	H1	H	0.633090000193	0.000060000000	0.221480000000
246	H1	H	0.633090000193	0.500070000097	0.721479999903
247	H1	H	0.133090000000	0.999929999903	0.278520000000
248	H1	H	0.133090000000	0.499940000193	0.778520000097
249	H1	H	0.633090000193	0.999929999903	0.778520000097
250	H1	H	0.633090000193	0.499940000193	0.278520000000
251	H1	H	0.866909999807	0.000060000000	0.278520000000
252	H1	H	0.866909999807	0.500070000097	0.778520000097
253	H1	H	0.366910000000	0.000060000000	0.778520000097
254	H1	H	0.366910000000	0.500070000097	0.278520000000
255	H1	H	0.866909999807	0.999929999903	0.721479999903
256	H1	H	0.866909999807	0.499940000193	0.221480000000
257	H1	H	0.366910000000	0.999929999903	0.221480000000
258	H1	H	0.366910000000	0.499940000193	0.721479999903
259	H2	H	0.416490000000	0.583520000097	0.916479999903
260	H2	H	0.583490000193	0.416490000000	0.916450000000
261	H2	H	0.416540000000	0.416460000000	0.083540000000
262	H2	H	0.583590000193	0.583359999807	0.083560000000
263	H2	H	0.916459999807	0.583470000097	0.416460000000
264	H2	H	0.916470000097	0.416490000000	0.583520000097
265	H2	H	0.083480000000	0.583529999903	0.583550000000
266	H2	H	0.083470000000	0.416470000000	0.416460000000
267	H2	H	0.583490000193	0.916470000097	0.416470000000
268	H2	H	0.416480000000	0.916479999903	0.583509999807
269	H2	H	0.416460000000	0.083440000000	0.416440000000
270	H2	H	0.583529999903	0.083460000000	0.583550000000

271	H2	H	0.083550000000	0.083540000000	0.083460000000
272	H2	H	0.916459999807	0.916470000097	0.083480000000
273	H2	H	0.916459999807	0.083520000000	0.916509999807
274	H2	H	0.083520000000	0.916479999903	0.916479999903
275	H3	H	0.084340000000	0.717040000193	0.500320000097
276	H3	H	0.217040000000	0.584340000193	0.500320000097
277	H3	H	0.217040000000	0.415660000000	0.499679999903
278	H3	H	0.084340000000	0.282960000000	0.499679999903
279	H3	H	0.282960000000	0.084340000000	0.499679999903
280	H3	H	0.415660000000	0.217040000000	0.499679999903
281	H3	H	0.584340000193	0.217040000000	0.500320000097
282	H3	H	0.717040000193	0.084340000000	0.500320000097
283	H3	H	0.782959999807	0.415660000000	0.500320000097
284	H3	H	0.915659999807	0.282960000000	0.500320000097
285	H3	H	0.584340000193	0.782959999807	0.499679999903
286	H3	H	0.717040000193	0.915659999807	0.499679999903
287	H3	H	0.915659999807	0.717040000193	0.499679999903
288	H3	H	0.782959999807	0.584340000193	0.499679999903
289	H3	H	0.282960000000	0.915659999807	0.500320000097
290	H3	H	0.415660000000	0.782959999807	0.500320000097
291	O1	O	0.170470000000	0.000000000000	0.095300000000
292	O1	O	0.170470000000	0.500000000000	0.595300000000
293	O1	O	0.670470000097	0.000000000000	0.595300000000
294	O1	O	0.670470000097	0.500000000000	0.095300000000
295	O1	O	0.829529999903	0.000000000000	0.095300000000
296	O1	O	0.829529999903	0.500000000000	0.595300000000
297	O1	O	0.329530000000	0.000000000000	0.595300000000
298	O1	O	0.329530000000	0.500000000000	0.095300000000
299	O1	O	0.829529999903	0.000000000000	0.904700000000
300	O1	O	0.829529999903	0.500000000000	0.404700000000
301	O1	O	0.329530000000	0.000000000000	0.404700000000
302	O1	O	0.329530000000	0.500000000000	0.904700000000
303	O1	O	0.170470000000	0.000000000000	0.904700000000
304	O1	O	0.170470000000	0.500000000000	0.404700000000
305	O1	O	0.670470000097	0.000000000000	0.404700000000
306	O1	O	0.670470000097	0.500000000000	0.904700000000
307	O1	O	0.095300000000	0.170470000000	0.000000000000
308	O3	O	0.095300000000	0.670470000097	0.500000000000
309	O3	O	0.595300000000	0.170470000000	0.500000000000
310	O1	O	0.595300000000	0.670470000097	0.000000000000
311	O1	O	0.095300000000	0.829529999903	0.000000000000
312	O3	O	0.095300000000	0.329530000000	0.500000000000
313	O3	O	0.595300000000	0.829529999903	0.500000000000
314	O1	O	0.595300000000	0.329530000000	0.000000000000
315	O1	O	0.904700000000	0.829529999903	0.000000000000
316	O3	O	0.904700000000	0.329530000000	0.500000000000
317	O3	O	0.404700000000	0.829529999903	0.500000000000
318	O1	O	0.404700000000	0.329530000000	0.000000000000
319	O1	O	0.904700000000	0.170470000000	0.000000000000
320	O3	O	0.904700000000	0.670470000097	0.500000000000
321	O3	O	0.404700000000	0.170470000000	0.500000000000
322	O1	O	0.404700000000	0.670470000097	0.000000000000
323	O1	O	0.000000000000	0.095300000000	0.170470000000
324	O1	O	0.000000000000	0.595300000000	0.670470000097

325	O1	O	0.500000000000	0.095300000000	0.670470000097
326	O1	O	0.500000000000	0.595300000000	0.170470000000
327	O1	O	0.000000000000	0.095300000000	0.829529999903
328	O1	O	0.000000000000	0.595300000000	0.329530000000
329	O1	O	0.500000000000	0.095300000000	0.329530000000
330	O1	O	0.500000000000	0.595300000000	0.829529999903
331	O1	O	0.000000000000	0.904700000000	0.829529999903
332	O1	O	0.000000000000	0.404700000000	0.329530000000
333	O1	O	0.500000000000	0.904700000000	0.329530000000
334	O1	O	0.500000000000	0.404700000000	0.829529999903
335	O1	O	0.000000000000	0.904700000000	0.170470000000
336	O1	O	0.000000000000	0.404700000000	0.670470000097
337	O1	O	0.500000000000	0.904700000000	0.670470000097
338	O1	O	0.500000000000	0.404700000000	0.170470000000
339	O1	O	0.000000000000	0.170470000000	0.904700000000
340	O1	O	0.000000000000	0.670470000097	0.404700000000
341	O1	O	0.500000000000	0.170470000000	0.404700000000
342	O1	O	0.500000000000	0.670470000097	0.904700000000
343	O1	O	0.000000000000	0.829529999903	0.904700000000
344	O1	O	0.000000000000	0.329530000000	0.404700000000
345	O1	O	0.500000000000	0.829529999903	0.404700000000
346	O1	O	0.500000000000	0.329530000000	0.904700000000
347	O1	O	0.000000000000	0.829529999903	0.095300000000
348	O1	O	0.000000000000	0.329530000000	0.595300000000
349	O1	O	0.500000000000	0.829529999903	0.595300000000
350	O1	O	0.500000000000	0.329530000000	0.095300000000
351	O1	O	0.000000000000	0.170470000000	0.095300000000
352	O1	O	0.000000000000	0.670470000097	0.595300000000
353	O1	O	0.500000000000	0.170470000000	0.595300000000
354	O1	O	0.500000000000	0.670470000097	0.095300000000
355	O1	O	0.170470000000	0.095300000000	0.000000000000
356	O3	O	0.170470000000	0.595300000000	0.500000000000
357	O3	O	0.670470000097	0.095300000000	0.500000000000
358	O1	O	0.670470000097	0.595300000000	0.000000000000
359	O1	O	0.829529999903	0.095300000000	0.000000000000
360	O3	O	0.829529999903	0.595300000000	0.500000000000
361	O3	O	0.329530000000	0.095300000000	0.500000000000
362	O1	O	0.329530000000	0.595300000000	0.000000000000
363	O1	O	0.829529999903	0.904700000000	0.000000000000
364	O3	O	0.829529999903	0.404700000000	0.500000000000
365	O3	O	0.329530000000	0.904700000000	0.500000000000
366	O1	O	0.329530000000	0.404700000000	0.000000000000
367	O1	O	0.170470000000	0.904700000000	0.000000000000
368	O3	O	0.170470000000	0.404700000000	0.500000000000
369	O3	O	0.670470000097	0.904700000000	0.500000000000
370	O1	O	0.670470000097	0.404700000000	0.000000000000
371	O1	O	0.095300000000	0.000000000000	0.829529999903
372	O1	O	0.095300000000	0.500000000000	0.329530000000
373	O1	O	0.595300000000	0.000000000000	0.329530000000
374	O1	O	0.595300000000	0.500000000000	0.829529999903
375	O1	O	0.095300000000	0.000000000000	0.170470000000
376	O1	O	0.095300000000	0.500000000000	0.670470000097
377	O1	O	0.595300000000	0.000000000000	0.670470000097
378	O1	O	0.595300000000	0.500000000000	0.170470000000

379	O1	O	0.904700000000	0.000000000000	0.170470000000
380	O1	O	0.904700000000	0.500000000000	0.670470000097
381	O1	O	0.404700000000	0.000000000000	0.670470000097
382	O1	O	0.404700000000	0.500000000000	0.170470000000
383	O1	O	0.904700000000	0.000000000000	0.829529999903
384	O1	O	0.904700000000	0.500000000000	0.329530000000
385	O1	O	0.404700000000	0.000000000000	0.329530000000
386	O1	O	0.404700000000	0.500000000000	0.829529999903
387	O2	O	0.056100000000	0.943900000000	0.943900000000
388	O2	O	0.056100000000	0.443900000000	0.443900000000
389	O2	O	0.556100000000	0.943900000000	0.443900000000
390	O2	O	0.556100000000	0.443900000000	0.943900000000
391	O2	O	0.943900000000	0.056100000000	0.943900000000
392	O2	O	0.943900000000	0.556100000000	0.443900000000
393	O2	O	0.443900000000	0.056100000000	0.443900000000
394	O2	O	0.443900000000	0.556100000000	0.943900000000
395	O2	O	0.943900000000	0.943900000000	0.056100000000
396	O2	O	0.943900000000	0.443900000000	0.556100000000
397	O2	O	0.443900000000	0.943900000000	0.556100000000
398	O2	O	0.443900000000	0.443900000000	0.056100000000
399	O2	O	0.056100000000	0.056100000000	0.056100000000
400	O2	O	0.056100000000	0.556100000000	0.556100000000
401	O2	O	0.556100000000	0.056100000000	0.556100000000
402	O2	O	0.556100000000	0.556100000000	0.056100000000
403	O1	O	0.943900000000	0.056100000000	0.056100000000
404	O1	O	0.943900000000	0.556100000000	0.556100000000
405	O1	O	0.443900000000	0.056100000000	0.556100000000
406	O1	O	0.443900000000	0.556100000000	0.056100000000
407	O1	O	0.056100000000	0.943900000000	0.056100000000
408	O1	O	0.056100000000	0.443900000000	0.556100000000
409	O1	O	0.556100000000	0.943900000000	0.556100000000
410	O1	O	0.556100000000	0.443900000000	0.056100000000
411	O1	O	0.943900000000	0.943900000000	0.943900000000
412	O1	O	0.943900000000	0.443900000000	0.443900000000
413	O1	O	0.443900000000	0.943900000000	0.443900000000
414	O1	O	0.443900000000	0.443900000000	0.943900000000
415	O1	O	0.056100000000	0.056100000000	0.943900000000
416	O1	O	0.056100000000	0.556100000000	0.443900000000
417	O1	O	0.556100000000	0.056100000000	0.443900000000
418	O1	O	0.556100000000	0.556100000000	0.943900000000
419	Zr1	Zr	0.119890000000	0.000000000000	0.000000000000
420	Zr1	Zr	0.119890000000	0.500000000000	0.500000000000
421	Zr1	Zr	0.619890000193	0.000000000000	0.500000000000
422	Zr1	Zr	0.619890000193	0.500000000000	0.000000000000
423	Zr1	Zr	0.880109999807	0.000000000000	0.000000000000
424	Zr1	Zr	0.880109999807	0.500000000000	0.500000000000
425	Zr1	Zr	0.380110000000	0.000000000000	0.500000000000
426	Zr1	Zr	0.380110000000	0.500000000000	0.000000000000
427	Zr1	Zr	0.000000000000	0.119890000000	0.000000000000
428	Zr1	Zr	0.000000000000	0.619890000193	0.500000000000
429	Zr1	Zr	0.500000000000	0.119890000000	0.500000000000
430	Zr1	Zr	0.500000000000	0.619890000193	0.000000000000
431	Zr1	Zr	0.000000000000	0.880109999807	0.000000000000
432	Zr1	Zr	0.000000000000	0.380110000000	0.500000000000

Zr1	Zr	0.500000000000	0.880109999807	0.500000000000
Zr1	Zr	0.500000000000	0.380110000000	0.000000000000
Zr1	Zr	0.000000000000	0.000000000000	0.119890000000
Zr1	Zr	0.000000000000	0.500000000000	0.619890000193
Zr1	Zr	0.500000000000	0.000000000000	0.619890000193
Zr1	Zr	0.500000000000	0.500000000000	0.119890000000
Zr1	Zr	0.000000000000	0.000000000000	0.880109999807
Zr1	Zr	0.000000000000	0.500000000000	0.380110000000
Zr1	Zr	0.500000000000	0.000000000000	0.380110000000
Zr1	Zr	0.500000000000	0.500000000000	0.880109999807
