# Supplementary material for: Defect-induced tuning of polarity-dependent adsorption in hydrophobic–hydrophilic UiO-66
Source: Commun Chem. 2022 Oct 7;5:120. doi: 10.1038/s42004-022-00742-z (PMC9814431; doi:10.1038/s42004-022-00742-z)
Supplement: Supplementary file 1 — Calero_PR File [file 42004_2022_742_MOESM1_ESM.pdf]

nature portfolio

Peer Review File

Defect-induced tuning of polarity-dependent adsorption in hydrophobic-hydrophilic UiO-66

Open Access This file is licensed under a Creative Commons Attribution 4.0 International License, which permits use, sharing, adaptation, distribution and reproduction in any medium or format, as long as you give appropriate credit to the original author(s) and the source, provide a link to the Creative Commons license, and indicate if changes were made. In the cases where the authors are anonymous, such as is the case for the reports of anonymous peer reviewers, author attribution should be to 'Anonymous Referee' followed by a clear attribution to the source work. The images or other third party material in this file are included in the article's Creative Commons license, unless indicated otherwise in a credit line to the material. If material is not included in the article's Creative Commons license and your intended use is not permitted by statutory regulation or exceeds the permitted use, you will need to obtain permission directly from the copyright holder. To view a copy of this license, visit http://creativecommons.org/licenses/by/4.0/.

Reviewers' comments:

Reviewer #1 (Remarks to the Author):

The manuscript from Jajko et al. details the gas adsorption characterises of UiO-66 with varying degree of defect sites that dictates the hydrophobicity of the framework. This work undoubtedly builds upon previous work on water adsorption for the same systems [10.1016/j.micromeso.2021.111555].

The authors have produced a detailed manuscript characterising the approaches to simulate and observe the adsorption of several gases within these materials and a microscopic understanding of the trends observed. While I recognise the importance of this study to delineate the behaviour of this widely studied MOF for different defect concentrations, I am not convinced that it is entirely original or will influence thinking in the wider chemical community.

This remains important work and I believe this study should be considered for publication in a more specialised journal.

I have several points, detailed below, that the authors should consider before publication:

Major

How are the configurations for the DFT+D calculations chosen? Can this limited sampling capture the most favourable adsorption sites? At what temperature is the Gibbs free energy calculated and is the convergence criteria tight enough to produce accurate vibration information?

Cell lengths (Table S5) are 20.7 Å, which is less than twice the cut-off distance of 12 Å. This may lead to numerical errors in the simulations.

The average occupation profiles provide a nice graphical description of the differences but subtleties are difficult to observe. This information could be analysed to produce pore location isotherms as described by Neimark and Evans [10.1021/jacs.9b00906, 10.26434/chemrxiv-2022-ls3rp]

I could not follow the dehydration and dehydroxylation employed by the thermal treatment for the FTIR experiments. How does this relate to the varying defect concentration of the materials?

Minor

Fig. 3 plots are displayed with inconsistent pressure axes (pressure and partial pressure).

The naming scheme makes it difficult to discern which labels refer to simulation and experiment.

Many figures of the IR spectra are missing the y-axis.

Please share the input and data files for the simulations used to ensure reproducibility.

Reviewer #2 (Remarks to the Author):

The paper studies the very well known case of UiO-66 with varying degrees of defectivity. From the initial works by Lillerud, later followed up by Serre, and especially from the studies by Greg Shearer, we all know that indeed UiOs may exist with varying amounts of hydroxyl groups: the less linkers, the more hydrophilic defects.
Logically, when recording adsorption isotherms, with MeOH as a hydrophilic probe, and CO2 as a more hydrophobic reporter molecule, such trends show up.

I cannot say that I acquired any new insight when reading this paper. It is a fine routine study, but certainly not up to the level of Nature Communications, unfortunately.

Reviewers' comments:

Reviewer #1 (Remarks to the Author):

The manuscript from Jajko et al. details the gas adsorption characterises of UiO-66 with varying degree of defect sites that dictates the hydrophobicity of the framework. This work undoubtedly builds upon previous work on water adsorption for the same systems [10.1016/j.micromeso.2021.111555].

The authors have produced a detailed manuscript characterising the approaches to simulate and observe the adsorption of several gases within these materials and a microscopic understanding of the trends observed. While I recognise the importance of this study to delineate the behaviour of this widely studied MOF for different defect concentrations, I am not convinced that it is entirely original or will influence thinking in the wider chemical community.

This remains important work and I believe this study should be considered for publication in a more specialised journal.

I have several points, detailed below, that the authors should consider before publication:

We thank the referee for the careful reading of our manuscript and the positive assessment of our work.

Major

How are the configurations for the DFT+D calculations chosen? Can this limited sampling capture the most favourable adsorption sites? At what temperature is the Gibbs free energy calculated and is the convergence criteria tight enough to produce accurate vibration information?

We select the conditions for the DFT calculations as a compromise between the ideal mapping of the adsorption sites and the computational time. As can be observed on the average occupation profiles, there are many possible positions for carbon dioxide and methanol molecules which means that there are several local minima of energy. We selected the locations to be sure that they are representative enough to estimate the difference of adsorption energy in the localization close to the linker or close to the zirconium cluster. Therefore, the locations were selected based on the initial Monte Carlo calculations and then transferred to the DFT + D calculations. Following this procedure, the approach is able to capture the most favorable adsorption sites.

To compute the Gibbs free energy, we kept same temperature as for the IR experiments for carbon dioxide and methanol. In our opinion, the convergence criteria is tight enough to produce accurate vibration information. However, this was checked by comparisons between simulated and experimental spectra, on the basis of the bands whose origin we were sure of without the need for calculations.

Cell lengths (Table S5) are 20.7 Å, which is less than twice the cut-off distance of 12 Å. This may lead to numerical errors in the simulations.

We thank to the referee for pointing out this question. We agree that 20.7004 Å is too low for calculations (less than twice the cut-off distance of 12 Å). Unfortunately, an error appeared in the Table S5 and this one dimension was given for the 1x1x1 cell, not for the 2x2x2 as it was written in the caption. As we explain in the methods section, we used a simulation box of 2x2x2 cells in order to avoid numerical errors during the simulations. The actual dimensions of the supercell in which the calculations were performed are a = b = c = 41.4008 Å. Table S5 is now correct.

The average occupation profiles provide a nice graphical description of the differences, but subtleties are difficult to observe. This information could be analysed to produce pore location isotherms as described by Neimark and Evans [10.1021/jacs.9b00906, 10.26434/chemrxiv-2022-ls3rp]

We appreciate this suggestion, and we agree that analyzing the pore filling of the structures provides a valuable information. In fact, we use average occupation profiles (AOP) for this regard. For sake of clarity, we kept the profiles in the ESI, but the main text contains the discussion coming from the analysis of the AOP. To help the reader, we have added five new figures (S4, S11, S15, S18, and S22) to the ESI. In those figures we show, following the work of Neimark et al., the adsorption isotherm of the target gas, and the AOPs for the selected pressure. In this way, it is possible to visualize the filling of the two pores at the same time and to observe the filling order. We prefer to use AOP instead of snapshots of the simulation, because they provide accurate information that do not depend on a given MC cycle but from the whole simulation. Figures S5, S12, S16, S19, and S23, depict the AOPs for the structures with defects. We have added a paragraph to the text explaining this procedure:

"*Figure S4 depicts the adsorption isotherm for the ideal structure with three points marked on the figure. Then, the AOPs for the selected pressure are shown. Figure S5 depicts the AOPs at the selected pressure but for all the studied structures (ideal and defective)*"

I could not follow the dehydration and dehydroxylation employed by the thermal treatment for the FTIR experiments. How does this relate to the varying defect concentration of the materials?

To clarify this point, we have added a new figure to the ESI (figure S25). Dehydroxylation can be observed most clearly in Figure S24 in ESI, where one can observe a disappearance of the OH groups located in the zirconium cluster (IR bands at 730 and 680 cm^{-1}) and in coordination of the linker, observed as the increase of the 745 cm^{-1}. For a clearer observation of dehydration and dehydroxylation caused by thermal treatment in the range of O-H stretching vibrations, we added an additional figure to ESI (S25, also added below): spectra after activation at 300 C. It can be seen that for the sample with the highest number of defects, the 3663 $cm^{-1}$and 3674 cm^{-1} bands disappear completely.

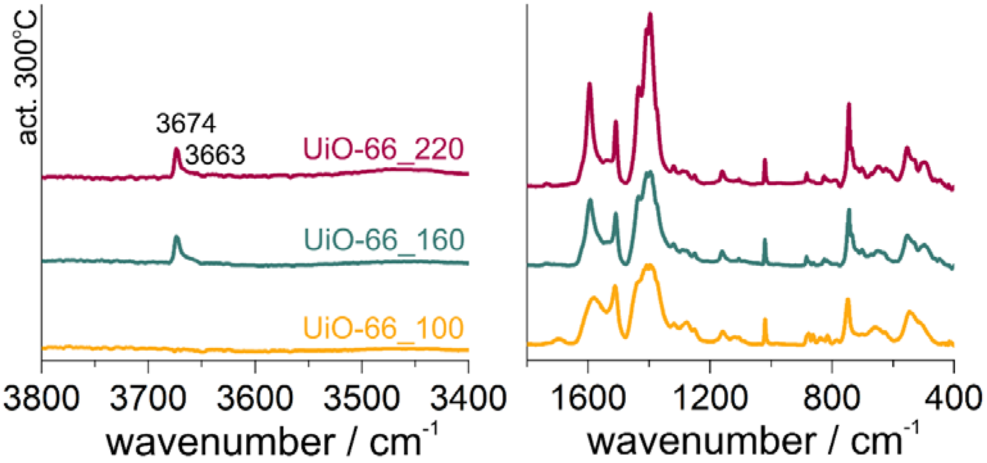

Minor

Fig. 3 plots are displayed with inconsistent pressure axes (pressure and partial pressure).

We thank the reviewer for pointing out this error. We changed the scale in Figure 3.

The naming scheme makes it difficult to discern which labels refer to simulation and experiment.

We know that working with many sets of data can lead to confusion to the reader. For this reason, we have separated the calculations and experiments in each figure, so that the reader can distinguish between experiment and calculations. As for the names of the samples, we deliberately used the scheme from our previous work so as not to create confusion. However, we explain the labels for the simulations in the method section:

"For clarity, we used the same labels, where UiO-66_0 represents the defect-free structure, while UiO-66_1, UiO-66_2, UiO-66_8, and UiO-66_32 have one, two, eight, and thirty-two 1,4-bdc linkers removed in the 2×2×2 supercell."

and the labels for the experimental samples at the beginning of the results and discussion section:

"Thus, the material synthesized at 220°C (UiO-66_200) was assumed to be ideal, while the ones synthesized at 160°C (UiO-66_160) and 100°C (UiO-66_100) contained some defects."

Many figures of the IR spectra are missing the y-axis.

All IR spectra are normalized to the same band as mentioned in the text, hence on the y axis we have "normalized absorbance" in unit a. u. As the reviewer suggests, we add it to the figures.

Please share the input and data files for the simulations used to ensure reproducibility.

All .cif files in which the calculations were performed are added to the manuscript, while in the ESI and the main text we provided all the information needed to reproduce our calculations (software, number of cycles, cell size, temperature, point loads, L-J parameters and mixing rules etc.).

Reviewer #2 (Remarks to the Author):

The paper studies the very well known case of UiO-66 with varying degrees of defectivity. From the initial works by Lillerud, later followed up by Serre, and especially from the studies by Greg Shearer, we all know that indeed UiOs may exist with varying amounts of hydroxyl groups: the less linkers, the more hydrophilic defects.

Logically, when recording adsorption isotherms, with MeOH as a hydrophilic probe, and CO2 as a more hydrophobic reporter molecule, such trends show up. I cannot say that I acquired any new insight when reading this paper. It is a fine routine study, but certainly not up to the level of Nature Communications, unfortunately.

We thank the reviewer for the critical read of our manuscript, but we cannot agree to consider our work as fine routine. This is a rigorous work, where we combine state-of the art methodology for experiments, simulations, and DFT to provide results, evidence, and explanations on the behavior of polar and non-polar gases in ideal and defective structures of UiO-66 metal organic framework. We would agree with the referee that this manuscript does not fit the Nature Communications profile, not because of its quality but because of its subject matter in chemistry, but we believe it fits perfectly in Communications Chemistry.

REVIEWERS' COMMENTS:

Reviewer #1 (Remarks to the Author):

I request the authors consider including the following change to the manuscript main text before publication.

*Details of how the initial configurations were chosen for the DFT+D calculations.

Finally, I implore the authors to share the simulation data (input files etc) with the community beyond the standard “reasonable request” statement. For example, the files could be uploaded to a repository such as Zenodo.

Reviewer #1 (Remarks to the Author):

I request the authors consider including the following change to the manuscript main text before publication.

*Details of how the initial configurations were chosen for the DFT+D calculations.

Response: To set the initial configurations for performing the DFT+D calculations we previously conducted Monte Carlo simulations. The equilibrated positions obtained after those simulations were then used as the starting positions for the DFT+D calculations. We added this details to the main text:

“The initial configurations for the molecules were selected on the basis of the preliminary Monte Carlo calculations.”

Finally, I implore the authors to share the simulation data (input files etc) with the community beyond the standard "reasonable request" statement. For example, the files could be uploaded to a repository such as Zenodo.

Response: We thank the suggestion of the referee and we will upload our files to the Zenodo repository as soon as the manuscript has a assigned DOI.
